# Accessing carbon, boron and germanium spiro stereocentres in a unified catalytic enantioselective approach

**DOI:** 10.1038/s41929-025-01352-3

**Published:** 2025-06-12

**Authors:** Yi-Xuan Cao, Anne-Sophie Chauvin, Shuo Tong, Layth Alama, Nicolai Cramer

**Affiliations:** 1 Laboratory of Asymmetric Catalysis and Synthesis, Institute of Chemical Sciences and Engineering, Ecole Polytechnic Fédérale de Lausanne, Lausanne, Switzerland; 2 Group of Coordination Chemistry, Institute of Chemical Sciences and Engineering, École Polytechnique Fédérale de Lausanne, Lausanne, Switzerland; 3 MOE Key Laboratory of Bioorganic Phosphorus and Chemical Biology, Department of Chemistry, Tsinghua University, Beijing, China

**Keywords:** Asymmetric catalysis, Synthetic chemistry methodology, Optical materials

## Abstract

Achieving substrate generality in asymmetric catalysis remains a long-standing goal, particularly for the selective construction of chiral heteroatoms. Compared with carbon, sulfur, phosphorus and silicon stereogenic centres, methods for the construction of their boron and germanium congeners remain very scarce. Chiral (hetero) spirocycles are of relevance in several research domains. Methods effective for constructing carbon-centred chiral spirocycles do not translate to boron and germanium, leaving these chiral centres unexplored. We describe a unified strategy for constructing carbon, boron and germanium-centred chiral spirocyclic skeletons via enantioselective hetero [2+2+2] cycloaddition of a bis-alkyne with a nitrile. A chiral designer Ni(0) *N*-heterocyclic carbene complex enables the required long-range enantioinduction. The resulting enantio-enriched spirocycles feature a pyridine motif, making them exploitable for ligand design and functional materials featuring attractive photophysical and chiroptical properties.

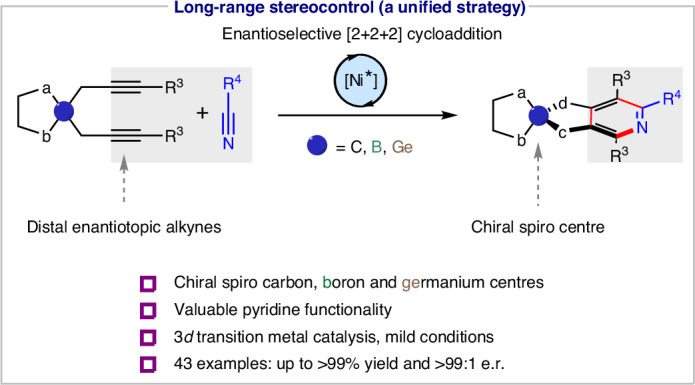

## Abstract (web summary)

General methods to access stereogenic centres of different heteroatoms are limited. Now the nickel-catalysed enantioselective construction of carbon, boron and germanium-centred chiral spirocyclic frameworks via a hetero [2+2+2] cycloaddition of a bis-alkyne with a nitrile is presented.

## Main

**Fig. 1:**
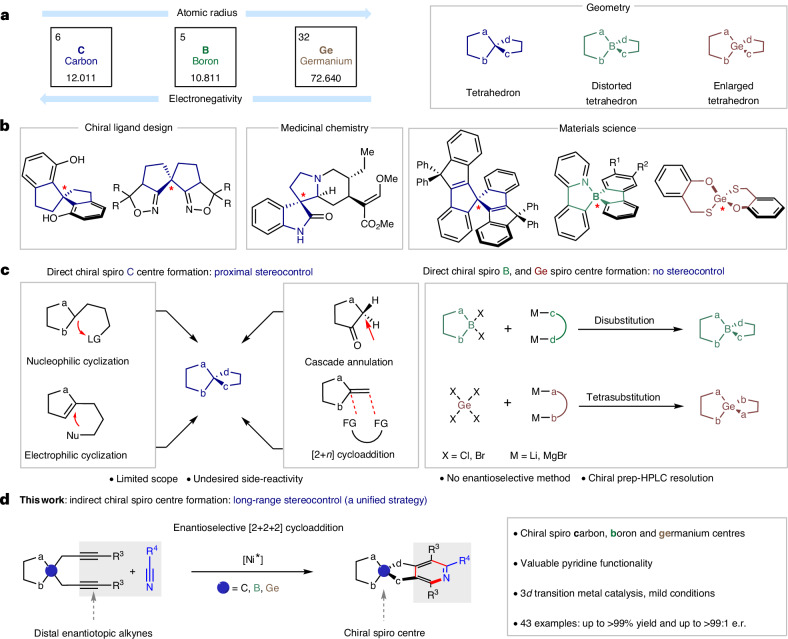
Catalytic enantioselective construction of chiral spirocyclic skeletons. **a**, Distinctive chemical–physical properties of carbon, boron and germanium-centred chiral spiro-skeletons. **b**, Examples of applications of chiral spirocycles in different fields. **c**, Established methodologies and challenges for enantioselective construction of chiral spirocycles. LG, leaving group; FG, functional group. **d**, This work: nickel-catalysed enantioselective hetero [2+2+2] cycloaddition for remote construction of chiral spirocycles.

Developing substrate generality is a central aim and grand challenge in asymmetric catalysis^1,2^, particularly in constructing quaternary stereogenic centres and their analogous main group heteroatom stereogenic centres. Methods for their assembly have gained great attention due to the intriguing chemical, physical, biological and stereoelectronic properties^3^ of the resulting compounds (Fig. 1a). For instance, carbon, boron and germanium-centred chiral spirocycles are frequently found in chiral ligand design^4^, biologically active compounds^5^ and materials^6–10^ (Fig. 1b). Typically, accessing different chiral central elements requires distinctive synthetic strategies. In contrast to the recent advances to catalytically construct sulfur^11^, phosphorus^12^ and silicon^13,14^ stereogenic centres, methods to access other hetero-element stereocentres—namely boron^15–18^ and germanium^19–21^—remain so far largely underdeveloped due to unique synthetic challenges. For instance, tetracoordinate boron compounds possess a dative bond resulting in distorted tetrahedral geometry^22^ and have potential lability. The longer and more labile germanium–carbon bonds in organogermanium compounds feature an enlarged tetrahedral geometry^23^ (Fig. 1a). These differences render most current strategies for the construction of carbon, sulfur, phosphorus and silicon stereogenic centres inapplicable for their boron and germanium congeners. Despite the considerable effort taken to explore the structural diversity of carbon-, boron- and germanium-centred spirocycles, it remains notoriously difficult to construct these chiral spirocyclic skeletons, particularly in a unified strategy or in an enantioselective manner. Methods to construct carbon-centred chiral spirocycles typically involve direct bond formations at the spirocentre. An enantioselective construction is achieved by differentiating functional groups near the spiro carbon atom via proximal stereocontrol^24,25^ (Fig. 1c). The performance of the chiral catalysts is generally restricted to specific quaternary chiral spirocentres, often resulting in limited scope and reduced applicability. There are further drawbacks in the construction of scaffolds featuring multiple functionalities where stereochemical information extends far from the spirocentre^26,27^. Accessing such elaborate chiral spirocyclic skeletons usually requires lengthy routes such as modifying synthesized prochiral spirocycles^25^, or racemate resolution via chiral high-performance liquid chromatography^28^. Moreover, established strategies cannot simply be extended to access chiral spirocycles with different central heteroatoms such as boron and germanium due to their unique reactivities (Fig. 1a,c)^29,30^. The enantioselective construction of boron- and germanium-centred chiral spiro-skeletons thus remains a formidable challenge.

A key challenge in the development of a unified strategy for the construction of carbon, boron and germanium-centred spirocyclic skeletons lies in the identification of a method of stereocontrol tolerant to distinctive tetrahedral geometries of the spirocyclic centre and to diverse functionalities. A remote desymmetrizing ring closure seemed to be a promising strategy to construct the targeted chiral spirocycles. Such an approach involves introducing an enantiotopic bis-substitution on the carbon, boron and germanium-based cyclic substrates. The enantioselective ring closure involving both enantiotopic substituents selectively creates the spiro stereogenic centre at a remote position (Fig. 1d). Examples of such strategies for the asymmetric construction of spirocycles are rare^31,32^ and largely exhibit limited scope. We turned to transition-metal-catalysed [2+2+2] cycloadditions^33–35^ as an exploitable strategy for the desymmetrizing ring closure via arene formation. Integrating chiral spiro-skeletons formation with the formation of a pyridine from two alkynes and a nitrile bears considerable value, this is due to the pyridine motif substantially enhances utility through its involvement in downstream applications in ligand or material design^36,37^. We therefore considered an enantioselective hetero [2+2+2] cycloaddition^38–40^ of two distal enantiotopic bis-alkynes with a nitrile as a promising strategy for constructing chiral spiro-skeletons if three key challenges could be addressed (Fig. 1d). First, the different side reactions of the bis-alkyne substrates such as alkyne cyclotrimerization or oligomerization need to be suppressed^41^. Second, as the stereodifferencing element is remote from the hetero [2+2+2] cycloaddition reaction site, a chiral catalyst with the ability to achieve efficient enantio-discrimination between the distal enantiotopic alkynes is required^42^. Third, the catalyst needs to accommodate the distorted and enlarged geometries of the boron and germanium centres while also working under mild reaction conditions to ensure survivability of these more fragile bonds.

Leveraging our experience in designing and applying chiral *N*-heterocyclic carbenes (NHCs) in asymmetric catalysis^43,44^, here we prepared *C*_2_-symmetric chiral Ni(0)NHC styrene complexes that are able to selectively catalyse hetero [2+2+2] cycloadditions assembling carbon-, boron- and germanium-centred spirocyclic skeletons via long-range enantioinductions. Mechanistic insights point towards a heterocoupling mechanism and allow understanding of the enantiodetermining step. Moreover, the rapid assembly of complex chiral compounds with attractive photophysical and chiroptical properties underscore the applicability for functional materials.

## Results

### Development of the enantioselective [2+2+2] cycloaddition

**Fig. 2:**
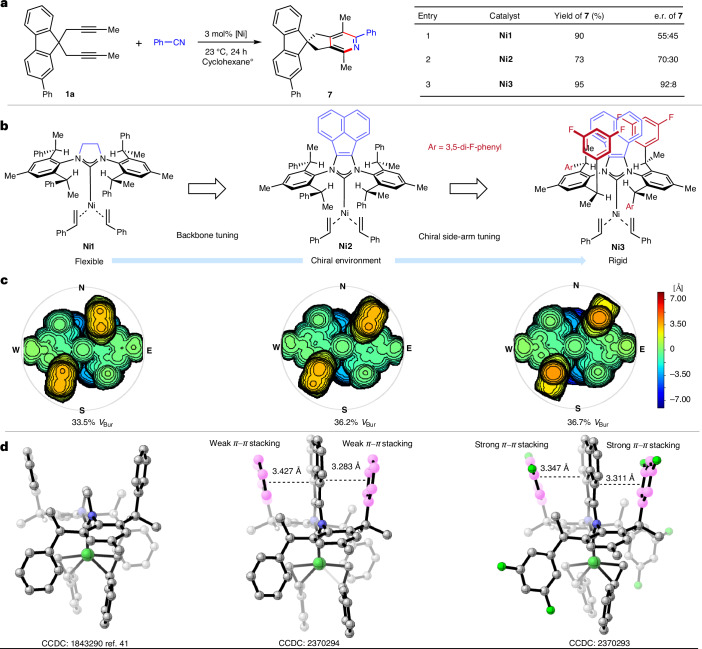
Catalyst development for enantioselective construction of chiral spiro-skeleton. **a**, Optimization table of target enantioselective [2+2+2] cycloaddition. **b**, Catalyst development. **c**, Steric maps of catalysts **Ni1**, **Ni2** and **Ni3**. % V_Bur_, calculated buried volumes. **d**, Crystal structures of catalyst **Ni1**, **Ni2** and **Ni3**, non-stereogenic hydrogen atoms are omitted for clarity.

Incorporating rigid, chirality-inducing spirocycles near exploitable motifs such as fluorene—which is often used for materials—is highly desirable^45^. Thus, fluorene substrate **1a** and benzonitrile were selected as substrates for the targeted hetero [2+2+2] cycloaddition. Employing just 3 mol% of chiral catalyst **Ni1** with a simple saturated ethylene bridge resulted in the formation of product **7** in excellent yield but with a very low level of enantioinduction (Fig. 2). By installing the acenaphthylene backbone (**Ni2**), we observed an improvement in catalytic performance and product **7** was formed with 70:30 e.r. (Fig. 2). Catalyst **Ni3**, which incorporated fluorine atoms into the chiral side arms further enhanced catalyst performance and gave **7** in 95% yield and a selectivity of 92:8 e.r. (Fig. 2). To gain a better understanding of how steric and electronic modulation in nickel–NHC complex influences enantioselectivity, we analysed the crystal structures of **Ni1** (ref. ^46^), **Ni2** and **Ni3**. The chiral side arm of **Ni1** complex is relatively flexible. Compared with **Ni2** and **Ni3**, it has a different chiral pocket and less buried volume. However, we observed no substantial visual differences in the chiral pockets of **Ni2** and **Ni3**. Furthermore, attempts to improve the design of **Ni2**, the incorporation of 3,5-dimethyl substituents to enhance the steric bulk of the chiral side arm resulted in a substantial drop in yield, without improvement in enantioselectivity (Supplementary Table 2, **L7**). It therefore became evident that simply a more confined microenvironment is unlikely to improve selectivity. Regarding electronic effects, weak *π*–*π* stacking was observed between the chiral side arm and the acenaphthylene motif in **Ni2** complex (Supplementary Figs. 1–4), reducing the flexibility of the chiral side arm. In total, six different molecules of **Ni2** complex appear in the unit cell of the crystal (Supplementary Fig. 1), each of which exhibits various distances between the chiral side arms and the acenaphthylene backbone, indicating that the flexibility of the chiral side arms might be still relevant (Supplementary Figs. 2–4). The electron density of the chiral side arm in the **Ni3** complex was effectively reduced by the introduction of 3,5-difluoro substituents. This modulation resulted in stronger *π*–*π* stacking with the acenaphthylene backbone establishing a more defined chiral environment as depicted in the crystal structure of **Ni3** (Supplementary Figs. 5 and 6). These findings underscore that a chiral side arm displaying rigidity and stability within the *C*_2_ symmetric chiral environment is of strong relevance for achieving high levels of enantioinduction. Refer to Supplementary Tables 1–8 for detailed reaction condition optimizations.

### Substrate scope of the enantioselective [2+2+2] cycloaddition

**Fig. 3:**
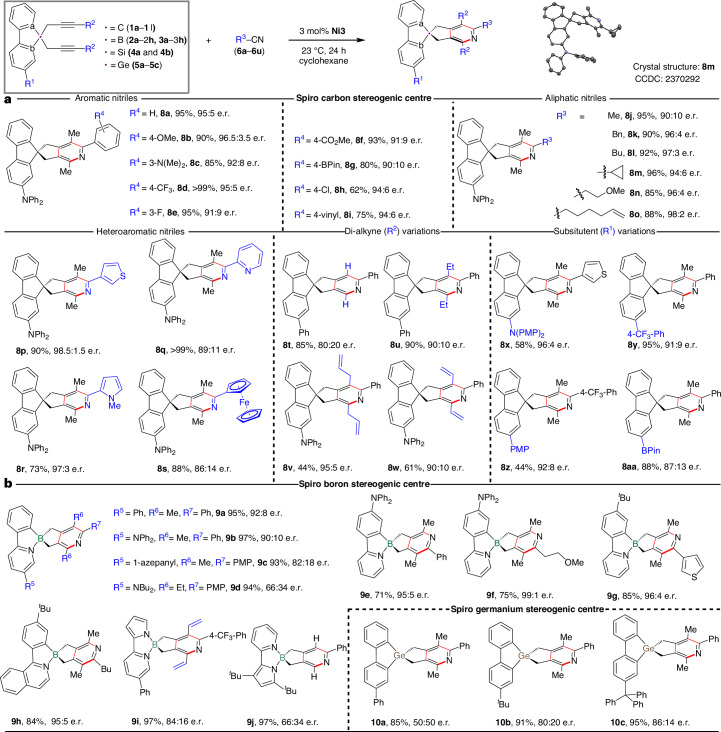
Scope of nickel–NHC-catalysed enantioselective hetero [2+2+2] cycloaddition. **a**, Examples of constructing chiral spiro carbon stereogenic centre. PMP, *p*-methoxy-phenyl. **b**, Examples of constructing chiral spiro boron and germanium stereogenic centre.

With the optimized catalyst and conditions, we first investigated the reactivity and selectivity of different aromatic nitriles in the process (Fig. 3). In this respect, both electron-donating (–OMe, –NMe_2_) and electron-withdrawing (–CF_3_, –F) groups were accommodated, providing the corresponding pyridines **8a** to **8e** in high yields and enantioselectivities. The process is mild and displays a good functional group tolerance, allowing incorporation of ester, pinacol boryl, chloro- and vinyl groups as substituents on the nitriles (**8f** to **8i**). Similarly, reactions with aliphatic nitriles including acetonitrile, phenyl acetonitrile, valeronitrile, cyclopropanecarbonitrile and 3-methoxy propionitrile provided pyridines **8j**–**8n** in high enantioselectivities. X-ray crystallographic analysis of pyridine **8m** allowed determination of its absolute configuration. Hept-6-ene-nitrile possessing a terminal olefin moiety cleanly gave **8o** in 88% yield and a 98:2 e.r. with no chain-walking products involving the olefin detected^42^. Electron-rich heteroaromatic nitriles such as 3-cyanothiophene and *N*-Me-2-cyano pyrrole yielded pyridines **8p** (90% and 98.5:1.5 e.r.) and **8r** (73% and 97:3 e.r.). The reaction with 2-cyanopyridine—an electron-poor heterocycle—yielded product **8q**, which features the sought-after 2,2′-bipyridine motif^37^. Cyano ferrocene was also effectively accommodated without catalyst poisoning or side-product formation. Concerning the alkyne pattern, ethyl substituents resulted in a similar outcome (**8u**). The challenging terminal dialkyne produced the desired desymmetrization product **8t**, albeit in somewhat reduced enantioselectivity. Functionalized bis-1,4-enyne **1i** and bis-1,3-enyne **1k** engaged in the hetero [2+2+2] cycloaddition yielding bis-olefin-bearing pyridines **8v** and **8w**, which could be exploited for subsequent polymerization reactions^47^. Variations on fluorene core substituent R^1^ include different aryl groups (**8y** and **8z**) and a bis-*p*-methoxy-phenyl (bis-PMP) amino group (**8x**) that has prominent appearances in spirofluorene materials^45^. Notably, installing a pinacol boryl group at R^1^ gave pyridine **8aa**, which can act as a broadly diversifiable intermediate via a variety of cross-couplings. Next we aimed to explore whether enantioselective hetero [2+2+2] cycloaddition was suitable for generating spiro heteroatom chirality. In this respect, we extended this process to chiral tetra-coordinated boron compounds^10^, focusing on the aza-borafluorene. **Ni3** displayed a robust performance under the same reaction conditions and delivered pyridine derivatives **9a** and **9b** with their boron stereogenic centres in excellent yields and very good enantioselectivity. A slight erosion of selectivity was found for products **9c** and **9d**, both of which have relatively smaller R^5^ substituents. Interestingly, the effect of the distorted tetrahedral geometry of the boron starting material manifests itself by different selectivities of substrates that are identical aside from having the bis-phenyl amino substituent on the phenyl ring (**9e**) or on the pyridine ring (**9b**), with the former providing a superior enantiomeric ratio. As for the previous carbon series, a diverse range of nitriles, including aromatic, aliphatic and heteroaromatic nitriles, were found to be compatible, leading to corresponding chiral spiro boron compounds **9e** to **9g** in excellent enantioselectivities. 1-(4-(^*t*^Bu)phenyl)isoquinoline-based boron dialkyne substrate **3g** displaying a complex steric environment yielded pyridine **9h** in 84% yield and 95:5 e.r. A further extension was reached with *N*,*N*′-chelated boron scaffolds^48^. These pyridyl pyrrolide chelated boron substrates were amenable to the transformation and the corresponding spirocyclic tetra-coordinated boron compounds **9i** and **9j** were formed in excellent yield and moderate to good selectivities. Our focus shifted next to the selective construction of chiral spirogermanium centre. Using germafluorene^9^ **5a** (R^1^ = Ph) as a starting material provided spiro germafluorene **10a** in 85% yield albeit in virtually racemic form. As phenyl was highly selective in the carbon as well as in the boron series as an R^1^ substituent, we attribute this lack of selectivity as a direct result of the longer C–Ge bond length and its induced distortion of the centre and altered vector of R^1^. However, the selectivity of the transformation was restored with substrate **5b** (R^1^ = ^*t*^Bu) leading to spiro germafluorene **10b** in 91% yield and 80:20 e.r. Furthermore, the reaction of **5c** (R^1^ = trityl) yielded compound **10c** in 95% yield and an increased e.r. of 86:14. Surprisingly, exposure of silicon-based analogues **4a** and **4b** to the reaction conditions caused substrate decomposition and the formation of pyridine products was not observed.

### Synthetic application of the chiral spirocycles

**Fig. 4:**
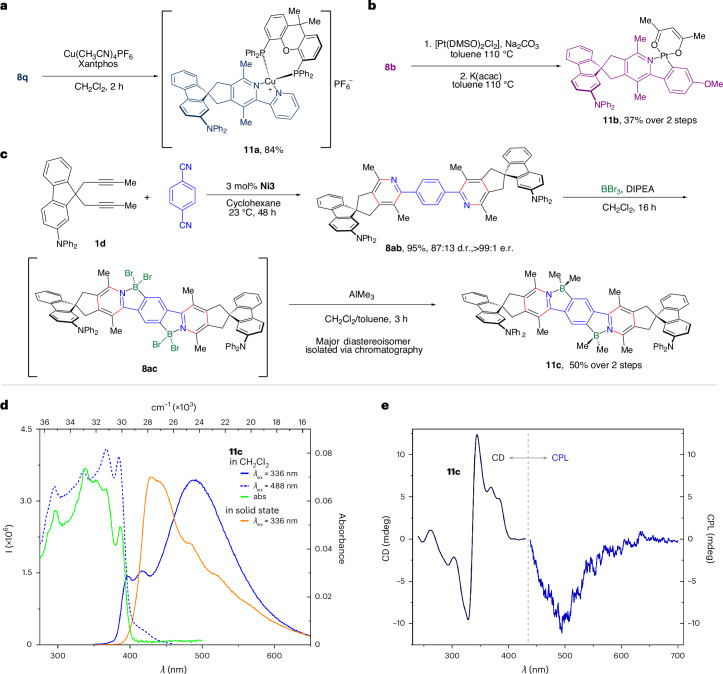
Synthetic applications. **a**, Synthesis of the Cu(I) complex **11a**. **b**, Synthesis of the Pt(II) complex **11b**. **c**, Synthesis of the boron compound **11c**. **d**, Corrected excitation (dotted line), emission (solid lines) and absorbance (solid green line, right *y*-axis scale) of compound **11c** (1 μM in CH_2_Cl_2_ or solid-state). **e**, Circular dichroism (CD) and CPL spectra of **11c** (1 μM in CH_2_Cl_2_). DIPEA, *N*,*N*-diisopropylethylamine.

Given the widespread usage of bipyridines and 2-phenyl pyridines as ligands or ligand precursors, we reasoned that the obtained spirocycles could be connected to a metal centre to impart intriguing material properties^28,37^. By using products **8q** and **8b**, we rapidly accessed the chiral variants of widely applied copper-based light-emitting electrochemical cells^49^ and platinum-based organic light-emitting diodes^50^, **11a** and **11b**, respectively (Fig. 4). The luminescent properties of **11a** and **11b** were analysed and fluorescence lifetimes and quantum yields compared well with those in the literature^49,50^ (Supplementary Figs. 8–11 and Supplementary Table 9). To further showcase the utility of the process with complex molecules displaying intriguing chiroptical properties, we prepared a chiral *C*_*2*_-symmetric bis-spirofluorene **11c** with a highly conjugated heptacyclic core in a rapid synthetic sequence. Bis-pyridine **8ab** was prepared in 95% yield with an 87:13 d.r. and >99:1 e.r. by a double enantioselective [2+2+2] heterocyclization of 1,4-dicyanobenzene with dialkyne **1d**. A double pyridine-directed electrophilic borylation gave tetrabromide **8ac**. Subsequent treatment of **8ac** with AlMe_3_ exhaustively replaced all four bromides with methyl groups leading to compound **11c** in 50% yield. The photophysical and advanced chiroptical properties of **11c** were measured (Fig. 4d). The emission spectrum of **11c** ranges from 350 nm to 650 nm, with three prominent maxima, the maximum was blue shifted in solid state by ~2,820 cm^−1^. The fluorescence lifetimes of **11c** are on the microsecond scale—similar to the known thermally activated delayed fluorescence materials^51^ (Supplementary Table 9). Notably, compound **11c** exhibited an appreciable circularly polarized luminescence (CPL) activity, with a dissymmetric factor |*g*_lum_| of 1.5 × 10^−3^ at 490 nm (Fig. 4e and Supplementary Fig. 12). The value of the important dissymmetric factor of **11c** is of similar magnitude to the ones of existing chiral organic dyes as well as helicenes^52,53^.

### Mechanistic studies

**Fig. 5:**
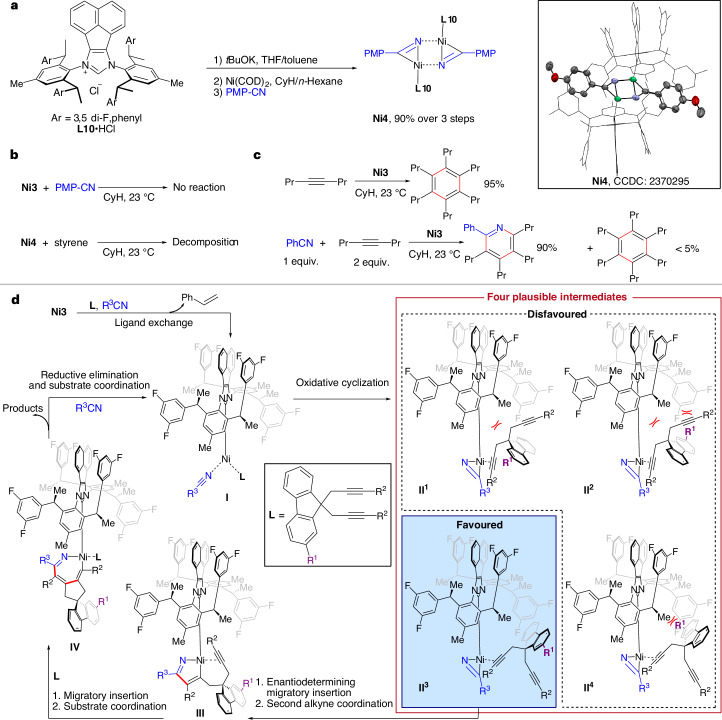
Mechanistic studies of enantioselective construction of chiral spirocycles. **a**, Synthesis of nickel nitrile complex **Ni4** (left) and its ORTEP drawing (right)**. b**, Investigations of the nitrile coordination behaviour in the presence of styrene. **c**, Comparison of pyridine formation rate versus alkyne cyclotrimerization rate. **d**, Suggested mechanism of the Ni(0)NHC-catalysed enantioselective hetero [2+2+2] cycloaddition.

The hetero [2+2+2] cycloaddition between two alkynes and one nitrile could proceed either by a homocoupling mechanism in which the two alkynes link together first, or a heterocoupling mechanism in which one of the alkynes and the nitrile link together first^34^. Correlation with a past report^54^ suggests that the heterocoupling path is indeed operative. To shed some light on the interactions of the chiral Ni(0)NHC complex with the substrates, we synthesized nickel–nitrile complex **Ni4** via complexation of **L10** with Ni(COD)_2_ and *p*-methoxybenzonitrile in 90% yield (Fig. 5a). Evidenced by crystal structure analysis, **Ni4** exhibits a dimeric structure with planar geometry. Each nitrile binds to two nickel atoms in *η*^1^ and *η*^2^ modes. The R–C–N bond angle of the former nitrile changes from 180° to 134.12° when in the complex, indicating substantial *π*-backbonding, in line with a strong *d*^8^ Ni(II) metallaazirene character^55^. **Ni4** is a competent catalyst and when used instead of **Ni3**, an identical reaction yield and enantiomeric ratio was observed (Supplementary Fig. 14), suggesting that the styrene ligands of **Ni3** probably have a pure spectator role in the later catalytic cycles. No reaction occurred between precatalyst **Ni3** and *p*-methoxybenzonitrile (Fig. 5b), indicating that nickel has a stronger affinity for styrene than nitrile and coordination of the nitrile is contingent following ligand exchange of styrene with alkyne substrate. Exposing **Ni4** to styrene did not result in formation of **Ni3**, but instead led to complex decomposition (Fig. 5b). This behaviour is further indicative of the Ni(II) character of the **Ni4** species and suggests that the formation of the metallaazirene species is irreversible^54^. In the absence of a nitrile, **Ni3** cleanly catalyses alkyne trimerization; however, the formation of pyridine is largely favoured in its presence. This indicates that pyridine formation proceeds much faster than cyclotrimerization of alkyne (Fig. 5c). With the heterocoupling mechanism operative, the enantiodetermining step is the selective migratory insertion of one of the enantiotopic alkynes into the Ni(II) metallaazirene. The crystal structure of **Ni4** shows that the nitrile substrates are perfectly sandwiched in the middle of chiral side arms with a planar geometry (Fig. 5), suggesting that the chiral side arms of the carbene can effectively discern stereochemical information from the dialkyne substrate to achieve enantioselectivity. In sum, these observations enable us to propose a mechanism for the enantioselective hetero [2+2+2] cycloaddition (Fig. 5d). To initiate a first cycle, ligand exchange occurs on **Ni3**, where a dialkyne substrate and nitrile replace both styrenes yielding intermediate **I**. Oxidative cyclization then occurs, leading to the formation of a metallaazirene intermediate. An enantiodetermining migratory insertion of the coordinated alkyne towards the metallaazirene species proceeds. The orientation of group R^1^ of the dialkyne substrate is paramount for the selectivity. The substituent R^3^ on the nitrile has much less impact on the selectivity due to its distance from the chiral side arm. The substrate-catalyst complex adopts an orientation in which the substituent R^1^ is favourably placed to the least hindered position leading preferentially to intermediate **II**^3^ over the more congested alternative orientations **II**^1^, **II**^2^ and **II**^4^. Following the migratory insertion step, the coordination of the second alkyne moiety leads to intermediate **III**. Subsequent second migratory insertion leads to the formation of a seven-membered metallacycle **IV**. Driven by aromatization, a facile reductive elimination forms the chiral spirocyclic pyridine matching the obtained absolute configuration of **8m**. Coordination of the next set of substrates regenerates intermediate **I** and completes the catalytic cycle.

## Conclusion

We reported a nickel-catalysed pyridine-forming enantioselective hetero [2+2+2] cycloaddition as a unified approach for constructing chiral carbon-, boron- and germanium-centred spiro-skeletons with excellent yield and good to moderate enantioselectivities. Although some of the obtained enantioselectivities of boron and germanium compounds are not yet ideal, the methodology provides a robust foundation for future advancements in this emerging field. The required remote stereocontrol is achieved by a designed chiral bulky NHC-Ni(0)–styrene complex. An electron-poor aryl group as chiral side arm provides a strong *π*–*π* stacking with the ligand backbone and creates a well-defined C_2_-symmetric chiral pocket that proved to be pivotal for the induced selectivity of the catalyst. The reaction proceeds via a heterocoupling mechanism with the migratory insertion of an enantiotopic alkyne towards the metallaazirene species being the enantiodetermining step. Moreover, the technology is suitable for rapidly assembling highly complex compounds with attractive and exploitable photophysical and chiro-optical properties. The outlined method considerably enlarges the toolbox to assemble chiral functional materials and to construct boron and germanium heteroatom chirality.

## Methods

### General procedure for synthesizing carbon-centred dialkyne starting materials

Under N_2_ atmosphere, a stirred mixture of fluorene substrate (1 equiv.), NaI (1 equiv.) and ^*t*^BuOK (3 equiv.) in THF (0.2 M) was added 1-bromo-2-butyne (3 equiv.). After stirring at 70 °C for 24 h, the reaction was allowed to cool to room temperature and quenched by addition of water. The mixture was extracted with EtOAc (×3). The organic phase was dried with Na_2_SO_4_, solvents were evaporated, and the residue was purified by flash column chromatography on silica gel.

### General procedure for synthesizing boron-centred dialkyne starting materials

#### Step 1

BBr_3_ (3.0 equiv., 1.0 M in CH_2_Cl_2_) was added to a stirred solution of 2-phenyl-pyridine derivatives and ^*i*^Pr_2_NEt (1.2 equiv.) in CH_2_Cl_2_ (1.0 M) at 0 °C. After being stirred at room temperature for 24 h, saturated K_2_CO_3_ aqueous solution was added to the reaction mixture. The organic layer was separated and extracted with CH_2_Cl_2_, washed with water, brine, and dried over anhydrous Na_2_SO_4_. The solvent was removed under vacuum, and the resulting solid was collected by filtration and washed with hexane to give crude dibromo-boron intermediate, which was used directly without further purification.

BCl_3_ (3.0 equiv., 1.0 M in CH_2_Cl_2_) was added dropwise to a solution of p8 or p9 in toluene (0.25 M) over 10 min at 0 °C under N_2_ atmosphere. After being stirred at room temperature for 24 h, saturated Na_2_CO_3_ aqueous solution was added to the reaction mixture. The organic layer was separated and extracted with CH_2_Cl_2_, washed with water, brine and dried over anhydrous Na_2_SO_4_. The solvent was removed under vacuum, and the resulting solid was collected by filtration and washed with hexane to give the crude dichloroboron intermediate, which was used directly without further purification.

#### Step 2

Freshly prepared propargyl magnesium bromide (2.2 equiv., in THF, around 0.5 M; note that the concentration should be titrated) was added to a solution of crude intermediate in THF (0.2 M) dropwise over 10 min at 0 °C under N_2_ atmosphere. After stirring for 30 min, the mixture was warmed to ambient temperature and stirred for 2 h. The reaction mixture was quenched with saturated NH_4_Cl aqueous solution and extracted with CH_2_Cl_2_, the combined organic layer was washed with brine and dried over anhydrous Na_2_SO_4_. The solvent was removed under vacuum and the residue was purified by flash chromatography on silica gel to afford compounds **2a**–**2i**.

### General procedure for synthesizing germanium-centred dialkyne starting materials

Freshly titrated *n*BuLi (2.05 equivalents) was added dropwise to a stirred solution of 2,2’-dibromobiaryl derivatives in dry Et_2_O (0.1 M) at −78 °C. The reaction mixture was stirred for 1 h at −78 °C. The reaction mixture was warmed and then stirred overnight at 0 °C. The solution was added dropwise by cannula to a stirred solution of GeCl_4_ (5.0 equiv.) precooled to −95 °C (liquid N_2_ and hexane) in Et_2_O (0.1 M) over at least 20 min. The reaction mixture was stirred for 2 h at −78 °C (acetone and dry ice), before being allowed to warm slowly to room temperature. The reaction mixture was then stirred overnight. The mixture was then cooled to −78 °C, the volatiles were removed by a Schlenk line and the evaporated solvents and GeCl_4_ were collected in a trap in liquid nitrogen. Et_2_O (0.1 M) was then added to the mixture, and then freshly prepared but-2-yn-1-yl magnesium bromide (4 equiv., concentration was titrated) was added dropwise. The reaction mixture was allowed to slowly warm to room temperature, with overnight stirring. The reaction was then quenched with saturated NH_4_Cl aqueous solution and extracted with CH_2_Cl_2_, the combined organic layer was washed with brine and dried over anhydrous Na_2_SO_4_. The solvent was removed under vacuum and the residue was purified by flash chromatography on silica gel to afford compounds **4a**, **4b**, **5a**, **5b** and **5c**.

### General procedure for nickel-catalysed enantioselective [2+2+2] cycloaddition

In a glovebox, an oven dried screw-capped 2 ml vial was charged with a magnetic stir bar, Ni(0)NHC styrene complex **Ni3** (3 μmol) and cyclohexane (0.5 ml) was then added. Dialkyne (0.1 mmol) and nitrile (0.1 mmol) were then added successively. The vial was sealed with a Teflon-lined screw cap and the reaction mixture was stirred inside the glovebox at room temperature. After 24 h, the vial was shipped outside of the glovebox. The reaction mixture was diluted with dichloromethane and filtered through a plug of silica gel. The crude solution was concentrated in vacuum and subjected to column chromatography to isolate the products. Refer to the Supplementary Information for more detailed experimental procedures.

## Supplementary information

Supplementary Information Supplementary Figs. 1–16, Discussion and Tables 1–14.

Supplementary Data Crystallographic data of **8m**.

Supplementary Data Crystallographic data of **Ni2**.

Supplementary Data Crystallographic data of **Ni3**.

Supplementary Data Crystallographic data of **Ni4**.

Supplementary Data Crystallographic data **8m** refine at space group *P*-1.

## Data Availability

CCDC deposition no. of **Ni2**: 2370294, CCDC deposition number of **Ni3**: 2370293, CCDC deposition no. **8m**: 2370292, CCDC deposition no. **Ni4**: 2370295. All other data is available from the authors on reasonable request.

## References

[1] Walsh, P. J. & Kozlowski, M. C. *Fundamentals of Asymmetric Catalysis* (Univ. Science Books, 2010).

[2] Wagen, C. C., McMinn, S. E., Kwan, E. E. & Jacobsen, E. N. Screening for generality in asymmetric catalysis. *Nature* **610**, 680–686 (2022). 36049504 10.1038/s41586-022-05263-2 PMC9645431

[3] Pop, F., Zigon, N. & Avarvari, N. Main-group-based electro- and photoactive chiral materials. *Chem. Rev.* **119**, 8435–8478 (2019). 30943018 10.1021/acs.chemrev.8b00770

[4] Ding, K., Han, Z. & Wang, Z. Spiro skeletons: a class of privileged structure for chiral ligand design. *Chem. Asian J.* **4**, 32–41 (2009). 18770872 10.1002/asia.200800192

[5] Zheng, Y., Tice, C. M. & Singh, S. B. The use of spirocyclic scaffolds in drug discovery. *Bioorg. Medicinal Chem. Lett.* **24**, 3673–3682 (2014). 10.1016/j.bmcl.2014.06.081 25052427

[6] Saragi, T. P. I., Spehr, T., Siebert, A., Fuhrmann-Lieker, T. & Salbeck, J. Spiro compounds for organic optoelectronics. *Chem. Rev.* **107**, 1011–1065 (2007). 17381160 10.1021/cr0501341

[7] Grell, M. et al. A compact device for the efficient, electrically driven generation of highly circularly polarized light. *Adv. Mater.* **13**, 577–580 (2001).

[8] Yang, S. Y. et al. Circularly polarized thermally activated delayed fluorescence emitters in through-space charge transfer on asymmetric spiro skeletons. *J. Am. Chem. Soc.* **142**, 17756–17765 (2020). 33021373 10.1021/jacs.0c08980

[9] Shynkaruk, O., He, G., McDonald, R., Ferguson, M. J. & Rivard, E. Modular synthesis of spirocyclic germafluorene-germoles: a new family of tunable luminogens. *Chem. Eur. J.* **22**, 248–257 (2016). 26603134 10.1002/chem.201503377

[10] Li, X., Zhang, G. & Song, Q. Recent advances in the construction of tetracoordinate boron compounds. *Chem. Commun.* **59**, 3812–3820 (2023). 10.1039/d2cc06982b 36883254

[11] Otocka, S., Kwiatkowska, M., Madalinska, L. & Kielbasinski, P. Chiral organosulfur ligands/catalysts with a stereogenic sulfur atom: applications in asymmetric synthesis. *Chem. Rev.* **117**, 4147–4181 (2017). 28191933 10.1021/acs.chemrev.6b00517

[12] Harvey, J. S. & Gouverneur, V. Catalytic enantioselective synthesis of P-stereogenic compounds. *Chem. Commun.* **46**, 7477–7485 (2010). 10.1039/c0cc01939a 20835471

[13] Ye, F., Xu, Z. & Xu, L.-W. The discovery of multifunctional chiral P ligands for the catalytic construction of quaternary carbon/silicon and multiple stereogenic centers. *Acc. Chem. Res.* **54**, 452–470 (2021). 33375791 10.1021/acs.accounts.0c00740

[14] Ge, Y., Ke, J. & He, C. Catalytic asymmetric dehydrogenative Si−H/X−H coupling toward Si-stereogenic silanes. *Acc. Chem. Res.* **58**, 375–398 (2025). 39841996 10.1021/acs.accounts.4c00667

[15] Zu, B., Guo, Y. & He, C. Catalytic enantioselective construction of chiroptical boron-stereogenic compounds. *J. Am. Chem. Soc.* **143**, 16302–16310 (2021). 34570969 10.1021/jacs.1c08482

[16] Guo, Y., Zu, B., Chen, C. D. & He, C. Boron-stereogenic compounds: synthetic developments and opportunities. *Chin. J. Chem.* **42**, 2401–2411 (2024).

[17] Ren, L. Q. et al. Modular enantioselective assembly of multi-substituted boron-stereogenic BODIPYs. *Nat. Chem.* **17**, 83–91 (2025). 39304724 10.1038/s41557-024-01649-z

[18] Zhan, B. et al. Catalytic asymmetric C–N cross-coupling towards boron-stereogenic 3-amino-BODIPYs. *Nat. Commun.* **16**, 438 (2025). 39762224 10.1038/s41467-024-55796-5 PMC11704012

[19] Han, A. C., Xiao, L. J. & Zhou, Q. L. Construction of Ge-stereogenic center by desymmetric carbene insertion of dihydrogermanes. *J. Am. Chem. Soc.* **146**, 5643–5649 (2024). 38327018 10.1021/jacs.3c14386

[20] Lin, W. et al. Cu-catalyzed asymmetric hydrogermylation towards C- and Ge-stereogenic germanes. *CCS Chem.* **7**, 1157–1167 (2025).

[21] Shintani, R., Takagi, C., Ito, T., Naito, M. & Nozaki, K. Rhodium-catalyzed asymmetric synthesis of silicon stereogenic dibenzosiloles by enantioselective [2+2+2] cycloaddition. *Angew. Chem., Int. Ed.* **54**, 1616–1620 (2015). 10.1002/anie.201409733 25491349

[22] Li, D., Zhang, H. & Wang, Y. Four-coordinate organoboron compounds for organic light-emitting diodes (OLEDs). *Chem. Soc. Rev.* **42**, 8416–8433 (2013). 23900268 10.1039/c3cs60170f

[23] Rogova, T., Ahrweiler, E., Schoetz, M. D. & Schoenebeck, F. Recent developments with organogermanes: their preparation and application in synthesis and catalysis. *Angew. Chem. Int. Ed.* **63**, No. e202314709 (2023). 10.1002/anie.202314709 37899306

[24] Rios, R. in *Spiro Compounds: Synthesis and Applications* 283–312 (Wiley, 2021).

[25] Xu, P. W. et al. Catalytic enantioselective construction of spiro quaternary carbon stereocenters. *ACS Catal.* **9**, 1820–1882 (2019).

[26] Talele, T. T. Opportunities for tapping into three-dimensional chemical space through a quaternary carbon. *J. Med. Chem.* **63**, 13291–13315 (2020). 32805118 10.1021/acs.jmedchem.0c00829

[27] Lou, Y., Wei, J., Li, M. & Zhu, Y. Distal ionic substrate–catalyst interactions enable long-range stereocontrol: access to remote quaternary stereocenters through a desymmetrizing Suzuki–Miyaura reaction. *J. Am. Chem. Soc.* **144**, 123–129 (2022). 34979078 10.1021/jacs.1c12345 PMC9549467

[28] Hamada, H., Itabashi, Y., Shang, R. & Nakamura, E. Axially chiral spiro-conjugated carbon-bridged *p*-phenylenevinylene congeners: synthetic design and materials properties. *J. Am. Chem. Soc.* **142**, 2059–2067 (2020). 31922417 10.1021/jacs.9b13019

[29] Yoshigoe, Y., Hashizume, K. & Saito, S. Synthesis and stereochemistry of chiral aza-boraspirobifluorenes with tetrahedral boron-stereogenic centers. *Dalton Trans.* **42**, 17035–17039 (2022). 10.1039/d2dt03303h 36305179

[30] Kitschke, P., Rüffer, T., Lang, H., Auer, A. A. & Mehring, M. Chiral spirocyclic germanium thiolates—an evaluation of their suitability for twin polymerization based on a combined experimental and theoretical study. *ChemistrySelect* **1**, 1184–1191 (2016).

[31] Zhang, Y.-F. et al. Dinuclear zinc-catalyzed desymmetric intramolecular aldolization: an enantioselective construction of spiro[cyclohexanone-oxindole] derivatives. *RSC Adv.* **6**, 30683–30689 (2016).

[32] Shan, C. C., Wang, Z.-L. & Xu, Y.-H. Copper-catalyzed desymmetric silylative-cyclization of 1,6-diynes for synthesis of spirocyclic compounds. *Org. Chem. Front.* **11**, 1211–1217 (2024).

[33] Matton, P., Huvelle, S., Haddad, M., Phansavath, P. & Ratovelomanana-Vidal, V. Recent progress in metal-catalyzed [2+2+2] cycloaddition reactions. *Synthesis* **54**, 4–32 (2022).

[34] Roglans, A., Pla-Quintana, A. & Solà, M. Mechanistic studies of transition-metal-catalyzed [2+2+2] cycloaddition reactions. *Chem. Rev.* **121**, 1894–1979 (2021). 32786426 10.1021/acs.chemrev.0c00062

[35] Hapke, M. & Hilt. G. in *Cobalt Catalysis in Organic Synthesis: Methods and Reactions* (eds Gläsel, T. & Hapke M.) 287–335 (Wiley, 2022).

[36] Kwong, H. L. et al. Chiral pyridine-containing ligands in asymmetric catalysis. *Coord. Chem. Rev.* **251**, 2188–2222 (2007).

[37] Costa, R. D. et al. Luminescent ionic transition-metal complexes for light-emitting electrochemical cells. *Angew. Chem. Int. Ed.* **51**, 8178–8211 (2012). 10.1002/anie.201201471 22887710

[38] Link, A. & Sparr, C. Stereoselective arene formation. *Chem. Soc. Rev.* **47**, 3804–3815 (2018). 29565066 10.1039/C7CS00875A

[39] Wang, H. et al. Enantio- and regioselective [2+2+2] cycloaddition of BN-diynes for construction of C–B axial chirality. *Chem* **10**, 317–329 (2024).

[40] Cai, J. et al. Ni-catalyzed enantioselective [2+2+2] cycloaddition of malononitriles with alkynes. *Chem* **7**, 799–811 (2021).

[41] Bhatarah, P., Smith, E. H. & Kensington, S. Nickel(0)-promoted synthesis of tetralin lactones from the monoynes and octa-1,7-diynes terminally substituted with groups co-cyclisation of ester or amide groups. *J. Chem. Soc. Perkin. Trans*. **1**, 2163–2168 (1992).

[42] Zeng, X. P., Cao, Z. Y., Wang, Y. H., Zhou, F. & Zhou, J. Catalytic enantioselective desymmetrization reactions to all-carbon quaternary stereocenters. *Chem. Rev.* **116**, 7330–7396 (2016). 27251100 10.1021/acs.chemrev.6b00094

[43] Diesel, J., Finogenova, A. M. & Cramer, N. Nickel-catalyzed enantioselective pyridone C–H functionalizations enabled by a bulky N-heterocyclic carbene ligand. *J. Am. Chem. Soc.* **140**, 4489–4493 (2018). 29543449 10.1021/jacs.8b01181

[44] Cao, Y. X., Wodrich, M. D. & Cramer, N. Nickel-catalyzed direct stereoselective α-allylation of ketones with non-conjugated dienes. *Nat. Commun.* **14**, 7640 (2023). 37993440 10.1038/s41467-023-43197-z PMC10665391

[45] Shaya, J. et al. Design, photophysical properties, and applications of fluorene-based fluorophores in two-photon fluorescence bioimaging: a review. *J. Photochem. Photobiol. C.* **52**, 100529 (2022).

[46] Cai, Y., Zhang, J. W., Li, F., Liu, J. M. & Shi, S. L. Nickel/N-heterocyclic carbene complex-catalyzed enantioselective redox-neutral coupling of benzyl alcohols and alkynes to allylic alcohols. *ACS Catal.* **9**, 1–6 (2019).

[47] Hancock, S. N., Yuntawattana, N., Valdez, S. M. & Michaudel, Q. Expedient synthesis and ring-opening metathesis polymerization of pyridinonorbornenes. *Polym. Chem.* **13**, 5530–5535 (2022). 37193226 10.1039/d2py00857b PMC10168028

[48] Yang, T., Tang, N., Wan, Q., Yin, S.-F. & Qiu, R. Recent progress on synthesis of *N*,*N*′-chelate organoboron derivatives. *Molecules* **26**, 1401 (2021). 33807680 10.3390/molecules26051401 PMC7961668

[49] Keller, S. et al. Shine bright or live long: substituent effects in [Cu(N^N)(P^P)]^+^-based light-emitting electrochemical cells where N^N is a 6-substituted 2,2′-bipyridine. *J. Mater. Chem. C.* **4**, 3857–3871 (2016).

[50] Brooks, J. et al. Synthesis and characterization of phosphorescent cyclometalated platinum complexes. *Inorg. Chem.* **41**, 3055–3066 (2002). 12054983 10.1021/ic0255508

[51] Stanoppi, M. & Lorbach, A. Boron-based donor-spiro-acceptor compounds exhibiting thermally activated delayed fluorescence (TADF). *Dalton Trans.* **47**, 10394–10398 (2018). 29737352 10.1039/c8dt01255e

[52] Zhang, D., Li, M. & Chen, C. Recent advances in circularly polarized electroluminescence based on organic light-emitting diodes. *Chem. Soc. Rev.* **49**, 1331–1343 (2020). 31999286 10.1039/c9cs00680j

[53] Guo, S. M. et al. A C–H activation-based enantioselective synthesis of lower carbo[*n*]helicenes. *Nat. Chem.* **15**, 872–880 (2023). 37024717 10.1038/s41557-023-01174-5 PMC10239729

[54] Stolley, R. M., Duong, H. A. & Louie, J. Mechanistic evaluation of the Ni(IPr)_2_-catalyzed cycloaddition of alkynes and nitriles to afford pyridines: evidence for the formation of a key η^1^-Ni(IPr)_2_(RCN) intermediate. *Organometallics* **32**, 4952–4960 (2013). 25214702 10.1021/om400666k PMC4159214

[55] Humke, J. N. et al. Nickel binding enables isolation and reactivity of previously inaccessible 7-aza-2,3-indolynes. *Science* **384**, 408–414 (2024). 38662814 10.1126/science.adi1606 PMC12045518

