# Supplementary material for: Accessing carbon, boron and germanium spiro stereocentres in a unified catalytic enantioselective approach
Source: Nat Catal. 2025 Jun 12;8(6):569–78. doi: 10.1038/s41929-025-01352-3 (PMC12198011; doi:10.1038/s41929-025-01352-3)
Supplement: Supplementary file 1 — Supplementary Figs. 1–16, Discussion and Tables 1–14. [file 41929_2025_1352_MOESM1_ESM.pdf]

nature catalysis

Supplementary information https://doi.org/10.1038/s41929-025-01352-3

# Accessing carbon, boron and germanium spiro stereocentres in a unified catalytic enantioselective approach

In the format provided by the authors and unedited

# Table of Contents

**Supplementary methods** ........ 3
**Experimental details and characterization data** ........ 5
Chiral aniline synthesis ........ 5
Chiral carbene precursor synthesis ........ 6
Chiral Ni-NHC styrene catalyst synthesis ........ 15
Supplementary Fig. 1. Unit cell of Ni2 complex. ........ 17
Supplementary Fig. 2. ORTEP diagram of selected Ni2 (thermal ellipsoids are shown at the 50% probability level), for the reason of clarity all the hydrogen atoms are omitted. Measured distance: 3.427 Å and 3.283 Å. ........ 18
Supplementary Fig. 3. ORTEP diagram of selected Ni2 (thermal ellipsoids are shown at the 50% probability level), for the reason of clarity all the hydrogen atoms are omitted. Measured distance: 3.469 Å and 3.389 Å. ........ 19
Supplementary Fig. 4. ORTEP diagram of selected Ni2 (thermal ellipsoids are shown at the 50% probability level), for the reason of clarity all the hydrogen atoms are omitted. Measured distance: 3.469 Å and 3.317 Å. ........ 20
Supplementary Fig. 5. Unit cell of Ni3 complex. ........ 22
Supplementary Fig. 6. ORTEP diagram of Ni3 (thermal ellipsoids are shown at the 50% probability level), for the reason of clarity all the hydrogen atoms are omitted. Measured distance: 3.347 Å and 3.311 Å. ........ 23
Calculation of percent Buried Volume ($\%V_{bur}$). ........ 24
**Optimization tables** ........ 25
Supplementary Table 1. Chiral carbene backbone investigations. ........ 26
Supplementary Table 2. Chiral carbene ligand investigations. ........ 27
Supplementary Table 3. Chiral carbene backbone investigations (with electron deficient chiral side arm). ........ 28
Supplementary Table 4. Solvent investigations. ........ 29
Supplementary Table 5. Control experiments. ........ 30
Supplementary Table 6. Temperature screening. ........ 30
Supplementary Table 7. Concentration screening. ........ 31
Supplementary Table 8. Nickel loading screening. ........ 31
Supplementary Table 9. Solvent investigations. ........ 32
Supplementary Table 10. Catalyst investigations. ........ 32
Supplementary Table 11. Chiral carbene ligand investigations. ........ 33
Supplementary Table 12. Catalyst investigations. ........ 34
Supplementary Table 13. Catalyst investigations. ........ 34
Supplementary Table 14. Solvent investigations. ........ 35
**Starting material synthesis** ........ 36
General procedure A: ........ 40
General overview for aza-borafluorene derivatives synthesis. ........ 53
General overview N, N-chelated boron derivatives synthesis. ........ 53
General procedure B: ........ 62
General procedure C: ........ 72
General overview for germa & silafluorene derivatives synthesis. ........ 82
General procedure D: ........ 87
Scope of enantioselective [2+2+2] cycloaddition ........ 94
General procedure E: ........ 94

Supplementary Fig. 7. ORTEP diagram of 8m (thermal ellipsoids are shown at the 50% probability level), for the reason of clarity all the hydrogen atoms are omitted. ........ 122
Silicon centered chiral spiro-skeleton construction. ........ 177
**Synthetic applications**. ........ 178
Photophysical properties of 11a, 11b, and 11c. ........ 187
Supplementary Fig. 8. Corrected excitation and emission spectra of 11a A). 1μM in DCM at 298 K. B). in solid state. C). Xantphos and 8q in DCM solution (1μM). D). 11a 1μM in DCM at 77K. ........ 187
Supplementary Fig. 9. Corrected excitation and emission spectra of A) 11b 1μM in DCM at 298 K and 77K. B). in solid state C) 8b (1μM in DCM). D). acac ligand in DCM solution (1μM). E). 11b 1μM in DCM at 77 K. ........ 188
Supplementary Table 9. Lifetimes and absolute quantum yield of 11a-c. ........ 189
Supplementary Fig. 10. CD spectra of compound 11a. ........ 190
Supplementary Fig. 11. CD spectra of compound 11b. ........ 191
Supplementary Fig. 12. CD spectra and CPL spectra of compound 11c. ........ 192
Supplementary Fig. 13. Synthesis of Ni4 species. ........ 193
Supplementary Fig. 14. Catalysis reactivity of Ni4. ........ 197
Supplementary Fig. 15. Control experiment 1. ........ 198
Supplementary Fig. 16. Control experiment 2. ........ 199
Crystal Data and Experimental ........ 200
**NMR spectra**. ........ 205
**Supplementary references** ........ 322

**Supplementary methods**

All reactions were carried out under an atmosphere of nitrogen in oven-dried glassware with magnetic stirring, unless otherwise indicated. Toluene, dichloromethane, tetrahydrofuran, acetonitrile and diethyl ether were purified by an Innovative Technology Solvent Delivery System. Chemicals were used as obtained from the suppliers. Flash chromatography was performed with Silicycle silica gel 60 (0.040-0.063 µm grade) or acidic alumina (C. Roth, Aluminium oxide 90 acidic). Analytical thin-layer chromatography was performed with commercial glass plates coated with 0.25 mm silica gel (E. Merck, Kieselgel 60 F254). Compounds were either visualised under UV-light at 254 nm or by dipping the plates in an aqueous potassium permanganate solution followed by heating. Proton nuclear magnetic resonance ($^{1}$H-NMR) data were acquired on a Bruker AV400 (400 MHz) or a Bruker DRX600 (600 MHz). Chemical shifts (δ) are reported in parts per million (ppm) relative to incompletely deuterated $CDCl_3$ (s, 7.26 ppm), $C_6D_6$ (s, 7.16 ppm), $CD_3OD$ (p, 3.31 ppm). Splitting patterns are designated as s, singlet; d, doublet; t, triplet; q, quartet; p, pentet; dd, doublet of doublets; qd, quadruplet of dublets; m, multiplet; br, broad. Proton decoupled Carbon-13 nuclear magnetic resonance ($^{13}$C-NMR) data were acquired on a Bruker AV400 (100 MHz) and a Bruker DRX600 (150 MHz) spectrometer. Chemical shifts are reported in ppm relative to $CDCl_3$ (77.16 ppm), $C_6D_6$ (128.06 ppm), $CD_3OD$ (49.00 ppm). Proton decoupled Fluorine-19 nuclear magnetic resonance ($^{19}$F-NMR) were acquired at 376 MHz on a Bruker AV400 spectrometer. Infrared (IR) data were recorded on an Alpha-P Bruker FT-IR Spectrometer. Absorbance frequencies are reported in reciprocal centimeters ($cm^{-1}$). HRMS measurements were performed by an Agilent LC-MS TOF. High resolution mass is given in m/z. Enantiomeric excesses were measured on an Agilent or Waters HPLC, or on a Thar SFC Investigator system using chiral stationary phase columns. Optical rotations were measured on a Polartronic M polarimeter using a 0.5 cm cell with a Na 589 nm filter. X-ray analysis was performed by Dr. R. Scopelliti and Dr. F. Fadaei Tirani at the EPF Lausanne.

The emission and excitation spectra were recorded on a Horiba-Jobin Yvon Fluorolog FL-3-22 fluorimeter equipped with CW 450W Xenon source for fluorescence mode. Data were collected by using a thermoelectrically cooled R2658P PMT (Hamamatsu, range 220-1010 nm) or a NIR PMT (950-1700 nm, thermoelectrically cooled H10330-75 NIR-PMT; Hamamatsu). The spectra were corrected by the instrumental correction function. Data processing was performed with the program Origin 8®. Decays of the excited state were recorded by using TCSPC Delta time unit, using a Xenon source. The data were analysed by using DAS software, by using a mono, bi- or tri-exponential function, and the best fits were kept. They are the averages of at least three independent measurements.

$1 \times 10^{-5}$ M solutions of the samples in DCM were analysed in a quartz capillary. Deaerated solutions were prepared in glove box. Spectra were recorded at room temperature or at low temperature by using a quartz dewar filled with liquid nitrogen.

Quantum yield measurements were performed using a G8 GMP integrating sphere [$\Phi = (Ec - Ea)/((La - Lc).Fatt(\lambda)$, where Ec is the integrated emission spectrum of the sample, Ea is the integrated "blank" emission spectrum, La is the "blank" absorption, and Lc is the sample absorption at the excitation wavelength). $Fatt(\lambda) = 100/T$ where T is the attenuation factor of

the neutral density filter expressed in percentage. Neutral density filters with an optical density of 1, 2 or 3 were used. Values are the averages of at least three independent measurements.
Optical properties: Optical properties were recorded in chromatographic gradesolvent (toluene, $CH_2Cl_2$, acetonitrile). UV-vis absorption spectra were recorded usingan Agilent® Cary-5000 UV-vis spectrophotometer at room temperature. Electronic S3 circular dichroism (ECD) spectra were recorded on a JASCOJ-815spectropolarimeter at room temperature in a 1 cm-cuvette.
The circularly polarized luminescence (CPL) spectra were recorded with a JASCOCPL-200 spectrometer at room temperature.

## Experimental details and characterization data

Chiral aniline synthesis

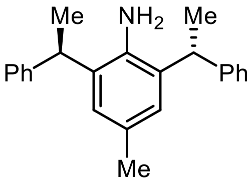


**a1**

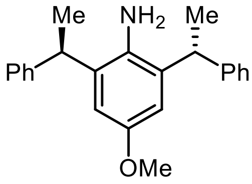


**a2**

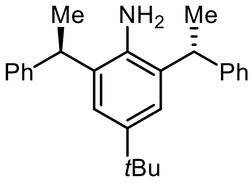


**a3**

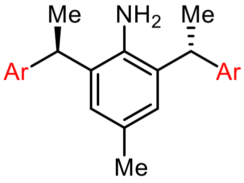


Ar = 3,5 di-Methyl-phenyl

**a4**

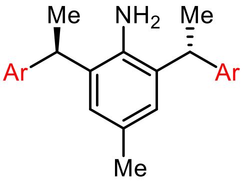


Ar = 3,5 di-F-phenyl

**a5**

Chiral aniline **a1**, **a2** & **a4** was synthesized according to reported literature[1,2].
Chiral aniline **a3** & **a5** was synthesized according to reported literature[3,4].

Chiral carbene precursor synthesis

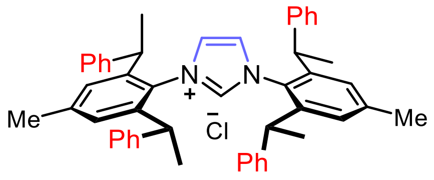

**L1**

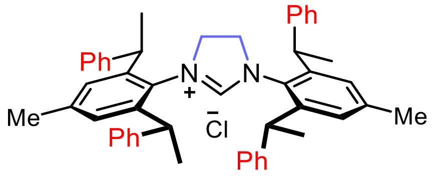

**L2**

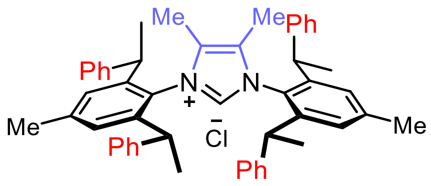

**L3**

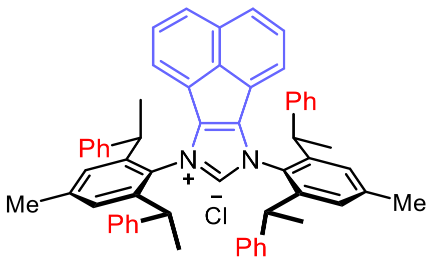

**L4**

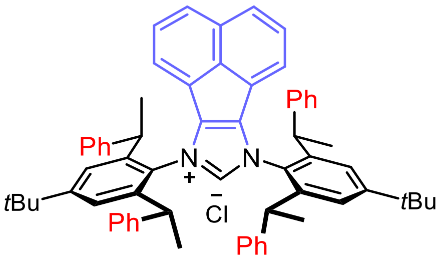

**L5**

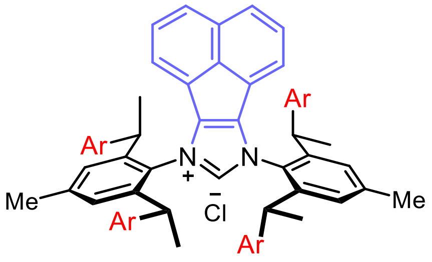

Ar = 3,5 xyl

**L6**

Chiral carbene ligand precursors **L1**, **L2**, **L3**, **L4**, **L5** & **L6** was synthesized according to the literature[1-4].

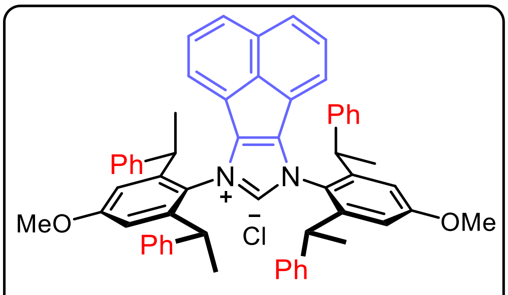


**L7**

**Name**: 7,9-bis(4-methoxy-2,6-bis(1-phenylethyl)phenyl)-7*H*-acenaphtho[1,2-d]imidazol-9-ium chloride (**L7**).

**Synthesis**:

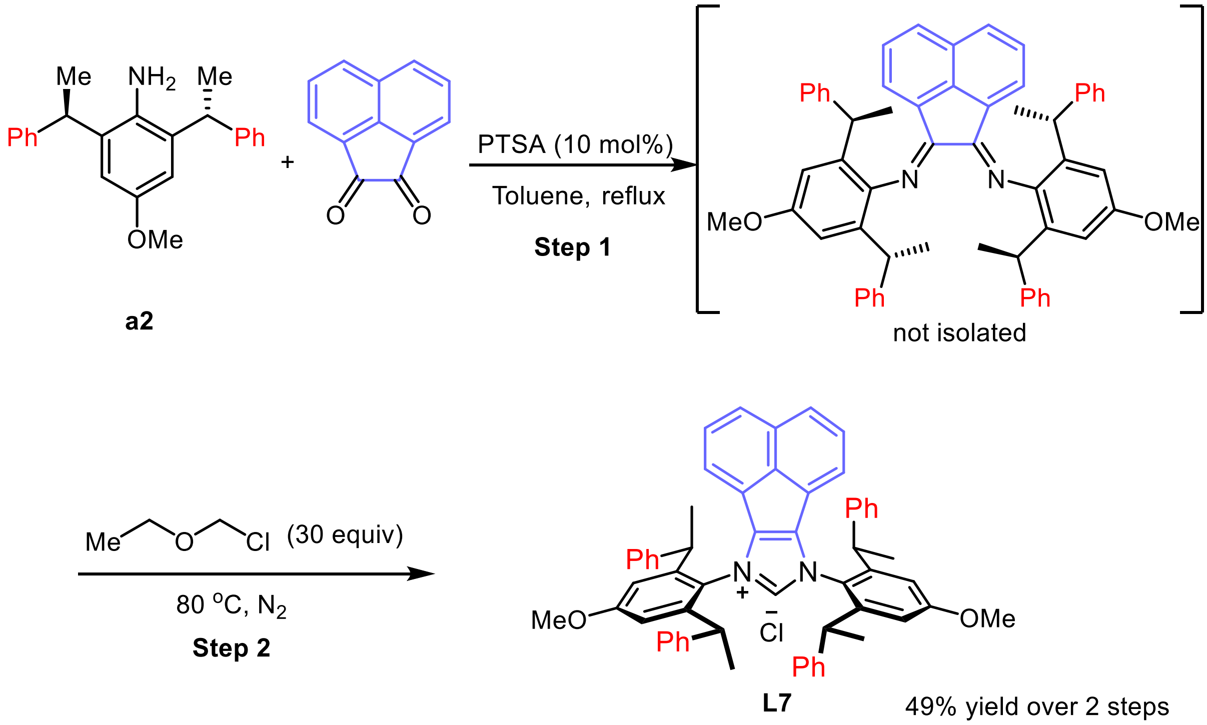


Chiral carbene was synthesized according to a slightly modified literature procedure. (*38*, *39*)

**Step 1**: In a two-necked flask, diketone (284 mg, 1.56 mmol, 1.0 eq) and PTSA (25 mg, 0.156 mmol, 5 mol%) were suspended in toluene (0.1 M) and heated at 80 °C for 5 minutes. Afterward, the chiral aniline (1104 mg, 3.33 mmol, 2.1 eq) was added, and the mixture was refluxed for 36 h. Subsequently, the mixture was concentrated and taken up in ethyl acetate (EA). The EA layer was separated and washed with brine, dried over $Na_2SO_4$, filtered, and evaporated to provide the crude bisimine as a brown solid. The crude product was directly submitted to the next step without any purification.

**Step 2**: In a flame-dried Schlenk tube, an excess of (chloromethoxy)ethane (2 mL, 30 equiv), neutralized over $K_2CO_3$, was added to the crude bisketimine, and the mixture was heated to 80 °C under $N_2$ and stirred overnight (10 hours). The crude reaction mixture was concentrated, dissolved in the minimum amount of DCM, and added dropwise to a pentane/diethyl ether mixture (4:1) to yield the desired acenaphthoquinone imidazolium salt *via* filtration with 650 mg, 0.758 mmol in 49% yield as a brown powder.

$^{1}$**H NMR** (400 MHz, $CD_2Cl_2$) δ 11.28 (s, 1H), 7.85 (d, *J* = 8.2 Hz, 2H), 7.47 – 7.29 (m, 10H), 7.21 (t, *J* = 6.7 Hz, 2H), 7.10 (d, *J* = 2.7 Hz, 2H), 6.86 – 6.76 (m, 4H), 6.71 – 6.52 (m, 10H), 4.06 (q, *J* = 7.0 Hz, 2H), 3.87 (s, 6H), 3.77 – 3.69 (m, 2H), 1.55 (d, *J* = 7.0 Hz, 6H), 1.41 (d, *J* = 6.8 Hz, 6H).

$^{13}$**C NMR** (101 MHz, $CD_2Cl_2$) δ 162.3, 145.6, 144.6, 144.4, 143.2, 138.3, 130.4, 130.14, 129.5, 129.5, 128.4, 128.0, 127.8, 127.3, 127.2, 126.5, 123.9, 123.6, 122.7, 113.2, 112.8, 56.0, 41.0, 39.2, 22.9, 21.4.

**HRMS** (ESI/QTOF) m/z: $[M]^+$ Calcd for $C_{59}H_{53}N_2O_2^+$ 821.4102; Found 821.4111.

$[\boldsymbol{\alpha}]_D^{20}$**:** 130.4 (c = 0.18, $CHCl_3$).

**IR (ATR)**: $\tilde{\nu}$ ($cm^{-1}$) = 2968, 2935, 2893, 2877, 2841, 2823, 2800, 2736, 2360, 2343, 2324, 1666, 1599, 1538, 1517, 1494, 1464, 1446, 1378, 1328, 1261, 1218, 1155, 1139, 1091, 1026, 950, 920, 874, 821, 795, 753, 700, 669, 658, 640, 621, 607, 587, 571, 562, 542, 531, 503, 484, 473, 460, 452, 441, 416, 406.

**Melting point**: decompose (200 °C) before reaching melting point.

**$R_f$**: -

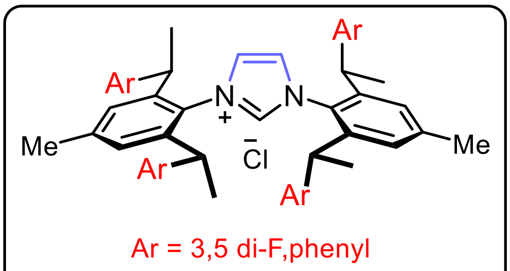


**L8**

**Name**: 1,3-bis(2,6-bis(3,5-difluorophenyl)ethyl)-4-methylphenyl)-1*H*-imidazol-3-ium chloride (**L8**).

**Synthesis**:

Chiral carbene was synthesized according to a slightly modified literature procedure[1,2].

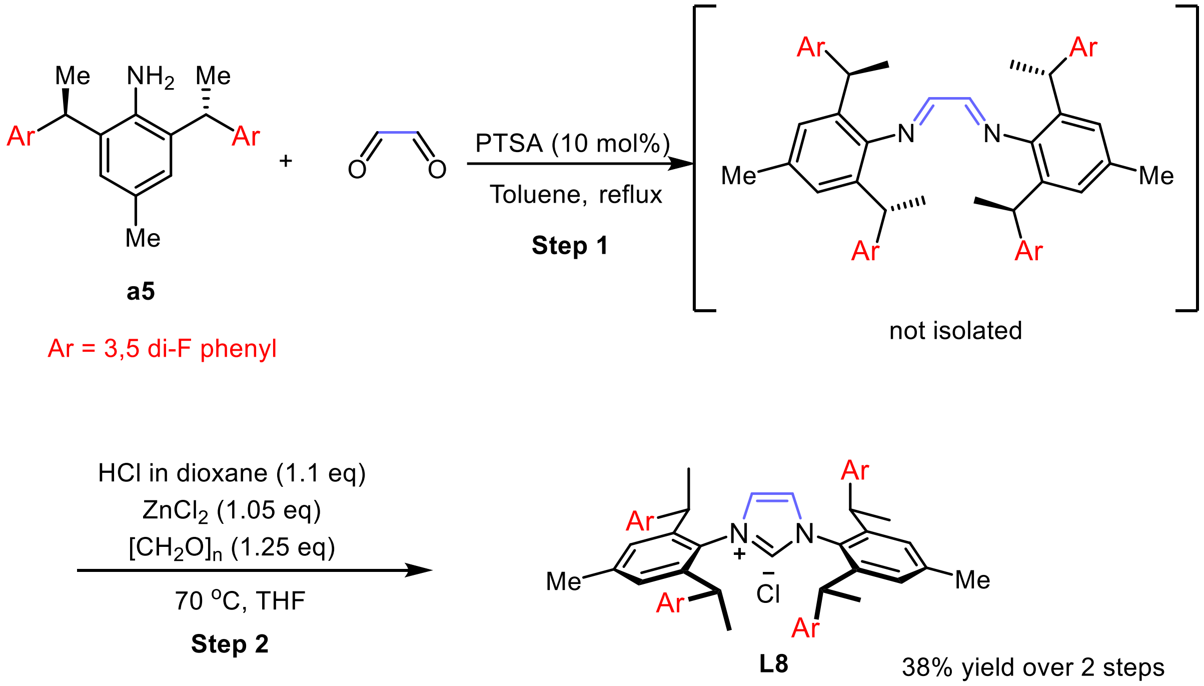


**Step 1**: Chiral aniline **a5** (1000 mg, 2.61 mmol, 1 eq) was suspended in toluene (20 mL) and a 40% solution of glyoxal (0.5 eq) in water, and PTSA (20 mg, 5 mol%) were added. The mixture was reflux under $N_2$ for 24 hours. The crude mixture was concentrated and subsequently recrystallized from MeOH to obtain the desired crude bisimine as brown solid, the crude product was directly submitted to the next step without any purification.

**Step 2**: In a 100 mL two-necked, round bottom flask above synthesized crude bisimine (1 eq) was dissolved in THF (0.25 M) and the solution was heated to 70°C. To this solution $ZnCl_2$ (188 mg, 1.37 mmol, 1.05 eq), a 4 M solution of HCl in 1,4- dioxane (0.41 mL, 1.25 eq) and paraformaldehyde (57.42 mg, 1.44 mmol, 1.1 eq) were added successively and rapidly. The mixture was stirred at 70 °C overnight. The mixture was concentrated and the resulting residue was dissolved in DCM and washed with 20 mL 2M HCl (x2) and 20 mLbrine of pH=8 (x1). The organic solution was dried over $Na_2SO_4$ and concentrated. The crude reaction mixture was concentrated, dissolved in the minimum amount of DCM, and added dropwise to a pentane/diethyl ether mixture (4:1) to yield the desired imidazolium salt *via* filtration with 439 mg, 0.50 mmol 38% yield as a pale-yellow powder.

$^{1}$**H NMR** (400 MHz, $CD_2Cl_2$) δ 12.92 (s, 1H), 7.20 – 7.08 (m, 6H), 6.95 (d, *J* = 1.5 Hz, 2H), 6.88 (d, *J* = 2.1 Hz, 2H), 6.64 (tt, *J* = 8.9, 2.3 Hz, 4H), 6.44 – 6.36 (m, 4H), 3.84 (q, *J* = 7.1 Hz, 2H), 3.71 (q, *J* = 6.9 Hz, 2H), 2.38 (s, 6H), 1.56 (d, *J* = 7.1 Hz, 6H), 1.43 (d, *J* = 7.1 Hz, 6H).

$^{13}$**C NMR** (101 MHz, $CD_2Cl_2$) δ 165.2 – 164.4 (m), 162.3 (t, *J* = 12.4 Hz), 149.6 (t, *J* = 8.4 Hz), 147.4 (t, *J* = 8.7 Hz), 143.4 – 142.9 (m), 140.7, 129.4, 129.1, 128.4, 125.2, 112.1 – 111.4 (m), 111.0 – 110.2 (m), 102.6 (td, *J* = 25.4, 10.5 Hz), 40.3, 38.9, 22.4, 21.9, 21.9.

$^{19}$**F NMR** (376 MHz, $CD_2Cl_2$) δ -109.3, -109.6.

**HRMS** (ESI/QTOF) m/z: $[M]^+$ Calcd for $C_{49}H_{41}F_8N_2^+$ 809.3137; Found 809.3160.

$[\alpha]_D^{20}$**:** 212.6 (c = 1.06, $CHCl_3$).

**IR (ATR)**: $\tilde{\nu}$ ($cm^{-1}$) = 2974, 2922, 2878, 2767, 1622, 1595, 1529, 1460, 1443, 1381, 1317, 1261, 1219, 1157, 1116, 1072, 1023, 980, 940, 856, 804, 752, 719, 694, 684, 662, 551, 511.

**Melting point**: decompose (200 °C) before reaching melting point.

$R_f$: -

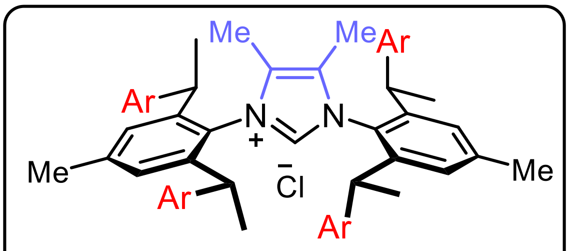


Ar = 3,5 di-F,phenyl

**L9**

**Name**: 1,3-bis(2,6-bis(3,5-difluorophenyl)ethyl)-4-methylphenyl)-4,5-dimethyl-1H-imidazol-3-ium chloride (**L9**).

**Synthesis**:

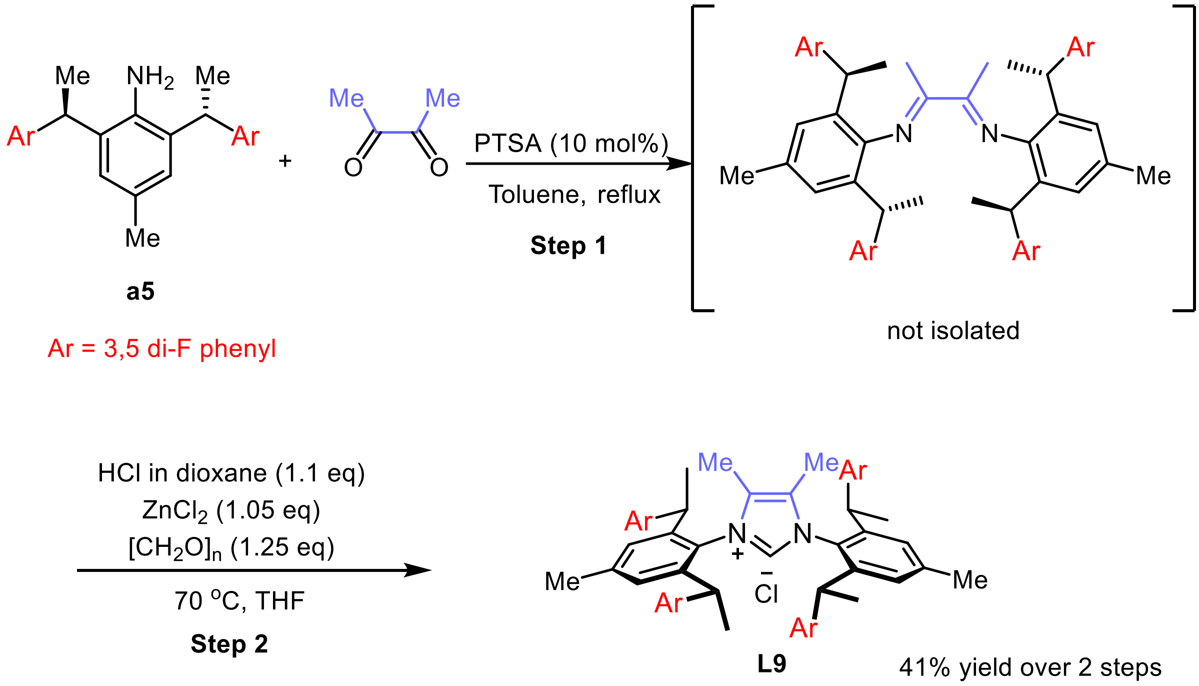


**Step 1**: Chiral aniline **a5** (1000 mg, 2.61 mmol, 1 eq) was suspended in toluene (20 mL) and diacetyl (112 mg, 1.30 mol, 0.5 eq) and PTSA (20 mg, 0.13 mmol, 5 mol%) were added. The mixture was reflux under $N_2$ for 24 hours. The crude mixture was concentrated and subsequently recrystallized from MeOH to obtain the desired crude bisimine as brown solid, the crude product was directly submitted to the next step without any purification.

**Step 2**: In a 100 mL two-necked, round bottom flask above synthesized crude bisimine (1.0 eq) was dissolved in THF (20 mL) and the solution was heated to 70°C. To this solution $ZnCl_2$ (187.5 mg, 1.37 mmol, 1.05 eq), a 4 M solution of HCl in 1,4-dioxane (0.41 mL, 1.25 eq) and paraformaldehyde (57 mg, 1.44 mmol, 1.1 eq) were added successively and rapidly. The mixture was stirred at 70 °C overnight. The mixture was concentrated and the resulting residue was dissolved in DCM and washed with 2M HCl (x2) and brine of pH=8 (x1). The organic solution was dried over $Na_2SO_4$ and concentrated. The crude reaction mixture was concentrated, dissolved in the minimum amount of DCM, and added dropwise to a pentane/diethyl ether mixture (4:1) to yield the desired imidazolium salt *via* filtration with 467 mg, 0.54 mmol, 41% yield as a grey powder.

$^{1}$**H NMR** (400 MHz, $CD_2Cl_2$) δ 12.46 (s, 1H), 7.26 (s, 2H), 7.07 (d, *J* = 6.4 Hz, 4H), 7.01 (s, 2H), 6.77 – 6.61 (m, 6H), 6.44 – 6.34 (m, 4H), 3.70 (q, *J* = 7.1 Hz, 2H), 3.55 (q, *J* = 7.1 Hz, 2H), 2.42 (s, 6H), 1.64 (d, *J* = 7.1 Hz, 6H), 1.58 (s, 6H), 1.37 (d, *J* = 7.0 Hz, 6H).

$^{13}$**C NMR** (101 MHz, $CD_2Cl_2$) δ 165.4 – 164.2 (m), 163.2 – 161.2 (m), 148.8 (t, *J* = 8.1 Hz), 147.4 (t, *J* = 8.7 Hz), 143.5, 142.8, 141.4, 140.7, 129.5, 129.1, 128.7, 127.6, 112.0 – 111.3 (m), 111.2 – 110.3 (m), 102.7 (q, *J* = 25.1 Hz), 40.6, 38.4, 22.7, 21.9, 21.2, 8.8.

$^{19}$**F NMR** (376 MHz, $CD_2Cl_2$) δ -109.1, -109.6.

**HRMS** (ESI/QTOF) m/z: $[M]^+$ Calcd for $C_{51}H_{45}F_8N_2^+$ 837.3450; Found 837.3465.

$[\alpha]_D^{20}$: -148.4 (c = 0.34, $CHCl_3$).

**IR (ATR)**: $\tilde{\nu}$ ($cm^{-1}$) = 2973, 2927, 1622, 1596, 1527, 1460, 1381, 1317, 1261, 1227, 1117, 1021, 981, 940, 856, 803, 751, 694, 660, 604, 550, 511, 491, 472, 449, 422, 404.

**Melting point**: decompose (200 °C) before reaching melting point.

$\mathbf{R_f}$: -

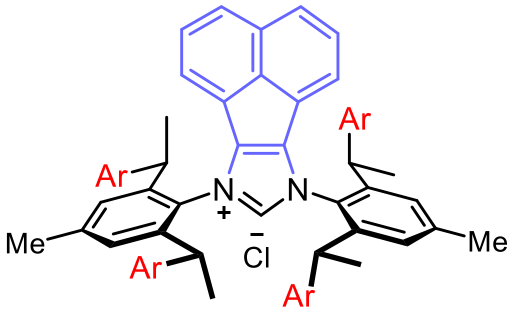


Ar = 3,5 di-F,phenyl

**L10**

**Name**: 7,9-bis(2,6-bis(3,5-difluorophenyl)ethyl)-4-methylphenyl)-7H-acenaphtho[1,2-d]imidazol-9-ium chloride (**L10**).

**Synthesis**:

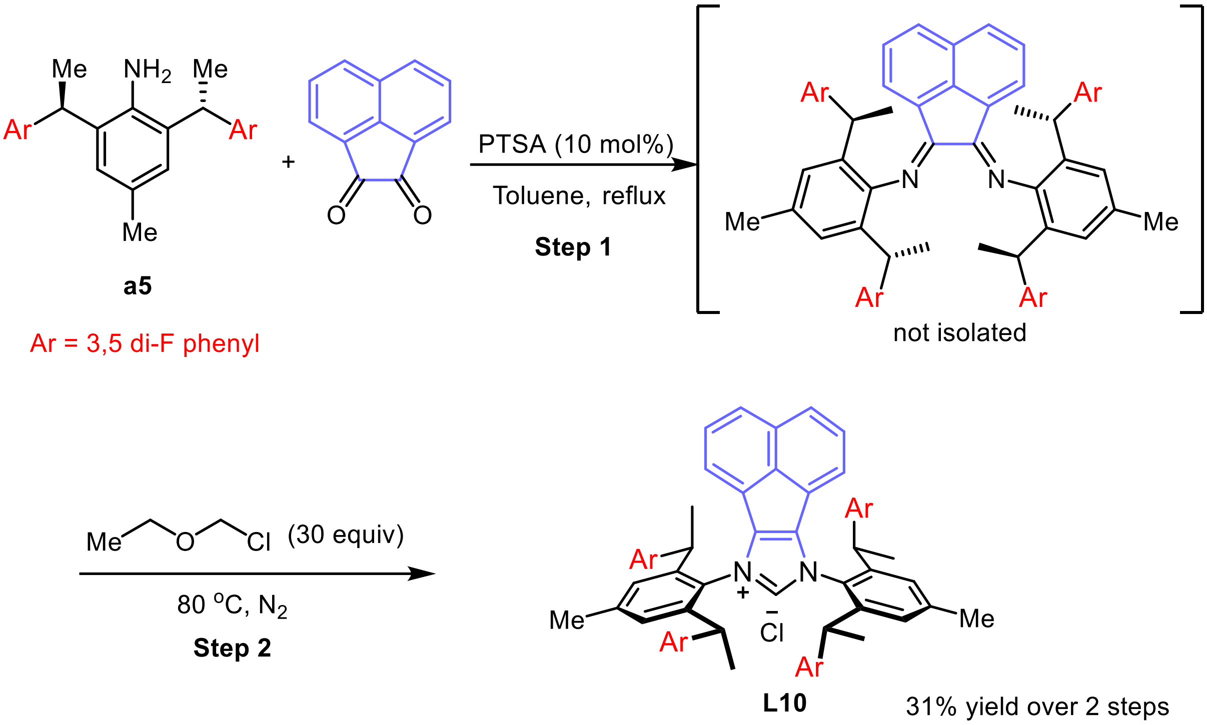


Chiral carbene was synthesized according to a slightly modified literature procedure. (*32*, *33*)
**Step 1**: In a two-necked flask, diketone (390 mg, 2.14 mmol, 1.0 eq) and PTSA (36 mg, 0.21 mmol, 10 mol%) were suspended in toluene (30 mL) and heated at 80 °C for 5 minutes. Afterward, the chiral aniline (1700 mg, 4.38 mmol, 2.05 eq) was added, and the mixture was refluxed for 36 h. Subsequently, the mixture was concentrated and taken up in ethyl acetate (EA). The EA layer was separated and washed with brine, dried over $Na_2SO_4$, filtered, and evaporated to provide the crude bisimine as a brown solid. The crude product was directly submitted to the next step without any purification.
**Step 2**: In a flame-dried Schlenk tube, an excess of (chloromethoxy)ethane (3 mL, 30 eq), neutralized over $K_2CO_3$, was added to the crude bisketimine (1 eq), and the mixture was heated to 80 °C under $N_2$ and stirred overnight. The crude reaction mixture was concentrated, dissolved in the minimum amount of DCM, and added dropwise to a pentane/diethyl ether

mixture (4:1) to yield the desired acenaphthoquinone imidazolium salt *via* filtration with 650 mg, 0.67 mmol, 31% yield as a pink solid.

**$^{1}$H NMR** (400 MHz, $CD_2Cl_2$) δ 12.65 (s, 1H), 7.91 (d, *J* = 8.4 Hz, 2H), 7.46 – 7.37 (m, 4H), 7.18 (d, *J* = 6.3 Hz, 4H), 7.04 (d, *J* = 1.8 Hz, 2H), 6.87 (d, *J* = 7.0 Hz, 2H), 6.67 – 6.61 (m, 2H), 6.20 (d, *J* = 6.1 Hz, 4H), 5.95 (tt, *J* = 9.0, 2.5 Hz, 2H), 4.13 (q, *J* = 7.1 Hz, 2H), 3.94 (q, *J* = 6.8 Hz, 2H), 2.51 (s, 6H), 1.52 (d, *J* = 7.1 Hz, 6H), 1.46 (d, *J* = 7.0 Hz, 6H).

**$^{13}$C NMR** (101 MHz, $CD_2Cl_2$) δ 164.9 (d, *J* = 13.0 Hz), 164.3 (d, *J* = 13.0 Hz), 162.4 (d, *J* = 13.0 Hz), 161.8 (d, *J* = 13.0 Hz), 148.7 (t, *J* = 8.4 Hz), 147.4 (t, *J* = 8.7 Hz), 144.1, 143.6, 143.2, 141.3, 138.2, 130.7, 130.2, 129.7, 129.3, 128.7, 128.4, 127.8, 123.7, 122.4, 111.9, 111.7, 110.8 – 110.2 (m), 102.7 (t, *J* = 25.4 Hz), 101.7 (t, *J* = 25.4 Hz), 41.2, 39.0, 22.6, 22.1, 21.5.

**$^{19}$F NMR** (376 MHz, $CD_2Cl_2$) δ -109.6, -110.3.

**HRMS** (ESI/QTOF) m/z: $[M]^+$ Calcd for $C_{59}H_{45}F_8N_2^+$ 933.3450; Found 933.3484.
**$[\alpha]_D^{20}$:** 105.8 (c = 0.46, $CHCl_3$).

**IR (ATR)**: $\tilde{\nu}$ ($cm^{-1}$) = 2971, 2917, 1621, 1595, 1513, 1437, 1316, 1261, 1217, 1117, 1022, 980, 939, 853, 821, 801, 748, 696, 683, 658, 604, 557, 510.

**Melting point**: decompose before reaching melting point.

**$R_f$**: -

Chiral Ni-NHC styrene catalyst synthesis

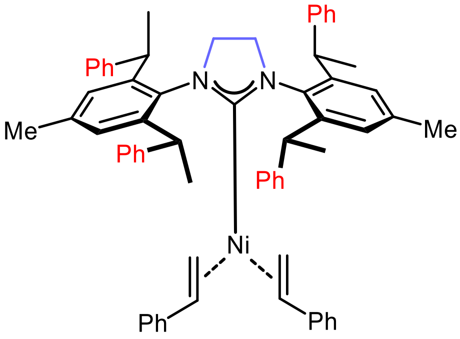


**Ni1**

Chiral Ni(0) complex **Ni1** was synthesized according to reported literature[5].

**$^1$H NMR** (400 MHz, $C_6D_6$) δ: 7.33 – 7.17 (m, 6H), 7.12 – 6.83 (m, 24H), 6.64 (s, 4H), 4.93 (s, 4H), 3.50 (s, 2H), 3.35 (t, J = 10.7 Hz, 2H), 2.91 (d, J = 12.1 Hz, 2H), 2.75 (s, 2H), 2.56 (d, J = 8.4 Hz, 2H), 2.10 (s, 6H), 1.69 (d, J = 6.3 Hz, 6H), 1.48 (d, J = 6.2 Hz, 6H).

And crystal structure of CCDC 1843290 **Ni1**[5] was used to generate steric map and crystal figures.

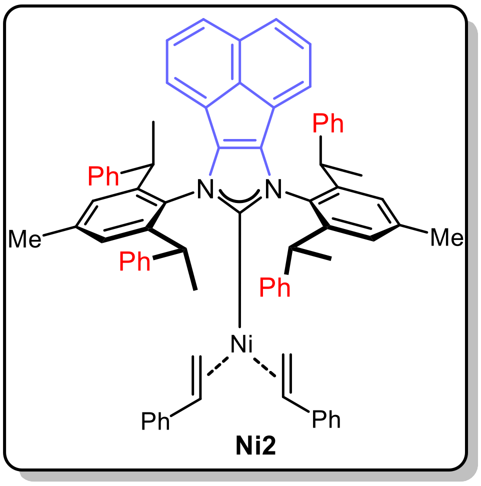

**Name**: **Ni2**

**Synthesis**:

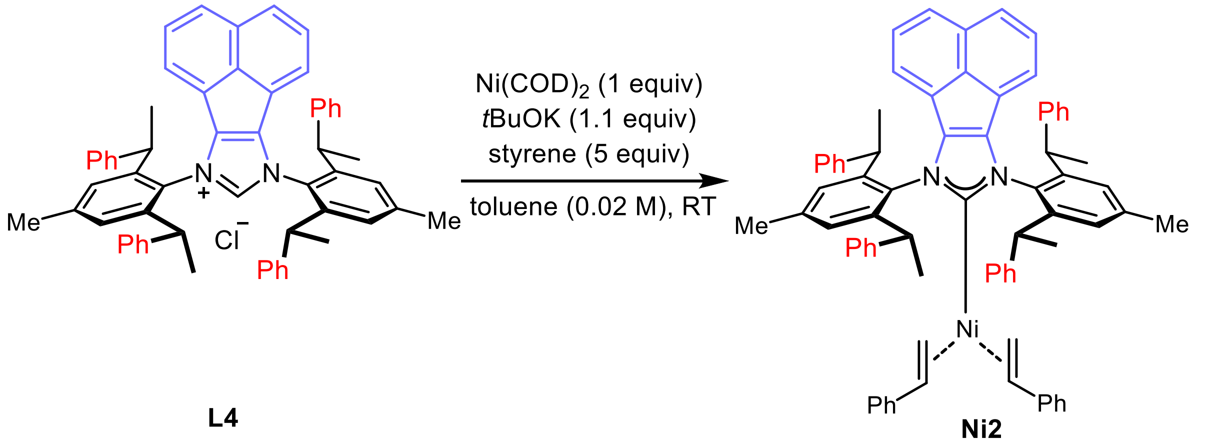


In a nitrogen-filled glove box, bis(1,5-cyclooctadiene) nickel (16.7 mg, 0.0605 mmol, 1.0 eq.), *t*BuOK (7.5 mg, 0.067 mmol, 1.1 eq.), **L4** (50 mg, 0.0605 mmol, 1.0 eq.) and anhydrous toluene (1 mL) were added to a 10 mL microwave vial equipped with a magnetic stirring bar. The reaction mixture was allowed to stir at room temperature for 1 h, followed by adding styrene (31.5 mg, 0.31 mmol, 5.0 eq.). The sealed reaction vial was stirred for 12 h. The resulting mixture was filtered *via* vacuum oven dried celite and concentrated in vacuum. The resulting solid was washed with hexane (1 mL) three times and dried in vacuo to provide **Ni2** as a reddish brown solid in 58.0 mg, 0.0549 mmol, 91% yield. The crystal was obtained by slow diffusion of *n*-hexane to a saturated solution of **Ni2** in $C_6H_6$.

**$^1$H NMR** (400 MHz, $C_6D_6$) δ 7.51 (s, 2H), 7.24 (d, *J* = 8.1 Hz, 2H), 7.12 – 6.95 (m, 21H), 6.86 (s, 3H), 6.75 (d, *J* = 7.5 Hz, 3H), 6.57 (d, *J* = 8.0 Hz, 2H), 6.33 (d, *J* = 7.5 Hz, 6H), 5.95 (d, *J* = 7.0 Hz, 2H), 5.25 (q, *J* = 6.8, 6.4 Hz, 2H), 4.46 (d, *J* = 7.5 Hz, 2H), 3.68 – 3.49 (m, 2H), 3.18 (d, *J* = 13.0 Hz, 2H), 2.93 (d, *J* = 8.9 Hz, 2H), 2.19 (s, 6H), 1.70 (d, *J* = 7.0 Hz, 6H), 1.51 (d, *J* = 7.0 Hz, 6H).

**$^{13}$C NMR** (101 MHz, $C_6D_6$) δ 211.9, 147.5, 146.8, 145.2, 143.9, 143.0, 141.5, 139.8, 135.9, 129.2, 128.8, 127.6, 127.2, 126.7, 126.6, 126.0, 125.6, 124.7, 123.7, 120.4, 120.2, 113.7, 73.7, 51.0, 42.6, 39.0, 28.4, 25.4, 21.9, 21.7.

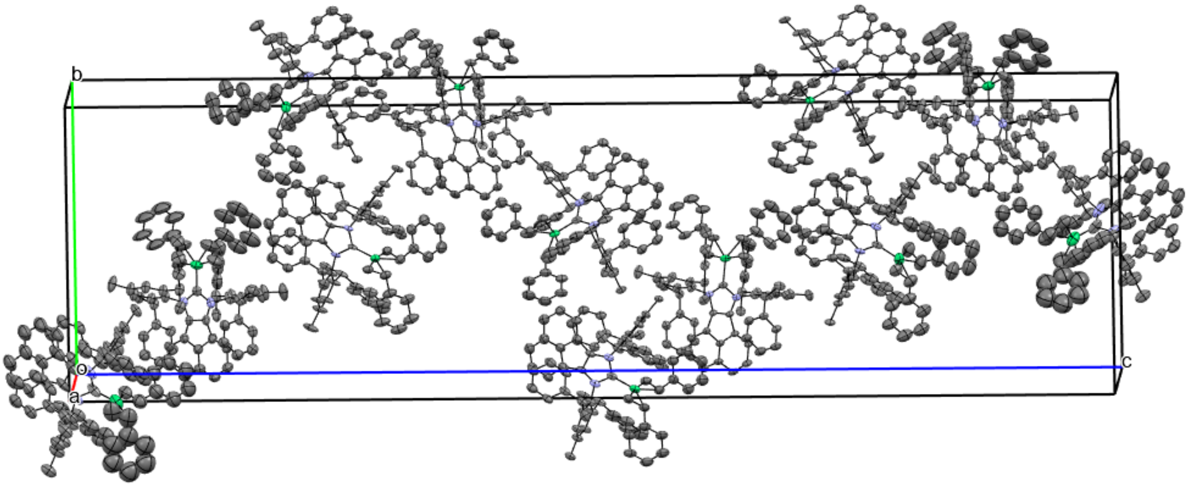


**Supplementary Fig. 1.** Unit cell of **Ni2** complex.

In order to visualize the π-π stacking of the chiral sidearm and acenaphthylene, the distance between the centroid of chiral side arms and plane of acenaphthylene are presented. In the unit cell of **Ni2** complex, we observed that the measured distance differs between each molecule (**Fig. S2** to **Fig. S4**). This indicate that a weak π-π stacking occurs between phenyl group and acenaphthylene backbone, that result the existence of nonnegligible flexibility of the chiral sidearm.

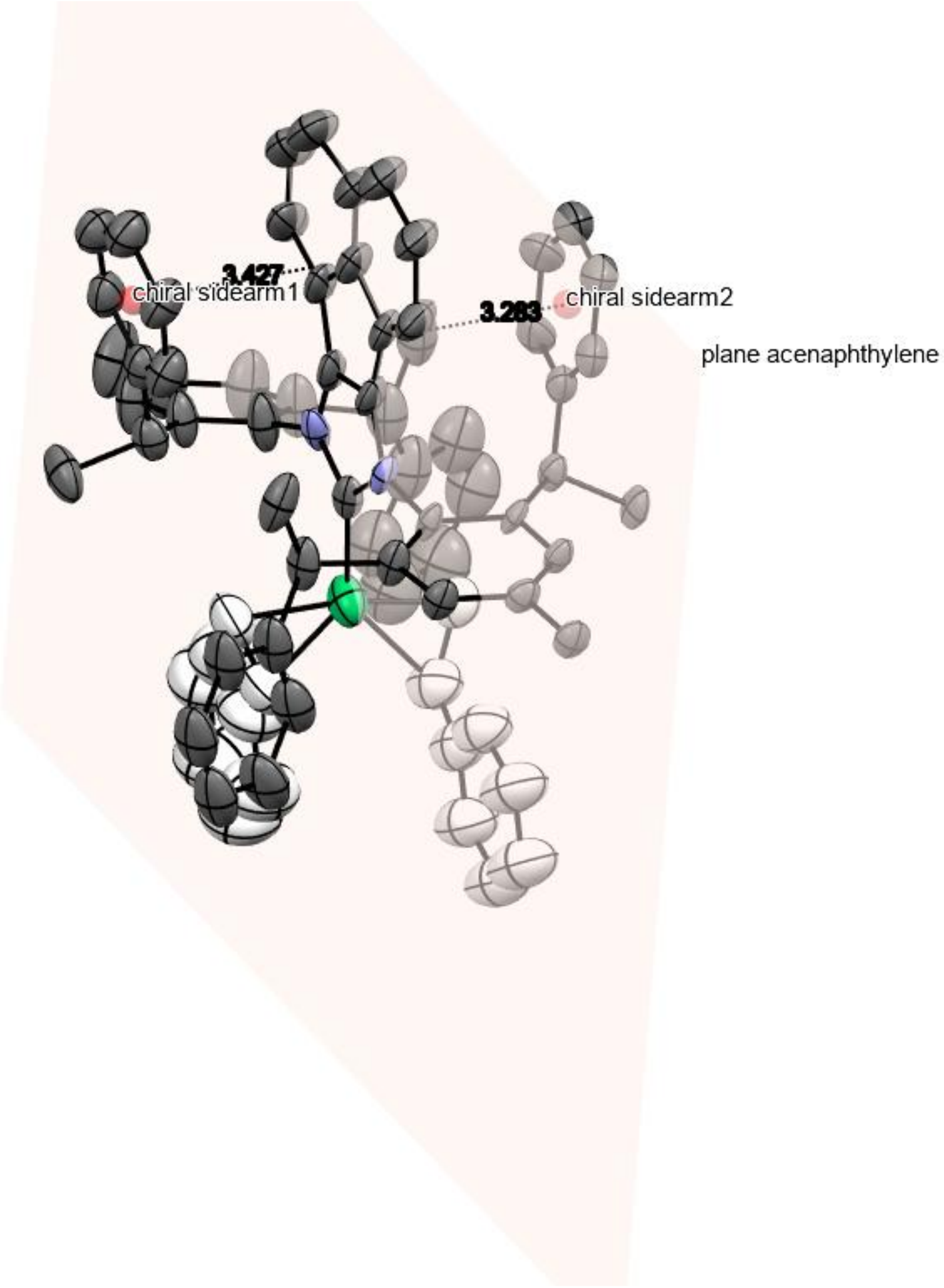


**Supplementary Fig. 2.** ORTEP diagram of selected **Ni2** (thermal ellipsoids are shown at the 50% probability level), for the reason of clarity all the hydrogen atoms are omitted. Measured distance: 3.427 Å and 3.283 Å.

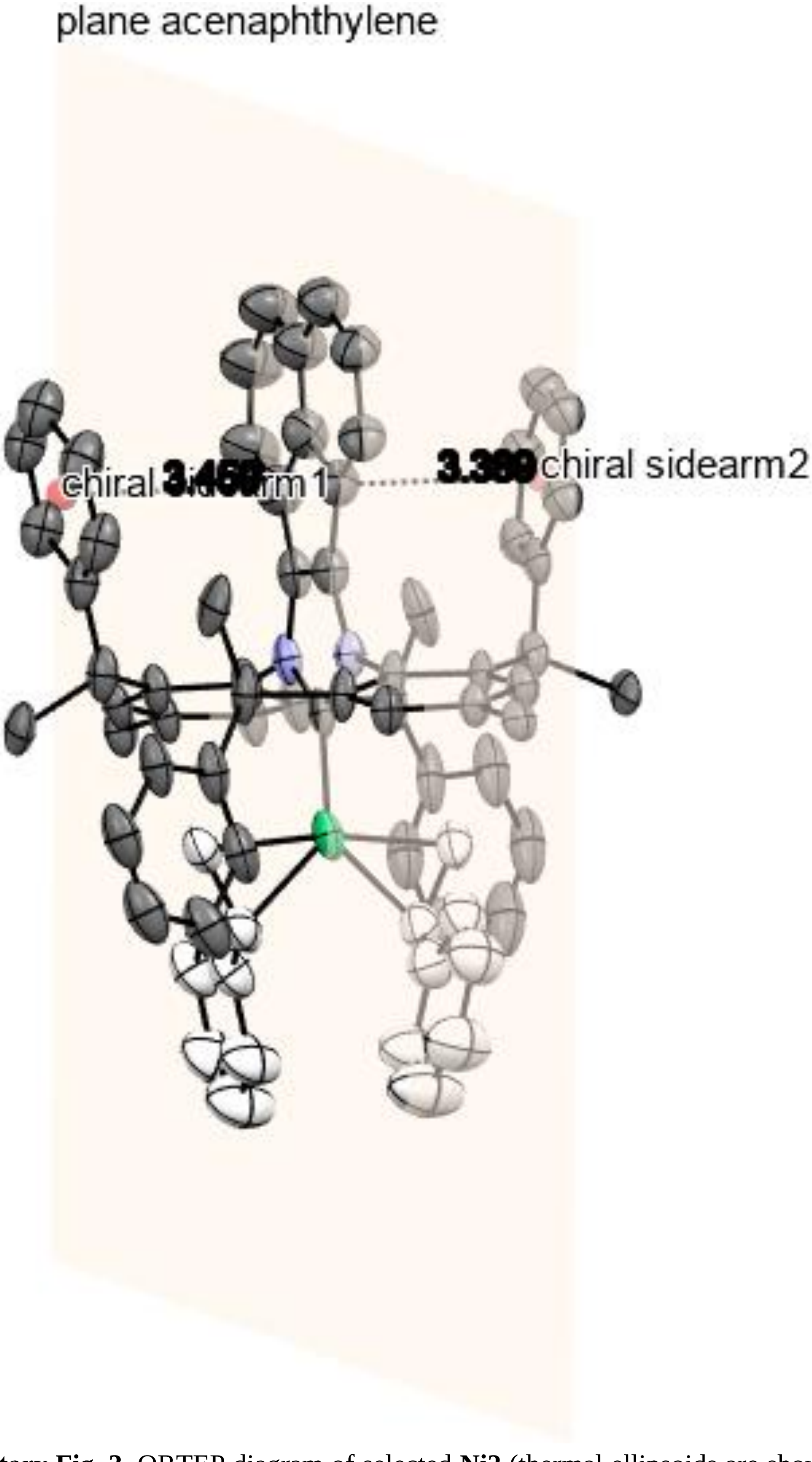


**Supplementary Fig. 3.** ORTEP diagram of selected **Ni2** (thermal ellipsoids are shown at the 50% probability level), for the reason of clarity all the hydrogen atoms are omitted. Measured distance: 3.469 Å and 3.389 Å.

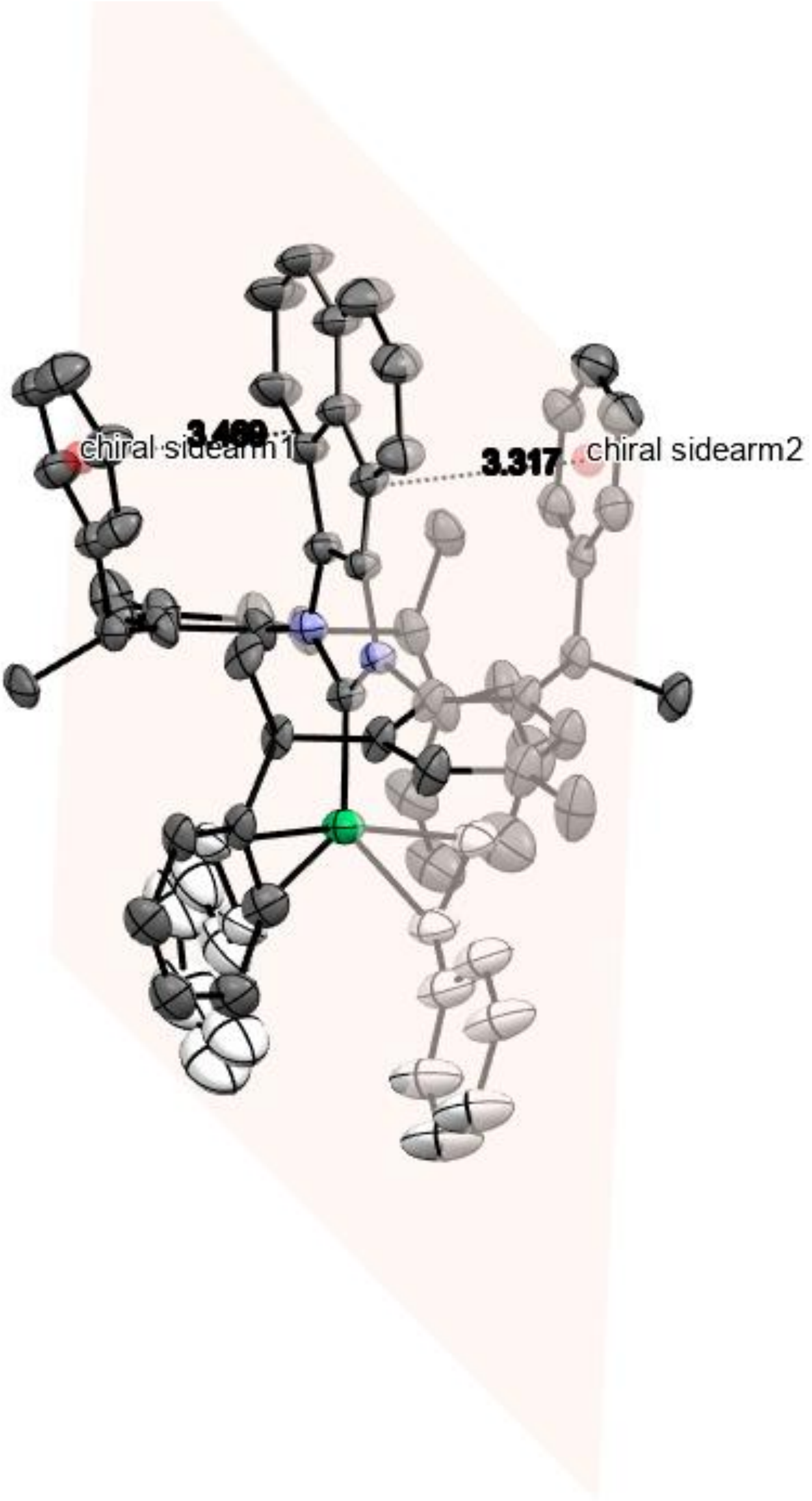


**Supplementary Fig. 4.** ORTEP diagram of selected **Ni2** (thermal ellipsoids are shown at the 50% probability level), for the reason of clarity all the hydrogen atoms are omitted. Measured distance: 3.469 Å and 3.317 Å.

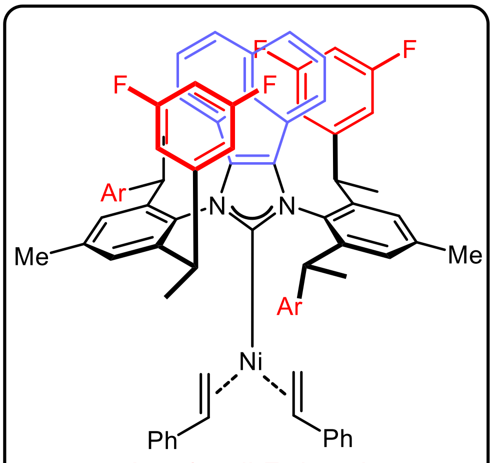


Ar = 3,5 di-F,phenyl
**Ni3**

**Name**: **Ni3**
**Synthesis**:

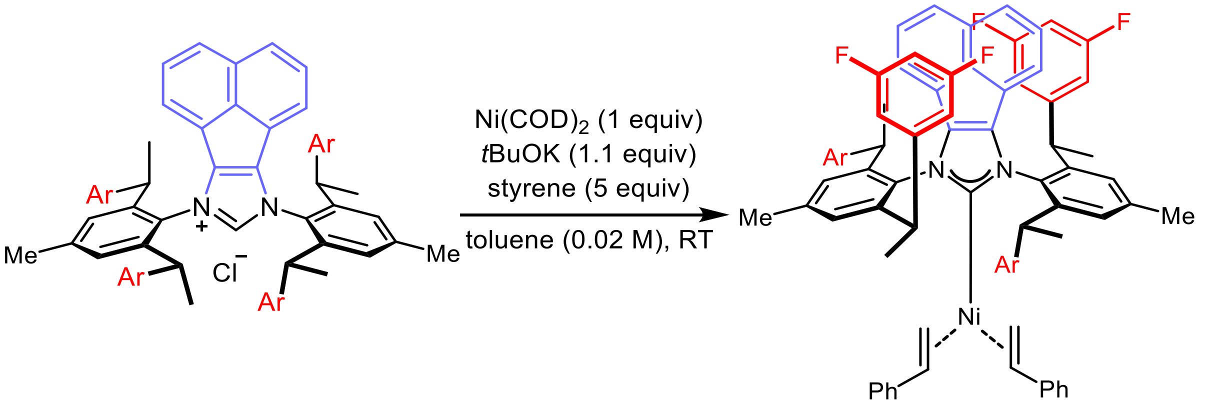


Ar = 3,5 di-F,phenyl
**L10**

Ar = 3,5 di-F,phenyl
**Ni3**

In a nitrogen-filled glove box, bis(1,5-cyclooctadiene) nickel (42.6 mg, 0.156 mmol, 1.0 eq.), *t*BuOK (1.1 eq.), **L10** (150 mg, 0.155 mmol, 1.0 eq.) and anhydrous toluene (3 mL) were added to a 10 mL microwave vial equipped with a magnetic stirring bar. The reaction mixture was allowed to stir at room temperature for 1 h, followed by adding styrene (80.6 mg, 0.774 mmol, 5.0 eq). The sealed reaction vial was stirred for 12 h. The resulting mixture was filtered *via* vacuum oven dried celite and concentrated in vacuum. The resulting solid was washed with hexane (1 mL) three times and dried in vacuo to provide **Ni3** as a reddish brown solid in 148 mg, 0.123 mmol, 80% yield. The crystal was obtained by slow diffusion of *n*-hexane to a saturated solution of **Ni3** in $C_6H_6$.

**$^1$H NMR** (400 MHz, $C_6D_6$) δ 7.31 (s, 2H), 7.15 – 6.95 (m, 9H), 6.87 (s, 2H), 6.79 – 6.70 (m, 2H), 6.66 – 6.53 (m, 7H), 6.40 – 6.21 (m, 6H), 6.09 (d, *J* = 7.0 Hz, 2H), 5.75 – 5.63 (m, 2H), 5.01 (q, *J* = 6.9 Hz, 2H), 4.28 (q, *J* = 6.8 Hz, 2H), 3.62 (dd, *J* = 12.8, 9.4 Hz, 2H), 2.94 (d, *J* = 12.8 Hz, 2H), 2.77 (d, *J* = 7.4 Hz, 2H), 2.15 (s, 6H), 1.50 (d, *J* = 7.1 Hz, 6H), 1.37 (d, *J* = 7.1 Hz, 6H).

**$^{13}$C NMR** (101 MHz, $C_6D_6$) δ 212.8, 165.6 – 160.7 (m), 149.0, 147.7, 146.0, 145.8, 141.8, 141.3, 140.8, 135.6, 129.1, 129.0, 128.7, 127.4, 127.0, 126.6, 125.8, 124.6, 124.4, 120.3, 111.9 (d, $J$ = 25.4 Hz), 110.6 (d, $J$ = 25.4 Hz), 102.5, 101.5, 73.9, 50.6, 42.6, 38.9, 24.3, 21.9, 21.6.

**$^{19}$F NMR** (376 MHz, $C_6D_6$) δ -108.9 (t, $J$ = 8.3 Hz), -110.6 (t, $J$ = 8.0 Hz).

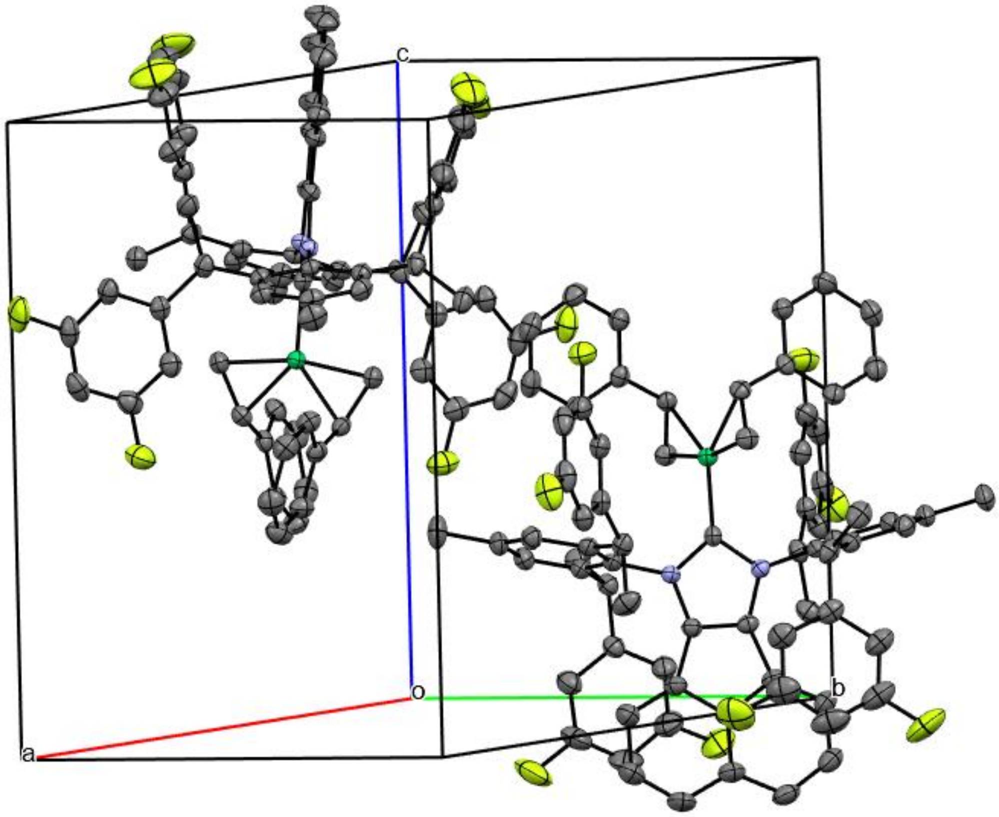


**Supplementary Fig. 5.** Unit cell of **Ni3** complex.

In order to visualize the π-π stacking of the chiral sidearm and acenaphthylene, the distance between the centroid of chiral side arms and plane of acenaphthylene are presented. In the unit cell of **Ni3** complex, distortion was not observed. And the measured distance is 3.347 Å and 3.311 Å (**Fig. S6**), which is highly symmetrical, which indicate that a strong π-π stacking occurs between chiral sidearm and acenaphthylene backbone[6], this result a rigid and well-defined chiral environment.

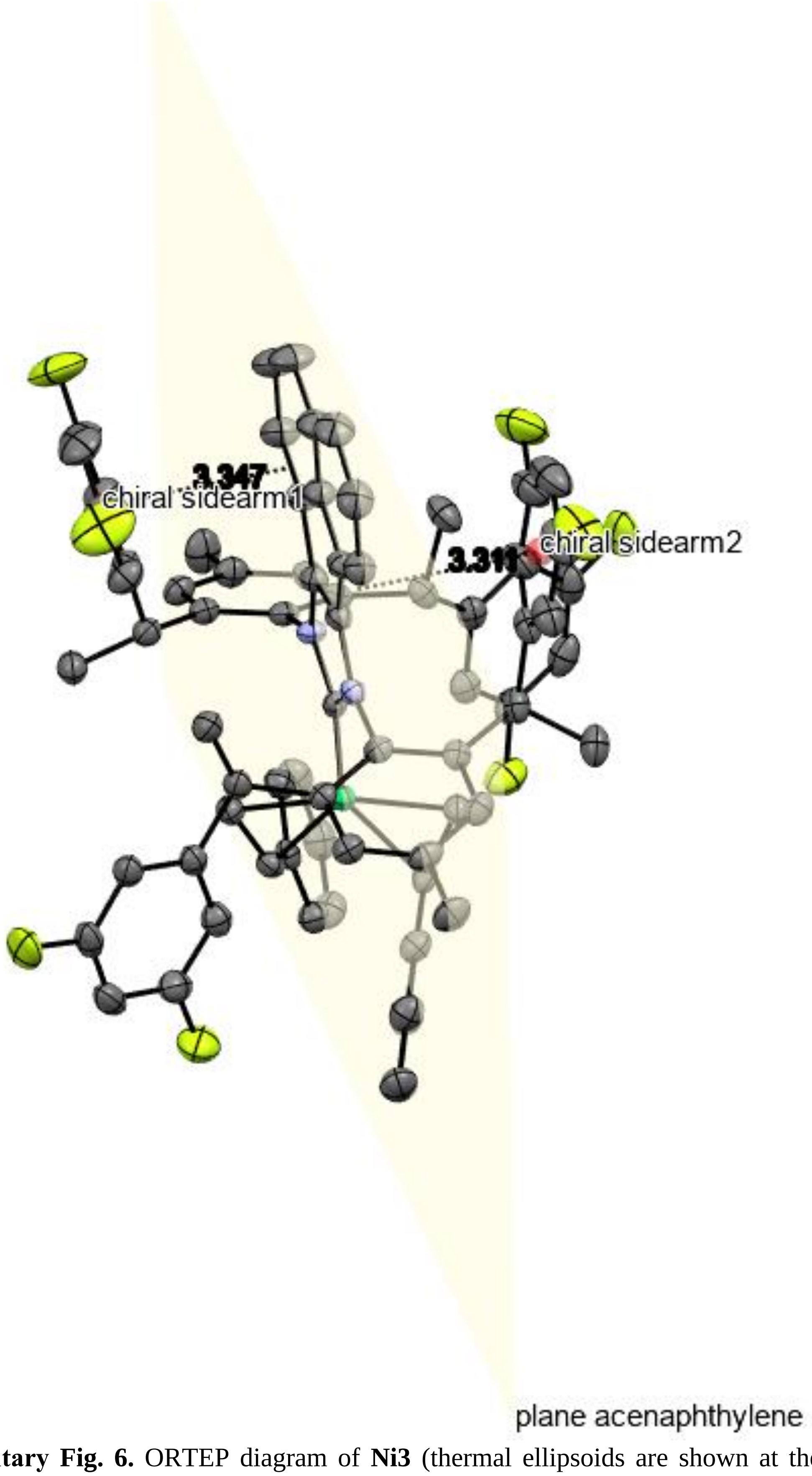


**Supplementary Fig. 6.** ORTEP diagram of **Ni3** (thermal ellipsoids are shown at the 50% probability level), for the reason of clarity all the hydrogen atoms are omitted. Measured distance: 3.347 Å and 3.311 Å.

**Calculation of percent Buried Volume (%$V_{bur}$).**

Calculations of percent buried volume were performed with the SambVca 2.1web tool[7,8].

The following parameters (set with respect to the parameters suggested by Nolan) were employed for the calculations:

- Center of sphere: NHC carbon
- Atoms for Z-axis definition: nitrogen atoms in NHC (Z-negative)
- Atoms for XZ plane definition: one of the nitrogen atoms in NHC
- All non-NHC atoms were deleted
- Bondii radii scaled by 1.17
- Sphere radius: 8.0 Å (expanded radius parameter)
- Distance of coordination point from center of sphere: 2.0 Å
- Mesh spacing for numerical integration: 0.1
- H atoms are included in calculation

The steric map of **Ni1**, **Ni2**, and **Ni3** are calculated.

**Optimization tables**

General procedure for reaction condition optimization.
In a nitrogen-filled glove box, a 2 mL vial equipped with a magnetic stirring bar was charged with bis(1,5-cyclooctadiene) nickel (3 mol%), *t*BuOK (3 mol%), and an imidazolium salt (3 mol%) in the presence of a solvent. The reaction mixture was stirred at room temperature for 30 minutes. Dialkyne (0.05 mmol) and nitrile (0.05 mmol) were then added to the reaction mixture. The vial was sealed and transferred outside the glovebox, allowing the reaction to stir at room temperature for 24 hours. The resulting mixture was filtered through a short pad of silica gel, eluting with EtOAc. The filtrate was concentrated in vacuo, and the yield was determined by crude NMR using $CH_2Br_2$ as an internal standard. The crude product was subsequently purified by flash silica gel column chromatography (using EtOAc in pentane) to obtain the desired product. Enantiomeric ratios (er) values were determined by chiral HPLC analysis.

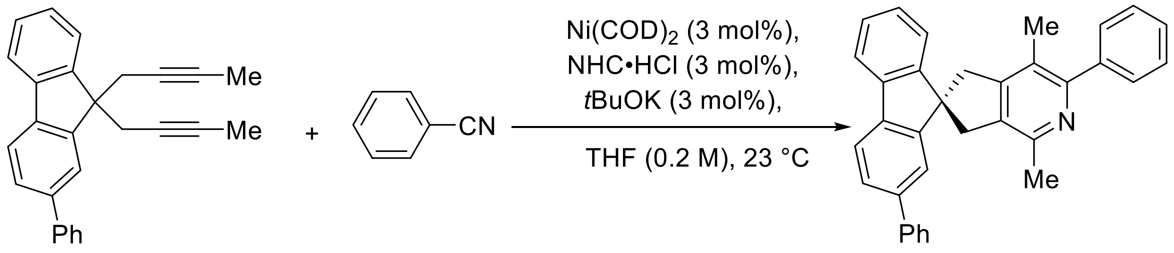


**1a** **7**

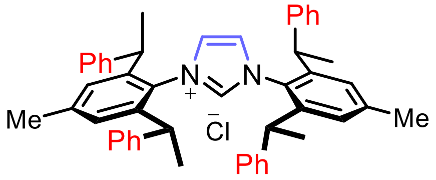

**L1**

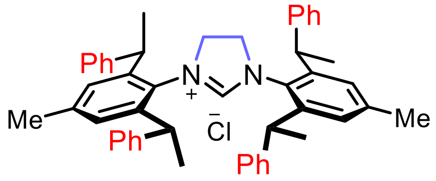

**L2**

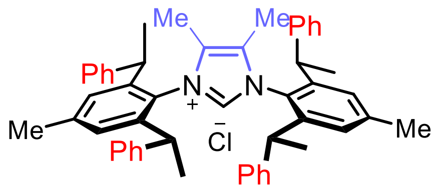

**L3**

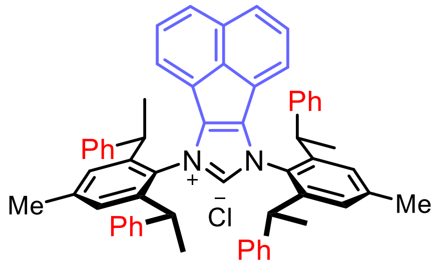

**L4**

**Supplementary Table 1.** Chiral carbene backbone investigations.

| Entry | NHC | % Conv.[a] | % **7** [a] | er of **7** [a] |
|---|---|---|---|---|
| 1 | L1 | 90 | 88 | 70:30 |
| 2 | L2 | 75 | 75 | 65:35 |
| 3 | L3 | 42 | 38 | 55:45 |
| 4 | L4 | 88 | 84 | 62:38 |

[a] Conversion and yield determined by crude NMR with $CH_2Br_2$ as internal standard. Enantiomeric ratio determined by chiral HPLC.

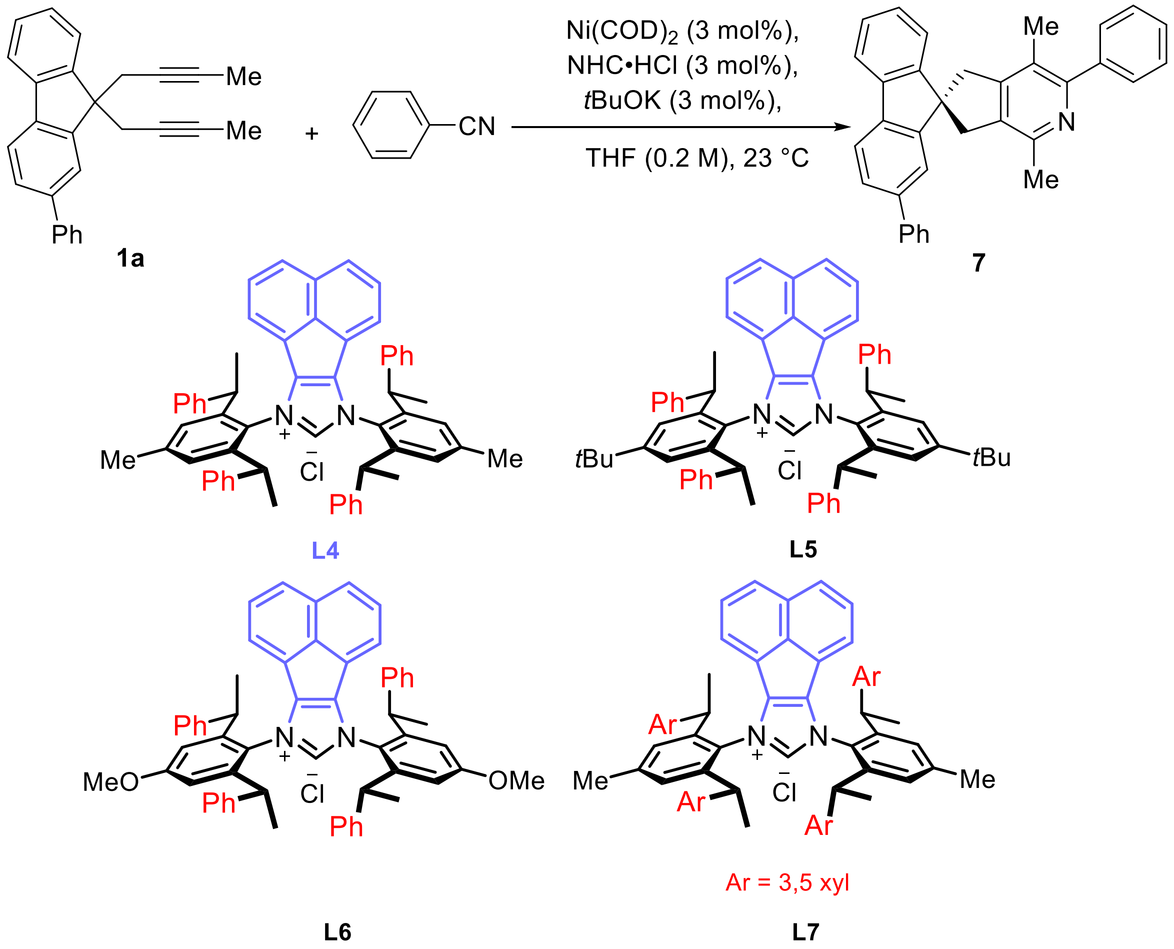


**Supplementary Table 2.** Chiral carbene ligand investigations.

| Entry | NHC | % Conv.[a] | % **7**[a] | er of **7**[a] |
|---|---|---|---|---|
| 1 | L4 | 90 | 84 | 62:38 |
| 2 | L5 | 90 | 88 | 60:40 |
| 3 | L6 | 88 | 84 | 62:38 |
| 4 | L7 | 20 | 14 | 66:34 |

[a] Conversion and yield determined by crude NMR with $CH_2Br_2$ as internal standard.

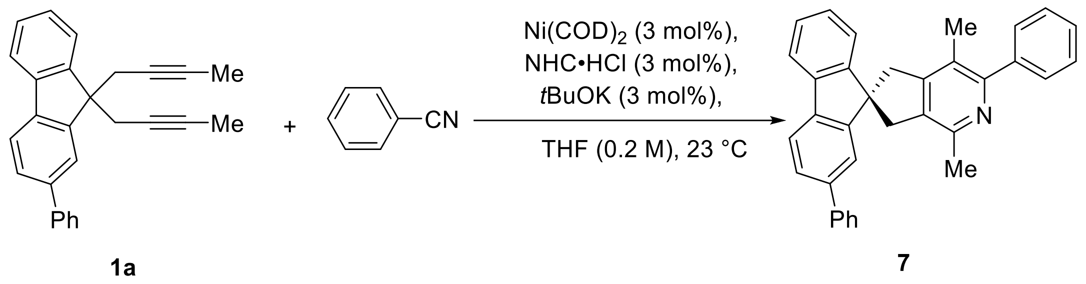


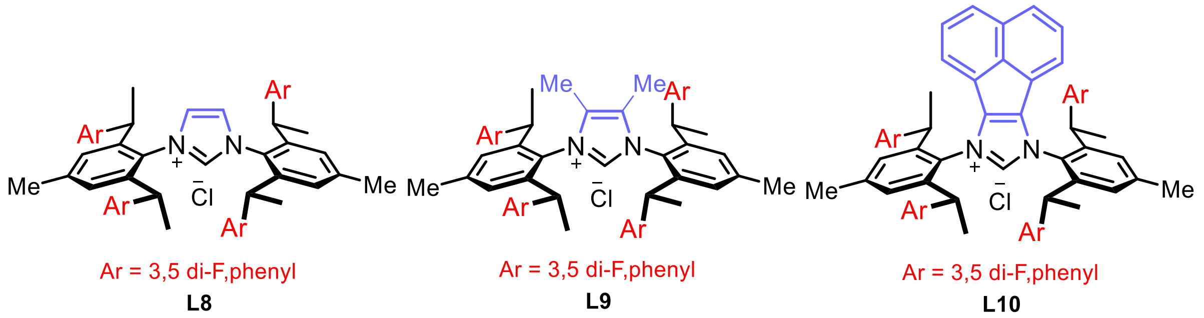


**Supplementary Table 3.** Chiral carbene backbone investigations (with electron deficient chiral side arm).

| Entry | NHC | % Conv.[a] | % **7**[a] | er of **7**[a] |
|---|---|---|---|---|
| 1 | L8 | 94 | 94 | 71:29 |
| 2 | L9 | 87 | 86 | 77:23 |
| 3 | L10 | 85 | 85 | 86:14 |

[a] Conversion and yield determined by crude NMR with $CH_2Br_2$ as internal standard. Enantiomeric ratio determined by chiral HPLC.

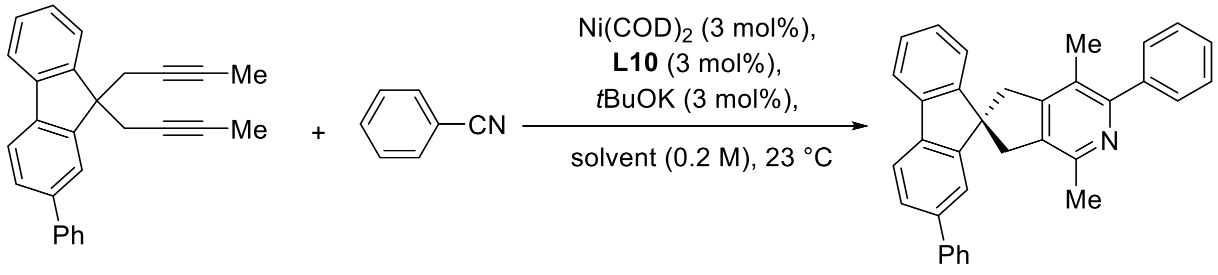


**1a** **7**

**Supplementary Table 4.** Solvent investigations.

| Entry | Solvent | % Conv.[a] | % **7**[a] | dr of **7**[a] |
|---|---|---|---|---|
| 1 | THF | 89 | 85 | 86:14 |
| 2 | TBME | 86 | 86 | 90:10 |
| 3 | CPME | 57 | 57 | 90:10 |
| 4 | 1,4 dioxane | 85 | 83 | 85:15 |
| 5 | 2-Me, THF | 85 | 85 | 89:11 |
| 6 | $PhCF_3$ | 86 | 80 | 90:10 |
| 7 | Toluene | 85 | 85 | 89.5:10.5 |
| 8 | Cyclohexane | 83 | 83 | 92:8 |
| 9 | Cyclopentane | 76 | 76 | 91:9 |
| 10 | Hexane | 100 | 100 | 90.5:9.5 |

[a] Conversion and yield determined by crude NMR with $CH_2Br_2$ as internal standard. Enantiomeric ratio determined by chiral HPLC.

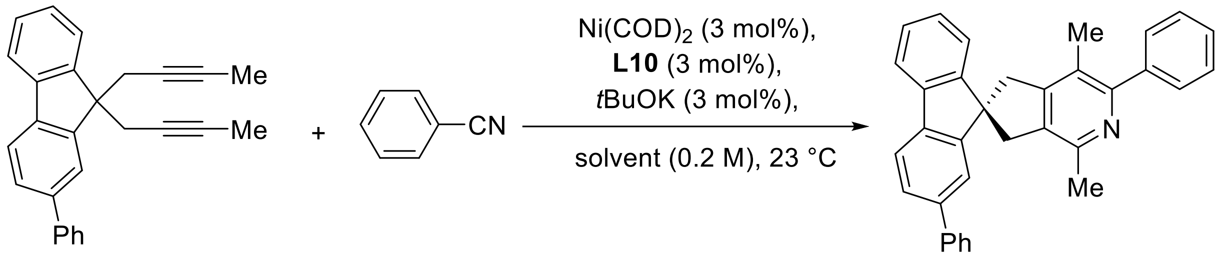


**1a**

**7**

**Supplementary Table 5.** Control experiments.

| Entry | deviation | % Conv.[a] | % **7**[a] | er of **7**[a] |
|---|---|---|---|---|
| 1 | No Ni(COD)2 | 0 | 0 | - |
| 2 | No NHC salt | 0 | 0 | - |
| 3 | **Ni3** was applie | 100 | 95 | 92:8 |

[a] Conversion and yield determined by crude NMR with $CH_2Br_2$ as internal standard. Enantiomeric ratio determined by chiral HPLC.

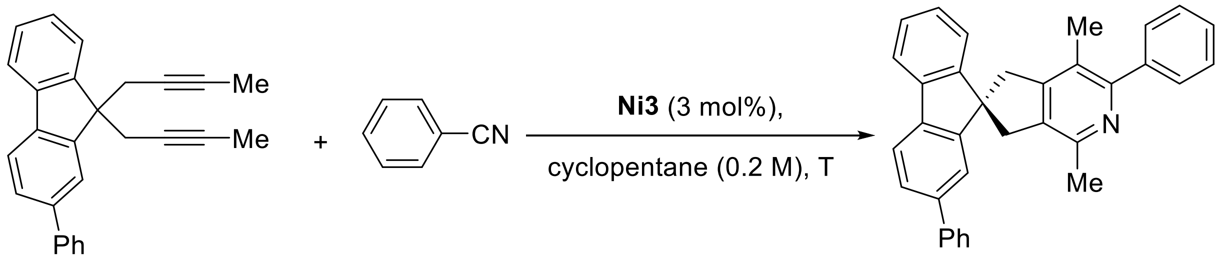


**1a**

**7**

**Supplementary Table 6.** Temperature screening.

| Entry | Temperature | % Conv.[a] | % **7**[a] | er of **7**[a] |
|---|---|---|---|---|
| 1 | 23 ºC | 90 | 88 | 91;9 |
| 2 | 10 ºC | 92 | 92 | 92:8 |
| 3 | 0 ºC | 33 | 30 | 92:8 |

[a] Conversion and yield determined by crude NMR with $CH_2Br_2$ as internal standard. Enantiomeric ratio determined by chiral HPLC.

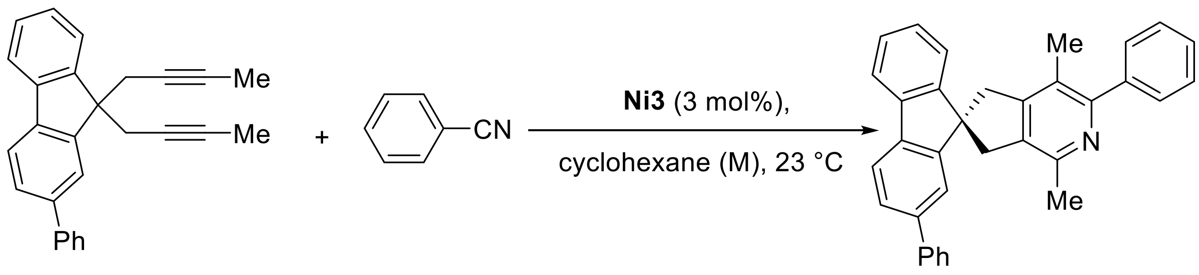


1a

7

**Supplementary Table 7.** Concentration screening.

| Entry | Concentratior | % Conv.[a] | % **7**[a] | er of **7**[a] |
|---|---|---|---|---|
| 1 | 0.2 M | 95 | 95 | 92:8 |
| 2 | 0.1 M | 83 | 82 | 92:8 |
| 3 | 0.05 M | 60 | 57 | 92:8 |

[a] Conversion and yield determined by crude NMR with $CH_2Br_2$ as internal standard. Enantiomeric ratio determined by chiral HPLC.

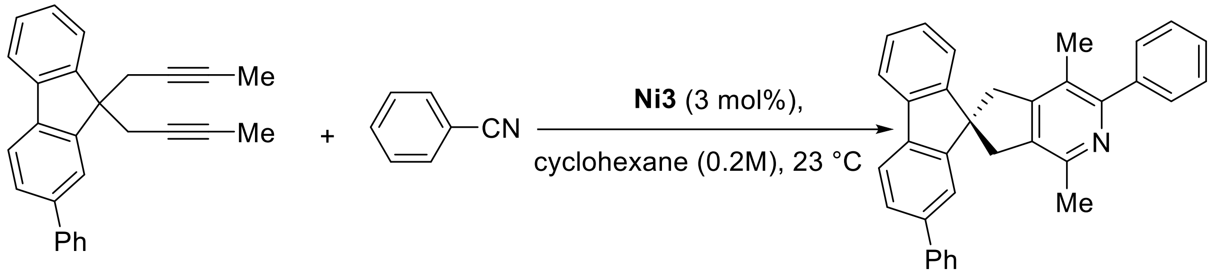


1a

7

**Supplementary Table 8.** Nickel loading screening.

| Entry | Ni loading | % Conv.[a] | % **7**[a] | er of **7**[a] |
|---|---|---|---|---|
| 1 | 3 mol% | 85 | 85 | 92:8 |
| 2 | 2 mol% | 51 | 51 | 92:8 |
| 3 | 1 mol% | 25 | 24 | 92:8 |

[a] Conversion and yield determined by crude NMR with $CH_2Br_2$ as internal standard. Enantiomeric ratio determined by chiral HPLC.

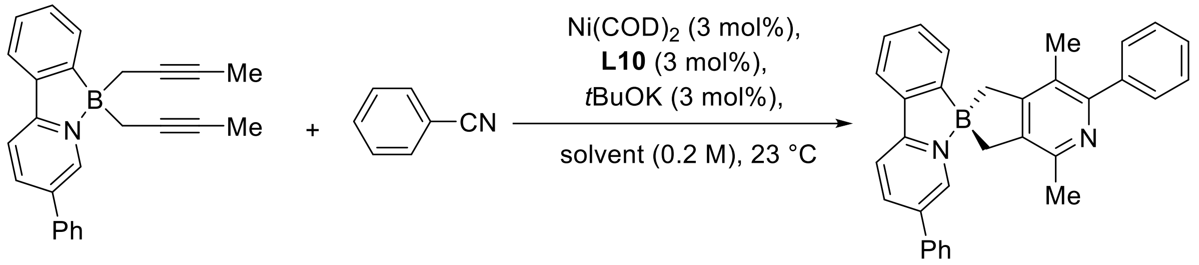


**3a** **9a**

**Supplementary Table 9.** Solvent investigations.

| Entry | Solvent | % Conv.[a] | % **9a**[a] | er of **9a**[a] |
|---|---|---|---|---|
| 1 | THF | 95 | 90 | 88:12 |
| 2 | TBME | 96 | 92 | 88:12 |
| 3 | CPME | 90 | 90 | 88:12 |
| 4 | 2-Me, THF | 93 | 90 | 89:11 |
| 5 | $PhCF_3$ | 88 | 85 | 89:11 |
| 6 | Toluene | 85 | 85 | 88:12 |
| 7 | Cyclohexane | 90 | 90 | 92:8 |
| 8 | Hexane | 100 | 100 | 90:10 |

[a] Conversion and yield determined by crude NMR with $CH_2Br_2$ as internal standard. Enantiomeric ratio determined by chiral HPLC.

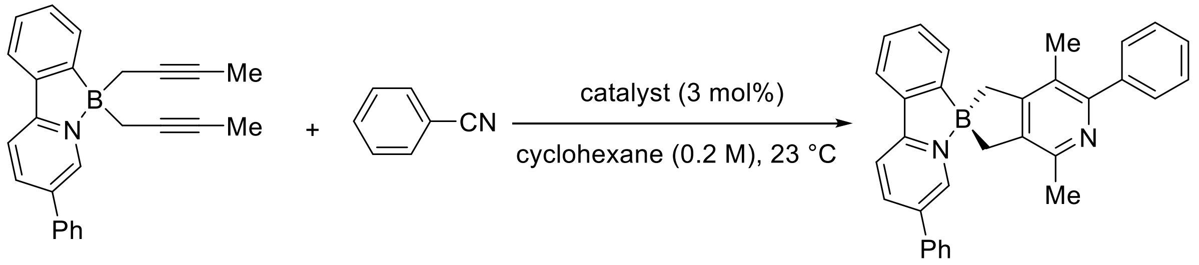


**3a** **9a**

**Supplementary Table 10.** Catalyst investigations.

| Entry | Catalyst | % Conv.[a] | % **9a**[a] | er of **9a**[a] |
|---|---|---|---|---|
| 1 | Ni1 | 95 | 90 | 53:47 |
| 2 | Ni2 | 83 | 80 | 70:30 |
| 3 | Ni3 | 95 | 95 | 92:8 |

[a] Conversion and yield determined by crude NMR with $CH_2Br_2$ as internal standard. Enantiomeric ratio determined by chiral HPLC.

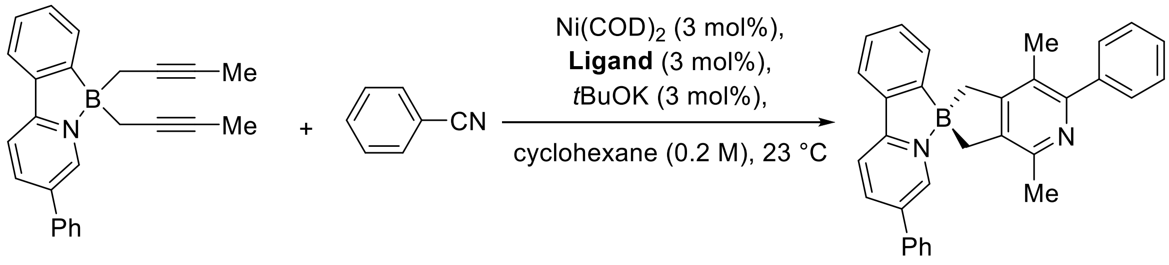


**3a** **9a**

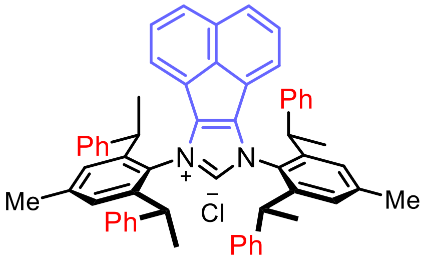

**L4**

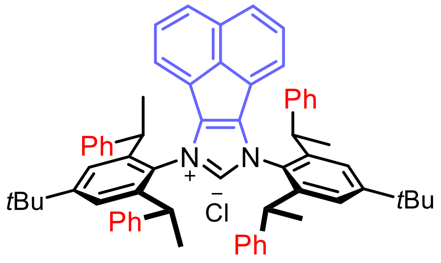

**L5**

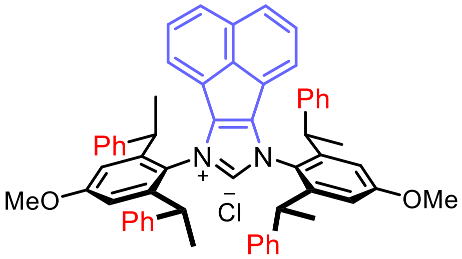

**L6**

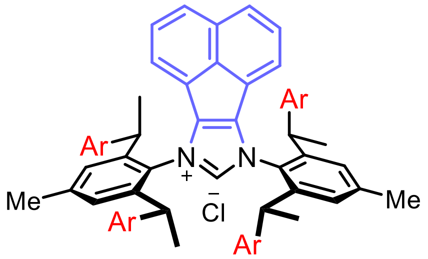

Ar = 3,5 xyl

**L7**

**Supplementary Table 11.** Chiral carbene ligand investigations.

| Entry | NHC | % Conv.[a] | % 9a[a] | er of **9a**[a] |
|---|---|---|---|---|
| 1 | L4 | 88 | 86 | 70:30 |
| 2 | L5 | 95 | 95 | 66:34 |
| 3 | L6 | 75 | 72 | 69:31 |
| 4 | L7 | <5 | <5 | - |

[a] Conversion and yield determined by crude NMR with $CH_2Br_2$ as internal standard.

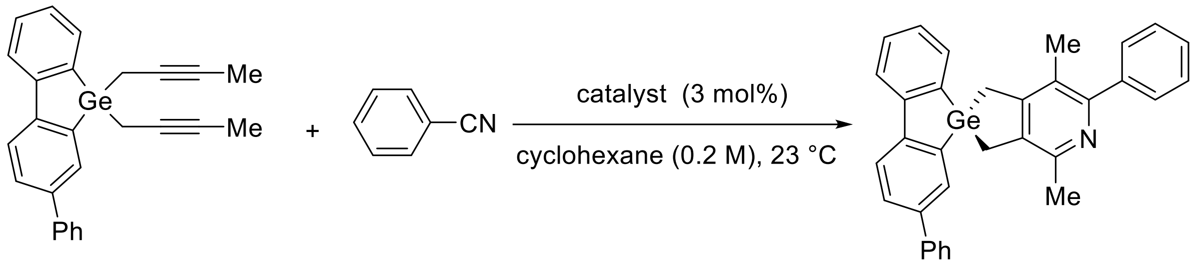

**5a** **10a**

**Supplementary Table 12.** Catalyst investigations.

| Entry | Catalyst | % Conv.[a] | % **10a**[a] | er of **10a**[a] |
|---|---|---|---|---|
| 1 | Ni1 | 80 | 80 | 50:50 |
| 2 | Ni2 | 85 | 83 | 50:50 |
| 3 | Ni3 | 85 | 85 | 50:50 |

[a] Conversion and yield determined by crude NMR with $CH_2Br_2$ as internal standard. Enantiomeric ratio determined by chiral HPLC.

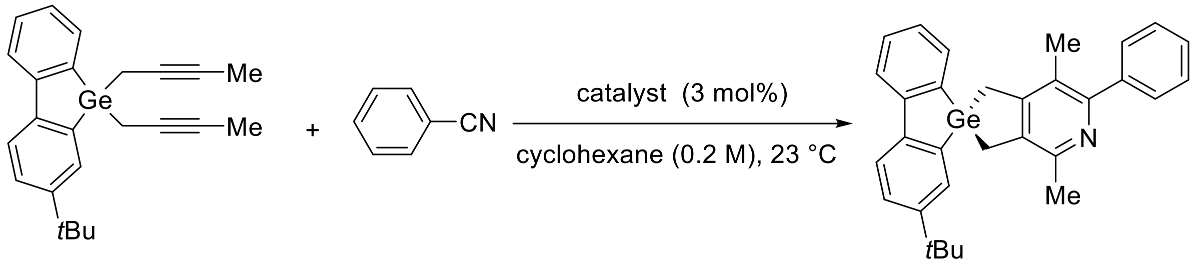

**5b** **10b**

**Supplementary Table 13.** Catalyst investigations.

| Entry | Catalyst | % Conv.[a] | % **10b**[a] | er of **10b**[a] |
|---|---|---|---|---|
| 1 | Ni1 | 90 | 90 | 50:50 |
| 2 | Ni2 | 85 | 75 | 71:29 |
| 3 | Ni3 | 91 | 91 | 80:20 |

[a] Conversion and yield determined by crude NMR with $CH_2Br_2$ as internal standard. Enantiomeric ratio determined by chiral HPLC.

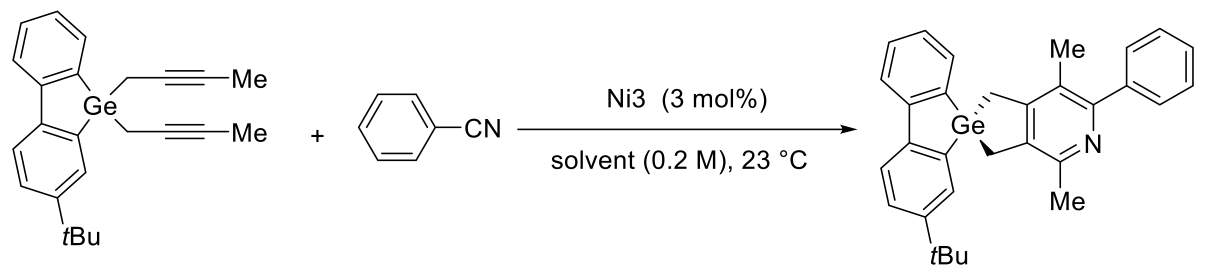


**5b** **10b**

**Supplementary Table 14.** Solvent investigations.

| Entry | Solvent | % Conv.[a] | % **10b**[a] | er of **10b**[a] |
|---|---|---|---|---|
| 1 | cyclohexane | 91 | 91 | 80:20 |
| 2 | THF | 80 | 80 | 78:22 |
| 3 | Toluene | 95 | 91 | 75:25 |

[a] Conversion and yield determined by crude NMR with $CH_2Br_2$ as internal standard. Enantiomeric ratio determined by chiral HPLC.

**Starting material synthesis**

General overview for fluorene derivatives synthesis

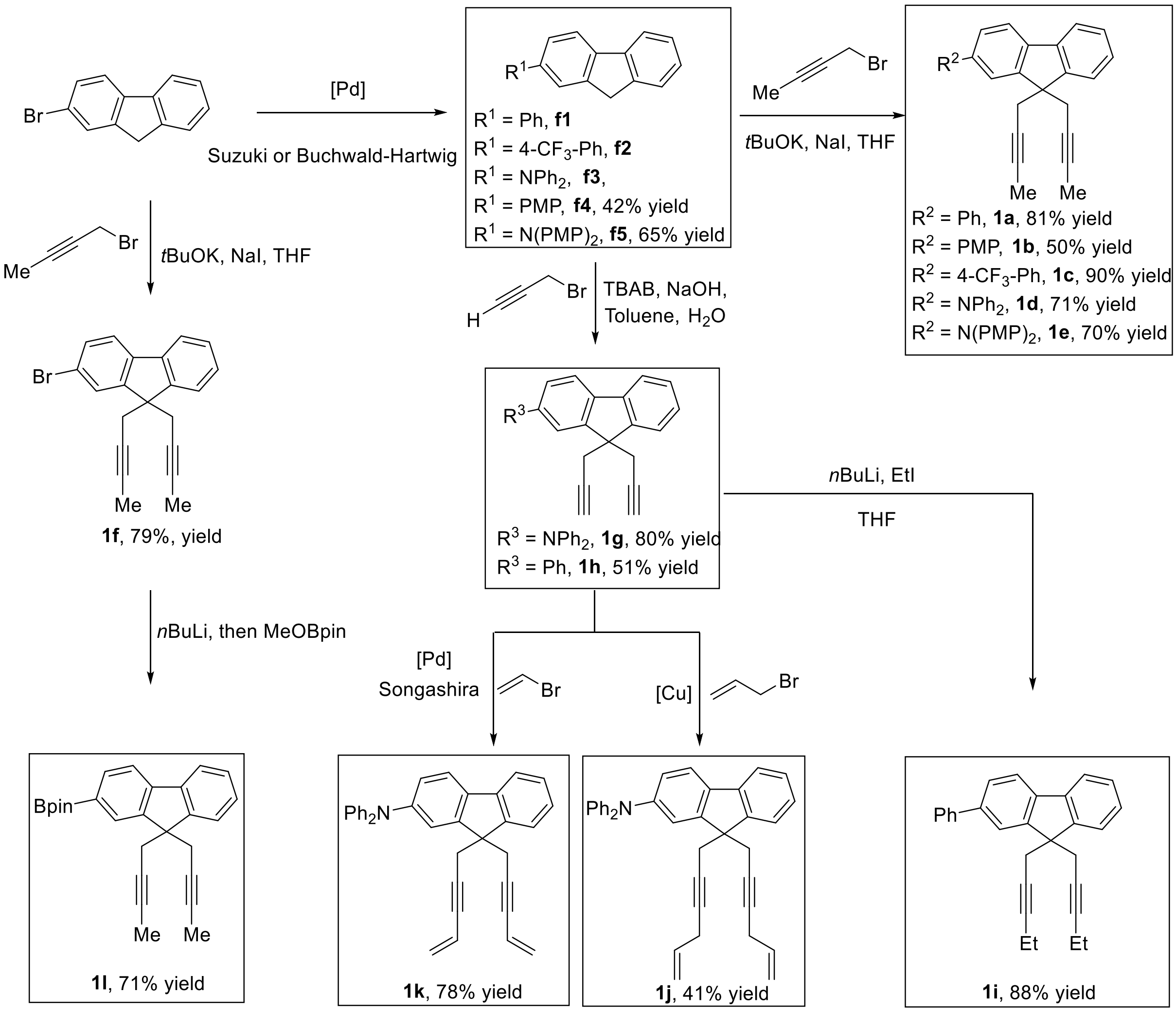


Detailed experimental procedures are shown below, and all the unknow compounds are characterized.

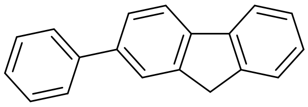

**f1**

Compound **f1** was synthesized according to literature[9].

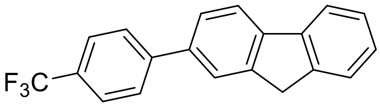


**f2**

Compound **f2** was synthesized according to literature[10].

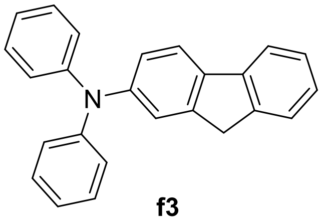


**f3**

Compound **f3** was synthesized according to literature[11].

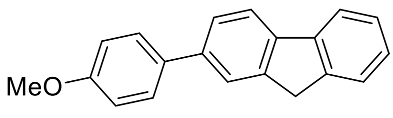


**f4**

**Name:** 2-(4-methoxyphenyl)-9*H*-fluorene (**f4**):

**Synthesis**:

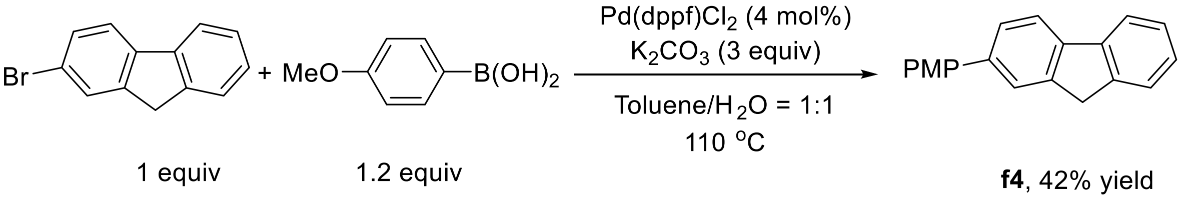


Following a slightly modified procedure from above mentioned literature[9-11]. In a flame-dried two-necked flask equipped with a reflux condenser, 2-bromo-9*H*-fluorene (1225.5 mg, 5 mmol, 1 eq), corresponding boronic acid (911.7 mg, 6 mmol, 1.2 eq), $K_2CO_3$ (2073.0 mg, 15 mmol, 3 eq) and $Pd(dppf)Cl_2$ (146.1 mg, 0.2 mmol, 4 mol%) were added and vacuum flashed with $N_2$ (3x). Toluene: $H_2O$ 1:1 (25 mL) was added. The reaction mixture was refluxed at 110 $^oC$ for 24 h. The reaction mixture was diluted with water and EtOAc at rt. The aqueous phase was extracted with EtOAc. The combined organic phases were dried over $MgSO_4$, filtered and concentrated under reduced pressure. The resulting crude product was purified *via* flash chromatography (EtOAc: Pentane/1:25) to afford **f4** (567.0 mg, 2.1 mmol, 42% yield) as a white solid.

**$^1$H NMR** (400 MHz, $CDCl_3$) δ 7.82 (t, J = 7.5 Hz, 2H), 7.74 (s, 1H), 7.63 – 7.55 (m, 4H), 7.40 (t, 1H), 7.32 (t, 1H), 7.05 – 6.96 (m, 2H), 3.96 (s, 2H), 3.87 (s, 3H).

**$^{13}$C NMR** (101 MHz, $CDCl_3$) δ 159.2, 144.0, 143.5, 141.6, 140.5, 139.6, 134.1, 128.3, 126.9, 126.7, 125.6, 125.1, 123.4, 120.2, 119.9, 114.3, 55.5, 37.1.

**HRMS** (APPI/LTQ-Orbitrap) m/z:$[M]^+$ Calcd for $C_{20}H_{16}O^+$ 272.1196; Found 272.1195.

**IR**: 3054, 3007, 2935, 2836, 2362, 1605, 1582, 1519, 1484, 1467, 1455, 1442, 1408, 1312, 1294, 1265, 1251, 1181, 1114, 1042, 1029, 842, 822, 769, 735, 704, 585, 536, 422.

**Melting point**: 225-226 $^oC$.

**$R_f$**: 0.81 (ethyl acetate: pentane = 1:4).

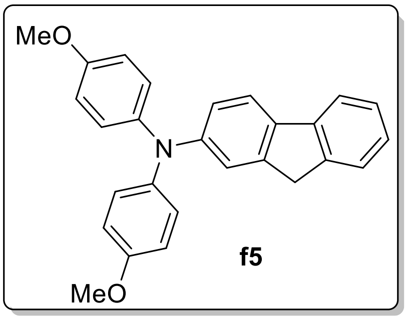


**Name**: *N*,*N*-bis(4-methoxyphenyl)-9*H*-fluorene-2-amine (**f5**):

**Synthesis**:

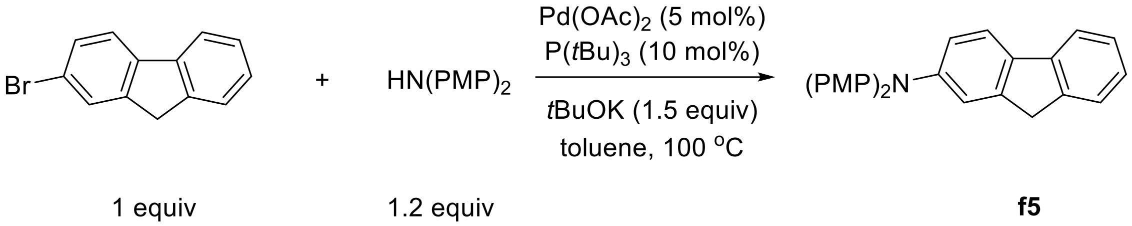


A mixture of 2-bromo-9*H*-fluorene (1225.5 mg, 5 mmol, 1 eq), $Pd(OAc)_2$ (56.1 mg, 0.25 mmol, 5 mol%), $P(tBu)_3{\cdot}HBF_4$ (145.01 mg, 0.5 mmol, 10 mol%), bis(4- methoxyphenyl)amine (1375.7 mg, 6 mmol, 1.2 eq), and sodium *tert*-butoxide (720 mg, 7.5 mmol, 1.5 eq) was placed in a Schlenk flask. The flask was subjected to three vacuum/nitrogen refill cycles to remove water and oxygen. Anhydrous toluene (40 mL) was added, and the mixture was stirred overnight at 100 °C or until the reaction was complete by TLC analysis. After cooling, the reaction was quenched by water, followed by product extraction with ethyl acetate. The organic layer was dried over anhydrous $Mg_2SO_4$ and evaporated under vacuum. Silica gel column chromatography (hexane/EtOAc, v/v, 5:1) further purified the collected residue to give **f5** (1278.0 mg, 3.25 mmol, 65% yield) as pink solid.

**$^1$H NMR** (400 MHz, $CDCl_3$) δ 7.66 (d, *J* = 7.8 Hz, 1H), 7.57 (d, *J* = 8.3 Hz, 1H), 7.47 (d, *J* = 7.5 Hz, 1H), 7.32 (t, *J* = 7.6 Hz, 1H), 7.21 (t, *J* = 7.4 Hz, 1H), 7.13 (s, 1H), 7.07 (d, *J* = 9.0 Hz, 4H), 6.98 (d, *J* = 10.3 Hz, 1H), 6.83 (d, *J* = 8.9 Hz, 4H), 3.81 (s, 6H), 3.77 (s, 2H).

**$^{13}$C NMR** (101 MHz, $CDCl_3$) δ 155.7, 148.2, 144.7, 143.0, 141.9, 141.6, 135.1, 126.8, 126.4, 125.7, 125.0, 120.6, 120.3, 119.2, 118.2, 114.8, 55.7, 37.1.

**HRMS** (ESI/QTOF) m/z: $[M]^+$ Calcd for $C_{27}H_{23}NO_2^+$ 393.1723; Found 393.1716.

**IR:** 3055, 3000, 2360, 2344, 2055, 850, 780, 765, 697, 683, 667, 563, 520, 508.

**Melting point**: 80-81 °C.

**$R_f$**: 0.73 (ethyl acetate: pentane = 1:4).

**General procedure A:**

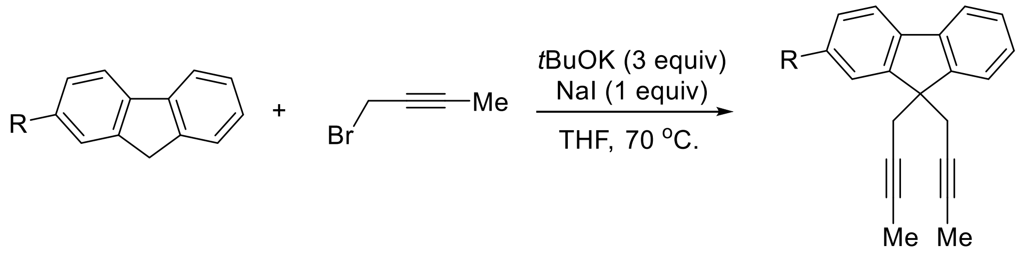


**f** 1 equiv 3 equiv **1**

Under $N_2$ atmosphere, a stirred mixture of fluorene substrate (1 eq), NaI (1 eq) and *t*BuOK (3 eq) in THF (0.2 M) was added 1-Bromo-2-butyne (3 eq). After stirred at 70 °C for 24 h the reaction was allowed to cool to room temperature and quenched by addition of water. The mixture was extracted with EtOAc (×3). The organic phase was dried with $Na_2SO_4$, solvents were evaporated, and the residue was purified by flash column chromatography on silica gel.

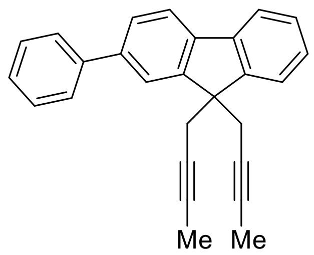

1a

**Name**: 9,9-di(but-2-yn-1-yl)-2-phenyl-9*H*-fluorene (**1a**).

**Synthesis**: Following General procedure A, (using 2-phenyl-9*H*-fluorene 1500 mg, 6.19 mmol; 1-bromobut-2-yne 2470 mg, 18.57 mmol; *t*BuOK 2083.5 mg, 18.6 mmol; NaI, 928.5 mg, 6.19 mmol) the desired product **1a** was obtained in 1746.0 mg, 5.04 mmol, 81% yield as a pale-yellow solid.

**$^1$H NMR** (400 MHz, $CDCl_3$) δ 8.02 (s, 1H), 7.81 – 7.72 (m, 3H), 7.68 (d, *J* = 7.9 Hz, 2H), 7.63 (dd, *J* = 7.9, 1.9 Hz, 1H), 7.47 (t, *J* = 7.7 Hz, 2H), 7.44 – 7.30 (m, 3H), 2.79 (q, *J* = 2.6 Hz, 4H), 1.84 (t, *J* = 2.6 Hz, 6H).

**$^{13}$C NMR** (101 MHz, $CDCl_3$) δ 150.1, 149.8, 141.8, 140.1, 139.7, 139.3, 128.9, 127.9, 127.3, 127.2, 126.9, 124.2, 123.2, 120.1, 120.0, 78.0, 76.4, 51.0, 27.9, 3.8.

**HRMS** (APPI/LTQ-Orbitrap) m/z: $[M]^+$ Calcd for $C_{27}H_{22}^+$ 346.1716; Found 346.1716.

**IR (ATR)**: $\tilde{\nu}$ ($cm^{-1}$) = 3058, 3031, 2916, 1599, 1482, 1468, 1453, 1425, 834, 758, 741, 698.

**Melting point**: 107-108 °C

**$R_f$**: 0.63 (ethyl acetate: pentane = 1:10)

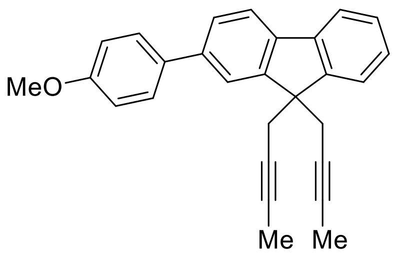


**1b**

**Name**: 9,9-di(but-2-yn-1-yl)-2-(4-methoxyphenyl)-9*H*-fluorene (**1b**).

**Synthesis**: Following General procedure A, (using 2-(4-methoxyl-phenyl)-9*H*-fluorene 435.2 mg, 1.60 mmol; 1-bromobut-2-yne 638.4 mg, 4.8 mmol; *t*BuOK 537.6 mg, 4.8 mmol; NaI, 240 mg, 1.60 mmol) the desired product **1b** was obtained in 318.0 mg, 0.8 mmol, 50% as a white solid.

**$^1$H NMR** (400 MHz, $CDCl_3$) δ 7.97 (s, 1H), 7.80 – 7.69 (m, 3H), 7.65 – 7.53 (m, 3H), 7.39 (t, J = 1.2 Hz, 1H), 7.32 (t, 1H), 7.05 – 6.97 (m, 2H), 3.87 (s, 3H), 2.81 (m, 4H), 1.84 (t, J = 2.5 Hz, 6H).

**$^{13}$C NMR** (101 MHz, $CDCl_3$) δ 159.2, 150.0, 149.7, 139.8, 139.7, 138.7, 134.3, 128.2, 127.8, 127.0, 126.4, 124.1, 122.6, 120.1, 119.8, 114.3, 77.9, 76.4, 55.5, 50.9, 27.8, 3.8.

**HRMS** (APPI/LTQ-Orbitrap) m/z: $[M + H]^+$ Calcd for $C_{28}H_{25}O^+$ 377.1900; Found 377.1908.

**IR**: 2955, 2916, 2834, 1736, 1607, 1517, 1486, 1466, 1453, 1429, 1406, 1294, 1245, 1180, 1045, 1034, 1024, 843, 824, 778, 757, 740, 674, 582.

**Melting point**: 199-200 $^o$C

**$R_f$**: 0.19 (dichloromethane: pentane = 1:4)

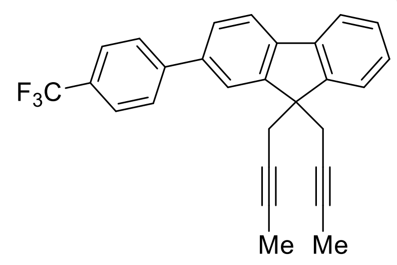


**1c**

**Name**: 9,9-di(but-2-yn-1-yl)-2-(4-(trifluoromethyl)phenyl)-9*H*-fluorene (**1c**).

**Synthesis**: Following General procedure A, (using 2-(4-$CF_3$-phenyl)-9*H*-fluorene 448.2 mg, 1.44 mmol; 1-bromobut-2-yne 574.6 mg, 4.32 mmol; *t*BuOK 483.84 mg, 4.32 mmol; NaI, 240 mg, 1.60 mmol) the desired product **1c** was obtained in 540.0 mg, 1.3 mmol, 90% as a yellow solid.

**$^1$H NMR** (400 MHz, $CDCl_3$) δ 8.03 (s, 1H), 7.83 – 7.69 (m, 7H), 7.63 (d, J = 8.0 Hz, 1H), 7.46 – 7.31 (m, 2H), 2.87 – 2.72 (m, 4H), 1.84 (s, 6H).

**$^{13}$C NMR** (101 MHz, $CDCl_3$) δ 150.3, 149.9, 145.3, 140.3, 139.4, 138.5, 129.3 (q, *J* = 32.4 Hz), 128.0, 127.6, 127.5, 127.1, 125.9 (q, *J* = 4.0 Hz), 124.2, 123.3, 120.3, 120.2, 78.1, 76.2, 51.0, 27.9, 3.8.

**$^{19}$F NMR** (376 MHz, $CDCl_3$) δ -62.30.

**HRMS** (APPI/LTQ-Orbitrap) m/z: $[M]^+$ Calcd for $C_{28}H_{21}F_3^+$ 414.1590; Found 414.1591.

**IR**: 3066, 2918, 1737, 1616, 1467, 1453, 1426, 1401, 1377, 1323, 1282, 1254, 1165, 1122, 1070, 1015, 900, 851, 826, 779, 757, 740, 648, 635, 594, 563, 405.

**Melting point**: 138-139 $^o$C

**$R_f$**: 0.63 (ethyl acetate: pentane = 1:10)

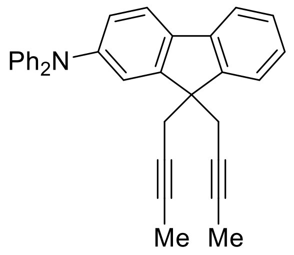


**1d**

**Name**: 9,9-di(but-2-yn-1-yl)-*N*,*N*-diphenyl-9*H*-fluoren-2-amine (**1d**).

**Synthesis**: Following General procedure A, (using 2-( *N*,*N*-diphenylamino)-9*H*-fluorene 2625.2 mg, 7.87 mmol; 1-bromobut-2-yne 3140.1 mg, 23.61 mmol; *t*BuOK 2644.3 mg, 23.61 mmol; NaI, 1180.5 mg, 7.87 mmol) the desired product **1d** was obtained in 2446.0 mg, 5.590 mmol, 71% yield as a pale-yellow solid.

**$^1$H NMR** (400 MHz, $CDCl_3$) δ 7.69 (d, *J* = 7.5 Hz, 1H), 7.64 (d, *J* = 7.5 Hz, 1H), 7.58 (d, *J* = 8.3 Hz, 1H), 7.54 (d, *J* = 2.1 Hz, 1H), 7.37 (td, *J* = 7.5, 1.3 Hz, 1H), 7.33 – 7.23 (m, 5H), 7.21 – 7.14 (m, 4H), 7.11 (dd, *J* = 8.3, 2.3 Hz, 1H), 7.07 – 7.00 (m, 2H), 2.68 (qq, *J* = 16.3, 2.6 Hz, 4H), 1.64 (t, *J* = 2.6 Hz, 6H).

**$^{13}$C NMR** (101 MHz, $CDCl_3$) δ 150.8, 149.4, 148.1, 147.2, 139.9, 134.6, 129.3, 127.8, 126.3, 124.5, 123.9, 123.5, 122.8, 120.3, 120.2, 119.3, 77.9, 76.2, 50.7, 27.7, 3.5.

**HRMS** (ESI/QTOF) m/z: $[M + H]^+$ Calcd for $C_{33}H_{28}N^+$ 438.2216; Found 438.2208.
**IR (ATR)**: $\tilde{\nu}$ ($cm^{-1}$) = 3035, 2916, 1584, 1486, 1468, 1450, 1424, 1347, 1329, 1271, 1218, 1174, 1154 ,1121, 1075, 1028, 907, 827, 753, 730, 696, 648, 618, 563, 513, 479, 468, 448, 428.

**Melting point**: 108-109 °C

**$R_f$**: 0.66 (ethyl acetate: pentane = 1:10)

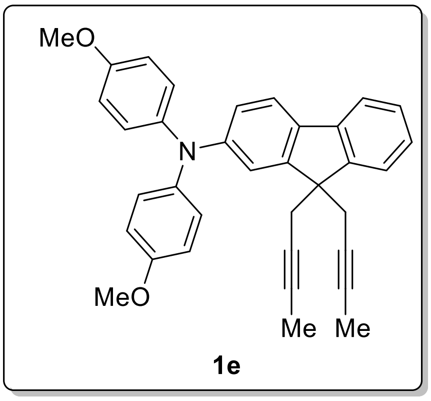


**Name**: 9,9-di(but-2-yn-1-yl)-*N*,*N*-(bis(4-methoxyphenyl)amino)-9*H*-fluoren-2-amine (**1e**).

**Synthesis**: Following General procedure A, (using 2-(*N*,*N*-bis(4-methoxyphenyl)amino)-9*H*-fluorene 1990.6 mg, 5.06 mmol; 1-bromobut-2-yne 2018.9 mg, 15.18 mmol; *t*BuOK 1700.2 mg, 15.18 mmol; NaI, 759 mg, 5.06 mmol) the desired product **1e** was obtained (1760.0 mg, 3.54 mmol, 70% yield) as a pink solid.

**$^{1}$H NMR** (400 MHz, $CDCl_3$) δ 7.66 (d, *J* = 7.5 Hz, 1H), 7.58 (d, *J* = 7.5 Hz, 1H), 7.48 (d, *J* = 8.3 Hz, 1H), 7.38 (d, *J* = 2.1 Hz, 1H), 7.32 (td, *J* = 7.4, 1.2 Hz, 1H), 7.21 (td, *J* = 7.5, 1.3 Hz, 1H), 7.13 – 7.06 (m, 4H), 6.94 (dd, *J* = 8.3, 2.2 Hz, 1H), 6.87 – 6.80 (m, 4H), 3.80 (s, 6H), 2.72 – 2.55 (m, 4H), 1.65 (t, *J* = 2.5 Hz, 6H).

**$^{13}$C NMR** (101 MHz, $CDCl_3$) δ 155.8, 150.8, 149.2, 148.3, 141.4, 140.1, 132.7, 127.8, 126.6, 125.8, 123.9, 120.4, 120.1, 119.0, 117.0, 114.8, 77.8, 76.3, 55.6, 50.5, 27.7, 3.6.

**HRMS** (APPI/LTQ-Orbitrap) m/z: $[M]^+$ Calcd for $C_{35}H_{32}NO_2^+$ 498.2428; Found 498.2419.

**IR (ATR)**: $\tilde{\nu}$ ($cm^{-1}$) = 3038, 3000, 2951, 2916, 2834, 1607, 1574, 1504, 1452, 1349, 1325, 1288, 1239, 1180, 1121, 1106, 1035, 910, 828, 756, 732, 648, 596, 565, 532.

**Appearance**: pink solid.

**Melting point**: 149-150 °C

**$R_f$**: 0.81 (ethyl acetate: pentane = 1:2)

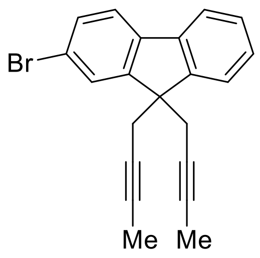


**1f**

**Name**: 2-bromo-9,9-di(but-2-yn-1-yl)-9*H*-fluorene (**1f**).

**Synthesis**: Following General procedure A, (using 2-(bromo)-9*H*-fluorene 1210.8 mg, 4.94 mmol; 1-bromobut-2-yne 1971.0 mg, 14.82 mmol; *t*BuOK 1659.8 mg, 14.82 mmol; NaI, 741 mg, 4.94 mmol) the desired product **1f** was obtained in 1511 mg, 4.3 mmol, 87% yield as pale-yellow solid.

**$^1$H NMR** (400 MHz, $CDCl_3$) δ 7.86 (d, J = 1.9 Hz, 1H), 7.74 – 7.64 (m, 2H), 7.57 (d, J = 8.1 Hz, 1H), 7.50 (m, 1H), 7.43 – 7.28 (m, 2H), 2.80 – 2.64 (m, 4H), 1.80 (t, J = 2.5 Hz, 6H).

**$^{13}$C NMR** (101 MHz, $CDCl_3$) δ 151.3, 149.2, 139.1, 139.0, 130.9, 128.0, 127.7, 127.6, 124.0, 121.1, 120.8, 119.9, 78.3, 75.7, 51.2, 27.7, 3.7.

**HRMS** (APPI/LTQ-Orbitrap) m/z: $[M + H]^+$ Calcd for $C_{21}H_{17}Br^+$ 348.0514; Found 348.0586.

**IR**: 3061, 2916, 2853, 1569, 1480, 1467, 1442, 1405, 1377, 1318, 1293, 1258, 1177, 1064, 1031, 1005, 880, 823, 788, 772, 756, 738, 563, 473, 459, 430.

**Melting point**: 101-102 $^o$C.

**R$_f$**: 0.57 (dichloromethane: pentane = 1:4).

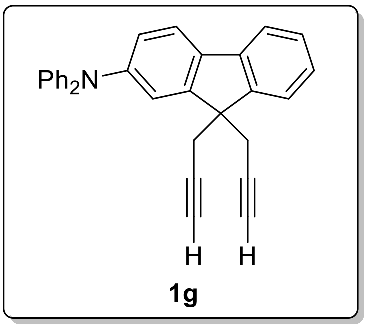

**Name**: *N*,*N*-diphenyl-9,9-di(prop-2-yn-1-yl)-9H-fluoren-2-amine (**1g**).

**Synthesis**:

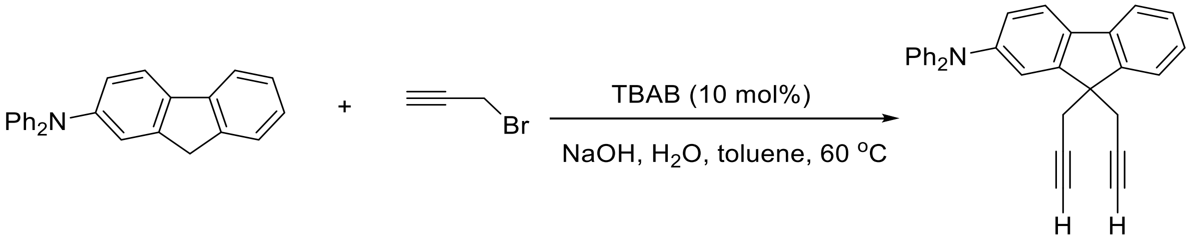


**f3** 1 equiv    3 equiv    **1g**

Following a slightly modified procedure from literature[12]. Add propargyl bromide (~80% in toluene, 4461 mg, 30 mmol, 3 eq) and NaOH aqueous (12.5 M in water, 10 mL) to a stirred mixture of fluorene **f3** (3330 mg, 10 mmol, 1 eq) and *n*-$Bu_4NBr$ (322 mg, 1.0 mmol, 10 mol%) in toluene (20 mL) at 25 °C. Stir the mixture at 60 °C for 24 hours. Remove the solvents under reduced pressure. Purify the residue by flash column chromatography on silica gel and the desired product **1g** was obtained (3273 mg, 8 mmol, 80% yield) as a pale-yellow solid.

**$^{1}$H NMR** (400 MHz, $CDCl_3$) δ 7.72 (d, *J* = 7.5 Hz, 1H), 7.63 (d, *J* = 7.6 Hz, 1H), 7.56 (d, *J* = 8.3 Hz, 1H), 7.49 (d, *J* = 2.1 Hz, 1H), 7.37 (td, *J* = 7.4, 1.1 Hz, 1H), 7.31 – 7.23 (m, 6H), 7.15 (dd, *J* = 8.6, 1.3 Hz, 4H), 7.08 (dd, *J* = 8.1, 2.1 Hz, 1H), 7.02 (tt, *J* = 7.1, 1.3 Hz, 2H), 2.84 – 2.68 (m, 4H), 1.87 (t, *J* = 2.6 Hz, 2H).

**$^{13}$C NMR** (101 MHz, $CDCl_3$) δ 149.7, 148.5, 147.9, 147.4, 140.0, 134.5, 129.4, 128.2, 126.5, 124.6, 123.8, 123.5, 123.0, 120.5, 119.5, 119.4, 81.0, 70.9, 50.0, 27.6.

**HRMS** (ESI/QTOF) m/z: $[M]^+$ Calcd for $C_{31}H_{23}N^+$ 409.1825; Found 409.1828.

**IR (ATR)**: $\tilde{\nu}$ ($cm^{-1}$) = 3296, 3058, 3034, 1586, 1488, 1469, 1451, 1425, 1347, 1320, 1276, 909, 827, 775, 754, 736, 698, 644, 514.

**Melting point**: 131-132 °C

**$R_f$**: 0.64 (ethyl acetate: pentane = 1:10).

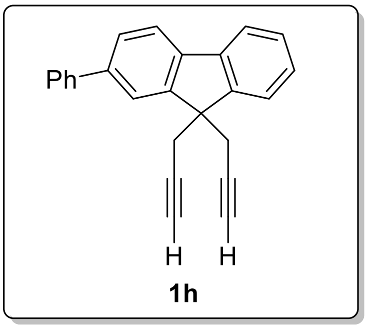


**Name**: 2-phenyl-9,9-di(prop-2-yn-1-yl)-9*H*-fluorene (**1h**).

**Synthesis**:

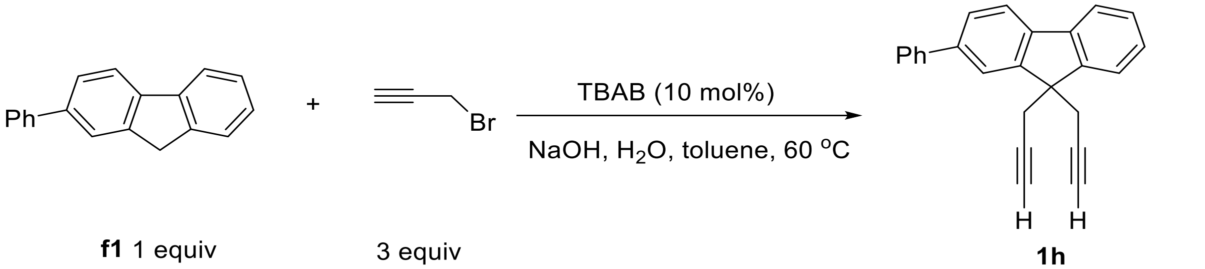


Following a slightly modified procedure from literature[12]. Add propargyl bromide (~80% in toluene, 2209 mg, 18.57 mmol, 3 eq) and NaOH aqueous (12.5 M in water, 6 mL) to a stirred mixture of fluorene **f1** (1500 mg, 6.190 mmol, 1 eq) and *n*-$Bu_4NBr$ (199 mg, 0.6910 mmol, 10 mol%) in toluene (12 mL) at 25 °C. Stir the mixture at 60 °C for 24 hours. Remove the solvents under reduced pressure. Purify the residue by flash column chromatography on silica gel and the desired product **1h** was obtained (1001 mg, 3.14 mmol 51% yield) as a pale-yellow solid.

**$^1$H NMR** (400 MHz, $CDCl_3$) δ 8.03 (d, *J* = 2.1 Hz, 1H), 7.84 – 7.75 (m, 3H), 7.72 – 7.63 (m, 3H), 7.53 – 7.33 (m, 5H), 2.98 – 2.86 (m, 4H), 2.08 (t, *J* = 2.6 Hz, 2H).

**$^{13}$C NMR** (101 MHz, $CDCl_3$) δ 149.2, 148.9, 141.5, 140.5, 139.8, 139.3, 129.0, 128.3, 127.4, 127.4, 127.4, 127.3, 124.0, 122.8, 120.3, 120.2, 81.1, 71.0, 50.1, 27.7.

**HRMS** (ESI/QTOF) m/z: $[M + Ag]^+$ Calcd for $C_{25}H_{18}Ag^+$ 425.0454; Found 425.0466.

**IR (ATR)**: $\tilde{\nu}$ ($cm^{-1}$) = 3287, 3057, 3030, 2902, 1599, 1482, 1468, 1453, 1421, 1264, 1019, 895, 835, 758, 737, 698, 639, 593, 565, 482, 461.

**Melting point**: 97-98 °C.

**$R_f$**: 0.82 (ethyl acetate: pentane = 1:4).

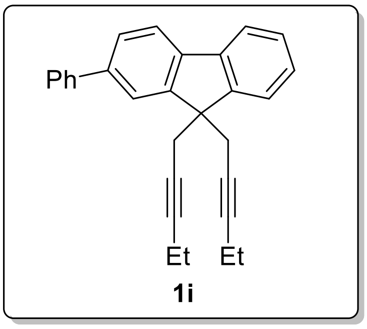


**Name**: 9,9-di(pent-2-yn-1-yl)-2-phenyl-9*H*-fluorene (**1i**).

**Synthesis**:

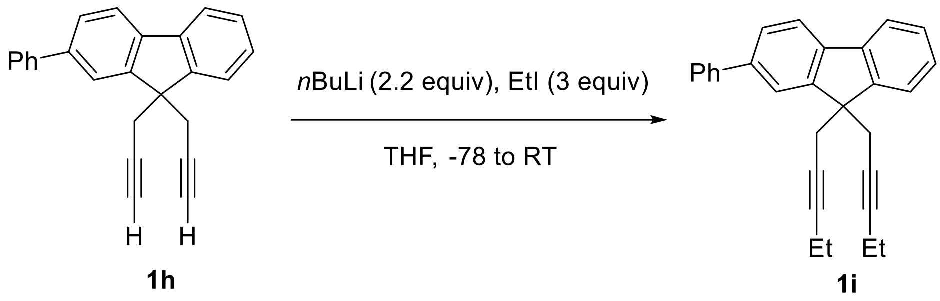


In an oven-dried Schlenk tube, dialkyne **1h** (150.0 mg, 0.471 mmol, 1 eq) and dry THF 2 mL were added under a nitrogen atmosphere. The Schlenk tube was then cooled to -78 °C, and *n*BuLi (2.5 M in hexane, 0.415 mL, 2.2 eq) was added dropwise. The reaction was stirred at -78 °C for 2 hours. Then, EtI (83.3 μL, 1.036 mmol, 2.2 equiv) was added, and the reaction mixture was allowed to slowly warm up to room temperature, stirring overnight. The reaction was quenched by adding $NH_4Cl$ (saturated water solution) at 0 °C. The resulting mixture was extracted with EtOAc (3 × 5 mL). The organic phase was dried with $Na_2SO_4$, and the solvents were evaporated. The residue was purified by flash column chromatography on silica gel, yielding the compound **1i** (156.0 mg, 0.415 mmol, 88% yield) as pale-yellow liquid.

**$^1$H NMR** (400 MHz, $CDCl_3$) δ 8.03 (d, *J* = 1.8 Hz, 1H), 7.81 – 7.72 (m, 3H), 7.68 (dd, *J* = 8.3, 1.3 Hz, 2H), 7.63 (dd, *J* = 7.9, 1.8 Hz, 1H), 7.50 – 7.43 (m, 2H), 7.43 – 7.30 (m, 3H), 2.98 – 2.70 (m, 4H), 2.18 (qt, *J* = 7.5, 2.4 Hz, 4H), 1.09 (t, *J* = 7.5 Hz, 6H).

**$^{13}$C NMR** (101 MHz, $CDCl_3$) δ 150.2, 149.8, 141.8, 140.1, 139.9, 139.5, 128.9, 127.9, 127.3, 127.3, 127.1, 126.9, 124.1, 123.1, 120.1, 119.9, 84.1, 76.6, 51.1, 27.9, 14.3, 12.6.

**HRMS** (ESI/QTOF) m/z: $[M + Ag]^+$ Calcd for $C_{29}H_{26}Ag^+$ 481.1080; Found 481.1095.

**IR (ATR)**: 3059, 3031, 2972, 2933, 2916, 2875, 2847, 1599, 1481, 1467, 1453, 1423, 1375, 1319, 1292, 1278, 1254, 1076, 1062, 1018, 895,, 833, 758, 739, 697, 642, 563, 476, 454.

**Melting point**: -

**$R_f$**: 0.43 (dichloromethane: pentane = 1:4).

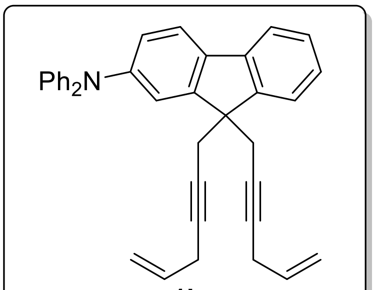


**1j**

**Name**: 9,9-di(hex-5-en-2-yn-1-yl)-*N*,*N*-diphenyl-9H-fluoren-2-amine (**1j**)
**Synthesis**:

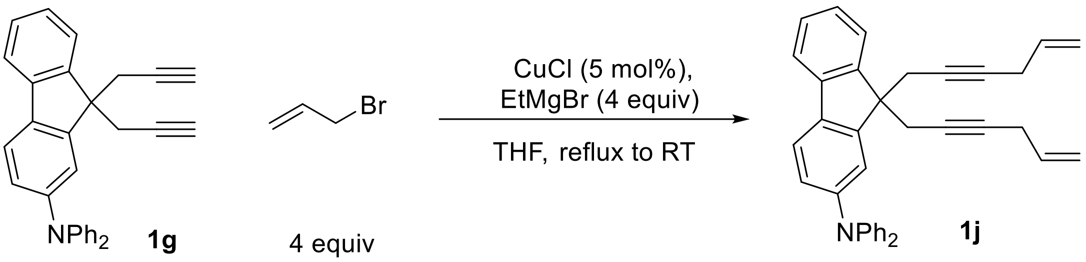


A solution of **1g** (100.0 mg, 0.244 mmol, 1 eq) in THF (2.5 mL) was added dropwise to ethylmagnesium bromide (2.5 M in $Et_2O$, 0.326 mL, 4.0 eq). After 90 minutes at reflux, CuCl (2.4 mg, 0.0122 mmol, 5 mol%) was added to the cold reaction mixture and after 15 minutes a solution of allyl bromide (118.2 mg, 0.977 mmol, 4 eq) in THF (2.5 mL) was added dropwise. After 18 hours at reflux, the cold reaction mixture was poured onto saturated $NH_4Cl$ solution at 0°C. The aqueous phase was extracted with $Et_2O$, then the organic phase was washed with $H_2O$, dried with $MgSO_4$, then concentrated. Purify the residue by column chromatography over silica gel using pentane/diethyl ether as eluent to obtain the product **1j** in around (49.1 mg, 0.1003 mmol, 41% yield) as pale-yellow liquid. (the compound is unstable and difficult to characterize, only **$H^1$ NMR** is provided) The obtained product should be immediately submitted to catalysis condition.

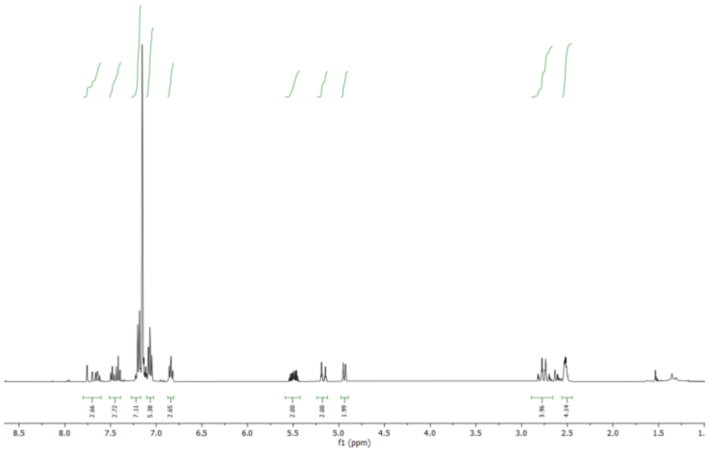

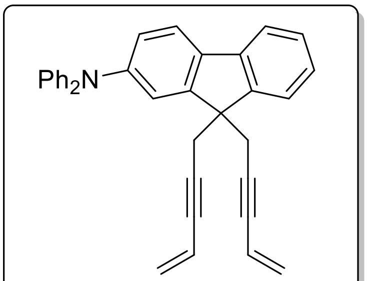

1k

**Name**: 9,9-di(pent-4-en-2-yn-1-yl)-*N*,*N*-diphenyl-9H-fluoren-2-amine (**1k**).
**Synthesis**:

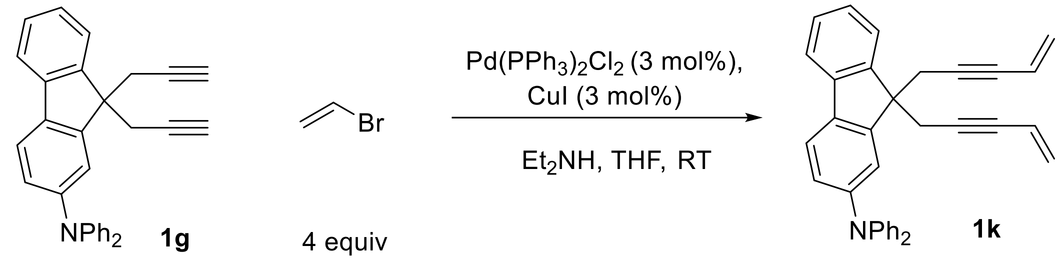


Add $Pd(PPh_3)_2Cl_2$ (5.1 mg, 7.3 µmol 3 mol %) and CuI (1.4 mg, 7.3 µmol, 3 mol %) into an oven-dried Schlenk flask, followed with dry degassed diethylamine 0.50 mL under $N_2$ atmosphere. Cool the mixture with ice bath. Add the terminal alkyne **1g** (100 mg, 0.2442 mmol, 1.0 equiv) and vinyl bromide (1.0 M in THF, 0.977 mL, 4 eq) *via* a syringe. Warm the resulting mixture up to room temperature. Monitor the complete conversion of the starting material by TLC. Wash the reaction mixture with water followed by extraction with *n*-pentane/diethyl ether (1:1). Dry the organic phase with anhydrous $Na_2SO_4$. Concentrate the resulting mixture under vacuum. Purify the residue by column chromatography over silica gel using pentane/diethyl ether as eluent to obtain the product **1k** (87.5 mg, 0.190 mmol, 78% yield) as a white foam.
**$^1$H NMR** (400 MHz, $C_6D_6$) δ 7.75 (d, *J* = 2.1 Hz, 1H), 7.65 (d, *J* = 7.4 Hz, 1H), 7.47 (d, *J* = 7.4 Hz, 1H), 7.41 (d, *J* = 8.1 Hz, 1H), 7.25 – 7.17 (m, 5H), 7.15 – 7.04 (m, 6H), 6.85 (t, *J* = 7.3 Hz, 2H), 5.47 (ddt, *J* = 17.5, 10.9, 2.1 Hz, 2H), 5.31 (dd, *J* = 17.6, 2.4 Hz, 2H), 5.01 (dd, *J* = 10.9, 2.4 Hz, 2H), 2.94 – 2.64 (m, 4H).
**$^{13}$C NMR** (101 MHz, $C_6D_6$) δ 150.8, 149.2, 148.6, 147.8, 140.4, 135.6, 129.7, 126.9, 126.1, 124.6, 124.6, 124.1, 123.0, 121.0, 120.6, 119.7, 117.7, 88.1, 82.1, 51.4, 28.7.

**HRMS** (ESI/QTOF) m/z: $[M + H]^+$ Calcd for $C_{35}H_{28}N^+$ 462.2216; Found 462.2206.

**IR (ATR)**: $\tilde{\nu}$ ($cm^{-1}$) = 3058, 3035, 3001, 2869, 2205, 1620, 1588, 1555, 1482, 1468, 1452, 1437, 1410, 1336, 1312, 1279, 1242, 1212, 1157, 1124, 1104, 1073, 1027, 973, 906, 783, 754, 731, 696, 517.

**Melting point**: -

**$R_f$**: 0.68 (ethyl acetate: pentane = 1:10).

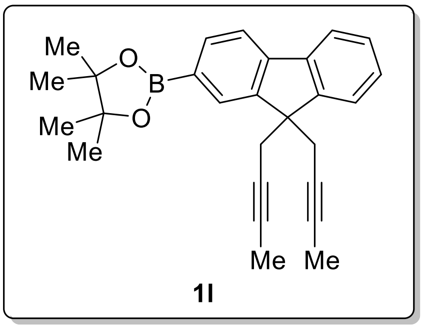


**Name**: 2-(9,9-di(but-2-yn-1-yl)-9*H*-fluoren-2-yl)-4,4,5,5-tetramethyl-1,3,2-dioxaborolane (**1l**).

**Synthesis**:

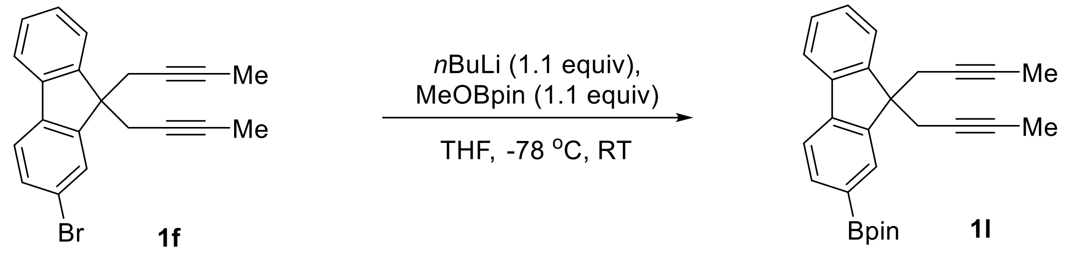


In a flame-dried Schlenk-flask, compound **1f** (1673 mg, 4.79 mmol, 1 eq) was dissolved in 23 mL dry THF at rt. Reaction was cooled to -78 °C (dry ice/Acetone). *n*BuLi (2.5 M in *n*-Hexane, 2.11 mL 1.1 eq) was added dropwise. After stirring at -78 °C for 50 mins MeOBpin (832 mg, 5.27 mmol, 1.1 eq) was added dropwise. Ice bad was removed and the reaction was allowed to warm up to rt. The reaction was quenched after TLC showed full conversion with sat. aq. $NH_4Cl$. The phases were separated and the aqueous phase was extracted with DCM. The combined organic phases were dried over $MgSO_4$, filtered and concentrated under reduced pressure. The resulting crude product was purified *via* flash chromatography (liquid load, $SiO_2$) to afford the functionalized product **1l (**1360 mg, 3.4 mmol, 71%) as a white solid.

**$^1$H NMR** (400 MHz, $CDCl_3$) δ 8.16 (s, 1H), 7.83 (d, J = 1.1 Hz, 1H), 7.79 – 7.68 (m, 3H), 7.42 – 7.29 (m, 2H), 2.86 – 2.66 (m, 4H), 1.79 (t, J = 2.5 Hz, 6H), 1.37 (s, 12H).

**$^{11}$B NMR** (128 MHz, $CDCl_3$) δ 30.4.

**$^{13}$C NMR** (101 MHz, $CDCl_3$) δ 150.0, 148.4, 143.0, 139.9, 134.5, 130.4, 127.7, 127.6, 124.2, 120.3, 119.2, 83.8, 78.0, 76.3, 51.1, 27.8, 25.1, 3.6.

**HRMS** (APPI/LTQ-Orbitrap) m/z: $[M + H]^+$ Calcd for $C_{27}H_{30}BO_2^+$ 397.2333; Found 397.2339.
**IR (ATR)**: $\tilde{\nu}$ ($cm^{-1}$) = 2977, 2918, 1610, 1492, 1456, 1415, 1379, 1355, 1322, 1287, 1261, 1214, 1144, 1109, 1082, 962, 866, 846, 760, 740, 702, 685, 421.
**Melting point**: 121-122 °C.
**$R_f$**: 0.79 (ethyl acetate: pentane = 1:4).

General overview for aza-borafluorene derivatives synthesis.

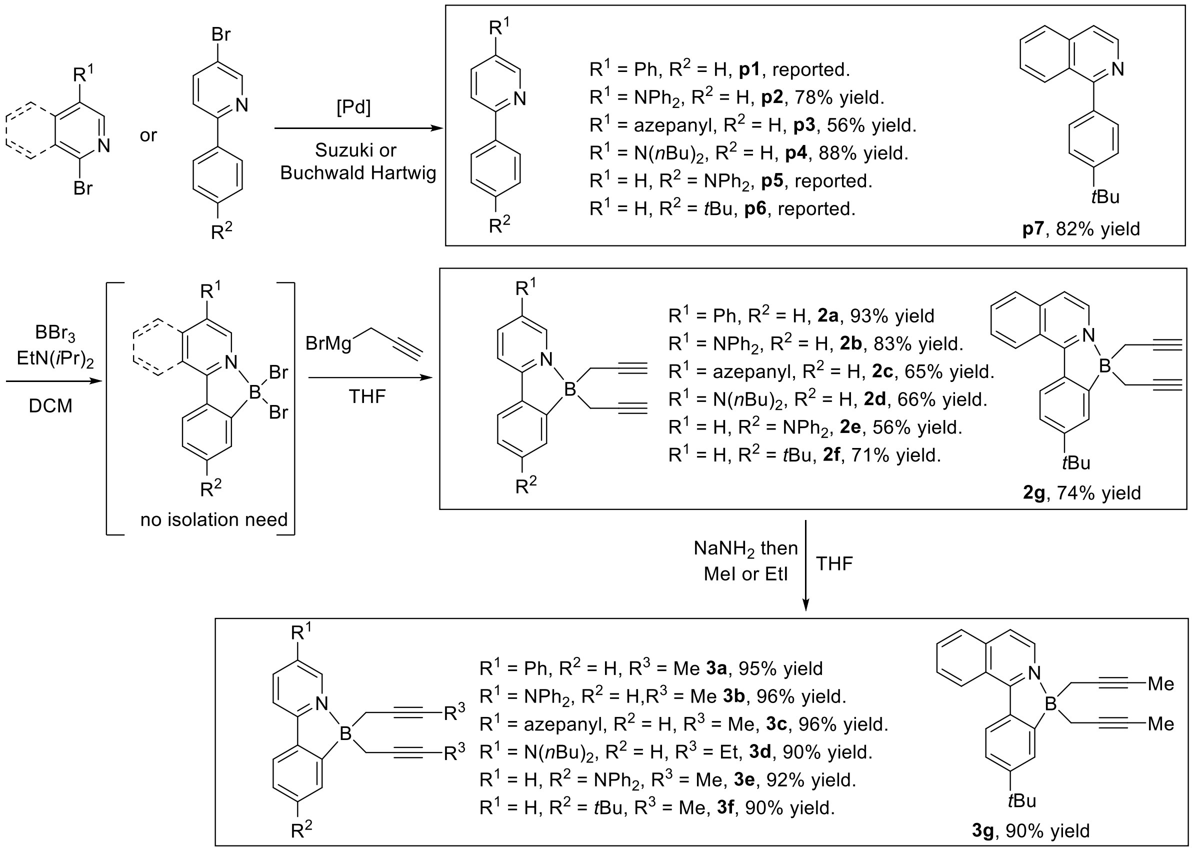


General overview *N*, *N*-chelated boron derivatives synthesis.

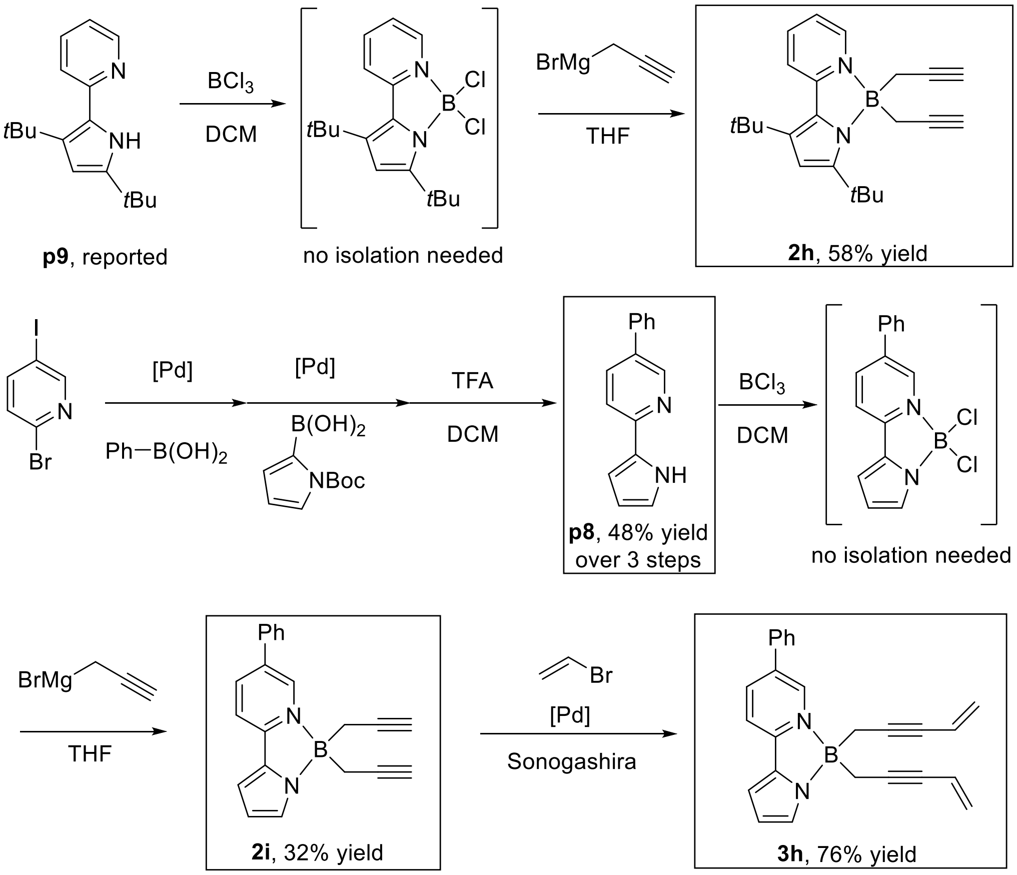

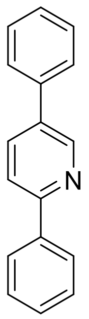

**p1**

Compound **p1** was synthesized according to literature[13].

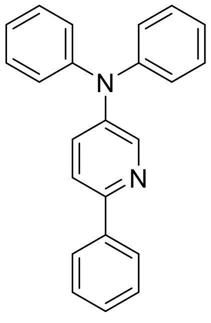

**p2**

**Name**: *N*,*N*,6-triphenylpyridin-3-amine (**p2**).

**Synthesis**:

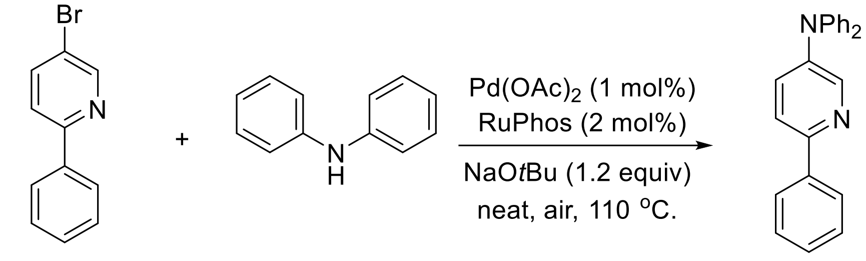


A screw-cap vial equipped with a magnetic stir bar was charged with the aryl bromide (614.5 mg, 2.625 mmol, 1.05 equiv), diphenyl amine (423.1 mg, 2.5 mmol, 1.0 eq), $Pd(OAc)_2$ (5.6 mg, 0.025 mmol, 1 mol%), RuPhos (23.3 mg, 0.05 mmol, 2 mol%), and powdered NaO*t*Bu (288.3 mg, 3 mmol, 1.2 eq). The vial was transferred to a preheated oil bath (110 °C). After 12 h, the reaction mixture was cooled and dissolved in $CH_2Cl_2/H_2O$ mixture (1:1). The organic phase was separated, the solvent was evaporated in vacuo, and the product **p2** was isolated by flash chromatography on a silica gel column in 630 mg, 3.0 mmol, 78% yield as a white solid[14].

**$^1$H NMR** (400 MHz, $CDCl_3$) δ 8.44 (dd, *J* = 2.8, 0.9 Hz, 1H), 7.99 – 7.92 (m, 2H), 7.61 (dd, *J* = 8.6, 0.9 Hz, 1H), 7.50 – 7.41 (m, 3H), 7.41 – 7.34 (m, 1H), 7.34 – 7.27 (m, 4H), 7.18 – 7.12 (m, 4H), 7.12 – 7.05 (m, 2H).

**$^{13}$C NMR** (101 MHz, $CDCl_3$) δ 150.6, 146.9, 144.7, 143.3, 130.3, 129.7, 128.8, 128.4, 126.5, 124.6, 123.9, 120.5.

**HRMS** (ESI/QTOF) m/z: $[M + H]^+$ Calcd for $C_{23}H_{19}N_2^+$ 323.1543; Found 323.1552.

**IR (ATR)**: $\tilde{\nu}$ ($cm^{-1}$) = 3059, 3033, 2924, 1583, 1555, 1485, 1473, 1444, 1392, 1333, 1315, 1282, 1226, 1175, 1155 1074, 1024, 837, 778, 753, 737, 694, 644, 629, 511.

**Melting point**: 133-134 °C.

**$R_f$**: 0.82 (ethyl acetate: pentane = 1:4).

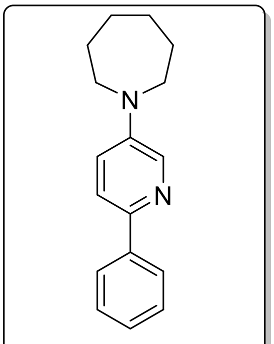

**p3**

**Name**: 1-(6-phenylpyridin-3-yl)azepane (**p3**).

**Synthesis**:

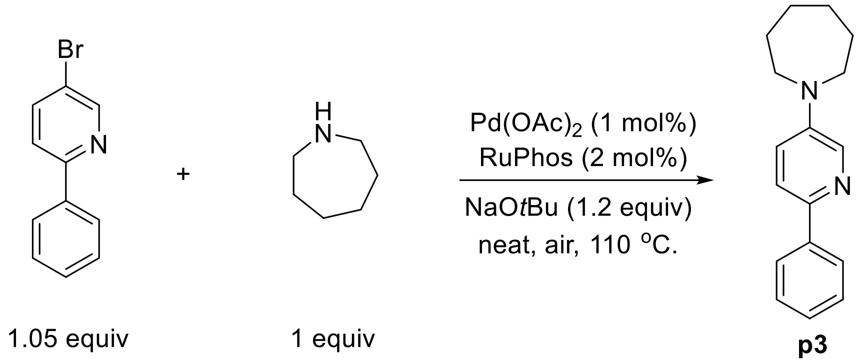


A screw-cap vial equipped with a magnetic stir bar was charged with the aryl bromide (614.5 mg, 2.625 mmol, 1.05 eq), azepane (247.9 mg, 2.5 mmol, 1eq), $Pd(OAc)_2$ (5.6 mg, 0.025 mmol, 1 mol%), RuPhos (23.3 mg, 0.05 mmol, 2 mol%), and powdered NaO*t*Bu (288.3 mg, 3 mmol, 1.2 eq). The vial was transferred to a preheated oil bath (110 °C). After 12 h, the reaction mixture was cooled and dissolved in $CH_2Cl_2/H_2O$ mixture (1:1). The organic phase was separated, the solvent was evaporated in vacuo, and the product **p3** was isolated by flash chromatography on a silica gel column in 356 mg, 1.411 mmol, 56% yield as a pale-yellow oil.

**$^1$H NMR** (400 MHz, $CDCl_3$) δ 8.21 (d, *J* = 3.1 Hz, 1H), 7.96 – 7.87 (m, 2H), 7.58 (d, *J* = 8.9 Hz, 1H), 7.46 – 7.38 (m, 2H), 7.35 – 7.27 (m, 1H), 7.03 (dd, *J* = 8.9, 3.1 Hz, 1H), 3.60 – 3.44 (m, 4H), 1.90 – 1.78 (m, 4H), 1.64 – 1.51 (m, 4H).

**$^{13}$C NMR** (101 MHz, $CDCl_3$) δ 144.3, 143.8, 139.6, 133.5, 128.7, 127.4, 125.8, 120.8, 118.4, 49.2, 27.5, 27.1.

**HRMS** (ESI/QTOF) m/z: $[M + H]^+$ Calcd for $C_{17}H_{21}N_2^+$ 253.1699; Found 253.1711.

**IR (ATR)**: $\tilde{\nu}$ ($cm^{-1}$) = 2924, 2852, 1586, 1553, 1501, 1486, 1446, 1405, 1368, 1355, 1269, 1246, 1230, 1200, 1184, 1000, 904, 823, 776, 734, 695.

**Appearance**: pale yellow liquid.

**Melting point**: -

**$R_f$**: 0.53 (ethyl acetate: pentane = 1:4).

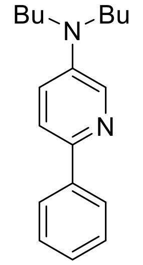

**p4**

**Name**: 1-(6-phenylpyridin-3-yl)azepane (**p4**).

**Synthesis**:

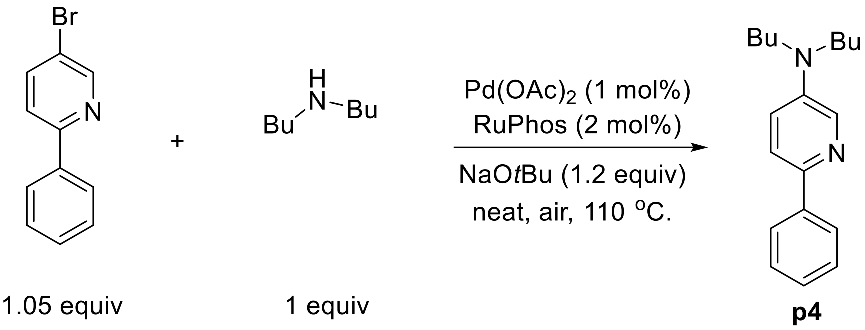


A screw-cap vial equipped with a magnetic stir bar was charged with the aryl bromide (1229 mg, 5.25 mmol, 1.05 eq), HN(*n*-Bu)$_2$ (646.2 mg, 5 mmol), Pd(OAc)$_2$ (11.2 mg, 0.05 mmol, 1 mol%), RuPhos (46.7 mg, 0.1 mmol, 2 mol%), and powdered NaO*t*Bu (576.6 mg, 6 mmol, 1.2 eq). The vial was transferred to a preheated oil bath (110 °C). After 12 h, the reaction mixture was cooled and dissolved in $CH_2Cl_2/H_2O$ mixture (1:1). The organic phase was separated, the solvent was evaporated in vacuo, and the product **p7** was isolated by flash chromatography on a silica gel column in 1240 mg, 4.391 mmol, 88% yield as a pale-yellow oil.
**$^1$H NMR** (400 MHz, $CDCl_3$) δ 8.16 (d, *J* = 3.1 Hz, 1H), 7.95 – 7.87 (m, 2H), 7.57 (d, *J* = 8.8 Hz, 1H), 7.42 (t, *J* = 7.7 Hz, 2H), 7.33 – 7.26 (m, 1H), 6.97 (dd, *J* = 8.9, 3.1 Hz, 1H), 3.41 – 3.23 (m, 4H), 1.69 – 1.53 (m, 4H), 1.38 (h, *J* = 7.3 Hz, 4H), 0.97 (t, *J* = 7.4 Hz, 6H).

**$^{13}$C NMR** (101 MHz, $CDCl_3$) δ 144.2, 143.0, 139.8, 134.0, 128.7, 127.2, 125.7, 120.6, 118.7, 50.7, 29.4, 20.4, 14.1.

**HRMS** (ESI/QTOF) m/z: $[M + H]^+$ Calcd for $C_{19}H_{27}N_2^+$ 283.2169; Found 283.2179.

**IR (ATR)**: $\tilde{\nu}$ ($cm^{-1}$) = 3059, 2959, 2922, 2853, 2165, 1597, 1550, 1492, 1445, 1413, 1385, 1260, 1089, 1022, 866, 799, 767, 741, 701, 634, 434, 400.

**Appearance**: pale yellow liquid.

**Melting point**: -

**$R_f$**: 0.77 (ethyl acetate: pentane = 1:4).

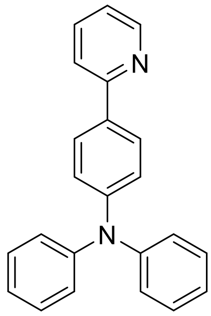


**p5**

Compound **p5** was synthesized according to literature[15].

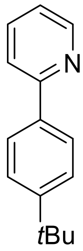


**p6**

Compound **p6** was synthesized according to literature[16].

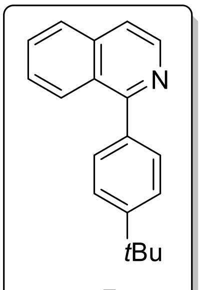


**p7**

**Name**: 1-(4-(*tert*-butyl)phenyl)isoquinoline (**p7**).

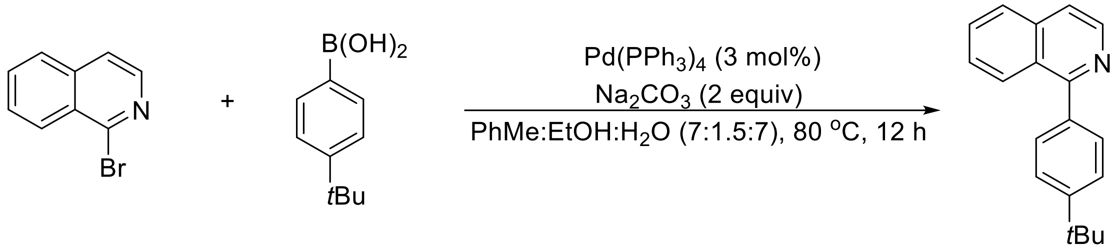


**Synthesis**: A 100 mL Schlenk flask equipped with a magnetic stir bar was charged with $Na_2CO_3$ (530 mg, 5 mmol, 2 eq), $Pd(PPh_3)_4$ (86.7 mg, 0.075 mmol, 3 mol%), and an aryl boronic acid (534 mg, 3 mmol, 1.2 eq). The flask was evacuated and backfilled with nitrogen three times. Subsequently, toluene (7 mL), ethanol (1.5 mL), $H_2O$ (7 mL), and 1-bromoisoquinoline (520 mg, 2.5 mmol) were added by syringes. The reaction mixture was refluxed for 12 h, and then cooled to room temperature. To the reaction mixture was added $H_2O$ (30 mL), extracted by ethyl acetate (2 × 30 mL), dried over $Na_2SO_4$. Then the solvent was removed under reduced pressure, and the residue was purified by flash chromatography on silica gel with hexanes/ethyl acetate as the eluent to give the known corresponding product **p7** in 538 mg, 2.058 mmol, 82% yield as colorless thick oil [17].

**$^1$H NMR** (400 MHz, $CDCl_3$) δ 8.60 (d, *J* = 5.6 Hz, 1H), 8.18 (d, *J* = 8.5 Hz, 1H), 7.88 (d, *J* = 8.3 Hz, 1H), 7.72 – 7.61 (m, 4H), 7.58 – 7.51 (m, 3H), 1.40 (s, 9H).

**$^{13}$C NMR** (101 MHz, $CDCl_3$) δ 160.9, 151.8, 142.3, 137.0, 136.7, 130.1, 129.8, 128.0, 127.2, 127.1, 126.9, 125.5, 119.8, 34.9, 31.5.

**HRMS** (ESI/QTOF) m/z: $[M + H]^+$ Calcd for $C_{19}H_{20}N^+$ 262.1590; Found 262.1597.

**IR (ATR)**: $\tilde{\nu}$ ($cm^{-1}$) = 3048, 2959, 2902, 2865, 1619, 1611, 1582, 1550, 1514, 1498, 1474, 1460, 1400, 1384, 1355, 1321, 1305, 1267, 1245, 1202, 1167, 1128, 1103, 1021, 975, 873, 822, 800, 745, 679, 664, 588, 520, 468, 434.

**Melting point**: -

**$R_f$**: 0.53 (ethyl acetate: pentane = 1:4).

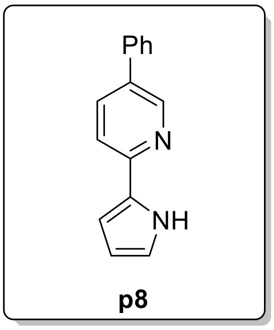


**Name**: 5-phenyl-2-(1H-pyrrol-2-yl)pyridine (**p8)**.
**Synthesis**:

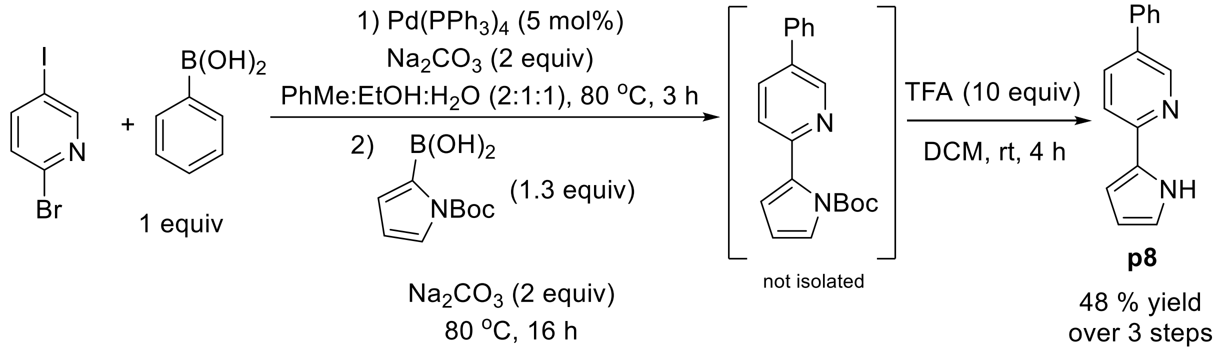


In a flame-dried flask, 2-bromo-5-iodopyridine (1.42 g, 5 mmol, 1 eq), phenylboronic acid (0.61 g, 5 mmol, 1 eq), $K_2CO_3$ (1.38 g, 10 mmol, 2 eq) and $Pd(PPh_3)_4$ (0.29 g, 0.25 mmol, 5 mol%) were added and vacuum flashed with $N_2$ (3x). Toluene: EtOH: $H_2O$ 2:1:1 (12 mL) were added. The reaction mixture was heated to 80 °C for 3 h (TLC control). Reaction was cooled to rt then [1- [(2-methylpropan-2-yl)oxycarbonyl]pyrrol-2-yl]boronic acid (1.48 g, 7 mmol, 1.4 eq) and $K_2CO_3$ (1.38 g, 10 mmol, 2 eq) were added to the reaction mixture and heated to 80 °C for 16 h. The reaction was quenched with the addition of EtOAc and $H_2O$ at rt. The phases were separated and the aqueous phase was extracted with EtOAc. The combined organic phases were dried over $MgSO_4$, filtered, and concentrated under reduced pressure. The Crude product was dissolved in 25 mL DCM and Trifluoroacetic acid (3 mL) was added and stirred at rt for 4 h. Reaction was quenched with $NaHCO_3$. The phases were separated and the aqueous phase was extracted with DCM. The combined organic phases were dried over $MgSO_4$, filtered, and concentrated under reduced pressure. The resulting oil was purified via flash chromatography (liquid load, $SiO_2$) (DCM) to afford the title compound **p8** (0.52 g, 2.4 mmol, 48%) as a pale-yellow solid. The data match with reported literature[18].

**$^1$H NMR** (400 MHz, $CDCl_3$) δ 9.87 (s, 1H), 8.72 (dd, *J* = 2.4, 0.9 Hz, 1H), 7.85 (dd, *J* = 8.3, 2.3 Hz, 1H), 7.61 (t, *J* = 8.4 Hz, 3H), 7.48 (t, *J* = 7.6 Hz, 2H), 7.39 (t, *J* = 7.3 Hz, 1H), 6.94 (td, *J* = 2.6, 1.3 Hz, 1H), 6.77 (ddd, *J* = 3.7, 2.4, 1.4 Hz, 1H), 6.33 (dt, *J* = 3.7, 2.6 Hz, 1H).

**$^{13}$C NMR** (101 MHz, $CDCl_3$) δ 149.6, 147.3, 138.0, 135.1, 133.4, 131.5, 129.2, 127.9, 126.9, 120.2, 118.2, 110.5, 107.5.

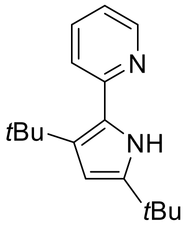

**p9**

Compound **p9** was synthesized according to literature.[19]

**General procedure B:**

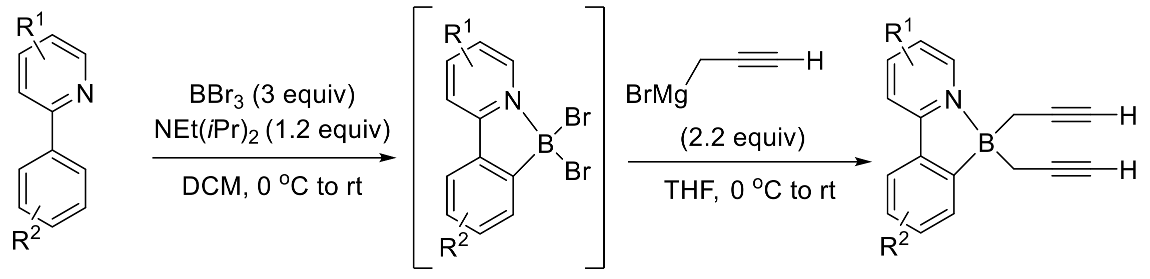


**p1-7** **2a-g**

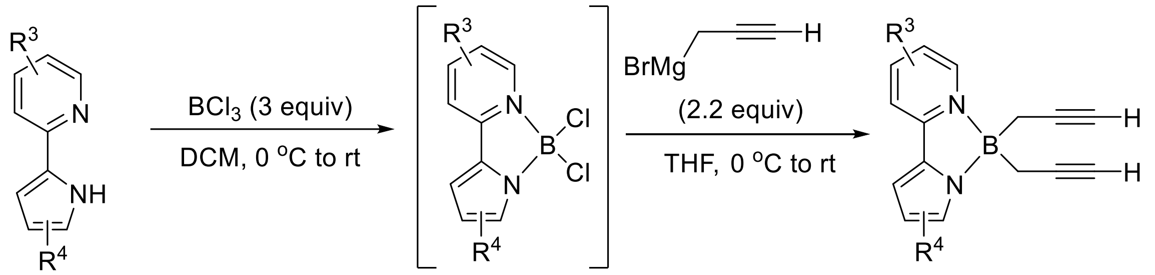


**p8, p9** **2h, 2i**

**Step 1**: To a stirred solution of 2-phenyl-pyridine derivatives and $i$Pr$_2$NEt (1.2 equiv) in $CH_2Cl_2$ (1.0 M) at 0 °C was added $BBr_3$ (3.0 equiv, 1.0 M in $CH_2Cl_2$). After being stirred at room temperature for 24 h, saturated $K_2CO_3$ aqueous solution was added to the reaction mixture. The organic layer was separated and extracted with $CH_2Cl_2$, washed with water, brine, dried over anhydrous $Na_2SO_4$. The solvent was removed under vacuum and the resulting solid was collected by filtration and washed with hexane to give crude dibromo-boron intermediate, which was used directly without further purification[20].

To a solution of **p8** or **p9** in toluene (C = 0.25 M) was added $BCl_3$ (3.0 equiv, 1.0 M in $CH_2Cl_2$) dropwise over 10 min at 0 °C under $N_2$ atmosphere. After being stirred at room temperature for 24 h, saturated $Na_2CO_3$ aqueous solution was added to the reaction mixture. The organic layer was separated and extracted with $CH_2Cl_2$, washed with water, brine, and dried over anhydrous $Na_2SO_4$. The solvent was removed under vacuum and the resulting solid was collected by filtration and washed with hexane to give the crude dichloro-boron intermediate, which was used directly without further purification[20].

**Step 2**: To a solution of crude intermediate in THF (0.2 M) was added fresh prepared propargyl magnesium bromide (2.2 equiv, in THF, around 0.5 M, note: the concentration should be titrated) dropwise over 10 min at 0 °C under $N_2$ atmosphere. After being stirred for 30 min, the mixture was warmed to ambient temperature and stirred for 2 h. The reaction mixture was quenched with saturated $NH_4Cl$ aqueous solution and extracted with $CH_2Cl_2$, the combined organic layer was washed with brine and dried over anhydrous $Na_2SO_4$. The solvent was removed under vacuum and the residue was purified by flash chromatography on silica gel to afford compound **2a-2i**[20].

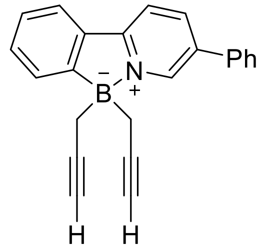

**2a**

**Name**: 3-phenyl-6,6-di(prop-2-yn-1-yl)-6*H*-5λ$^4$,6λ$^4$-benzo[3,4][1,2]azaborolo[1,5-*a*]pyridine (**2a**).

**Synthesis**: Following General procedure B, (**Step 1**: using **p1** 516.6 mg, 2.23 mmol; $BBr_3$ 1 M in DCM, 6.7 mL 6.69 mmol; *i*$Pr_2NEt$ 346.4 mg, 2.68 mmol. **Step** 2: using 9.8 mL, 0.5 M propargyl magnesium bromide THF solution) the desired product **2a** was obtained in 663 mg, 2.077 mmol, 93% yield as a white solid.

**$^1$H NMR** (400 MHz, $CDCl_3$) δ 9.15 (d, *J* = 2.4 Hz, 1H), 8.32 – 8.22 (m, 1H), 8.04 (d, *J* = 8.4 Hz, 1H), 7.87 (d, *J* = 7.6 Hz, 1H), 7.76 (d, *J* = 7.3 Hz, 1H), 7.67 (d, *J* = 7.3 Hz, 2H), 7.55 (t, *J* = 7.4 Hz, 2H), 7.52 – 7.44 (m, 2H), 7.37 (t, *J* = 7.4 Hz, 1H), 1.84 (dd, *J* = 16.3, 2.9 Hz, 2H), 1.73 (t, *J* = 2.8 Hz, 2H), 1.62 (dd, *J* = 16.3, 2.9 Hz, 2H).

**$^{11}$B NMR** (128 MHz, $CDCl_3$) δ -0.6.

**$^{13}$C NMR** (101 MHz, $CDCl_3$) δ 156.3, 141.1, 139.1, 135.9, 135.8, 135.4, 130.9, 129.6, 129.6, 129.2, 127.1, 126.6, 121.6, 117.9, 88.4, 66.2, 12.4 – 10.9 (m).

**HRMS** (ESI/QTOF) m/z: $[M + Ag]^+$ Calcd for $C_{23}H_{18}AgBN^+$ 426.0578; Found 426.0582.
**IR (ATR)**: $\tilde{\nu}$ (cm$^{-1}$) = 3287, 3059, 2875, 2095, 1622, 1601, 1508, 1482, 1461, 1448, 1408, 1386, 1330, 1287, 1211, 1160, 1067, 1022, 996, 970, 910, 848, 758, 738, 697, 616.

**Melting point**: 162-163 $^oC$.

**$R_f$**: 0.51 (ethyl acetate: pentane = 1:4).

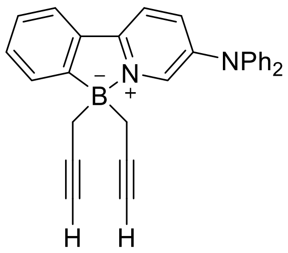

**2b**

**Name**: *N*,*N*-diphenyl-6,6-di(prop-2-yn-1-yl)-6*H*-5λ$^4$,6λ$^4$-benzo[3,4][1,2]azaborolo[1,5-*a*]pyridin-3-amine (**2b**).

**Synthesis**: Following General procedure B, (**Step 1**: using **p2** 1192.9 mg, 3.70 mmol; $BBr_3$ 1 M in DCM, 11.1 mL 11.1 mmol; *i*$Pr_2NEt$ 573.9 mg, 4.44 mmol. **Step** 2: using 14.5 mL, 0.56 M propargyl magnesium bromide THF solution) the desired product **2b** was obtained in 1259 mg, 3.068 mmol 83% yield as a yellow solid.

.

**$^1$H NMR** (400 MHz, $CDCl_3$) δ 8.58 (d, *J* = 2.1 Hz, 1H), 7.82 – 7.63 (m, 4H), 7.41 – 7.27 (m, 6H), 7.23 – 7.10 (m, 6H), 1.65 (dd, *J* = 15.8, 3.3 Hz, 2H), 1.53 – 1.39 (m, 4H).

**$^{11}$B NMR** (128 MHz, $CDCl_3$) δ -0.8.

**$^{13}$C NMR** (101 MHz, $CDCl_3$) δ 150.5, 145.8, 143.6, 136.3, 135.9, 132.4, 130.2, 129.7, 129.3, 126.4, 125.3, 125.1, 120.5, 117.8, 88.2, 66.1, 11.9 – 11.1 (m).

**HRMS** (ESI/QTOF) m/z: $[M + Ag]^+$ Calcd for $C_{29}H_{23}AgBN_2^+$ 517.1000; Found 517.1021.

**IR (ATR)**: $\tilde{\nu}$ ($cm^{-1}$) = 3303, 3057, 2875, 2096, 1619, 1589, 1557, 1489, 1447, 1408, 1342, 1329, 1288, 1270, 1236, 1212, 1174, 1157, 1075, 1061, 1028, 996, 970, 902, 842, 784, 755, 733, 697, 616, 563, 514.

**Melting point**: 119-120 °C.

**$R_f$**: 0.66 (ethyl acetate: pentane = 1:4).

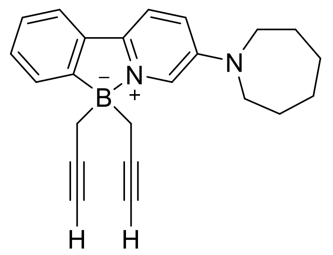

**2c**

**Name**: 3-(azepan-1-yl)-6,6-di(prop-2-yn-1-yl)-6*H*-5λ$^4$,6λ$^4$-benzo[3,4][1,2]azaborolo[1,5-*a*]pyridine (**2c**).

**Synthesis**: Following General procedure B, (**Step 1**: using **p3** 627.5 mg, 2.49 mmol; $BBr_3$ 1 M in DCM, 7.5 mL, 7.5 mmol; *i*$Pr_2$NEt 386.2 mg, 2.99 mmol. **Step 2**: using 11.5 mL, 0.48 M propargyl magnesium bromide THF solution) the desired product **2c** was obtained (550 mg, 1.62 mmol, 65% yield) as pale-yellow solid.

**$^1$H NMR** (400 MHz, $CDCl_3$) δ 8.35 (d, *J* = 2.9 Hz, 1H), 7.72 (d, *J* = 9.0 Hz, 1H), 7.69 – 7.62 (m, 2H), 7.35 – 7.24 (m, 3H), 3.60 – 3.49 (m, 4H), 1.92 – 1.82 (m, 4H), 1.80 – 1.69 (m, 4H), 1.65 – 1.50 (m, 6H).

**$^{11}$B NMR** (128 MHz, $CDCl_3$) δ -1.0.

**$^{13}$C NMR** (101 MHz, $CDCl_3$) δ 145.2, 144.3, 137.0, 129.2, 128.4, 126.4, 126.1, 122.5, 119.4, 118.1, 89.0, 65.8, 49.5, 27.3, 27.0, 12.4 – 11.2 (m).

**HRMS** (ESI/QTOF) m/z: $[M + Ag]^+$ Calcd for $C_{23}H_{25}AgBN_2^+$ 447.1156; Found 447.1166.

**IR (ATR)**: $\tilde{\nu}$ ($cm^{-1}$) = 3303, 2925, 2094, 1618, 1560, 1506, 1449, 1417, 1400, 1367, 1358, 1337, 1272, 1204, 1173, 998, 829, 780, 744, 728, 618.

**Melting point**: 98-99 °C.

**$R_f$**: 0.49 (ethyl acetate: pentane = 1:4).

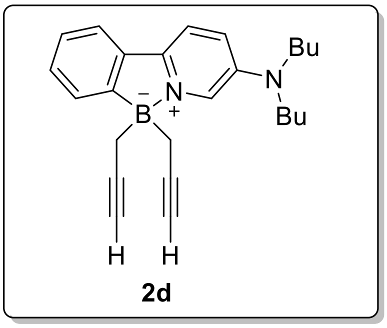

**Name**: 3-(azepan-1-yl)-6,6-di(prop-2-yn-1-yl)-6*H*-5λ$^4$,6λ$^4$-benzo[3,4][1,2]azaborolo[1,5-*a*]pyridine (**2d**).

**Synthesis**: Following General procedure B, (**Step 1**: using **p4** 899.6 mg, 3.19 mmol; $BBr_3$ 1 M in DCM, 9.6 mL, 9.6 mmol; *i*$Pr_2$NEt 494.4 mg, 3.83 mmol. **Step 2**: using 15.9 mL, 0.44 M propargyl magnesium bromide THF solution) the desired product **2d** was obtained in 780 mg, 2.106 mmol, 66% yield as pale-yellow oil.

**$^1$H NMR** (400 MHz, $CDCl_3$) δ 8.28 (d, *J* = 2.8 Hz, 1H), 7.78 – 7.55 (m, 3H), 7.36 – 7.19 (m, 3H), 3.41 – 3.27 (m, 4H), 1.83 – 1.50 (m, 10H), 1.40 (h, *J* = 7.4 Hz, 4H), 0.99 (t, *J* = 7.4 Hz, 6H).

**$^{11}$B NMR** (128 MHz, $CDCl_3$) δ -0.85.

**$^{13}$C NMR** (101 MHz, $CDCl_3$) δ 145.0, 143.5, 137.0, 129.2, 128.4, 126.5, 126.2, 122.6, 119.4, 118.0, 89.1, 65.8, 51.1, 29.2, 20.4, 14.1, 12.3 – 11.3 (m).

**HRMS** (ESI/QTOF) m/z: $[M + Na]^+$ Calcd for $C_{25}H_{31}BN_2Na^+$ 393.2473; Found 393.2475.

**IR (ATR)**: $\tilde{\nu}$ ($cm^{-1}$) = 3311, 2956, 2929, 2896, 2871, 2095, 1618, 1559, 1506, 1449, 1418, 1406, 1367, 1337, 1286, 1261, 1225, 1183, 1101, 1064, 1036, 995, 969, 938, 899, 866, 828, 780, 760, 741, 728, 612, 589, 465.

**Melting point**: -.

**$R_f$**: 0.71 (ethyl acetate: pentane = 1:4).

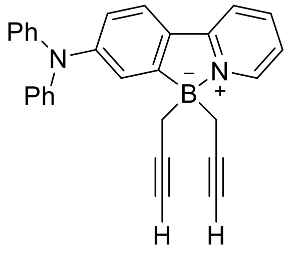

**2e**

**Name**: *N*,*N*-diphenyl-6,6-di(prop-2-yn-1-yl)-6*H*-5λ$^4$,6λ$^4$-benzo[3,4][1,2]azaborolo[1,5-*a*]pyridin-8-amine (**2e**).

**Synthesis**: Following General procedure B, (**Step 1**: using **p5** 1622.6 mg, 5.04 mmol; $BBr_3$ 1 M in DCM, 15.1 mL, 15.1 mmol; *i*$Pr_2NEt$ 780.7 mg, 3.83 mmol. **Step 2**: using 14.2 mL, 0.78 M propargyl magnesium bromide THF solution) the desired product **2e** was obtained in 1158 mg, 2.822 mmol, 56% yield as a yellow solid.

**$^1$H NMR** (400 MHz, $CDCl_3$) δ 8.75 (d, *J* = 5.8 Hz, 1H), 7.95 (td, *J* = 7.7, 1.6 Hz, 1H), 7.81 (d, *J* = 8.3 Hz, 1H), 7.64 (d, *J* = 8.4 Hz, 1H), 7.40 (d, *J* = 2.3 Hz, 1H), 7.32 – 7.25 (m, 5H), 7.19 (d, *J* = 7.3 Hz, 4H), 7.07 (t, *J* = 7.3 Hz, 2H), 6.95 (dd, *J* = 8.5, 2.3 Hz, 1H), 1.66 (dd, *J* = 16.1, 2.9 Hz, 2H), 1.61 (t, *J* = 2.9 Hz, 2H), 1.51 (dd, *J* = 16.1, 2.9 Hz, 2H).

**$^{11}$B NMR** (128 MHz, $CDCl_3$) δ -1.2.

**$^{13}$C NMR** (101 MHz, $CDCl_3$) δ 163.6 – 162.0 (m), 157.6, 150.3, 147.5, 142.7, 140.4, 129.6, 129.4, 125.6, 123.7, 122.5, 122.2, 120.4, 120.2, 117.2, 88.3, 66.0, 16.3 – 5.9 (m).

**HRMS** (ESI/QTOF) m/z: $[M + Ag]^+$ Calcd for $C_{29}H_{23}AgBN_2^+$ 517.1000; Found 517.1017.

**IR (ATR)**: $\tilde{\nu}$ ($cm^{-1}$) = 3303, 3056, 3035, 2874, 2095, 1621, 1590, 1556, 1483, 1470, 1452, 1438, 1337, 1312, 1281, 1244, 1212, 1158, 1125, 1106, 1074, 1026, 995, 879, 823, 783, 756, 732, 698, 618, 517, 476, 429, 413.

**Melting point**: 185-186 °C.

**$R_f$**: 0.38 (ethyl acetate: pentane = 1:4).

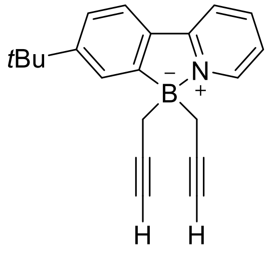

**2f**

**Name**: 8-(*tert*-butyl)-6,6-di(prop-2-yn-1-yl)-6*H*-5λ$^4$,6λ$^4$-benzo[3,4][1,2]azaborolo[1,5-*a*]pyridine (**2f**).

**Synthesis**: Following General procedure B, (**Step 1**: using **p6** 422.6 mg, 2 mmol; $BBr_3$ 1 M in DCM, 6 mL, 6 mmol; *i*$Pr_2NEt$ 489.2 mg, 2.4 mmol. **Step 2**: using 7.9 mL, 0.56 M propargyl magnesium bromide THF solution) the desired product **2f** was obtained in 425 mg, 1.42 mmol, 71% yield as a white solid.

**$^1$H NMR** (400 MHz, $CDCl_3$) δ 8.85 (d, *J* = 5.8 Hz, 1H), 8.01 (t, *J* = 7.8 Hz, 1H), 7.93 (d, *J* = 8.1 Hz, 1H), 7.77 (d, *J* = 7.6 Hz, 2H), 7.46 – 7.33 (m, 2H), 1.82 – 1.67 (m, 4H), 1.57 (dd, *J* = 16.0, 3.1 Hz, 2H), 1.39 (s, 9H).

**$^{11}$B NMR** (128 MHz, $CDCl_3$) δ -1.0.

**$^{13}$C NMR** (101 MHz, $CDCl_3$) δ 157.9, 154.2, 142.9, 140.6, 133.6, 126.4, 124.0, 121.2, 117.7, 88.5, 66.0, 35.3, 31.6, 12.1 – 10.9 (m).

**HRMS** (ESI/QTOF) m/z: $[M + Ag]^+$ Calcd for $C_{21}H_{22}AgBN^+$ 406.0891; Found 406.0884.

**IR (ATR)**: $\tilde{\nu}$ ($cm^{-1}$) = 3284, 2961, 2900, 2869, 2095, 1621, 1604, 1476, 1439, 1405, 1362, 1331, 1266, 1210, 1160, 994, 791, 768, 736, 616.

**Appearance**: white solid.

**Melting point**: 107-108 $^oC$.

**$R_f$**: 0.46 (ethyl acetate: pentane = 1:4)

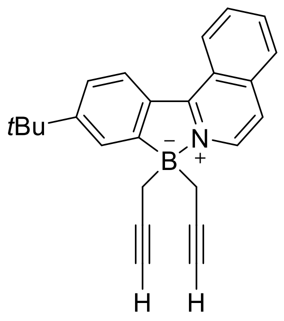

**2g**

**Name**: 8-(*tert*-butyl)-6,6-di(prop-2-yn-1-yl)-6*H*-5λ$^4$,6λ$^4$-benzo[3,4][1,2]azaborolo[1,5-*a*]pyridine (**2g**).

**Synthesis**: Following General procedure B, (**Step 1**: using **p7** 151.4 mg, 0.58 mmol; $BBr_3$ 1 M in DCM, 1.7 mL, 1.7 mmol; *i*$Pr_2NEt$ 142.7 mg, 0.7 mmol. **Step 2**: using 2.5 mL, 0.51 M propargyl magnesium bromide THF solution) the desired product **2g** was obtained in 150 mg, 0.43 mmol, 74% yield as a white solid.

**$^1$H NMR** (400 MHz, $CDCl_3$) δ 9.15 (d, *J* = 7.8 Hz, 1H), 8.68 (d, *J* = 6.5 Hz, 1H), 8.47 (d, *J* = 8.5 Hz, 1H), 8.03 – 7.95 (m, 1H), 7.94 – 7.79 (m, 3H), 7.71 (d, *J* = 6.4 Hz, 1H), 7.49 (dd, *J* = 8.4, 2.1 Hz, 1H), 1.85 – 1.76 (m, 2H), 1.71 – 1.60 (m, 4H), 1.43 (s, 9H).

**$^{11}$B NMR** (128 MHz, $CDCl_3$) δ -1.8.

**$^{13}$C NMR** (101 MHz, $CDCl_3$) δ 157.2, 153.9, 138.2, 135.3, 134.3, 132.5, 129.1, 128.1, 126.9, 126.3, 125.6, 125.6, 124.2, 120.2, 88.6, 66.1, 35.3, 31.5, 12.3 – 10.9 (m).

**HRMS** (ESI/QTOF) m/z: $[M + Na]^+$ Calcd for $C_{25}H_{24}BNNa^+$ 372.1894; Found 372.1877.

**IR (ATR)**: $\tilde{\nu}$ ($cm^{-1}$) = 3304, 2960, 2900, 2869, 2094, 1626, 1599, 1545, 1507, 1452, 1429, 1393, 1358, 1321, 1259, 1208, 1162, 1144, 1079, 1026, 990, 898, 869, 826, 798, 740, 703, 683, 663, 613, 592, 564, 459.

**Melting point**: 122-123 $^o$C.

**$R_f$**: 0.50 (ethyl acetate: pentane = 1:4)

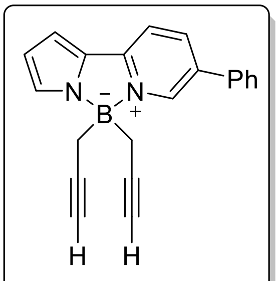

2h

**Name**: 8-phenyl-5,5-di(prop-2-yn-1-yl)-5H-5λ$^4$,6λ$^4$-pyrrolo[1',2':3,4][1,3,2]diazaborolo[1,5-a]pyridine (**2h**).

**Synthesis**: Following General procedure B, (**Step 1**: using **p7** 481.8 mg, 2.19 mmol; $BCl_3$ 1 M in DCM, 6.6 mL, 6.6 mmol. **Step 2**: using 9.6 mL, 0.50 M propargyl magnesium bromide THF solution) the desired product **2h** was obtained (226 mg, 0.7 mmol, 32%) as an orange solid.

**$^1$H NMR** (400 MHz, $CDCl_3$) δ 8.77 (s, 1H), 8.10 (d, *J* = 8.6 Hz, 1H), 7.61 (dd, *J* = 12.3, 8.4 Hz, 3H), 7.55 – 7.49 (m, 2H), 7.46 (d, *J* = 7.3 Hz, 1H), 7.17 (d, *J* = 2.1 Hz, 1H), 6.78 (d, *J* = 4.6 Hz, 1H), 6.47 – 6.43 (m, 1H), 1.85 – 1.75 (m, 4H), 1.65 (dd, *J* = 16.2, 3.0 Hz, 2H).

**$^{11}$B NMR** (128 MHz, $CDCl_3$) δ 1.65.

**$^{13}$C NMR** (101 MHz, $CDCl_3$) δ 147.8, 139.6, 139.3, 135.8, 132.2, 131.2, 129.6, 128.9, 126.7, 125.0, 116.4, 114.6, 106.9, 86.4, 66.9, 14.0 – 10.1 (m).

**HRMS** (ESI/QTOF) m/z: $[M + Na]^+$ Calcd for $C_{21}H_{17}BN_2Na^+$ 331.1377; Found 331.1374.

**IR (ATR)**: $\tilde{\nu}$ ($cm^{-1}$) = 3283, 3060, 3031, 2902, 2877, 2097, 1631, 1572, 1538, 1467, 1448, 1395, 1335, 1299, 1287, 1259, 1220, 1164, 1070, 1048, 1028, 1010, 957, 908, 840, 764, 735, 695, 607, 549, 519, 483.

**Appearance**: orange solid.

**Melting point**: 156-157 °C.

**$R_f$**: 0.57 (ethyl acetate: pentane = 1:4).

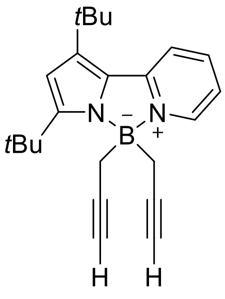

**2i**

**Name**: 1,3-di-tert-butyl-5,5-di(prop-2-yn-1-yl)-5H-5λ[4],6λ[4]-pyrrolo[1',2':3,4][1,3,2]diazaborolo[1,5-a]pyridine (**2i**).

**Synthesis**: Following General procedure B, (**Step 1**: using **p8** 1103.0 mg, 4.30 mmol; $BCl_3$ 1 M in DCM, 12.9 mL, 12.9 mmol. **Step 2**: using 18.9 mL, 0.50 M propargyl magnesium bromide THF solution) the desired product **2i** was obtained (859 mg, 2.495 mmol, 58% yield) as a yellow solid.

**$^1$H NMR** (400 MHz, $CDCl_3$) δ 8.47 (d, *J* = 6.0 Hz, 1H), 7.85 – 7.69 (m, 2H), 7.04 – 6.92 (m, 1H), 6.16 (s, 1H), 1.98 (dd, *J* = 17.1, 2.9 Hz, 2H), 1.70 (dd, *J* = 17.1, 2.8 Hz, 2H), 1.55 (t, *J* = 2.8 Hz, 2H), 1.42 (s, 9H), 1.39 (s, 9H).

**$^{11}$B NMR** (128 MHz, $CDCl_3$) δ 1.33.

**$^{13}$C NMR** (101 MHz, $CDCl_3$) δ 149.6, 148.5, 140.6, 139.8, 136.8, 128.4, 117.6, 116.0, 111.4, 86.6, 66.7, 33.4, 32.0, 31.3, 30.8, 15.2 – 11.5 (m).

**HRMS** (ESI/QTOF) m/z: $[M + Na]^+$ Calcd for $C_{23}H_{29}BN_2Na^+$ 367.2316; Found 367.2326.

**IR (ATR)**: $\tilde{\nu}$ ($cm^{-1}$) = 3309, 3280, 2956, 2926, 2870, 2100, 1622, 1548, 1519, 1488, 1471, 1408, 1390, 1361, 1340, 1286, 1262, 1244, 1203, 1158, 1087, 1048, 1023, 993, 963, 925, 876, 834, 807, 766, 746, 725, 712, 697, 670, 616, 545, 480.

**Melting point**: 149-150 °C.

**$R_f$**: 0.76 (ethyl acetate: pentane = 1:4).

**General procedure C:**

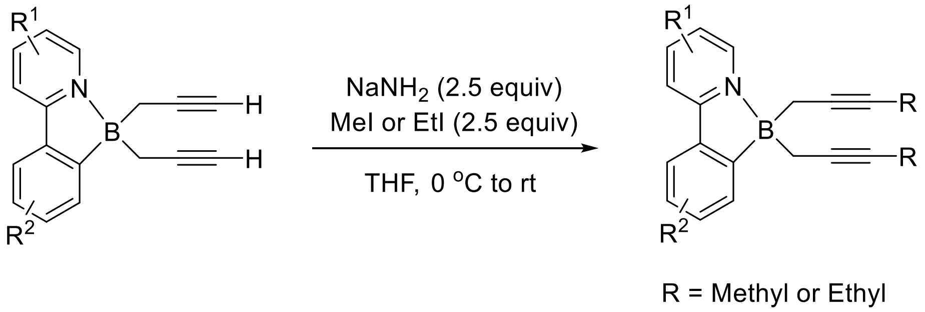


**2a-g** **3a-g**

Within the $N_2$-filled glovebox, compound **2a-g** was combined with 2.5 equivalents of sodium amide in a microwave tube. Subsequently, the tube was sealed and brought outside the glovebox. THF (0.25 M) was introduced at 0°C under a $N_2$ atmosphere. Following a 10-minute stirring period, MeI or EtI (2.5 equivalents) was added to the reaction mixture. The mixture was then allowed to warm to ambient temperature and stirred overnight. The reaction mixture was quenched with saturated $NH_4Cl$ aqueous solution and extracted with $CH_2Cl_2$, the combined organic layer was washed with brine, and dried over anhydrous $Na_2SO_4$. The solvent was removed under vacuum and the residue was purified by flash chromatography on silica gel to afford compound **3a-g**.

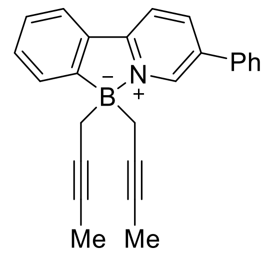

**3a**

**Name**: 6,6-di(but-2-yn-1-yl)-3-phenyl-6*H*-5λ[4],6λ[4]-benzo[3,4][1,2]azaborolo[1,5-*a*]pyridine (**3a**).

**Synthesis**: Following General procedure C, (using **2a** 100 mg 0.313 mmol) the desired product **3a** was obtained in 103.2 mg, 0.297 mmol, 95% yield as a white solid.

**$^{1}$H NMR** (400 MHz, $CDCl_3$) δ 9.19 (d, *J* = 2.3 Hz, 1H), 8.29 – 8.20 (m, 1H), 8.01 (d, *J* = 8.5 Hz, 1H), 7.85 (d, *J* = 7.8 Hz, 1H), 7.73 (d, *J* = 7.3 Hz, 1H), 7.71 – 7.64 (m, 2H), 7.54 (t, *J* = 7.4 Hz, 2H), 7.51 – 7.42 (m, 2H), 7.34 (t, *J* = 6.9 Hz, 1H), 1.83 – 1.64 (m, 8H), 1.50 (dq, *J* = 13.6, 3.3 Hz, 2H).

**$^{11}$B NMR** (128 MHz, $CDCl_3$) δ -0.6.

**$^{13}$C NMR** (101 MHz, $CDCl_3$) δ 156.1, 141.5, 138.6, 136.2, 135.8, 135.0, 130.6, 129.6, 129.6, 129.1, 127.0, 126.2, 121.5, 117.6, 82.5, 73.3, 12.5 – 11.6 (m), 3.9.

**HRMS** (ESI/QTOF) m/z: $[M + Ag]^+$ Calcd for $C_{25}H_{22}AgBN^+$ 454.0891; Found 454.0904.

**IR (ATR)**: $\tilde{\nu}$ ($cm^{-1}$) = 3298, 3058, 2875, 2095, 1623, 1601, 1508, 1483, 1461, 1448, 1408, 1386, 1330, 1310, 1287, 1212, 1159, 1067, 1022, 996, 970, 942, 911, 848, 793, 758, 738, 697, 622, 541, 468, 432, 402.

**Melting point**: 129-130 $^{o}$C

**$R_f$**: 0.53 (ethyl acetate: pentane = 1:4).

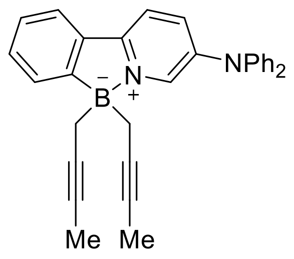


**3b**

**Name**: 6,6-di(but-2-yn-1-yl)-*N*,*N*-diphenyl-6*H*-5λ$^4$,6λ$^4$-benzo[3,4][1,2]azaborolo[1,5-*a*]pyridin-3-amine (**3b**).

**Synthesis**: Following General procedure C, (using **2b** 100 mg, 0.244 mmol) the desired product **3b** was obtained in 103.0 mg, 0.235 mmol, 96% yield as a yellow foam.

**$^1$H NMR** (400 MHz, $CDCl_3$) δ 8.66 (d, *J* = 2.4 Hz, 1H), 7.79 – 7.60 (m, 4H), 7.42 – 7.27 (m, 6H), 7.22 – 7.12 (m, 6H), 1.80 – 1.43 (m, 8H), 1.37 (dq, *J* = 15.8, 2.6 Hz, 2H).

**$^{11}$B NMR** (128 MHz, $CDCl_3$) δ 0.7.

**$^{13}$C NMR** (101 MHz, $CDCl_3$) δ 150.6, 146.1, 143.2, 136.7, 136.3, 132.6, 130.1, 129.5, 129.3, 126.1, 125.0, 124.9, 120.4, 117.6, 82.3, 73.0, 12.3 – 11.4 (m), 3.6.

**HRMS** (ESI/QTOF) m/z: $[M + Ag]^+$ Calcd for $C_{31}H_{27}AgBN_2^+$ 545.1313; Found 545.1328.

**IR (ATR)**: $\tilde{\nu}$ ($cm^{-1}$) = 3058, 2913, 2871, 1588, 1557, 1487, 1447, 1409, 1341, 1328, 1287, 1271, 1217, 1174, 1156, 1076, 1059, 1028, 1001, 947, 909, 842, 754, 731, 697, 513.

**Melting point**: -

**$R_f$**: 0.76 (ethyl acetate: pentane = 1:4).

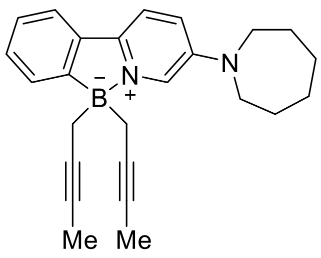


**3c**

**Name**: 3-(azepan-1-yl)-6,6-di(but-2-yn-1-yl)-6*H*-5λ[4],6λ[4]-benzo[3,4][1,2]azaborolo[1,5-a]pyridine (**3c**).

**Synthesis**: Following General procedure C, (using **2c**, 100 mg, 0.294 mmol) the desired product **3c** was obtained in 104.1 mg, 0.282 mmol, 96% yield as a pale-yellow oil.

**$^1$H NMR** (400 MHz, $CDCl_3$) δ 8.36 (d, *J* = 2.9 Hz, 1H), 7.66 (d, *J* = 9.0 Hz, 1H), 7.63 – 7.55 (m, 2H), 7.30 – 7.17 (m, 3H), 3.55 – 3.45 (m, 4H), 1.88 – 1.79 (m, 4H), 1.71 (t, *J* = 2.8 Hz, 6H), 1.65 – 1.54 (m, 6H), 1.39 (dq, *J* = 16.0, 2.8 Hz, 2H).

**$^{11}$B NMR** (128 MHz, $CDCl_3$) δ -1.2.

$^{13}$C NMR (101 MHz, $CDCl_3$) δ 145.1, 144.0, 137.0, 129.2, 128.3, 126.8, 125.8, 122.1, 119.3, 117.8, 82.9, 72.8, 49.5, 27.3, 27.0, 13.0 – 11.5 (m), 4.02.

**HRMS** (ESI/QTOF) m/z: $[M + Ag]^+$ Calcd for $C_{25}H_{29}AgBN_2^+$ 475.1469; Found 475.1469.

**IR (ATR)**: $\tilde{\nu}$ ($cm^{-1}$) = 2914, 2855, 1617, 1560, 1504, 1448, 1415, 1398, 1367, 1357, 1336, 1271, 1202, 1171, 998, 905, 863, 827, 778, 725, 647, 605, 463, 432.

**Melting point**: -

**$R_f$**: 0.51 (ethyl acetate: pentane = 1:4).

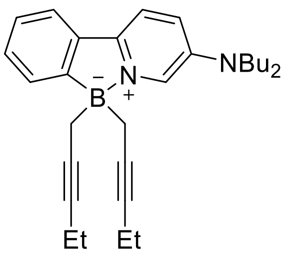


**Name:** 6,6-di(but-2-yn-1-yl)-*N*,*N*-dibutyl-6*H*-5λ$^4$,6λ$^4$-benzo[3,4][1,2]azaborolo[1,5-*a*]pyridin-8-amine (**3d**).

**Synthesis**: Following General procedure C, (using **2d**, 100 mg, 0.270 mmol) the desired product **3d** was obtained (104.0 mg, 0.244 mmol, 90% yield) as a colorless oil.

**$^1$H NMR** (400 MHz, $CDCl_3$) δ 8.20 (d, *J* = 2.9 Hz, 1H), 7.72 – 7.59 (m, 3H), 7.35 – 7.19 (m, 3H), 3.41 – 3.31 (m, 4H), 2.07 (qt, *J* = 7.5, 2.6 Hz, 4H), 1.71 – 1.57 (m, 6H), 1.52 – 1.35 (m, 6H), 1.04 – 0.94 (m, 12H).

**$^{11}$B NMR** (128 MHz, $CDCl_3$) δ 0.2.

**$^{13}$C NMR** (101 MHz, $CDCl_3$) δ 145.3, 143.4, 137.2, 129.2, 128.3, 126.9, 125.8, 122.5, 119.2, 117.7, 83.1, 79.2, 50.9, 29.2, 20.4, 15.1, 14.1, 12.9, 12.8 – 11.5 (m).

**HRMS** (ESI/QTOF) m/z: $[M + H]^+$ Calcd for $C_{29}H_{40}BN_2^+$ 427.3279; Found 427.3288.

**IR (ATR)**: $\tilde{\nu}$ ($cm^{-1}$) = 3056, 2958, 2930, 2871, 2795, 2216, 1618, 1559, 1505, 1449, 1417, 1368, 1336, 1318, 1285, 1261, 1225, 1182, 1155, 1100, 1062, 1036, 986, 961, 931, 897, 866, 828, 779, 763, 731, 695, 564, 497, 462, 450, 430.

**Melting point**: -

**$R_f$**: 0.77 (ethyl acetate: pentane = 1:4).

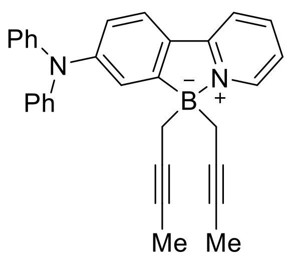

**3e**

**Name:** 6,6-di(but-2-yn-1-yl)-*N*,*N*-diphenyl-6*H*-5$\lambda^4$,6$\lambda^4$-benzo[3,4][1,2]azaborolo[1,5-*a*]pyridin-8-amine (**3e**).

**Synthesis**: Following General procedure C, (using **2e**, 100 mg, 0.244 mmol) the desired product **3e** was obtained (98.1 mg, 0.224 mmol, 92% yield) as an orange-yellow foam.

**$^1$H NMR** (400 MHz, $CDCl_3$) δ 8.69 (d, *J* = 5.6 Hz, 1H), 7.92 (td, *J* = 8.0, 1.6 Hz, 1H), 7.79 (d, *J* = 8.1 Hz, 1H), 7.63 (d, *J* = 8.4 Hz, 1H), 7.41 (d, *J* = 2.4 Hz, 1H), 7.29 (d, *J* = 8.4 Hz, 5H), 7.18 (d, *J* = 7.4 Hz, 4H), 7.06 (t, *J* = 7.3 Hz, 2H), 6.95 (dd, *J* = 8.4, 2.3 Hz, 1H), 1.60 (t, *J* = 2.8 Hz, 6H), 1.53 (dq, *J* = 15.8, 2.8 Hz, 2H), 1.41 (dq, *J* = 15.9, 2.8 Hz, 2H).

**$^{11}$B NMR** (128 MHz, $CDCl_3$) δ -1.1.

**$^{13}$C NMR** (101 MHz, $CDCl_3$) δ 157.3, 150.1, 147.7, 142.7, 140.0, 129.6, 129.4, 125.5, 123.5, 122.5, 122.3, 120.2, 119.9, 117.0, 82.4, 73.1, 12.6 – 11.2 (m), 3.7.

**HRMS** (TOF) m/z: $[M + Ag]^+$ Calcd for $C_{31}H_{27}AgBN_2^+$ 545.1313; Found 545.1316.
**IR (ATR)**: $\tilde{\nu}$ ($cm^{-1}$) = 3057, 3035, 2913, 2870, 1620, 1590, 1555, 1483, 1468, 1452, 1437, 1378, 1336, 1312, 1280, 1243, 1217, 1158, 1074, 1026, 999, 882, 782, 756, 736, 698, 519.

**Melting point**: -

**$R_f$**: 0.39 (ethyl acetate: pentane = 1:4).

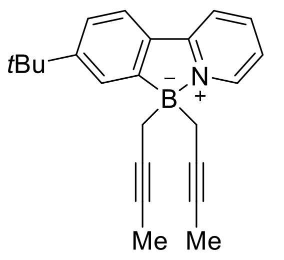


**3f**

**Name**: 6,6-di(but-2-yn-1-yl)-8-(*tert*-butyl)-6*H*-5λ$^4$,6λ$^4$-benzo[3,4][1,2]azaborolo[1,5-*a*]pyridine (**3f**).

**Synthesis**: Following General procedure C, (using **2f**, 100 mg, 0.334 mmol) the desired product **3f** was obtained (98.2 mg, 0.299 mmol, 90% yield) as a pale-yellow thick oil.

**$^1$H NMR** (400 MHz, $CDCl_3$) δ 8.80 (d, *J* = 5.8 Hz, 1H), 7.98 (td, *J* = 7.8, 1.5 Hz, 1H), 7.90 (d, *J* = 8.1 Hz, 1H), 7.80 (d, *J* = 2.0 Hz, 1H), 7.75 (d, *J* = 8.1 Hz, 1H), 7.41 – 7.31 (m, 2H), 1.74 (t, *J* = 2.8 Hz, 6H), 1.63 (dq, *J* = 15.9, 2.8 Hz, 2H), 1.48 (dq, *J* = 15.8, 2.7 Hz, 2H), 1.39 (s, 9H).

**$^{11}$B NMR** (128 MHz, $CDCl_3$) δ -0.7.

**$^{13}$C NMR** (101 MHz, $CDCl_3$) δ 157.6, 153.7, 142.9, 140.1, 133.5, 126.4, 123.7, 121.0, 120.9, 117.4, 82.6, 73.1, 35.3, 31.6, 12.6 – 11.0 (m), 3.9.

**HRMS** (ESI/QTOF) m/z: $[M + Ag]^+$ Calcd for $C_{23}H_{26}AgBN^+$ 434.1204; Found 434.1210.

**IR (ATR)**: $\tilde{\nu}$ ($cm^{-1}$) = 2961, 2913, 2866, 1621, 1603, 1476, 1439, 1406, 1362, 1331, 1265, 1201, 1159, 1132, 1064, 907, 828, 789, 764, 727, 686, 647, 482.

**Melting point**: -

**$R_f$**: 0.54 (ethyl acetate: pentane = 1:4).

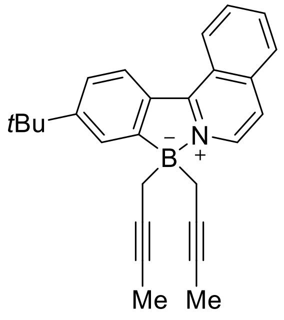

**3g**

**Name**: 8,8-di(but-2-yn-1-yl)-10-(*tert*-butyl)-8*H*-5λ$^4$,6λ$^4$-benzo[3,4][1,2]azaborolo[5,1-a]isoquinoline (**3g**)

**Synthesis**: Following General procedure C, (using **2g**, 150 mg, 0.429 mmol) the desired product **3g** was obtained (146.3 mg, 0.386 mmol, 90% yield) as pale-yellow thick oil.

**$^1$H NMR** (400 MHz, $CDCl_3$) δ 9.17 (d, *J* = 8.8 Hz, 1H), 8.71 – 8.64 (m, 1H), 8.48 (d, *J* = 8.4 Hz, 1H), 8.04 – 7.79 (m, 4H), 7.75 – 7.68 (m, 1H), 7.53 – 7.45 (m, 1H), 1.79 – 1.55 (m, 10H), 1.45 (s, 9H).

**$^{11}$B NMR** (128 MHz, $CDCl_3$) δ -0.7.

**$^{13}$C NMR** (101 MHz, $CDCl_3$) δ 156.9, 153.4, 138.0, 135.2, 134.6, 132.3, 128.9, 128.0, 126.8, 126.5, 125.7, 125.5, 123.9, 119.8, 82.7, 73.2, 35.2, 31.6, 3.9.

**HRMS** (APCI/QTOF) m/z: $[M + Na]^+$ Calcd for $C_{27}H_{28}BNNa^+$ 400.2207; Found 400.2201.

**IR (ATR)**: $\tilde{\nu}$ ($cm^{-1}$) = 3000, 2961, 2912, 2866, 1626, 1598, 1545, 1507, 1451, 1429, 1392, 1358, 1320, 1258, 1214, 1162, 1144, 1078, 1025, 989, 967, 897, 868, 826, 798, 748, 681, 663, 624, 582, 563, 458, 442.

**Appearance**: pale yellow thick oil.
**Melting point**: -.
**$R_f$**: 0.58 (ethyl acetate: pentane = 1:4)

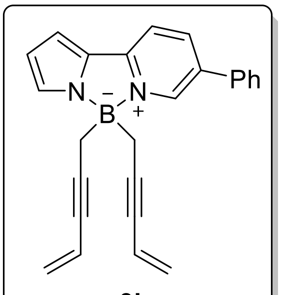


**3h**

**Name:** 5,5-di(pent-4-en-2-yn-1-yl)-8-phenyl-5H-5λ[4],6λ[4]-pyrrolo[1',2':3,4][1,3,2]diazaborolo[1,5-a]pyridine (**3h**).

**Synthesis**:

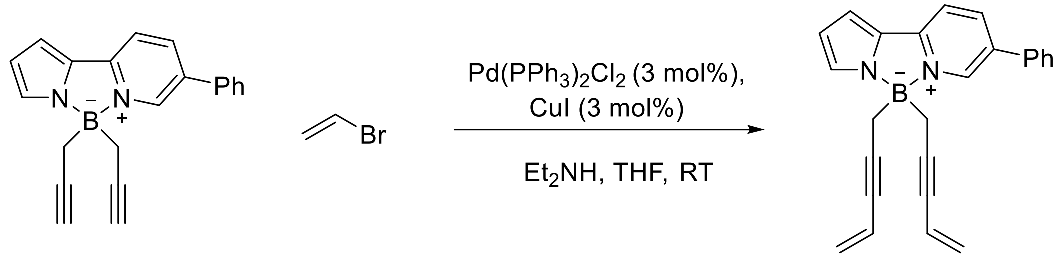


**2h** 4 equiv **3h**

Add $Pd(PPh_3)_2Cl_2$ (8.2 mg, 0.0116 mmol, 3 mol %) and CuI (2.2 mg, 0.0116 mmol, 3 mol %) into an oven-dried Schlenk flask, followed with dry degassed diethylamine (0.50 mL) under $N_2$ atmosphere. Cool the mixture with ice bath. Add the corresponding terminal alkyne (120 mg, 0.0388 mmol, 1.0 equiv) and vinyl bromide (1.0 M in THF, 1.55 mL, 4 equiv,) *via* a syringe. Warm the resulting mixture up to room temperature. Monitor the complete conversion of the starting material by TLC. Wash the reaction mixture with water followed by extraction with n-pentane/diethyl ether (1:1). Dry the organic phase with anhydrous $Na_2SO_4$. Concentrate the resulting mixture under vacuum. Purify the residue by column chromatography over silica gel using pentane/diethyl ether as eluent to obtain the product **3h** (106 mg, 0.293 mmol, 76% yield) as red oil.

**$^1$H NMR** (400 MHz, $CD_2Cl_2$) δ 8.76 (d, *J* = 1.3 Hz, 1H), 8.15 (dd, *J* = 8.5, 2.0 Hz, 1H), 7.75 – 7.59 (m, 3H), 7.57 – 7.37 (m, 3H), 7.11 (d, *J* = 1.3 Hz, 1H), 6.85 – 6.72 (m, 1H), 6.41 (dd, *J* = 3.6, 2.1 Hz, 1H), 6.04 – 5.52 (m, 2H), 5.39 – 5.22 (m, 4H), 1.87 (dd, *J* = 16.4, 2.5 Hz, 2H), 1.72 (dd, *J* = 16.4, 2.5 Hz, 2H).

**$^{11}$B NMR** (128 MHz, $CDCl_3$) δ 3.4.

**$^{13}$C NMR** (101 MHz, $CD_2Cl_2$) δ 148.0, 139.9, 139.7, 136.1, 132.5, 131.6, 129.7, 129.1, 126.9, 125.0, 124.2, 118.7, 116.6, 114.7, 107.0, 93.5, 79.0, 13.5.

**HRMS** (ESI/QTOF) m/z: $[M + H]^+$ Calcd for $C_{25}H_{22}BN_2^+$ 361.1871; Found 361.1876.

**IR (ATR)**: $\tilde{\nu}$ ($cm^{-1}$) = 3093, 3061, 3034, 3002, 2965, 2873, 2209, 1631, 1604, 1572, 1538, 1467, 1448, 1395, 1335, 1287, 1259, 1220, 1163, 1070, 1048, 1017, 974, 908, 840, 766, 754, 736, 695, 679, 607, 523, 493.

**Melting point**: -

**$R_f$**: 0.65 (ethyl acetate: pentane = 1:4).

General overview for germa & silafluorene derivatives synthesis.

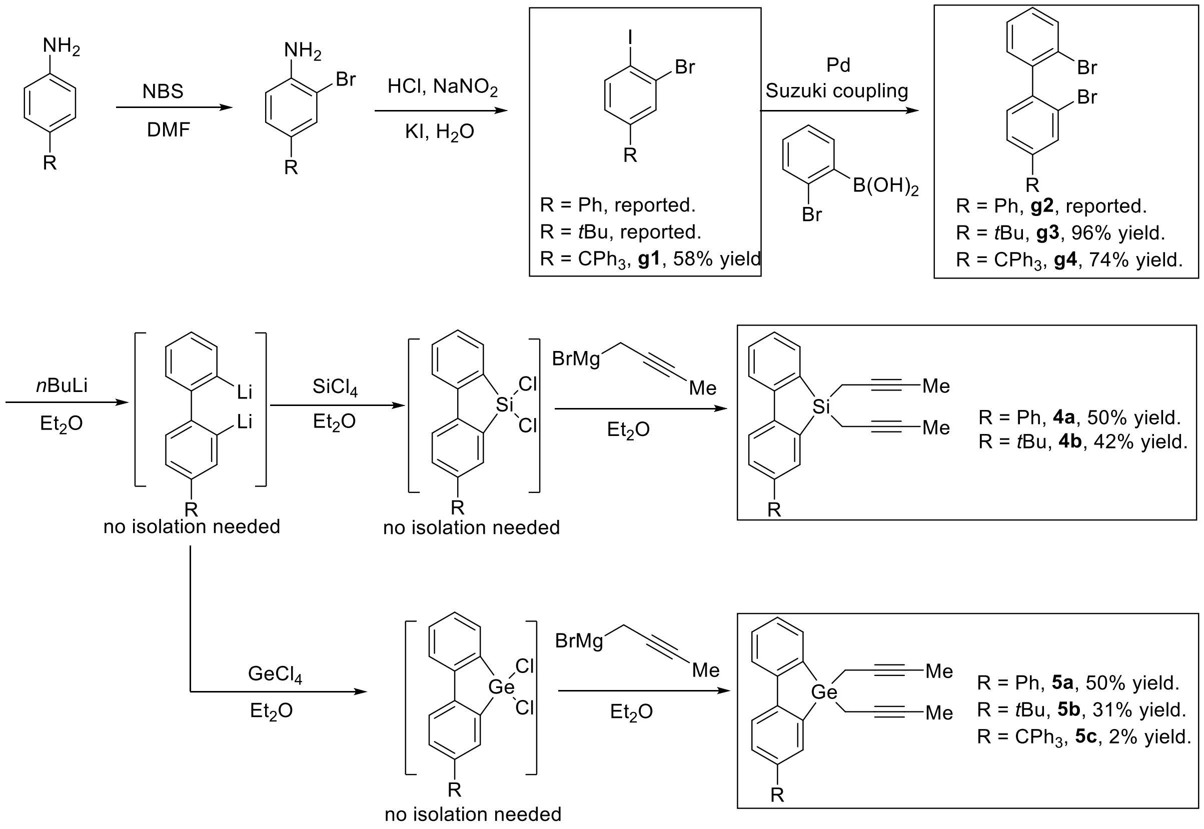


Detailed experimental procedures are shown below, and all the unknow products are characterized.

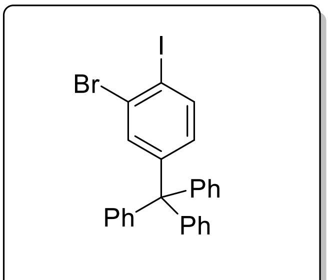

g1

**Name**: ((3-bromo-4-iodophenyl)methanetriyl)tribenzene (**g1**).
**Synthesis**:

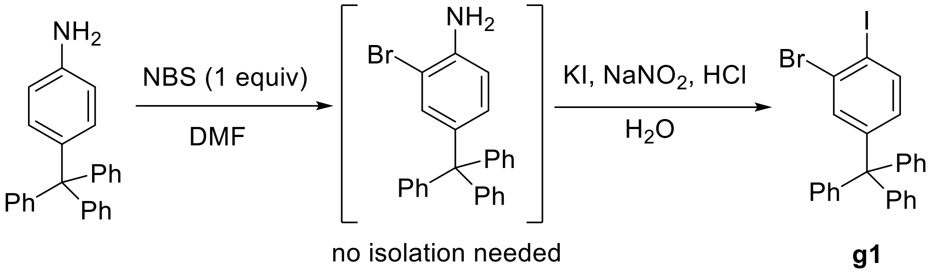



Add a solution of *N*-bromosuccinimide (10 mmol) in DMF (10 mL) in small portions at room temperature to a solution of 4-*trityl*.-butylaniline (10 mmol) in DMF (20 mL).Monitor the progress of the reaction by TLC (cyclohexane/EtOAc = 9:1). Keep for 15 min and consume all starting material. Remove all volatiles under reduced pressure (60 °C, 10 –3 mbar). Take the residue in EtOAc (100 mL) and brine (100 mL) and separate the layers. Wash the organic layer with brine (2x50 mL) and dry over $Na_2SO_4$. Filter and remove all volatiles with the aid of a rotary evaporator, the crude product was used directly without further purification.

Add 2-bromo-4-tritylaniline (1.45 g, 3.5 mmol, 1 eq) to acetone (35 mL). Add concentrated HCl (3.5 mL) in water (5 mL) to the reaction mixture until it dissolves. Cool the solution to 0 °C. Add sodium nitrite (0.400 g, 5.8 mmol) in water (2.5 mL) dropwise to the reaction mixture with stirring. Stir the solution at 0°C for another 30 minutes. Add potassium iodide (1.00 g, 6.05 mmol) in water (3.5 mL) dropwise to the solution. Stir the resulting solution at 0 °C for 1 hour then at room temperature for 1 hour and at 60 °C for 2 hours. Add sodium bisulfite (1 g) to the mixture to consume any iodine that may forms in the reaction. Extract the mixture with ether (150 mL). Wash the extract with water (60 mL), followed by brine (2 x 60 mL). Dry the extract over anhydrous $MgSO_4$. Concentrate the extract on the rotary evaporator. Purify the product by chromatography on silica gel and elution with hexane/$CH_2Cl_2$, to obtain **g1** (1058 mg, 2.014 mmol, 58% yield) as a white solid.

**$^1$H NMR** (400 MHz, $CD_2Cl_2$) δ 7.74 (d, *J* = 8.4 Hz, 1H), 7.54 (s, 1H), 7.33 – 7.17 (m, 15H), 6.90 (dt, *J* = 8.5, 2.1 Hz, 1H).

**$^{13}$C NMR** (101 MHz, $CD_2Cl_2$) δ 149.8, 146.2, 139.6, 135.2, 132.1, 131.2, 129.4, 128.2, 126.7, 98.5, 65.0.
**HRMS** (APPI/LTQ-Orbitrap) m/z: $[M]^+$ Calcd for $C_{25}H_{18}BrI^+$ 523.9631; Found 523.9626.

**IR (ATR)**: 3085, 3055, 3028, 1596, 1539, 1491, 1456, 1444, 1366, 1264, 1187, 1106, 1083, 1035, 1003, 944, 902, 879, 819, 767, 749, 724, 701, 671, 633, 528, 512, 496, 441, 406.

**Appearance**: white solid

**Melting point**: 216-217 °C.

**$R_f$**: 0.70 (dichloromethane: pentane = 1:4).

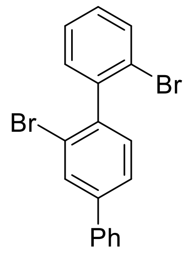


**g2**

Compound **g2** was synthesized according to literature[21].

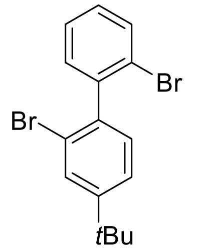


**g3**

**Name**: 2,2'-dibromo-4-(*tert*-butyl)-1,1'-biphenyl (**g3**).

**Synthesis**:

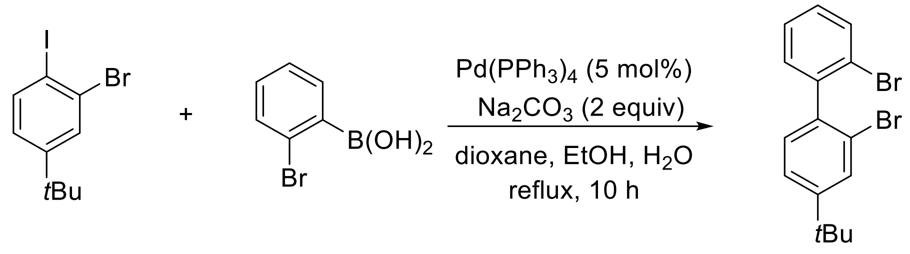


1.1 equiv **g3**

An ovendried Schlenk flask was charged with 2-bromo-4-(tert-butyl)-1-iodobenzene (1000 mg, 2.95 mmol, 1 eq) and $Pd(PPh_3)_4$ (170 mg, 0.148 mmol, 5 mol %). After being degassed and filled with $N_2$, 1,4-dioxane (10 mL), 2-bromophenyl boronic acid (652 mg, 3.24 mmol, 1.1 eq) in ethanol (1 mL) and $Na_2CO_3$ 2 equiv in water (1 mL) were added *via* syringe. The mixture was stirred at 110 °C for 10 h. Upon cooling to room temperature, 30 mL of ethyl acetate was added. The mixture was then stirred vigorously for 10 min. The organic layer was separated, washed with saturated brine, and dried over anhydrous $Na_2SO_4$. After the solvent was evaporated, the crude product was purified by silica gel chromatography to provide **g3** (1040 mg, 2.825 mmol, 96% yield) as a colorless liquid[21].

**$^1$H NMR** (400 MHz, $CDCl_3$) δ 7.70 – 7.63 (m, 2H), 7.43 – 7.33 (m, 2H), 7.29 – 7.22 (m, 2H), 7.17 (d, *J* = 8.1 Hz, 1H), 1.36 (s, 9H).

**$^{13}$C NMR** (101 MHz, $CDCl_3$) δ 153.0, 142.2, 139.2, 132.7, 131.4, 130.6, 129.7, 129.4, 127.2, 124.4, 123.9, 123.3, 34.9, 31.4.

**HRMS** (APPI/LTQ-Orbitrap) m/z: $[M]^+$ Calcd for $C_{16}H_{16}Br_2^+$ 365.9613; Found 365.9614.
**IR (ATR)**: 2962, 2903, 2868, 1604, 1541, 1460, 1434, 1423, 1382, 1363, 1258, 1115, 1079, 1046, 1027, 1000, 879, 856, 829, 782, 755, 735, 726, 704, 666, 647, 622, 562, 450.

**Melting point**: -

**$R_f$**: 0.86 (dichloromethane: pentane = 1:4).

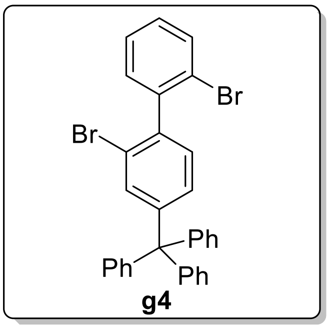


**Name**: 2,2'-dibromo-4-trityl-1,1'-biphenyl (**g4**).
**Synthesis**:

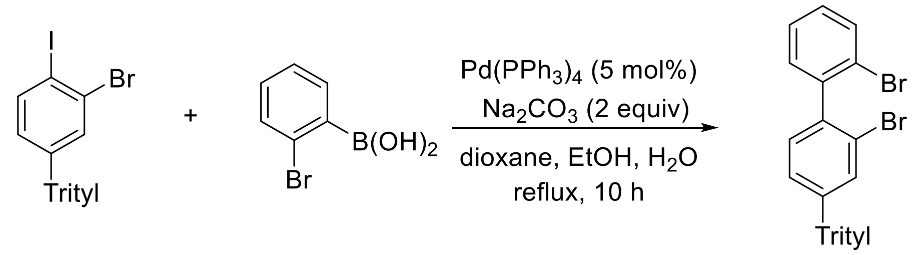


**g1** 1.1 equiv **g4**

An ovendried Schlenk flask was charged with 2-bromo-4-(trityl)-1-iodobenzene (1112 mg, 2.117 mmol, 1 eq) and $Pd(PPh_3)_4$ (122 mg, 0.106 mmol, 5 mol %). After being degassed and filled with $N_2$, 1,4-dioxane (8 mL), 2-bromophenyl boronic acid (467.7 mg, 2.33 mmol, 1.1 eq) in ethanol (0.8 mL) and $Na_2CO_3$ 2 equiv in water (0.8 mL) were added *via* syringe. The mixture was stirred at 110 °C for 10 h. Upon cooling to room temperature, 30 mL of ethyl acetate was added. The mixture was then stirred vigorously for 10 min. The organic layer was separated, washed with saturated brine, and dried over anhydrous $Na_2SO_4$. After the solvent was evaporated, the crude product was purified by silica gel chromatography to provide **g4** (868 mg, 1.57 mmol, 74% yield) as a white solid.

**$^1$H NMR** (400 MHz, $CD_2Cl_2$) δ 7.67 (d, *J* = 8.3 Hz, 1H), 7.56 (d, *J* = 2.0 Hz, 1H), 7.42 – 7.37 (m, 1H), 7.35 – 7.20 (m, 18H), 7.14 (d, *J* = 8.1 Hz, 1H).

**$^{13}$C NMR** (101 MHz, $CD_2Cl_2$) δ 149.1, 146.6, 142.2, 140.0, 134.8, 132.9, 131.5, 131.4, 130.6, 130.4, 129.8, 128.1, 127.6, 126.6, 123.9, 123.1, 65.1.

**HRMS** (nanochip-ESI/LTQ-Orbitrap) m/z: $[M]^+$ Calcd for $C_{31}H_{22}Br_2^+$ 552.0083; Found 552.0084.
**IR (ATR)**: 3085, 3056, 3029, 1597, 1538, 1491, 1460, 1445, 1374, 1188, 1158, 1081, 1047, 1030, 1001, 952, 881, 831, 782, 754, 745, 702, 673, 659, 648, 635, 511, 453.

**Melting point**: 195-196 $^o$C.

**$R_f$**: 0.62 (dichloromethane: pentane = 1:4).

**General procedure D:**

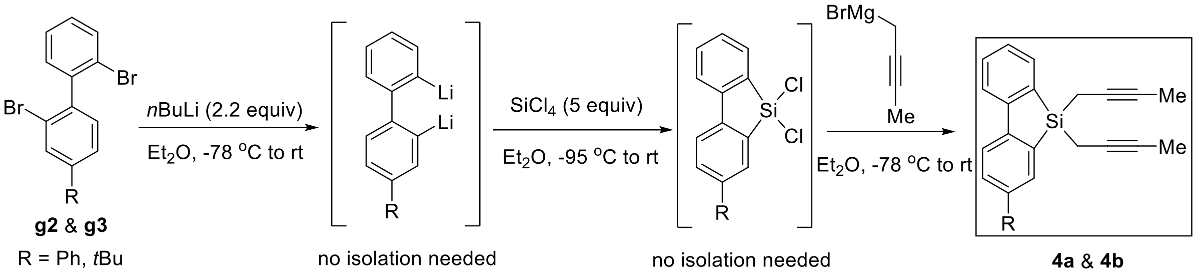


Add freshly titrated *n*BuLi (2.05 equivalents) dropwise to a stirred solution of 2,2'-dibromobiaryl derivatives in dry $Et_2O$ (0.1 M) at -78 °C. Stir the reaction mixture for one hour at -78 °C. Warm the reaction mixture. Stir the reaction mixture overnight at 0 °C. Add the solution dropwise by cannula to a stirred solution of $SiCl_4$ (5.0 equivalents) precooled to -95 °C (liquid $N_2$ and hexane) in $Et_2O$ (0.1 M) in at least 20 mins. Stir the reaction mixture for 2 hours at -78 °C (acetone and dry ice). Allow the reaction mixture to warm slowly to room temperature. Stir the reaction mixture overnight. Then the mixture was cooled to -78 °C, the volatiles are removed by Schlenk line, the evaporated solvents and $SiCl_4$ are collected in a trap in liquid nitrogen. $Et_2O$ (0.1 M) was added to the mixture, then freshly prepared but-2-yn-1-yl magnesium bromide (4 equivalents, concentration was titrated) was added dropwise. Reaction mixture was allowed to slowly warm to room temperature and stir overnight. The reaction was quenched with saturated $NH_4Cl$ aqueous solution and extracted with $CH_2Cl_2$, the combined organic layer was washed with brine and dried over anhydrous $Na_2SO_4$. The solvent was removed under vacuum and the residue was purified by flash chromatography on silica gel to afford compound **4a** & **4b**.

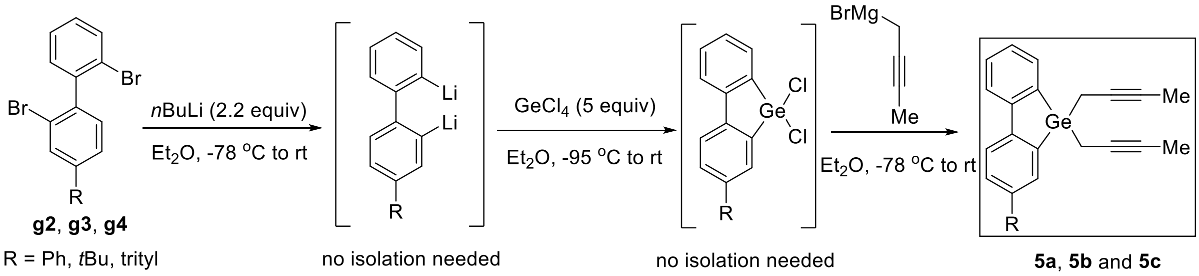


Add freshly titrated *n*BuLi (2.05 equivalents) dropwise to a stirred solution of 2,2'-dibromobiaryl derivatives in dry $Et_2O$ (0.1 M) at -78 °C. Stir the reaction mixture for one hour at -78 °C. Warm the reaction mixture. Stir the reaction mixture overnight at 0 °C. Add the solution dropwise by cannula to a stirred solution of $GeCl_4$ (5.0 equivalents) precooled to -95 °C (liquid $N_2$ and hexane) in $Et_2O$ (0.1 M) in at least 20 mins. Stir the reaction mixture for 2 hours at -78 °C (acetone and dry ice). Allow the reaction mixture to warm slowly to room temperature. Stir the reaction mixture overnight. Then the mixture was cooled to -78 °C, the volatiles are removed by Schlenk line, the evaporated solvents and $GeCl_4$ are collected in a trap in liquid nitrogen. $Et_2O$ (0.1 M) was added to the mixture, then freshly prepared but-2-yn-1-yl magnesium bromide (4 equivalents, concentration was titrated) was added dropwise. Reaction mixture was allowed to slowly warm to room temperature and stir overnight. The reaction was quenched with saturated $NH_4Cl$ aqueous solution and extracted with $CH_2Cl_2$, the combined organic layer was washed with brine and dried over anhydrous $Na_2SO_4$. The solvent was

removed under vacuum and the residue was purified by flash chromatography on silica gel to afford compound **4a**, **4b**, **5a**, **5b** & **5c**.

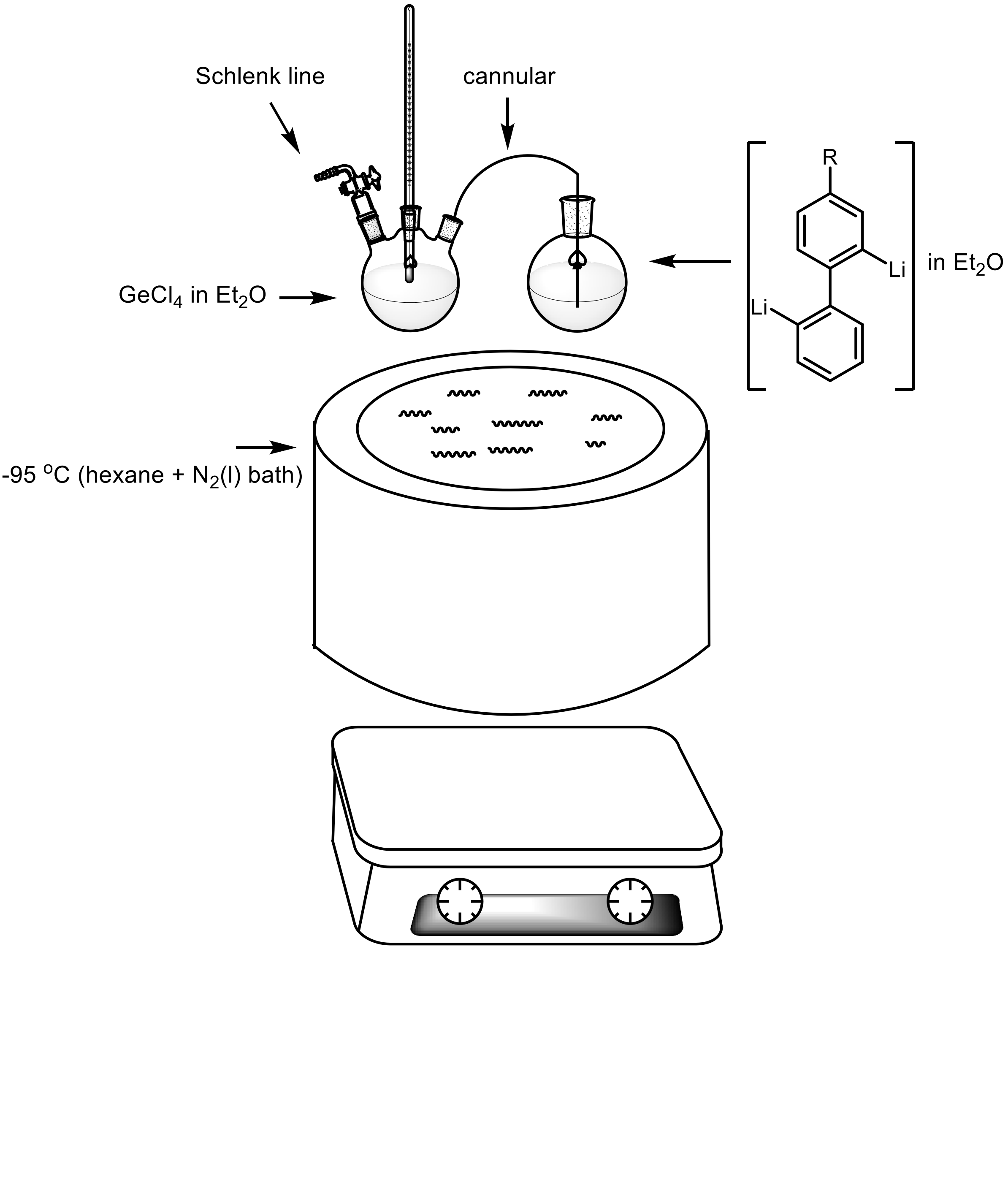

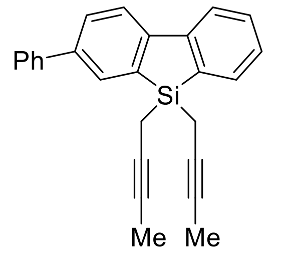

**4a**

**Name**: 5,5-di(but-2-yn-1-yl)-3-phenyl-5*H*-dibenzo[*b*,*d*]silole (**4a**).

**Synthesis**: Following General procedure D, (**Step 1**: using **g2** 1800 mg, 4.64 mmol; *n*BuLi 2.5 M in hexane, 3.8 mL, 9.51 mmol; $SiCl_4$ 3940 mg, 23.2 mmol. **Step 2**: but-2-yn-1-yl magnesium bromide 1.2 M in $Et_2O$, 15.5 mL, 18.6 mmol) the desired product **4a** was obtained 836 mg, 2.306 mmol, 50% yield as pale-yellow oil.

**$^1$H NMR** (400 MHz, $CDCl_3$) δ 8.00 (d, *J* = 2.0 Hz, 1H), 7.87 (dd, *J* = 14.4, 7.9 Hz, 2H), 7.77 (d, *J* = 7.0 Hz, 1H), 7.71 (dd, *J* = 8.0, 2.0 Hz, 1H), 7.68 – 7.63 (m, 2H), 7.52 – 7.44 (m, 3H), 7.39 – 7.28 (m, 2H), 2.00 (q, *J* = 2.8 Hz, 4H), 1.86 (t, *J* = 2.8 Hz, 6H).

**$^{13}$C NMR** (101 MHz, $CDCl_3$) δ 148.0, 147.4, 141.2, 140.3, 135.5, 135.0, 134.0, 132.7, 131.2, 130.1, 129.0, 127.6, 127.4, 127.1, 121.4, 121.2, 74.4, 3.9, 2.1.

**$^{29}$Si NMR** (79 MHz, $CDCl_3$) δ -6.73.

**HRMS** (ESI/QTOF) m/z: $[M + Ag]^+$ Calcd for $C_{26}H_{22}AgSi^+$ 469.0536; Found 469.0546.

**IR (ATR)**: $\tilde{\nu}$ ($cm^{-1}$) = 3045, 2915, 2886, 2854, 1598, 1496, 1464, 1435, 1392, 1276, 1251, 1170, 1146, 1129, 1074, 1064, 1023, 839, 823, 781, 756, 736, 697, 533, 486, 452, 435, 410.

**Melting point**: -

**$R_f$**: 0.34 (dichloromethane: pentane = 1:4).

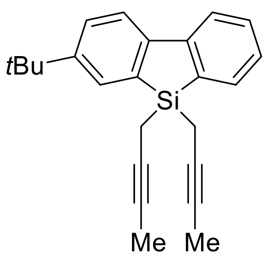

**4b**

**Name**: 5,5-di(but-2-yn-1-yl)-3-(*tert*-butyl)-5*H*-dibenzo[*b*,*d*]silole (**4b**).

**Synthesis**: Following General procedure D, (**Step 1**: using **g3** 1046 mg, 2.84 mmol; *n*BuLi 2.5 M in hexane, 2.3 mL, 5.83 mmol; $SiCl_4$ 2414 mg, 14.21 mmol. **Step 2**: but-2-yn-1-yl magnesium bromide 1.2 M in $Et_2O$, 9.5 mL, 11.4 mmol) the desired product **4b** was obtained in 410.0 mg, 1.20 mmol,42% yield as a white solid.

**$^1$H NMR** (400 MHz, $CDCl_3$) δ 7.85 – 7.71 (m, 4H), 7.51 (dd, *J* = 8.3, 2.1 Hz, 1H), 7.45 (td, *J* = 7.6, 1.4 Hz, 1H), 7.30 – 7.22 (m, 1H), 1.97 (q, *J* = 2.7 Hz, 4H), 1.88 (t, *J* = 2.8 Hz, 6H), 1.38 (s, 9H).

**$^{13}$C NMR** (101 MHz, $CDCl_3$) δ 150.3, 148.3, 145.7, 134.9, 134.5, 133.8, 131.1, 130.8, 128.4, 127.2, 120.8, 120.6, 75.7, 74.7, 34.8, 31.5, 3.9, 2.2.

**$^{29}$Si NMR** (79 MHz, $CDCl_3$) δ -7.30.

**HRMS** (ESI/QTOF) m/z: $[M + Ag]^+$ Calcd for $C_{24}H_{26}AgSi^+$ 449.0849; Found 449.0855.

**IR (ATR)**: $\tilde{\nu}$ ($cm^{-1}$) = 3047, 2961, 2916, 2864, 1590, 1475, 1460, 1435, 1392, 1362, 1255, 1164, 1128, 1116, 1075, 1061, 867, 834, 795, 774, 753, 736, 703, 648, 485, 455, 404.

**Appearance**: white solid.

**Melting point**: 104-105 °C.

**$R_f$**: 0.44 (dichloromethane: pentane = 1:4).

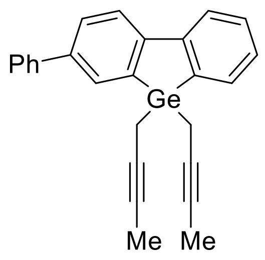


**5a**

**Name**: 5,5-di(but-2-yn-1-yl)-3-phenyl-5*H*-dibenzo[*b*,*d*]germole (**5a**).

**Synthesis**: Following General procedure D, (**Step 1**: using **g2** 1010 mg, 2.60 mmol; *n*BuLi 2.5 M in hexane, 2.1 mL, 5.33 mmol; $GeCl_4$ 2791 mg, 13.01 mmol. **Step 2**: but-2-yn-1-yl magnesium bromide 1.2 M in $Et_2O$, 8.7 mL, 10.4 mmol) the desired product **5a** was obtained in 527 mg, 1.30 mmol, 50% yield as a pale-yellow oil.

**$^1$H NMR** (400 MHz, $CDCl_3$) δ 8.00 – 7.86 (m, 3H), 7.76 – 7.63 (m, 4H), 7.51 – 7.43 (m, 3H), 7.39 – 7.30 (m, 2H), 2.17 (q, *J* = 2.8 Hz, 4H), 1.84 (t, *J* = 2.8 Hz, 6H).

**$^{13}$C NMR** (101 MHz, $CDCl_3$) δ 146.2, 145.6, 141.2, 140.5, 138.0, 137.5, 133.8, 132.5, 130.4, 129.2, 128.9, 127.8, 127.4, 127.1, 121.9, 121.8, 75.8, 75.2, 3.9, 2.8.

**HRMS** (ESI/QTOF) m/z: $[M + Ag]^+$ Calcd for $C_{26}H_{22}AgGe^+$ 514.9979; Found 514.9989.

**IR (ATR)**: $\tilde{\nu}$ ($cm^{-1}$) = 3059, 2951, 2914, 2853, 1598, 1464, 1441, 1435, 1395, 1275, 1162, 1144, 1122, 1021, 838, 779, 757, 729, 718, 697, 532, 426.

**Melting point**: -

**$R_f$**: 0.25 (dichloromethane: pentane = 1:4).

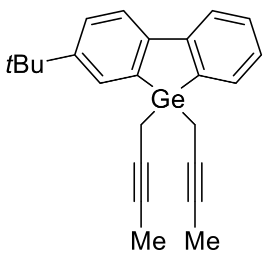

**5b**

**Name**: 5,5-di(but-2-yn-1-yl)-3-(*tert*-butyl)-5*H*-dibenzo[*b*,*d*]germole (**5b**).

**Synthesis**: Following General procedure D, (**Step 1**: using **g3** 1010 mg, 2.77 mmol; *n*BuLi 2.5 M in hexane, 2.3 mL, 5.62 mmol; $GeCl_4$ 2942 mg, 13.7 mmol. **Step 2**: but-2-yn-1-yl magnesium bromide 1.2 M in $Et_2O$, 9.2 mL, 11.0 mmol) the desired product **5b** was obtained in 328 mg, 0.85 mmol, 31% yield as a white foam.

**$^1$H NMR** (400 MHz, $CD_2Cl_2$) δ 7.96 – 7.73 (m, 3H), 7.69 (d, *J* = 8.4 Hz, 1H), 7.53 – 7.40 (m, 2H), 7.28 (t, *J* = 7.3 Hz, 1H), 2.20 – 2.04 (m, 4H), 1.90 – 1.74 (m, 6H), 1.37 (s, 9H).

$^{13}$C NMR (101 MHz, $CD_2Cl_2$) δ 151.0, 146.7, 144.0, 137.6, 137.2, 134.0, 130.9, 130.5, 127.8, 127.6, 121.6, 121.4, 75.8, 75.4, 35.0, 31.5, 3.8, 3.1.

**HRMS** (ESI/QTOF) m/z: $[M + Ag]^+$ Calcd for $C_{24}H_{26}AgGe^+$ 495.0292; Found 495.0310.

**IR (ATR)**: $\tilde{\nu}$ (cm$^{-1}$) = 3052, 2961, 2916, 2858, 1935, 1476, 1436, 1396, 1379, 1362, 1257, 1202, 1165, 1120, 1067, 1053, 1027, 923, 857, 831, 821, 775, 735, 685, 626, 456, 443, 419.

**Melting point**: -

**$R_f$**: 0.41 (dichloromethane: pentane = 1:4).

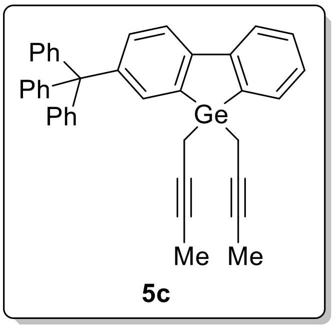


**Name**: 5,5-di(but-2-yn-1-yl)-3-trityl-5*H*-dibenzo[*b,d*]germole (**5c**).

**Synthesis**: Following General procedure D, (**Step 1**: using **g4** 1110 mg, 2.00 mmol; *n*BuLi 2.5 M in hexane, 1.6 mL, 4.10 mmol; $GeCl_4$ 2147 mg, 10.0 mmol. **Step 2**: but-2-yn-1-yl magnesium bromide 1.2 M in $Et_2O$, 6.7 mL, 8.0 mmol) the desired product **5c** was obtained in (28.5 mg, 0.05 mmol, 2% yield) as a white solid. Due to poor solubility of the starting material, low yield was obtained.

**$^1$H NMR** (400 MHz, $CD_2Cl_2$) δ 7.83 (d, *J* = 7.9 Hz, 1H), 7.75 (d, *J* = 8.4 Hz, 1H), 7.69 – 7.65 (m, 2H), 7.46 – 7.41 (m, 1H), 7.33 – 7.25 (m, 13H), 7.23 – 7.17 (m, 4H), 2.04 (dq, *J* = 5.4, 2.8 Hz, 4H), 1.62 (t, *J* = 2.8 Hz, 6H).

**$^{13}$C NMR** (101 MHz, $CD_2Cl_2$) δ 147.4, 146.7, 146.4, 144.4, 137.8, 136.8, 136.1, 134.0, 133.8, 131.4, 131.2, 130.5, 128.0, 127.8, 126.3, 121.8, 120.9, 75.8, 75.1, 65.4, 3.7, 3.0.

**HRMS** (ESI/QTOF) m/z: $[M + Ag]^+$ Calcd for $C_{39}H_{32}AgGe^+$ 681.0761; Found 681.0788.
**IR (ATR)**: $\tilde{\nu}$ ($cm^{-1}$) = 3052, 2961, 2916, 1436, 1396, 1379, 1362, 1257, 1120, 1067, 1053, 1027, 923, 857, 831, 821, 775, 626, 456, 443, 419.

**Appearance**: white solid.

**Melting point**: 172-173 $^o$C

**R$_f$**: 0.38 (dichloromethane: pentane = 1:4).

## Scope of enantioselective [2+2+2] cycloaddition

**General procedure E:**
In a glovebox, an oven dried screw-capped 2 mL vial was charged with a magnetic stir bar, Ni(0)NHC styrene complex **Ni3** (3 μmol), cyclohexane (0.5 mL) was then added. Dialkyne (0.1 mmol) and nitrile (0.1 mmol) was then added successively. The vial was sealed with a Teflon-lined screw cap, and running inside the glovebox at room temperature. After 24 h, the vial was shipped outside of the glovebox. The reaction mixture was diluted with dichloromethane and filtered through a plug of silica gel. The crude solution was concentrated in vacuum and subjected to column chromatography to isolate the products.

Note: The racemic trace was obtained by using complex IPr-Ni-(styrene)$_2$ (*68*) instead of **Ni3**.

General overview of the commercial nitriles.

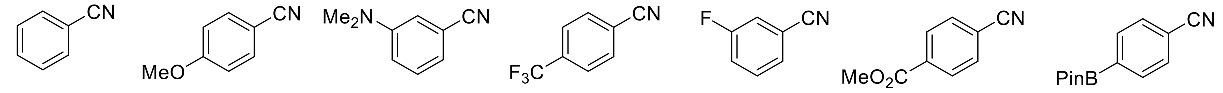


**6a** **6b** **6c** **6d** **6e** **6f** **6g**

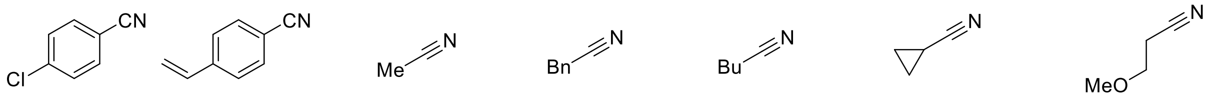


**6h** **6i** **6j** **6k** **6l** **6m** **6n**

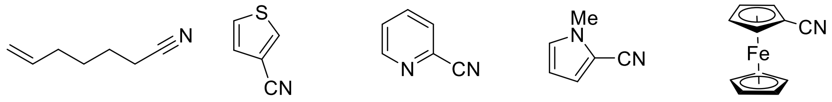


**6o** **6p** **6r** **6s** **6u**

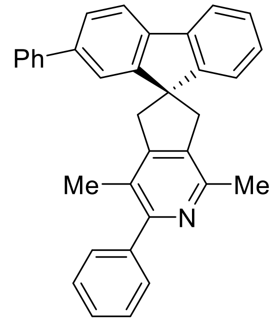

**7**

**Name**: (*R*)-1,4-dimethyl-2',3-diphenyl-5,7-dihydrospiro[cyclopenta[c]pyridine-6,9'-fluorene] (**7**).

**Synthesis**: Following a slight modified general procedure E (using **1a** 17.3 mg, 0.05 mmol; **6a** 5.2 mg, 0.05 mmol; **Ni3** 1.8 mg, 1.5 µmol) compound **7** was obtained in 95% yield and 92:8 er as a white foam.

**$^1$H NMR** (400 MHz, $CDCl_3$) δ 7.85 – 7.75 (m, 2H), 7.68 – 7.54 (m, 6H), 7.53 – 7.27 (m, 9H), 3.66 – 3.40 (m, 4H), 2.53 (s, 3H), 2.22 (s, 3H).

**$^{13}$C NMR** (101 MHz, $CDCl_3$) δ 157.1, 153.3, 153.2, 152.3, 151.0, 141.4, 141.2, 141.0, 139.2, 139.0, 135.2, 129.4, 129.0, 128.3, 128.0, 127.8, 127.8, 127.4, 127.3, 126.9, 124.8, 122.3, 121.2, 120.4, 120.1, 56.6, 45.3, 44.3, 22.2, 16.5.

**HRMS** (ESI/QTOF) m/z: $[M + H]^+$ Calcd for $C_{34}H_{28}N^+$ 450.2216; Found 450.2225.
$[\alpha]_D^{20}$**:** 53.3 (c = 0.25, $CHCl_3$).

**IR (ATR)**: $\tilde{\nu}$ ($cm^{-1}$) = 3056, 3030, 2923, 2851, 1572, 1496, 1481, 1467, 1452, 1426, 1412, 1379, 1346, 1261, 1198, 1157, 1073, 1021, 970, 916, 891, 834, 802, 784, 759, 737, 698, 677, 647, 613, 575, 506, 475, 447.

**Melting point**: -

**$R_f$**: 0.49 (ethyl acetate: pentane = 1:4).

**HPLC** Chiralpak IC 4.6 x 250 mm; hexane:*i*-PrOH 95:5, 1.0 mL/min, 210 nm; *t*R (major) = 9.8 min, *t*R (minor) = 11.1 min, 92:8 er.

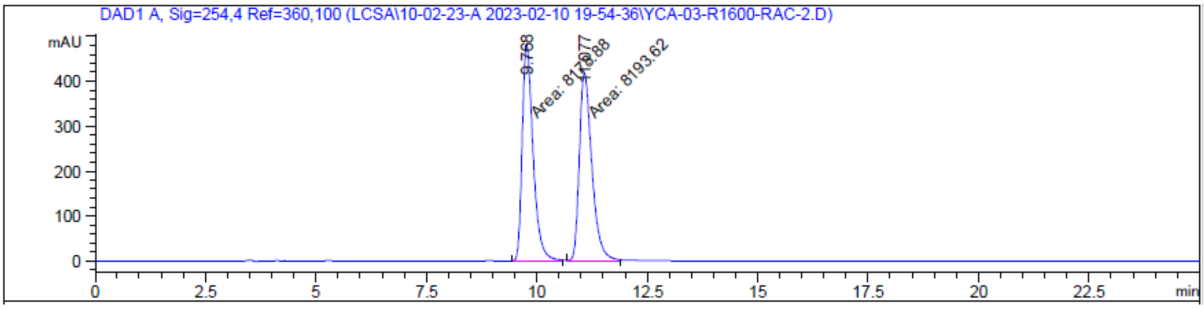


Signal 1: DAD1 A, Sig=254,4 Ref=360,100

| Peak # | RetTime [min] | Type | Width [min] | Area [mAU*s] | Height [mAU] | Area % |
|---|---|---|---|---|---|---|
| 1 | 9.768 | MM | 0.2834 | 8178.87646 | 480.95544 | 49.9550 |
| 2 | 11.077 | MM | 0.3277 | 8193.62109 | 416.71527 | 50.0450 |

Totals : 1.63725e4 897.67072

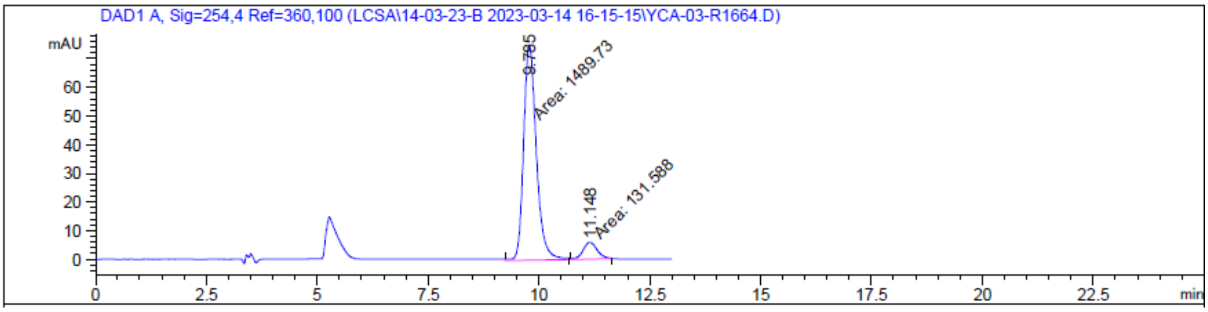


Signal 1: DAD1 A, Sig=254,4 Ref=360,100

| Peak # | RetTime [min] | Type | Width [min] | Area [mAU*s] | Height [mAU] | Area % |
|---|---|---|---|---|---|---|
| 1 | 9.785 | MM | 0.3314 | 1489.72693 | 74.92619 | 91.8839 |
| 2 | 11.148 | MM | 0.3716 | 131.58829 | 5.90139 | 8.1161 |

Totals : 1621.31522 80.82758

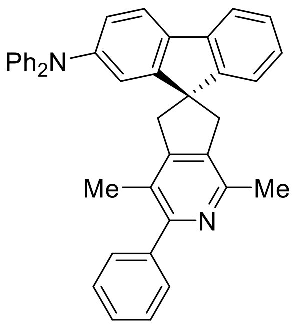


**8a**

**Name**: (*R*)-1,4-dimethyl-N,N,3-triphenyl-5,7-dihydrospiro[cyclopenta[c]pyridine-6,9'-fluoren]-2'-amine (**8a**)

**Synthesis**: Following general procedure E, (using **1d** 43.8 mg, 0.1 mmol; **6a** 10.3 mg, 0.1 mmol; **Ni3** 3.6 mg, 3.0 μmol) compound **8a** was obtained in 51.5 mg, 0.0952 mmol, 95% yield and 95:5 er as a pale-yellow foam.

**$^{1}$H NMR** (400 MHz, $CDCl_3$) δ 7.71 – 7.64 (m, 1H), 7.63 – 7.57 (m, 1H), 7.54 – 7.42 (m, 4H), 7.41 – 7.32 (m, 2H), 7.30 – 7.22 (m, 4H), 7.21 – 7.17 (m, 2H), 7.17 – 7.05 (m, 6H), 7.06 – 6.99 (m, 2H), 3.45 – 3.30 (m, 4H), 2.46 (s, 3H), 2.15 (s, 3H).

**$^{13}$C NMR** (101 MHz, $CDCl_3$) δ 157.0, 153.3, 152.8, 152.3, 150.7, 148.0, 147.9, 141.0, 139.3, 135.1, 134.4, 129.4, 128.2, 127.7, 127.7, 127.0, 124.7, 124.4, 123.3, 123.1, 122.0, 120.6, 119.4, 117.9, 56.6, 45.1, 44.1, 22.1, 16.4.

**HRMS** (ESI/QTOF) m/z: $[M + H]^+$ Calcd for $C_{40}H_{33}N_2^+$ 541.2638; Found 541.2659.
$[\alpha]_D^{20}$**:** 61.5 (c = 0.39, $CHCl_3$).

**IR (ATR)**: $\tilde{\nu}$ ($cm^{-1}$) = 3010, 2925, 2853, 1584, 1487, 1450, 1427, 1379, 1346, 1331, 1314, 1276, 1216, 1175, 1155, 1074, 1025, 970, 803, 747, 696, 666, 620, 572, 513, 453, 407.

**Melting point**: -

**$R_f$**: 0.50 (ethyl acetate: pentane = 1:4).

**HPLC** Chiralpak IC 4.6 x 250 mm; hexane:*i*-PrOH 99:1, 1.0 mL/min, 210 nm; *t*R (major) = 7.3 min, *t*R (minor) = 9.6 min, 95:5 er.

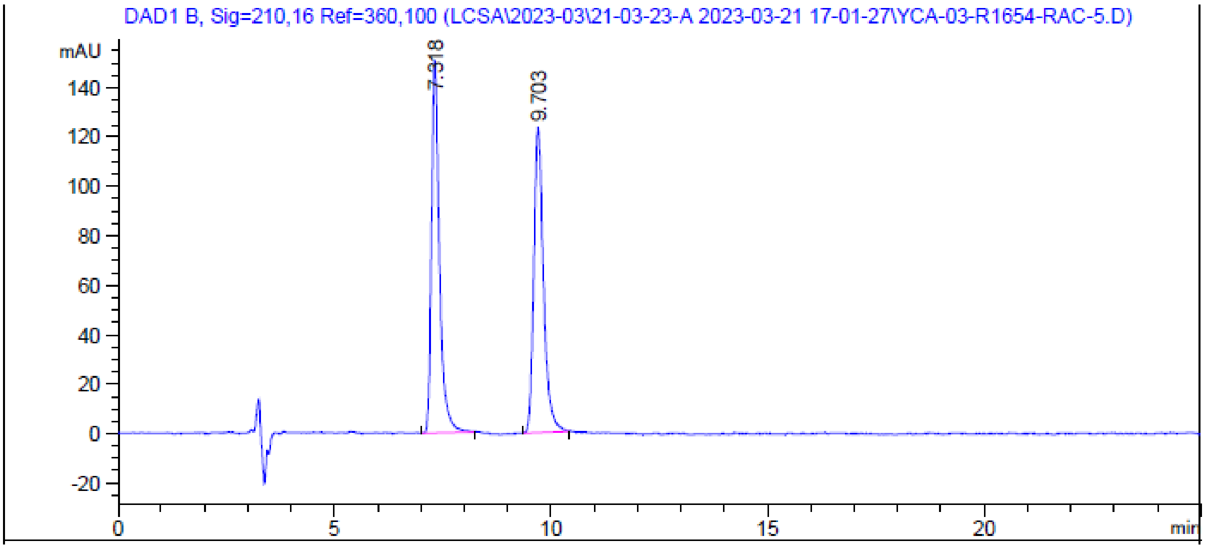


Signal 2: DAD1 B, Sig=210,16 Ref=360,100

| Peak # | RetTime [min] | Type | Width [min] | Area [mAU*s] | Height [mAU] | Area % |
|---|---|---|---|---|---|---|
| 1 | 7.318 | BB | 0.1921 | 1939.72107 | 150.78099 | 50.0707 |
| 2 | 9.703 | BB | 0.2385 | 1934.24402 | 123.62061 | 49.9293 |

Totals : 3873.96509 274.40160

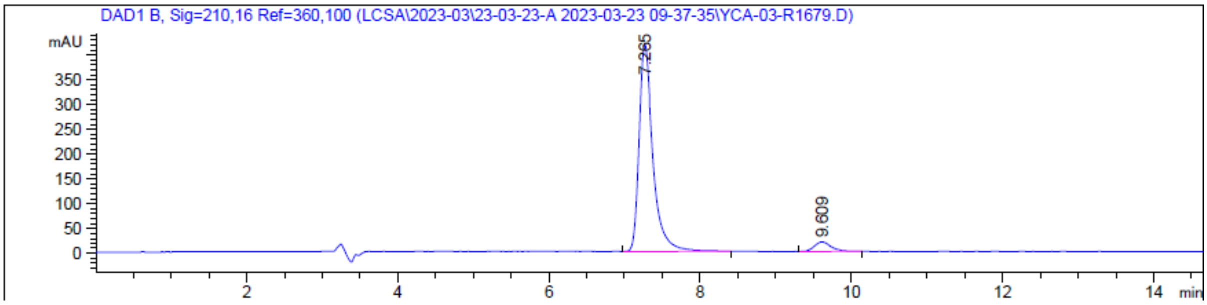


Signal 2: DAD1 B, Sig=210,16 Ref=360,100

| Peak # | RetTime [min] | Type | Width [min] | Area [mAU*s] | Height [mAU] | Area % |
|---|---|---|---|---|---|---|
| 1 | 7.265 | VB | 0.1943 | 5394.69580 | 418.76205 | 94.7969 |
| 2 | 9.609 | BB | 0.2392 | 296.09473 | 19.06330 | 5.2031 |

Totals : 5690.79053 437.82536

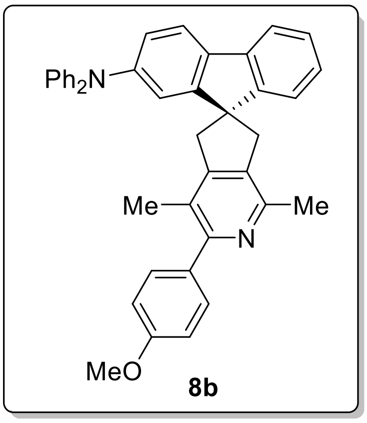


**Name**: (*R*)-3-(4-methoxyphenyl)-1,4-dimethyl-*N*,*N*-diphenyl-5,7-dihydrospiro[cyclopenta[c]pyridine-6,9'-fluoren]-2'-amine (**8b**).

**Synthesis**: Following general procedure E (using **1d** 43.8 mg, 0.1 mmol; **6b** 13.3 mg, 0.1 mmol; **Ni3** 3.6 mg, 3.0 μmol) compound **8b** was obtained in 51.6 mg, 0.904 mmol, 90% yield and 96.5:3.5 er as colorless thick oil.

**$^1$H NMR** (400 MHz, $CDCl_3$) δ 7.65 (d, *J* = 7.6 Hz, 1H), 7.62 – 7.57 (m, 1H), 7.48 – 7.42 (m, 2H), 7.38 – 7.31 (m, 1H), 7.29 – 7.21 (m, 4H), 7.19 – 7.15 (m, 2H), 7.15 – 7.06 (m, 6H), 7.05 – 6.95 (m, 4H), 3.87 (s, 3H), 3.47 – 3.26 (m, 4H), 2.44 (s, 3H), 2.16 (s, 3H).

**$^{13}$C NMR** (101 MHz, $CDCl_3$) δ 159.3, 156.7, 153.4, 152.9, 152.2, 150.6, 148.0, 147.9, 139.3, 134.7, 134.4, 133.6, 130.6, 129.4, 127.7, 127.0, 124.5, 124.4, 123.3, 123.0, 122.0, 120.6, 119.4, 118.0, 113.6, 56.6, 55.5, 45.1, 44.1, 22.1, 16.5.

**HRMS** (ESI/QTOF) m/z: $[M + H]^+$ Calcd for $C_{41}H_{35}N_2O^+$ 571.2744; Found 571.2759.
**$[\alpha]_D^{20}$:** 75.0 (c = 0.34, $CHCl_3$).

**IR (ATR)**: $\tilde{\nu}$ ($cm^{-1}$) = 3007, 2923, 2852, 1609, 1584, 1513, 1486, 1450, 1427, 1408, 1378, 1331, 1314, 1276, 1245, 1218, 1175, 1106, 1073, 1029, 970, 835, 806, 750, 696, 666, 625, 570, 515, 456.

**Melting point**: -

**$R_f$**: 0.31 (ethyl acetate: pentane = 1:4).

**HPLC**: Chiralpak IC 4.6 x 250 mm; hexane:*i*-PrOH 90:10, 1 mL/min, 254 nm; *t*R (major) = 7.8 min, *t*R (minor) = 11.5 min, 96.5:3.5 er.

**Chromatogram**

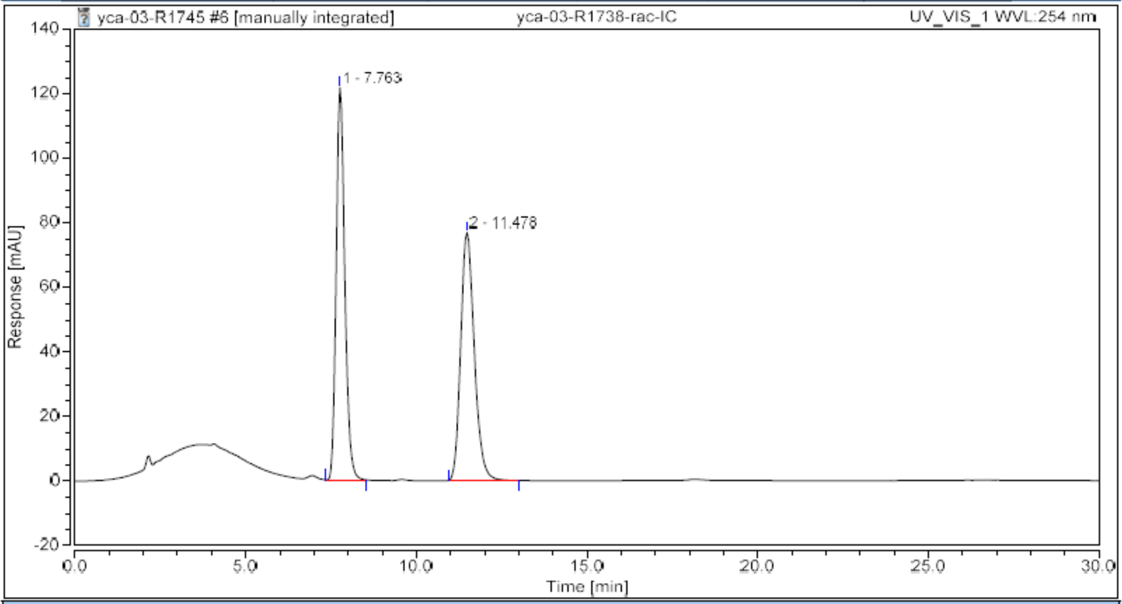


**Integration Results**

| No. | Peak Name | Retention Time<br>min | Area<br>mAU*min | Height<br>mAU | Relative Area<br>% | Relative Height<br>% | Amount<br>n.a. |
|---|---|---|---|---|---|---|---|
| 1 | | 7.763 | 35.417 | 121.552 | 49.99 | 61.23 | n.a. |
| 2 | | 11.478 | 35.429 | 76.953 | 50.01 | 38.77 | n.a. |
| **Total:** | | | **70.846** | **198.505** | **100.00** | **100.00** | |

**Chromatogram**

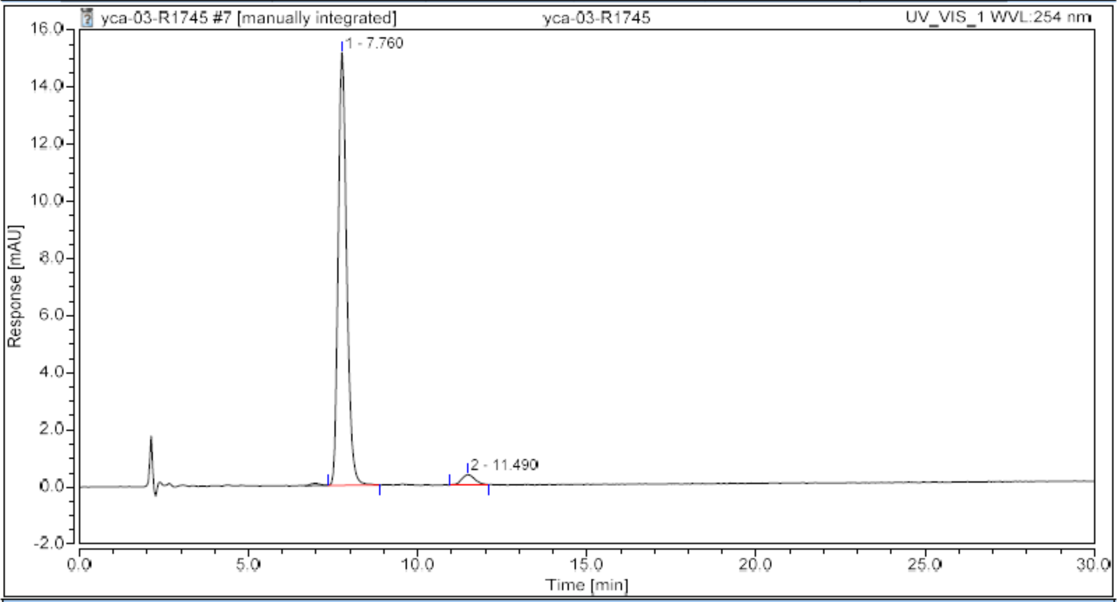


**Integration Results**

| No. | Peak Name | Retention Time<br>min | Area<br>mAU*min | Height<br>mAU | Relative Area<br>% | Relative Height<br>% | Amount<br>n.a. |
|---|---|---|---|---|---|---|---|
| 1 | | 7.760 | 4.429 | 15.151 | 96.50 | 97.69 | n.a. |
| 2 | | 11.490 | 0.161 | 0.358 | 3.50 | 2.31 | n.a. |
| **Total:** | | | **4.590** | **15.508** | **100.00** | **100.00** | |

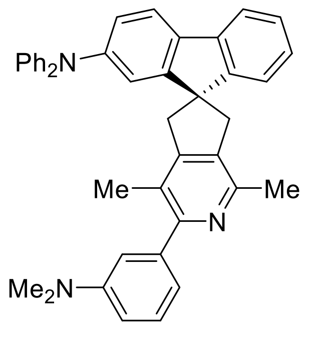


**8c**

**Name**: (*R*)-3-(3-(dimethylamino)phenyl)-1,4-dimethyl-*N*,*N*-diphenyl-5,7-dihydrospiro[cyclopenta[c]pyridine-6,9'-fluoren]-2'-amine (**8c**).

**Synthesis**: Following general procedure E, (using **1d** 43.8 mg, 0.1 mmol; **6c** 14.6 mg, 0.1 mmol; **Ni3** 3.6 mg, 3.0 μmol) compound **8c** was obtained in 49.6 mg, 0.0849 mmol, 85% yield and 92:8 er as a colorless thick oil.

**$^1$H NMR** (400 MHz, $CDCl_3$) δ 7.65 (d, *J* = 7.5 Hz, 1H), 7.59 (d, *J* = 8.0 Hz, 1H), 7.40 – 7.28 (m, 2H), 7.28 – 7.21 (m, 4H), 7.19 – 7.15 (m, 2H), 7.14 – 7.06 (m, 6H), 7.01 (t, *J* = 7.3 Hz, 2H), 6.88 – 6.71 (m, 3H), 3.44 – 3.28 (m, 4H), 2.99 (s, 6H), 2.45 (s, 3H), 2.15 (s, 3H).

**$^{13}$C NMR** (101 MHz, $CDCl_3$) δ 158.0, 153.4, 153.1, 152.0, 150.7, 150.4, 148.0, 147.9, 141.7, 139.2, 134.8, 134.4, 129.4, 128.8, 127.7, 127.0, 124.7, 124.4, 123.3, 123.0, 122.0, 120.6, 119.4, 118.0, 117.9, 113.7, 112.2, 56.5, 45.1, 44.2, 40.9, 22.1, 16.4.

**HRMS** (ESI/QTOF) m/z: $[M + H]^+$ Calcd for $C_{42}H_{38}N_3^+$ 584.3060; Found 584.3084.
$[\boldsymbol{\alpha}]_D^{20}$**:** 60.6 (c = 0.22, $CHCl_3$).

**IR (ATR)**: $\tilde{\nu}$ ($cm^{-1}$) = 3007, 2926, 1598, 1584, 1486, 1450, 1427, 1376, 1347, 1330, 1314, 1275, 1260, 1216, 1181, 1154, 1073, 1024, 950, 860, 800, 747, 696, 665, 573, 513, 450.

**Melting point**: -

**$R_f$**: 0.54 (ethyl acetate: pentane = 1:1).

**HPLC:** Chiralpak ID 4.6 x 250 mm; hexane:*i*-PrOH 95:5, 1 mL/min, 254 nm; *t*R (major) = 15.8 min, *t*R (minor) = 21.6 min, 92:8 er.

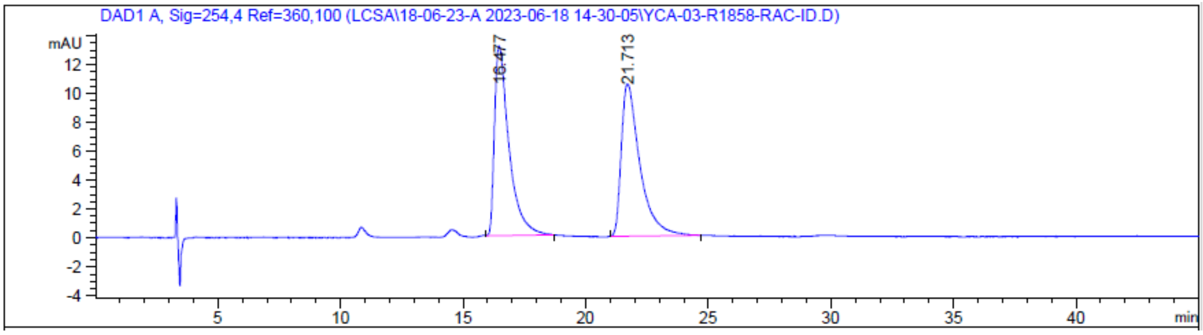


Signal 1: DAD1 A, Sig=254,4 Ref=360,100

| Peak # | RetTime [min] | Type | Width [min] | Area [mAU*s] | Height [mAU] | Area % |
|---|---|---|---|---|---|---|
| 1 | 16.477 | BB | 0.5915 | 539.48926 | 13.12735 | 49.1625 |
| 2 | 21.713 | BB | 0.7388 | 557.87067 | 10.55215 | 50.8375 |

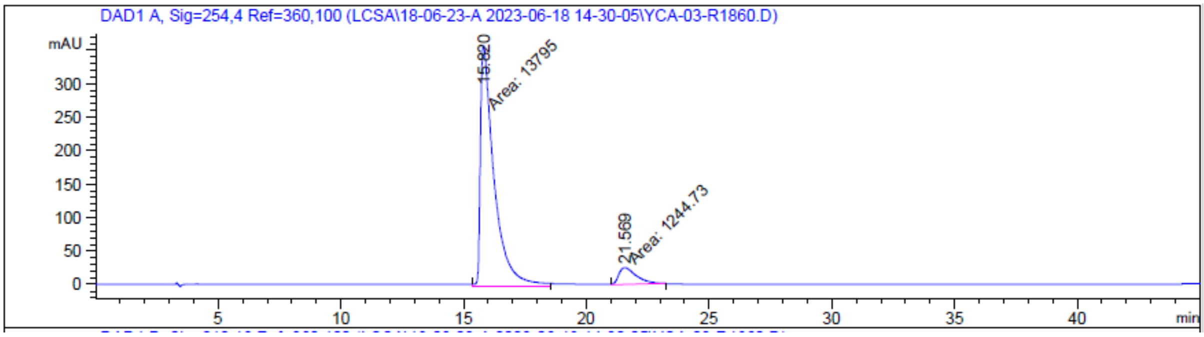


Signal 1: DAD1 A, Sig=254,4 Ref=360,100

| Peak # | RetTime [min] | Type | Width [min] | Area [mAU*s] | Height [mAU] | Area % |
|---|---|---|---|---|---|---|
| 1 | 15.820 | MM | 0.6423 | 1.37950e4 | 357.93314 | 91.7237 |
| 2 | 21.569 | MM | 0.8289 | 1244.72839 | 25.02908 | 8.2763 |

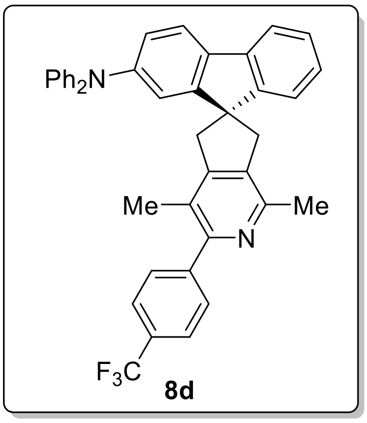

**Name**: (*R*)-1,4-dimethyl-*N*,*N*-diphenyl-3-(4-(trifluoromethyl)phenyl)-5,7-dihydrospiro[cyclopenta[c]pyridine-6,9'-fluoren]-2'-amine (**8d**).

**Synthesis**: Following general procedure E, (using **1d** 43.8 mg, 0.1 mmol; **6d** 17.1 mg, 0.1 mmol; **Ni3** 3.6 mg, 3.0 μmol) compound **8d** was obtained in 60.8 mg, 0.0998 mmol, >99% yield and 95:5 er as a pink thick oil.

**$^{1}$H NMR** (400 MHz, $CDCl_3$) δ 7.76 – 7.68 (m, 2H), 7.65 (d, *J* = 7.5 Hz, 1H), 7.63 – 7.56 (m, 3H), 7.34 (dq, *J* = 8.2, 4.1 Hz, 1H), 7.29 – 7.21 (m, 4H), 7.20 – 7.15 (m, 2H), 7.14 – 6.97 (m, 8H), 3.46 – 3.31 (m, 4H), 2.45 (s, 3H), 2.14 (s, 3H).

**$^{13}$C NMR** (101 MHz, $CDCl_3$) δ 155.5, 153.2, 152.6, 152.5, 151.1, 148.1, 147.9, 144.6, 139.4, 135.9, 134.3, 130.2 – 129.5 (m), 129.4, 127.8, 127.0, 125.8, 125.2 (q, *J* = 3.7 Hz), 124.7, 124.4, 123.4, 123.1, 122.0, 120.7, 119.5, 117.8, 56.6, 45.0, 44.2, 22.1, 16.3.

**$^{19}$F NMR** (376 MHz, $CDCl_3$) δ -62.4.

**HRMS** (ESI/QTOF) m/z: $[M + H]^+$ Calcd for $C_{41}H_{32}F_3N_2^+$ 609.2512; Found 609.2519.

$[\alpha]_D^{20}$**:** 55.4 (c = 0.28, $CHCl_3$).

**IR (ATR)**: $\tilde{\nu}$ ($cm^{-1}$) = 2923, 1586, 1489, 1452, 1428, 1404, 1324, 1278, 1220, 1165, 1124, 1107, 1066, 1018, 971, 849, 823, 801, 779, 754, 736, 697, 668, 651, 630, 515, 429, 406.

**Appearance**: pink thick oil.
**Melting point**: -
**$R_f$**: 0.78 (ethyl acetate: pentane = 1:4).
**HPLC**: Chiralpak IC 4.6 x 250 mm; hexane:*i*-PrOH 98:2, 1 mL/min, 254 nm; *t*R (major) = 4.0 min, *t*R (minor) = 4.8 min, 95:5 er.

**Chromatogram**

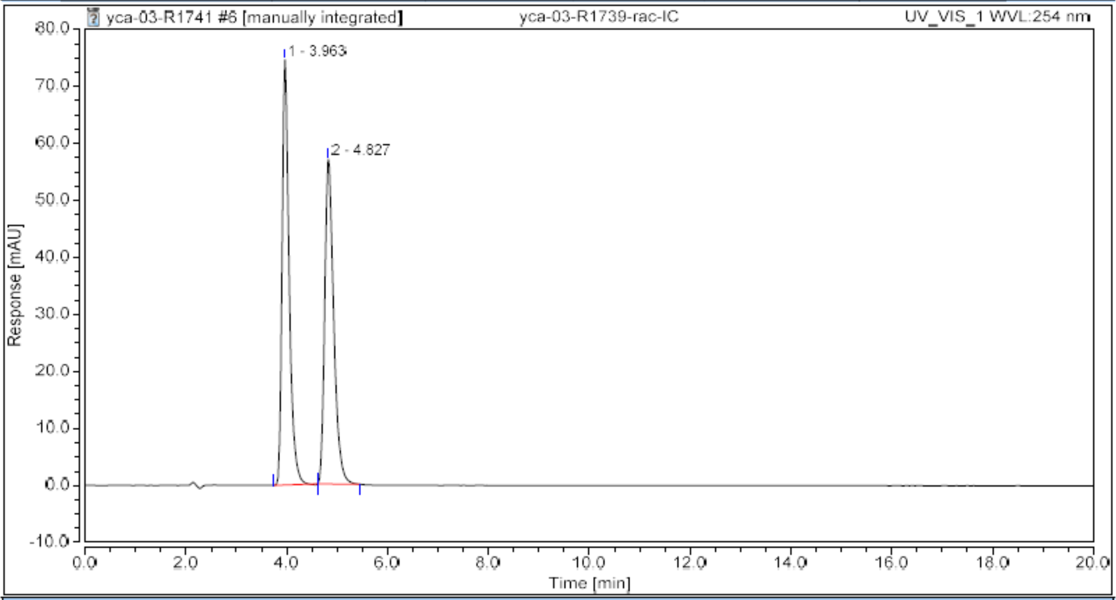


**Integration Results**

| No. | Peak Name | Retention Time min | Area mAU*min | Height mAU | Relative Area % | Relative Height % | Amount n.a. |
|---|---|---|---|---|---|---|---|
| 1 | | 3.963 | 12.061 | 74.560 | 50.55 | 56.68 | n.a. |
| 2 | | 4.827 | 11.797 | 56.977 | 49.45 | 43.32 | n.a. |
| **Total:** | | | **23.858** | **131.537** | **100.00** | **100.00** | |

**Chromatogram**

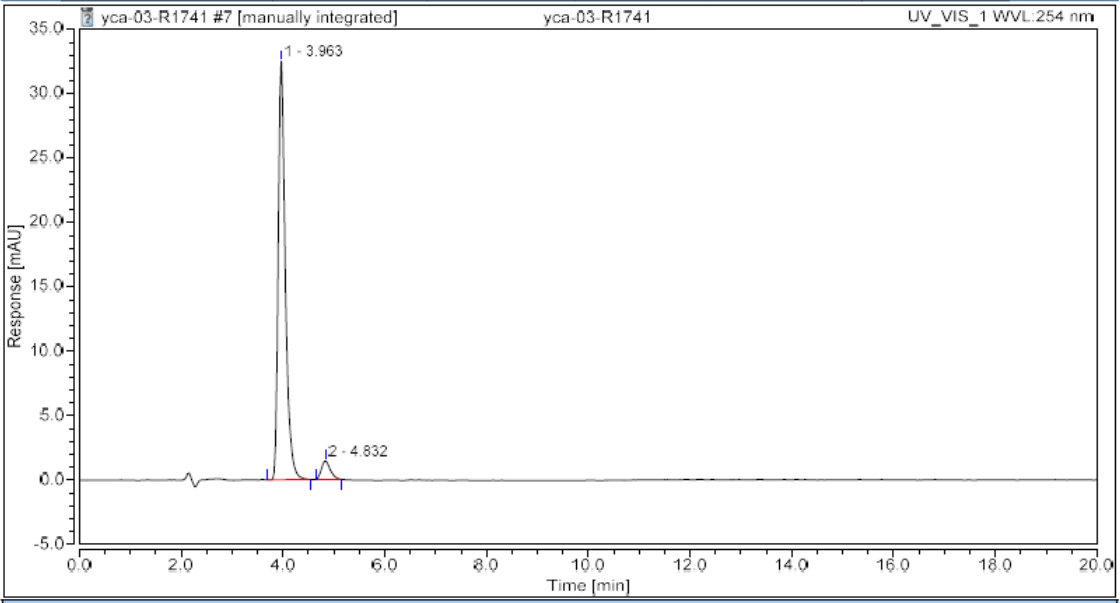


**Integration Results**

| No. | Peak Name | Retention Time min | Area mAU*min | Height mAU | Relative Area % | Relative Height % | Amount n.a. |
|---|---|---|---|---|---|---|---|
| 1 | | 3.963 | 5.236 | 32.522 | 94.81 | 95.76 | n.a. |
| 2 | | 4.832 | 0.287 | 1.439 | 5.19 | 4.24 | n.a. |
| **Total:** | | | **5.523** | **33.961** | **100.00** | **100.00** | |

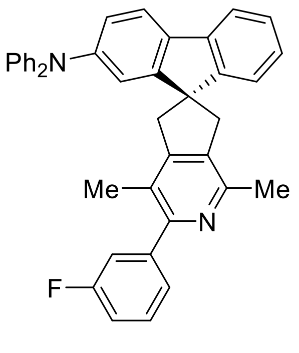


**8e**

**Name**: (*R*)-3-(3-fluorophenyl)-1,4-dimethyl-*N*,*N*-diphenyl-5,7-dihydrospiro[cyclopenta[c]pyridine-6,9'-fluoren]-2'-amine (**8e**).

**Synthesis**: Following general procedure E, (using **1d** 43.8 mg, 0.1 mmol; **6e** 12.1 mg, 0.1 mmol; **Ni3** 3.6 mg, 3.0 µmol) compound **8e** was obtained in 53.3 mg, 0.0954 mmol, 95% yield and 91:9 er as a colorless thick oil.

**$^{1}$H NMR** (400 MHz, $CDCl_3$) δ 7.65 (d, *J* = 7.6 Hz, 1H), 7.59 (d, *J* = 8.3 Hz, 1H), 7.43 – 7.31 (m, 2H), 7.29 – 7.16 (m, 8H), 7.15 – 6.98 (m, 9H), 3.47 – 3.28 (m, 4H), 2.45 (s, 3H), 2.14 (s, 3H).

**$^{13}$C NMR** (101 MHz, $CDCl_3$) δ 164.0, 161.5, 155.6 (d, *J* = 3.1 Hz), 153.3, 152.6, 152.4, 150.9, 148.0, 147.8, 143.2 (d, *J* = 7.4 Hz), 139.4, 135.6, 134.3, 129.7 (d, *J* = 8.1 Hz), 129.4, 127.8, 127.0, 125.1 (d, *J* = 3.1 Hz), 124.6, 124.4, 123.3, 123.1, 122.0, 120.7, 119.4, 117.8, 116.5 (d, *J* = 22.3 Hz), 114.6 (d, *J* = 21.1 Hz), 56.6, 45.0, 44.1, 22.1, 16.1.

**$^{19}$F NMR** (376 MHz, $CDCl_3$) δ -113.6.

**HRMS** (ESI/QTOF) m/z: $[M + H]^+$ Calcd for $C_{40}H_{32}FN_2^+$ 559.2544; Found 559.2558.

$[\alpha]_D^{20}$**:** 57.9 (c = 0.19, $CHCl_3$).

**IR (ATR)**: $\tilde{\nu}$ ($cm^{-1}$) =: 1912, 1888, 1865, 1846, 1822, 1794, 1778, 1755, 1741, 1719, 1698, 1684, 1656, 1613, 1585, 1489, 1468, 1451, 1427, 1413, 1375, 1331, 1314, 1279, 1265, 1242, 1218, 1190, 1174, 1155, 1118, 1102, 1078, 1025, 948, 934, 897, 882, 867, 852, 822, 804, 794, 776, 753, 737, 721, 697, 669, 650, 633, 623, 610, 598, 588, 574, 565, 554, 538, 516, 500, 493, 472, 464, 455, 444, 421, 401.

**Melting point**: -

**R$_f$**: 0.52 (ethyl acetate: pentane = 1:4).

**HPLC:** Chiralpak IC 4.6 x 250 mm; hexane:*i*-PrOH 98:2, 1 mL/min, 220 nm; *t*R (major) = 10.5 min, *t*R (minor) = 15.0 min, 91:9 er.

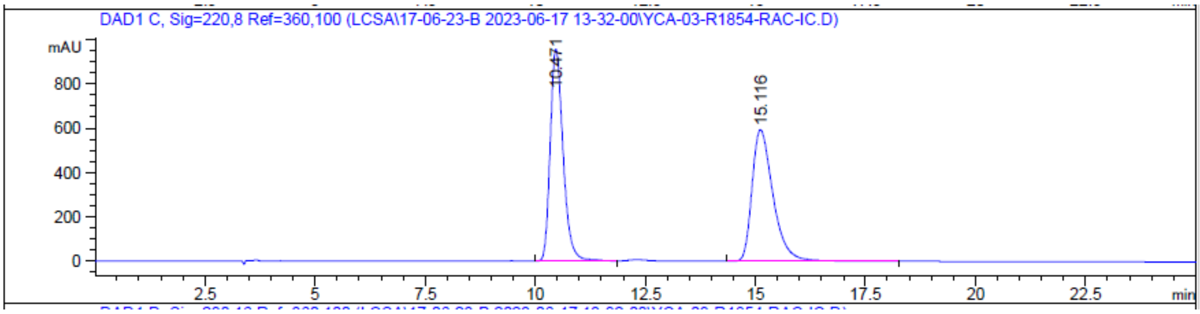


Signal 3: DAD1 C, Sig=220,8 Ref=360,100

| Peak # | RetTime [min] | Type | Width [min] | Area [mAU*s] | Height [mAU] | Area % |
|---|---|---|---|---|---|---|
| 1 | 10.471 | BB | 0.3136 | 1.95024e4 | 957.77020 | 49.7010 |
| 2 | 15.116 | BB | 0.5075 | 1.97370e4 | 594.26282 | 50.2990 |

Totals : 3.92394e4 1552.03302

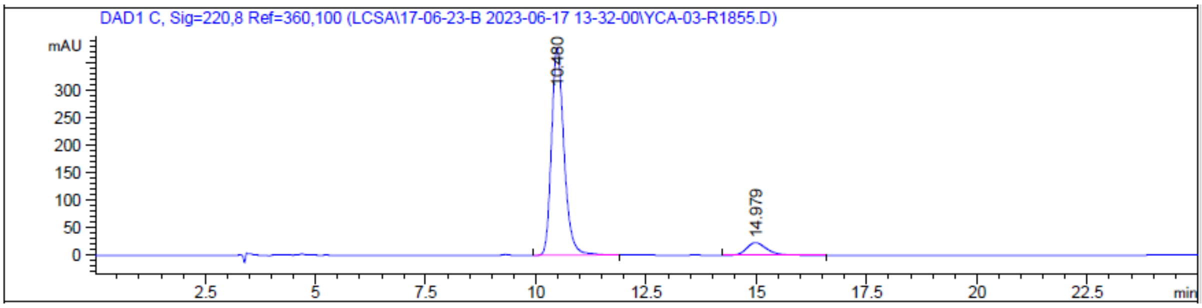


Signal 3: DAD1 C, Sig=220,8 Ref=360,100

| Peak # | RetTime [min] | Type | Width [min] | Area [mAU*s] | Height [mAU] | Area % |
|---|---|---|---|---|---|---|
| 1 | 10.480 | BB | 0.3018 | 7433.51270 | 377.32587 | 90.9877 |
| 2 | 14.979 | BB | 0.4886 | 736.29114 | 22.81140 | 9.0123 |

Totals : 8169.80383 400.13727

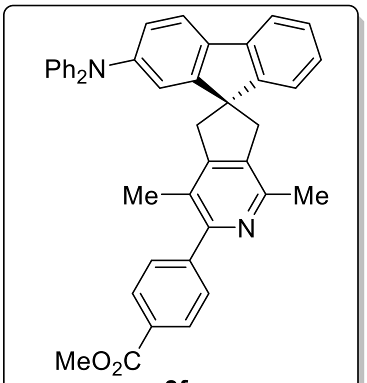


**8f**

**Name**: methyl (*R*)-4-(2'-(diphenylamino)-1,4-dimethyl-5,7-dihydrospiro[cyclopenta[c]pyridine-6,9'-fluoren]-3-yl)benzoate (**8f**).

**Synthesis**: Following general procedure E, (using **1d** 43.8 mg, 0.1 mmol; **6f** 16.1 mg, 0.1 mmol; **Ni3** 3.6 mg, 3.0 μmol) compound **8f** was obtained in 55.6 mg, 0.0928 mmol, 93% yield and 91:9 er as a colorless oil.

**$^1$H NMR** (400 MHz, $CDCl_3$) δ 8.19 – 8.08 (m, 2H), 7.65 (d, *J* = 7.5 Hz, 1H), 7.62 – 7.54 (m, 3H), 7.34 (dp, *J* = 7.7, 4.1 Hz, 1H), 7.29 – 7.21 (m, 4H), 7.20 – 7.16 (m, 2H), 7.14 – 6.98 (m, 8H), 3.95 (s, 3H), 3.44 – 3.28 (m, 4H), 2.45 (s, 3H), 2.13 (s, 3H).

**$^{13}$C NMR** (101 MHz, $CDCl_3$) δ 167.2, 155.9, 153.2, 152.6, 152.4, 151.0, 148.0, 147.8, 145.6, 139.3, 135.8, 134.3, 129.6, 129.5, 129.4, 129.3, 127.8, 127.0, 124.8, 124.4, 123.3, 123.1, 122.0, 120.7, 119.4, 117.8, 56.6, 52.3, 45.0, 44.1, 22.1, 16.3.

**HRMS** (ESI/QTOF) m/z: $[M + H]^+$ Calcd for $C_{42}H_{35}N_2O_2^+$ 599.2693; Found 599.2700.

$[\alpha]_D^{20}$**:** 73.4 (c = 0.24, $CHCl_3$).

**IR (ATR)**: $\tilde{\nu}$ ($cm^{-1}$) =: 2365, 2357, 2342, 2331, 2310, 2240, 2214, 2205, 2181 2160, 2149, 2142, 2095, 2078, 2032, 2022, 2006, 1979, 1932, 1889, 1723, 1609, 1587, 1488, 1468, 1451, 1427, 1408, 1372, 1346, 1310, 1276, 1218, 1197, 1176, 1155, 1103, 1080, 1044, 1019, 989, 973, 958, 889, 865, 830, 812, 790, 778, 757, 736, 696, 680, 664, 615, 608, 585, 555, 518, 502, 488, 468, 456, 436, 425, 405.

**Melting point**: -

**$R_f$**: 0.30 (ethyl acetate: pentane = 1:4).

**HPLC:** Chiralpak IF 4.6 x 250 mm; hexane:*i*-PrOH 95:5, 1 mL/min, 230 nm; *t*R (major) = 14.4 min, *t*R (minor) = 23.9 min, 91:9 er.

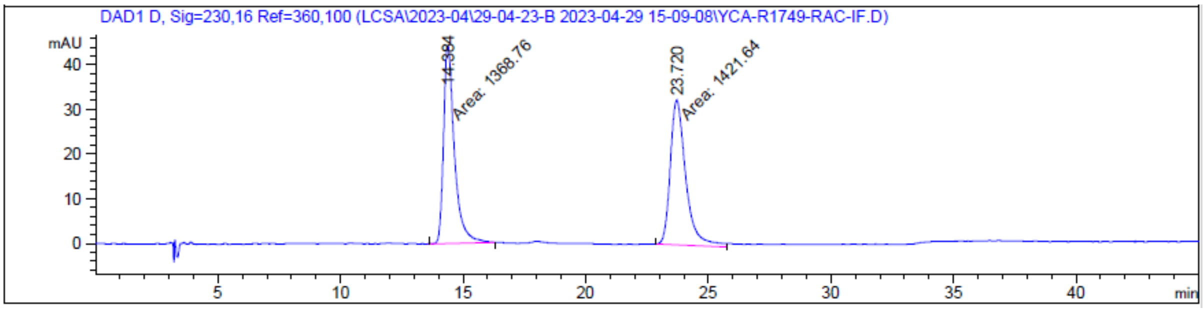


Signal 3: DAD1 D, Sig=230,16 Ref=360,100

| Peak # | RetTime [min] | Type | Width [min] | Area [mAU*s] | Height [mAU] | Area % |
|---|---|---|---|---|---|---|
| 1 | 14.384 | MM | 0.5141 | 1368.76404 | 44.37308 | 49.0526 |
| 2 | 23.720 | MM | 0.7305 | 1421.63550 | 32.43626 | 50.9474 |

Totals : 2790.39954 76.80934

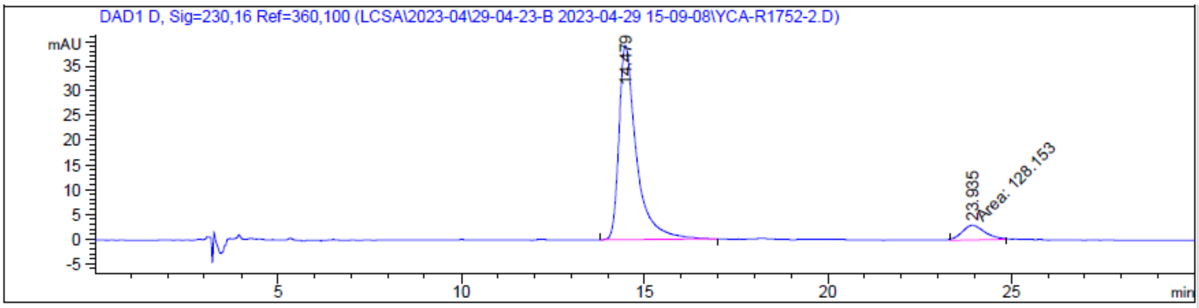


Signal 3: DAD1 D, Sig=230,16 Ref=360,100

| Peak # | RetTime [min] | Type | Width [min] | Area [mAU*s] | Height [mAU] | Area % |
|---|---|---|---|---|---|---|
| 1 | 14.479 | BB | 0.4886 | 1302.04224 | 39.28980 | 91.0395 |
| 2 | 23.935 | MM | 0.7194 | 128.15269 | 2.96905 | 8.9605 |

Totals : 1430.19493 42.25885

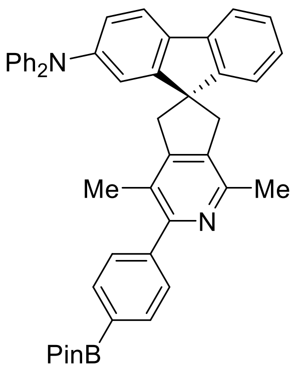

**8g**

**Name**: (*R*)-1,4-dimethyl-*N*,*N*-diphenyl-3-(4-(4,4,5,5-tetramethyl-1,3,2-dioxaborolan-2-yl)phenyl)-5,7-dihydrospiro[cyclopenta[c]pyridine-6,9'-fluoren]-2'-amine (**8g**).

**Synthesis**: Following general procedure E, (using **1d** 43.8 mg, 0.1 mmol; **6g** 22.9 mg, 0.1 mmol; **Ni3** 3.6 mg, 3.0 μmol) compound **8g** was obtained in 53.2 mg, 0.0798 mmol, 80% yield and 91:9 er as a pink thick oil.

**$^{1}$H NMR** (400 MHz, $CDCl_3$) δ 7.89 (d, *J* = 8.1 Hz, 2H), 7.65 (d, *J* = 7.5 Hz, 1H), 7.59 (d, *J* = 8.1 Hz, 1H), 7.48 (d, *J* = 8.1 Hz, 2H), 7.37 – 7.31 (m, 1H), 7.30 – 7.21 (m, 4H), 7.21 – 7.15 (m, 2H), 7.14 – 6.97 (m, 8H), 3.44 – 3.26 (m, 4H), 2.45 (s, 3H), 2.12 (s, 3H), 1.38 (s, 12H).

**$^{11}$B NMR** (128 MHz, $CDCl_3$) δ -6.9.

**$^{13}$C NMR** (101 MHz, $CDCl_3$) δ 156.9, 153.4, 152.7, 152.2, 150.7, 148.0, 147.8, 143.8, 139.3, 135.2, 134.7, 134.3, 129.4, 128.7, 127.7, 127.0, 124.7, 124.4, 123.3, 123.1, 122.0, 120.6, 119.4, 117.9, 83.9, 56.6, 45.0, 44.1, 25.0, 22.0, 16.3.

**HRMS** (ESI/QTOF) m/z: $[M + H]^+$ Calcd for $C_{46}H_{44}BN_2O_2^+$ 667.3490; Found 667.3519.

**$[\alpha]_D^{20}$:** 63.5 (c = 0.46, $CHCl_3$).

**IR (ATR)**: $\tilde{\nu}$ ($cm^{-1}$) = 3036, 2977, 2928, 1610, 1585, 1550, 1517, 1488, 1451, 1427, 1393, 1358, 1318, 1301, 1274, 1216, 1143, 1088, 1021, 962, 859, 845, 827, 799, 778, 754, 736, 697, 678, 662, 575, 516, 458.

**Melting point**: -

**$R_f$**: 0.32 (ethyl acetate: pentane = 1:4).

**HPLC:** Chiralpak ID 4.6 x 250 mm; hexane:*i*-PrOH 99:1, 1 mL/min, 254 nm; *t*R (major) = 29.3 min, *t*R (minor) = 41.2 min, 91:9 er.

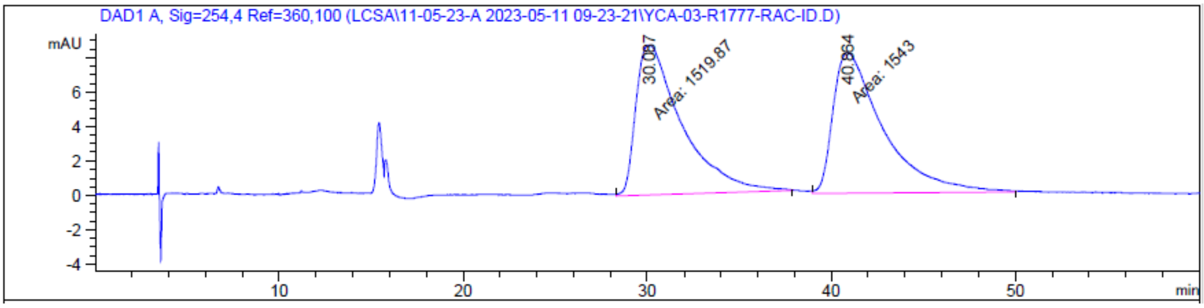


Signal 1: DAD1 A, Sig=254,4 Ref=360,100

| Peak # | RetTime [min] | Type | Width [min] | Area [mAU*s] | Height [mAU] | Area % |
|---|---|---|---|---|---|---|
| 1 | 30.087 | MM | 2.9046 | 1519.86536 | 8.72102 | 49.6224 |
| 2 | 40.864 | MM | 3.1464 | 1542.99609 | 8.17337 | 50.3776 |

Totals : 3062.86145 16.89440

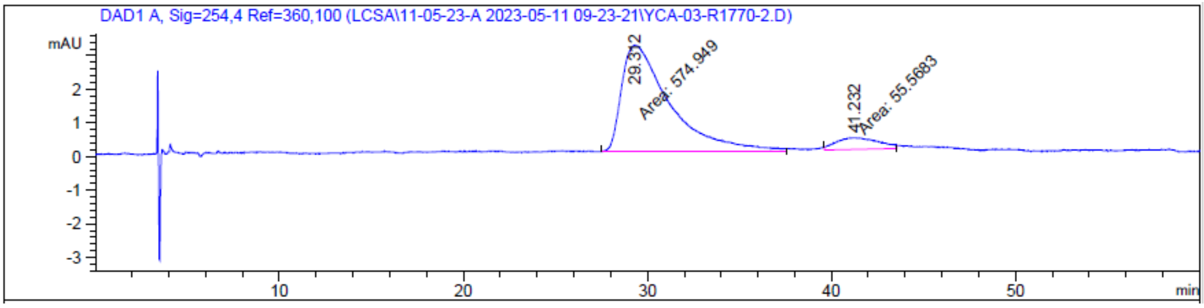


Signal 1: DAD1 A, Sig=254,4 Ref=360,100

| Peak # | RetTime [min] | Type | Width [min] | Area [mAU*s] | Height [mAU] | Area % |
|---|---|---|---|---|---|---|
| 1 | 29.312 | MM | 3.0312 | 574.94904 | 3.16131 | 91.1869 |
| 2 | 41.232 | MM | 2.6793 | 55.56827 | 3.45666e-1 | 8.8131 |

Totals : 630.51731 3.50698

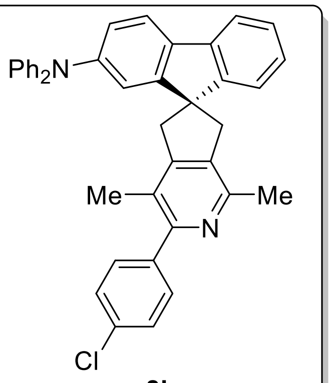

**8h**

**Name**: (*R*)-3-(4-chlorophenyl)-1,4-dimethyl-*N*,*N*-diphenyl-5,7-dihydrospiro[cyclopenta[c]pyridine-6,9'-fluoren]-2'-amine (**8h**)

**Synthesis**: Following general procedure E, (using **1d** 43.8 mg, 0.1 mmol; **6h** 13.8 mg, 0.1 mmol; **Ni3** 3.6 mg, 3.0 µmol) compound **8h** was obtained in 35.6 mg, 0.0619 mmol, 62% yield and 94:6 er as a pale-yellow thick oil.

**$^1$H NMR** (400 MHz, $CDCl_3$) δ 7.65 (d, *J* = 7.6 Hz, 1H), 7.59 (d, *J* = 8.1 Hz, 1H), 7.48 – 7.39 (m, 4H), 7.38 – 7.31 (m, 1H), 7.29 – 7.21 (m, 4H), 7.20 – 7.15 (m, 2H), 7.14 – 6.98 (m, 8H), 3.47 – 3.28 (m, 4H), 2.44 (s, 3H), 2.13 (s, 3H).

**$^{13}$C NMR** (101 MHz, $CDCl_3$) δ 155.8, 153.3, 152.7, 152.4, 150.9, 148.0, 147.9, 139.5, 139.3, 135.5, 134.4, 133.7, 130.8, 129.4, 128.4, 127.8, 127.0, 124.6, 124.4, 123.4, 123.1, 122.0, 120.7, 119.5, 117.9, 56.6, 45.0, 44.1, 22.1, 16.4.

**HRMS** (ESI/QTOF) m/z: $[M + H]^+$ Calcd for $C_{40}H_{32}ClN_2^+$ 575.2249; Found 575.2263.

$[\alpha]_D^{20}$**:** 69.7 (c = 0.22, $CHCl_3$).

**IR (ATR)**: $\tilde{\nu}$ ($cm^{-1}$) =: 3037, 2959, 2923, 2853, 1585, 1488, 1451, 1426, 1346, 1331, 1314, 1260, 1219, 1195, 1174, 1154, 1089, 1014, 970, 867, 834, 799, 753, 735, 696, 660, 623, 575, 513, 495, 454.

**Melting point**: -

**R$_f$**: 0.57 (ethyl acetate: pentane = 1:4).

**HPLC:** Chiralpak IC 4.6 x 250 mm; hexane:*i*-PrOH 98:2, 1 mL/min, 230 nm; *t*R (major) = 6.5 min, *t*R (minor) = 9.3 min, 94:6 er.

**Chromatogram**

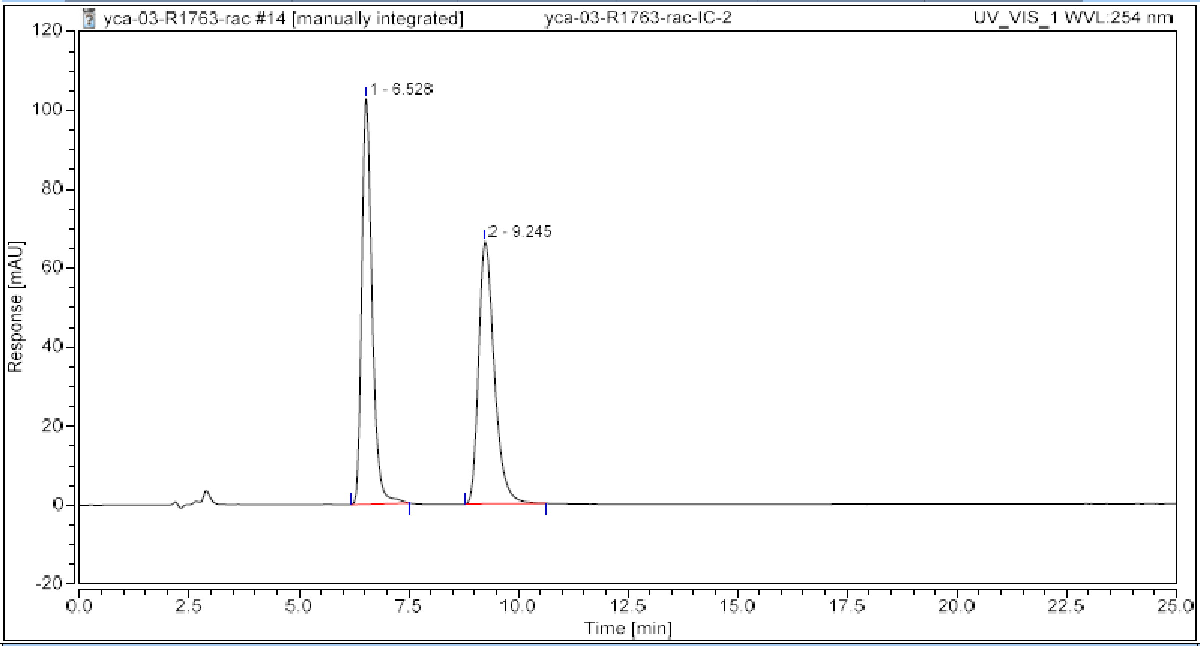


**Integration Results**

| No. | Peak Name | Retention Time min | Area mAU*min | Height mAU | Relative Area % | Relative Height % | Amount n.a. |
|---|---|---|---|---|---|---|---|
| 1 | | 6.528 | 28.043 | 102.584 | 50.40 | 60.70 | n.a. |
| 2 | | 9.245 | 27.600 | 66.414 | 49.60 | 39.30 | n.a. |
| **Total:** | | | **55.642** | **168.998** | **100.00** | **100.00** | |

**Chromatogram**

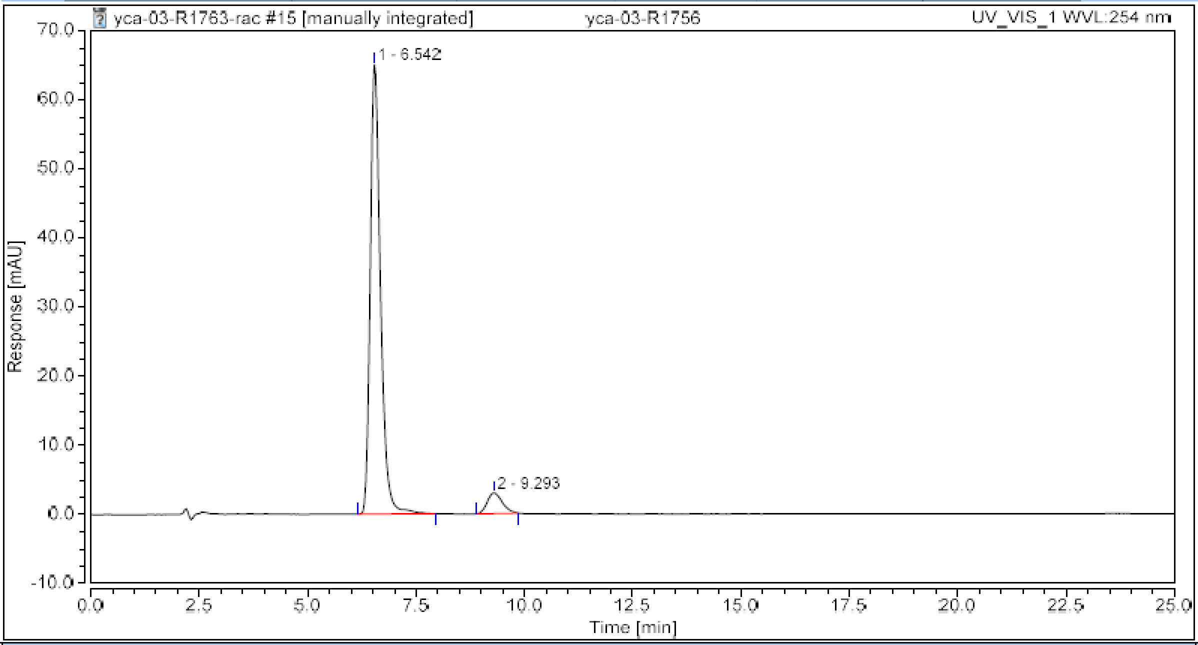


**Integration Results**

| No. | Peak Name | Retention Time min | Area mAU*min | Height mAU | Relative Area % | Relative Height % | Amount n.a. |
|---|---|---|---|---|---|---|---|
| 1 | | 6.542 | 17.884 | 65.066 | 93.75 | 95.59 | n.a. |
| 2 | | 9.293 | 1.193 | 2.999 | 6.25 | 4.41 | n.a. |
| **Total:** | | | **19.077** | **68.065** | **100.00** | **100.00** | |

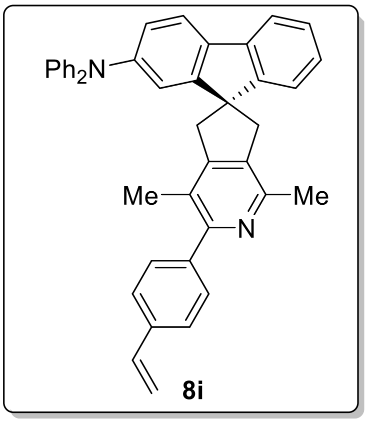

**Name**: (*R*)-1,4-dimethyl-*N*,*N*-diphenyl-3-(4-vinylphenyl)-5,7-dihydrospiro[cyclopenta[c]pyridine-6,9'-fluoren]-2'-amine (**8i**)

**Synthesis**: Following general procedure E, (using **1d** 43.8 mg, 0.1 mmol; **6i** 12.9 mg, 0.1 mmol; **Ni3** 3.6 mg, 3.0 μmol) compound **8i** was obtained in 42.5 mg, 0.0749 mmol, 75% yield and 94:6 er as a pale-yellow thick oil.

**$^1$H NMR** (400 MHz, $CDCl_3$) δ 7.65 (d, *J* = 7.5 Hz, 1H), 7.59 (d, *J* = 8.1 Hz, 1H), 7.53 – 7.43 (m, 4H), 7.37 – 7.31 (m, 1H), 7.29 – 7.21 (m, 4H), 7.20 – 7.15 (m, 2H), 7.15 – 7.05 (m, 6H), 7.01 (t, *J* = 7.3 Hz, 2H), 6.78 (dd, *J* = 17.6, 10.9 Hz, 1H), 5.81 (d, *J* = 17.5 Hz, 1H), 5.28 (d, *J* = 11.0 Hz, 1H), 3.47 – 3.26 (m, 4H), 2.45 (s, 3H), 2.16 (s, 3H).

**$^{13}$C NMR** (101 MHz, $CDCl_3$) δ 156.6, 153.3, 152.8, 152.2, 150.8, 148.0, 147.9, 140.5, 139.3, 136.9, 136.8, 135.1, 134.4, 129.6, 129.4, 127.7, 127.0, 126.1, 124.6, 124.4, 123.3, 123.1, 122.0, 120.6, 119.4, 117.9, 114.0, 56.6, 45.0, 44.1, 22.1, 16.4.

**HRMS** (ESI/QTOF) m/z: $[M + H]^+$ Calcd for $C_{42}H_{35}N_2^+$ 567.2795; Found 567.2816.

$[\alpha]_D^{20}$**:** 94.0 (c = 0.11, $CHCl_3$).

**IR (ATR)**: $\tilde{\nu}$ ($cm^{-1}$) =: 3059, 3034, 2960, 2923, 2851, 1585, 1488, 1450, 1427, 1379, 1345, 1331, 1314, 1276, 1261, 1220, 1174, 1155, 1073, 1017, 907, 847, 798, 754, 735, 696, 670, 658, 619, 596, 511, 495, 487, 475, 453, 400.

**Appearance**: pale yellow thick oil.

**Melting point**: -

**R$_f$**: 0.51 (ethyl acetate: pentane = 1:4).

**HPLC:** Chiralpak ID 4.6 x 250 mm; hexane:*i*-PrOH 98:2, 1 mL/min, 210 nm; *t*R (major) = 14.3 min, *t*R (minor) = 20.9 min, 94:6 er.

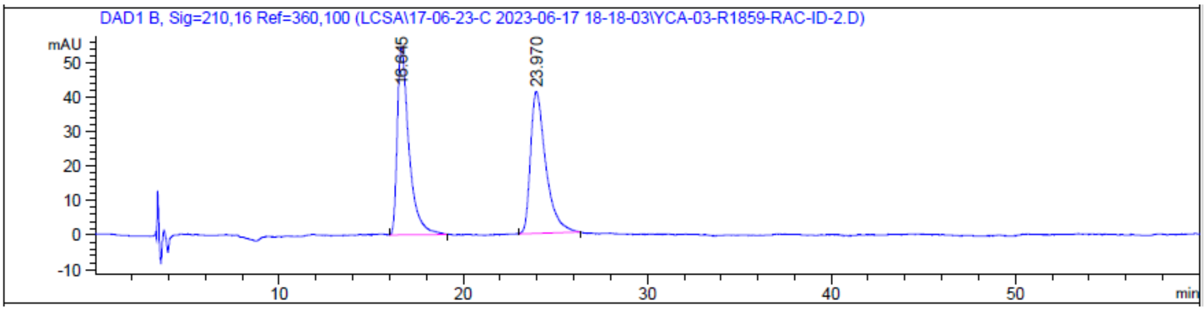


Signal 2: DAD1 B, Sig=210,16 Ref=360,100

| Peak # | RetTime [min] | Type | Width [min] | Area [mAU*s] | Height [mAU] | Area % |
|---|---|---|---|---|---|---|
| 1 | 16.645 | BB | 0.6385 | 2402.39697 | 54.47842 | 50.3397 |
| 2 | 23.970 | BB | 0.8205 | 2369.97632 | 41.11684 | 49.6603 |

Totals : 4772.37329 95.59526

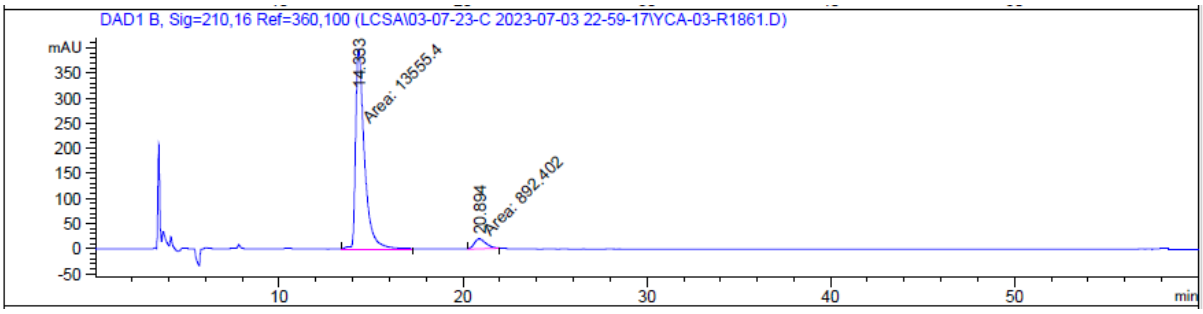


Signal 2: DAD1 B, Sig=210,16 Ref=360,100

| Peak # | RetTime [min] | Type | Width [min] | Area [mAU*s] | Height [mAU] | Area % |
|---|---|---|---|---|---|---|
| 1 | 14.333 | MM | 0.5659 | 1.35554e4 | 399.25098 | 93.8233 |
| 2 | 20.894 | MM | 0.7364 | 892.40186 | 20.19651 | 6.1767 |

Totals : 1.44478e4 419.44748

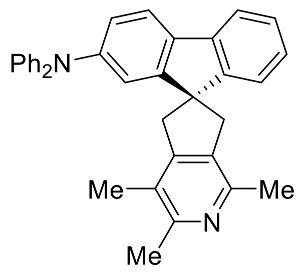

**8j**

**Name**: (*R*)-1,3,4-trimethyl-*N*,*N*-diphenyl-5,7-dihydrospiro[cyclopenta[c]pyridine-6,9'-fluoren]-2'-amine (**8j**)

**Synthesis**: Following general procedure E, (using **1d** 43.8 mg, 0.1 mmol; **6j** 4.1 mg, 0.1 mmol; **Ni3** 3.6 mg, 3.0 μmol) compound **8j** was obtained in 45.5 mg, 0.095 mmol, 95% yield and 90:10 er as a colorless thick oil.

**$^1$H NMR** (400 MHz, $CDCl_3$) δ 7.63 (d, *J* = 7.6 Hz, 1H), 7.58 (d, *J* = 8.2 Hz, 1H), 7.31 (td, *J* = 7.5, 1.3 Hz, 1H), 7.28 – 7.19 (m, 4H), 7.17 – 6.97 (m, 10H), 3.39 – 3.20 (m, 4H), 2.49 (s, 3H), 2.37 (s, 3H), 2.12 (s, 3H).

**$^{13}$C NMR** (101 MHz, $CDCl_3$) δ 154.7, 153.2, 153.1, 151.2, 149.8, 148.0, 147.9, 139.2, 134.5, 134.0, 129.4, 127.6, 127.0, 125.1, 124.4, 123.4, 123.0, 121.9, 120.6, 119.4, 118.2, 56.7, 44.8, 44.0, 22.3, 21.8, 15.3.

**HRMS** (ESI/QTOF) m/z: $[M + H]^+$ Calcd for $C_{35}H_{31}N_2^+$ 479.2482; Found 479.2504.

$[\alpha]_D^{20}$**:** 25.0 (c = 0.16, $CHCl_3$).

**IR (ATR)**: $\tilde{\nu}$ ($cm^{-1}$) =: 3035, 2961, 2920, 2852, 1584, 1486, 1467, 1449, 1426, 1377, 1346, 1330, 1313, 1261, 1219, 1201, 1174, 1154, 1075, 1018, 870, 798, 753, 735, 695, 670, 659, 513, 455.

**Melting point**: -

**$R_f$**: 0.38 (ethyl acetate: pentane = 1:1).

**HPLC:** Chiralpak ID 4.6 x 250 mm; hexane:*i*-PrOH 99:1, 1 mL/min, 254 nm; *t*R (major) = 13.2 min, *t*R (minor) = 15.8 min, 90:10 er.

**Chromatogram**

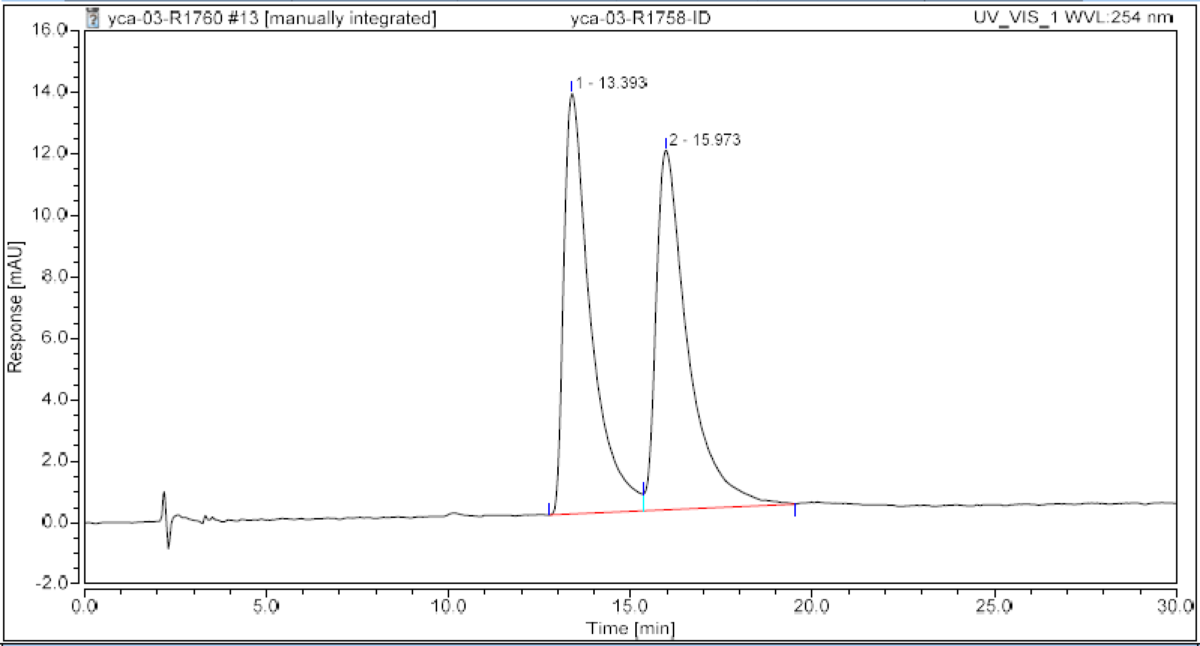


**Integration Results**

| No. | Peak Name | Retention Time min | Area mAU*min | Height mAU | Relative Area % | Relative Height % | Amount n.a. |
|---|---|---|---|---|---|---|---|
| 1 | | 13.393 | 11.756 | 13.694 | 49.69 | 53.90 | n.a. |
| 2 | | 15.973 | 11.904 | 11.715 | 50.31 | 46.10 | n.a. |
| **Total:** | | | **23.661** | **25.409** | **100.00** | **100.00** | |

**Chromatogram**

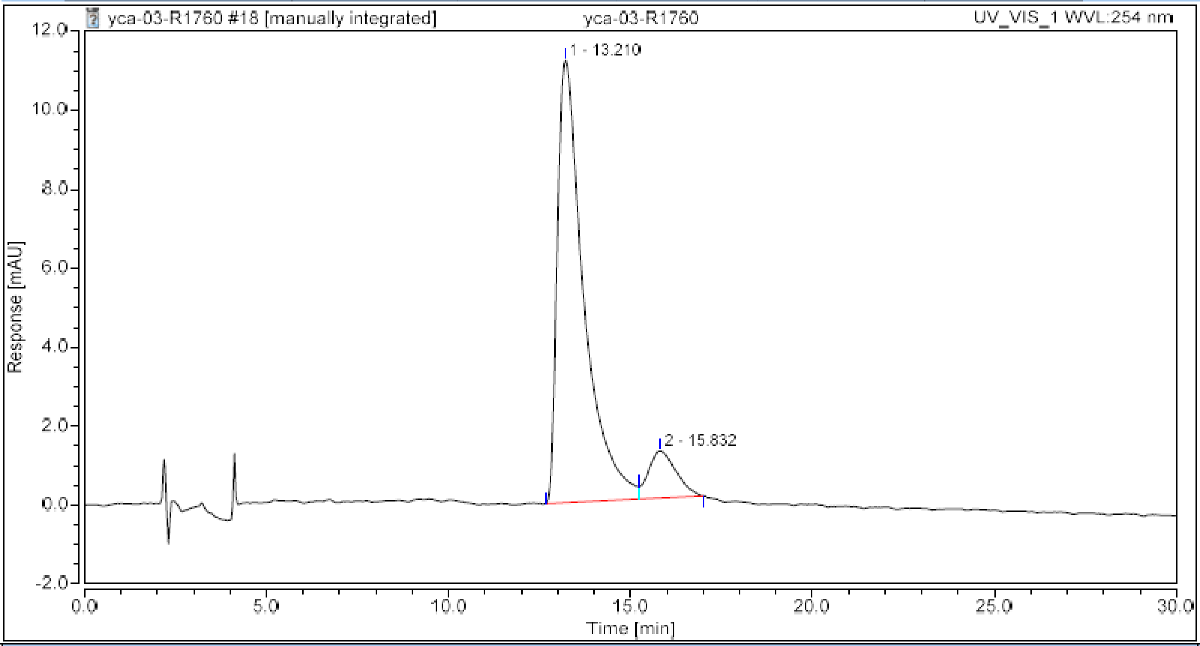


**Integration Results**

| No. | Peak Name | Retention Time min | Area mAU*min | Height mAU | Relative Area % | Relative Height % | Amount n.a. |
|---|---|---|---|---|---|---|---|
| 1 | | 13.210 | 9.453 | 11.217 | 90.00 | 90.39 | n.a. |
| 2 | | 15.832 | 1.051 | 1.192 | 10.00 | 9.61 | n.a. |
| **Total:** | | | **10.503** | **12.409** | **100.00** | **100.00** | |

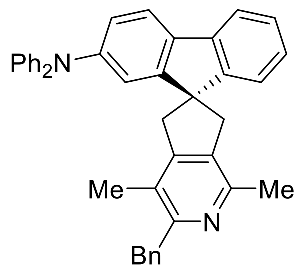


**8k**

**Name**: (*R*)-3-benzyl-1,4-dimethyl-*N*,*N*-diphenyl-5,7-dihydrospiro[cyclopenta[c]pyridine-6,9'-fluoren]-2'-amine (**8k**).

**Synthesis**: Following general procedure E, (using **1d** 43.8 mg, 0.1 mmol; **6k** 11.7 mg, 0.1 mmol; **Ni3** 3.6 mg, 3.0 μmol) compound **8k** was obtained in 50.0 mg, 0.0901 mmol, 90% yield and 96:4 er as a pale-yellow thick oil.

**$^{1}$H NMR** (400 MHz, $CDCl_3$) δ 7.63 (d, *J* = 7.4 Hz, 1H), 7.58 (d, *J* = 9.1 Hz, 1H), 7.35 – 7.16 (m, 10H), 7.16 – 6.97 (m, 10H), 4.21 (s, 2H), 3.43 – 3.17 (m, 4H), 2.42 (s, 3H), 2.05 (s, 3H).

**$^{13}$C NMR** (101 MHz, $CDCl_3$) δ 156.5, 153.2, 153.0, 151.9, 150.2, 148.0, 147.9, 139.8, 139.2, 134.6, 134.4, 129.4, 128.6, 128.4, 127.7, 127.0, 126.0, 125.6, 124.3, 123.4, 123.0, 121.9, 120.6, 119.4, 118.1, 56.6, 44.8, 44.1, 42.1, 22.0, 15.1.

**HRMS** (ESI/QTOF) m/z: $[M + H]^+$ Calcd for $C_{38}H_{37}N_2^+$ 521.2951; Found 521.2975.

$[\boldsymbol{\alpha}]_D^{20}$**:** 34.7 (c = 0.25, $CHCl_3$).

**IR (ATR)**: $\tilde{\nu}$ ($cm^{-1}$) =: 3031, 2922, 1584, 1486, 1468, 1449, 1426, 1330, 1313, 1263, 1220, 1174, 1155, 1074, 1028, 822, 802, 778, 753, 732, 695, 658, 619, 608, 588, 566, 512, 455.

**Melting point**: -

**R*f***: 0.35 (ethyl acetate: pentane = 1:4).

**HPLC:** Chiralpak IA 4.6 x 250 mm; hexane:*i*-PrOH 99:1, 1 mL/min, 254 nm; *t*R (major) = 13.6 min, *t*R (minor) = 10.9 min, 96:4 er.

## Chromatogram

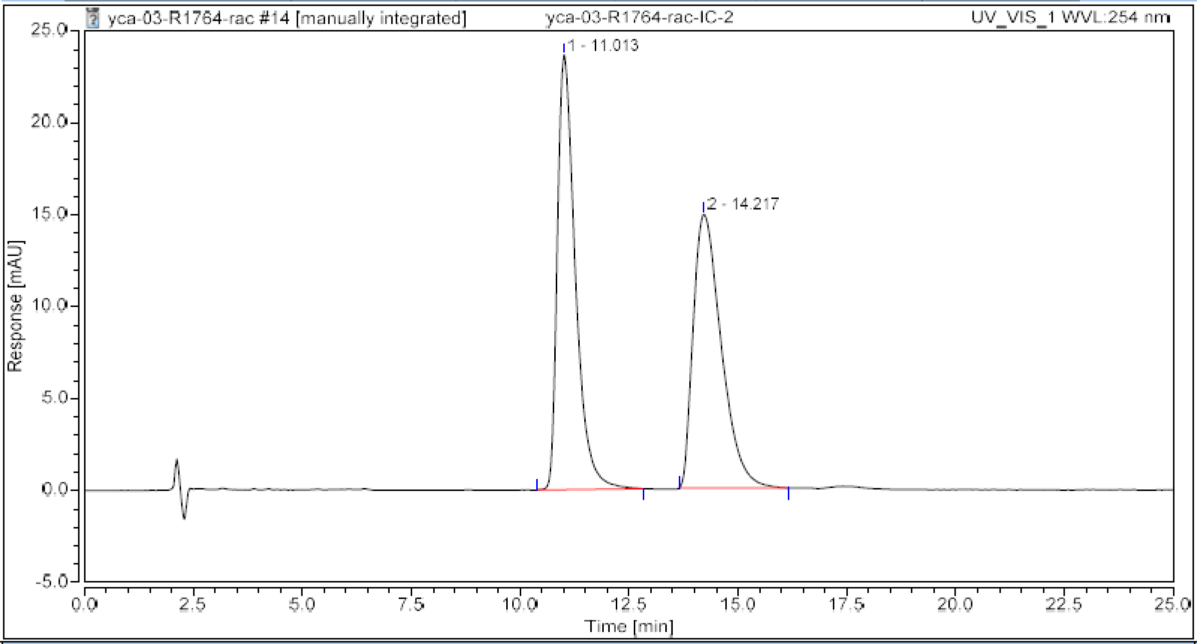


## Integration Results

| No. | Peak Name | Retention Time<br>min | Area<br>mAU*min | Height<br>mAU | Relative Area<br>% | Relative Height<br>% | Amount<br>n.a. |
|---|---|---|---|---|---|---|---|
| 1 | | 11.013 | 11.306 | 23.661 | 50.41 | 61.35 | n.a. |
| 2 | | 14.217 | 11.124 | 14.905 | 49.59 | 38.65 | n.a. |
| **Total:** | | | **22.430** | **38.566** | **100.00** | **100.00** | |

## Chromatogram

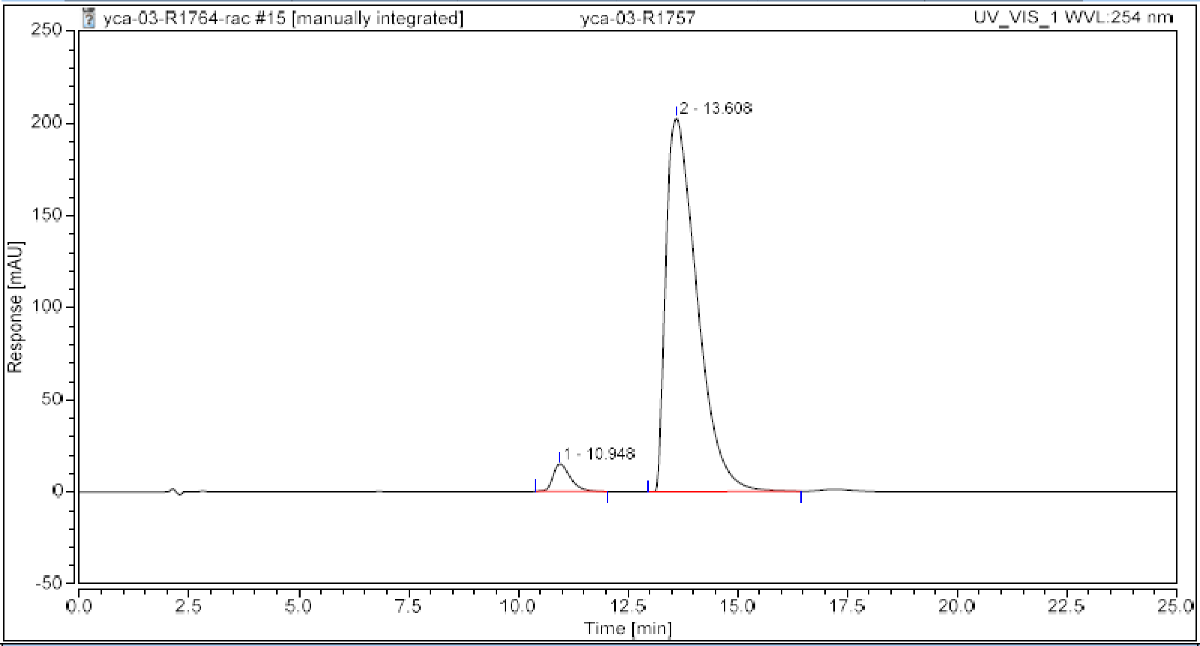


## Integration Results

| No. | Peak Name | Retention Time<br>min | Area<br>mAU*min | Height<br>mAU | Relative Area<br>% | Relative Height<br>% | Amount<br>n.a. |
|---|---|---|---|---|---|---|---|
| 1 | | 10.948 | 6.813 | 14.934 | 3.92 | 6.87 | n.a. |
| 2 | | 13.608 | 166.784 | 202.568 | 96.08 | 93.13 | n.a. |
| **Total:** | | | **173.597** | **217.502** | **100.00** | **100.00** | |

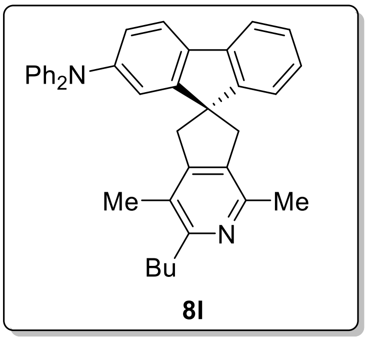


**Name**: (*R*)-3-butyl-1,4-dimethyl-*N*,*N*-diphenyl-5,7-dihydrospiro[cyclopenta[c]pyridine-6,9'-fluoren]-2'-amine (**8l**).

**Synthesis**: Following general procedure E, (using **1d** 43.8 mg, 0.1 mmol; **6l** 8.3 mg, 0.1 mmol; **Ni3** 3.6 mg, 3.0 μmol) compound **8l** was obtained in 48.1 mg, 0.0923 mmol, 92% yield and 97:3 er as a pale-yellow thick oil.

**$^{1}$H NMR** (400 MHz, $CD_2Cl_2$) δ 7.64 (d, *J* = 7.6 Hz, 1H), 7.59 (d, *J* = 8.1 Hz, 1H), 7.36 – 7.28 (m, 1H), 7.27 – 7.20 (m, 4H), 7.17 – 7.11 (m, 2H), 7.11 – 6.97 (m, 8H), 3.35 – 3.21 (m, 4H), 2.80 – 2.67 (m, 2H), 2.32 (s, 3H), 2.14 (s, 3H), 1.60 (p, *J* = 7.5 Hz, 2H), 1.43 (h, *J* = 7.3 Hz, 2H), 0.98 (t, *J* = 7.3 Hz, 3H).

**$^{13}$C NMR** (101 MHz, $CD_2Cl_2$) δ 158.8, 154.0, 153.2, 151.5, 150.1, 148.2, 148.2, 139.6, 134.6, 133.8, 129.6, 127.8, 127.2, 124.6, 124.6, 123.5, 123.2, 122.4, 120.8, 119.5, 118.3, 57.0, 45.1, 44.2, 35.4, 32.1, 23.4, 21.9, 14.9, 14.3.

**HRMS** (ESI/QTOF) m/z: $[M + H]^+$ Calcd for $C_{41}H_{35}N_2^+$ 555.2795; Found 555.2804.

$[\alpha]_D^{20}$**:** 17.7 (c = 0.16, $CHCl_3$).

**IR (ATR)**: $\tilde{\nu}$ ($cm^{-1}$) =: 3035, 2954, 2925, 2856, 1584, 1486, 1449, 1427, 1377, 1330, 1313, 1274, 1262, 1220, 1174, 1154, 1075, 1026, 875, 802, 779, 753, 735, 695, 658, 513, 455.

**Melting point**: -

**$R_f$**: 0.39 (ethyl acetate: pentane = 1:4).

**HPLC:** Chiralpak IA 4.6 x 250 mm; hexane:*i*-PrOH 99:1, 1 mL/min, 254 nm; *t*R (major) = 6.0 min, *t*R (minor) = 5.5 min, 97:3 er.

**Chromatogram**

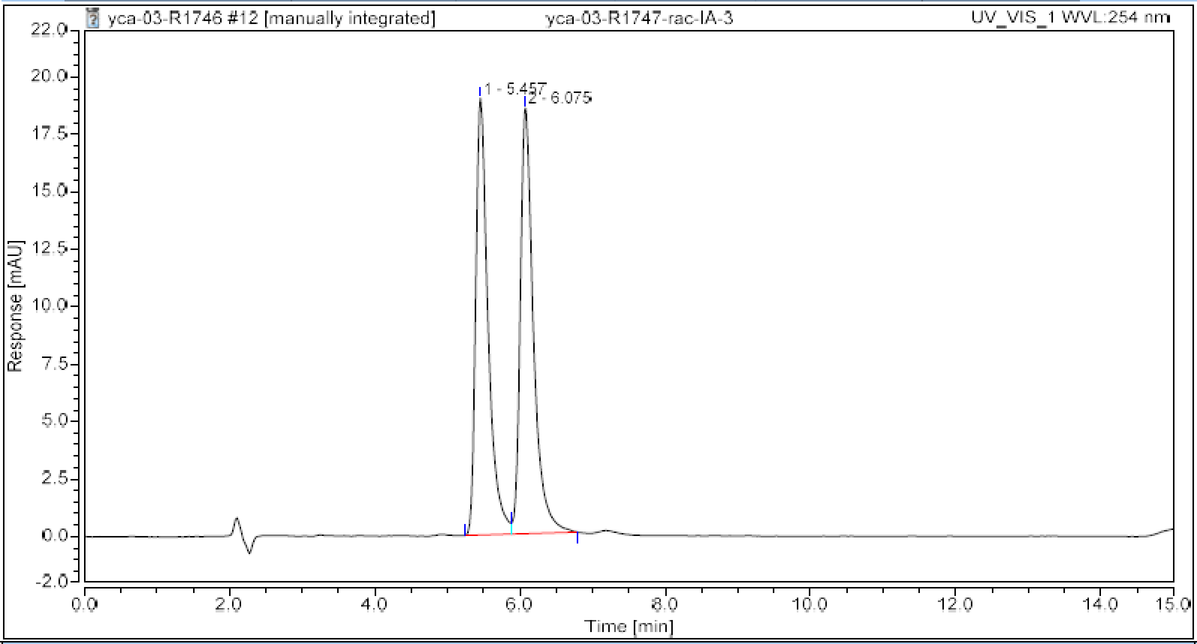


**Integration Results**

| No. | Peak Name | Retention Time min | Area mAU*min | Height mAU | Relative Area % | Relative Height % | Amount n.a. |
|---|---|---|---|---|---|---|---|
| 1 | | 5.457 | 3.744 | 19.015 | 49.12 | 50.63 | n.a. |
| 2 | | 6.075 | 3.879 | 18.542 | 50.88 | 49.37 | n.a. |
| **Total:** | | | **7.623** | **37.557** | **100.00** | **100.00** | |

**Chromatogram**

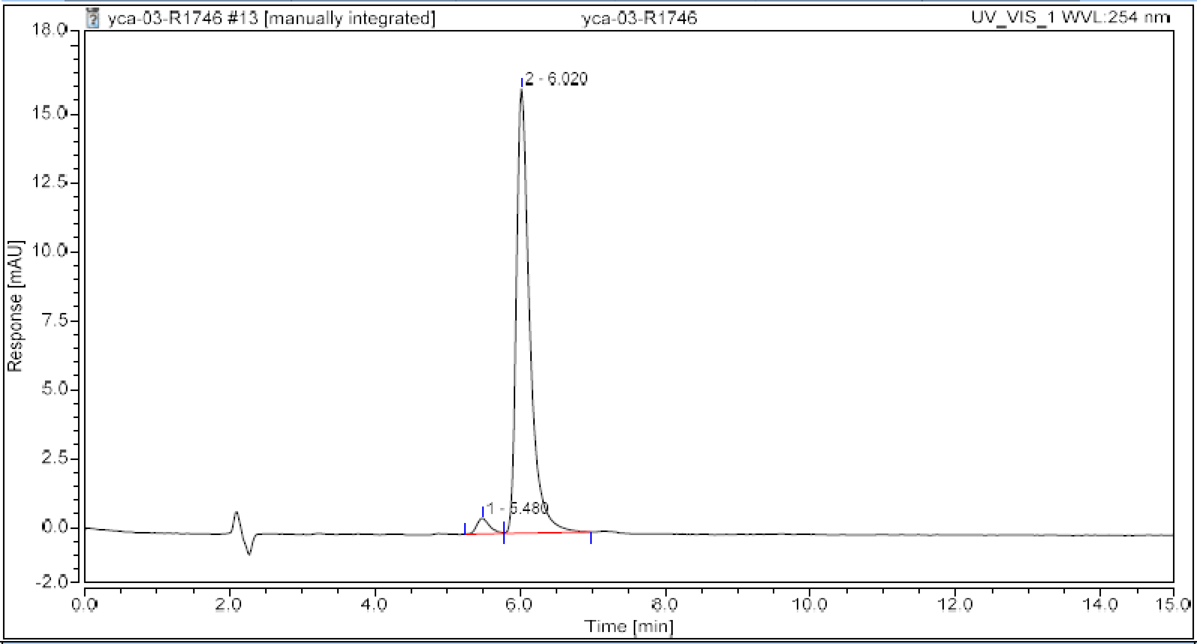


**Integration Results**

| No. | Peak Name | Retention Time min | Area mAU*min | Height mAU | Relative Area % | Relative Height % | Amount n.a. |
|---|---|---|---|---|---|---|---|
| 1 | | 5.480 | 0.116 | 0.569 | 3.25 | 3.41 | n.a. |
| 2 | | 6.020 | 3.448 | 16.100 | 96.75 | 96.59 | n.a. |
| **Total:** | | | **3.563** | **16.669** | **100.00** | **100.00** | |

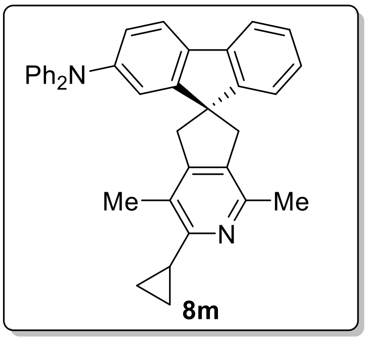

**Name**: (*R*)-3-cyclopropyl-1,4-dimethyl-*N*,*N*-diphenyl-5,7-dihydrospiro[cyclopenta[c]pyridine-6,9'-fluoren]-2'-amine (**8m**).

**Synthesis**: Following general procedure E, (using **1d** 43.8 mg, 0.1 mmol; **6m** 6.7 mg, 0.1 mmol; **Ni3** 3.6 mg, 3.0 μmol) compound **8m** was obtained in 48.6 mg, 0.0963 mmol, 96% yield and 94:6 er as a pale-yellow oil.

**$^1$H NMR** (400 MHz, $CDCl_3$) δ 7.63 (d, *J* = 7.5 Hz, 1H), 7.58 (d, *J* = 8.1 Hz, 1H), 7.31 (td, *J* = 7.4, 1.4 Hz, 1H), 7.27 – 7.20 (m, 4H), 7.15 – 6.97 (m, 10H), 3.37 – 3.16 (m, 4H), 2.31 (s, 3H), 2.25 (s, 3H), 2.12 – 2.02 (m, 1H), 1.18 – 1.09 (m, 1H), 0.99 – 0.84 (m, 3H).

**$^{13}$C NMR** (101 MHz, $CDCl_3$) δ 157.8, 153.3, 150.7, 149.9, 147.9, 139.2, 134.5, 133.1, 129.4, 127.6, 127.0, 125.0, 124.4, 123.3, 123.0, 122.0, 120.6, 119.3, 118.2, 56.6, 44.7, 44.0, 22.0, 14.7, 13.8, 8.5, 7.8.

**HRMS** (ESI/QTOF) m/z: $[M + H]^+$ Calcd for $C_{37}H_{33}N_2^+$ 505.2638; Found 505.2653.

$[\boldsymbol{\alpha}]_D^{20}$**:** 6.0 (c = 0.64, $CHCl_3$).

**IR (ATR)**: $\tilde{\nu}$ ($cm^{-1}$) =: 2957, 1724, 1585, 1487, 1450, 1430, 1313, 1275, 1260, 1220, 1179, 1092, 1035, 920, 870, 802, 780, 754, 737, 697, 662, 515.

**Melting point**: -

**$R_f$**: 0.81 (ethyl acetate: pentane = 1:4).

**HPLC:** Chiralpak ID 4.6 x 250 mm; hexane:*i*-PrOH 95:5, 1 mL/min, 210 nm; *t*R (major) = 4.9 min, *t*R (minor) = 5.4 min, 94:6 er.

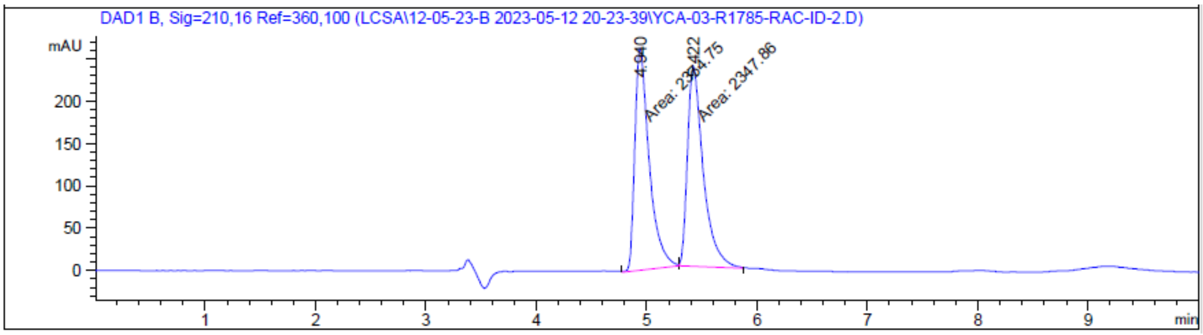


Signal 2: DAD1 B, Sig=210,16 Ref=360,100

| Peak # | RetTime [min] | Type | Width [min] | Area [mAU*s] | Height [mAU] | Area % |
|---|---|---|---|---|---|---|
| 1 | 4.940 | MM | 0.1509 | 2374.74561 | 262.36523 | 50.2847 |
| 2 | 5.422 | MM | 0.1661 | 2347.85571 | 235.62570 | 49.7153 |

Totals : 4722.60132 497.99094

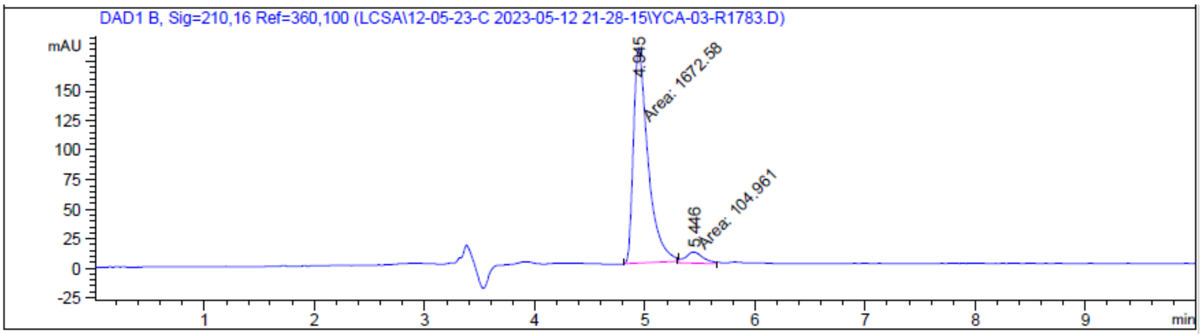


Signal 2: DAD1 B, Sig=210,16 Ref=360,100

| Peak # | RetTime [min] | Type | Width [min] | Area [mAU*s] | Height [mAU] | Area % |
|---|---|---|---|---|---|---|
| 1 | 4.945 | MM | 0.1535 | 1672.57922 | 181.60103 | 94.0952 |
| 2 | 5.446 | MM | 0.1862 | 104.96093 | 9.39656 | 5.9048 |

Totals : 1777.54015 190.99759

**8m** was crystallized in $CHCl_3$

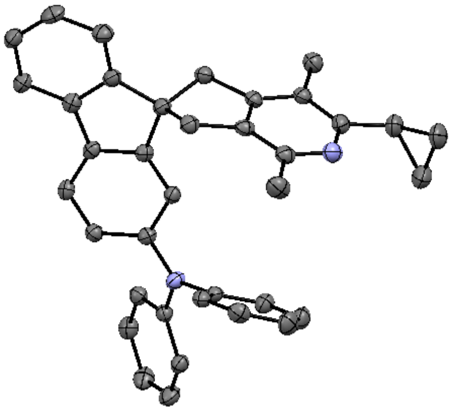

**Supplementary Fig. 7.** ORTEP diagram of **8m** (thermal ellipsoids are shown at the 50% probability level), for the reason of clarity all the hydrogen atoms are omitted.

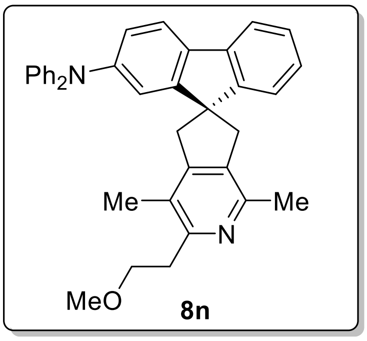

**8n**

**Name**: (*R*)-3-(2-methoxyethyl)-1,4-dimethyl-*N*,*N*-diphenyl-5,7-dihydrospiro[cyclopenta[c]pyridine-6,9'-fluoren]-2'-amine (**8n**).

**Synthesis**: Following general procedure E, (using **1d** 43.8 mg, 0.1 mmol; **6n** 8.5 mg, 0.1 mmol; **Ni3** 3.6 mg, 3.0 μmol) compound **8n** was obtained in 44.3 mg, 0.0847 mmol, 85% yield and 96:4 er as a pale-yellow thick oil.

**$^1$H NMR** (400 MHz, $CDCl_3$) δ 7.63 (d, *J* = 7.5 Hz, 1H), 7.57 (d, *J* = 8.3 Hz, 1H), 7.32 (td, *J* = 7.4, 1.3 Hz, 1H), 7.28 – 7.20 (m, 4H), 7.17 – 6.97 (m, 10H), 3.80 – 3.65 (m, 2H), 3.38 (s, 3H), 3.35 – 3.22 (m, 4H), 3.08 (t, *J* = 7.1 Hz, 2H), 2.37 (s, 3H), 2.17 (s, 3H).

**$^{13}$C NMR** (101 MHz, $CDCl_3$) δ 154.9, 153.3, 153.0, 151.6, 150.2, 148.0, 147.9, 139.2, 134.4, 129.4, 127.7, 127.0, 125.5, 124.4, 123.3, 123.0, 121.9, 120.6, 119.4, 118.0, 72.4, 58.9, 56.6, 44.9, 44.1, 35.4, 21.8, 15.0.

**HRMS** (ESI/QTOF) m/z: $[M + H]^+$ Calcd for $C_{37}H_{35}N_2O^+$ 523.2744; Found 523.2762.

**$[\alpha]_D^{20}$:** 26.8 (c = 0.18, $CHCl_3$).

**IR (ATR)**: $\tilde{\nu}$ ($cm^{-1}$) =: 3035, 2922, 2892, 1584, 1486, 1449, 1427, 1380, 1345, 1330, 1313, 1264, 1220, 1198, 1176, 1155, 1111, 1075, 1027, 964, 938, 924, 895, 873, 822, 803, 779, 753, 735, 695, 658, 618, 569, 512, 454.

**Melting point**: -

**$R_f$**: 0.26 (ethyl acetate: pentane = 1:1).

**HPLC:** Chiralpak IF 4.6 x 250 mm; hexane:*i*-PrOH 95:5, 1 mL/min, 210 nm; *t*R (major) = 10.0 min, *t*R (minor) = 11.7 min, 96:4 er.

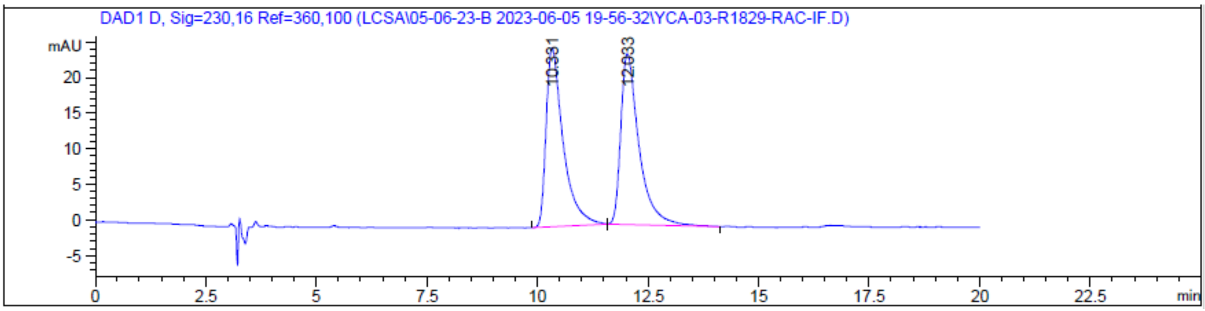


Signal 3: DAD1 D, Sig=230,16 Ref=360,100

| Peak # | RetTime [min] | Type | Width [min] | Area [mAU*s] | Height [mAU] | Area % |
|---|---|---|---|---|---|---|
| 1 | 10.331 | BB | 0.3897 | 671.90533 | 25.14987 | 49.4837 |
| 2 | 12.033 | BB | 0.4216 | 685.92548 | 23.98938 | 50.5163 |

Totals : 1357.83081 49.13925

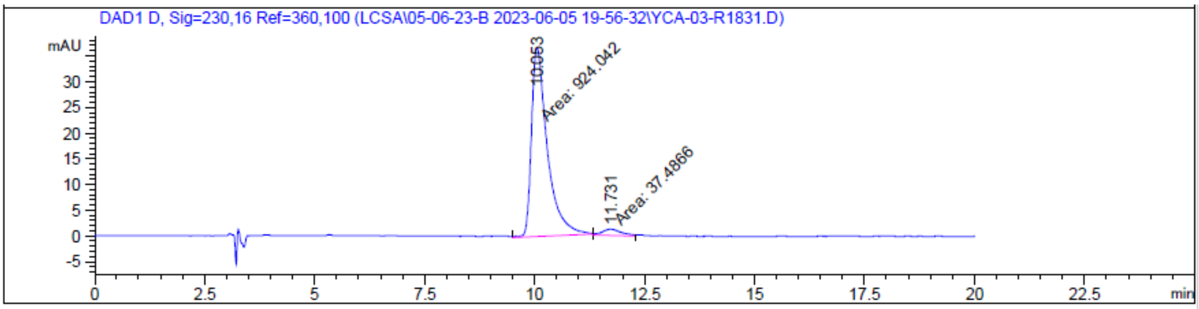


Signal 3: DAD1 D, Sig=230,16 Ref=360,100

| Peak # | RetTime [min] | Type | Width [min] | Area [mAU*s] | Height [mAU] | Area % |
|---|---|---|---|---|---|---|
| 1 | 10.053 | MM | 0.4189 | 924.04169 | 36.76600 | 96.1014 |
| 2 | 11.731 | MM | 0.5129 | 37.48661 | 1.21818 | 3.8986 |

Totals : 961.52829 37.98417

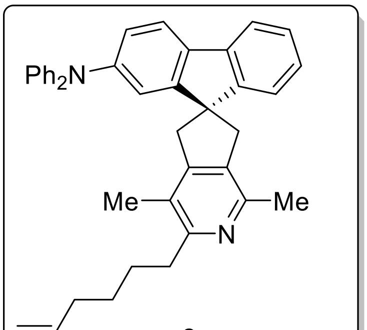

**8o**

**Name**: (*R*)-3-(hex-5-en-1-yl)-1,4-dimethyl-*N*,*N*-diphenyl-5,7-dihydrospiro[cyclopenta[c]pyridine-6,9'-fluoren]-2'-amine (**8o**).

**Synthesis**: Following general procedure E, (using **1d** 43.8 mg, 0.1 mmol; **6o** 10.9 mg, 0.1 mmol; **Ni3** 3.6 mg, 3.0 µmol) compound **8o** was obtained in 48.3 mg, 0.0883 mmol, 88% yield and 98:2 er as apale yellow thick oil.

**$^{1}$H NMR** (400 MHz, $CDCl_3$) δ 7.63 (d, *J* = 7.6 Hz, 1H), 7.57 (d, *J* = 8.1 Hz, 1H), 7.31 (t, *J* = 7.9 Hz, 1H), 7.27 – 7.20 (m, 5H), 7.17 – 6.95 (m, 10H), 5.96 – 5.74 (m, 1H), 5.17 – 4.83 (m, 2H), 3.42 – 3.18 (m, 4H), 2.87 – 2.71 (m, 2H), 2.36 (s, 3H), 2.21 – 2.05 (m, 5H), 1.67 – 1.61 (m, 2H), 1.59 – 1.45 (m, 2H).

**$^{13}$C NMR** (101 MHz, $CDCl_3$) δ 158.5, 153.3, 153.1, 151.5, 150.1, 148.0, 147.9, 139.2, 139.2, 134.4, 133.8, 129.4, 127.6, 127.0, 124.4, 124.4, 123.3, 123.0, 121.9, 120.6, 119.4, 118.1, 114.5, 56.6, 44.9, 44.1, 35.6, 33.9, 29.4, 29.3, 21.9, 14.9.

**HRMS** (ESI/QTOF) m/z: $[M + H]^+$ Calcd for $C_{40}H_{39}N_2^+$ 547.3108; Found 547.3123.

$[\alpha]_D^{20}$**:** 35.2 (c = 0.14, $CHCl_3$).

**IR (ATR)**: $\tilde{\nu}$ ($cm^{-1}$) =: 3036, 2925, 2855, 1584, 1487, 1450, 1428, 1345, 1330, 1313, 1263, 1220, 1174, 1154, 1075, 1026, 910, 875, 800, 779, 753, 734, 695, 658, 567, 512, 455, 409.

**Melting point**: -

**$R_f$**: 0.43 (ethyl acetate: pentane = 1:4).

**HPLC:** Chiralpak IG 4.6 x 250 mm; hexane:*i*-PrOH 98:2, 1 mL/min, 210 nm; *t*R (major) = 9.6 min, *t*R (minor) = 11.5 min, 98:2 er.

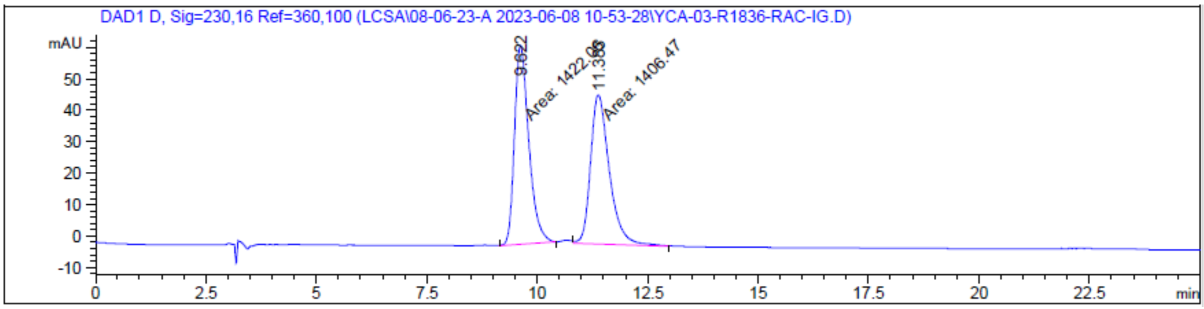


Signal 3: DAD1 D, Sig=230,16 Ref=360,100

| Peak # | RetTime [min] | Type | Width [min] | Area [mAU*s] | Height [mAU] | Area % |
|---|---|---|---|---|---|---|
| 1 | 9.622 | MM | 0.3755 | 1422.06299 | 63.12491 | 50.2756 |
| 2 | 11.383 | MM | 0.4943 | 1406.47351 | 47.42529 | 49.7244 |

Totals : 2828.53650 110.55021

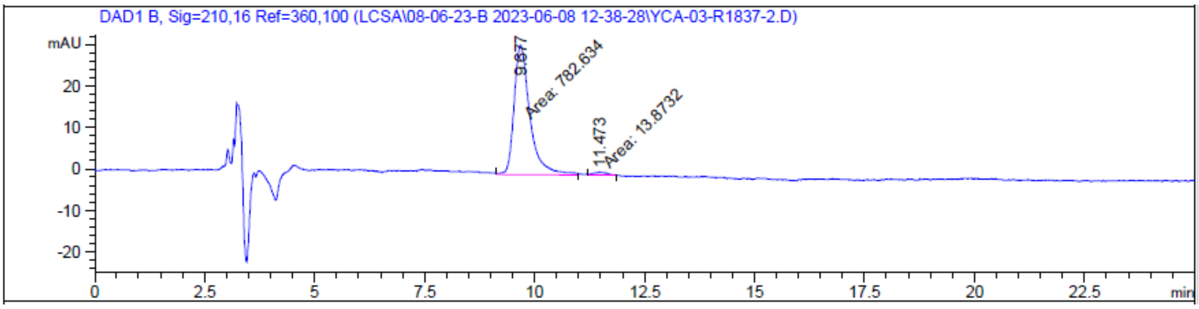


Signal 2: DAD1 B, Sig=210,16 Ref=360,100

| Peak # | RetTime [min] | Type | Width [min] | Area [mAU*s] | Height [mAU] | Area % |
|---|---|---|---|---|---|---|
| 1 | 9.677 | MM | 0.4183 | 782.63354 | 31.18027 | 98.2582 |
| 2 | 11.473 | MM | 0.3149 | 13.87318 | 7.34351e-1 | 1.7418 |

Totals : 796.50672 31.91462

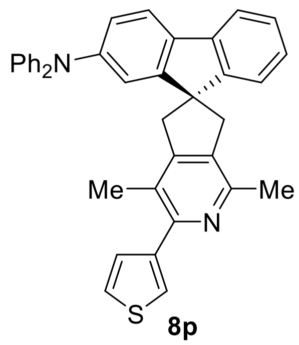

**8p**

**Name**: (*R*)-1,4-dimethyl-*N*,*N*-diphenyl-3-(thiophen-3-yl)-5,7-dihydrospiro[cyclopenta[c]pyridine-6,9'-fluoren]-2'-amine (**8p**).

**Synthesis**: Following general procedure E, (using **1d** 43.8 mg, 0.1 mmol; **6p** 10.9 mg, 0.1 mmol; **Ni3** 3.6 mg, 3.0 μmol) compound **8p** was obtained in 49.2 mg, 0.0899 mmol, 90% yield and 98.5:1.5 er as colorless thick oil.

**$^{1}$H NMR** (400 MHz, $CDCl_3$) δ 7.65 (d, *J* = 7.5 Hz, 1H), 7.62 – 7.56 (m, 1H), 7.50 – 7.44 (m, 1H), 7.42 – 7.30 (m, 3H), 7.28 – 7.20 (m, 4H), 7.19 – 7.06 (m, 8H), 7.00 (t, *J* = 7.3 Hz, 2H), 3.45 – 3.28 (m, 4H), 2.44 (s, 3H), 2.23 (s, 3H).

**$^{13}$C NMR** (101 MHz, $CDCl_3$) δ 153.2, 153.0, 152.2, 152.1, 150.8, 148.0, 147.9, 142.0, 139.2, 135.0, 134.4, 129.4, 129.2, 127.7, 127.0, 125.0, 124.8, 124.5, 124.4, 123.4, 123.0, 121.9, 120.7, 119.4, 118.0, 56.6, 45.1, 44.1, 22.1, 16.5.

**HRMS** (ESI/QTOF) m/z: $[M + H]^+$ Calcd for $C_{38}H_{31}N_2S^+$ 547.2202; Found 547.2219.

$[\alpha]_D^{20}$**:** 76.8 (c = 0.24, $CHCl_3$).

**IR (ATR)**: $\tilde{\nu}$ ($cm^{-1}$) =: 3035, 2961, 2922, 1582, 1485, 1467, 1449, 1426, 1346, 1330, 1312, 1262, 1218, 1174, 1154, 1073, 1026, 895, 863, 822, 795, 779, 733, 695, 657, 618, 572, 514, 454.

**Melting point**: -

**$R_f$**: 0.46 (ethyl acetate: pentane = 1:4).

**HPLC:** Chiralpak IA 4.6 x 250 mm; hexane:*i*-PrOH 99:1, 1 mL/min, 254 nm; *t*R (major) = 11.6 min, *t*R (minor) = 10.5 min, 98:2 er.

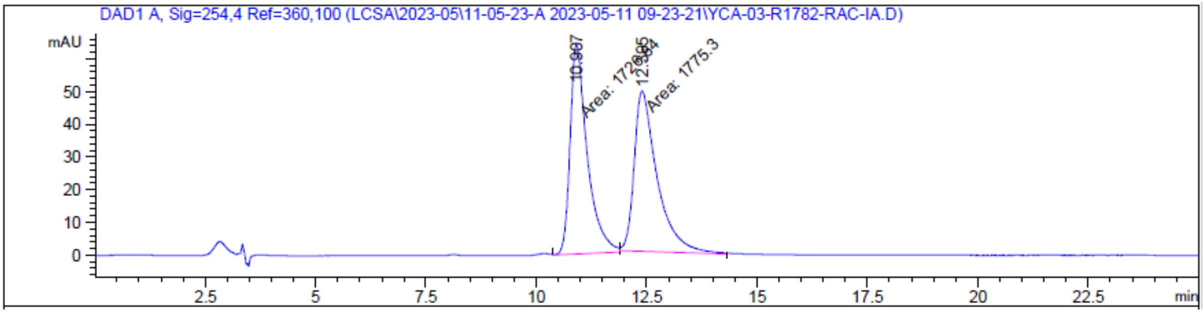


Signal 1: DAD1 A, Sig=254,4 Ref=360,100

| Peak # | RetTime [min] | Type | Width [min] | Area [mAU*s] | Height [mAU] | Area % |
|---|---|---|---|---|---|---|
| 1 | 10.907 | MM | 0.4499 | 1726.63623 | 63.96808 | 49.3051 |
| 2 | 12.395 | MM | 0.6049 | 1775.30420 | 48.91809 | 50.6949 |

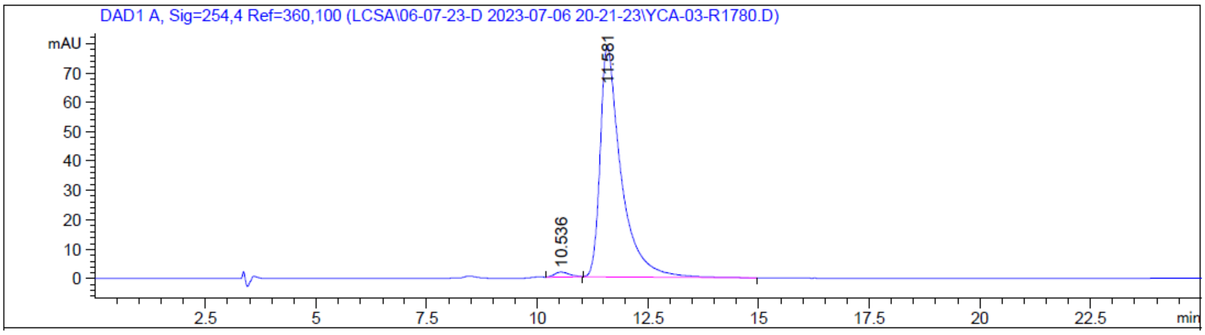


Signal 1: DAD1 A, Sig=254,4 Ref=360,100

| Peak # | RetTime [min] | Type | Width [min] | Area [mAU*s] | Height [mAU] | Area % |
|---|---|---|---|---|---|---|
| 1 | 10.536 | BB | 0.3264 | 37.45065 | 1.67809 | 1.4316 |
| 2 | 11.581 | BB | 0.4729 | 2578.50000 | 78.55930 | 98.5684 |
| Totals : | | | | 2615.95065 | 80.23738 | |

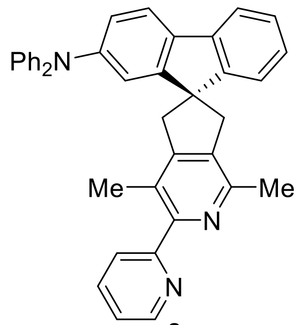

**8q**

**Name**: (*R*)-1,4-dimethyl-*N*,*N*-diphenyl-3-(pyridin-2-yl)-5,7-dihydrospiro[cyclopenta[c]pyridine-6,9'-fluoren]-2'-amine (**8q**).

**Synthesis**: Following general procedure E, (using **1d** 43.8 mg, 0.1 mmol; **6q** 10.4 mg, 0.1 mmol; **Ni3** 3.6 mg, 3.0 μmol) compound **8q** was obtained in 53.8 mg, 0.0993 mmol, 99% yield and 89:11 er as a pale-yellow foam.

**$^{1}$H NMR** (400 MHz, $CDCl_3$) δ 8.70 (d, *J* = 4.8 Hz, 1H), 7.86 – 7.78 (m, 1H), 7.72 (d, *J* = 7.8 Hz, 1H), 7.68 – 7.56 (m, 2H), 7.40 – 7.20 (m, 6H), 7.20 – 7.05 (m, 8H), 7.05 – 6.95 (m, 2H), 3.52 – 3.29 (m, 4H), 2.47 (s, 3H), 2.28 (s, 3H).

**$^{13}$C NMR** (101 MHz, $CDCl_3$) δ 159.4, 154.8, 153.2, 153.0, 152.7, 150.7, 148.7, 148.0, 147.9, 139.0, 136.6, 136.2, 134.6, 129.4, 127.7, 127.1, 125.9, 124.6, 124.3, 123.5, 123.0, 122.5, 122.0, 120.7, 119.4, 118.2, 56.5, 45.0, 44.2, 22.0, 16.0.

**HRMS** (ESI/QTOF) m/z: $[M + H]^+$ Calcd for $C_{39}H_{32}N_3^+$ 542.2591; Found 542.2604.

$[\alpha]_D^{20}$**:** 43.9 (c = 0.19, $CHCl_3$).

**IR (ATR)**: $\tilde{\nu}$ ($cm^{-1}$) =: 3037, 2918, 2849, 1584, 1563, 1487, 1468, 1450, 1426, 1410, 1378, 1346, 1331, 1314, 1275, 1264, 1220, 1173, 1154, 1092, 1075, 1027, 997, 978, 801, 779, 736, 697, 661, 618, 516.

**Melting point**: -

**$R_f$**: 0.19 (ethyl acetate: pentane = 1:1).

**HPLC:** Chiralpak IC 4.6 x 250 mm; hexane:*i*-PrOH 95:5, 1 mL/min, 254 nm; *t*R (major) = 48.6 min, *t*R (minor) = 29.3 min, 89:11 er.

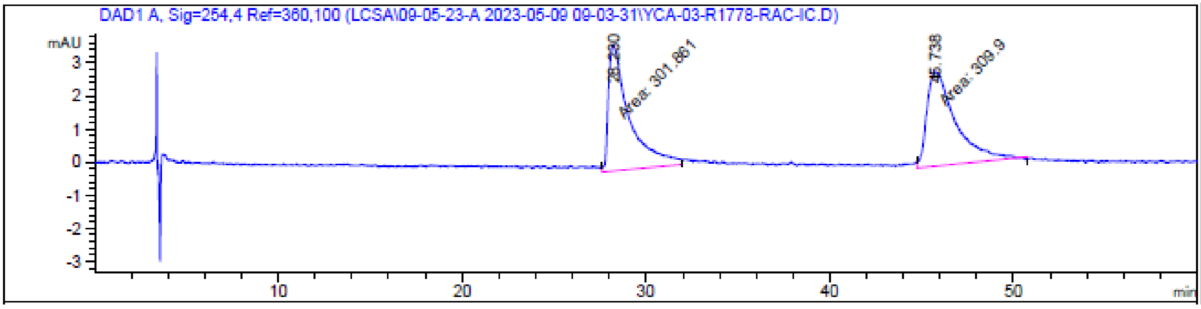


Signal 1: DAD1 A, Sig=254,4 Ref=360,100

| Peak # | RetTime [min] | Type | Width [min] | Area [mAU*s] | Height [mAU] | Area % |
|---|---|---|---|---|---|---|
| 1 | 28.230 | MM | 1.3192 | 301.86053 | 3.81363 | 49.3429 |
| 2 | 45.738 | MM | 1.8072 | 309.89975 | 2.85798 | 50.6571 |

Totals : 611.76028 6.67162

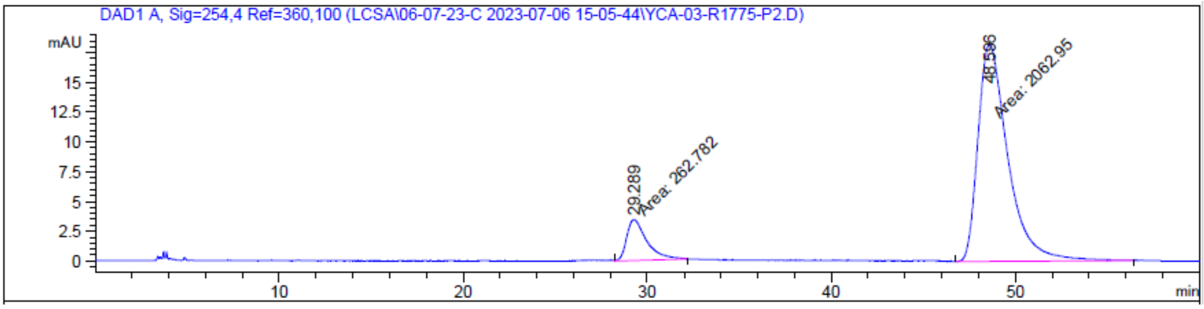


Signal 1: DAD1 A, Sig=254,4 Ref=360,100

| Peak # | RetTime [min] | Type | Width [min] | Area [mAU*s] | Height [mAU] | Area % |
|---|---|---|---|---|---|---|
| 1 | 29.289 | MM | 1.2777 | 262.78195 | 3.42788 | 11.2989 |
| 2 | 48.596 | MM | 1.8875 | 2062.95337 | 18.21574 | 88.7011 |

Totals : 2325.73532 21.64361

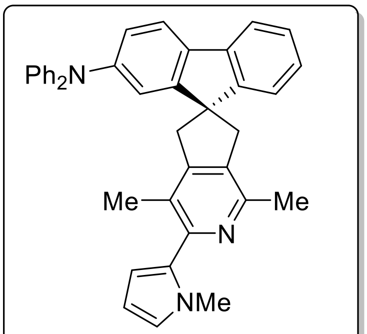

**8r**

**Name**: (*R*)-1,4-dimethyl-3-(1-methyl-1H-pyrrol-2-yl)-*N*,*N*-diphenyl-5,7-dihydrospiro[cyclopenta[c]pyridine-6,9'-fluoren]-2'-amine (**8r**).

**Synthesis**: Following general procedure E, (using **1d** 43.8 mg, 0.1 mmol; **6r** 10.6 mg, 0.1 mmol; **Ni3** 3.6 mg, 3.0 μmol) compound **8r** was obtained in 39.5 mg, 0.0726 mmol, 73% yield and 97:3 er as a colorless foam.

**$^1$H NMR** (400 MHz, $CDCl_3$) δ 7.66 (d, *J* = 7.6 Hz, 1H), 7.63 – 7.56 (m, 1H), 7.34 (dq, *J* = 8.2, 4.1 Hz, 1H), 7.30 – 7.21 (m, 4H), 7.20 – 7.16 (m, 2H), 7.14 – 7.07 (m, 6H), 7.04 – 6.98 (m, 2H), 6.73 – 6.68 (m, 1H), 6.31 – 6.24 (m, 1H), 6.22 – 6.17 (m, 1H), 3.63 (s, 3H), 3.36 (d, *J* = 10.7 Hz, 4H), 2.43 (s, 3H), 2.20 (s, 3H).

**$^{13}$C NMR** (101 MHz, $CDCl_3$) δ 153.4, 152.8, 152.1, 150.3, 149.5, 148.0, 147.9, 139.3, 134.5, 134.4, 131.4, 129.3, 127.7, 127.0, 126.5, 124.4, 123.4, 123.0, 122.0, 120.7, 119.4, 117.9, 110.7, 107.0, 56.5, 45.1, 44.2, 35.2, 22.0, 16.4.

**HRMS** (ESI/QTOF) m/z: $[M + H]^+$ Calcd for $C_{39}H_{34}N_3^+$ 544.2747; Found 544.2757.

$[\alpha]_D^{20}$**:** 66.7 (c = 0.11, $CHCl_3$).

**IR (ATR)**: $\tilde{\nu}$ ($cm^{-1}$) = 3036, 2961, 2923, 2853, 1584, 1542, 1485, 1468, 1449, 1426, 1414, 1377, 1346, 1330, 1314, 1274, 1261, 1220, 1193, 1174, 1155, 1090, 1057, 1026, 961, 872, 801, 754, 735, 716, 696, 660, 608, 515.

**Melting point**: -

**$R_f$**: 0.38 (ethyl acetate: pentane = 1:4).

**HPLC:** Chiralpak IF 4.6 x 250 mm; hexane:*i*-PrOH 99:1, 1 mL/min, 254 nm; *t*R (major) = 14.6 min, *t*R (minor) = 18.3 min, 97:3 er.

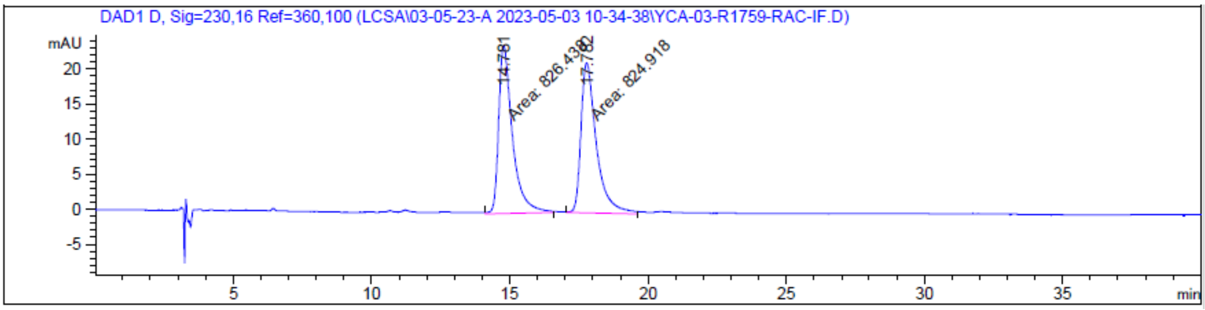


Signal 3: DAD1 D, Sig=230,16 Ref=360,100

| Peak # | RetTime [min] | Type | Width [min] | Area [mAU*s] | Height [mAU] | Area % |
|---|---|---|---|---|---|---|
| 1 | 14.781 | MM | 0.5764 | 826.43835 | 23.89776 | 50.0460 |
| 2 | 17.782 | MM | 0.6441 | 824.91797 | 21.34551 | 49.9540 |

Totals : 1651.35632 45.24327

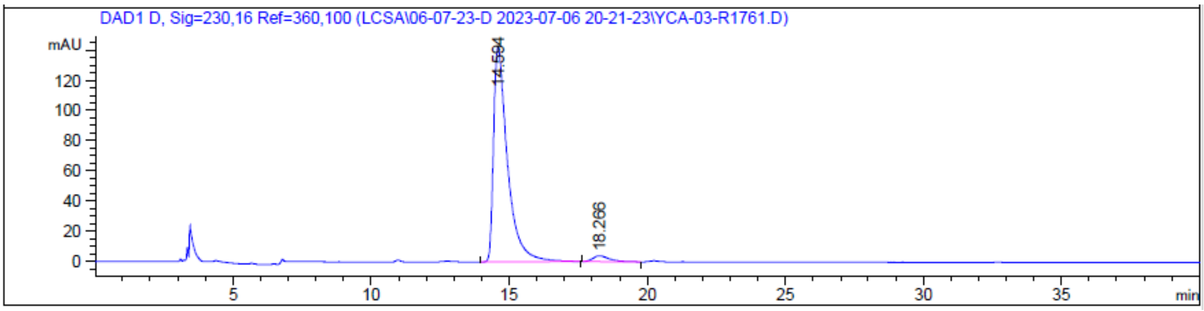


Signal 3: DAD1 D, Sig=230,16 Ref=360,100

| Peak # | RetTime [min] | Type | Width [min] | Area [mAU*s] | Height [mAU] | Area % |
|---|---|---|---|---|---|---|
| 1 | 14.594 | BB | 0.5153 | 4990.30273 | 142.24870 | 97.0275 |
| 2 | 18.266 | BB | 0.5737 | 152.88257 | 3.79757 | 2.9725 |

Totals : 5143.18530 146.04627

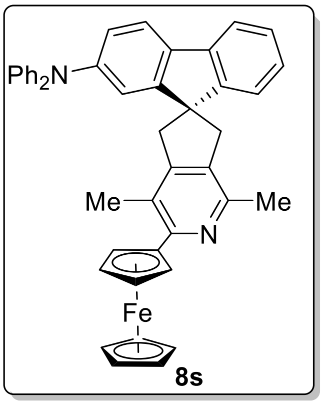


**Synthesis**: Following general procedure E, (using **1d** 43.8 mg, 0.1 mmol; **6s** 21.1 mg, 0.1 mmol; **Ni3** 3.6 mg, 3.0 μmol) compound **8s** was obtained in 57.0 mg, 0.0878 mmol, 88% yield and 86:14 er as an orange foam.

**$^{1}$H NMR** (400 MHz, $CDCl_3$) δ 7.64 (d, *J* = 7.6 Hz, 1H), 7.59 (d, *J* = 8.3 Hz, 1H), 7.35 – 7.30 (m, 1H), 7.26 – 7.20 (m, 4H), 7.16 – 7.06 (m, 8H), 7.03 – 6.98 (m, 2H), 4.99 – 4.77 (m, 2H), 4.36 (s, 2H), 4.12 (s, 5H), 3.42 – 3.23 (m, 4H), 2.56 – 2.30 (m, 6H).

**$^{13}$C NMR** (101 MHz, $CDCl_3$) δ 154.3, 153.3, 148.0, 147.9, 139.2, 134.6, 129.4, 127.7, 127.1, 124.3, 123.6, 123.0, 121.9, 120.7, 119.4, 118.4, 112.7, 70.5 – 68.7 (m), 56.5, 45.1, 44.1, 16.6.

**HRMS** (ESI/QTOF) m/z: $[M + H]^+$ Calcd for $C_{44}H_{37}FeN_2^+$ 649.2301; Found 649.2313.

$[\boldsymbol{\alpha}]_{\boldsymbol{D}}^{\mathbf{20}}$**:** 45.8 (c = 0.20, $CHCl_3$).

**IR (ATR)**: $\tilde{\nu}$ ($cm^{-1}$) = 3036, 2960, 2924, 2851, 1584, 1486, 1450, 1426, 1346, 1330, 1313, 1275, 1263, 1220, 1174, 1155, 1105, 1075, 1049, 1028, 1002, 888, 818, 780, 753, 735, 697, 658, 501, 482.

**Melting point**: -

**$R_f$**: 0.73 (ethyl acetate: pentane = 1:4).

**HPLC:** Chiralpak ID 4.6 x 250 mm; hexane:*i*-PrOH 98:2, 1 mL/min, 254 nm; *t*R (major) = 5.1 min, *t*R (minor) = 6.5 min, 86:14 er.

## Chromatogram

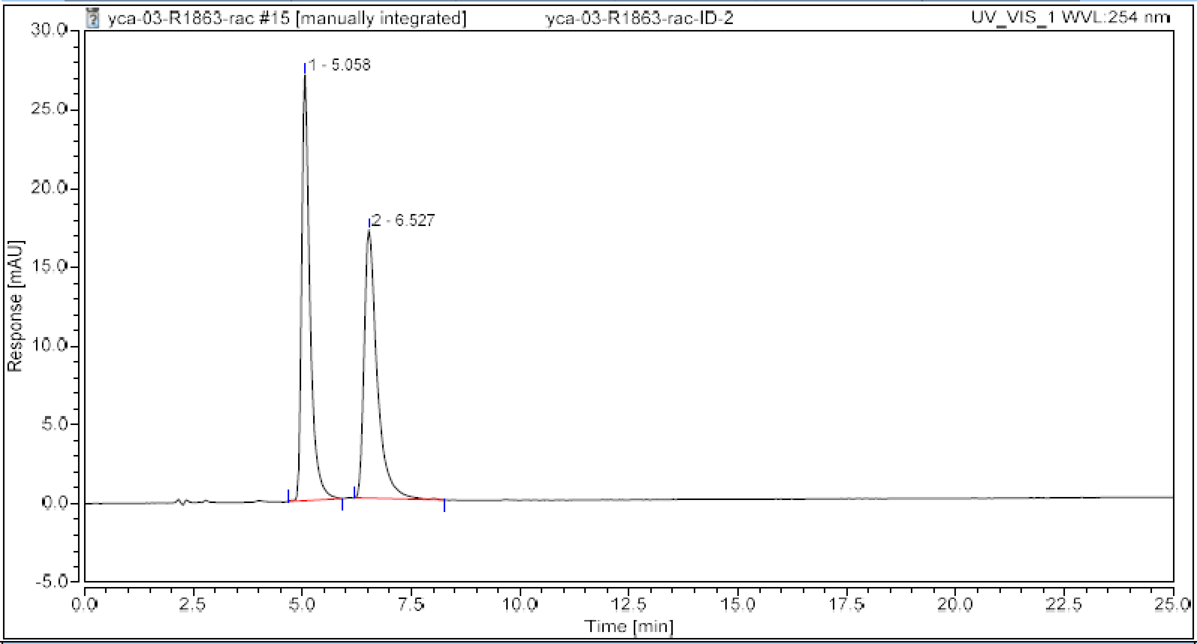


## Integration Results

| No. | Peak Name | Retention Time min | Area mAU*min | Height mAU | Relative Area % | Relative Height % | Amount n.a. |
|---|---|---|---|---|---|---|---|
| 1 | | 5.058 | 5.978 | 27.039 | 50.46 | 61.36 | n.a. |
| 2 | | 6.527 | 5.867 | 17.025 | 49.54 | 38.64 | n.a. |
| **Total:** | | | **11.845** | **44.064** | **100.00** | **100.00** | |

## Chromatogram

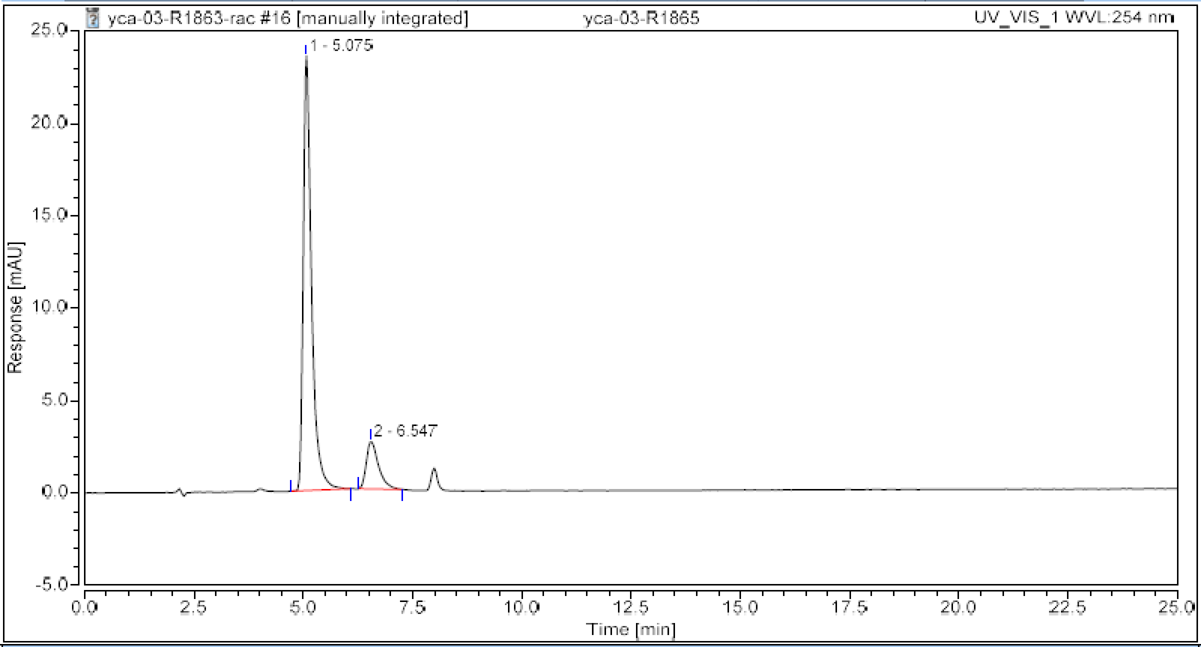


## Integration Results

| No. | Peak Name | Retention Time min | Area mAU*min | Height mAU | Relative Area % | Relative Height % | Amount n.a. |
|---|---|---|---|---|---|---|---|
| 1 | | 5.075 | 5.156 | 23.523 | 86.02 | 90.10 | n.a. |
| 2 | | 6.547 | 0.838 | 2.586 | 13.98 | 9.90 | n.a. |
| **Total:** | | | **5.994** | **26.109** | **100.00** | **100.00** | |

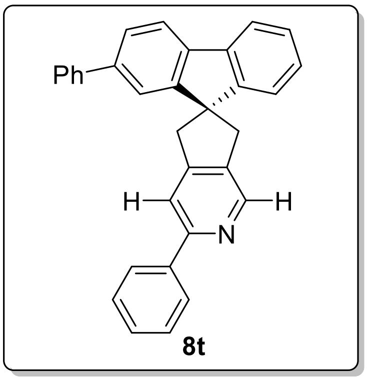


**Name**: (*R*)-2',3-diphenyl-5,7-dihydrospiro[cyclopenta[c]pyridine-6,9'-fluorene] (**8t**).

**Synthesis**: Following general procedure E, (using **1h** 31.8 mg, 0.1 mmol; **6a** 10.3 mg, 0.1 mmol; **Ni3** 3.6 mg, 3.0 μmol) compound **8t** was obtained in 36.1 mg, 0.0856 mmol, 85% yield and 80:20 er as pale-yellow thick oil.

**$^{1}$H NMR** (400 MHz, $CDCl_3$) δ 8.67 (s, 1H), 8.16 – 7.98 (m, 2H), 7.85 – 7.72 (m, 3H), 7.63 (dd, *J* = 7.9, 1.8 Hz, 1H), 7.59 – 7.48 (m, 5H), 7.47 – 7.37 (m, 4H), 7.36 – 7.31 (m, 1H), 7.27 – 7.20 (m, 2H), 3.70 – 3.44 (m, 4H).

**$^{13}$C NMR** (101 MHz, $CDCl_3$) δ 156.4, 153.8, 152.3, 145.6, 141.3, 141.1, 139.6, 139.3, 137.7, 129.0, 128.9, 127.9, 127.4, 127.2, 127.1, 127.0, 122.3, 121.2, 120.4, 120.2, 117.2, 57.3, 45.5, 43.0.

**HRMS** (ESI/QTOF) m/z: $[M + H]^+$ Calcd for $C_{32}H_{24}N^+$ 422.1903; Found 422.1910.

$[\boldsymbol{\alpha}]_{\boldsymbol{D}}^{\mathbf{20}}$**:** 41.3 (c = 0.25, $CHCl_3$).

**IR (ATR)**: $\tilde{\nu}$ ($cm^{-1}$) = 3055, 3029, 2923, 2851, 1600, 1556, 1476, 1468, 1451, 1424, 1414, 1381, 1262, 1224, 1156, 1101, 1074, 1022, 890, 834, 804, 778, 759, 736, 695, 675, 413.

**Melting point**: -

**$R_f$**: 0.50 (ethyl acetate: pentane = 1:4).

**HPLC:** Chiralpak IC 4.6 x 250 mm; hexane:*i*-PrOH 95:5, 1 mL/min, 254 nm; *t*R (major) = 20.0 min, *t*R (minor) = 13.6 min, 80:20 er.

**Chromatogram**

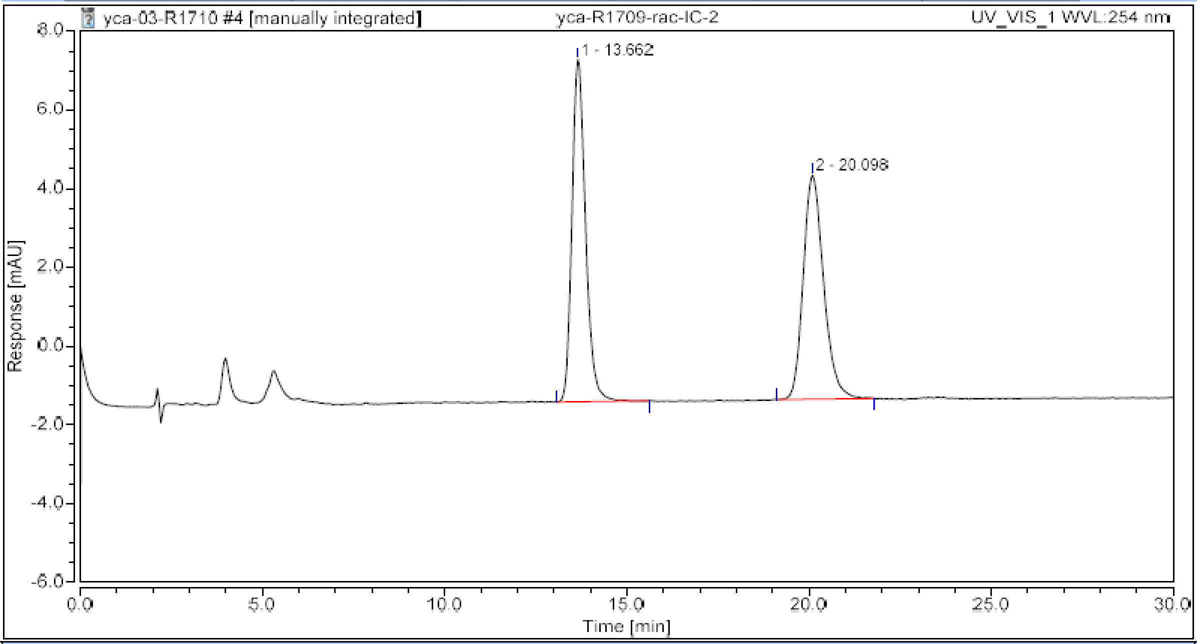


**Integration Results**

| No. | Peak Name | Retention Time min | Area mAU*min | Height mAU | Relative Area % | Relative Height % | Amount n.a. |
|---|---|---|---|---|---|---|---|
| 1 | | 13.662 | 3.743 | 8.700 | 50.26 | 60.46 | n.a. |
| 2 | | 20.098 | 3.705 | 5.689 | 49.74 | 39.54 | n.a. |
| **Total:** | | | **7.448** | **14.389** | **100.00** | **100.00** | |

**Chromatogram**

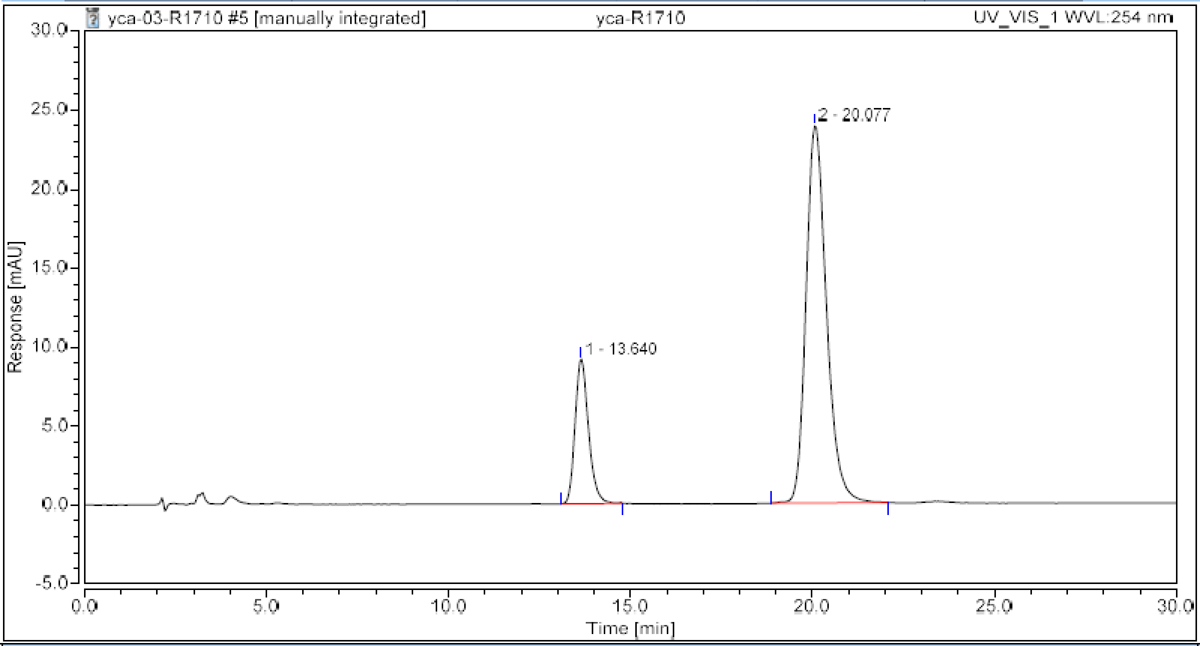


**Integration Results**

| No. | Peak Name | Retention Time min | Area mAU*min | Height mAU | Relative Area % | Relative Height % | Amount n.a. |
|---|---|---|---|---|---|---|---|
| 1 | | 13.640 | 3.932 | 9.179 | 20.17 | 27.75 | n.a. |
| 2 | | 20.077 | 15.562 | 23.895 | 79.83 | 72.25 | n.a. |
| **Total:** | | | **19.494** | **33.074** | **100.00** | **100.00** | |

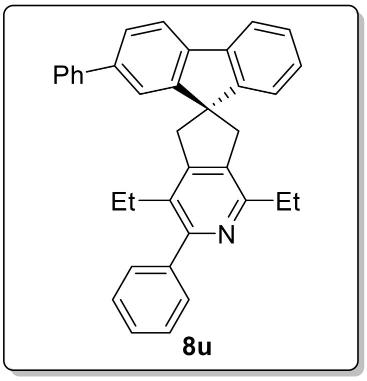


**Name**: (*R*)-1,4-diethyl-2',3-diphenyl-5,7-dihydrospiro[cyclopenta[c]pyridine-6,9'-fluorene] (**8u**).

**Synthesis**: Following general procedure E, (using **1i** 37.5 mg, 0.1 mmol; **6a** 10.3 mg, 0.1 mmol; **Ni3** 3.6 mg, 3.0 μmol) compound **8u** was obtained in 43.1 mg, 0.0902 mmol, 90% yield and 90:10 er as a pale-yellow thick oil.

**$^1$H NMR** (400 MHz, $CDCl_3$) δ 7.85 – 7.74 (m, 2H), 7.66 – 7.51 (m, 6H), 7.49 – 7.30 (m, 7H), 7.27 – 7.22 (m, 2H), 3.64 – 3.43 (m, 4H), 2.80 (q, *J* = 7.6 Hz, 2H), 2.57 (q, *J* = 7.6 Hz, 2H), 1.27 (t, *J* = 7.6 Hz, 3H), 1.04 (t, *J* = 7.5 Hz, 3H).

**$^{13}$C NMR** (101 MHz, $CDCl_3$) δ 157.2, 156.0, 153.0, 152.7, 141.4, 141.1, 139.2, 139.1, 135.1, 131.4, 129.2, 129.0, 128.3, 128.0, 127.7, 127.4, 127.2, 127.0, 122.2, 121.2, 120.4, 120.2, 57.1, 44.5, 43.6, 29.3, 23.2, 14.6, 13.6.

**HRMS** (ESI/QTOF) m/z: $[M + H]^+$ Calcd for $C_{36}H_{32}N^+$ 478.2529; Found 478.2536.

$[\alpha]_D^{20}$**:** 47.2 (c = 0.24, $CHCl_3$).

**IR (ATR)**: $\tilde{\nu}$ ($cm^{-1}$) = 3055, 3030, 2963, 2928, 2870, 1598, 1571, 1481, 1465, 1452, 1416, 1372, 1261, 1156, 1089, 1074, 1020, 914, 891, 833, 800, 758, 735, 697, 669, 613, 593, 574, 506, 445.

**Melting point**: -

**$R_f$**: 0.58 (ethyl acetate: pentane = 1:4).

**HPLC:** Chiralpak IA 4.6 x 250 mm; hexane:*i*-PrOH 95:5, 1 mL/min, 254 nm; *t*R (major) = 3.6 min, *t*R (minor) = 3.2 min, 90:10 er.

**Chromatogram**

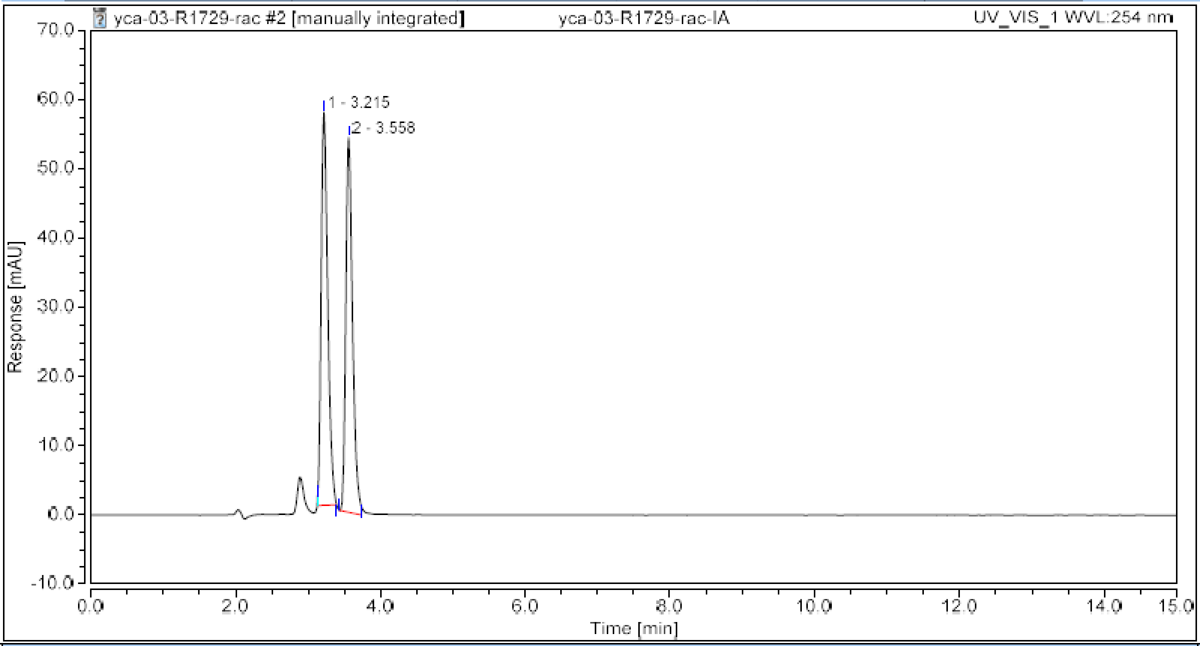


**Integration Results**

| No. | Peak Name | Retention Time min | Area mAU*min | Height mAU | Relative Area % | Relative Height % | Amount n.a. |
|---|---|---|---|---|---|---|---|
| 1 | | 3.215 | 5.940 | 56.860 | 50.80 | 51.19 | n.a. |
| 2 | | 3.558 | 5.754 | 54.208 | 49.20 | 48.81 | n.a. |
| **Total:** | | | **11.695** | **111.069** | **100.00** | **100.00** | |

**Chromatogram**

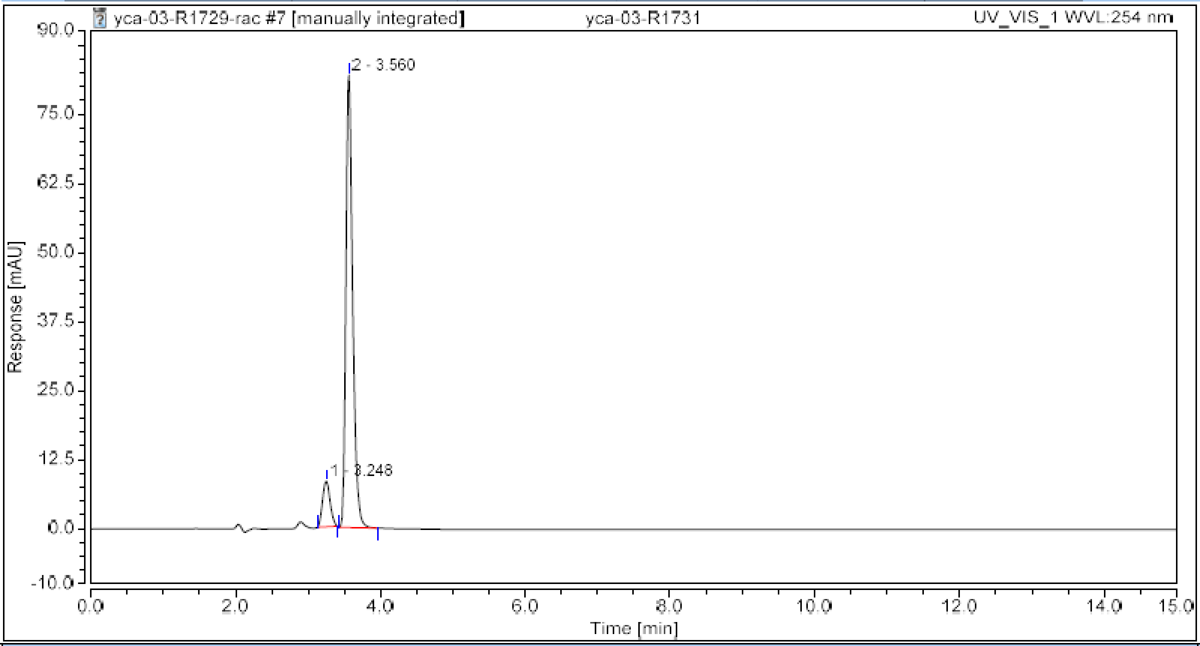


**Integration Results**

| No. | Peak Name | Retention Time min | Area mAU*min | Height mAU | Relative Area % | Relative Height % | Amount n.a. |
|---|---|---|---|---|---|---|---|
| 1 | | 3.248 | 1.001 | 8.270 | 10.25 | 9.18 | n.a. |
| 2 | | 3.560 | 8.766 | 81.861 | 89.75 | 90.82 | n.a. |
| **Total:** | | | **9.768** | **90.132** | **100.00** | **100.00** | |

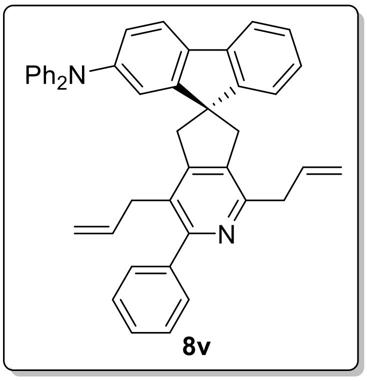


**Name**: (*R*)-1,4-diallyl-*N*,*N*,3-triphenyl-5,7-dihydrospiro[cyclopenta[c]pyridine-6,9'-fluoren]-2'-amine (**8v**).

**Synthesis**: Following general procedure E, (using **1j** 49.0 mg, 0.1 mmol; **6a** 10.3 mg, 0.1 mmol; **Ni3** 3.6 mg, 3.0 μmol) compound **8v** was obtained in 26.2 mg, 0.0442 mmol, 44% yield and 95:5 er as a pale-yellow thick oil.

**$^1$H NMR** (400 MHz, $CDCl_3$) δ 7.65 (d, *J* = 7.5 Hz, 1H), 7.58 (d, *J* = 8.1 Hz, 1H), 7.54 – 7.29 (m, 6H), 7.28 – 7.16 (m, 6H), 7.13 – 6.95 (m, 9H), 6.05 – 5.89 (m, 1H), 5.77 (ddt, *J* = 15.8, 10.6, 5.6 Hz, 1H), 5.11 – 4.88 (m, 3H), 4.79 (dd, *J* = 17.1, 1.9 Hz, 1H), 3.63 – 3.15 (m, 8H).

**$^{13}$C NMR** (101 MHz, $CDCl_3$) δ 153.4, 152.7, 152.3, 148.0, 147.9, 139.5, 136.0, 135.7, 135.2, 134.3, 129.4, 129.2, 128.2, 127.8, 127.0, 124.4, 123.4, 123.1, 122.1, 120.7, 119.5, 117.9, 116.2, 57.0, 44.4, 43.4, 41.2, 34.3.

**HRMS** (ESI/QTOF) m/z: $[M + H]^+$ Calcd for $C_{44}H_{37}N_2^+$ 593.2951; Found 593.2972.

$[\alpha]_D^{20}$**:** 35.0 (c = 0.10, $CHCl_3$).

**IR (ATR)**: $\tilde{\nu}$ ($cm^{-1}$) = 3032, 2240, 2218, 2200, 2033, 2017, 2003, 1991, 1910, 1847, 1837, 1804, 1750, 1722, 1680, 1663, 16541489, 1451, 1426, 1315, 884, 821, 667, 630, 600, 572, 553, 542, 515, 452, 442, 435, 419, 410, 403.

**Melting point**: -

**R$_f$**: 0.67 (ethyl acetate: pentane = 1:4).

**HPLC:** Chiralpak IA 4.6 x 250 mm; hexane:*i*-PrOH 98:2, 1 mL/min, 254 nm; *t*R (major) = 5.2 min, *t*R (minor) = 4.8 min, 95:5 er.

**Chromatogram**

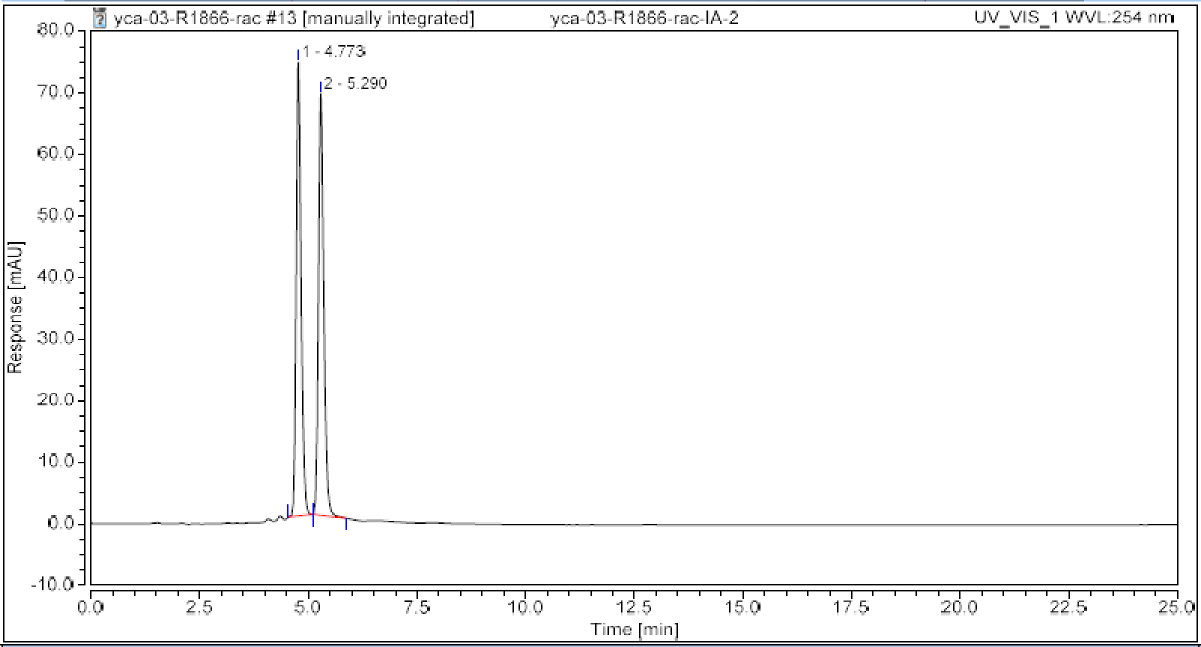


**Integration Results**

| No. | Peak Name | Retention Time<br>min | Area<br>mAU*min | Height<br>mAU | Relative Area<br>% | Relative Height<br>% | Amount<br>n.a. |
|---|---|---|---|---|---|---|---|
| 1 | | 4.773 | 9.566 | 73.699 | 49.66 | 51.81 | n.a. |
| 2 | | 5.290 | 9.696 | 68.537 | 50.34 | 48.19 | n.a. |
| **Total:** | | | **19.262** | **142.236** | **100.00** | **100.00** | |

**Chromatogram**

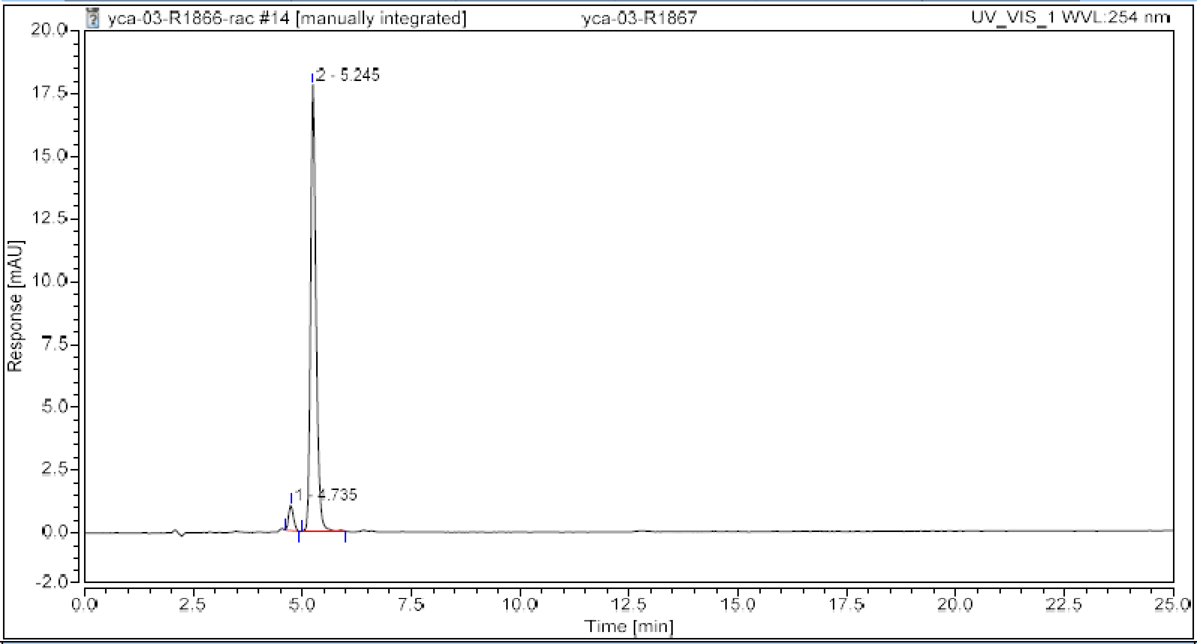


**Integration Results**

| No. | Peak Name | Retention Time<br>min | Area<br>mAU*min | Height<br>mAU | Relative Area<br>% | Relative Height<br>% | Amount<br>n.a. |
|---|---|---|---|---|---|---|---|
| 1 | | 4.735 | 0.130 | 1.023 | 4.77 | 5.43 | n.a. |
| 2 | | 5.245 | 2.585 | 17.809 | 95.23 | 94.57 | n.a. |
| **Total:** | | | **2.714** | **18.832** | **100.00** | **100.00** | |

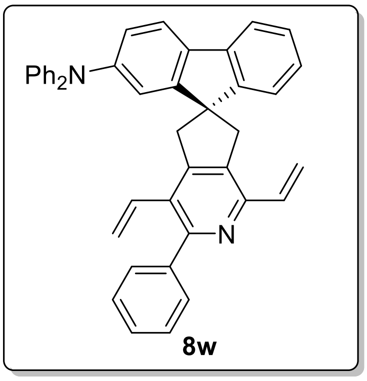

**Name**: (*R*)-*N*,*N*,3-triphenyl-1,4-divinyl-5,7-dihydrospiro[cyclopenta[c]pyridine-6,9'-fluoren]-2'-amine (**8w**).

**Synthesis**: Following general procedure E, (using **1k** 46.2 mg, 0.1 mmol; **6a** 10.3 mg, 0.1 mmol; **Ni3** 3.6 mg, 3.0 μmol) compound **8w** was obtained in 34.5 mg, 0.610 mmol, 61% yield and 90:10 er as a pale-yellow thick oil.

**$^1$H NMR** (400 MHz, $CDCl_3$) δ 7.73 – 7.51 (m, 4H), 7.50 – 7.30 (m, 4H), 7.30 – 7.19 (m, 4H), 7.20 – 6.90 (m, 10H), 6.82 (dd, *J* = 18.3, 9.9 Hz, 1H), 6.62 (dd, *J* = 17.4, 12.1 Hz, 1H), 6.32 (d, *J* = 17.3 Hz, 1H), 5.49 (d, *J* = 10.8 Hz, 1H), 5.44 – 5.13 (m, 2H), 3.52 (d, *J* = 11.4 Hz, 4H).

**$^{13}$C NMR** (101 MHz, $CDCl_3$) δ 156.6, 152.8, 152.2, 151.5, 148.9, 148.0, 147.8, 140.6, 139.4, 135.4, 134.4, 134.3, 134.2, 130.3, 129.4, 128.1, 128.0, 127.8, 127.7, 127.0, 124.5, 123.1, 122.0, 120.7, 119.6, 119.4, 119.3, 117.6, 57.2, 46.3, 43.6.

**HRMS** (ESI/QTOF) m/z: $[M + H]^+$ Calcd for $C_{42}H_{33}N_2^+$ 565.2638; Found 565.2648.

$[\boldsymbol{\alpha}]_{\boldsymbol{D}}^{\boldsymbol{20}}$**:** 33.3 (c = 0.11, $CHCl_3$).

**IR (ATR)**: $\tilde{\nu}$ ($cm^{-1}$) =: 3055, 3036, 2959, 2923, 2851, 1718, 1586, 1554, 1488, 1468, 1450, 1426, 1372, 1345, 1330, 1314, 1277, 1221, 1175, 1155, 1099, 1075, 1028, 921, 873, 823, 802, 772, 754, 737, 697, 669, 652, 573, 516, 453, 427.

**Melting point**: -

**$R_f$**: 0.92 (ethyl acetate: pentane = 1:4).

**HPLC:** Chiralpak IB 4.6 x 250 mm; hexane:*i*-PrOH 99:1, 1 mL/min, 254 nm; *t*R (major) = 7.2 min, *t*R (minor) = 6.4 min, 90:10 er.

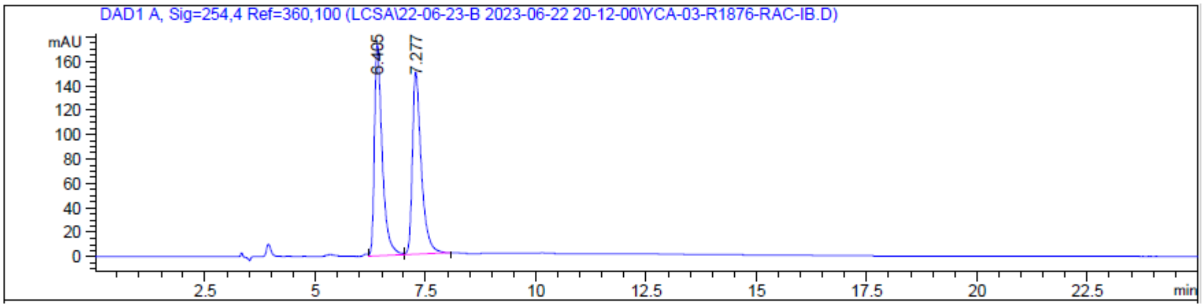


Signal 1: DAD1 A, Sig=254,4 Ref=360,100

| Peak # | RetTime [min] | Type | Width [min] | Area [mAU*s] | Height [mAU] | Area % |
|---|---|---|---|---|---|---|
| 1 | 6.405 | VV | 0.1808 | 2120.17432 | 173.17906 | 50.3104 |
| 2 | 7.277 | VB | 0.2081 | 2094.01489 | 148.91505 | 49.6896 |

Totals : 4214.18921 322.09412

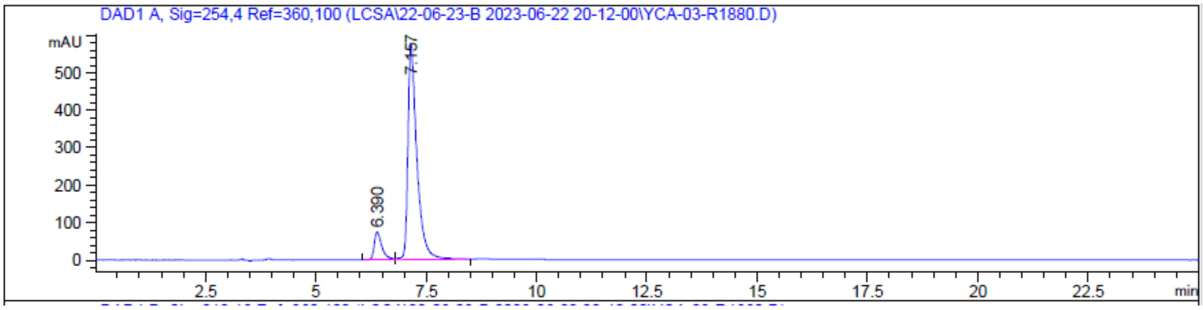


Signal 1: DAD1 A, Sig=254,4 Ref=360,100

| Peak # | RetTime [min] | Type | Width [min] | Area [mAU*s] | Height [mAU] | Area % |
|---|---|---|---|---|---|---|
| 1 | 6.390 | BV | 0.1776 | 892.91400 | 74.64552 | 9.8806 |
| 2 | 7.157 | VB | 0.2054 | 8144.10742 | 574.62585 | 90.1194 |

Totals : 9037.02142 649.27138

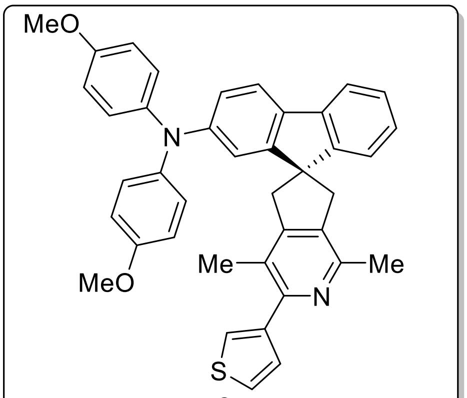

**8x**

**Name**: (*R*)-*N*,*N*-bis(4-methoxyphenyl)-1,4-dimethyl-3-(thiophen-3-yl)-5,7-dihydrospiro[cyclopenta[c]pyridine-6,9'-fluoren]-2'-amine (**8x**).

**Synthesis**: Following general procedure E, (using **1e** 49.8 mg, 0.1 mmol; **6p** 10.9 mg, 0.1 mmol; **Ni3** 3.6 mg, 3.0 μmol) compound **8x** was obtained in 35.2 mg, 0.058 mmol, 58% yield and 96:4 er as a pale-yellow foam.

**$^1$H NMR** (400 MHz, $CDCl_3$) δ 7.61 (d, *J* = 7.5 Hz, 1H), 7.55 – 7.48 (m, 2H), 7.43 – 7.36 (m, 2H), 7.35 – 7.28 (m, 1H), 7.16 – 7.11 (m, 2H), 7.06 (d, *J* = 8.9 Hz, 4H), 6.97 – 6.91 (m, 2H), 6.82 (d, *J* = 8.9 Hz, 4H), 3.78 (s, 6H), 3.34 (d, *J* = 10.8 Hz, 4H), 2.46 (s, 3H), 2.25 (s, 3H).

**$^{13}$C NMR** (101 MHz, $CDCl_3$) δ 155.9, 153.0, 152.5, 152.0, 150.6, 149.1, 141.1, 139.6, 135.2, 132.4, 129.1, 127.7, 126.5, 125.0, 124.8, 124.6, 121.8, 120.4, 120.2, 119.1, 114.8, 56.6, 55.6, 45.1, 44.2, 22.0, 16.5.

**HRMS** (ESI/QTOF) m/z: $[M + H]^+$ Calcd for $C_{40}H_{35}N_2O_2S^+$ 607.2414; Found 607.2429.

$[\alpha]_D^{20}$**:** 75.0 (c = 0.12, $CHCl_3$).

**IR (ATR)**: $\tilde{\nu}$ ($cm^{-1}$) = 2925, 1608, 1579, 1505, 1453, 1347, 1323, 1276, 1241, 1180, 1104, 1035, 943, 895, 860, 828, 800, 746, 735, 676, 650, 599, 576, 554, 540, 500, 468, 448, 433, 413, 402.

**Melting point**: -

**$R_f$**: 0.17 (ethyl acetate: pentane = 1:4).

**HPLC:** Chiralpak IA 4.6 x 250 mm; hexane:*i*-PrOH 95:5, 1 mL/min, 254 nm; *t*R (major) = 26.2 min, *t*R (minor) = 13.4 min, 96:4 er.

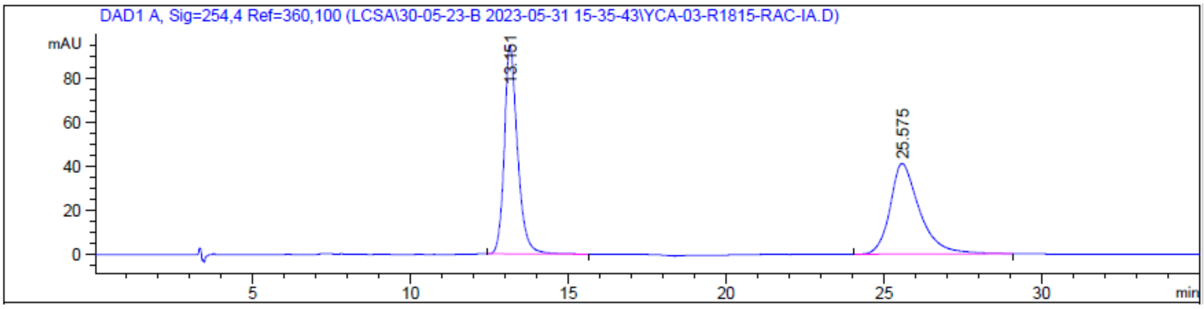


Signal 1: DAD1 A, Sig=254,4 Ref=360,100

| Peak # | RetTime [min] | Type | Width [min] | Area [mAU*s] | Height [mAU] | Area % |
|---|---|---|---|---|---|---|
| 1 | 13.151 | BB | 0.4331 | 2748.44238 | 94.56483 | 50.6906 |
| 2 | 25.575 | BB | 0.9317 | 2673.55127 | 41.05798 | 49.3094 |

Totals : 5421.99365 135.62281

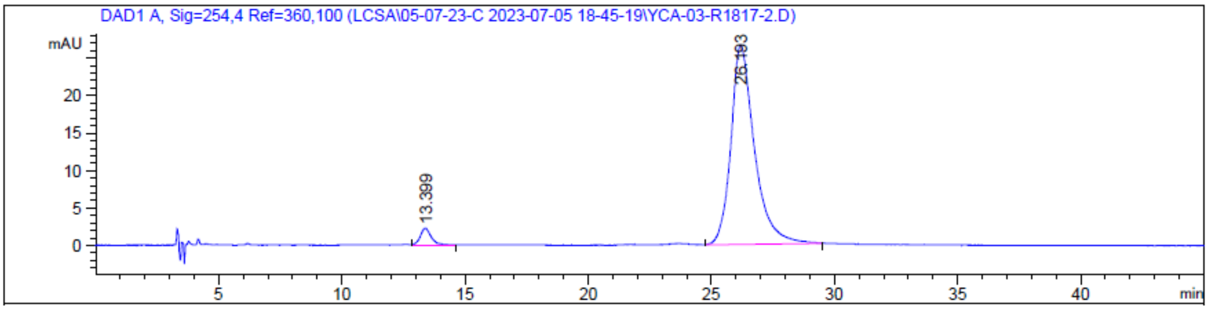


Signal 1: DAD1 A, Sig=254,4 Ref=360,100

| Peak # | RetTime [min] | Type | Width [min] | Area [mAU*s] | Height [mAU] | Area % |
|---|---|---|---|---|---|---|
| 1 | 13.399 | BB | 0.3971 | 67.01641 | 2.21440 | 3.6661 |
| 2 | 26.193 | BB | 0.9499 | 1760.99890 | 26.54801 | 96.3339 |

Totals : 1828.01531 28.76241

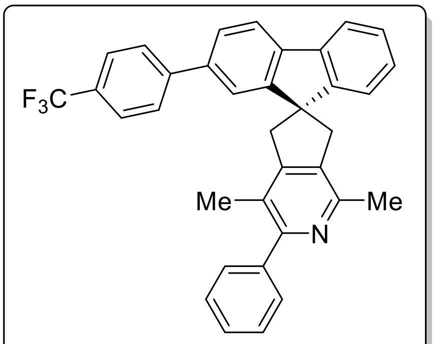

**8y**

**Name**: (*R*)-1,4-dimethyl-3-phenyl-2'-(4-(trifluoromethyl)phenyl)-5,7-dihydrospiro[cyclopenta[c]pyridine-6,9'-fluorene] (**8y**).

**Synthesis**: Following general procedure E, (using **1c** 41.4 mg, 0.1 mmol; **6a** 10.3 mg, 0.1 mmol; **Ni3** 3.6 mg, 3.0 μmol) compound **8y** was obtained in 49.1 mg, 0.095 mmol, 95% yield and 91:9 er as a white foam.

**$^1$H NMR** (400 MHz, $CDCl_3$) δ 7.85 (d, *J* = 7.9 Hz, 1H), 7.80 (d, *J* = 7.5 Hz, 1H), 7.71 – 7.66 (m, 4H), 7.63 (dd, *J* = 7.9, 1.7 Hz, 1H), 7.60 – 7.55 (m, 3H), 7.50 – 7.37 (m, 4H), 7.30 (d, *J* = 4.2 Hz, 2H), 3.63 – 3.42 (m, 4H), 2.52 (s, 3H), 2.23 (s, 3H).

**$^{13}$C NMR** (101 MHz, $CDCl_3$) δ 157.2, 153.4 (d, *J* = 2.8 Hz), 152.2, 151.0, 144.9, 141.0, 140.0, 139.6, 138.8, 135.1, 129.4, 128.4, 128.3, 127.9, 127.8, 127.5, 127.1, 125.9 (q, *J* = 3.8 Hz), 124.8, 122.4, 121.4, 120.6, 120.3, 56.6, 45.3, 44.3, 22.2, 16.5.

**$^{19}$F NMR** (376 MHz, $CDCl_3$) δ -62.4.

**HRMS** (ESI/QTOF) m/z: $[M + H]^+$ Calcd for $C_{35}H_{27}F_3N^+$ 518.2090; Found 518.2094.

$[\alpha]_D^{20}$**:** 26.8 (c = 0.13, $CHCl_3$).

**IR (ATR)**: $\tilde{\nu}$ ($cm^{-1}$) =: 2923, 1589, 1487, 1444, 1279, 1220, 1165, 1124, 1107, 1066, 1018, 971, 849, 823, 801, 779, 754, 736, 697, 668, 651, 630, 515, 429, 406.

**Appearance**: white foam

**Melting point**: -

**$R_f$**: 0.73 (ethyl acetate: pentane = 1:4).

**HPLC**: Chiralpak ID 4.6 x 250 mm; hexane:*i*-PrOH 98:2, 1.0 mL/min, 254 nm; *t*R (major) = 5.5 min, *t*R (minor) = 6.6 min, 92:8 er.

**Chromatogram**

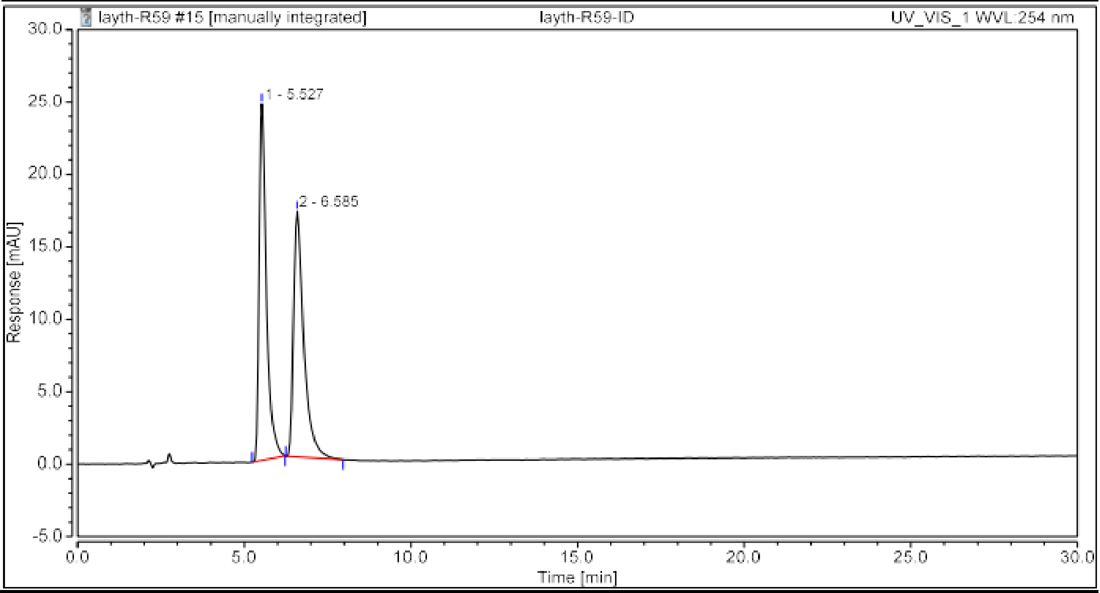


**Integration Results**

| No. | Peak Name | Retention Time<br>min | Area<br>mAU*min | Height<br>mAU | Relative Area<br>% | Relative Height<br>% | Amount<br>n.a. |
|---|---|---|---|---|---|---|---|
| 1 | | 5.527 | 6.149 | 24.602 | 50.22 | 59.20 | n.a. |
| 2 | | 6.585 | 6.095 | 16.952 | 49.78 | 40.80 | n.a. |
| **Total:** | | | **12.244** | **41.555** | **100.00** | **100.00** | |

**Chromatogram**

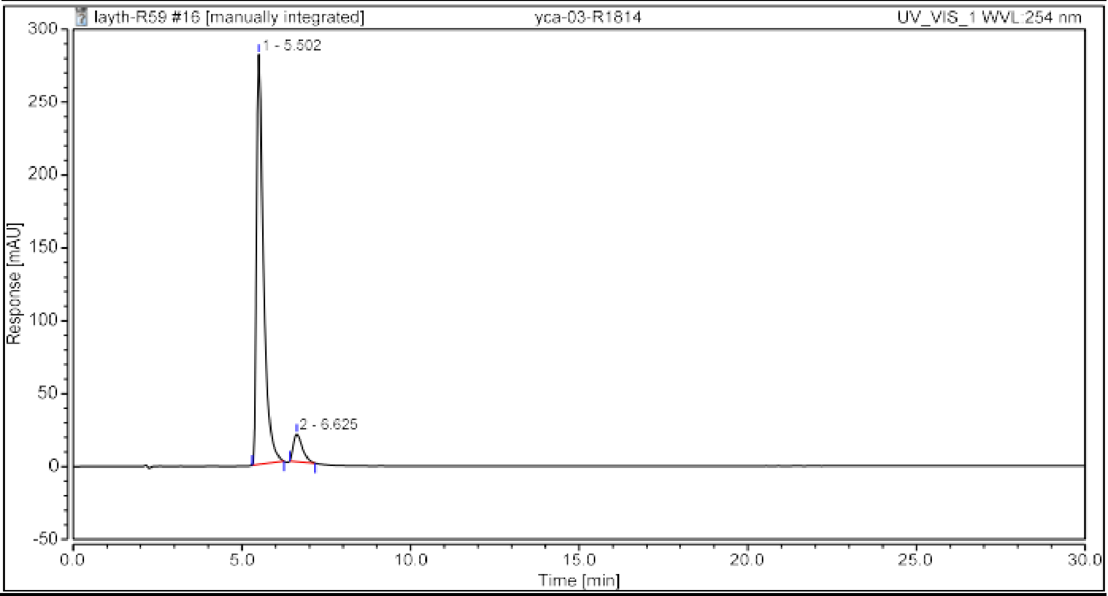


**Integration Results**

| No. | Peak Name | Retention Time<br>min | Area<br>mAU*min | Height<br>mAU | Relative Area<br>% | Relative Height<br>% | Amount<br>n.a. |
|---|---|---|---|---|---|---|---|
| 1 | | 5.502 | 67.743 | 281.317 | 91.98 | 93.70 | n.a. |
| 2 | | 6.625 | 5.906 | 18.908 | 8.02 | 6.30 | n.a. |
| **Total:** | | | **73.648** | **300.225** | **100.00** | **100.00** | |

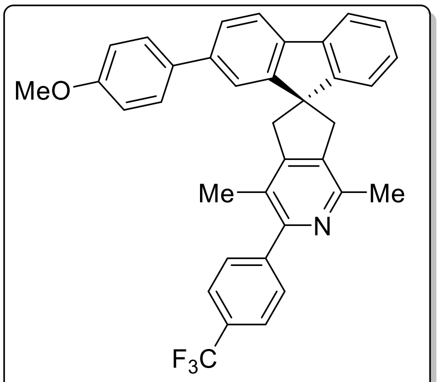

**8z**

**Name**: (*R*)-2'-(4-methoxyphenyl)-1,4-dimethyl-3-(4-(trifluoromethyl)phenyl)-5,7-dihydrospiro[cyclopenta[c]pyridine-6,9'-fluorene] (**8z**).

**Synthesis**: Following general procedure E, (using **1b** 37.6 mg, 0.1 mmol; **6d** 17.1 mg, 0.1 mmol; **Ni3** 3.6 mg, 3.0 μmol) compound **8z** was obtained in 24.0 mg, 0.044 mmol, 44% yield and 92:8 er as a white foam.

**$^1$H NMR** (400 MHz, $CDCl_3$) δ 7.82 – 7.68 (m, 6H), 7.61 – 7.56 (m, 1H), 7.54 – 7.47 (m, 3H), 7.42 – 7.36 (m, 1H), 7.29 – 7.25 (m, 2H), 7.01 – 6.93 (m, 2H), 3.85 (s, 3H), 3.63 – 3.42 (m, 4H), 2.52 (s, 3H), 2.22 (s, 3H).

**$^{13}$C NMR** (101 MHz, $CDCl_3$) δ 159.4, 155.5, 153.0 (d, *J* = 5.3 Hz), 151.4, 140.9, 139.3, 138.4, 136.1, 133.9, 129.9, 128.3, 127.9 (d, *J* = 2.7 Hz), 126.6, 125.4 – 125.2 (m), 122.2, 120.7, 120.42, 120.1, 56.5, 55.5, 45.4, 44.3, 22.1, 16.4.

**$^{19}$F NMR** (376 MHz, $CDCl_3$) δ -62.2.

**HRMS** (ESI/QTOF) m/z: $[M + H]^+$ Calcd for $C_{36}H_{29}F_3NO^+$ 548.2196; Found 548.2211.

$[\boldsymbol{\alpha}]_D^{20}$**:** 37.4 (c = 0.10, $CHCl_3$).

**IR (ATR)**: $\tilde{\nu}$ ($cm^{-1}$) =: 2923, 2836, 1606, 1582, 1571, 1517, 1486, 1465, 1453, 1427, 1404, 1322, 1294, 1246, 1163, 1121, 1106, 1065, 1044, 1018, 971, 848, 822, 805, 782, 758, 736, 703, 668, 648, 633, 622, 579, 549, 524, 497, 451, 427, 411.

**Melting point**: -

**R$_f$**: 0.63 (ethyl acetate: pentane = 1:4).

**HPLC**: Chiralpak IA; hexane:iPrOH 85:15, 1 mL/min, 360 nm; *t*R (major) = 14.3 min, *t*R (minor) = 16.1 min, 92:8 er.

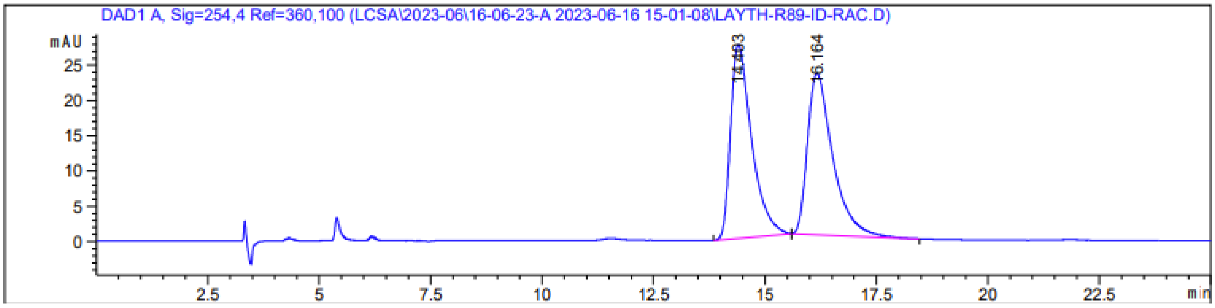


| Peak # | RetTime [min] | Type | Width [min] | Area [mAU*s] | Height [mAU] | Area % |
|---|---|---|---|---|---|---|
| 1 | 14.403 | BB | 0.4970 | 905.55853 | 27.44259 | 50.0415 |
| 2 | 16.164 | BB | 0.5857 | 904.05481 | 22.84621 | 49.9585 |

Totals : 1809.61334 50.28881

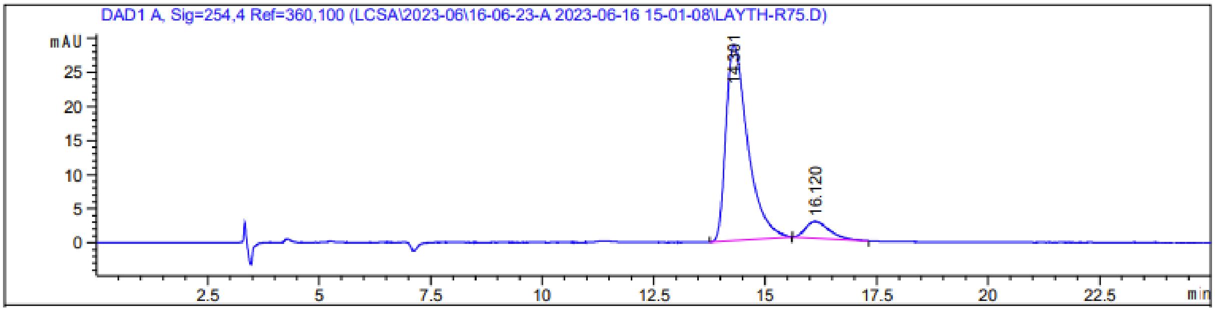


| Peak # | RetTime [min] | Type | Width [min] | Area [mAU*s] | Height [mAU] | Area % |
|---|---|---|---|---|---|---|
| 1 | 14.301 | BB | 0.5107 | 976.72473 | 28.72775 | 91.6234 |
| 2 | 16.120 | BB | 0.4922 | 89.29681 | 2.43315 | 8.3766 |

Totals : 1066.02155 31.16090

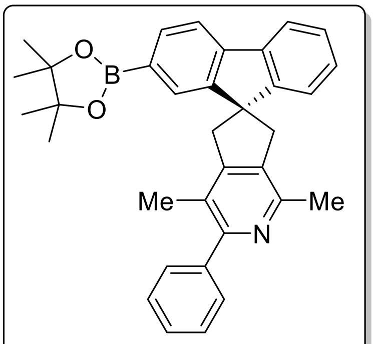


**8aa**

**Name**: (*R*)-1,4-dimethyl-3-phenyl-2'-(4,4,5,5-tetramethyl-1,3,2-dioxaborolan-2-yl)-5,7-dihydrospiro[cyclopenta[c]pyridine-6,9'-fluorene] (**8aa**).

**Synthesis**: Following general procedure E, (using **1l** 39.6 mg, 0.1 mmol; **6a** 10.3 mg, 0.1 mmol; **Ni3** 3.6 mg, 3.0 μmol) compound **8aa** was obtained in 43.9 mg, 0.088 mmol, 88% yield and 87:13 er as a white foam.

**$^{1}$H NMR** (400 MHz, $CDCl_3$) δ 7.91 (s, 1H), 7.89 – 7.85 (m, 1H), 7.79 – 7.74 (m, 2H), 7.61 – 7.55 (m, 2H), 7.47 (t, *J* = 7.5 Hz, 2H), 7.42 – 7.34 (m, 2H), 7.26 – 7.22 (m, 1H), 7.18 (d, *J* = 7.5 Hz, 1H), 3.71 – 3.59 (m, 2H), 3.35 (dd, *J* = 16.9, 8.4 Hz, 2H), 2.50 (s, 3H), 2.20 (s, 3H), 1.36 (s, 12H).

**$^{13}$C NMR** (101 MHz, $CDCl_3$) δ 157.0, 154.6, 152.3, 151.3, 150.9, 143.0, 141.1, 139.0, 135.3, 134.5, 129.4, 129.1, 128.6, 128.3, 127.7, 124.8, 122.1, 120.5, 119.4, 84.0, 56.2, 45.3, 44.2, 25.0, 22.2, 16.4.

**HRMS** (ESI/QTOF) m/z: $[M + H]^+$ Calcd for $C_{34}H_{35}BNO_2{}^+$ 500.2755; Found 500.2766.

$[\alpha]_D^{20}$**:** 16.7 (c = 0.12, $CHCl_3$).

**IR (ATR)**: $\tilde{\nu}$ ($cm^{-1}$) =: 3060, 3041, 2977, 2929, 2854, 2368, 2359, 2340, 2312, 2144, 2007, 1993, 1976, 1611, 1572, 1492, 1455, 1415, 1380, 1355, 1316, 1266, 1242, 1217, 1144, 1105, 1081, 1023, 963, 871, 852, 833, 798, 787, 761, 740, 702, 688, 668, 637, 627, 585, 453, 427, 406.

**Melting point**: -

**$R_f$**: 0.42 (ethyl acetate: pentane = 1:4).

**HPLC:** Chiralpak IF 4.6 x 250 mm; hexane:*i*-PrOH 98:2, 1 mL/min, 254 nm; *t*R (major) = 9.2 min, *t*R (minor) = 12.1 min, 87:13 er.

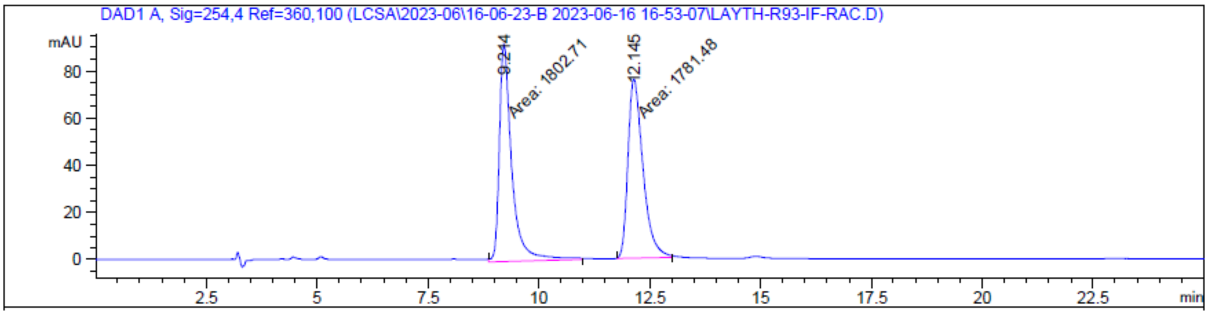


Signal 1: DAD1 A, Sig=254,4 Ref=360,100

| Peak # | RetTime [min] | Type | Width [min] | Area [mAU*s] | Height [mAU] | Area % |
|---|---|---|---|---|---|---|
| 1 | 9.214 | MM | 0.3266 | 1802.70972 | 92.00240 | 50.2962 |
| 2 | 12.145 | MM | 0.3915 | 1781.47778 | 75.84402 | 49.7038 |

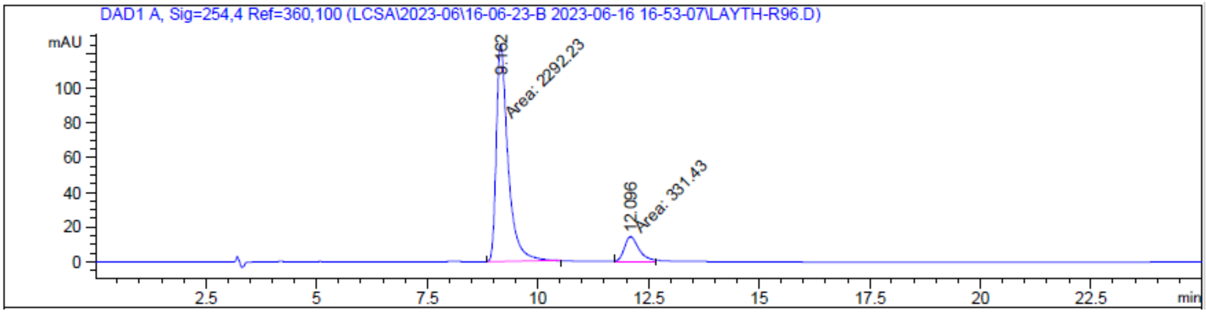


Signal 1: DAD1 A, Sig=254,4 Ref=360,100

| Peak # | RetTime [min] | Type | Width [min] | Area [mAU*s] | Height [mAU] | Area % |
|---|---|---|---|---|---|---|
| 1 | 9.162 | MM | 0.3057 | 2292.23340 | 124.95936 | 87.3677 |
| 2 | 12.096 | MM | 0.3842 | 331.42984 | 14.37927 | 12.6323 |

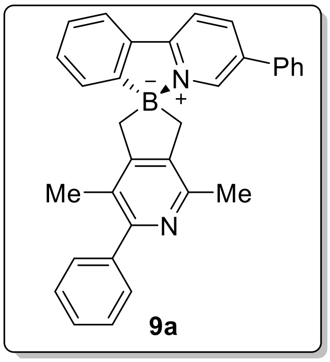

9a

**Name**: (*R*) 8-(tert-butyl)-4',7'-dimethyl-6'-(thiophen-3-yl)-1',3'-dihydro-5λ$^4$,6λ$^4$-spiro[benzo[3,4][1,2]azaborolo[1,5-a]pyridine-6,2'-borolo[3,4-c]pyridine] (**9a**).

**Synthesis**: Following general procedure E, (using **3a** 34.7 mg, 0.1 mmol; **6a** 10.3 mg, 0.1 mmol; **Ni3** 3.6 mg, 3.0 μmol) compound **9a** was obtained in 42.9 mg, 0.0952 mmol, 95% yield and 92:8 er as a brown foam.

**$^1$H NMR** (400 MHz, $CDCl_3$) δ 8.44 – 8.40 (m, 1H), 8.21 (dd, *J* = 8.4, 2.0 Hz, 1H), 8.05 (dd, *J* = 8.5, 0.9 Hz, 1H), 7.91 (d, *J* = 7.8 Hz, 1H), 7.73 (d, *J* = 7.3 Hz, 1H), 7.60 – 7.31 (m, 12H), 2.55 (s, 3H), 2.47 – 2.34 (m, 2H), 2.23 (s, 3H), 1.99 (dd, *J* = 18.0, 7.6 Hz, 2H).

**$^{11}$B NMR** (128 MHz, $CDCl_3$) δ 4.7.

**$^{13}$C NMR** (101 MHz, $CDCl_3$) δ 158.9, 155.8, 154.8, 152.8, 142.1, 141.6, 139.7, 138.8, 136.2, 135.6, 135.5, 131.1, 129.6, 129.6, 129.6, 129.2, 128.0, 127.1, 127.0, 126.9, 126.1, 121.6, 117.9, 108.0, 67.7, 31.4 – 30.0 (m), 29.8 – 28.7 (m), 24.0, 22.4, 16.8.

$[\alpha]_D^{20}$**:** 76.4 (c = 0.24, $CHCl_3$).

**IR (ATR)**: $\tilde{\nu}$ ($cm^{-1}$) =: 3034, 2857, 1620, 1601, 1560, 1506, 1480, 1447, 1413, 1386, 1328, 1284, 1263, 1214, 1156, 1140, 1072, 1052, 1021, 989, 960, 910, 846, 799, 774, 758, 732, 697, 665, 614, 554, 537, 495, 454, 435.

**Melting point**: -

**$R_f$**: 0.10 (ethyl acetate: pentane = 1:4).

**HPLC**: Chiralpak IC; hexane:iPrOH 80:20, 1 mL/min, 254 nm; *t*R (major) = 11.1 min, *t*R (minor) = 9.9 min, 92:8 er.

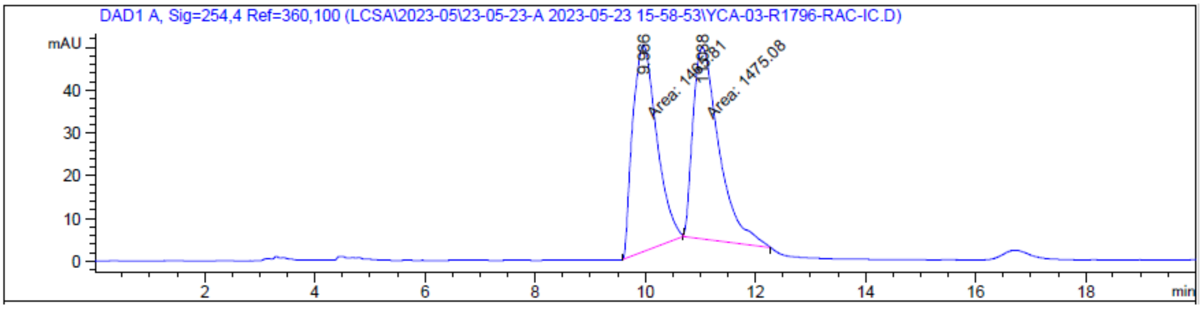


Signal 1: DAD1 A, Sig=254,4 Ref=360,100

| Peak # | RetTime [min] | Type | Width [min] | Area [mAU*s] | Height [mAU] | Area % |
|---|---|---|---|---|---|---|
| 1 | 9.966 | MM | 0.5050 | 1465.80811 | 48.38047 | 49.8424 |
| 2 | 11.038 | MM | 0.5482 | 1475.08044 | 44.84278 | 50.1576 |

Totals : 2940.88855 93.22325

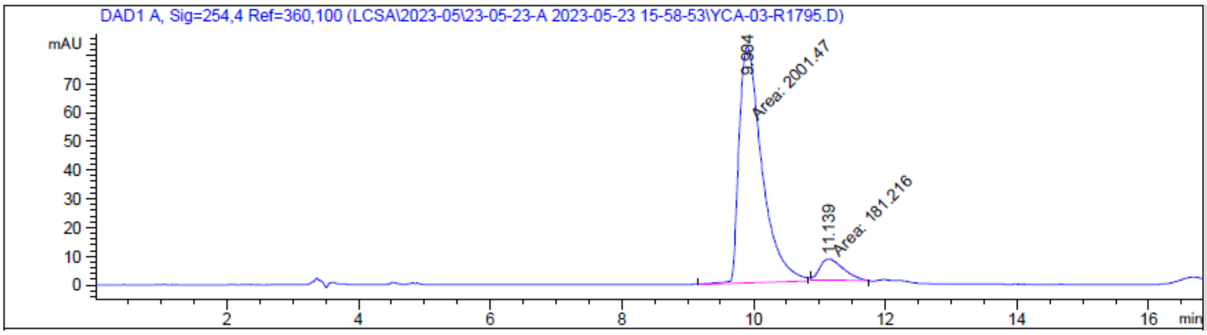


| Peak # | RetTime [min] | Type | Width [min] | Area [mAU*s] | Height [mAU] | Area % |
|---|---|---|---|---|---|---|
| 1 | 9.904 | MM | 0.4047 | 2001.47290 | 82.42200 | 91.6976 |
| 2 | 11.139 | MM | 0.4124 | 181.21611 | 7.32399 | 8.3024 |

Totals : 2182.68901 89.74599

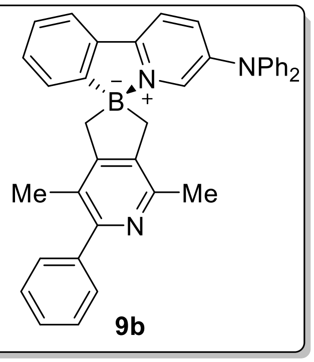

9b

**Name**: (*R*) 4',7'-dimethyl-*N*,*N*,6'-triphenyl-1',3'-dihydro-5λ$^4$,6λ$^4$-spiro[benzo[3,4][1,2]azaborolo[1,5-a]pyridine-6,2'-borolo[3,4-c]pyridin]-3-amine (**9b**).

**Synthesis**: Following general procedure E, (using **3b** 43.8 mg, 0.1 mmol; **6a** 10.3 mg, 0.1 mmol; **Ni3** 3.6 mg, 3.0 μmol) compound **9b** was obtained in 52.5 mg, 0.0969 mmol, 97% yield and 90:10 er, green-yellow foam.

**$^1$H NMR** (400 MHz, $CDCl_3$) δ 7.82 – 7.71 (m, 3H), 7.69 – 7.62 (m, 2H), 7.46 – 7.38 (m, 5H), 7.37 – 7.24 (m, 6H), 7.16 – 7.04 (m, 6H), 2.44 (s, 3H), 2.28 (dd, *J* = 17.8, 13.4 Hz, 2H), 2.11 (s, 3H), 1.78 (dd, *J* = 17.8, 3.0 Hz, 2H).

**$^{11}$B** NMR (128 MHz, $CDCl_3$) δ 6.0.

$^{13}$C NMR (101 MHz, $CDCl_3$) δ 158.7, 154.4, 152.2, 149.7, 145.5, 144.0, 141.2, 136.1, 134.1, 132.0, 130.0, 129.8, 129.5, 129.4, 128.0, 127.0, 126.4, 126.0, 125.3, 124.9, 120.5, 117.8, 30.7 – 29.9 (m), 29.8 – 28.8 (m), 22.3, 16.7.

**HRMS** (ESI/QTOF) m/z: $[M + H]^+$ Calcd for $C_{38}H_{33}BN_3^+$ 542.2762; Found 542.2779.

$[\alpha]_D^{20}$: 73.5 (c = 0.22, $CHCl_3$).

**IR (ATR)**: $\tilde{\nu}$ ($cm^{-1}$) =: 3036, 2961, 2916, 2853, 2787, 2126, 1944, 1801, 1735, 1588, 1557, 1487, 1446, 1412, 1340, 1326, 1285, 1261, 1213, 1171, 1154, 1136, 1074, 1051, 1023, 960, 928, 896, 840, 798, 773, 731, 695, 662, 630, 555, 512, 442, 427.

**Melting point**: -

**$R_f$**: 0.10 (ethyl acetate: pentane = 1:4).

**HPLC**: Chiralpak IC; hexane:iPrOH 90:10, 1 mL/min, 210 nm; *t*R (major) = 16.2 min, *t*R (minor) = 13.9 min, 90:10 er.

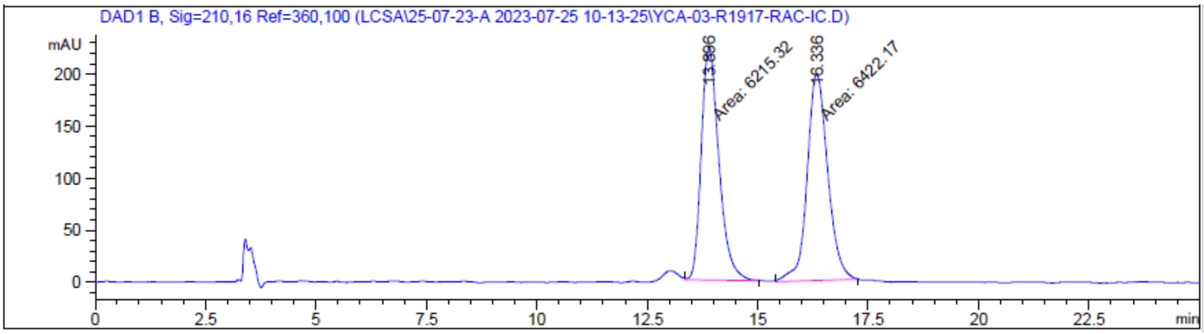


Signal 2: DAD1 B, Sig=210,16 Ref=360,100

| Peak # | RetTime [min] | Type | Width [min] | Area [mAU*s] | Height [mAU] | Area % |
|---|---|---|---|---|---|---|
| 1 | 13.896 | MM | 0.4626 | 6215.32422 | 223.90759 | 49.1816 |
| 2 | 16.336 | MM | 0.5386 | 6422.16748 | 198.72382 | 50.8184 |

Totals : 1.26375e4 422.63141

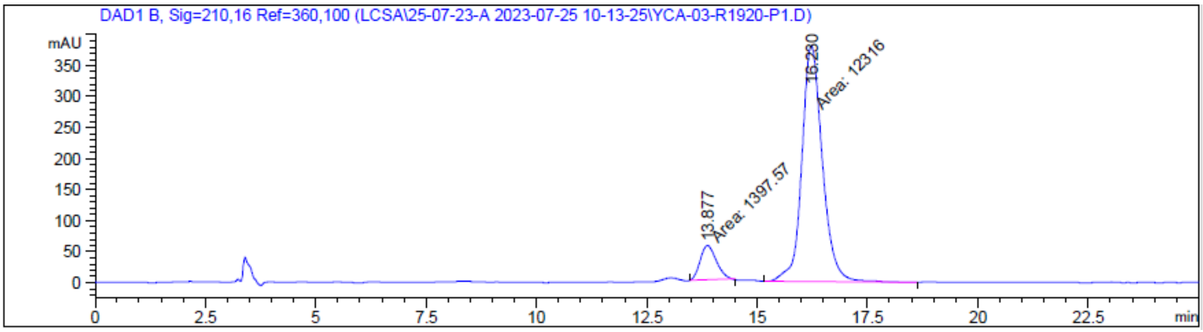


Signal 2: DAD1 B, Sig=210,16 Ref=360,100

| Peak # | RetTime [min] | Type | Width [min] | Area [mAU*s] | Height [mAU] | Area % |
|---|---|---|---|---|---|---|
| 1 | 13.877 | MM | 0.4211 | 1397.56763 | 55.31473 | 10.1912 |
| 2 | 16.230 | MM | 0.5393 | 1.23160e4 | 380.61505 | 89.8088 |

Totals : 1.37135e4 435.92978

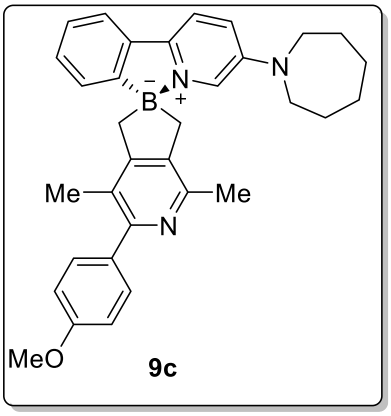


**Name**: (*R*) 3-(azepan-1-yl)-6'-(4-methoxyphenyl)-4',7'-dimethyl-1',3'-dihydro-5λ[4],6λ[4]-spiro[benzo[3,4][1,2]azaborolo[1,5-a]pyridine-6,2'-borolo[3,4-c]pyridine] (**9c**).

**Synthesis**: Following general procedure E, (using **3b** 36.8 mg, 0.1 mmol; **6b** 13.3 mg, 0.1 mmol; **Ni3** 3.6 mg, 3.0 μmol) compound **9c** was obtained in 46.8 mg, 0.0933 mmol, 93% yield and 82:18 er as a pale-yellow foam.

**$^1$H NMR** (400 MHz, $CDCl_3$) δ 7.78 – 7.61 (m, 3H), 7.52 – 7.43 (m, 3H), 7.39 – 7.23 (m, 3H), 7.02 – 6.94 (m, 2H), 3.86 (s, 3H), 3.39 – 3.26 (m, 4H), 2.52 (s, 3H), 2.35 (t, *J* = 18.0 Hz, 2H), 2.22 (s, 3H), 1.86 (d, *J* = 17.6 Hz, 2H), 1.72 – 1.61 (m, 4H), 1.56 – 1.46 (m, 4H).

**$^{11}$B NMR** (128 MHz, $CDCl_3$) δ 4.9.

**$^{13}$C NMR** (101 MHz, $CDCl_3$) δ 159.5, 158.9, 154.2, 152.4, 144.8, 144.4, 141.6, 137.0, 134.8, 130.7, 129.2, 128.7, 126.6, 125.7, 124.2, 122.4, 119.5, 118.1, 113.5, 55.5, 49.5, 31.9 – 30.7 (m), 30.0 – 29.2 (m), 27.0, 26.9, 22.4, 16.9.

**HRMS** (ESI/QTOF) m/z: $[M + H]^+$ Calcd for $C_{33}H_{37}BN_3O^+$ 502.3024; Found 502.3028.

$[\alpha]_D^{20}$**:** 39.3 (c = 0.14, $CHCl_3$).

**IR (ATR)**: $\tilde{\nu}$ ($cm^{-1}$) =: 2924, 2854, 1611, 1558, 1506, 1449, 1409, 1367, 1335, 1290, 1261, 1246, 1203, 1170, 1100, 1051, 1032, 896, 833, 801, 778, 754, 736, 701, 554.

**Melting point**: -

**$R_f$**: 0.14 (ethyl acetate: pentane = 1:1).

**HPLC**: Chiralpak IG; hexane:iPrOH 80:20, 1 mL/min, 210 nm; *t*R (major) = 8.8 min, *t*R (minor) = 15.1 min, 82:18 er.

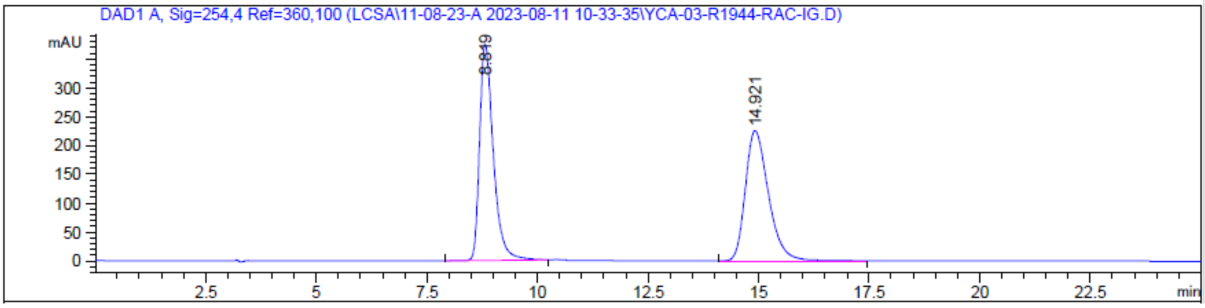


Signal 1: DAD1 A, Sig=254,4 Ref=360,100

| Peak # | RetTime [min] | Type | Width [min] | Area [mAU*s] | Height [mAU] | Area % |
|---|---|---|---|---|---|---|
| 1 | 8.819 | BB | 0.3271 | 8063.20898 | 374.59232 | 49.6951 |
| 2 | 14.921 | BB | 0.5555 | 8162.16211 | 225.89455 | 50.3049 |

Totals : 1.62254e4 600.48686

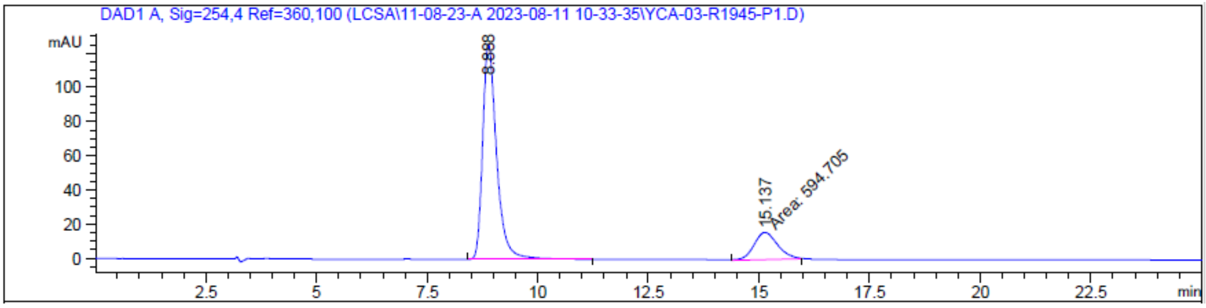


Signal 1: DAD1 A, Sig=254,4 Ref=360,100

| Peak # | RetTime [min] | Type | Width [min] | Area [mAU*s] | Height [mAU] | Area % |
|---|---|---|---|---|---|---|
| 1 | 8.888 | BB | 0.3319 | 2759.85254 | 125.81808 | 82.2717 |
| 2 | 15.137 | MM | 0.6220 | 594.70526 | 15.93559 | 17.7283 |

Totals : 3354.55780 141.75366

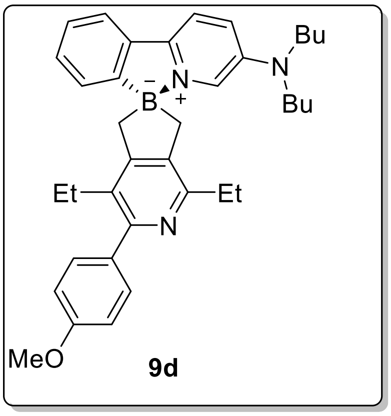


**Name**: (*R*) *N*,*N*-dibutyl-4',7'-diethyl-6'-(4-methoxyphenyl)-1',3'-dihydro-5λ$^4$,6λ$^4$-spiro[benzo[3,4][1,2]azaborolo[1,5-a]pyridine-6,2'-borolo[3,4-c]pyridin]-3-amine (**9d**).

**Synthesis**: Following general procedure E, (using **3d** 42.6 mg, 0.1 mmol; **6b** 13.3 mg, 0.1 mmol; **Ni3** 3.6 mg, 3.0 µmol) compound **9d** was obtained in 52.8 mg, 0.094 mmol, 94% yield and 66:34 er as a pale-yellow foam.

**$^1$H NMR** (400 MHz, $CDCl_3$) δ 7.75 – 7.65 (m, 3H), 7.47 – 7.40 (m, 2H), 7.40 – 7.33 (m, 2H), 7.33 – 7.27 (m, 1H), 7.24 – 7.16 (m, 1H), 6.96 (d, *J* = 8.6 Hz, 2H), 3.86 (s, 3H), 3.12 (dd, *J* = 9.4, 6.4 Hz, 4H), 2.85 (q, *J* = 7.6 Hz, 2H), 2.62 (q, *J* = 7.5 Hz, 2H), 2.47 – 2.37 (m, 2H), 1.97 – 1.86 (m, 2H), 1.42 (p, *J* = 8.3, 7.8 Hz, 4H), 1.30 – 1.15 (m, 7H), 1.07 (t, *J* = 7.4 Hz, 3H), 0.87 (t, *J* = 7.3 Hz, 6H).

**$^{11}$B NMR** (128 MHz, $CDCl_3$) δ 4.9.

**$^{13}$C NMR** (101 MHz, $CDCl_3$) δ 158.8, 157.6, 154.4, 144.4, 143.7, 141.4, 137.2, 135.2, 133.1, 130.4, 129.2, 128.6, 125.8, 124.0, 122.5, 119.5, 118.0, 113.5, 55.4, 51.0, 30.3 – 29.7 (m), 29.4, 29.0, 23.4, 20.2, 14.8, 14.0.

**HRMS** (ESI/QTOF) m/z: $[M + H]^+$ Calcd for $C_{37}H_{47}BN_3O^+$ 560.3807; Found 560.3811.

$[\alpha]_D^{20}$**:** 18.6 (c = 0.35, $CHCl_3$).

**IR (ATR)**: $\tilde{\nu}$ ($cm^{-1}$) =: 2958, 2927, 2870, 1613, 1558, 1507, 1450, 1416, 1368, 1335, 1286, 1263, 1244, 1226, 1208, 1174, 1104, 1059, 1034, 970, 907, 835, 800, 782, 760, 732, 701, 641, 612, 589, 554, 512.

**Melting point**: -

**$R_f$**: 0.12 (ethyl acetate: pentane = 1:4).

**HPLC**: Chiralpak IC; hexane:iPrOH 90:10, 1 mL/min, 254 nm; *t*R (major) = 11.1 min, *t*R (minor) = 13.0 min, 66:34 er.

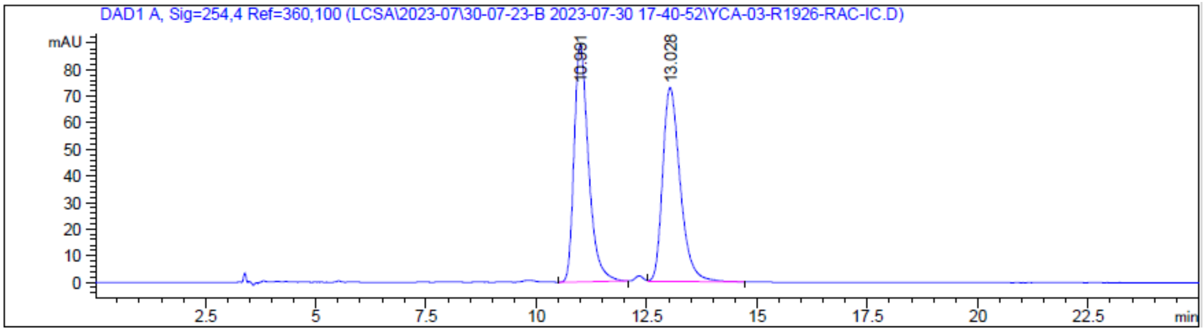


Signal 1: DAD1 A, Sig=254,4 Ref=360,100

| Peak # | RetTime [min] | Type | Width [min] | Area [mAU*s] | Height [mAU] | Area % |
|---|---|---|---|---|---|---|
| 1 | 10.991 | BB | 0.3473 | 2024.53857 | 89.02372 | 50.1660 |
| 2 | 13.028 | VB | 0.4215 | 2011.13855 | 72.99049 | 49.8340 |

Totals : 4035.67712 162.01421

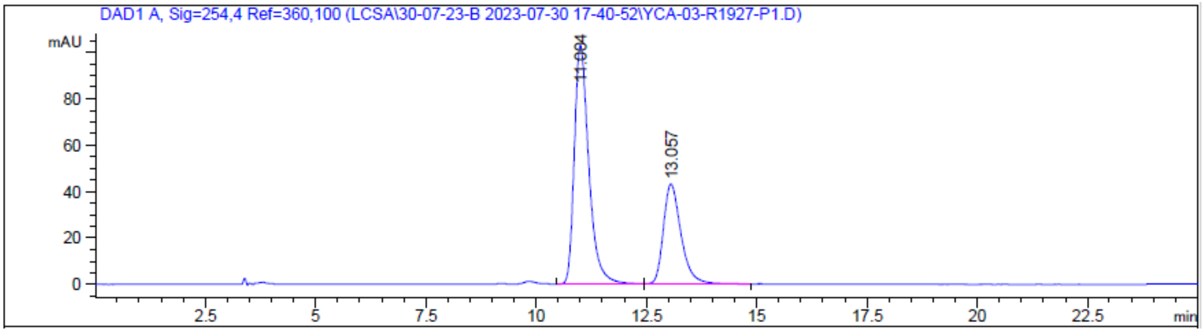


Signal 1: DAD1 A, Sig=254,4 Ref=360,100

| Peak # | RetTime [min] | Type | Width [min] | Area [mAU*s] | Height [mAU] | Area % |
|---|---|---|---|---|---|---|
| 1 | 11.004 | BV | 0.3509 | 2351.47534 | 102.79809 | 66.2873 |
| 2 | 13.057 | VB | 0.4244 | 1195.92664 | 43.01276 | 33.7127 |

Totals : 3547.40198 145.81085

Assignment of absolute configuration of **9e**, **9f**, **9g**, **9h**

Based on the crystal structures of Ni4 and mechanistic studies, the formation of the metallaazirene species is the key intermediate responsible for reactivity and enantioselectivity. In this context, the nitrile substituent ($R^3$), positioned far from the chiral sidearm, has minimal impact on enantioselectivity. Instead, the presence of bulky substituents ($R^1$) on the fluorene, aza-borafluorene, and germafluorene is critical for achieving enantioselectivity. Moreover, the crystal structure of 8m allowed us to determine the absolute configuration of the chiral carbon products and confirm the mechanism proposed.
Regarding the chiral boron compounds, they are expected to form *via* the same mechanism as chiral carbon compounds. Therefore, it is logical that when bulky substituents are present on the pyridine or benzene ring, the resulting products exhibit different nitrile orientations relative to the boron chiral center (see Figure below).

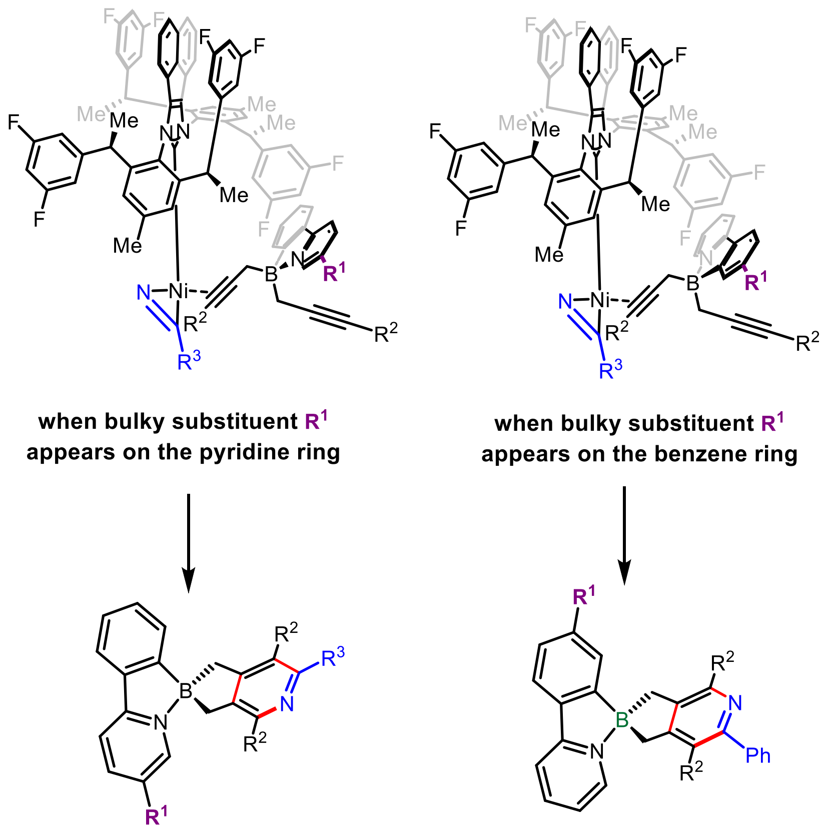

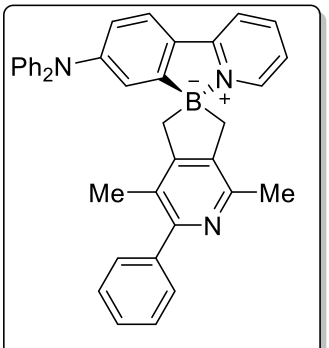

**9e**

**Name**: (*S*) 4',7'-dimethyl-*N*,*N*,6'-triphenyl-1',3'-dihydro-5$\lambda^4$,6$\lambda^4$-spiro[benzo[3,4][1,2]azaborolo[1,5-a]pyridine-6,2'-borolo[3,4-c]pyridin]-8-amine (**9e**).

**Synthesis**: Following general procedure E, (using **3e** 43.8 mg, 0.1 mmol; **6a** 10.3 mg, 0.1 mmol; **Ni3** 3.6 mg, 3.0 µmol) compound **9e** was obtained in 38.5 mg, 0.0711 mmol, 71% yield and 95:5 er as a green-yellow foam.

**$^1$H NMR** (400 MHz, $CDCl_3$) δ 8.05 (dt, *J* = 5.8, 1.3 Hz, 1H), 7.96 – 7.88 (m, 1H), 7.85 – 7.78 (m, 1H), 7.70 (d, *J* = 8.4 Hz, 1H), 7.58 – 7.48 (m, 2H), 7.46 – 7.25 (m, 8H), 7.23 – 7.12 (m, 5H), 7.10 – 7.04 (m, 2H), 7.01 (dd, *J* = 8.4, 2.3 Hz, 1H), 2.52 (s, 3H), 2.32 – 2.17 (m, 5H), 1.86 (dd, *J* = 18.1, 4.3 Hz, 2H).

**$^{11}$B NMR** (128 MHz, $CDCl_3$) δ 6.1.

**$^{13}$C NMR** (101 MHz, $CDCl_3$) δ 156.8, 152.4, 150.8, 147.6, 142.0, 141.6, 140.0, 129.6, 129.6, 129.5, 128.1, 127.3, 127.1, 125.5, 123.7, 122.5, 122.4, 120.9, 120.5, 117.2, 22.1, 16.7.

**HRMS** (ESI/QTOF) m/z: $[M + H]^+$ Calcd for $C_{38}H_{33}BN_3^+$ 542.2762; Found 542.2769.

$[\alpha]_D^{20}$**:** 34.8 (c = 0.11, $CHCl_3$).

**IR (ATR)**: $\tilde{\nu}$ ($cm^{-1}$) =: 3034, 2961, 2922, 2853, 1619, 1589, 1556, 1482, 1467, 1452, 1436, 1415, 1335, 1311, 1262, 1214, 1158, 1074, 1019, 877, 799, 785, 755, 732, 696, 667, 644, 516.

**Melting point**: -

**$R_f$**: 0.29 (methanol: dichloromethane = 1:10).

**HPLC**: Chiralpak ID; hexane:iPrOH 90:10, 1 mL/min, 230 nm; *t*R (major) = 34.4 min, *t*R (minor) = 27.4 min, 95:5 er.

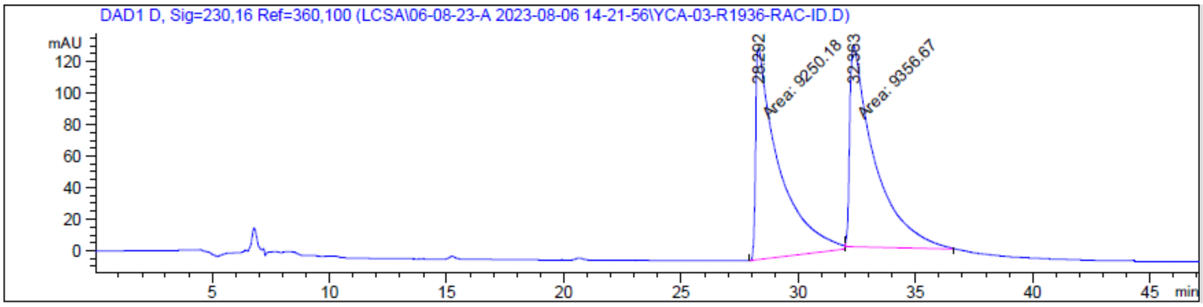


Signal 3: DAD1 D, Sig=230,16 Ref=360,100

| Peak # | RetTime [min] | Type | Width [min] | Area [mAU*s] | Height [mAU] | Area % |
|---|---|---|---|---|---|---|
| 1 | 28.292 | MM | 1.1512 | 9250.17773 | 133.92584 | 49.7138 |
| 2 | 32.363 | MM | 1.2177 | 9356.66992 | 128.06241 | 50.2862 |

Totals : 1.86068e4 261.98825

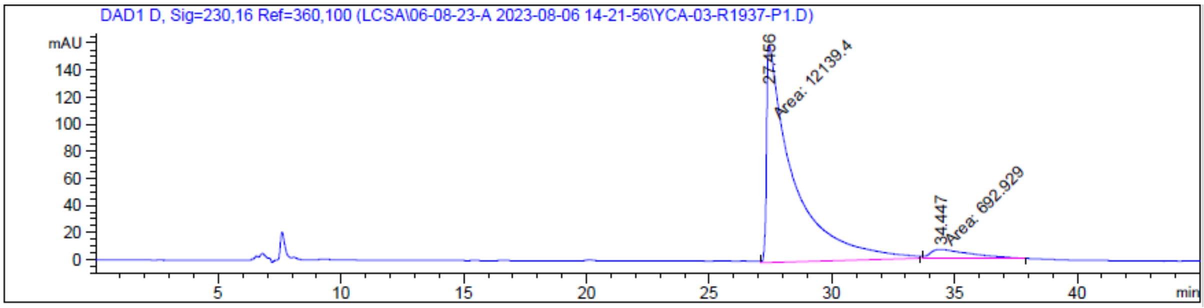


Signal 3: DAD1 D, Sig=230,16 Ref=360,100

| Peak # | RetTime [min] | Type | Width [min] | Area [mAU*s] | Height [mAU] | Area % |
|---|---|---|---|---|---|---|
| 1 | 27.456 | MM | 1.2589 | 1.21394e4 | 160.71796 | 94.6001 |
| 2 | 34.447 | MM | 1.8236 | 692.92908 | 6.33295 | 5.3999 |

Totals : 1.28323e4 167.05090

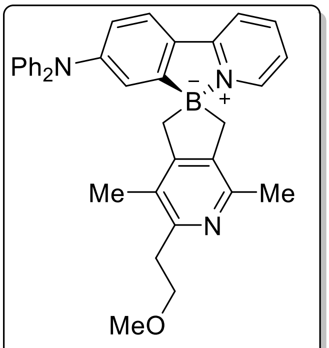

**9f**

**Name**: (*S*) 6'-(2-methoxyethyl)-4',7'-dimethyl-*N*,*N*-diphenyl-1',3'-dihydro-5λ[4],6λ[4]-spiro[benzo[3,4][1,2]azaborolo[1,5-a]pyridine-6,2'-borolo[3,4-c]pyridin]-8-amine (**9f**).

**Synthesis**: Following general procedure E, (using **3e** 43.8 mg, 0.1 mmol; **6n** 8.5 mg, 0.1 mmol; **Ni3** 3.6 mg, 3.0 μmol) compound **9f** was obtained in 39.0 mg, 0.0745 mmol, 75% yield and 99:1 er as a green yellow foam.

**$^1$H NMR** (400 MHz, $CD_2Cl_2$) δ 7.97 (td, *J* = 7.8, 1.6 Hz, 1H), 7.91 – 7.81 (m, 2H), 7.72 (d, *J* = 8.4 Hz, 1H), 7.36 – 7.05 (m, 12H), 6.97 (dd, *J* = 8.4, 2.2 Hz, 1H), 3.79 (t, *J* = 6.5 Hz, 2H), 3.51 – 3.30 (m, 5H), 2.72 (s, 3H), 2.32 – 2.16 (m, 5H), 1.99 (d, *J* = 19.4 Hz, 1H), 1.88 (d, *J* = 18.0 Hz, 1H).

**$^{11}$B NMR** (128 MHz, $CD_2Cl_2$) δ 5.3.

**$^{13}$C NMR** (101 MHz, $CD_2Cl_2$) δ 157.3, 151.3, 148.5, 147.8, 147.6, 145.3, 141.6, 141.1, 130.0, 129.8, 125.8, 124.1, 123.0, 122.1, 121.6, 120.9, 117.9, 71.9, 59.0, 31.2, 17.6, 15.1.

**HRMS** (ESI/QTOF) m/z: $[M + H]^+$ Calcd for $C_{35}H_{35}BN_3O^+$ 524.2868; Found 524.2867.

$[\alpha]_D^{20}$**:** 1.0 (c = 0.16, $CHCl_3$).

**IR (ATR)**: $\tilde{\nu}$ ($cm^{-1}$) =: 2960, 2922, 2898, 2867, 2854, 2160, 2016, 1973, 1620, 1556, 1486, 1469, 1390, 1337, 1312, 1280, 1261, 1236, 1213, 1196, 1023, 985, 872, 863, 814, 795, 757, 734, 698, 623, 610, 602, 593, 579, 499, 478.

**Melting point**: -

**$R_f$**: 0.26 (methanol: dichloromethane = 1:10).

**HPLC**: Chiralpak IC; hexane:iPrOH 80:20, 1 mL/min, 222 nm; *t*R (major) = 19.5 min, *t*R (minor) = 17.5 min, 98.5:1.5 er.

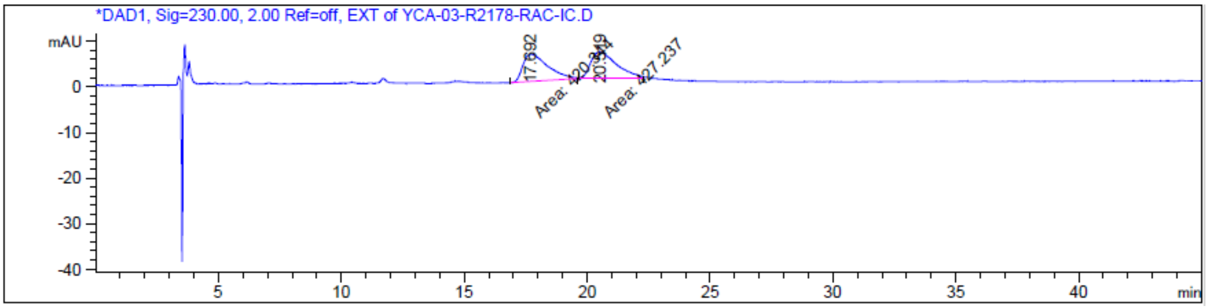


Signal 3: DAD1, Sig=230.00, 2.00 Ref=off, EXT
Signal has been modified after loading from rawdata file!

| Peak # | RetTime [min] | Type | Width [min] | Area [mAU*s] | Height [mAU] | Area % |
|---|---|---|---|---|---|---|
| 1 | 17.692 | MM | 1.1653 | 420.34399 | 6.01201 | 49.5934 |
| 2 | 20.519 | MM | 1.2326 | 427.23721 | 5.77707 | 50.4066 |

Totals : 847.58121 11.78908

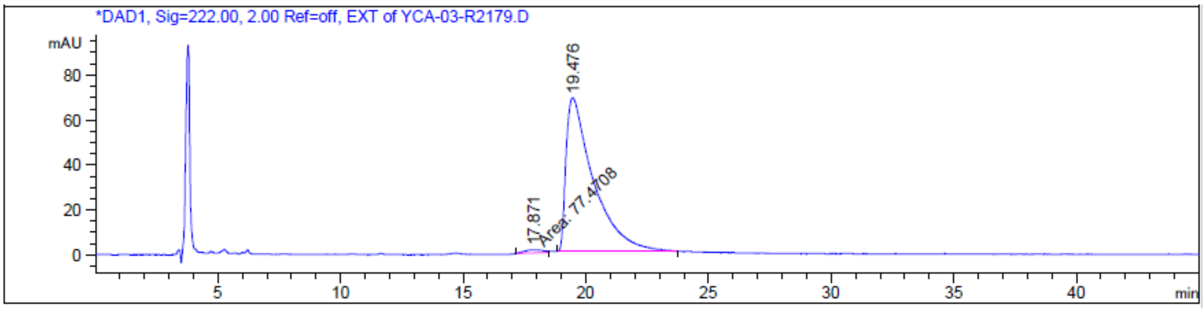


Signal 3: DAD1, Sig=222.00, 2.00 Ref=off, EXT
Signal has been modified after loading from rawdata file!

| Peak # | RetTime [min] | Type | Width [min] | Area [mAU*s] | Height [mAU] | Area % |
|---|---|---|---|---|---|---|
| 1 | 17.871 | MM | 0.8725 | 77.47085 | 1.47985 | 1.4441 |
| 2 | 19.476 | BB | 1.0435 | 5287.13770 | 68.26901 | 98.5559 |

Totals : 5364.60854 69.74886

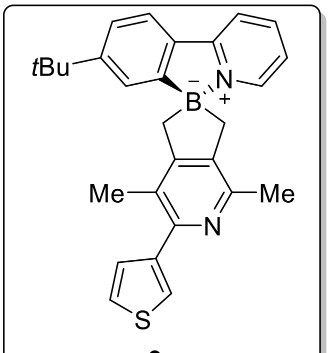


**Name**: (*S*) 8-(tert-butyl)-4',7'-dimethyl-6'-(thiophen-3-yl)-1',3'-dihydro-5$\lambda^4$,6$\lambda^4$-spiro[benzo[3,4][1,2]azaborolo[1,5-a]pyridine-6,2'-borolo[3,4-c]pyridine] (**9g**).

**Synthesis**: Following general procedure E, (using **3f** 32.7 mg, 0.1 mmol; **6p** 10.9 mg, 0.1 mmol; **Ni3** 3.6 mg, 3.0 μmol) compound **9g** was obtained in 37.2 mg, 0.0852 mmol, 85% yield and 96:4 er as a brown foam.

**$^1$H NMR** (400 MHz, $CDCl_3$) δ 8.13 (d, *J* = 5.8 Hz, 1H), 8.00 – 7.90 (m, 2H), 7.85 – 7.78 (m, 2H), 7.52 – 7.47 (m, 1H), 7.46 – 7.40 (m, 2H), 7.39 – 7.32 (m, 1H), 7.25 – 7.19 (m, 1H), 2.53 (s, 3H), 2.39 (dd, *J* = 18.1, 13.3 Hz, 2H), 2.31 (s, 3H), 1.88 (dd, *J* = 18.1, 6.6 Hz, 2H), 1.40 (s, 9H).

**$^{11}$B NMR** (128 MHz, $CDCl_3$) δ 4.4.

**$^{13}$C NMR** (101 MHz, $CDCl_3$) δ 159.3, 157.0, 154.5, 152.8, 149.8, 143.0, 141.9, 141.6, 140.1, 133.5, 129.4, 127.2, 126.2, 124.6, 123.9, 123.7, 121.8, 121.3, 117.6, 35.4, 31.6, 31.1 – 30.3 (m), 30.1 – 28.9 (m), 22.4, 16.8.

**HRMS** (ESI/QTOF) m/z: $[M + H]^+$ Calcd for $C_{28}H_{30}BN_2S^+$ 437.2217; Found 437.2215.

$[\alpha]_D^{20}$: 3.1 (c = 0.16, $CHCl_3$).

**IR (ATR)**: $\tilde{\nu}$ ($cm^{-1}$) = 2961, 2922, 2863, 1620, 1603, 1561, 1475, 1436, 1403, 1362, 1330, 1310, 1262, 1215, 1168, 1159, 1095, 1054, 1016, 980, 893, 861, 844, 827, 785, 762, 733, 700, 673, 645, 548, 480.

**Melting point**: -

**$R_f$**: 0.32 (methanol: dichloromethane = 1:10).

**HPLC**: Chiralpak ID; hexane:iPrOH 90:10, 1 mL/min, 254 nm; *t*R (major) = 9.9 min, *t*R (minor) = 9.1 min, 96:4 er.

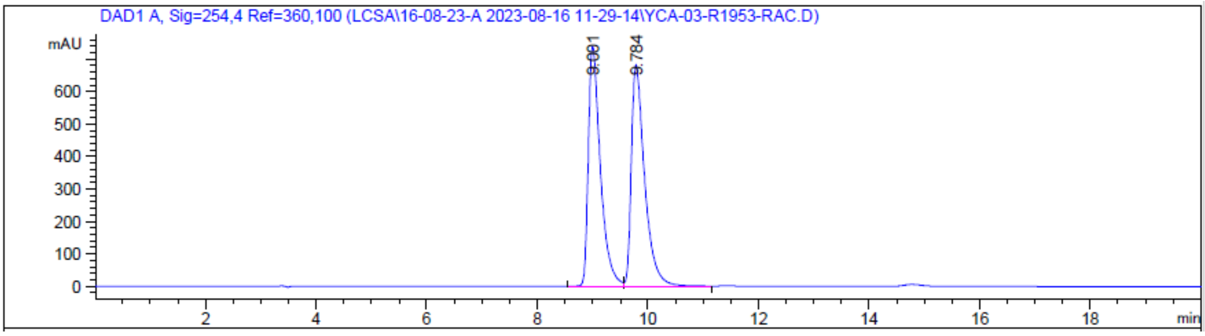


| Peak # | RetTime [min] | Type | Width [min] | Area [mAU*s] | Height [mAU] | Area % |
|---|---|---|---|---|---|---|
| 1 | 9.001 | BV | 0.2268 | 1.12065e4 | 739.46539 | 49.6243 |
| 2 | 9.784 | VB | 0.2507 | 1.13762e4 | 681.56354 | 50.3757 |

Totals : 2.25828e4 1421.02893

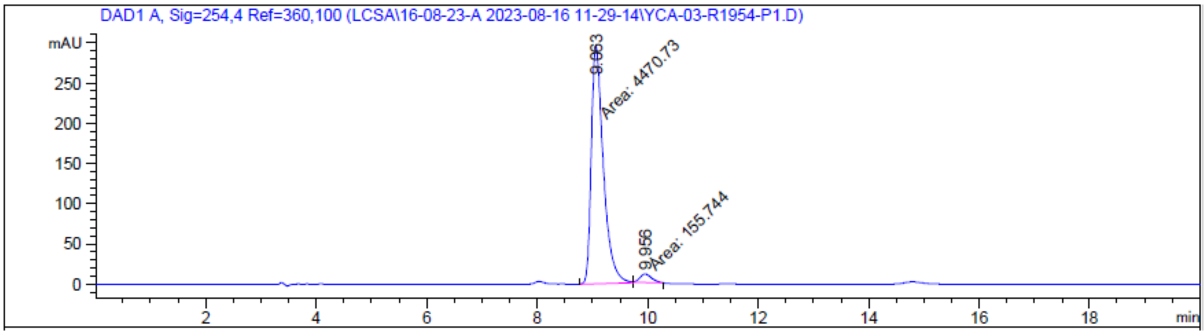


| Peak # | RetTime [min] | Type | Width [min] | Area [mAU*s] | Height [mAU] | Area % |
|---|---|---|---|---|---|---|
| 1 | 9.063 | MM | 0.2498 | 4401.00293 | 293.66156 | 96.3991 |
| 2 | 9.956 | MM | 0.2567 | 164.39714 | 10.67550 | 3.6009 |

Totals : 4565.40007 304.33706

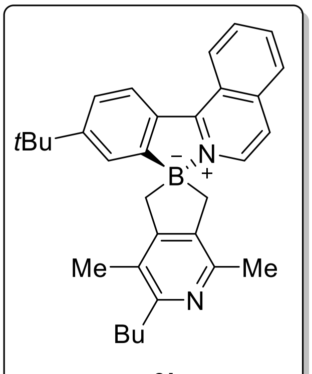


**Name**: (*S*) 10-(tert-butyl)-6'-butyl-4',7'-dimethyl-1',3'-dihydro-7$\lambda^4$,8$\lambda^4$-spiro[benzo[3,4][1,2]azaborolo[5,1-a]isoquinoline-8,2'-borolo[3,4-c]pyridine] **(9h)**.

**Synthesis**: Following general procedure E, (using **3g** 37.8 mg, 0.1 mmol; **6l** 8.3 mg, 0.1 mmol; **Ni3** 3.6 mg, 3.0 μmol) compound **9h** was obtained in 38.6 mg, 0.0838 mmol, 84% yield and 95:5 er as a brown foam.

**$^1$H NMR** (400 MHz, $CDCl_3$) δ 9.15 (d, *J* = 8.5 Hz, 1H), 8.51 (d, *J* = 8.1 Hz, 1H), 8.03 – 7.74 (m, 5H), 7.56 (d, *J* = 6.3 Hz, 1H), 7.50 (dd, *J* = 8.4, 2.1 Hz, 1H), 2.93 (t, *J* = 7.9 Hz, 2H), 2.55 (s, 3H), 2.39 (d, *J* = 18.1 Hz, 2H), 2.26 (s, 3H), 1.97 – 1.83 (m, 2H), 1.76 – 1.67 (m, 2H), 1.55 – 1.46 (m, 2H), 1.42 (s, 9H), 0.99 (t, *J* = 7.4 Hz, 3H).

**$^{11}$B NMR** (128 MHz, $CDCl_3$) δ 3.3.

**$^{13}$C NMR** (101 MHz, $CDCl_3$) δ 156.0, 154.2, 138.0, 135.1, 133.4, 132.4, 129.3, 128.1, 126.7, 126.2, 125.2, 124.0, 120.9, 35.3, 32.3, 31.6, 23.2, 15.0, 14.2.

**HRMS** (ESI/QTOF) m/z: $[M + H]^+$ Calcd for $C_{32}H_{38}BN_2^+$ 461.3123; Found 461.3118.

$[\alpha]_D^{20}$**:** -5.7 (c = 0.15, $CHCl_3$).

**IR (ATR)**: $\tilde{\nu}$ ($cm^{-1}$) = 2867, 2262, 2145, 2075, 2036, 2011, 2000, 1969, 1959, 1669, 1626, 1600, 1568, 1546, 1508, 1465, 1451, 1426, 1392, 1358, 1320, 1293, 1260, 1187, 1163, 1093, 1061, 1036, 1019, 932, 699, 684, 618, 606, 587, 578, 559, 547, 531, 520, 495, 473, 464, 452, 440, 422, 411, 401.

**Melting point**: -

**$R_f$**: 0.35 (methanol: ethyl acetate = 1:10).

**HPLC**: Chiralpak IC; hexane:iPrOH 90:10, 1 mL/min, 254 nm; *t*R (major) = 16.3 min, *t*R (minor) = 13.4 min, 95:5 er.

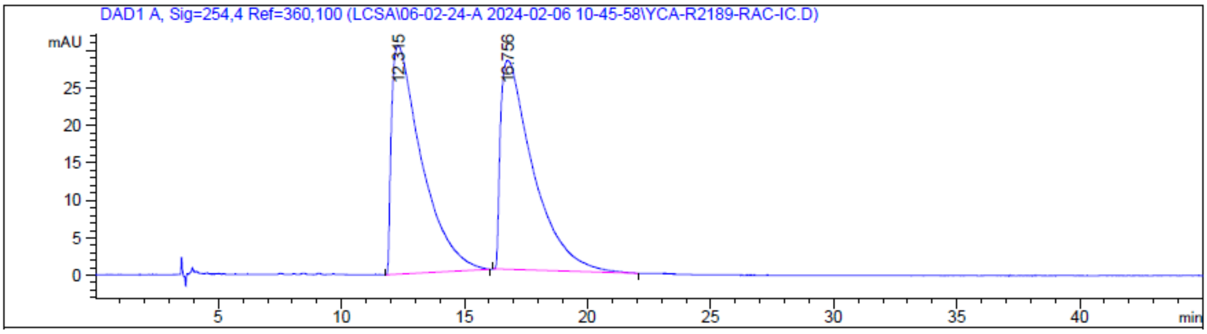


Signal 1: DAD1 A, Sig=254,4 Ref=360,100

| Peak # | RetTime [min] | Type | Width [min] | Area [mAU*s] | Height [mAU] | Area % |
|---|---|---|---|---|---|---|
| 1 | 12.315 | BB | 1.1472 | 2594.73584 | 30.54063 | 50.1953 |
| 2 | 16.756 | BB | 1.2101 | 2574.54639 | 27.91101 | 49.8047 |

Totals : 5169.28223 58.45164

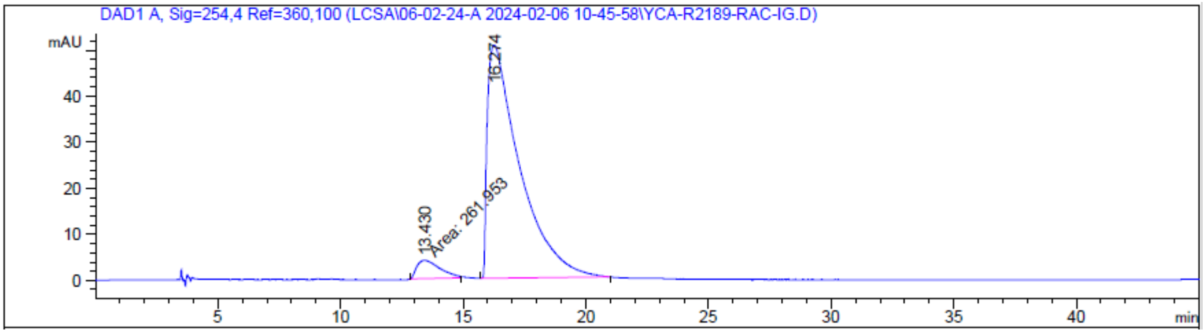


Signal 1: DAD1 A, Sig=254,4 Ref=360,100

| Peak # | RetTime [min] | Type | Width [min] | Area [mAU*s] | Height [mAU] | Area % |
|---|---|---|---|---|---|---|
| 1 | 13.430 | MM | 1.0875 | 261.95340 | 4.01476 | 5.3454 |
| 2 | 16.274 | BB | 1.2354 | 4638.62939 | 50.51134 | 94.6546 |

Totals : 4900.58279 54.52610

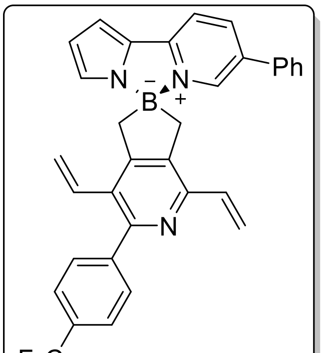

**9i**

**Name**: (*R*) 8'-phenyl-6-(4-(trifluoromethyl)phenyl)-4,7-divinyl-1,3-dihydro-2λ$^4$,6λ$^4$-spiro[borolo[3,4-c]pyridine-2,5'-pyrrolo[1',2':3,4][1,3,2]diazaborolo[1,5-a]pyridine] (**9i**).

**Synthesis**: Following general procedure E, (using **3h** 36.1 mg, 0.1 mmol; **6d** 17.1 mg, 0.1 mmol; **Ni3** 3.6 mg, 3.0 μmol) compound **9i** was obtained in 51.6 mg, 0.0971 mmol, 97% yield and 84:16 er as a yellow foam.

**$^1$H NMR** (400 MHz, $CD_2Cl_2$) δ 8.16 (d, *J* = 2.9 Hz, 1H), 8.11 (dd, *J* = 8.5, 2.0 Hz, 1H), 7.79 (d, *J* = 8.1 Hz, 2H), 7.73 – 7.64 (m, 3H), 7.52 – 7.39 (m, 5H), 7.16 – 7.02 (m, 2H), 6.80 (d, *J* = 2.5 Hz, 1H), 6.67 (dd, *J* = 17.9, 11.7 Hz, 1H), 6.44 (dd, *J* = 3.7, 2.1 Hz, 1H), 6.37 (dd, *J* = 17.3, 2.5 Hz, 1H), 5.54 – 5.33 (m, 3H), 2.41 – 2.25 (m, 4H).

**$^{11}$B NMR** (128 MHz, $CD_2Cl_2$) δ 7.4.

**$^{13}$C NMR** (101 MHz, $CD_2Cl_2$) δ 157.1, 153.6, 150.5, 147.9, 146.0, 141.4, 140.1, 138.5, 136.0, 135.1, 134.8, 133.5, 131.4, 130.9, 130.0, 129.8 – 129.0 (m), 127.0, 126.3, 125.0 (q, *J* = 3.7 Hz), 124.4, 123.6, 120.0, 118.0, 116.8, 115.1, 106.8, 33.4 – 31.9 (m), 29.1 – 27.8 (m).

**$^{19}$F NMR** (376 MHz, $CD_2Cl_2$) δ -62.6.

**HRMS** (ESI/QTOF) m/z: $[M + H]^+$ Calcd for $C_{33}H_{26}BF_3N_3^+$ 532.2166; Found 532.2176.
$[\alpha]_D^{20}$**:** 6.9 (c = 0.24, $CHCl_3$).

**IR (ATR)**: $\tilde{\nu}$ ($cm^{-1}$) =: 3031, 2963, 2922, 2857, 1631, 1572, 1537, 1467, 1449, 1395, 1323, 1261, 1222, 1201, 1162, 1118, 1064, 1047, 1016, 923, 875, 848, 798, 764, 735, 695, 664, 643, 623, 606, 584, 552, 499, 482, 464, 406.

**Melting point**: -

**R$_f$**: 0.66 (ethyl acetate: pentane = 1:4).

**HPLC**: Chiralpak IB; hexane:iPrOH 90:10, 1 mL/min, 254 nm; *t*R (major) = 8.0 min, *t*R (minor) = 7.7 min, 84:16 er.

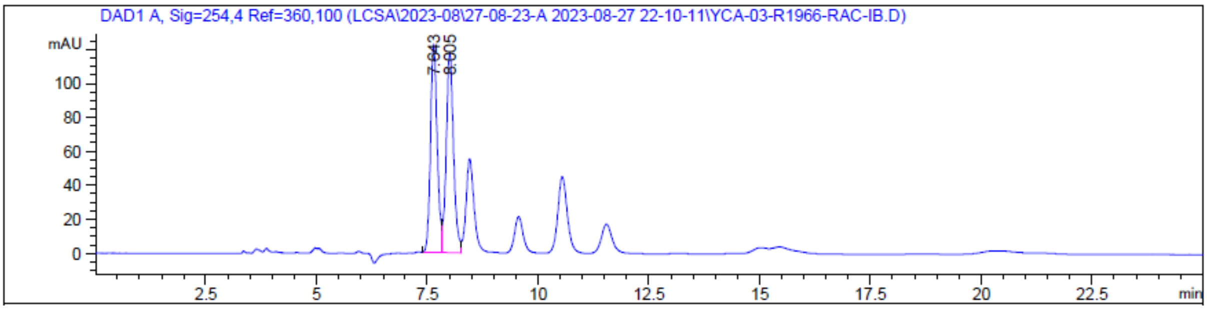


Signal 1: DAD1 A, Sig=254,4 Ref=360,100

| Peak # | RetTime [min] | Type | Width [min] | Area [mAU*s] | Height [mAU] | Area % |
|---|---|---|---|---|---|---|
| 1 | 7.643 | BV | 0.1588 | 1286.22742 | 122.21471 | 48.8435 |
| 2 | 8.005 | VV | 0.1714 | 1347.13477 | 117.82655 | 51.1565 |

Totals : 2633.36218 240.04127

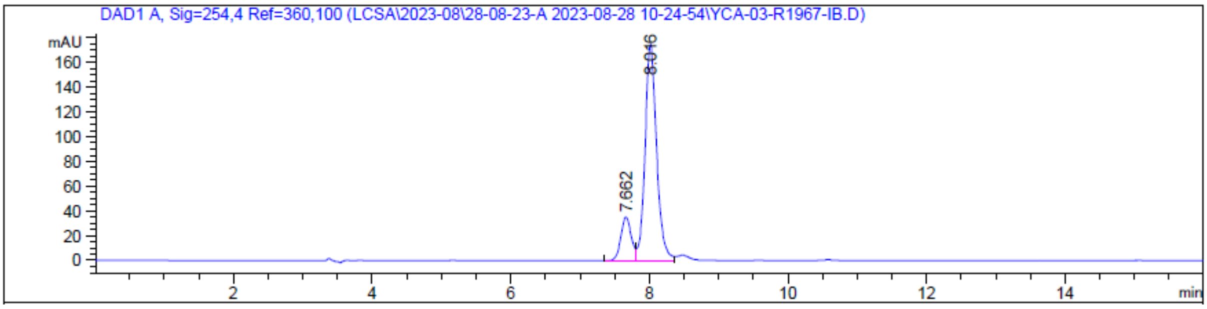


Signal 1: DAD1 A, Sig=254,4 Ref=360,100

| Peak # | RetTime [min] | Type | Width [min] | Area [mAU*s] | Height [mAU] | Area % |
|---|---|---|---|---|---|---|
| 1 | 7.662 | BV | 0.1605 | 371.15741 | 35.36146 | 15.5184 |
| 2 | 8.016 | VV | 0.1748 | 2020.56384 | 174.81342 | 84.4816 |

Totals : 2391.72125 210.17488

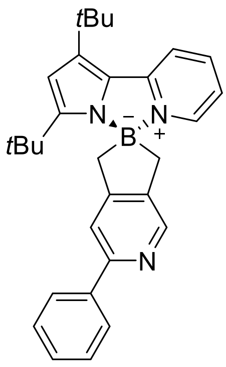


**9j**

**Name**: (*S*) 1',3'-di-tert-butyl-6-phenyl-1,3-dihydro-2l4,6'l4-spiro[borolo[3,4-c]pyridine-2,5'-pyrrolo[1',2':3,4][1,3,2]diazaborolo[1,5-a]pyridine] (**9j**).

**Synthesis**: Following general procedure E, (using **2h** 34.3 mg, 0.1 mmol; **6a** 10.3 mg, 0.1 mmol; **Ni3** 3.6 mg, 3.0 μmol) compound **9j** was obtained in 43.7 mg, 0.0974 mmol, 97% yield and 66:34 er as a green-yellow foam.

**$^1$H NMR** (400 MHz, $CD_2Cl_2$) δ 8.53 (s, 1H), 8.03 (dd, *J* = 8.4, 1.4 Hz, 2H), 7.84 – 7.62 (m, 4H), 7.53 – 7.41 (m, 2H), 7.41 – 7.32 (m, 1H), 6.90 – 6.82 (m, 1H), 6.24 (s, 1H), 2.60 (dd, *J* = 19.3, 16.4 Hz, 2H), 1.97 (dd, *J* = 18.8, 8.4 Hz, 2H), 1.46 (s, 9H), 1.31 (s, 9H).

**$^{11}$B NMR** (128 MHz, $CD_2Cl_2$) δ 5.9.

**$^{13}$C NMR** (101 MHz, $CD_2Cl_2$) δ 158.6, 154.1, 149.8, 149.1, 147.5, 143.1, 140.8, 140.1, 139.6, 137.4, 128.9, 128.4, 127.4, 127.0, 120.1, 118.0, 117.5, 111.8, 33.7, 32.1, 31.5, 31.0.

**HRMS** (ESI/QTOF) m/z: $[M + H]^+$ Calcd for $C_{30}H_{35}BN_3^+$ 448.2919; Found 448.2917.
$[\alpha]_D^{20}$**:** 18.2 (c = 0.22, $CHCl_3$).

**IR (ATR)**: $\tilde{\nu}$ ($cm^{-1}$) = 2958, 2920, 2869, 1620, 1591, 1546, 1519, 1488, 1470, 1446, 1405, 1390, 1360, 1345, 1323, 1261, 1245, 1211, 1187, 1160, 1083, 1022, 992, 975, 943, 919, 889, 867, 803, 769, 755, 734, 694, 615, 580, 452, 417, 403.

**Melting point**: -

**$R_f$**: 0.25 (ethyl acetate: pentane = 1:4).

**HPLC**: Chiralpak IG; hexane:iPrOH 95:5, 1 mL/min, 254 nm; *t*R (major) = 11.9 min, *t*R (minor) = 10.5 min, 66:34 er.

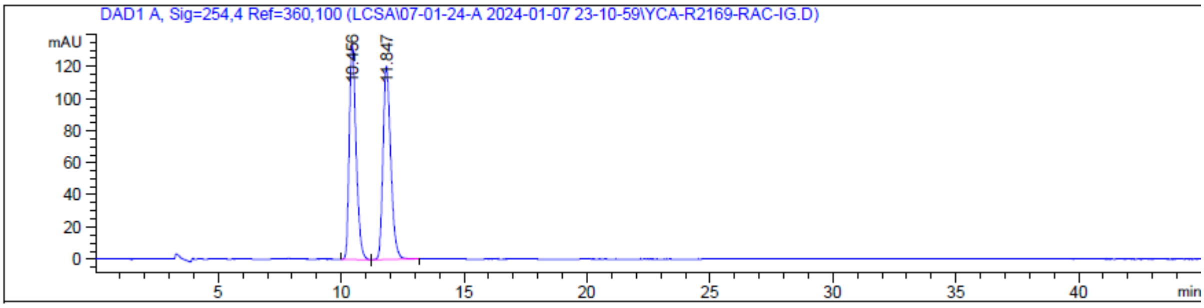


Signal 1: DAD1 A, Sig=254,4 Ref=360,100

| Peak # | RetTime [min] | Type | Width [min] | Area [mAU*s] | Height [mAU] | Area % |
|---|---|---|---|---|---|---|
| 1 | 10.556 | BB | 0.2966 | 1462.95752 | 75.31725 | 33.6236 |
| 2 | 11.976 | BB | 0.3364 | 2888.02881 | 131.42720 | 66.3764 |

Totals : 4350.98633 206.74445

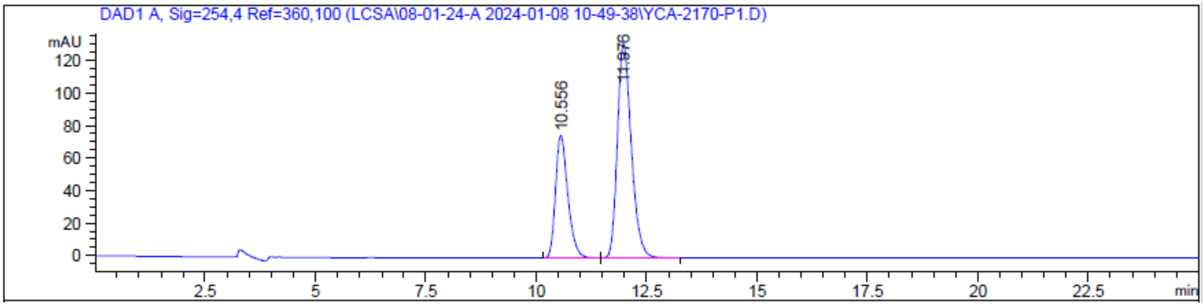


Signal 1: DAD1 A, Sig=254,4 Ref=360,100

| Peak # | RetTime [min] | Type | Width [min] | Area [mAU*s] | Height [mAU] | Area % |
|---|---|---|---|---|---|---|
| 1 | 10.556 | BB | 0.2966 | 1462.95752 | 75.31725 | 33.6236 |
| 2 | 11.976 | BB | 0.3364 | 2888.02881 | 131.42720 | 66.3764 |

Totals : 4350.98633 206.74445

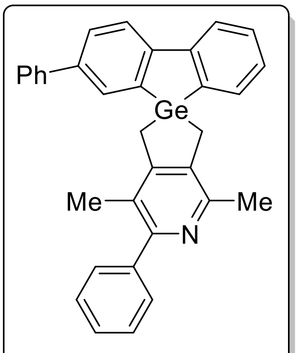


**10a**

**Name**: 4',7'-dimethyl-3,6'-diphenyl-1',3'-dihydrospiro[dibenzo[b,d]germole-5,2'-germolo[3,4-c]pyridine] (**10a**).

**Synthesis**: Following general procedure E compound, (using **5a** 40.7 mg, 0.1 mmol; **6a** 10.3 mg, 0.1 mmol; **Ni3** 3.6 mg, 3.0 μmol) **10a** was obtained in 43.2 mg, 0.0846 mmol, 85% yield and 50:50 er as a pink foam.

**$^{1}$H NMR** (400 MHz, $CDCl_3$) δ 7.99 (dd, *J* = 12.6, 7.9 Hz, 2H), 7.91 (d, *J* = 1.9 Hz, 1H), 7.78 – 7.62 (m, 4H), 7.58 – 7.32 (m, 10H), 2.71 (d, *J* = 20.1 Hz, 4H), 2.64 (s, 3H), 2.31 (s, 3H).

**$^{13}$C NMR** (101 MHz, $CDCl_3$) δ 155.7, 154.6, 151.6, 146.3, 145.6, 141.5, 141.0, 140.8, 138.1, 137.4, 134.8, 133.4, 131.9, 130.5, 129.4, 129.3, 129.0, 128.3, 128.2, 128.0, 127.6, 127.6, 127.1, 122.1, 121.9, 23.9, 19.5, 18.4, 17.8.

**HRMS** (APCI/QTOF) m/z: $[M + H]^+$ Calcd for $C_{33}H_{28}GeN^+$ 512.1428; Found 512.1423.

$[\boldsymbol{\alpha}]_{\boldsymbol{D}}^{\mathbf{20}}$**: -**

**IR (ATR)**: $\tilde{\boldsymbol{\nu}}$ ($cm^{-1}$) = 3057, 2915, 1598, 1551, 1495, 1442, 1412, 1265, 1204, 1143, 1121, 1074, 1053, 1034, 1022, 969, 916, 897, 839, 803, 780, 758, 736, 716, 700, 643, 576, 534, 433, 402.

**Melting point**: -

**$R_f$**: 0.55 (ethyl acetate: pentane = 1:4).

**HPLC**: Chiralpak ID; hexane:iPrOH 99:1, 1 mL/min, 254 nm; *t*R (major) = 30.9 min, *t*R (minor) = 36.3 min, 50:50 er.

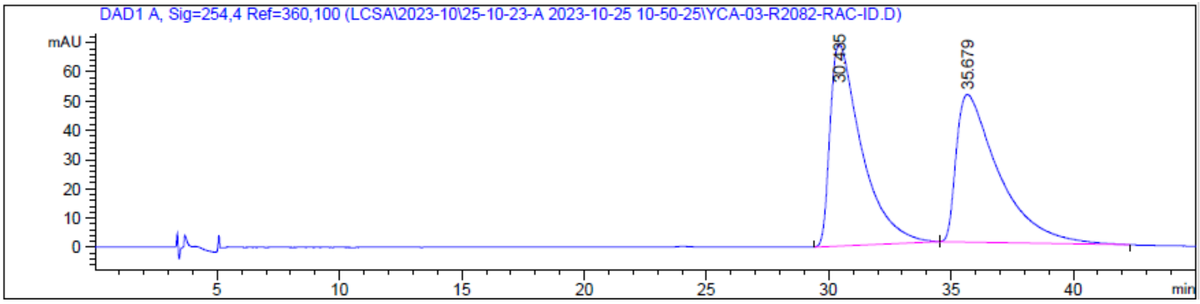


Signal 1: DAD1 A, Sig=254,4 Ref=360,100

| Peak # | RetTime [min] | Type | Width [min] | Area [mAU*s] | Height [mAU] | Area % |
|---|---|---|---|---|---|---|
| 1 | 30.435 | BB | 1.2627 | 6193.31104 | 69.16299 | 50.3891 |
| 2 | 35.679 | BB | 1.6076 | 6097.65723 | 50.53854 | 49.6109 |

Totals : 1.22910e4 119.70153

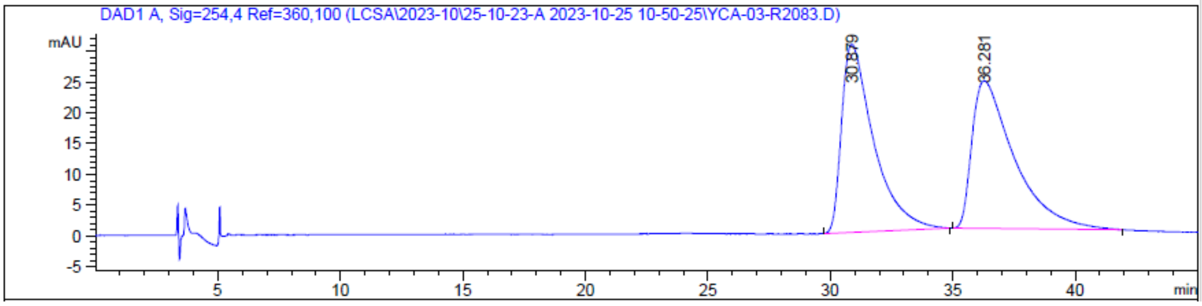


Signal 1: DAD1 A, Sig=254,4 Ref=360,100

| Peak # | RetTime [min] | Type | Width [min] | Area [mAU*s] | Height [mAU] | Area % |
|---|---|---|---|---|---|---|
| 1 | 30.879 | BB | 1.2320 | 2789.38965 | 30.52685 | 49.1761 |
| 2 | 36.281 | BB | 1.5831 | 2882.85571 | 23.93640 | 50.8239 |

Totals : 5672.24536 54.46325

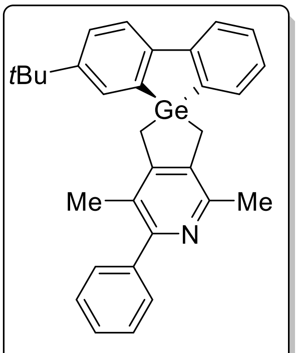


**10b**

**Name**: (*R*)-3-(tert-butyl)-4',7'-dimethyl-6'-phenyl-1',3'-dihydrospiro[dibenzo[b,d]germole-5,2'-germolo[3,4-c]pyridine] (**10b**).

**Synthesis**: Following general procedure E, (using **5b** 38.7 mg, 0.1 mmol; **6a** 10.3 mg, 0.1 mmol; **Ni3** 3.6 mg, 3.0 μmol) compound **10b** was obtained in 44.7 mg, 0.0911 mmol, 91% yield and 80:20 er as a pink foam.

**$^1$H NMR** (400 MHz, $CDCl_3$) δ 7.89 (dd, *J* = 12.4, 8.1 Hz, 2H), 7.69 (d, *J* = 2.1 Hz, 1H), 7.64 (d, *J* = 7.3 Hz, 1H), 7.56 – 7.50 (m, 3H), 7.50 – 7.42 (m, 3H), 7.41 – 7.35 (m, 1H), 7.30 (td, *J* = 7.3, 1.1 Hz, 1H), 2.77 – 2.58 (m, 7H), 2.31 (s, 3H), 1.37 (s, 9H).

**$^{13}$C NMR** (101 MHz, $CDCl_3$) δ 155.7, 154.6, 151.8, 151.3, 146.6, 144.0, 141.5, 137.4, 137.1, 135.1, 133.2, 130.3, 130.0, 129.4, 128.3, 128.1, 127.8, 127.6, 127.6, 121.5, 121.4, 35.0, 31.6, 23.9, 19.6, 18.5, 17.8.

**HRMS** (APCI/QTOF) m/z: $[M + H]^+$ Calcd for $C_{31}H_{32}GeN^+$ 492.1741; Found 492.1740.

$[\boldsymbol{\alpha}]_D^{20}$**:** 10.0 (c = 0.15, $CHCl_3$).

**IR (ATR)**: $\tilde{\nu}$ ($cm^{-1}$) = 3053, 2961, 2864, 1550, 1495, 1475, 1449, 1435, 1412, 1362, 1259, 1203, 1142, 1092, 1070, 1022, 968, 894, 868, 833, 800, 777, 767, 734, 700, 642, 626, 604, 575, 535, 443.

**Melting point**: -

**$R_f$**: 0.59 (ethyl acetate: pentane = 1:4).

**HPLC**: Chiralpak ID; hexane:iPrOH 99:1, 1 mL/min, 254 nm; *t*R (major) = 12.2 min, *t*R (minor) = 16.7 min, 80:20 er.

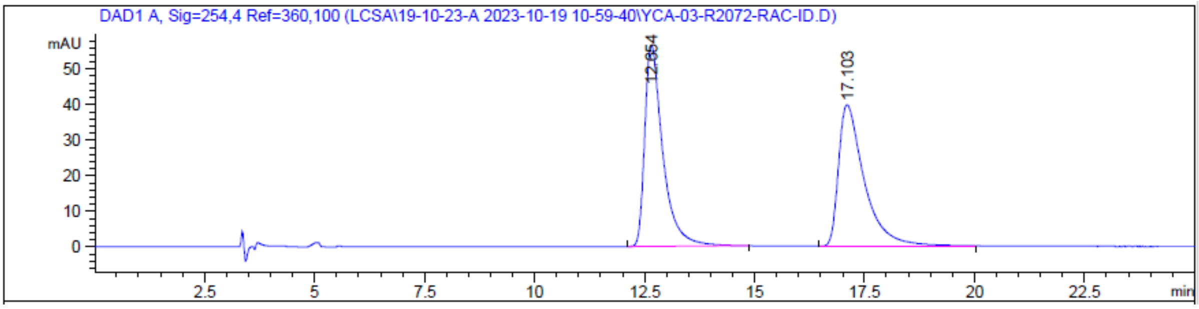


Signal 1: DAD1 A, Sig=254,4 Ref=360,100

| Peak # | RetTime [min] | Type | Width [min] | Area [mAU*s] | Height [mAU] | Area % |
|---|---|---|---|---|---|---|
| 1 | 12.654 | BB | 0.4239 | 1622.81116 | 56.69454 | 50.0419 |
| 2 | 17.103 | BB | 0.5988 | 1620.09155 | 39.81068 | 49.9581 |

Totals : 3242.90271 96.50523

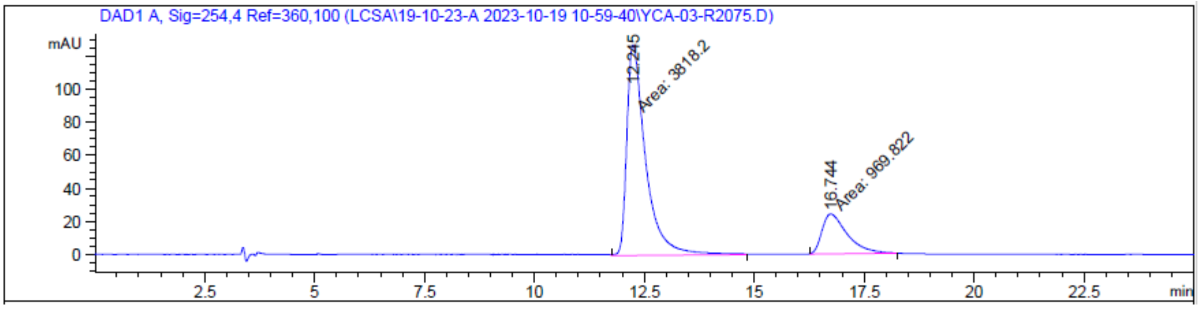


Signal 1: DAD1 A, Sig=254,4 Ref=360,100

| Peak # | RetTime [min] | Type | Width [min] | Area [mAU*s] | Height [mAU] | Area % |
|---|---|---|---|---|---|---|
| 1 | 12.245 | MM | 0.4997 | 3818.20044 | 127.34927 | 79.7448 |
| 2 | 16.744 | MM | 0.6691 | 969.82178 | 24.15588 | 20.2552 |

Totals : 4788.02222 151.50516

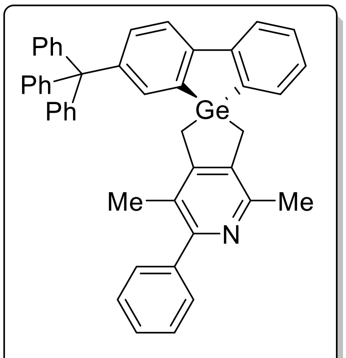


**10c**

**Name**: (*R*)-4',7'-dimethyl-6'-phenyl-3-trityl-1',3'-dihydrospiro[dibenzo[b,d]germole-5,2'-germolo[3,4-c]pyridine] (**10c**).

**Synthesis**: reaction was carried out as 0.05 mmol scale, following general procedure E, (using **5c** 28.7 mg, 0.05 mmol; **6a** 5.1 mg, 0.05 mmol; **Ni3** 1.8 mg, 3.0 μmol) compound **10c** was obtained in 32.1 mg, 0.0474 mmol, 95% yield and 86:14 er as a pink foam.

**$^1$H NMR** (400 MHz, $CD_2Cl_2$) δ 7.90 (d, *J* = 7.9 Hz, 1H), 7.83 (d, *J* = 8.3 Hz, 1H), 7.67 – 7.59 (m, 2H), 7.54 – 7.11 (m, 23H), 2.76 – 2.42 (m, 7H), 2.25 (s, 3H).

**$^{13}$C NMR** (101 MHz, $CD_2Cl_2$) δ 155.5, 154.5, 151.9, 147.4, 147.3, 146.4, 144.6, 141.9, 138.3, 136.9, 135.8, 135.1, 133.6, 133.5, 131.3, 130.5, 129.6, 128.3, 128.2, 128.1, 128.1, 127.7, 126.3, 121.9, 121.1, 65.5, 23.7, 19.7, 18.6, 17.7.

**HRMS** (APCI/QTOF) m/z: $[M + H]^+$ Calcd for $C_{46}H_{38}GeN^+$ 678.2211; Found 678.2216.

$[\alpha]_D^{20}$**:** 11.1 (c = 0.12, $CHCl_3$).

**IR (ATR)**: $\tilde{\nu}$ ($cm^{-1}$) = 3059, 2959, 2922, 2853, 2165, 1597, 1550, 1492, 1445, 1413, 1385, 1260, 1089, 1022, 866, 799, 767, 741, 701, 634, 434, 400.

**Melting point**: -

**$R_f$**: 0.41 (ethyl acetate: pentane = 1:4).

**HPLC**: Chiralpak IC; hexane:iPrOH 98:2, 1 mL/min, 254 nm; *t*R (major) = 21.6 min, *t*R (minor) = 19.1 min, 86:14 er.

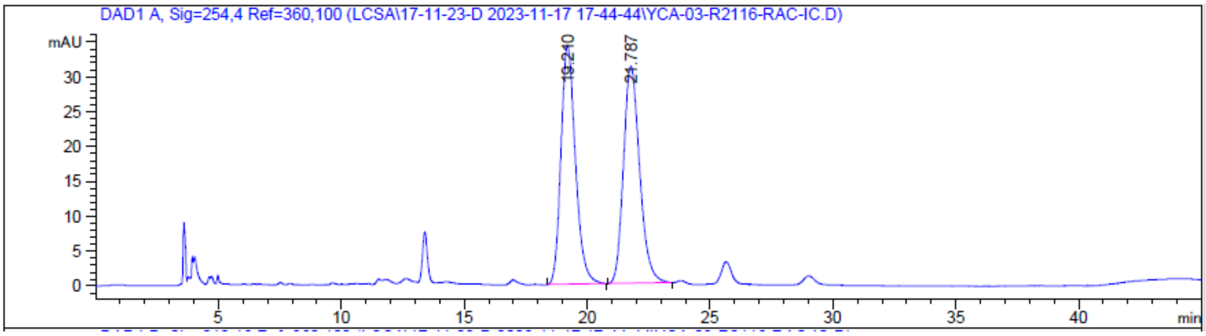


Signal 1: DAD1 A, Sig=254,4 Ref=360,100

| Peak # | RetTime [min] | Type | Width [min] | Area [mAU*s] | Height [mAU] | Area % |
|---|---|---|---|---|---|---|
| 1 | 19.210 | BB | 0.6210 | 1397.96399 | 34.19432 | 49.5837 |
| 2 | 21.787 | BB | 0.6916 | 1421.44104 | 31.07666 | 50.4163 |

Totals : 2819.40503 65.27098

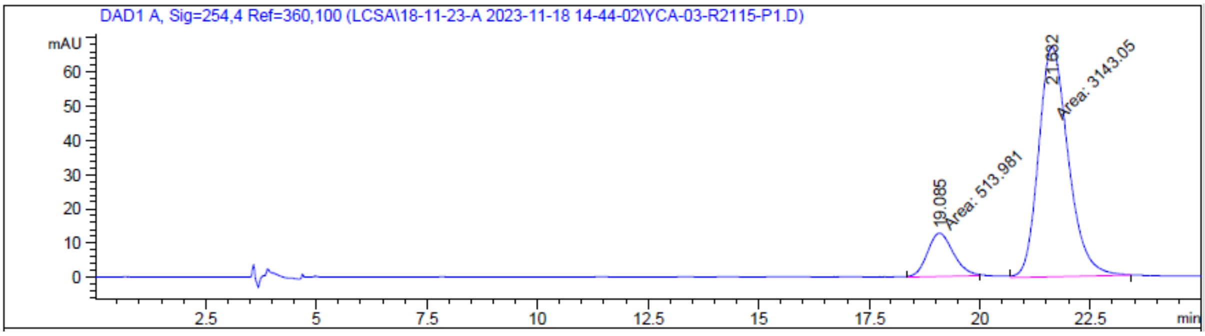


Signal 1: DAD1 A, Sig=254,4 Ref=360,100

| # | [min] | | [min] | [mAU*s] | [mAU] | % |
|---|---|---|---|---|---|---|
| 1 | 19.085 | MM | 0.6777 | 513.98120 | 12.64048 | 14.0546 |
| 2 | 21.632 | MM | 0.7775 | 3143.04541 | 67.37550 | 85.9454 |

Totals : 3657.02661 80.01598

**Silicon centered chiral spiro-skeleton construction.**

Compound **4a**, **4b** cannot deliver the desired product, starting material decompositions were observed.

**Synthetic applications.**

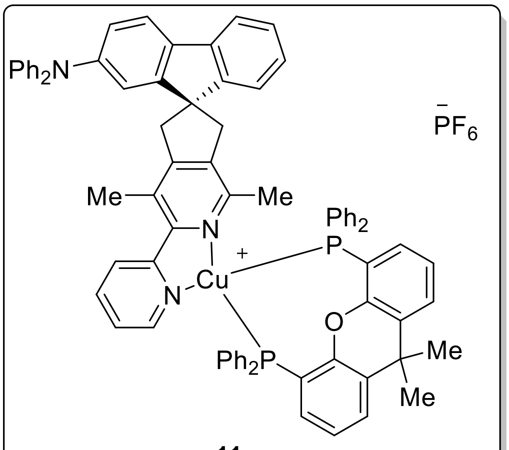


**11a**

**Synthesis**:

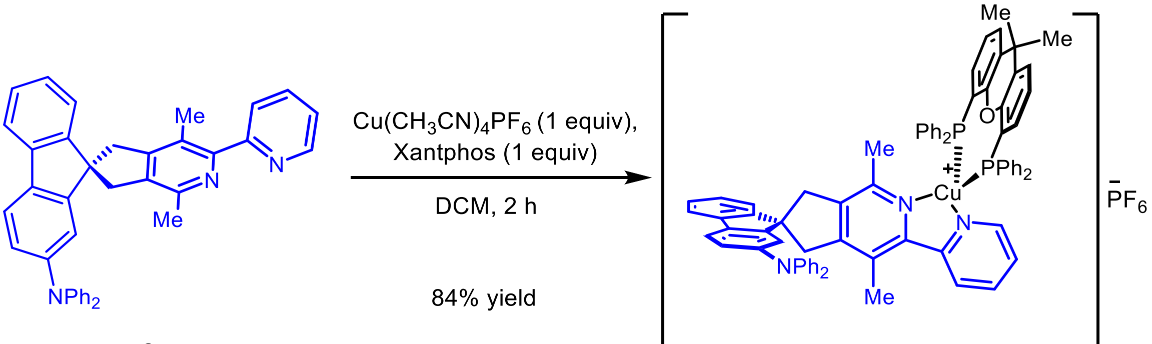


The ligands xantphos (135.4 mg, 0.25 mmol) and Phbpy ligand (144.7 mg, 0.25 mmol, **8q**) were dissolved in $CH_2Cl_2$ (20 mL) and the solution was added dropwise to a colourless solution of $[Cu(MeCN)_4][PF_6]$ (93.2 mg, 0.25 mmol) in $CH_2Cl_2$ (20 mL). The final solution changed to yellow during the addition and then to red; after stirring for 2 h at room temperature, a yellow colour persisted and the reaction mixture was filtered, and solvent removed from the filtrate in vacuo. The yellow-brown sticky residue was redissolved in $CH_2Cl_2$ and addition of $Et_2O$ yielded a pale-yellow precipitate. It was washed with hexane and [Cu(xantphos)(**8q**)][$PF_6$] was isolated as a yellow solid  with 280.3 mg, 0.211 mmol, 84% yield as a green solid[22].

**$^1$H NMR** (400 MHz, $CD_2Cl_2$) δ 8.02 – 7.78 (m, 3H), 7.74 – 7.54 (m, 4H), 7.49 – 6.80 (m, 38H), 6.68 (t, *J* = 7.1 Hz, 1H), 6.61 (t, *J* = 7.1 Hz, 1H), 3.50 – 3.32 (m, 2H), 3.23 (d, *J* = 17.0 Hz, 1H), 3.09 (d, *J* = 17.0 Hz, 1H), 2.51 (s, 3H), 2.05 (s, 3H), 1.78 (s, 3H), 1.70 (s, 3H).

**$^{13}$C NMR** (101 MHz, $CD_2Cl_2$) δ 155.9, 155.6 – 155.0 (m), 154.3, 153.0, 152.2, 151.6, 150.7, 149.4, 148.5, 148.2, 139.3, 139.2, 137.8, 134.8, 134.4 – 134.3 (m), 133.8 – 133.6 (m), 133.5 – 133.2 (m), 133.0 – 132.7 (m), 132.4 – 131.5 (m), 131.3, 131.0, 130.5, 130.2, 129.7, 129.4 – 128.8 (m), 128.4, 127.8, 127.6, 127.3, 126.6, 126.0 – 125.4 (m), 124.8, 124.7, 124.0, 123.4, 121.6, 121.2, 119.9, 118.3, 56.3, 46.0, 44.4, 36.5, 28.4, 28.1, 23.9, 18.3.

**$^{19}$F NMR** (376 MHz, $CD_2Cl_2$) δ -71.6 (d, *J* = 710.2 Hz).

**$^{31}$P NMR** (162 MHz, $CD_2Cl_2$) δ -7.1 – -15.0 (m), -130.9 – -153.8 (m).

**HRMS** (ESI/QTOF) m/z: $[M]^+$ Calcd for $C_{78}H_{63}CuN_3OP_2^+$ 1182.3737; Found 1182.3734.

$[\boldsymbol{\alpha}]_{D}^{20}$**:** -3.9 (c = 0.56, $CHCl_3$).

**IR (ATR)**: $\tilde{\nu}$ ($cm^{-1}$) = 3056, 2963, 2925, 1586, 1487, 1451, 1435, 1403, 1314, 1276, 1263, 1223, 1154, 1096, 1073, 1027, 875, 836, 795, 744, 694, 666, 639, 557, 534, 511, 485, 461, 426, 411.

**Melting point**: -
**$R_f$**: -

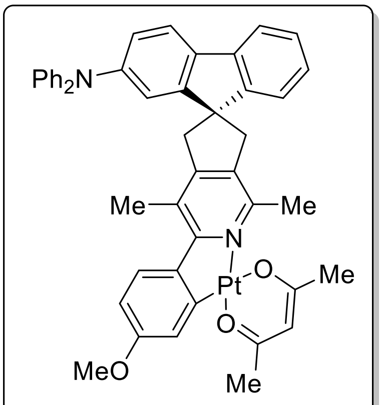


**11b**

**Synthesis**:

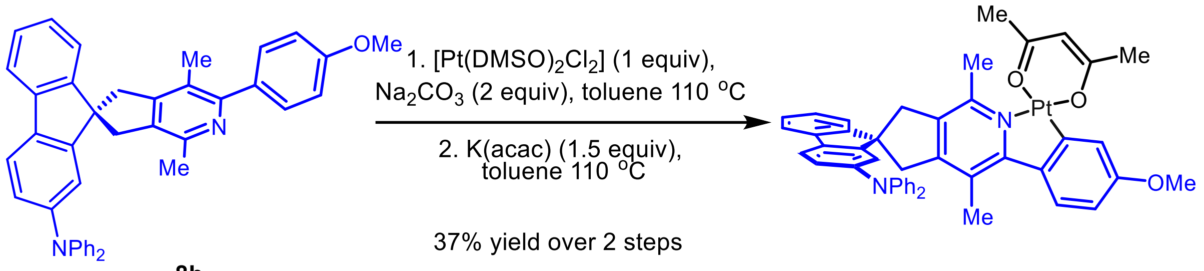


**8b** **11b**

A dried Schlenk tube was charged with the substrated **8b** (110 mg, 0.1927 mmol, 1eq), $[Pt(DMSO)_2Cl_2]$ (81.4 mg, 0.1924 mmol, 1 eq) and $Na_2CO_3$ (40.9 mg, 0.386 mmol, 2 eq). After cycles of vacuum-Ar, dried and deoxygented toluene (4 mL) was added and the resulting reaction mixture was stirred at 110 °C for 16 h. Then, reaction was cooled to rt, filtered through a pad of celite, washed with $CH_2Cl_2$ and concentrated to dryness in the rotavap. Then the residue was added 1.5 equiv K(acac) (40.0 mg, 0.289 mmol, 1.5 eq) then reflux in toluene under inert atmosphere overnight. Then, reaction was cooled to rt, filtered through a pad of celite, washed with $CH_2Cl_2$ and concentrated to dryness in the rotavap, then the desired compound **11b** was isolated by chromatography in 61.0 mg, 0.0705 mmol, 37% yield as a yellow solid[23].

**$^1$H NMR** (400 MHz, $CD_2Cl_2$) δ 7.65 (d, $J$ = 7.5 Hz, 1H), 7.61 (d, $J$ = 8.1 Hz, 1H), 7.55 (d, $J$ = 8.9 Hz, 1H), 7.37 – 7.30 (m, 1H), 7.28 – 7.03 (m, 13H), 6.99 (t, $J$ = 7.4 Hz, 2H), 6.64 (dd, $J$ = 8.8, 2.8 Hz, 1H), 5.49 (s, 1H), 3.86 (s, 3H), 3.44 – 3.27 (m, 4H), 2.79 (s, 3H), 2.49 (s, 3H), 1.97 (s, 3H), 1.89 (s, 3H).

**$^{13}$C NMR** (101 MHz, $CD_2Cl_2$) δ 186.1, 184.0, 166.1, 158.0, 155.9, 154.9, 153.2, 152.9, 148.4, 148.1, 140.9, 139.8, 139.5, 134.5, 134.0, 129.6, 128.2, 128.1, 127.4, 124.7, 123.5, 123.4, 123.3, 122.4, 120.9, 119.6, 118.2, 114.0, 109.6, 101.7, 56.2, 55.3, 45.8, 45.0, 28.1, 27.3, 22.5, 18.2.

**HRMS** (ESI/QTOF) m/z: $[M + H]^+$ Calcd for $C_{46}H_{41}N_2O_3Pt^+$ 864.2760; Found 864.2777.
$[\alpha]_D^{20}$**:** -30.8 (c = 0.33, $CHCl_3$).

**IR (ATR)**: $\tilde{\nu}$ (cm$^{-1}$) =: 2923, 2852, 1583, 1519, 1488, 1451, 1424, 1398, 1331, 1301, 1273, 1233, 1217, 1181, 1154, 1137, 1056, 1041, 1026, 974, 938, 866, 801, 778, 754, 738, 697, 666, 618, 583, 570, 559, 519, 475, 452, 435.

**Melting point**: decompose (200 °C) before reaching the melting point.

**$R_f$**: 0.64 (ethyl acetate: pentane = 1:4).

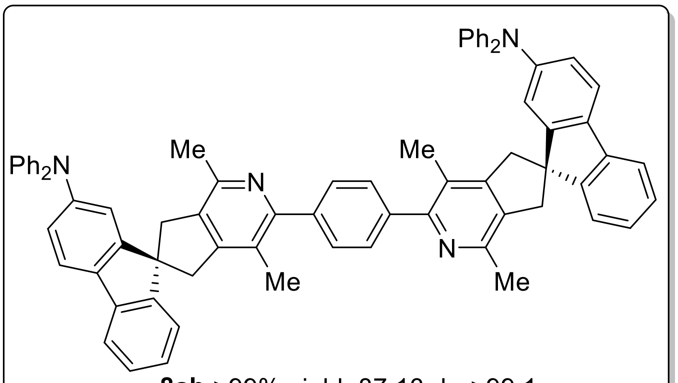


**8ab** >99% yield 87:13 dr, >99:1 er

**Synthesis**:

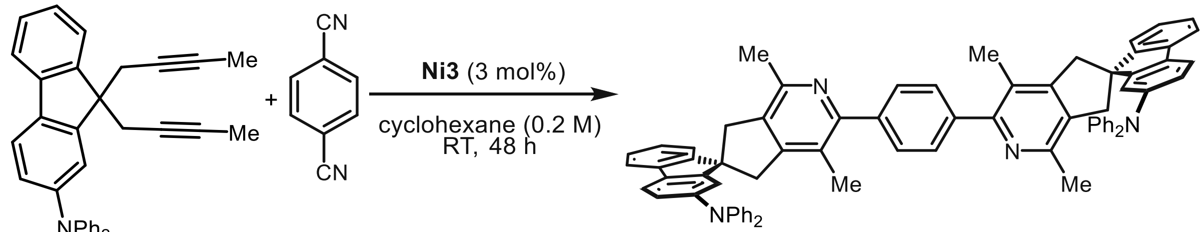


**1d** **8ab**, 95% yield, 87:13 dr, >99:1 er

In a glovebox, an oven dried screw-capped 5 mL vial was charged with a magnetic stir bar, Ni(0)NHC styrene complex **Ni3** (7.2 mg, 6 μmol, 3 mol%), cyclohexane (2 mL) was then added. Dialkyne **1d** (175.0 mg, 0.4 mmol, 2 eq) and nitrile (25.6 mg, 0.1 mmol, 1 eq) was then added successively. The vial was sealed with a Teflon-lined screw cap, and running inside the glovebox at room temperature. After 24 h, the vial was shipped outside of the glovebox. The reaction mixture was diluted with dichloromethane and filtered through a plug of silica gel. The crude solution was concentrated in vacuum and subjected to column chromatography to isolate the product **8ab** with 190.0 mg, 0.1894 mmol, 95% yield, 87:13 dr, >99:1 er (dr come from the meso compound) as a pale-yellow foam.

**$^1$H NMR** (400 MHz, $CDCl_3$) δ 7.65 (d, *J* = 7.6 Hz, 2H), 7.62 – 7.50 (m, 6H), 7.38 – 7.30 (m, 2H), 7.29 – 7.15 (m, 12H), 7.15 – 7.04 (m, 12H), 7.04 – 6.97 (m, 4H), 3.51 – 3.25 (m, 8H), 2.47 (s, 6H), 2.18 (s, 6H).

**$^{13}$C NMR** (101 MHz, $CDCl_3$) δ 156.9, 153.4, 152.9, 152.3, 150.7, 148.0, 147.9, 140.3, 139.3, 135.1, 134.4, 129.4, 129.2, 127.7, 127.0, 124.9, 124.4, 123.3, 123.1, 122.0, 120.6, 119.4, 118.0, 56.6, 45.1, 44.2, 22.2, 16.5.

**HRMS** (ESI/QTOF) m/z: $[M + H]^+$ Calcd for $C_{74}H_{59}N_4^+$ 1003.4734; Found 1003.4729.

$[\alpha]_D^{20}$: 103.1 (c = 0.27, $CHCl_3$).

**IR (ATR)**: $\tilde{\nu}$ ($cm^{-1}$) = 3035, 2961, 1583, 1486, 1468, 1450, 1426, 1345, 1330, 1313, 1261, 1220, 1196, 1174, 1154, 1073, 1019, 971, 894, 874, 849, 801, 779, 753, 734, 695, 660, 635, 587, 575, 513, 455, 409.

**Melting point**: -

**R$_f$**: 0.24 (ethyl acetate: pentane = 1:1).

**HPLC**: Chiralpak ID; hexane:iPrOH 99:1, 1 mL/min, 254 nm; *t*R (major) = 14.2 min, *t*R (minor) = 30.9 min, >99:1 er *t*R (diastereoisomer) = 21.4 min, 87:13 dr.

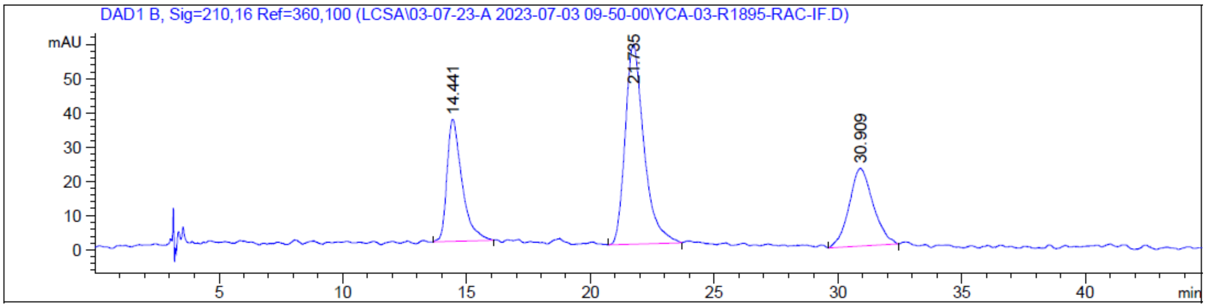


Signal 2: DAD1 B, Sig=210,16 Ref=360,100

| Peak # | RetTime [min] | Type | Width [min] | Area [mAU*s] | Height [mAU] | Area % |
|---|---|---|---|---|---|---|
| 1 | 14.441 | BB | 0.5940 | 1505.51074 | 35.70126 | 24.5459 |
| 2 | 21.735 | BB | 0.7314 | 3094.96826 | 58.47966 | 50.4604 |
| 3 | 30.909 | BB | 0.8146 | 1532.98181 | 22.79525 | 24.9937 |

Totals : 6133.46082 116.97616

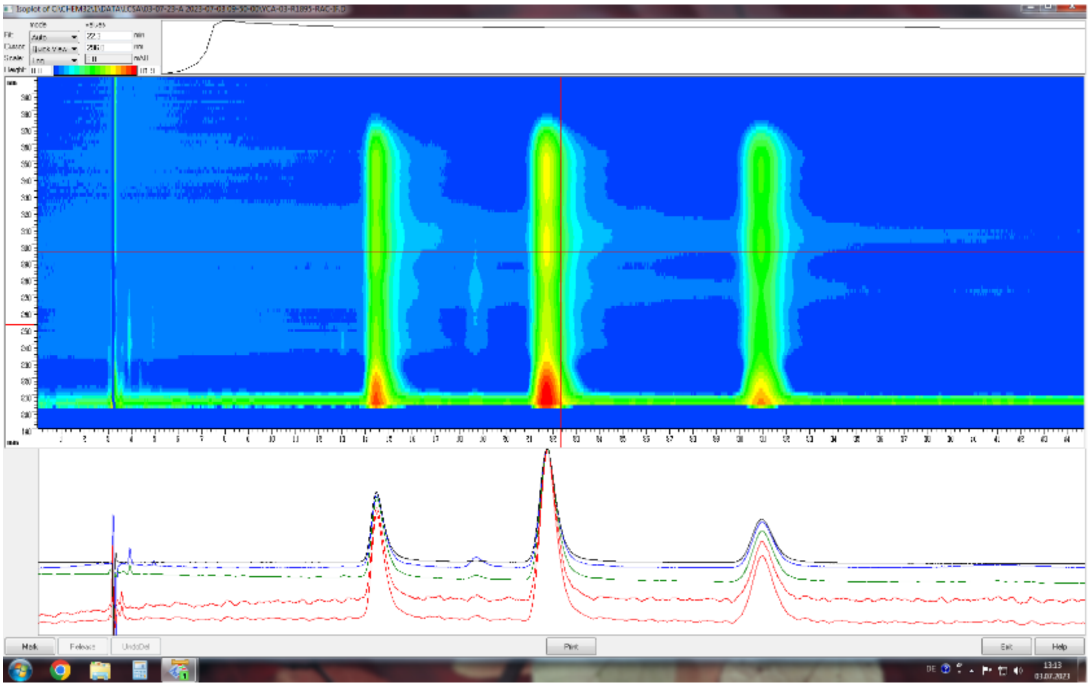

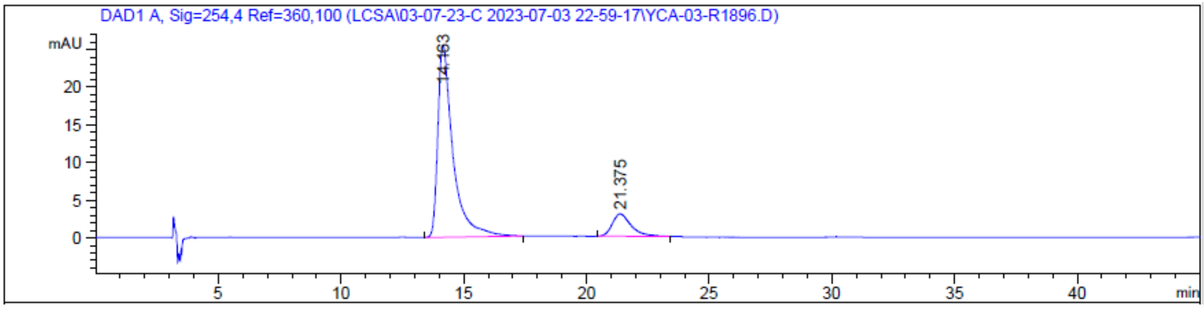


Signal 1: DAD1 A, Sig=254,4 Ref=360,100

| Peak # | RetTime [min] | Type | Width [min] | Area [mAU*s] | Height [mAU] | Area % |
|---|---|---|---|---|---|---|
| 1 | 14.163 | BB | 0.6193 | 1091.06494 | 25.49427 | 87.2499 |
| 2 | 21.375 | BB | 0.6469 | 159.44061 | 2.96729 | 12.7501 |

Totals : 1250.50555 28.46156

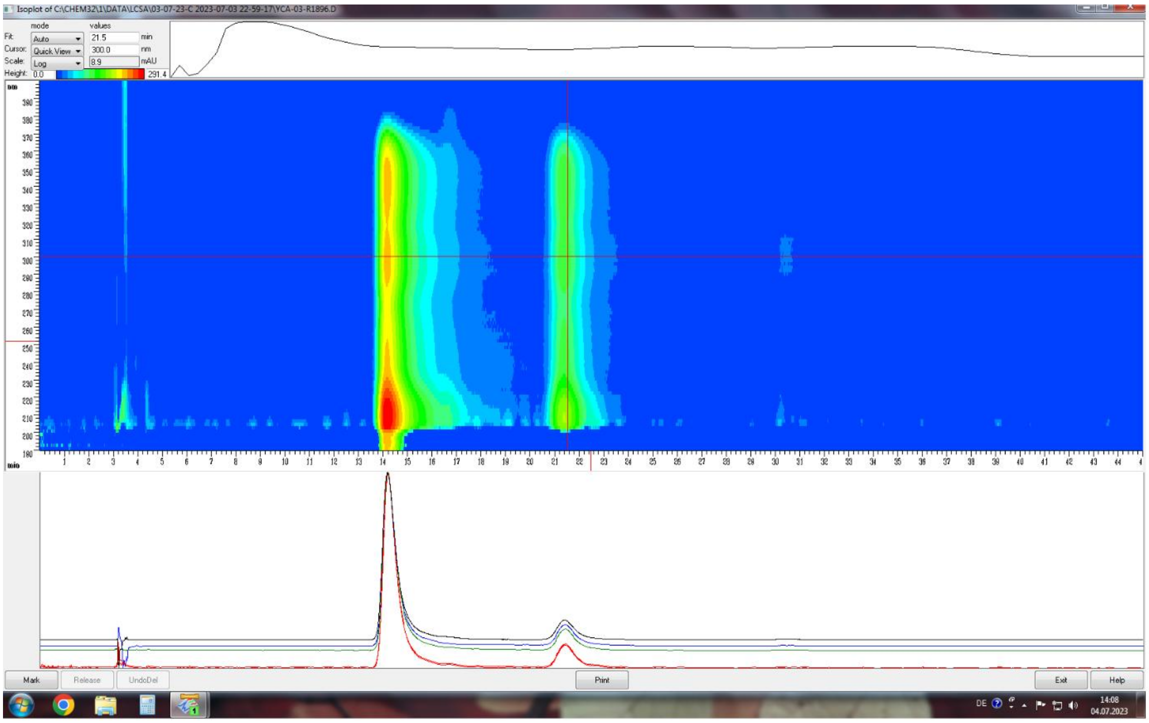

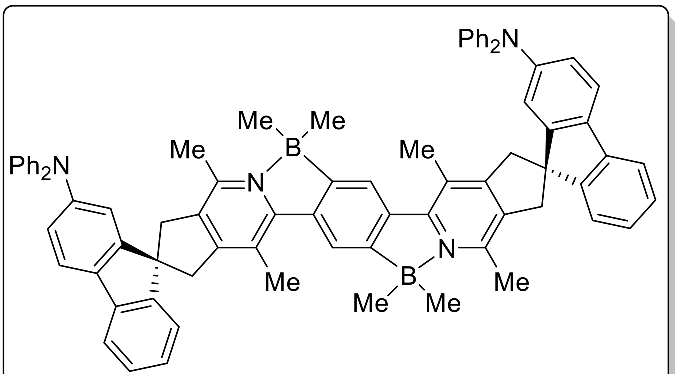

**11c**

**Synthesis**:

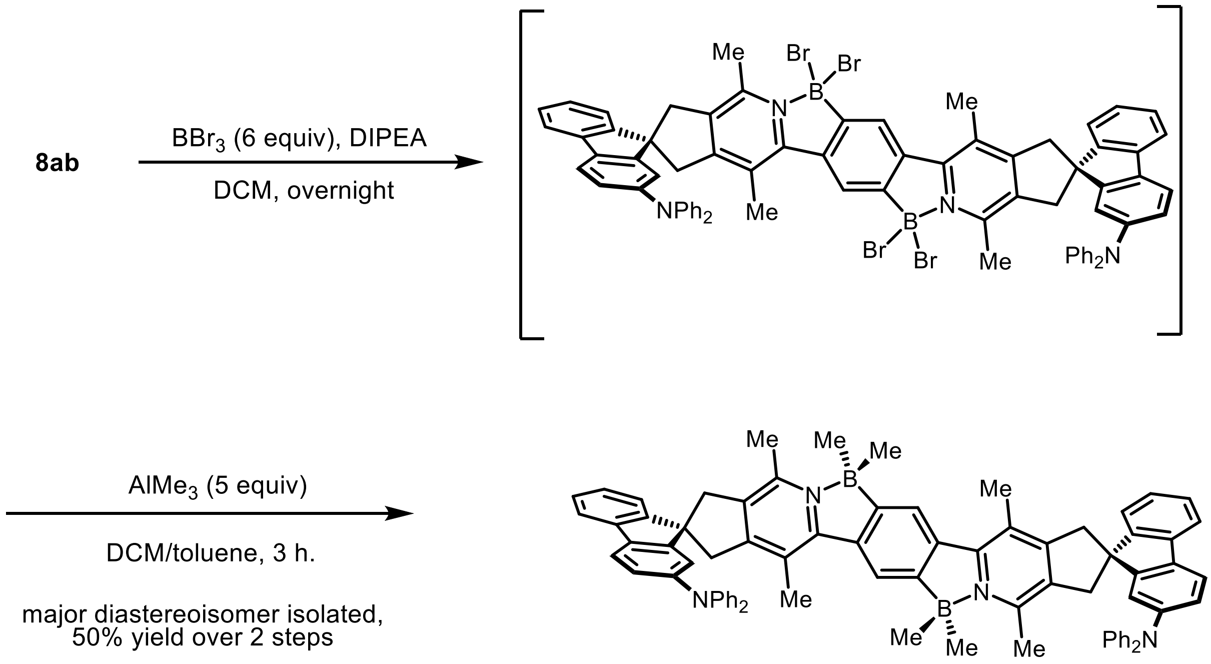


**11c**

Add $BBr_3$ (1.0 M in $CH_2Cl_2$, 0.285 mL, 6 equiv) to a stirred solution of 1,4-di(2-pyridyl)benzene **8ab** (190 mg, 0.1894 mmol) and *i*-$Pr_2NEt$ (73.4 mg, 0.568 mmol, 3 equiv) in $CH_2Cl_2$ (10 mL) at 0 °C. Stir the reaction mixture at room temperature for 24 h. Add saturated $K_2CO_3$ aqueous solution to the reaction mixture. Extract the filtrate with $CHCl_3$ and dry with $Na_2SO_4$. Then concentrated to dryness in the rotavap, and desired intermediate was allowed to directly submit to next step without purification.

To a stirred solution of the boron dibromide species in toluene (5.0 mL) and $CH_2Cl_2$ (5.0 mL) at room temperature was added $Me_3Al$ (2.0 M in hexane, 0.38 mL, 5 equiv). After being stirred at this temperature for 3 h, the reaction was quenched by adding water. The organic layer was separated and extracted with AcOEt (10 mL ×2), washed with water (10 mL ×1), brine (10 mL ×1), and dried over $MgSO_4$ and concentrated. The residue was purified by silica gel chromatography to afford **11c** in 82.0 mg, 0.0757 mmol, 50 % yield over 2 steps as a white solid (major diastereoisomer isolated *via* chromatography).

**$^{1}$H NMR** (400 MHz, $CD_2Cl_2$) δ 8.17 (s, 2H), 7.68 (d, *J* = 7.5 Hz, 2H), 7.62 (d, *J* = 8.1 Hz, 2H), 7.36 (t, *J* = 8.1 Hz, 2H), 7.27 – 7.15 (m, 12H), 7.14 – 7.05 (m, 10H), 7.02 (d, *J* = 2.3 Hz, 2H), 6.96 (t, *J* = 7.4 Hz, 4H), 3.61 – 3.36 (m, 8H), 2.87 – 2.55 (m, 12H), 0.37 – 0.11 (m, 12H).

**$^{11}$B NMR** (128 MHz, $CD_2Cl_2$) δ 0.6.

**$^{13}$C NMR** (101 MHz, $CD_2Cl_2$) δ 166.1, 156.1, 155.6, 153.2, 152.3, 148.9, 148.5, 148.0, 139.7, 138.6, 136.2, 134.3, 129.7, 128.2, 127.4, 126.0, 124.8, 124.7, 123.5, 123.4, 122.4, 120.9, 119.7, 117.9, 56.6, 45.7, 44.2, 18.5, 18.0, 9.4 – 8.6 (m).

**HRMS** (ESI/QTOF) m/z: $[M + H]^+$ Calcd for $C_{78}H_{69}B_2N_4^+$ 1083.5703; Found 1083.5667.

$[\alpha]_D^{20}$**:** 131.2 (c = 0.16, $CHCl_3$).

**IR (ATR)**: $\tilde{\nu}$ ($cm^{-1}$) =: 3056, 2922, 2853, 1597, 1490, 1452, 1429, 1378, 1347, 1314, 1280, 1243, 1222, 1156, 1106, 1071, 1048, 1019, 990, 941, 876, 825, 776, 754, 736, 697, 666, 648, 620, 609, 587, 572, 541, 519, 494, 476, 464, 440, 431, 423, 414.

**Melting point**: -

**$R_f$**: 0.80 (ethyl acetate: pentane = 1:4).

**Photophysical properties of 11a, 11b, and 11c.**

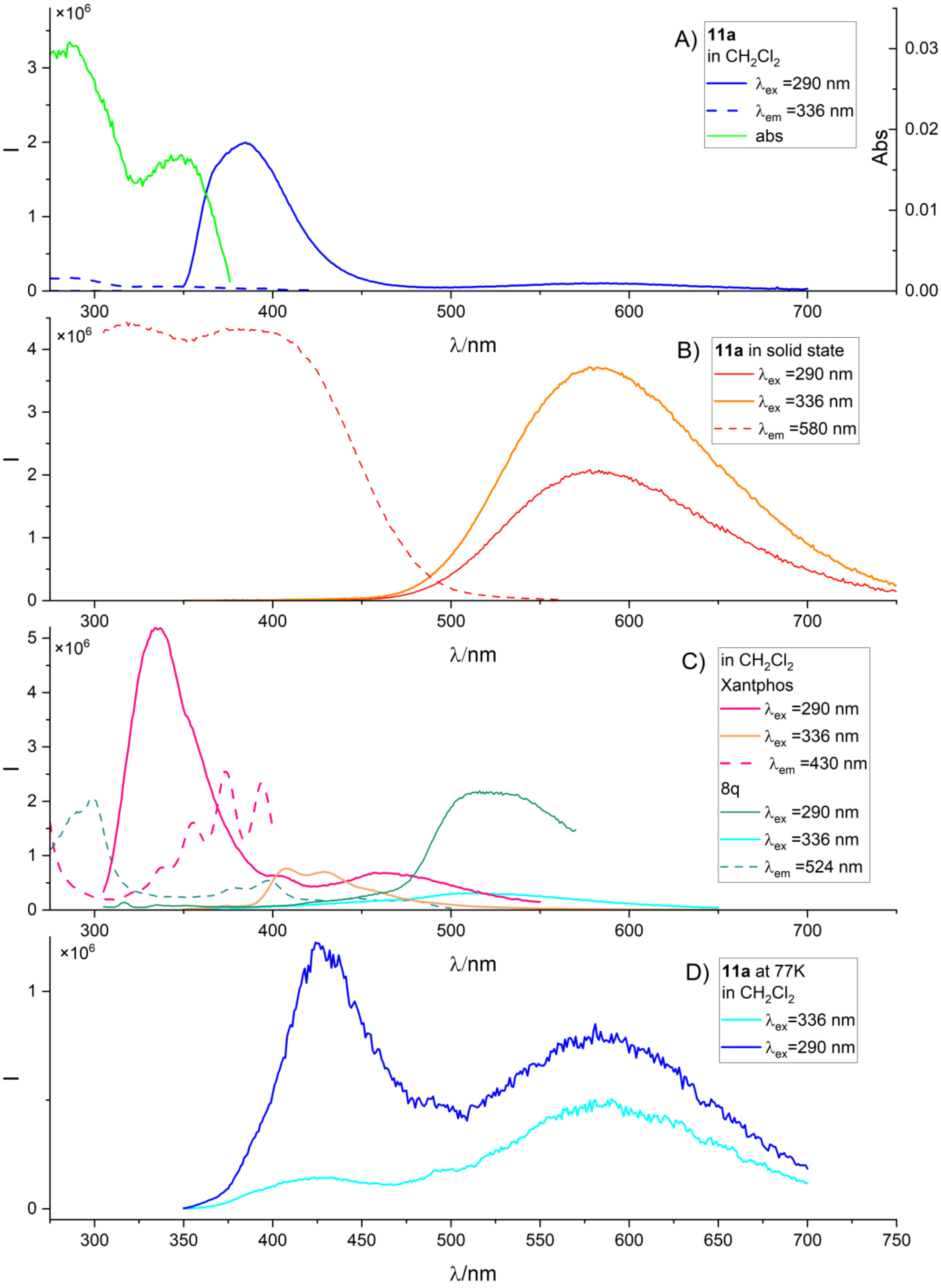


**Supplementary Fig. 8**. Corrected excitation and emission spectra of **11a A)**. 1μM in DCM at 298 K. **B)**. in solid state. **C)**. Xantphos and **8q** in DCM solution (1μM). **D)**. **11a** 1μM in DCM at 77K.

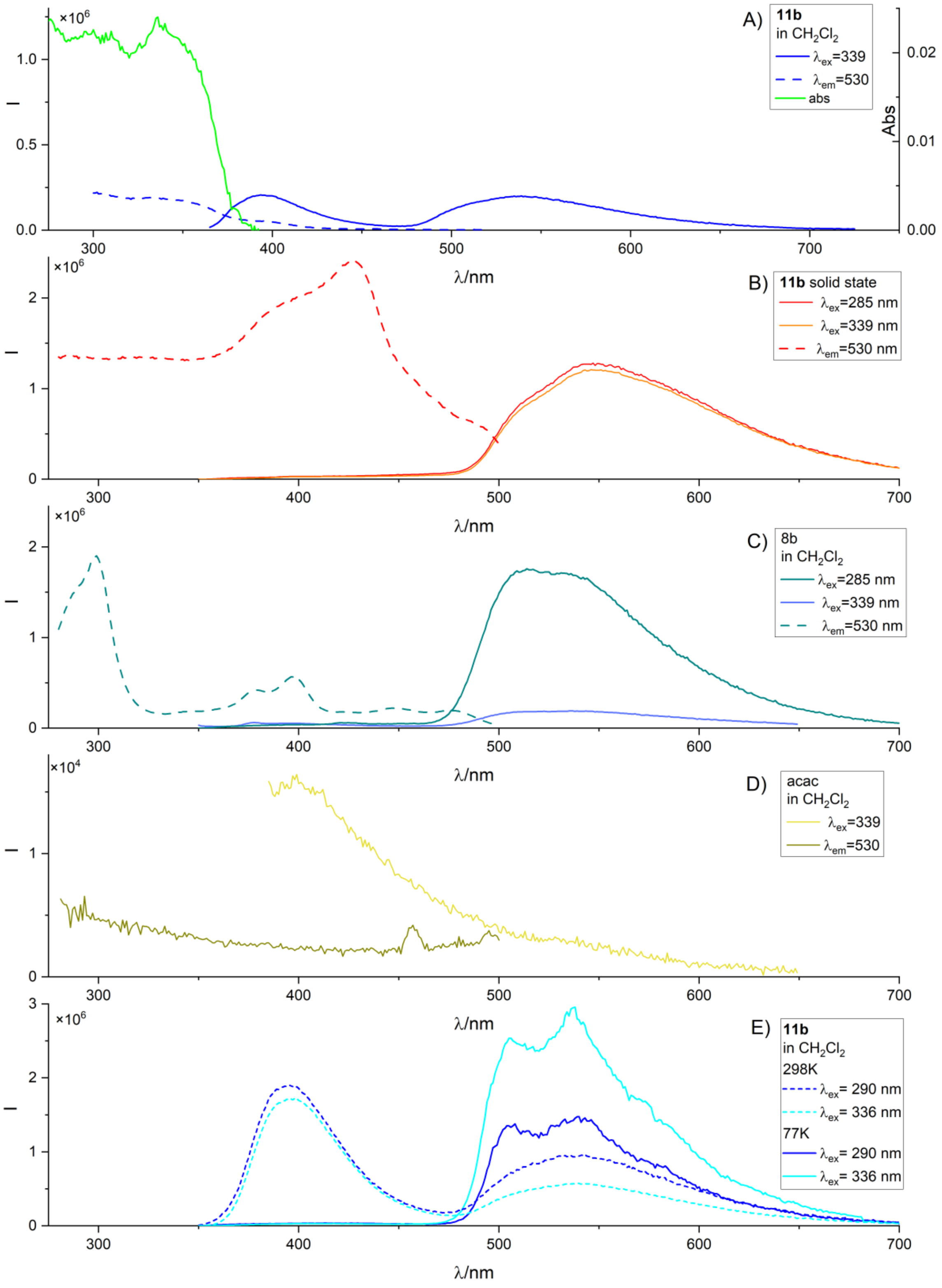


**Supplementary Fig. 9.** Corrected excitation and emission spectra of **A) 11b** 1μM in DCM at 298 K and 77K. **B)**. in solid state **C) 8b** (1μM in DCM). **D)**. acac ligand in DCM solution (1μM). **E)**. **11b** 1μM in DCM at 77 K.

**Supplementary Table 9.** Lifetimes and absolute quantum yield of **11a-c.**

| Entr | Compounc | conditior | $\lambda_{em}$/nı | $\lambda_{em}$/nı | $\tau_1$/μs | $\tau_2$/μs | Φ/% |
|---|---|---|---|---|---|---|---|
| 1 | **11a** | a | 290 | 580 | 0.34±0.0 | 5.91±0.7[illegible] | 1.32±0.2 |
| 2 | | a | 336 | 580 | 1.15±0.0 | 10.45±0.4 | 1.28±0.1 |
| 3 | | b | 290 | 580 | 0.69±0.0 | 8.18±0.1 | 0.45±0.0 |
| 4 | | b | 336 | 580 | 0.65±0.0 | 8.06±0.0[illegible] | 0.45±0.1 |
| 5 | | c | 290 | 580 | 0.47±0.0 | 7.59±1.0[illegible] | 2.78±0.2 |
| 6 | | c | 336 | 580 | 2.61±0.0 | 8.33±0.0[illegible] | 1.93±0.[illegible] |
| 7 | **11b** | a | 297 | 530 | 0.48±0.0 | 3.30±0.1[illegible] | -[d] |
| 8 | | a | 339 | 530 | 0.95±0.0 | 7.16±0.3[illegible] | -[d] |
| 9 | | b | 297 | 530 | 0.39±0.0 | 4.10±0.4[illegible] | -[d] |
| 10 | | b | 339 | 530 | 0.74±0.0 | 8.38±0.6[illegible] | -[d] |
| 11 | | c | 290 | 584 | 1.27±0.0 | 9.47±0.0[illegible] | 0.38±0.0 |
| 12 | **11c** | a | 336 | 489 | 0.99±0.0 | 8.74±0.0[illegible] | 1.02±0.0 |
| 13 | | b | 336 | 489 | 1.10±0.0 | 8.88±0.0[illegible] | 0.95±0.0 |
| 14 | | c | 384 | 426 | 0.97±0.0 | 8.61±0.0[illegible] | 2.59±0.4 |
| 15 | | c | 384 | 462 | 0.79±0.0 | 8.27±0.0[illegible] | 2.58±0.3 |

[a]1 μM in deoxygenated DCM [b] in presence of $O_2$. [c]in solid state [d]The quantum yield was not measured due to limited emission.

**Chiral optical properties.**

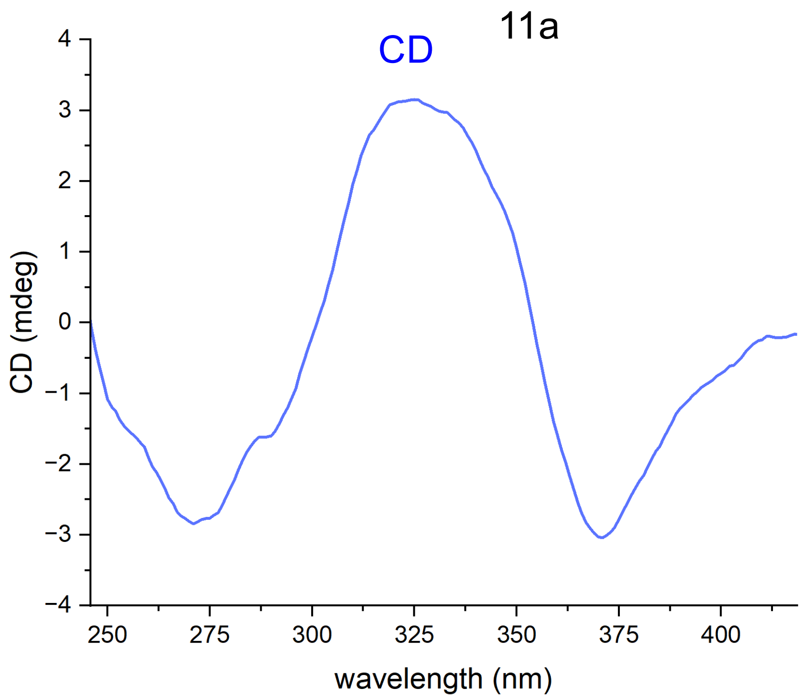


**Supplementary Fig. 10.** CD spectra of compound **11a**.

No CPL activity observed for **11a**.

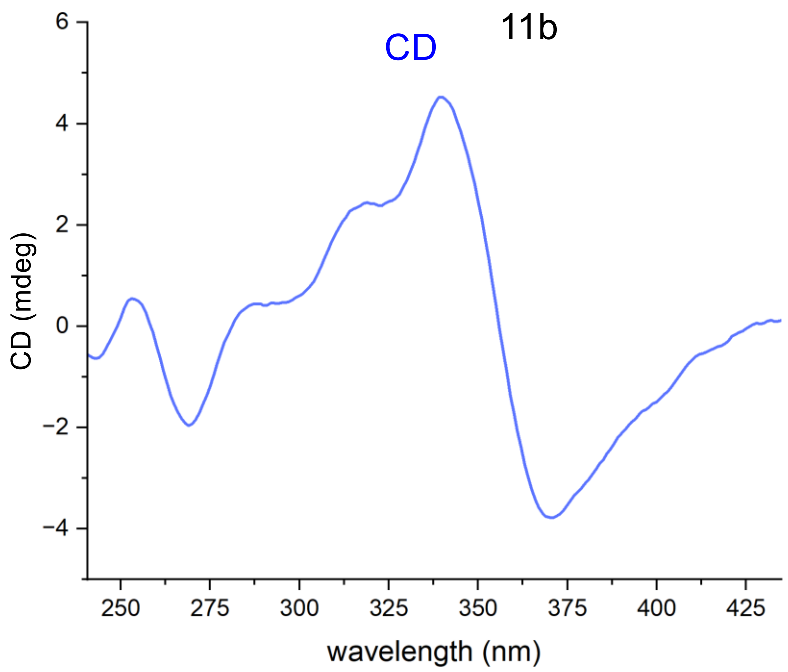


**Supplementary Fig. 11.** CD spectra of compound **11b.**

No CPL activity observed for **11b**.

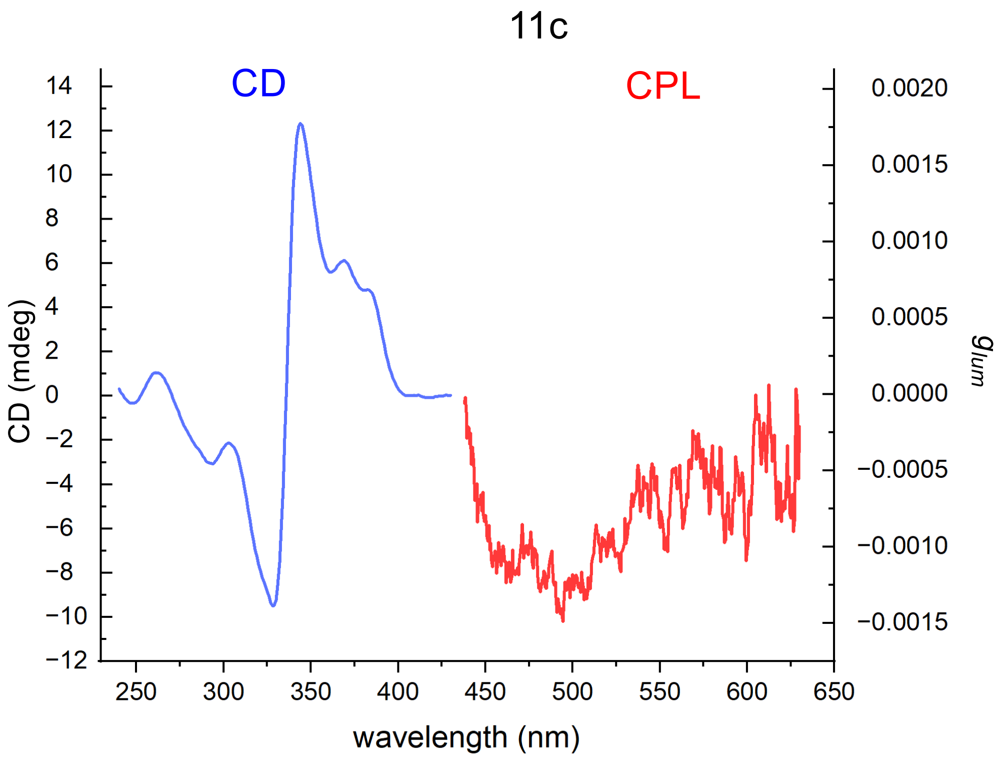


**Supplementary Fig. 12.** CD spectra and CPL spectra of compound **11c.**

**Mechanistic studies.**

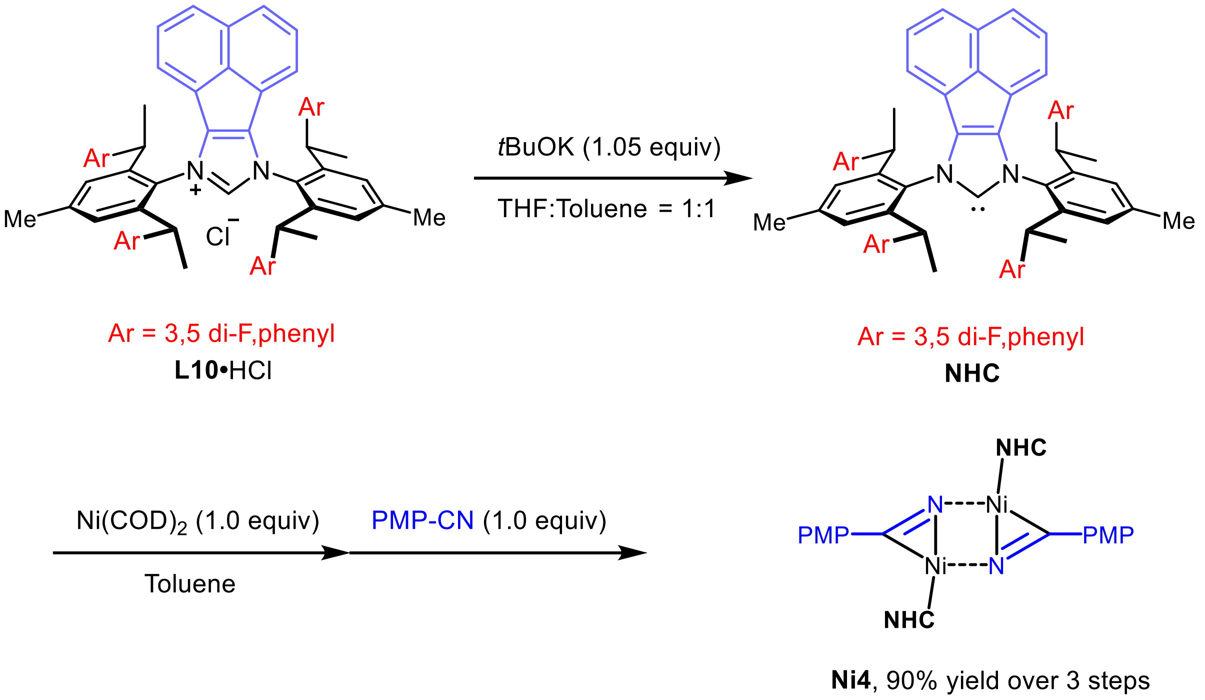


**Supplementary Fig. 13.** Synthesis of **Ni4** species.

**Experimental.** Inside the glovebox, the NHC salt **L10**•HCl (50 mg, 0.0515 mmol, 1 eq) was suspended in THF/toluene (1:1 mL) and KO*t*Bu (6.1 mg, 1.05 equiv) was added. The mixture was stirred inside the glovebox for 4 hours. After removal of the solid by-products by filtration through a pad of celite on a frit. The solution was concentrated directly afforded the desired free carbenes, the free carbene was directly submitted to the next step without further purification[24,25].

Then dissolve $Ni(COD)_2$ (14.2 mg, 0.0515 mmol, 1 eq) and free carbene in hexane:cyclohexane 1:1 (4 mL). Stir the reaction for 60 min, add 4-methoxybenzonitrile (7.5 mg, 0.051500 mmol) in one portion. Use an additional 0.5 ml hexane:cyclohexane 1:1 to ensure complete transfer of nitrile to reaction mixture, reaction become turbid after 15 min. Stir for 1 hr, cool the reaction mixture to -30 °C and allow to sit overnight to induce maximum precipitation. Decant the remaining solvent and dry the precipitate under vacuo to obtain product **Ni4** in 52.1 mg, 0.023 mmol, 90% yield over 3 steps as a brown solid.

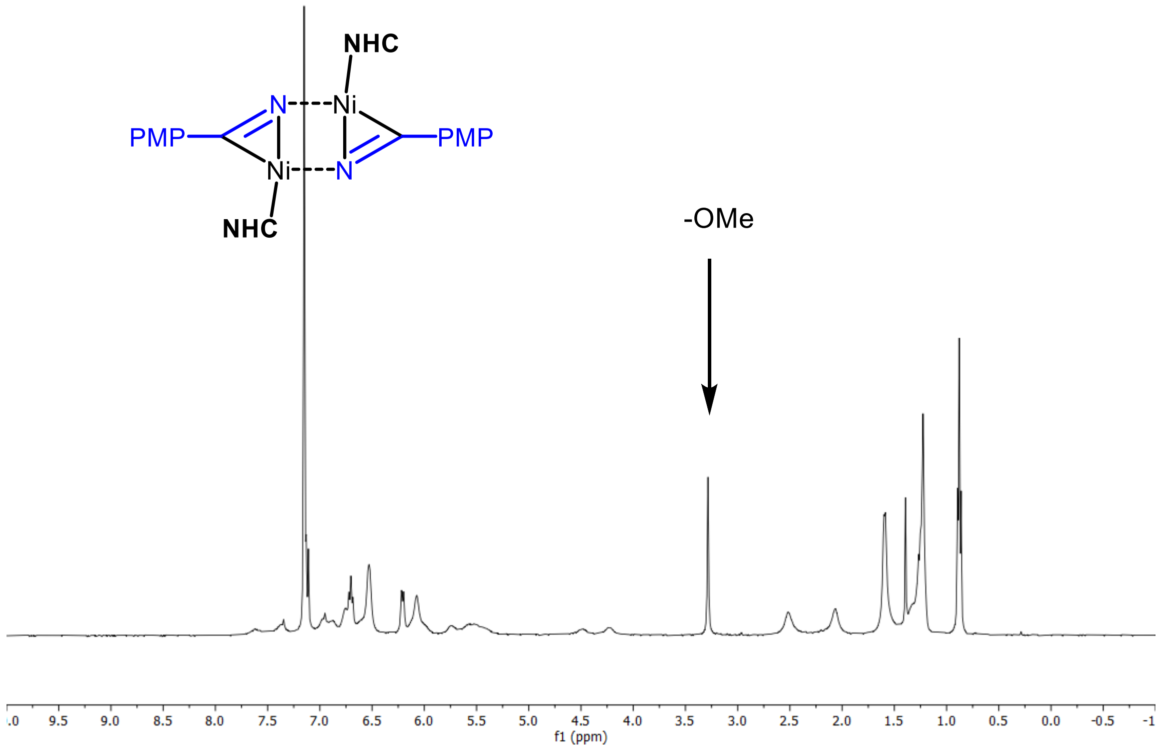


Crude $^{1}$H NMR of the obtain solid, only one methoxy-signal was observed.

The crystal was obtained by slow diffusion of *n*-hexane to a saturated solution of **Ni4** in $C_6H_6$.

Front view of crystal structure **Ni4**.

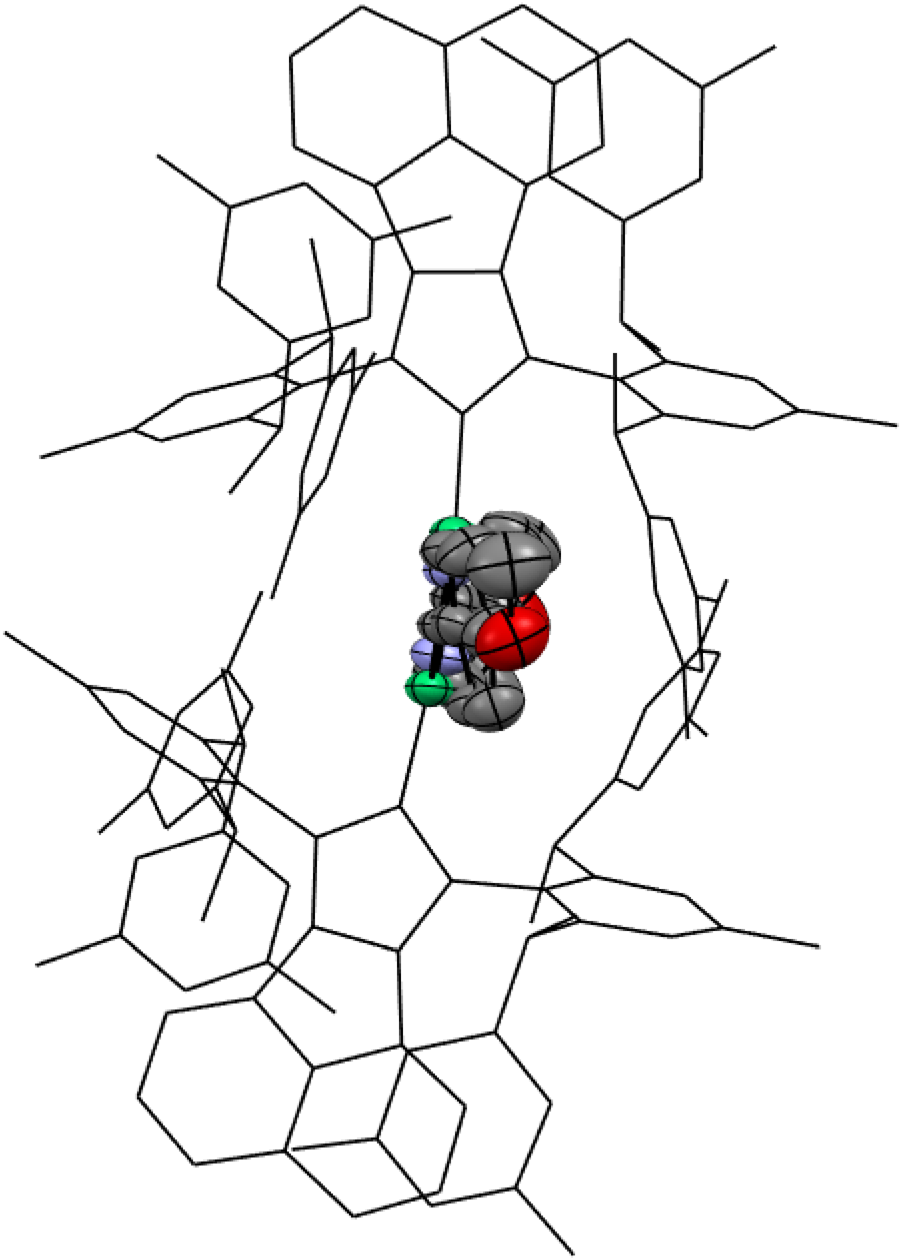

ORTEP diagram of **Ni4** (thermal ellipsoids are shown at the 50% probability level), for the reason of clarity all the hydrogen atoms are omitted, and wire frame style was applied on NHC ligand.

Side view of crystal structure **Ni4**.

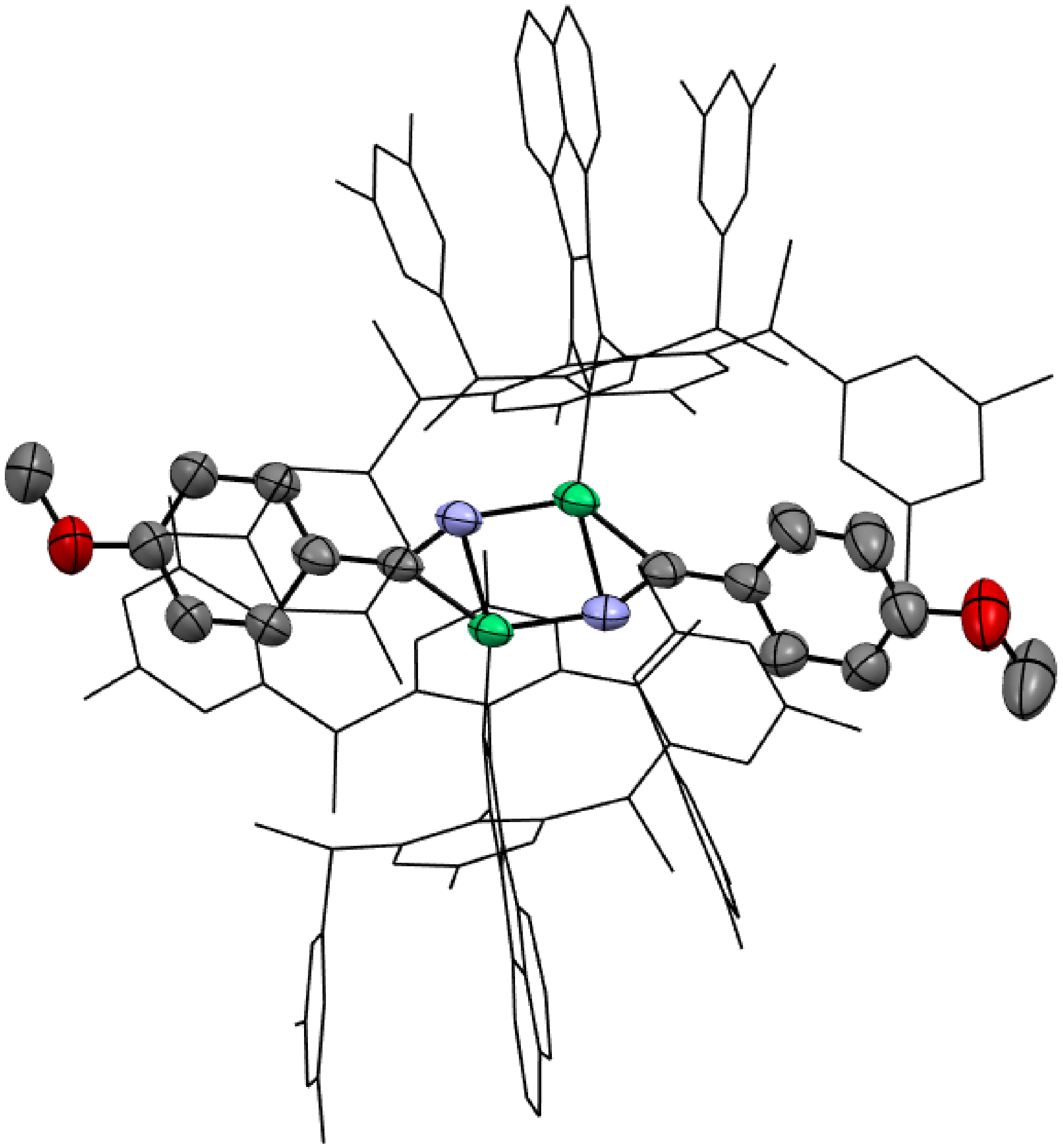

ORTEP diagram of **Ni4** (thermal ellipsoids are shown at the 50% probability level), for the reason of clarity all the hydrogen atoms are omitted, and wire frame style was applied on NHC ligand.

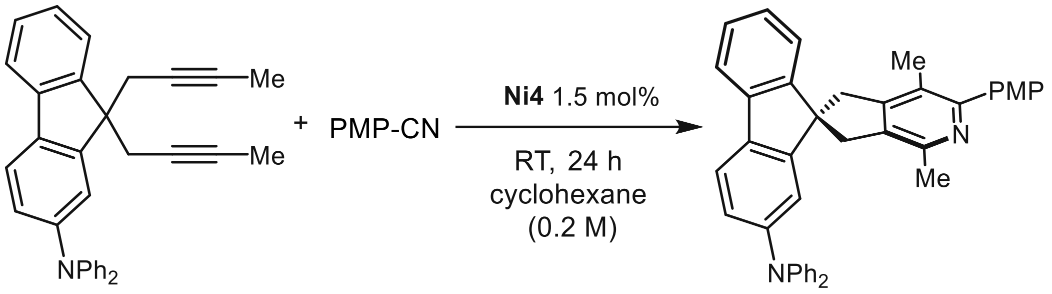


**1d**

**8b**, 95% yield, 96.5:3.5 er

**Supplementary Fig. 14.** Catalysis reactivity of **Ni4**.

**Experimental.** In a glovebox, an oven dried screw-capped 2 mL vial was charged with a magnetic stir bar, Ni(II) complex **Ni4** (1.7 mg, 1.5 μmol, 1.5 mol%), cyclohexane (0.25 mL) was then added. Dialkyne **1d** (21.9 mg, 0.05 mmol) and PMP-CN (6.7 mg, 0.05 mmol) was then added successively. The vial was sealed with a Teflon-lined screw cap and running inside the glovebox at room temperature. After 24 h, the vial was shipped outside of the glovebox. The reaction mixture was diluted with dichloromethane and filtered through a plug of silica gel. The crude solution was concentrated in vacuum and subjected to column chromatography to isolate the product **8b** in 27.1 mg, 0.0474 mmol, 95% yield and 96.5: 3.5 er.

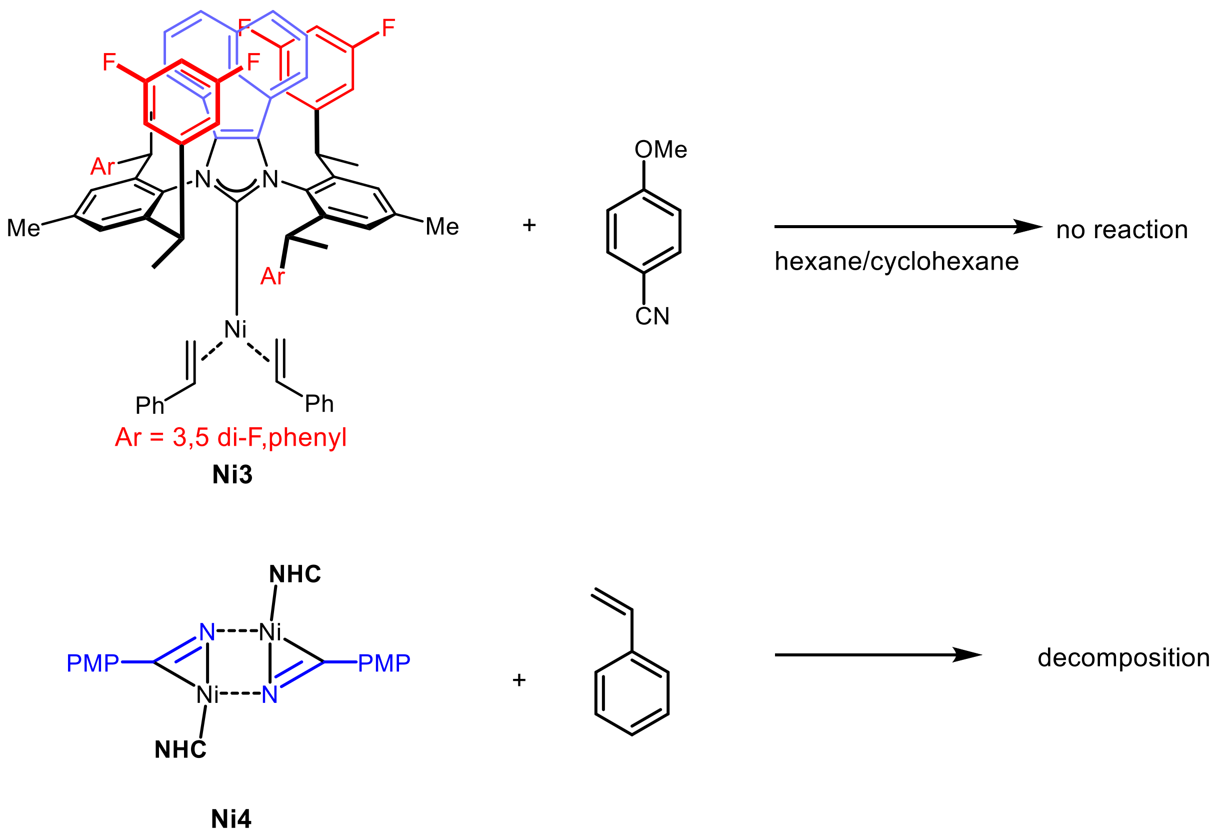


**Supplementary Fig. 15.** Control experiment 1.

**Experimental.** In a glovebox, an oven dried screw-capped 2 mL vial was charged with a magnetic stir bar, **Ni3** (24 mg, 0.020 mmol, 1 eq), 4-methoxybenzonitrile (2.7 mg, 0.02 mmol) was added in hexane:cyclohexane 1:1 (2 mL). No reaction occurred, **Ni3** was fully recovered.

**Experimental.** In a glovebox, an oven dried screw-capped 2 mL vial was charged with a magnetic stir bar, **Ni4** (11.2 mg, 0.010 mmol, 1 eq), styrene (10.4 mg, 0.10 mmol) was added in hexane:cyclohexane 1:1 (1 mL). Complex decomposed, formation of nickel black was observed.

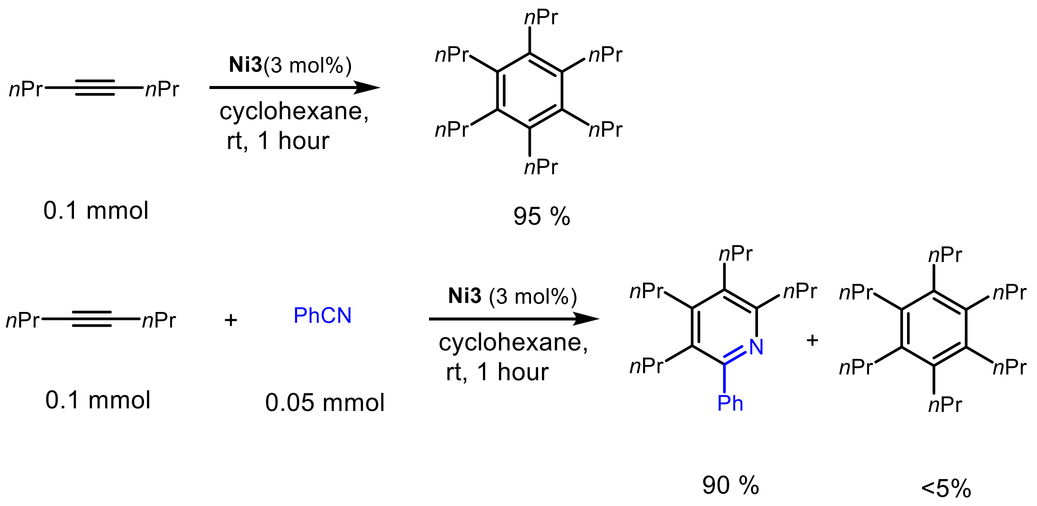


**Supplementary Fig. 16.** Control experiment 2.

**Experimental.** In a glovebox, an oven dried screw-capped 2 mL vial was charged with a magnetic stir bar, Ni(0)NHC styrene complex **Ni3** (3.6 mg, 3 μmol, 3 mol%), cyclohexane (0.5 mL) was then added. 4-octyne (11.0 mg, 0.1 mmol, 1 eq) was then added successively. The vial was sealed with a Teflon-lined screw cap and running inside the glovebox at room temperature. After 1 h, the vial was shipped outside of the glovebox. The reaction mixture was diluted with dichloromethane and filtered through a plug of silica gel. The crude solution was concentrated in vacuum and subjected to column chromatography to isolate the trimerization product in 10.5 mg, 0.032 mmol, 95% yield.

In a glovebox, an oven dried screw-capped 2 mL vial was charged with a magnetic stir bar, Ni(0)NHC styrene complex **Ni3** (3.6 mg 3 μmol), cyclohexane (0.5 mL) was then added. 4-octyne (11.0 mg, 0.1 mmol, 1 eq) and benzonitrile (5.2 mg, 0.05 mmol, 0.5 eq) was then added successively. The vial was sealed with a Teflon-lined screw cap and running inside the glovebox at room temperature. After 1 h, the vial was shipped outside of the glovebox. The reaction mixture was diluted with dichloromethane and filtered through a plug of silica gel. The crude solution was concentrated in vacuum and subjected to column chromatography to isolate the product in 14.6 mg, 0.045 mmol, 90% yield, trace amount of alkyne trimerization product was detected.

**Crystal Data and Experimental**

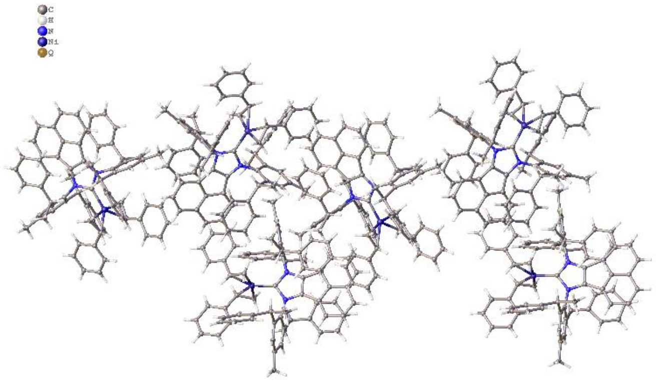

**Experimental.** Single clear intense orange irregular-shaped crystals of **Ni2** (yca-R2122) were used as supplied. A suitable crystal with dimensions 0.64 × 0.22 × 0.18 $mm^3$ was selected and mounted on a XtaLAB Synergy R, DW system, HyPix-Arc 150 diffractometer. The crystal was kept at a steady $T$ = 139.99(10) K during data collection. The structure was solved with the ShelXT (Sheldrick, 2015) solution program using dual methods and by using Olex2 1.5 (Dolomanov et al., 2009) as the graphical interface. The model was refined with ShelXL 2019/3 (Sheldrick, 2015) using full matrix least squares minimisation on $\boldsymbol{F^2}$.

**Crystal data**: $C_{80.75}H_{81.42}N_2Ni$, $M_r$ = 1138.60, monoclinic, $P2_1$ (No. 4), a = 11.30822(8) Å, b = 21.95655(16) Å, c = 76.8633(9) Å, $\beta$ = 90.0228(8)°, $\alpha = \gamma$ = 90°, $V$ = 19084.3(3) Å$^3$, $T$ = 139.99(10) K, $Z$ = 12, $Z'$ = 6, $\mu$(Cu $K_\alpha$) = 0.781, 455910 reflections measured, 59300 unique ($R_{int}$ = 0.0684) which were used in all calculations. The final $wR_2$ was 0.3843 (all data) and $R_1$ was 0.1516 (I≥2 $\sigma$(I)).

**CCDC: 2370294**

- The overall quality of the crystal was bad, but their crystallization was extremely difficult, and several were tried, before the launching of the data collection.
- The diffraction pattern displayed twinning and modulation issues at the same time. The structure could be solved as orthorhombic, but the obtained model was very disordered and refined hardly.
- The Monoclinic setting was chosen because the amount of disorder was considerably lower, and the model refined more easily (although not thoroughly).
- The solvent has been removed from the model in the last stages of the refinement (there are about 6 hexane molecules in the asymmetric unit) and some details about it are included within the CIF file**.**

Solvent masking (PLATON/SQUEEZE) information for **Ni2**

| **No** | **x** | **y** | **z** | **V** | **e** | **Content** |
|---|---|---|---|---|---|---|
| 1 | -0.042 | -0.192 | 0.120 | 723.2 | 137.7 | 2.75hexane |
| 2 | -0.640 | 0.307 | 0.384 | 768.2 | 150.7 | 3hexane |
| 3 | 0.709 | -0.193 | 0.616 | 768.2 | 150.7 | 3hexane |
| 4 | -0.134 | 0.031 | 0.781 | 27.9 | 0.0 | ? |

| No | x | y | z | V | e | Content |
|---|---|---|---|---|---|---|
| 5 | -0.576 | 0.308 | 0.880 | 723.2 | 137.5 | 2.75hexane |
| 6 | 0.134 | 0.531 | 0.219 | 27.9 | 0.0 | - |
| 7 | 0.181 | 0.109 | 0.972 | 23.9 | 0.0 | - |
| 8 | 0.204 | 0.619 | 0.734 | 35.3 | 0.0 | - |
| 9 | 0.452 | 0.185 | 0.637 | 36.3 | 0.0 | - |
| 10 | 0.548 | 0.685 | 0.363 | 36.3 | 0.0 | - |
| 11 | 0.796 | 0.119 | 0.266 | 35.3 | 0.0 | - |
| 12 | 0.819 | 0.609 | 0.028 | 23.9 | 0.0 | - |

**Explanations of B level alerts:**

PLAT084_ALERT_3_B High wR2 Value (i.e. > 0.25) ................... 0.38 Report.

- Author Response: The crystal diffracted quite poorly, and the model displays disordered moieties that cannot be handled correctly.

PLAT097_ALERT_2_B Large Reported Max. (Positive) Residual Density 3.01 eA-3.

- Author Response: This residual is rather close to one Ni atom and is maybe due to absorption artefacts

PLAT341_ALERT_3_B Low Bond Precision on C-C Bonds ............... 0.01852 Ang.

- Author Response: This alert is, very likely, due to the extensive disorder shown by some phenyl rings

PLAT411_ALERT_2_B Short Inter H...H Contact H11 ..H34D . 1.93 Ang. 1+x,y,z = 1_655 Check.

- Author Response: This alert is, very likely, due to the extensive disorder shown by some phely rings and, because of this, their orientation cannot be established with certainty.

PLAT411_ALERT_2_B Short Inter H...H Contact H17A ..H19 . 1.92 Ang. 1+x,y,z = 1_655 Check.

- Author Response: This alert is, very likely, due to the extensive disorder shown by some phely rings and, because of this, their orientation cannot be established with certainty.

PLAT411_ALERT_2_B Short Inter H...H Contact H93 ..H368 . 1.89 Ang. -1+x,-1+y,z = 1_445 Check

- Author Response: This alert is, very likely, due to the extensive disorder shown by some phely rings and, because of this, their orientation cannot be established with certainty.

PLAT411_ALERT_2_B Short Inter H...H Contact H254 ..H440 . 1.80 Ang. x,-1+y,z = 1_545 Check

- Author Response: This alert is, very likely, due to the extensive disorder shown by some phely rings and, because of this, their orientation cannot be established with certainty.

PLAT411_ALERT_2_B Short Inter H...H Contact H370 ..H371 . 1.80 Ang. 1+x,1+y,z = 1_665 Check

- Author Response: This alert is, very likely, due to the extensive disorder shown by some phely rings and, because of this, their orientation cannot be established with certainty.

PLAT987_ALERT_1_B The Flack x is >> 0 - Do a BASF/TWIN Refinement Please Check.

- Author Response: The TWIN/BASF combination has been used; this alert is likely to be a computer artifact.

## Crystal Data and Experimental

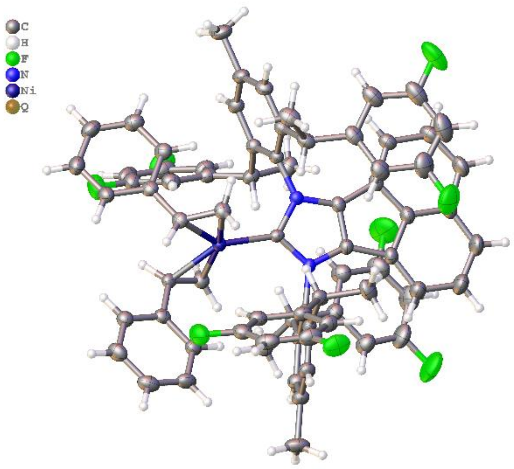


**Experimental.** Single clear intense red plate-shaped crystals of **Ni3** (yca-R2094) were used as supplied. A suitable crystal with dimensions 0.43 × 0.32 × 0.19 $mm^3$ was selected and mounted on a SuperNova, Dual, Cu at home/near, AtlasS2 diffractometer. The crystal was kept at a steady $T$ = 140.00(10) K during data collection. The structure was solved with the ShelXT 2018/2 (Sheldrick, 2018) solution program using dual methods and by using Olex2 1.5 (Dolomanov et al., 2009) as the graphical interface. The model was refined with ShelXL 2019/3 (Sheldrick, 2015) using full matrix least squares minimisation on $\boldsymbol{F}^2$.

**Crystal Data.** $C_{75}H_{60}F_8N_2Ni$, $M_r$ = 1199.96, monoclinic, $P2_1$ (No. 4), a = 14.6283(12) Å, b = 13.2988(13) Å, c = 15.3812(15) Å, $\beta$ = 94.122(8)°, $\alpha = \gamma$ = 90°, $V$ = 2984.5(5) Å$^3$, $T$ = 140.00(10) K, $Z$ = 2, $Z'$ = 1, $\mu$(Cu $K_\alpha$) = 1.064, 27458 reflections measured, 11132 unique ($R_{int}$ = 0.0410) which were used in all calculations. The final $wR_2$ was 0.1032 (all data) and $R_1$ was 0.0401 (I≥2 $\sigma$(I)).

**CCDC: 2370293**

## Crystal Data and Experimental

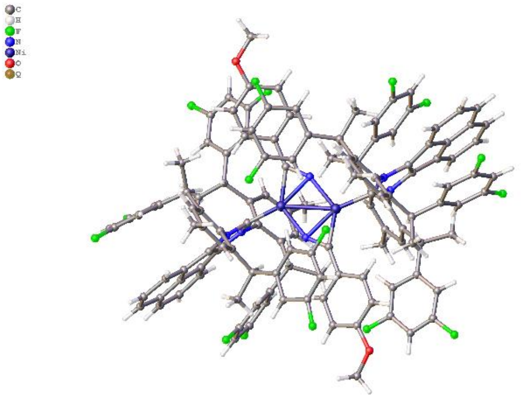

**Experimental.** Single clear intense brown irregular-shaped crystals of **Ni4**(yca-R2139) were used as supplied. A suitable crystal with dimensions 0.51 × 0.14 × 0.10 mm$^3$ was selected and mounted on a SuperNova, Dual, Cu at home/near, Atlas diffractometer. The crystal was kept at a steady $T$ = 200.00(10) K during data collection. The structure was solved with the ShelXT (Sheldrick, 2015) solution program using dual methods and by using Olex2 1.5 (Dolomanov et al., 2009) as the graphical interface. The model was refined with ShelXL 2019/3 (Sheldrick, 2015) using full matrix least squares minimisation on $\boldsymbol{F^2}$.

**Crystal Data.** $C_{146}H_{130}F_{16}N_6Ni_2O_2$, $M_r$ = 2421.97, orthorhombic, $P2_12_12_1$ (No. 19), a = 14.3100(8) Å, b = 25.8231(12) Å, c = 32.595(3) Å, $\alpha = \beta = \gamma = 90^\circ$, $V$ = 12044.8(14) Å$^3$, $T$ = 200.00(10) K, $Z$ = 4, $Z'$ = 1, $\mu$(Cu K$_\alpha$) = 1.074, 24441 reflections measured, 7276 unique ($R_{int}$ = 0.1165) which were used in all calculations. The final $wR_2$ was 0.2323 (all data) and $R_1$ was 0.0851 (I≥2 $\sigma$(I)).

Solvent masking (PLATON/SQUEEZE) information for **Ni4**

| No | x | y | z | V | e | Content |
|---|---|---|---|---|---|---|
| 1 | 0.000 | -0.941 | 0.750 | 979.2 | 186.9 | 4C6H14 |
| 2 | -0.500 | 0.024 | 0.250 | 979.2 | 186.6 | 4C6H14 |

**CCDC: 2370295**

**Explanation of alerts:**

THETM01_ALERT_3_A The value of sine(theta_max)/wavelength is less than 0.550 Calculated sin(theta_max)/wavelength = 0.4166

- Author Response: The crystals diffracted very weakly, and many were tried. No observable diffraction data can be found beyond resolution = 1.2 angstrom. This explains why the data have been cut by the SHEL instruction, causing this alert.

PLAT201_ALERT_2_A Isotropic non-H Atoms in Main Residue(s) ....... 158 Report

F1 F2 F3 F4 F5 F6 F7 F8 F9 F10 F11 F12 F13 F14 F15 F16 O1 O2 N1 N2 N3 N4 N5 N6 C1 C2 C3 C4 C5 C6 C7 C8 C9 C10 C11 C12 C13 C14 C15 C16 C17 C18 C19 C20 C21 C22 C23 C24 C25 C26 C27 C28 C29 C30 C31 C32 C33 C34 C35 C36 C37 C38 C39 C40 C41 C42 C43

C44 C45 C46 C47 C48 C49 C50 C51 C52 C53 C54 C55 C56 C57 C58 C59 C60 C61 C62 C63 C64 C65 C66 C67 C68 C69 C70 C71 C72 C73 C74 C75 C76

- Author Response: Due to the weakness of the diffaction pattern and in order to get an acceptable data/parameter ratio, the light atoms were kept and refined as isotropic.

Alert level B PLAT035_ALERT_1_B _chemical_absolute_configuration Info Not Given Please Do ! PLAT341_ALERT_3_B Low Bond Precision on C-C Bonds ............... 0.03373 Ang.

- Author Response: The diffraction pattern was quite weak, and this causes this type of alerts

**Crystal Data and Experimental**

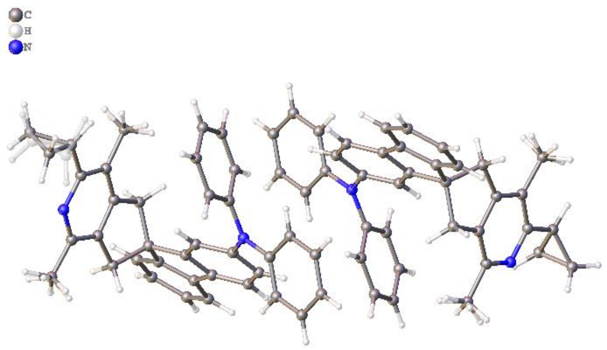


**Experimental.** Single clear pale colourless irregular-shaped crystals of **8m** (yca-R1783) were used as supplied. A suitable crystal with dimensions 0.18 × 0.15 × 0.08 $mm^3$ was selected and mounted on a XtaLAB Synergy R, DW system, HyPix-Arc 150 diffractometer. The crystal was kept at a steady $T$ = 140.00(10) K during data collection. The structure was solved with the ShelXT (Sheldrick, 2015) solution program using dual methods and by using Olex2 1.5 (Dolomanov et al., 2009) as the graphical interface. The model was refined with ShelXL 2019/3 (Sheldrick, 2015) using full matrix least squares minimisation on $\boldsymbol{F}^2$.

**Crystal Data.** $C_{37}H_{32}N_2$, $M_r$ = 504.64, triclinic, $P$1 (No. 1), a = 8.89455(7) Å, b = 9.31775(9) Å, c = 17.10151(16) Å, $\alpha$ = 85.9082(7)°, $\beta$ = 79.1934(7)°, $\gamma$ = 83.0531(7)°, $V$ = 1380.25(2) Å$^3$, $T$ = 140.00(10) K, $Z$ = 2, $Z'$ = 2, $\mu$(Cu $K_\alpha$) = 0.535, 60437 reflections measured, 10696 unique ($R_{int}$ = 0.0238) which were used in all calculations. The final $wR_2$ was 0.0909 (all data) and $R_1$ was 0.0345 (I≥2 $\sigma$(I)

**CCDC: 2370292**

In response to the referee's concern regarding the correct selection of space group (P1 or P(-1)), the refinement results for P-1 were significantly less favorable, with an R-factor of 6.95% compared to 3.45% for P1. Furthermore, the Flack parameter in P1 is very close to zero (-0.06(14)), further supporting the conclusion that the crystal does not contain a racemic mixture. Based on these findings, P1 is apparently the more appropriate space group. For reference, we have included the file **yca_R1783_P-1.res**.

# NMR spectra

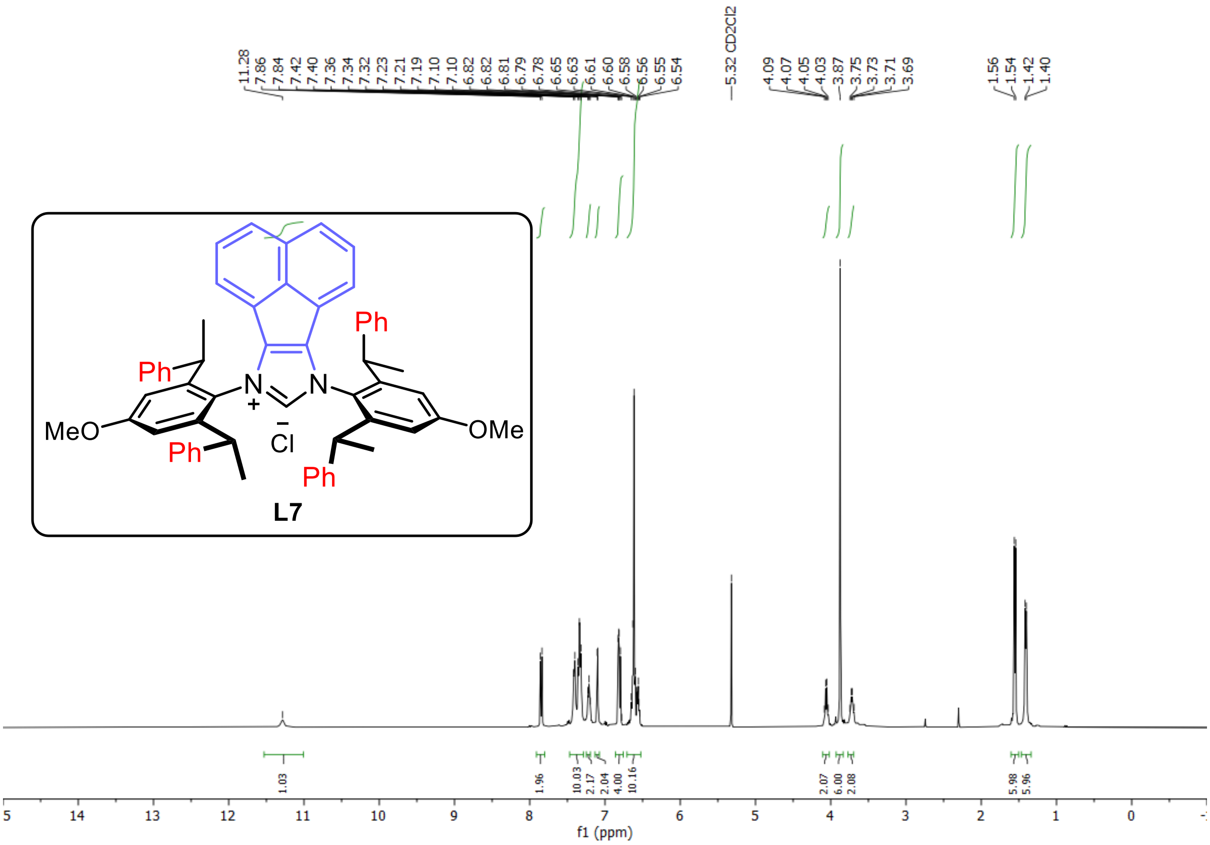
11.28 7.86 7.84 7.42 7.40 7.36 7.34 7.32 7.23 7.21 7.19 7.10 6.82 6.82 6.81 6.79 6.78 6.65 6.63 6.61 6.60 6.58 6.56 6.55 6.54
5.32 CD2Cl2
4.09 4.07 4.05 4.03 3.87 3.75 3.73 3.71 3.69
1.56 1.54 1.42 1.40
Ph
MeO
OMe
Cl
L7
1.03 1.96 10.03 2.17 2.04 4.00 10.16 2.07 6.00 2.08 5.98 5.96
f1 (ppm)

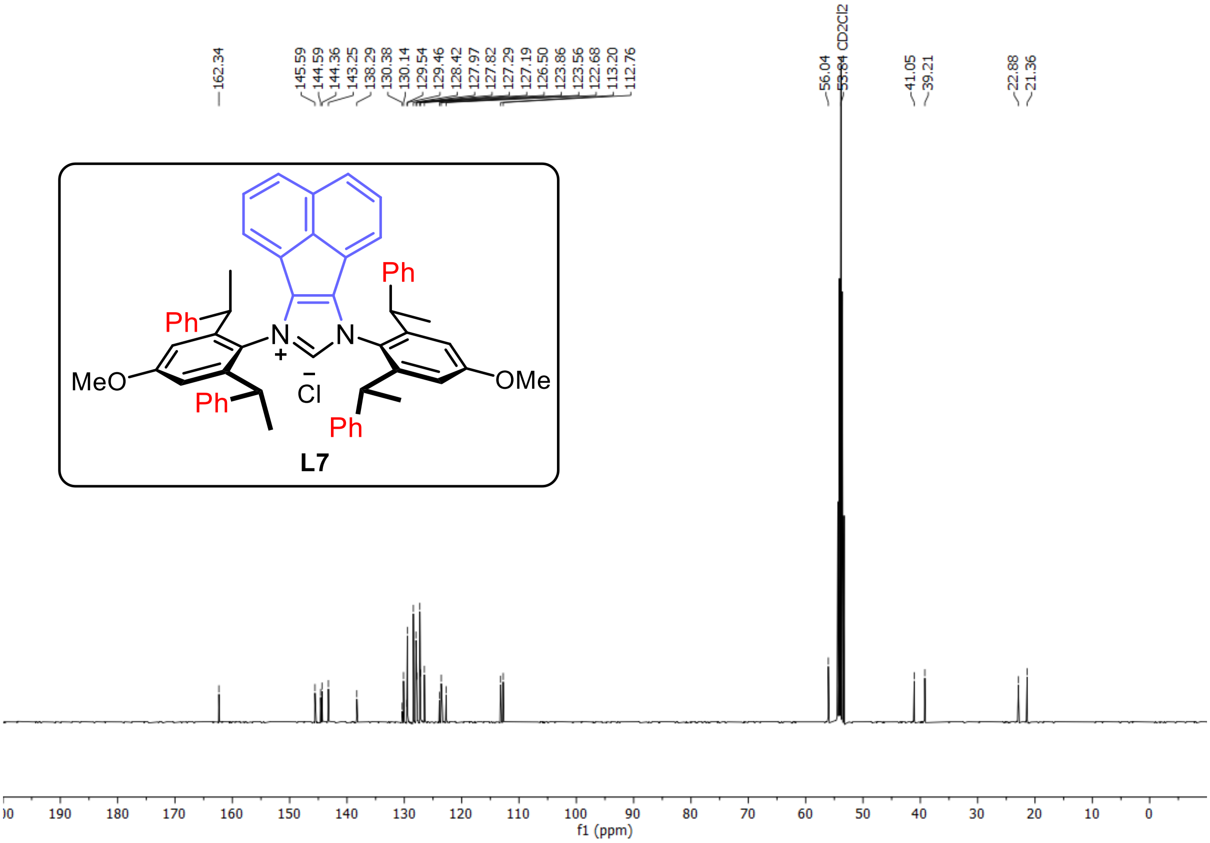
162.34
145.59 144.59 144.36 143.25 138.29 130.38 130.14 129.54 129.46 128.42 127.97 127.82 127.29 127.19 126.50 123.86 123.56 122.68 113.20 112.76
56.04
53.84 CD2Cl2
41.05 39.21
22.88 21.36
Ph
MeO
OMe
Cl
L7
f1 (ppm)

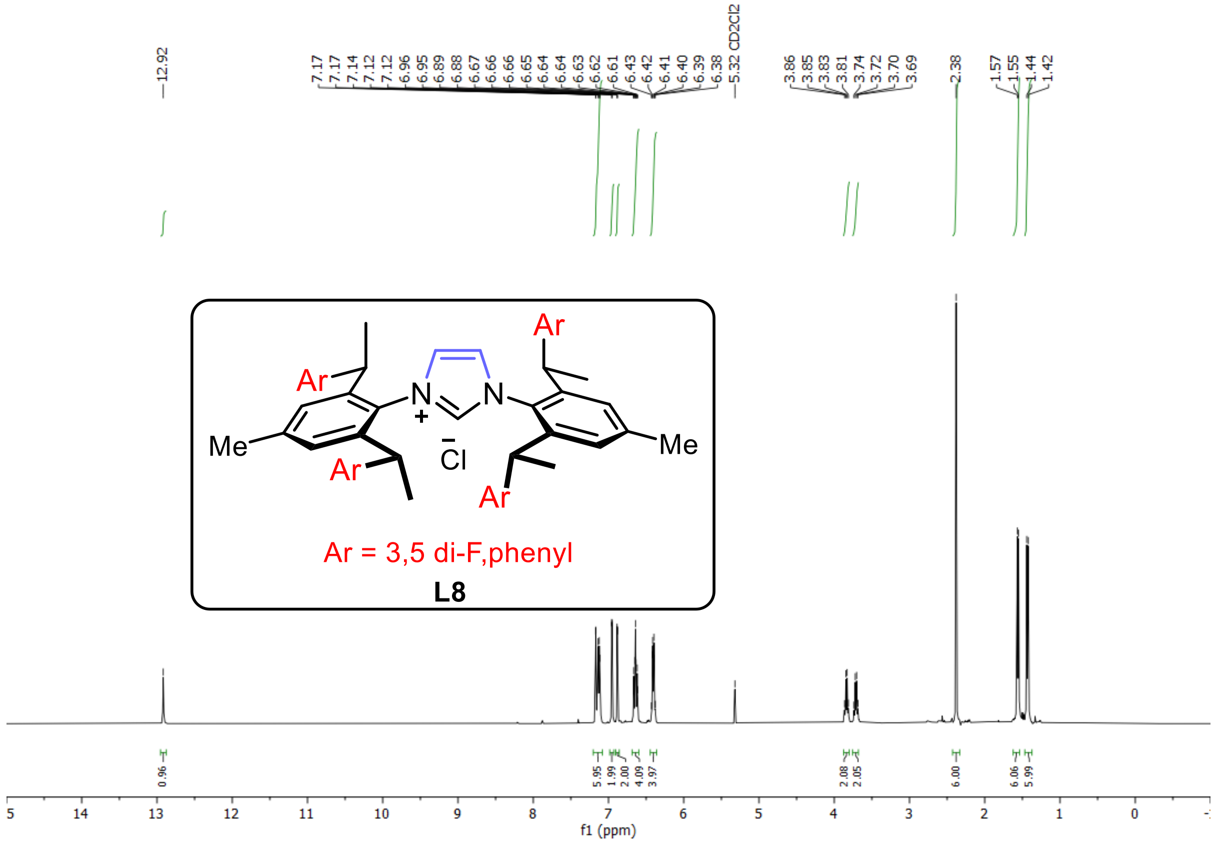
12.92
7.17 7.17 7.14 7.12 7.12 7.12 6.96 6.95 6.89 6.88 6.67 6.66 6.66 6.65 6.64 6.64 6.63 6.62 6.61 6.43 6.42 6.41 6.40 6.39 6.38
5.32 CD2Cl2
3.86 3.85 3.83 3.81 3.74 3.72 3.70 3.69
2.38
1.57 1.55 1.44 1.42
Ar
Me
N
N
Cl
Ar = 3,5 di-F,phenyl
L8
f1 (ppm)

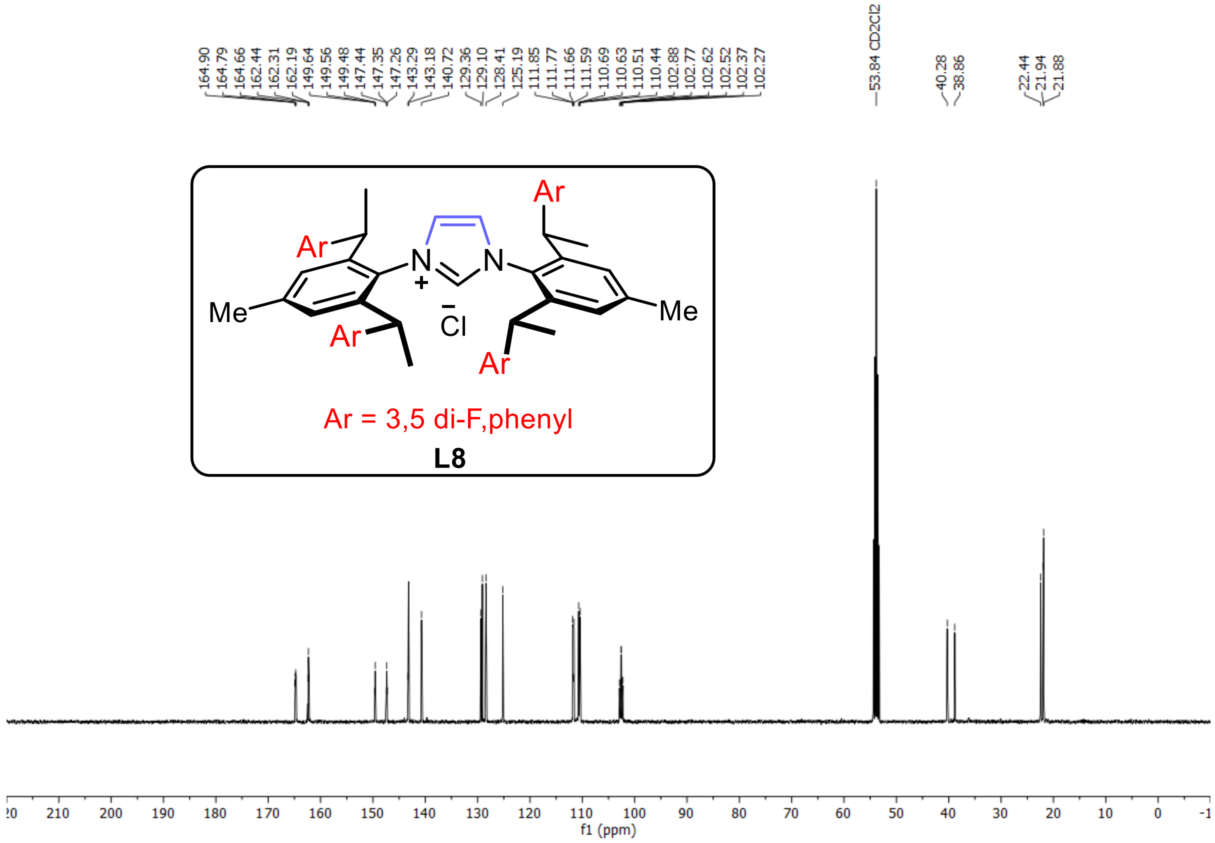
164.90 164.79 164.66 162.44 162.31 162.19 149.64 149.56 149.48 147.44 147.35 147.26 143.29 143.18 140.72 129.36 129.10 128.41 125.19 111.85 111.77 111.66 111.59 110.69 110.63 110.51 110.44 102.88 102.77 102.62 102.52 102.37 102.27
53.84 CD2Cl2
40.28 38.86
22.44 21.94 21.88
Ar
Me
N
N
Cl
Ar = 3,5 di-F,phenyl
L8
f1 (ppm)

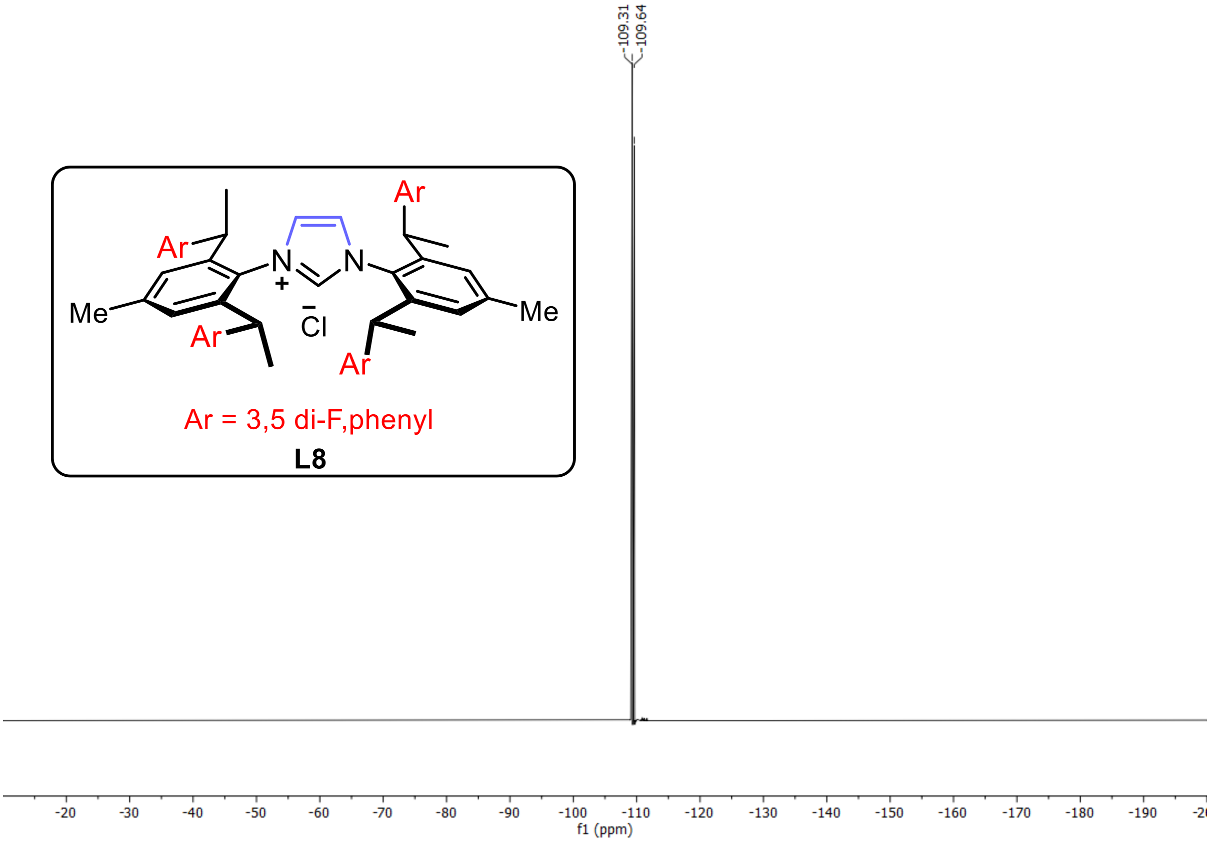

-109.31
-109.64
Ar = 3,5 di-F,phenyl
L8
f1 (ppm)


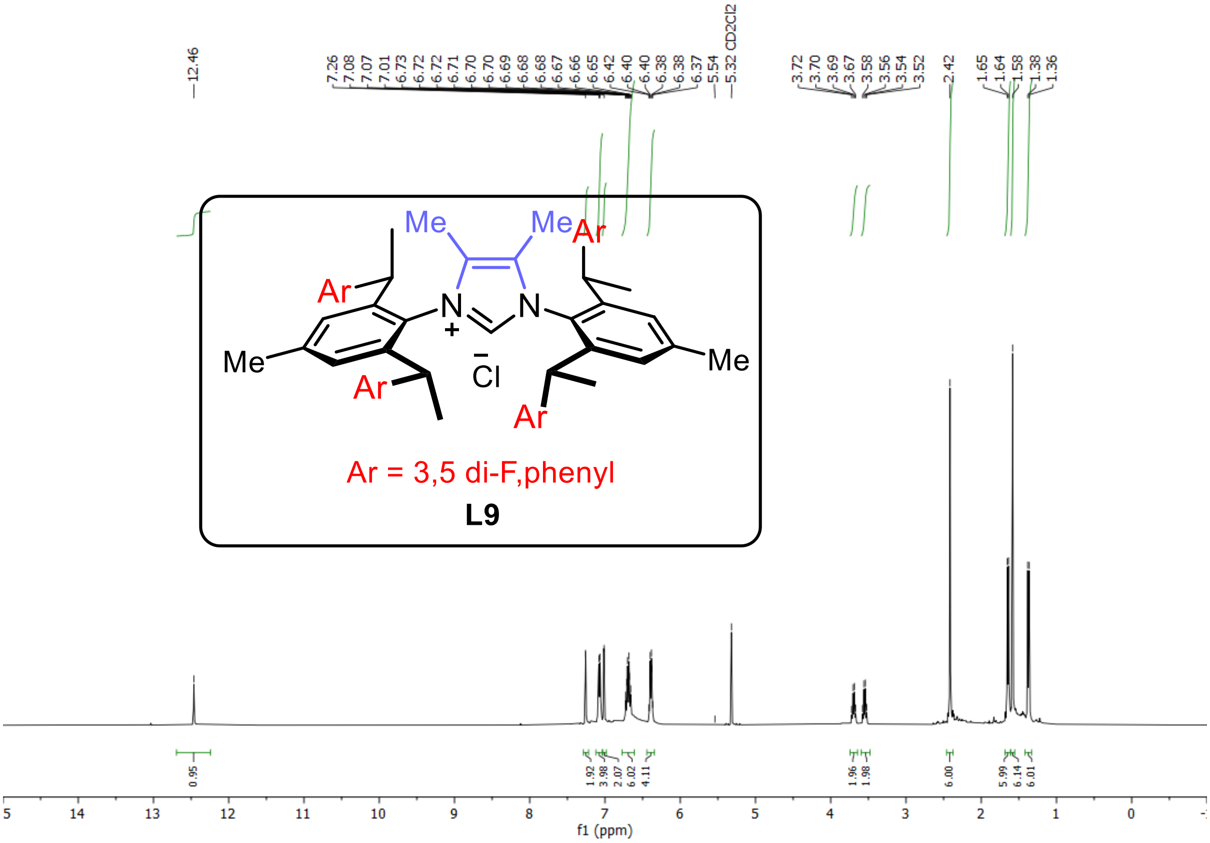

12.46
CD2Cl2
Ar = 3,5 di-F,phenyl
L9
f1 (ppm)

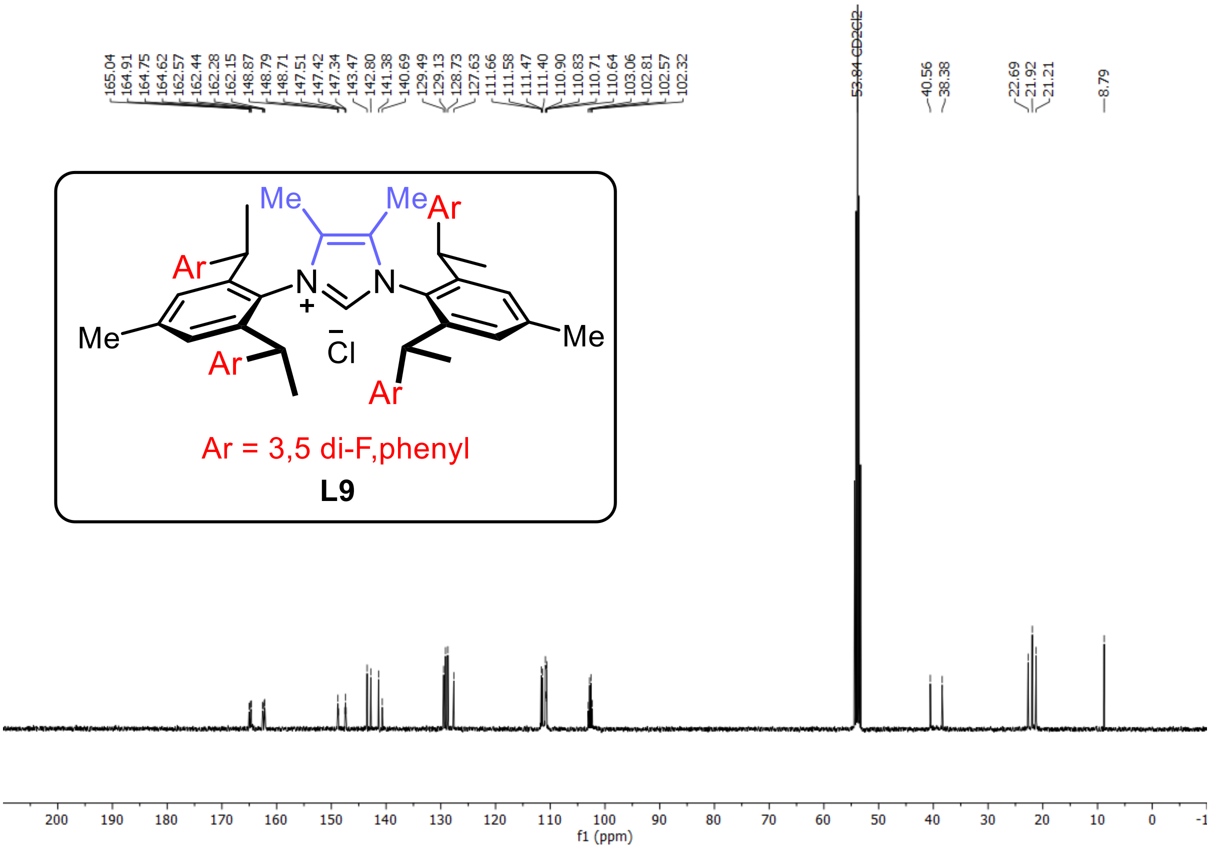

165.04
164.91
164.75
164.62
162.57
162.44
162.28
162.15
148.87
148.79
148.71
147.51
147.42
147.34
143.47
142.80
141.38
140.69
129.49
129.13
128.73
127.63
111.66
111.58
111.47
111.40
110.90
110.83
110.71
110.64
103.06
102.81
102.57
102.32
53.84 CD2Cl2
40.56
38.38
22.69
21.92
21.21
8.79
Me
Me
Ar
Ar
N
N
Cl
Me
Me
Ar
Ar
Ar = 3,5 di-F,phenyl
L9
f1 (ppm)


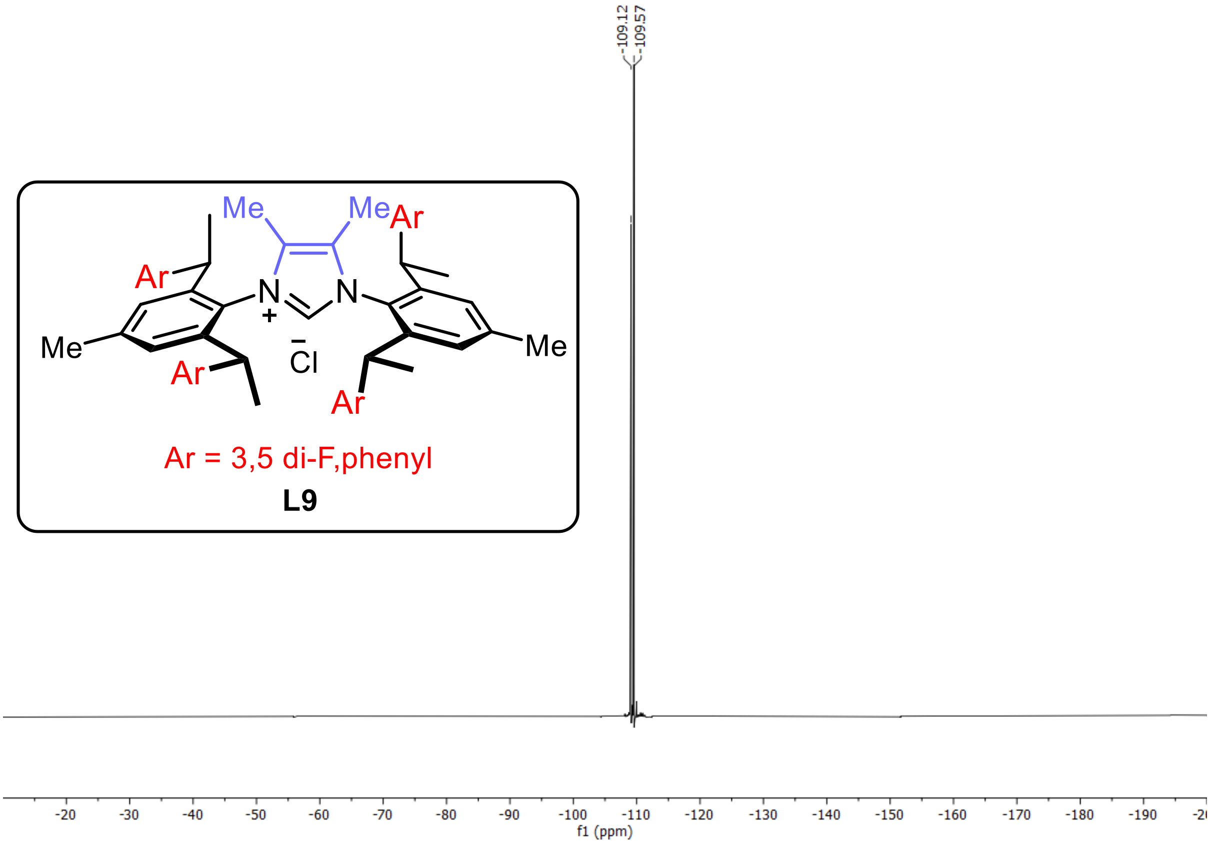

-109.12
-109.57
Me
Me
Ar
Ar
N
N
Cl
Me
Me
Ar
Ar
Ar = 3,5 di-F,phenyl
L9
f1 (ppm)

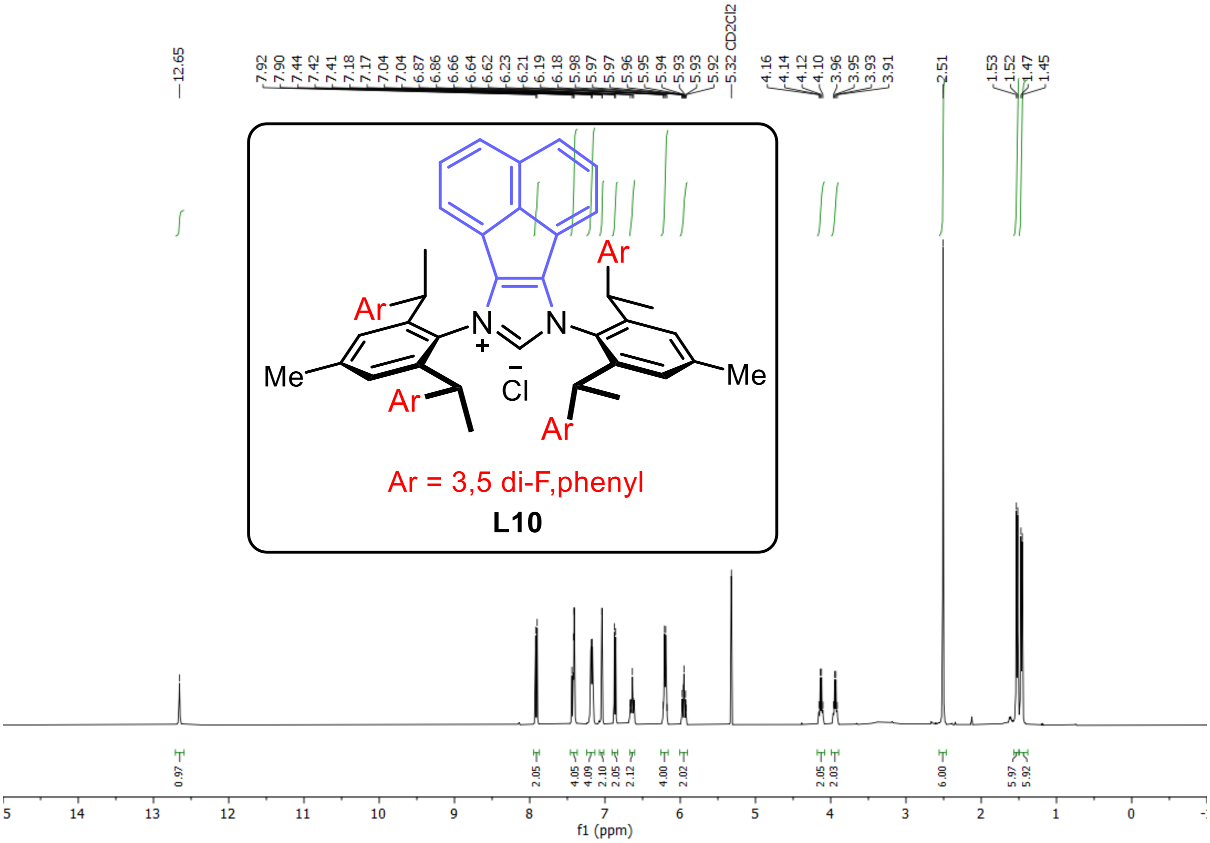

12.65
7.92 7.90 7.44 7.42 7.41 7.18 7.17 7.04 7.04 6.87 6.86 6.66 6.64 6.62 6.23 6.21 6.19 6.18 5.98 5.97 5.97 5.96 5.95 5.94 5.93 5.93 5.92 5.92
5.32 CD2Cl2
4.16 4.14 4.12 4.10 3.96 3.95 3.93 3.91
2.51
1.53 1.52 1.47 1.45
Ar
Ar
Ar
Ar
N
N
+
Cl
Me
Me
Ar = 3,5 di-F,phenyl
L10
0.97 2.05 4.05 4.09 2.10 2.05 2.12 4.00 2.02 2.05 2.03 6.00 5.97 5.92
f1 (ppm)


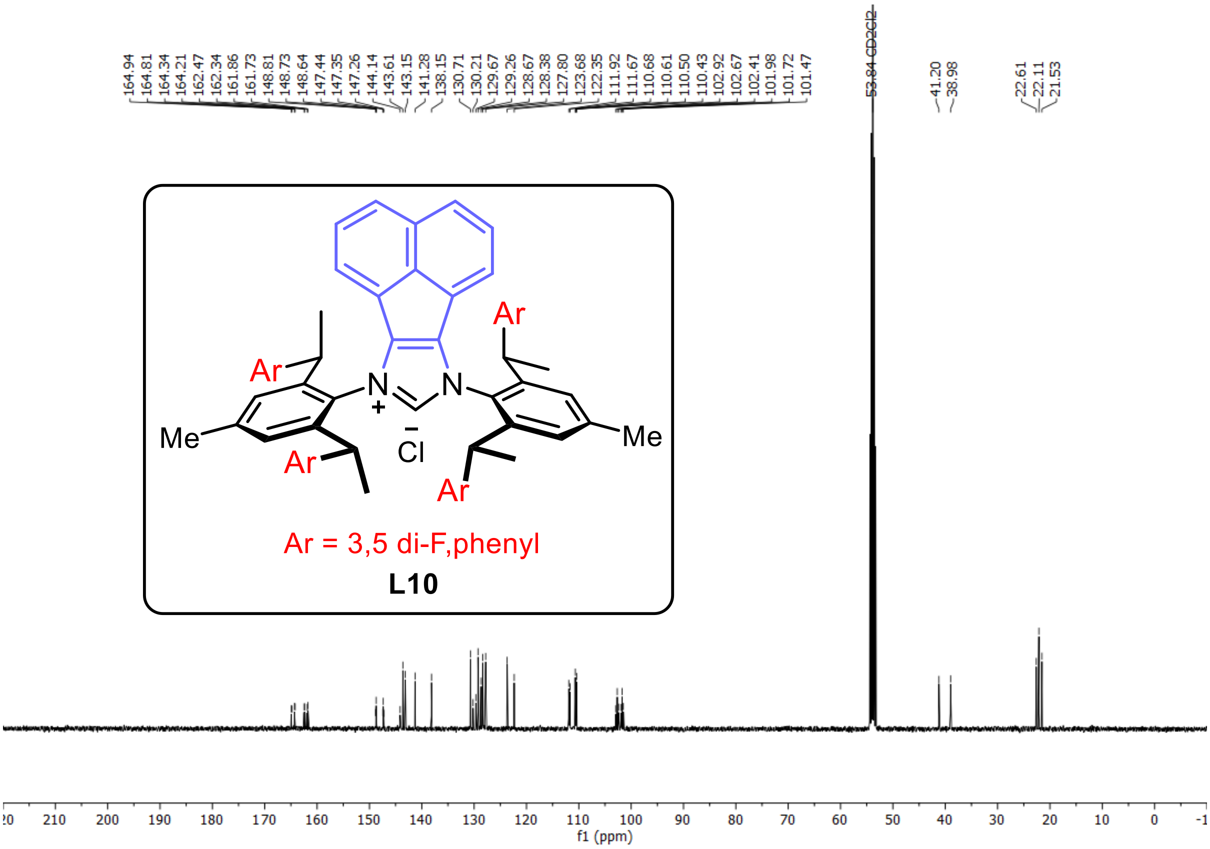

164.94 164.81 164.34 164.21 162.47 162.34 161.86 161.73 148.81 148.73 148.64 147.44 147.35 147.26 144.14 143.61 143.15 141.28 138.15 130.71 130.21 129.67 129.26 128.67 128.38 127.80 123.68 122.35 111.92 111.67 110.68 110.61 110.50 110.43 102.92 102.67 102.41 101.98 101.72 101.47
53.84 CD2Cl2
41.20 38.98
22.61 22.11 21.53
Ar
Ar
Ar
Ar
N
N
+
Cl
Me
Me
Ar = 3,5 di-F,phenyl
L10
f1 (ppm)

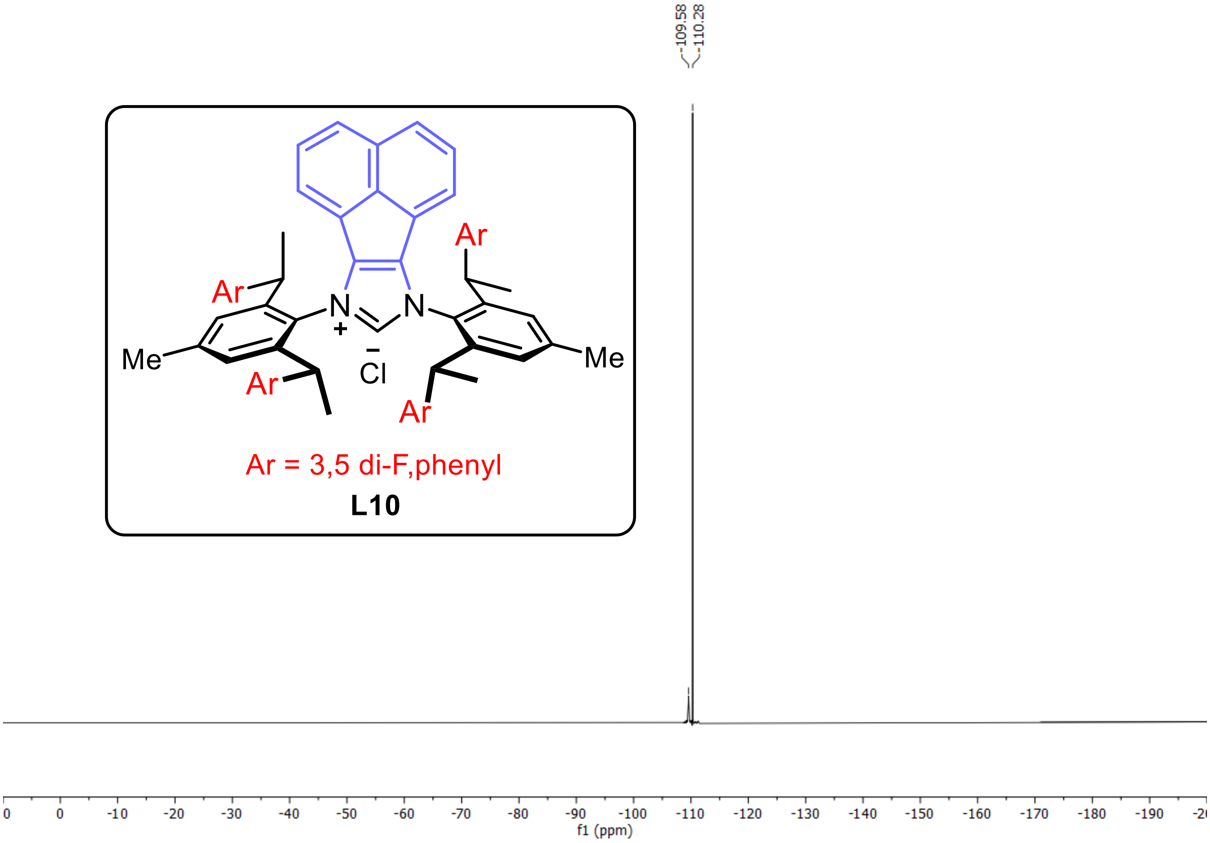
Ar = 3,5 di-F,phenyl
L10
f1 (ppm)

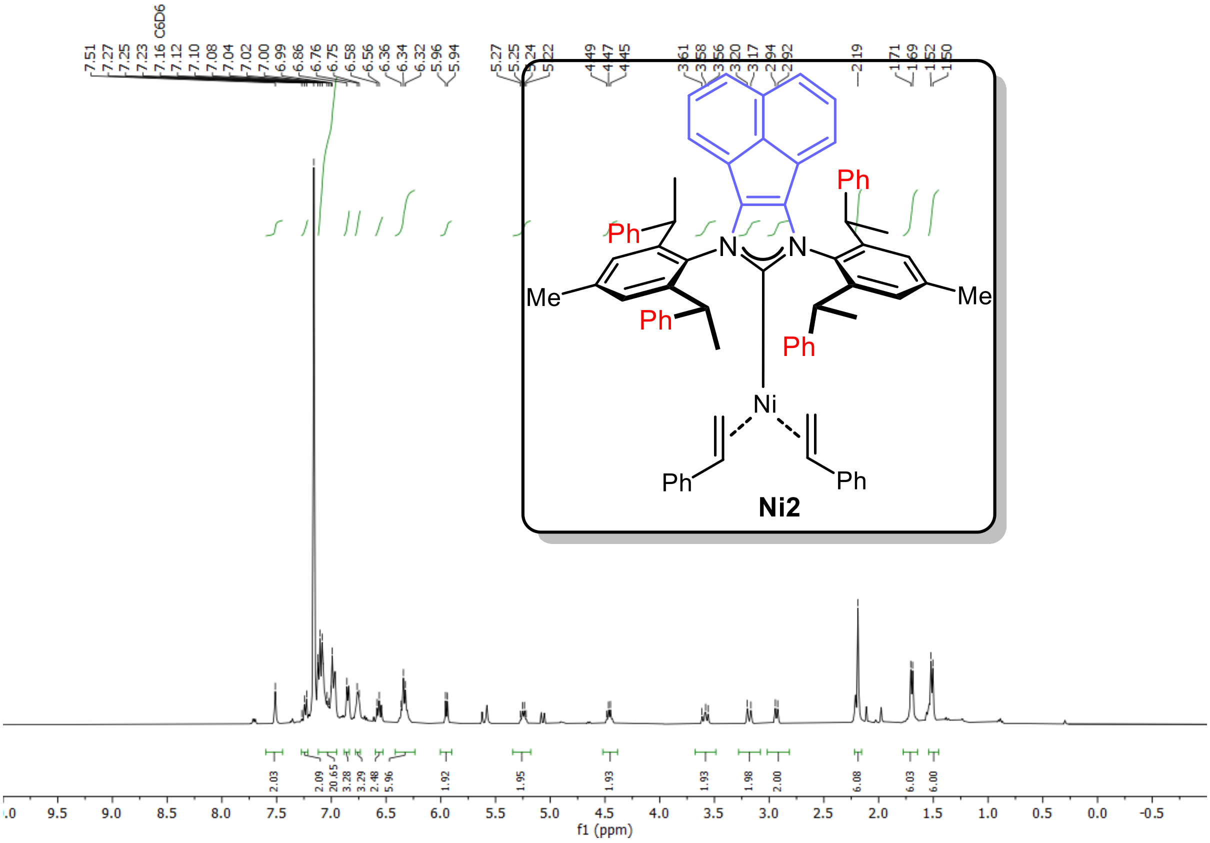
Ni2
f1 (ppm)

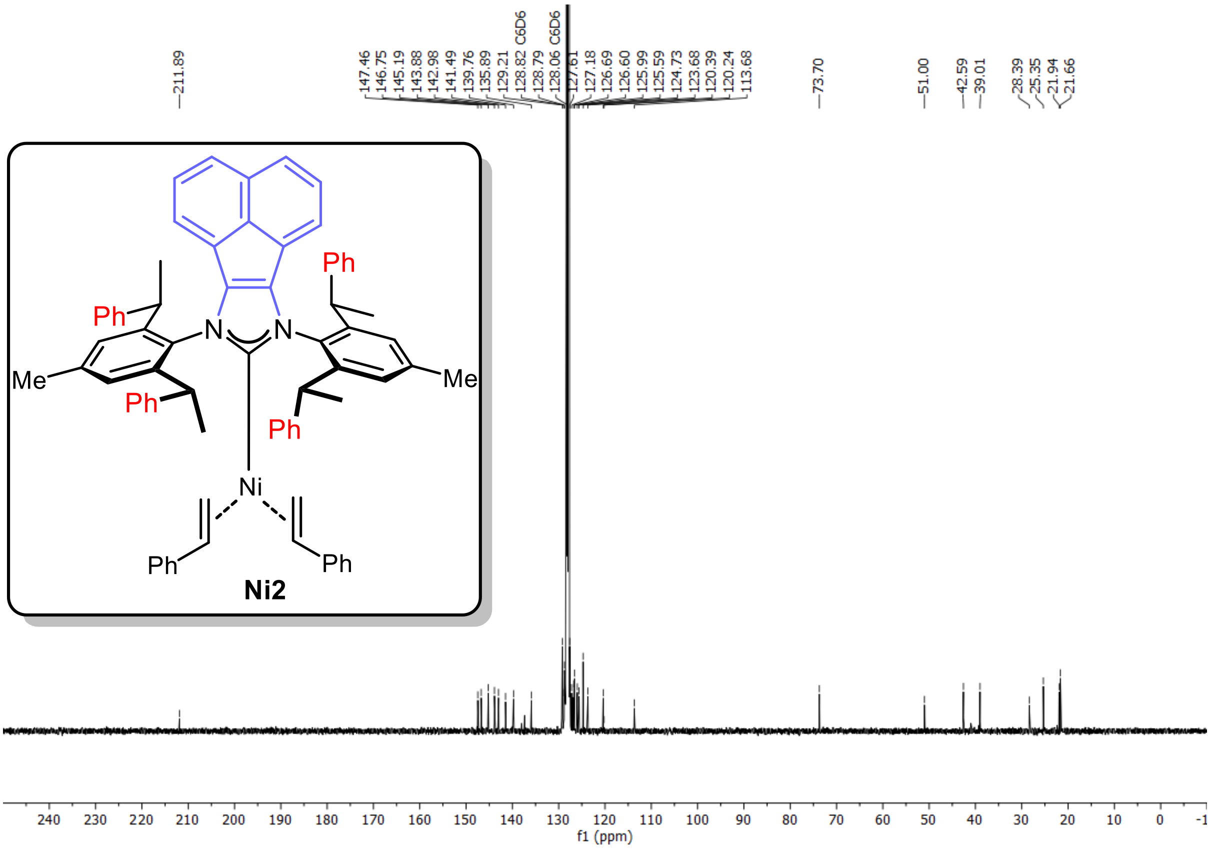

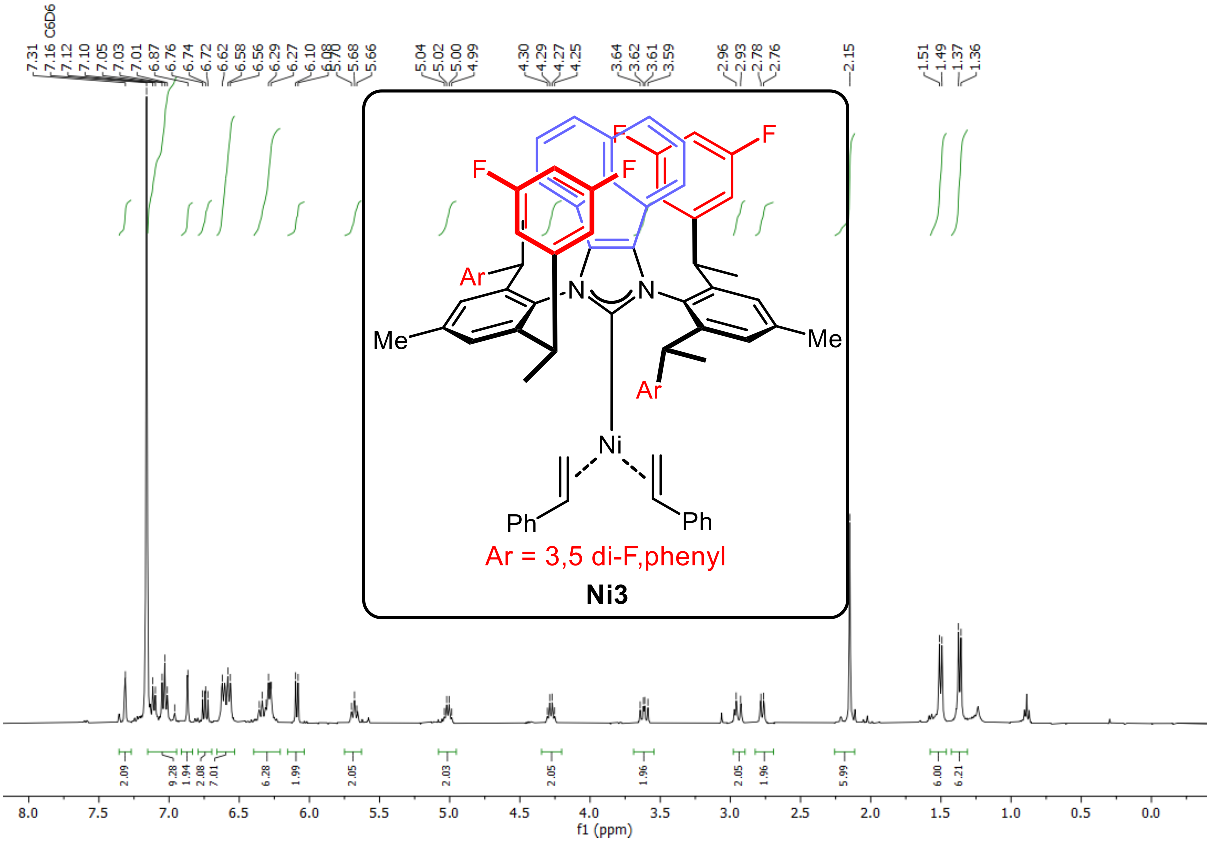

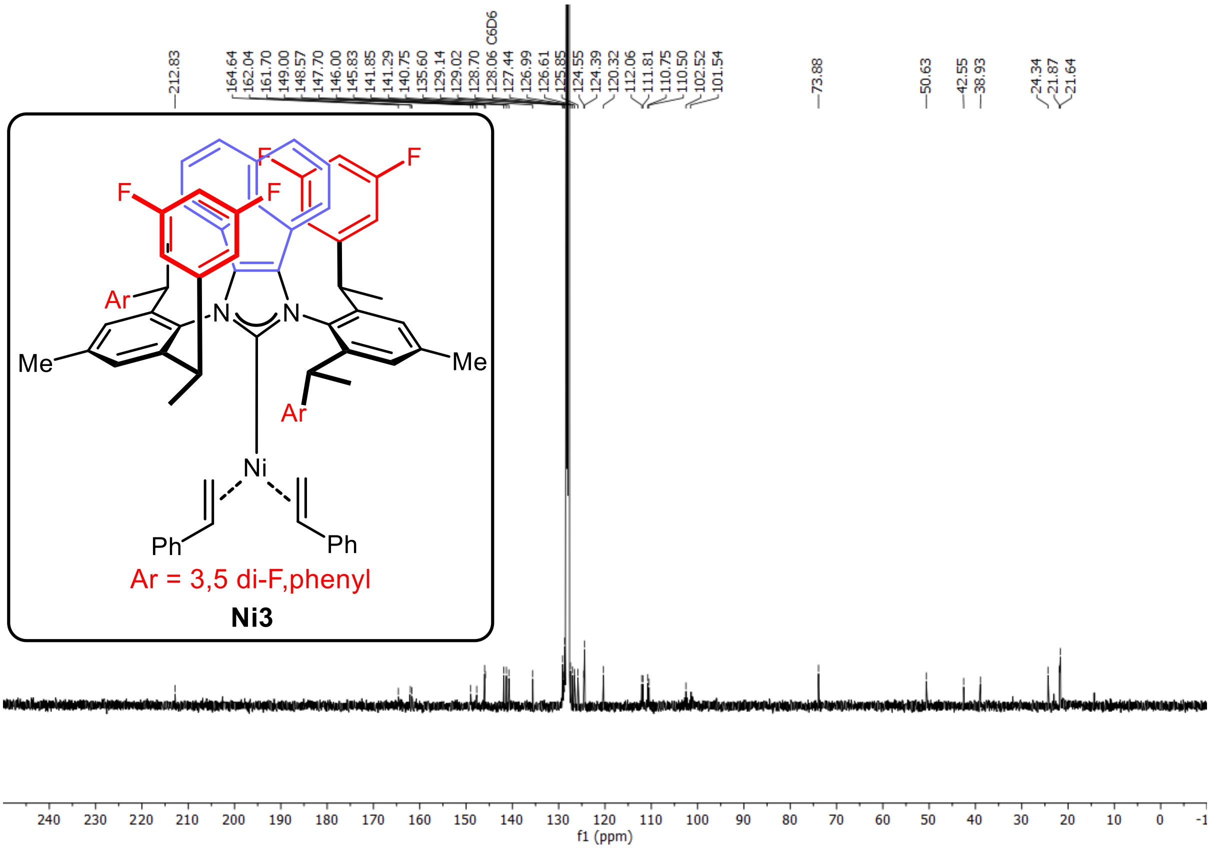
212.83
164.64
162.04
161.70
149.00
148.57
147.70
146.00
145.83
141.85
141.29
140.75
135.60
129.14
129.02
128.70
128.06 C6D6
127.44
126.99
126.61
125.85
124.55
124.39
120.32
112.06
111.81
110.75
110.50
102.52
101.54
73.88
50.63
42.55
38.93
24.34
21.87
21.64
F
F
F
Ar
N
N
Me
Me
Ar
Ni
Ph
Ph
Ar = 3,5 di-F,phenyl
Ni3
f1 (ppm)

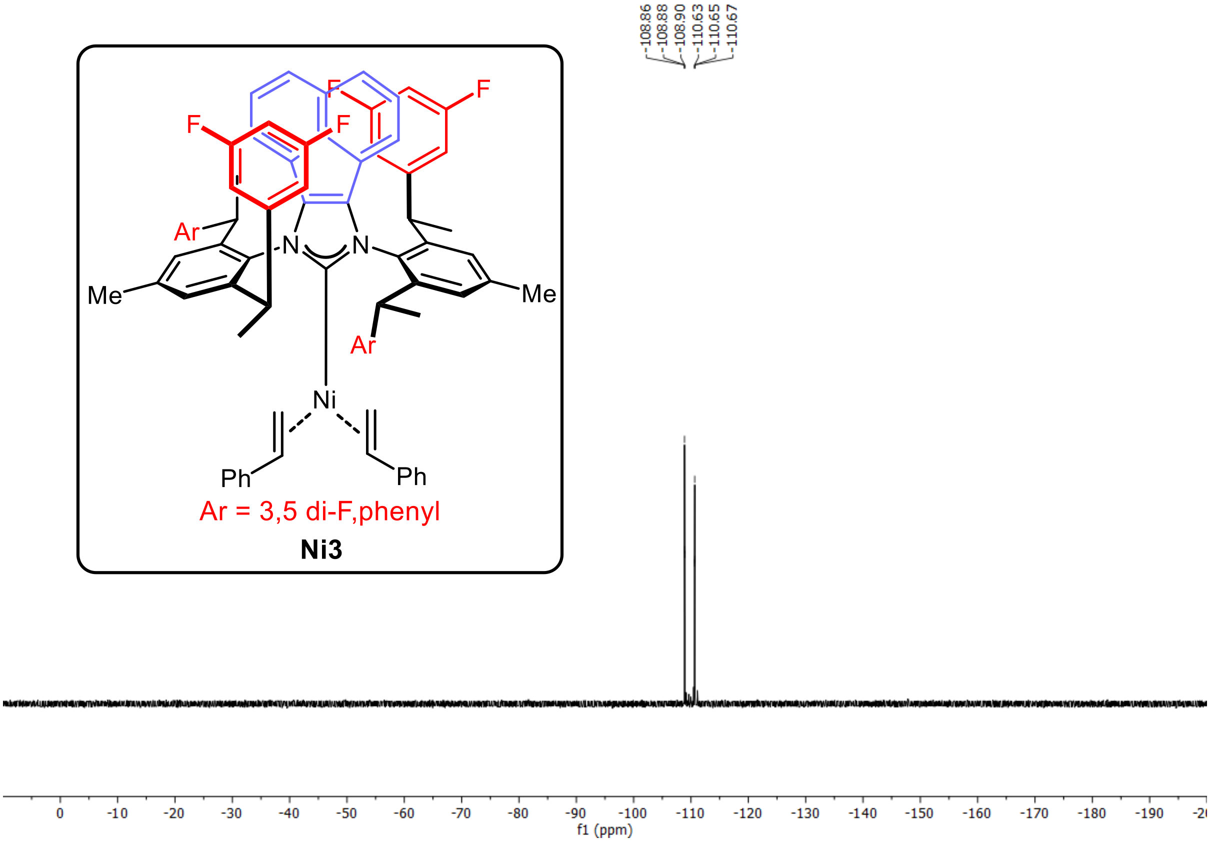
-108.86
-108.88
-108.90
-110.63
-110.65
-110.67
F
F
F
Ar
N
N
Me
Me
Ar
Ni
Ph
Ph
Ar = 3,5 di-F,phenyl
Ni3
f1 (ppm)

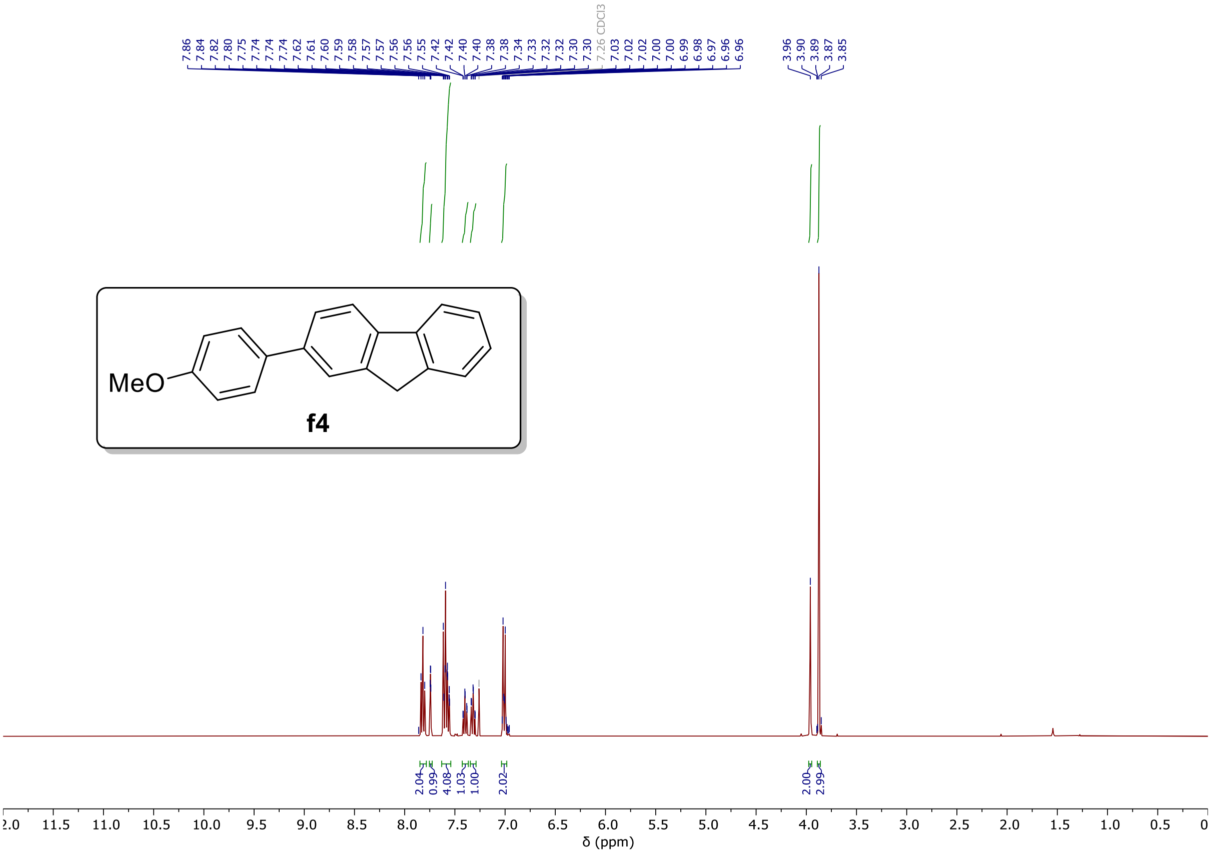
7.86
7.84
7.82
7.80
7.75
7.74
7.74
7.74
7.62
7.61
7.60
7.59
7.58
7.57
7.57
7.56
7.56
7.55
7.42
7.42
7.40
7.40
7.38
7.38
7.34
7.33
7.32
7.32
7.30
7.30
7.26 CDCl3
7.03
7.02
7.02
7.00
7.00
6.99
6.98
6.97
6.96
6.96
3.96
3.90
3.89
3.87
3.85
MeO
f4
2.04
0.99
4.08
1.03
1.00
2.02
2.00
2.99
δ (ppm)

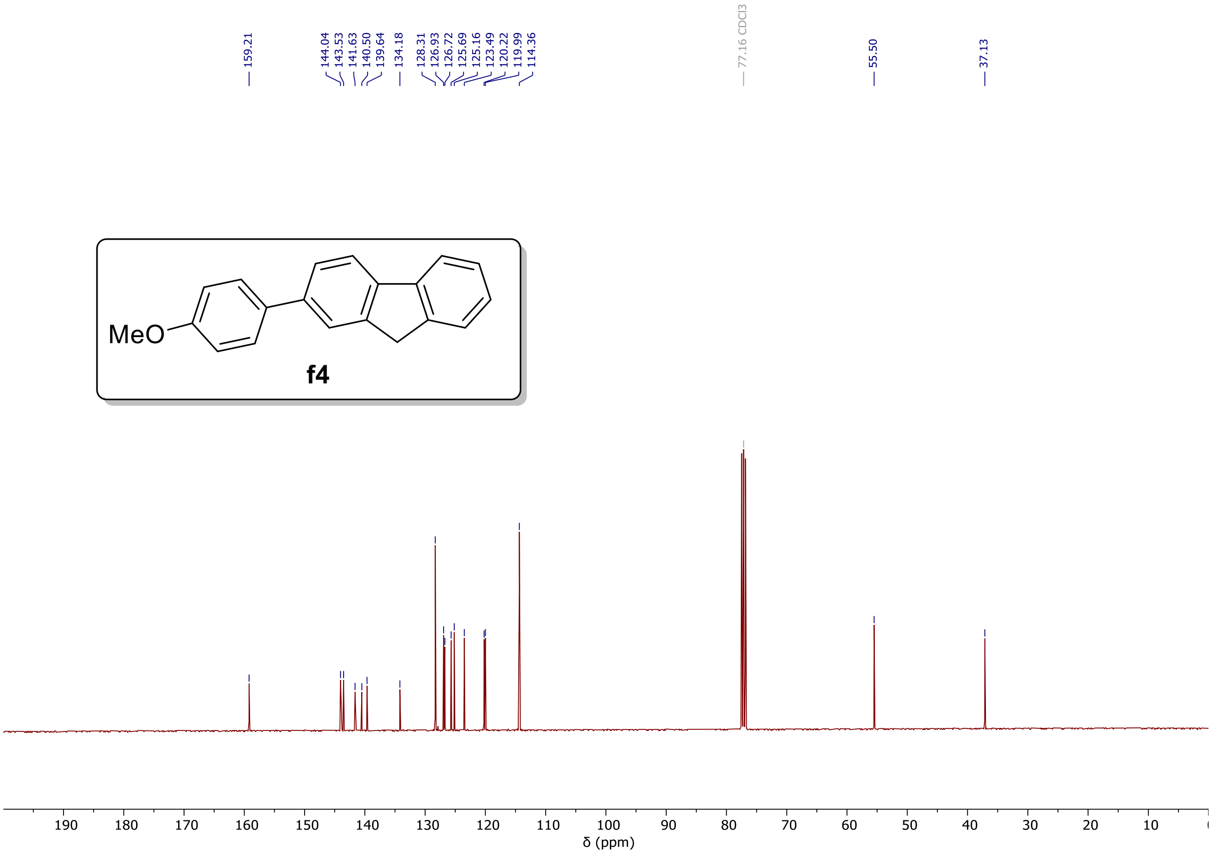
159.21
144.04
143.53
141.63
140.50
139.64
134.18
128.31
126.93
126.72
125.69
125.16
123.49
120.22
119.99
114.36
77.16 CDCl3
55.50
37.13
MeO
f4
δ (ppm)

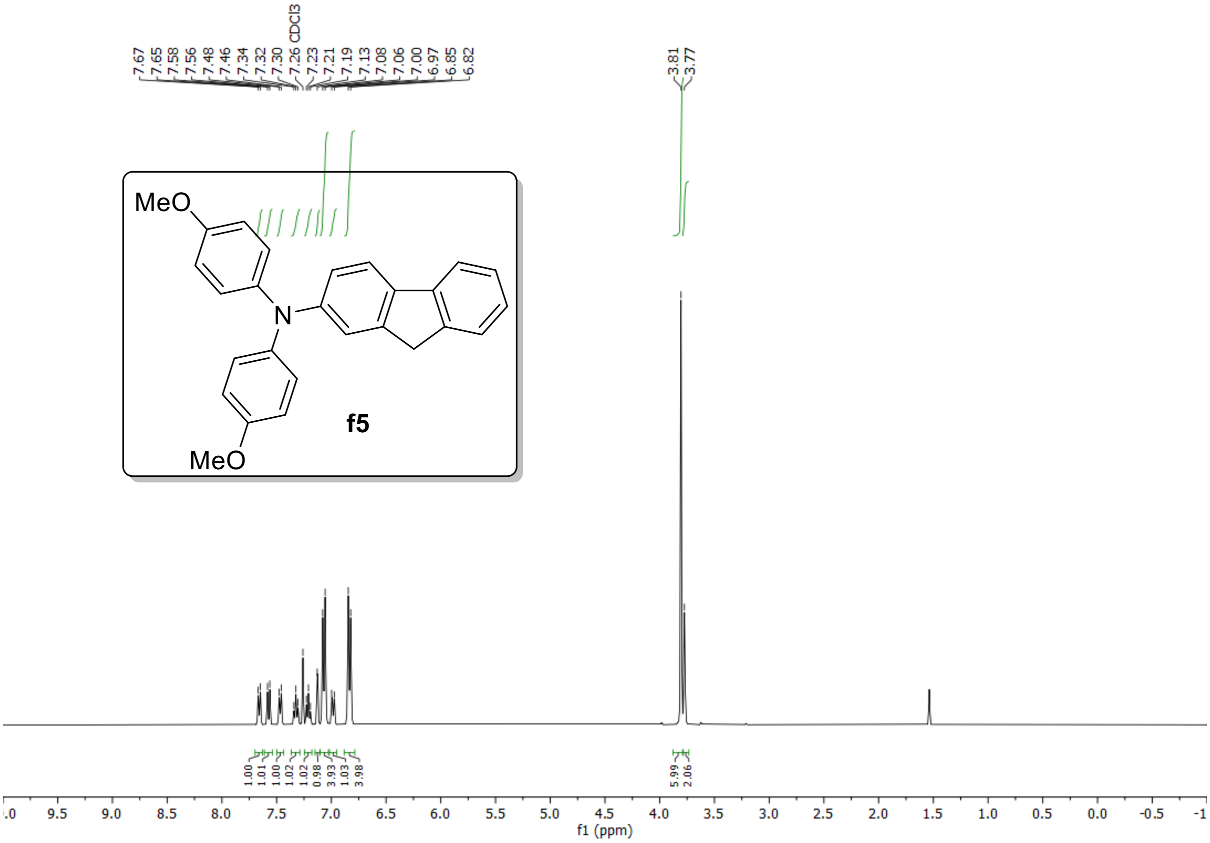
7.67
7.65
7.58
7.56
7.48
7.46
7.34
7.32
7.30
7.26 CDCl3
7.23
7.21
7.19
7.13
7.08
7.06
7.00
6.97
6.85
6.82
3.81
3.77
MeO
N
MeO
f5
1.00
1.01
1.00
1.02
0.98
3.93
1.03
3.98
5.99
2.06
f1 (ppm)

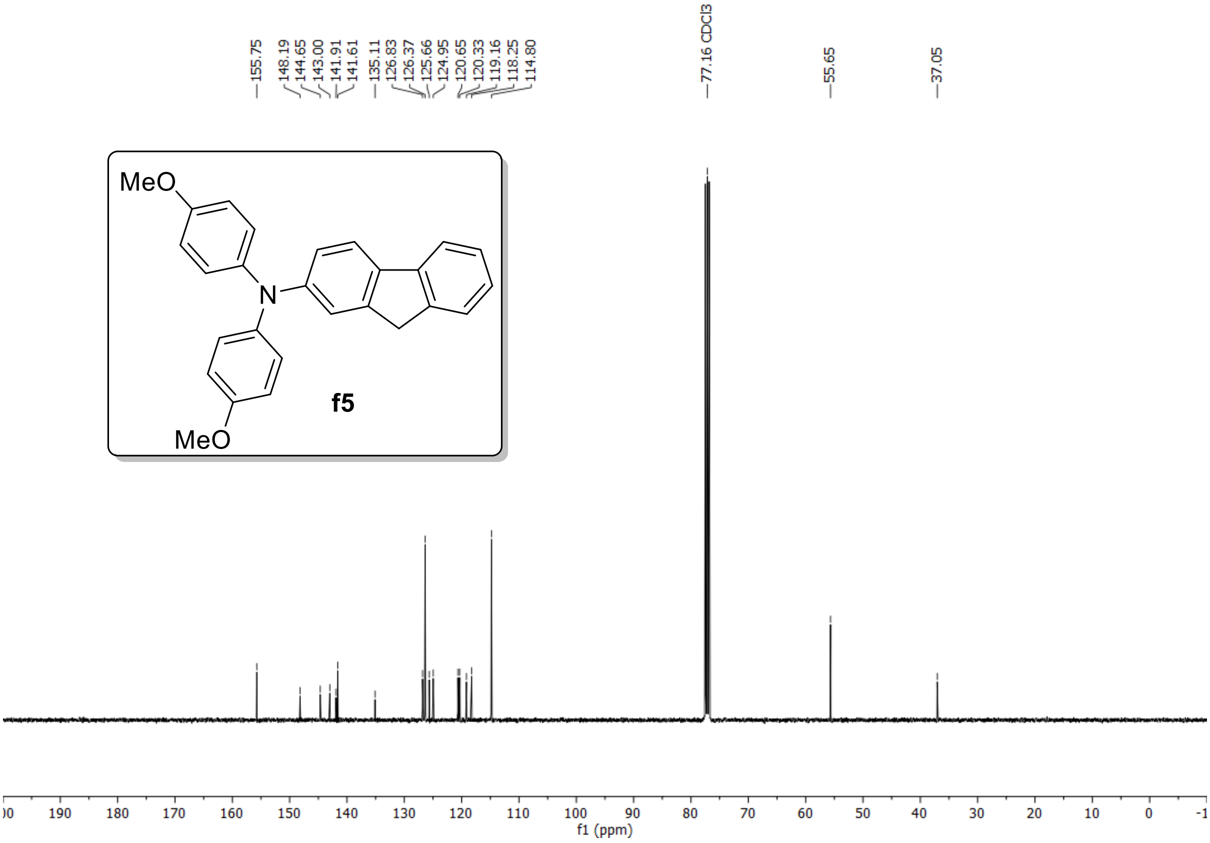
155.75
148.19
144.65
143.00
141.91
141.61
135.11
126.83
126.37
125.66
124.95
120.65
120.33
119.16
118.25
114.80
77.16 CDCl3
55.65
37.05
MeO
N
MeO
f5
f1 (ppm)

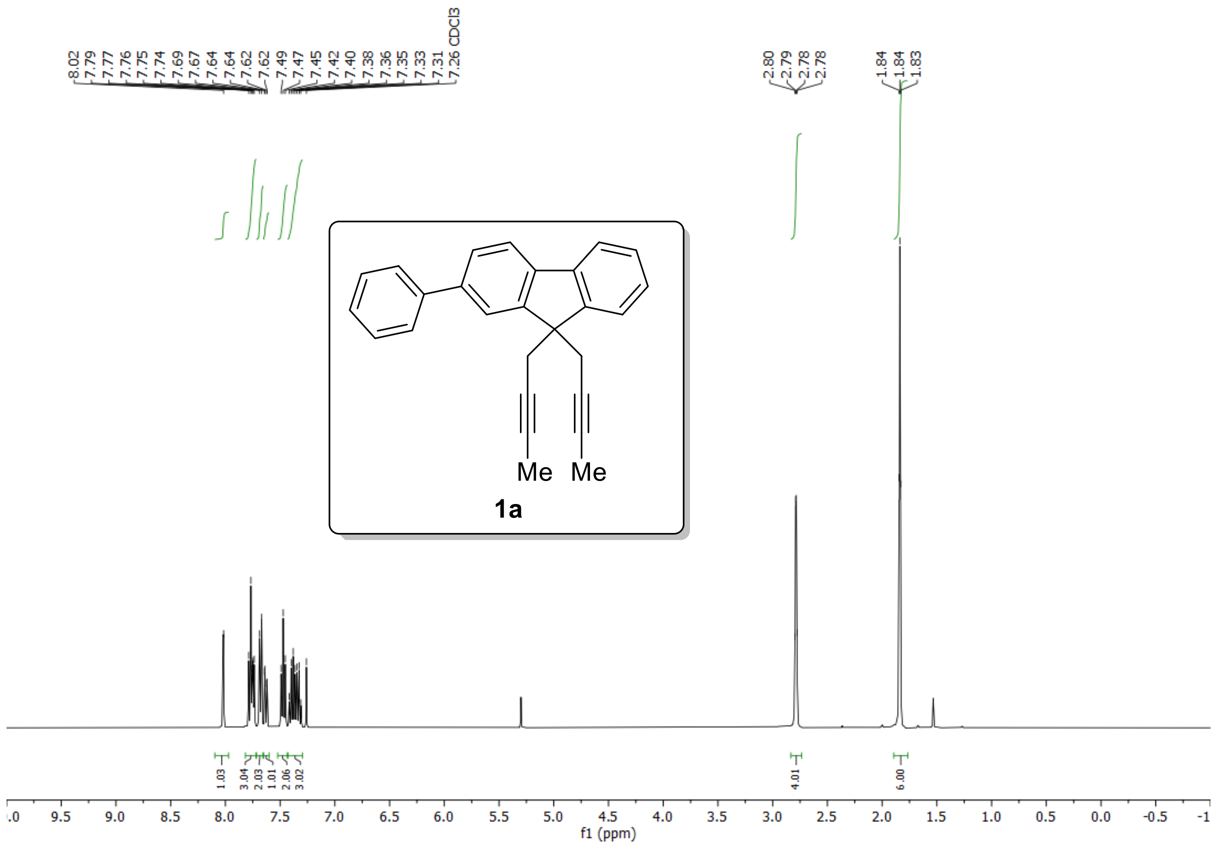
8.02
7.79
7.77
7.76
7.75
7.74
7.69
7.67
7.64
7.64
7.62
7.62
7.49
7.47
7.45
7.42
7.40
7.38
7.36
7.35
7.33
7.31
7.26 CDCl3
2.80
2.79
2.78
2.78
1.84
1.84
1.83
Me Me
1a
1.03
3.04
2.03
1.01
2.06
3.02
4.01
6.00
f1 (ppm)

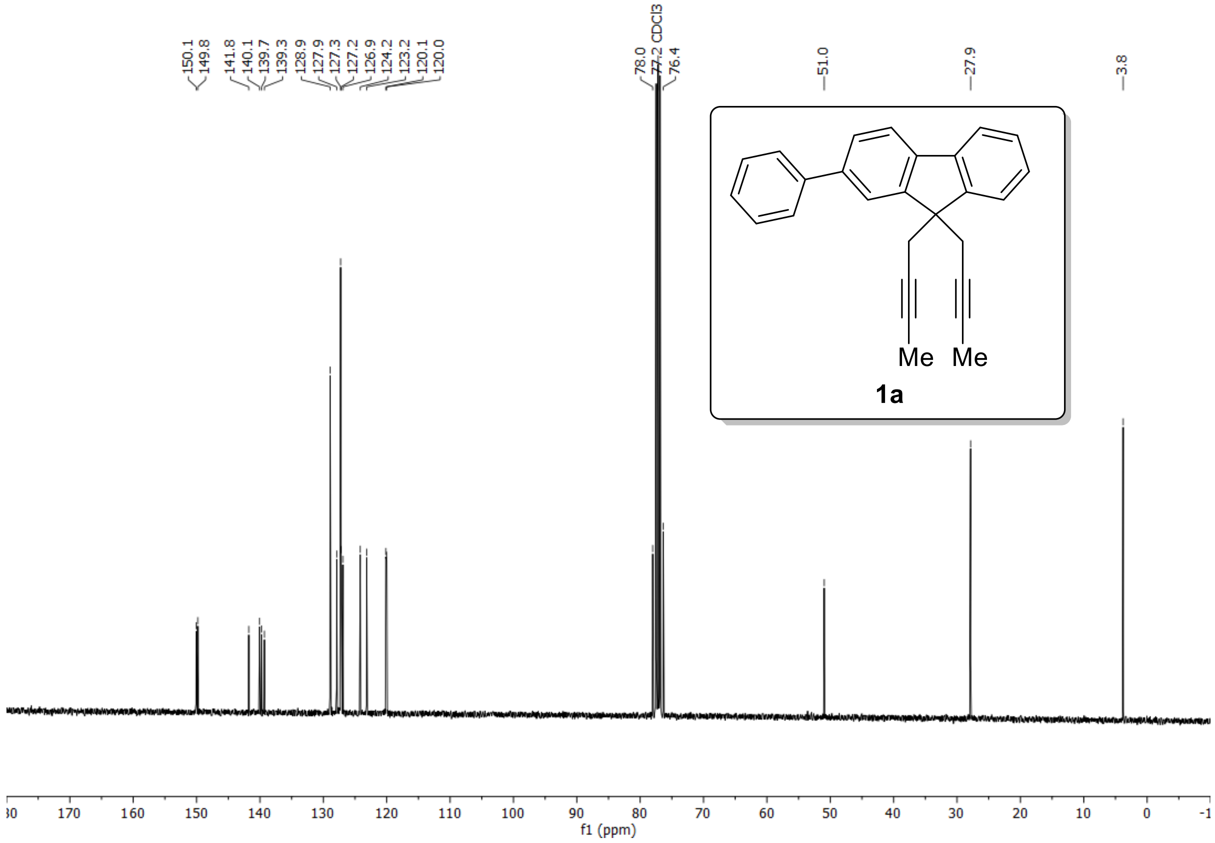
150.1
149.8
141.8
140.1
139.7
139.3
128.9
127.9
127.3
127.2
126.9
124.2
123.2
120.1
120.0
78.0
77.2 CDCl3
76.4
51.0
27.9
3.8
Me Me
1a
f1 (ppm)

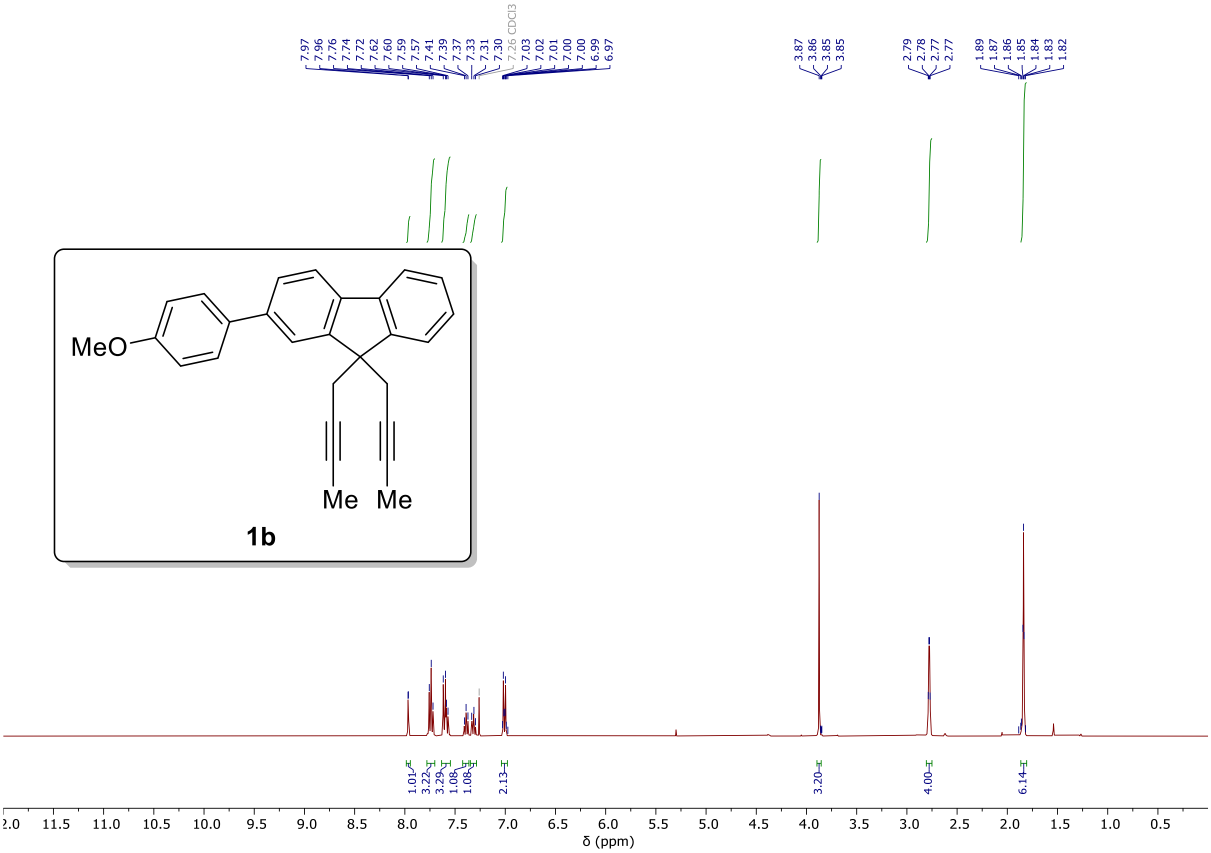
7.97 7.96 7.76 7.74 7.72 7.62 7.60 7.59 7.57 7.41 7.39 7.37 7.33 7.31 7.30 7.26 CDCl3 7.03 7.02 7.01 7.00 7.00 6.99 6.97
3.87 3.86 3.85 3.85
2.79 2.78 2.77 2.77
1.89 1.87 1.86 1.85 1.84 1.83 1.82
MeO
Me Me
1b
1.01 3.22 3.29 1.08 1.08 2.13
3.20
4.00
6.14
δ (ppm)

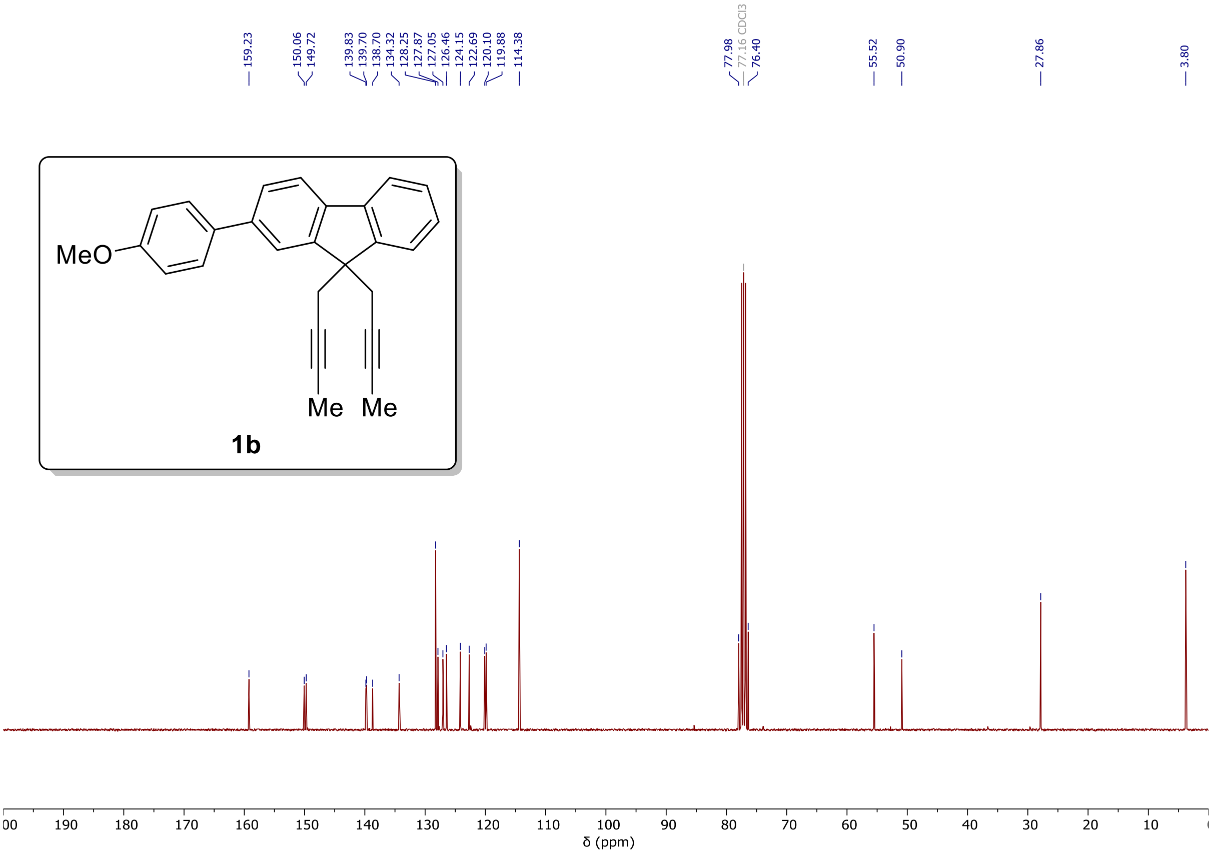
159.23
150.06 149.72
139.83 139.70 138.70 134.32 128.25 127.87 127.05 126.46 124.15 122.69 120.10 119.88 114.38
77.98 77.16 CDCl3 76.40
55.52
50.90
27.86
3.80
MeO
Me Me
1b
δ (ppm)

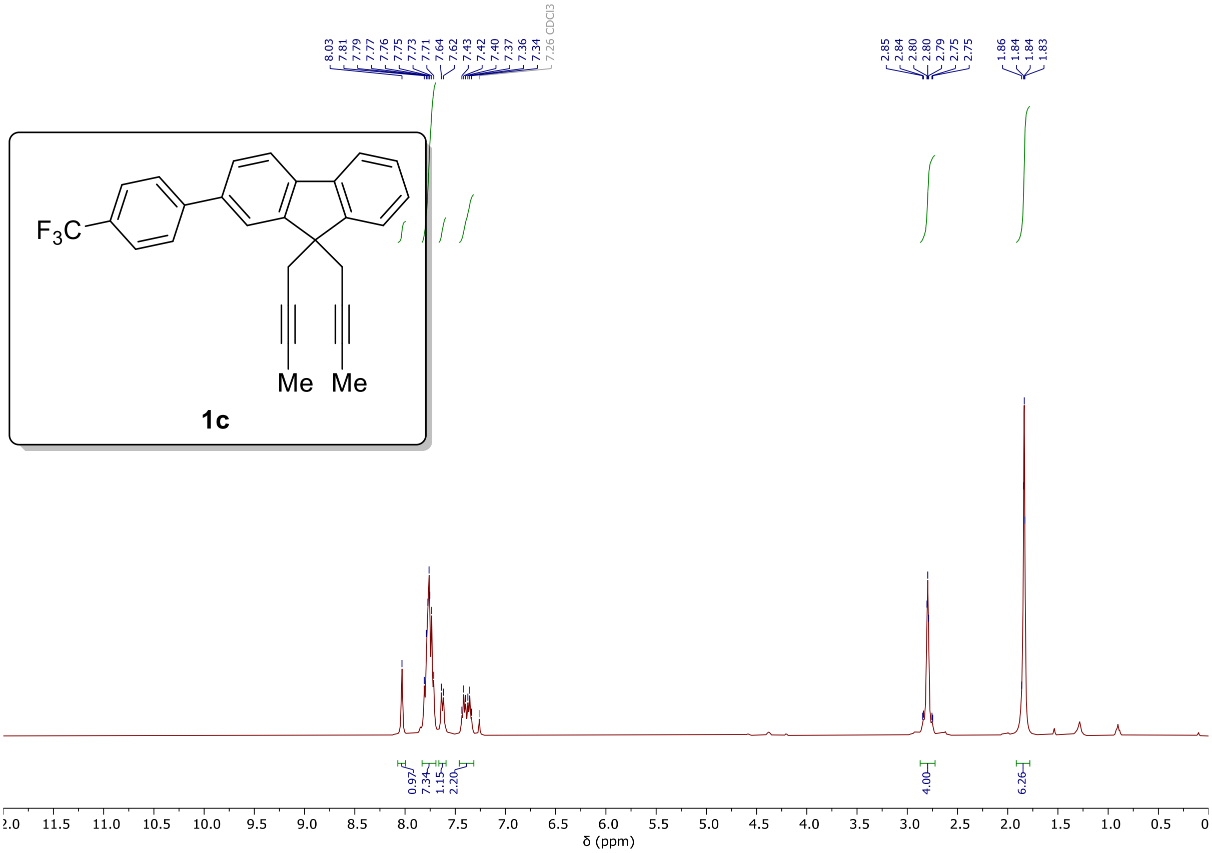
8.03 7.81 7.79 7.77 7.76 7.75 7.73 7.71 7.64 7.62 7.43 7.42 7.40 7.37 7.36 7.34 7.26 CDCl3
2.85 2.84 2.80 2.80 2.79 2.75 2.75
1.86 1.84 1.84 1.83
F3C
Me Me
1c
0.97 7.34 1.15 2.20
4.00
6.26
δ (ppm)

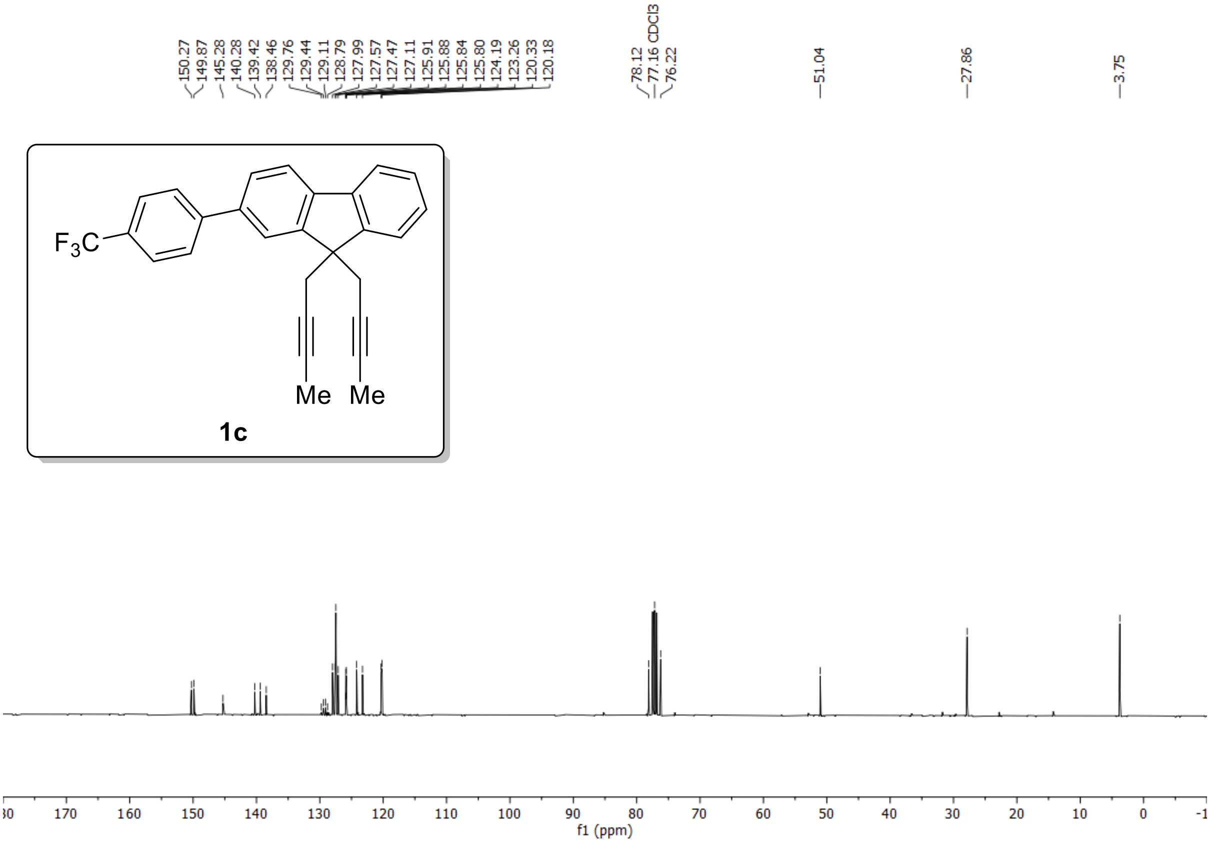
150.27 149.87 145.28 140.28 139.42 138.46 129.76 129.44 129.11 128.79 127.99 127.57 127.47 127.11 125.91 125.88 125.84 125.80 124.19 123.26 120.33 120.18
78.12 77.16 CDCl3 76.22
51.04
27.86
3.75
F3C
Me Me
1c
f1 (ppm)

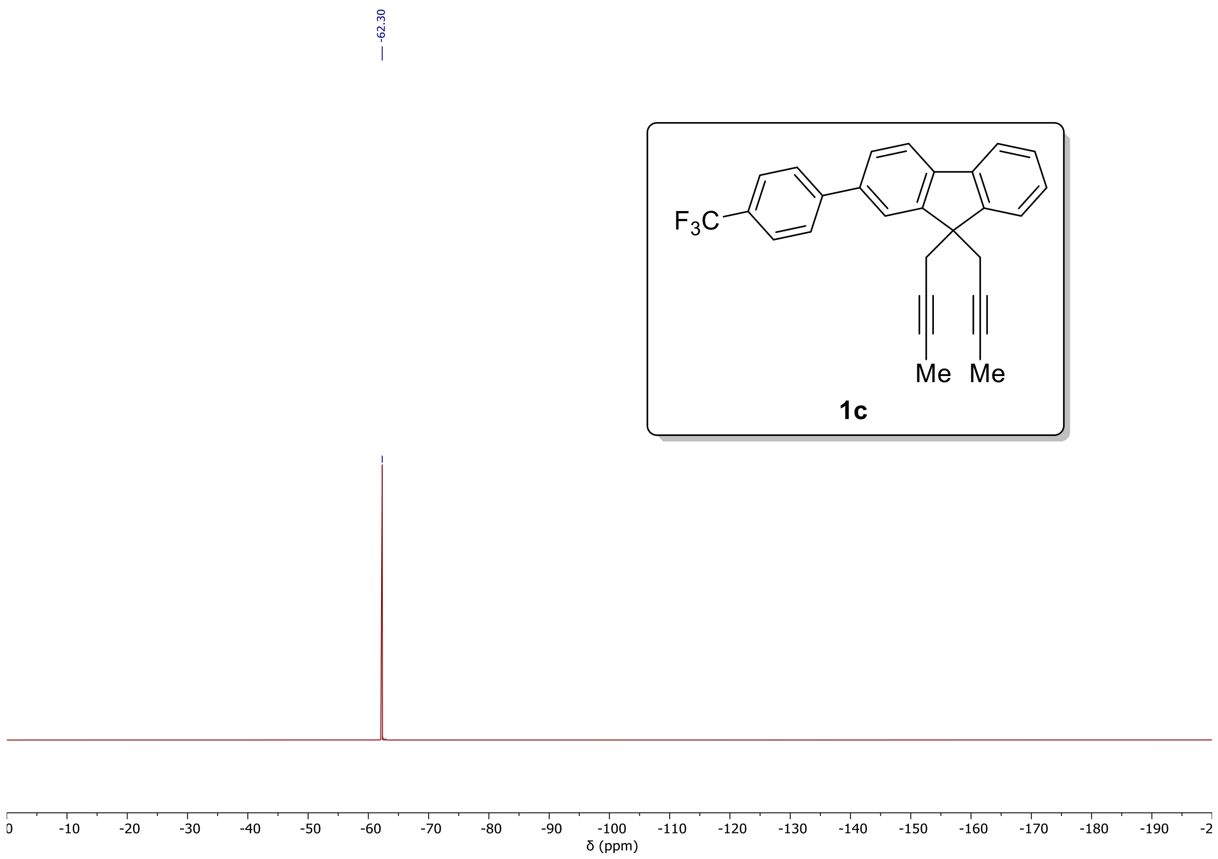
-62.30
F3C
Me Me
1c
δ (ppm)

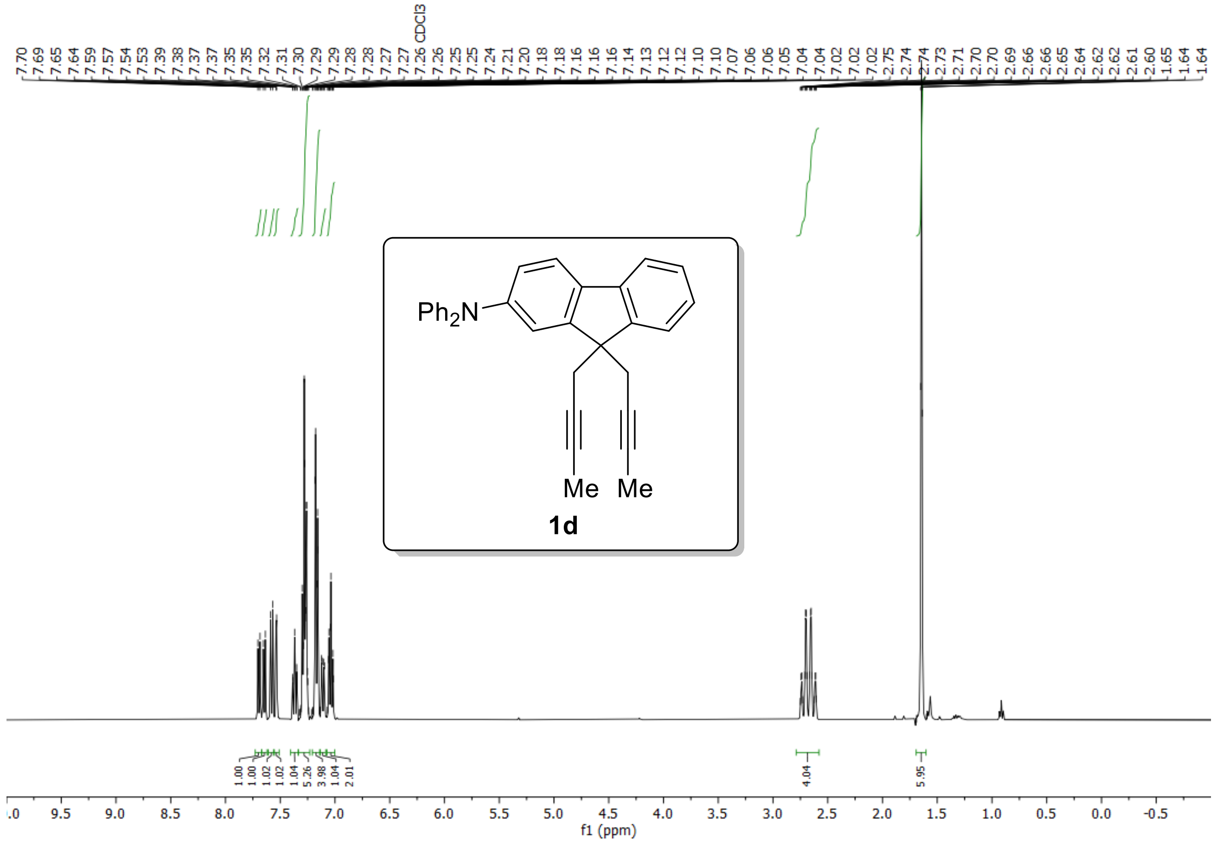
CDCl3
Ph2N
Me Me
1d
f1 (ppm)

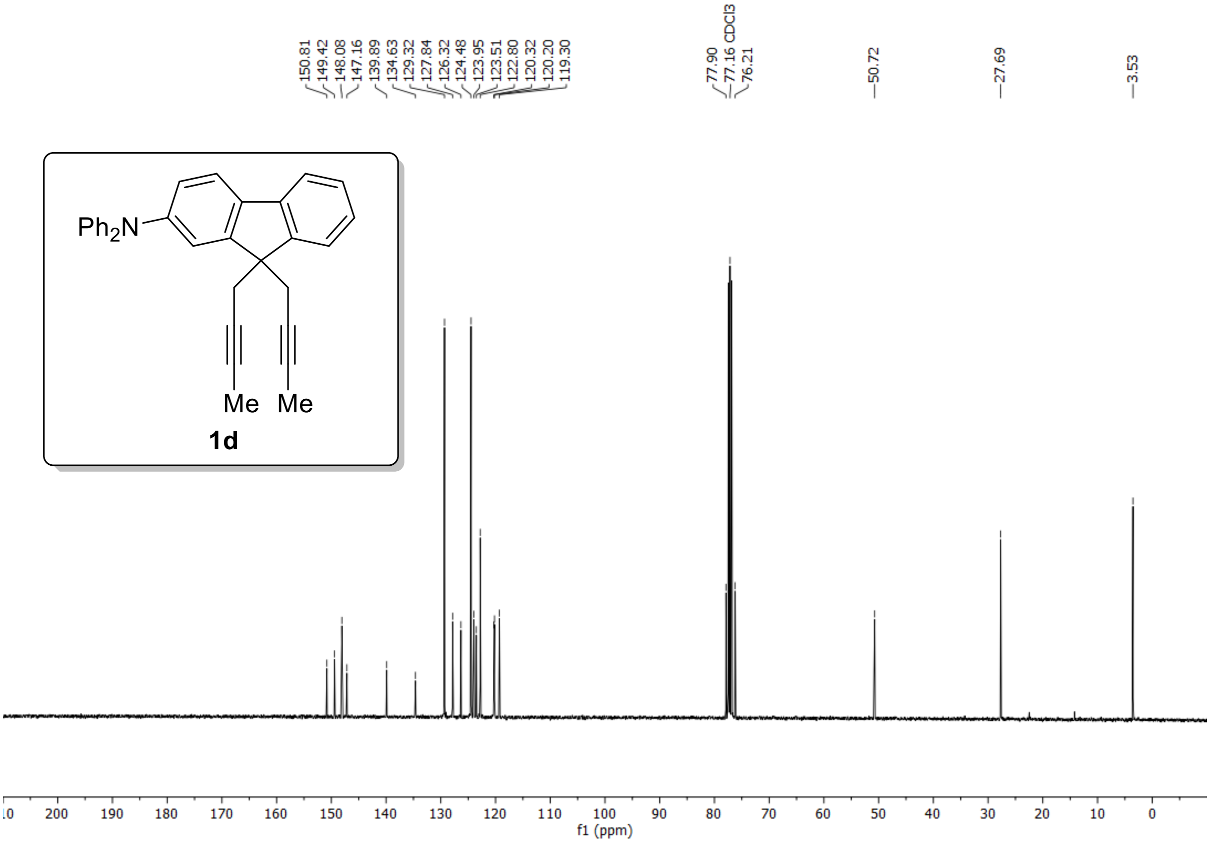
150.81
149.42
148.08
147.16
139.89
134.63
129.32
127.84
126.32
124.48
123.95
123.51
122.80
120.32
120.20
119.30
77.90
77.16 CDCl3
76.21
50.72
27.69
3.53
Ph2N
Me Me
1d
f1 (ppm)

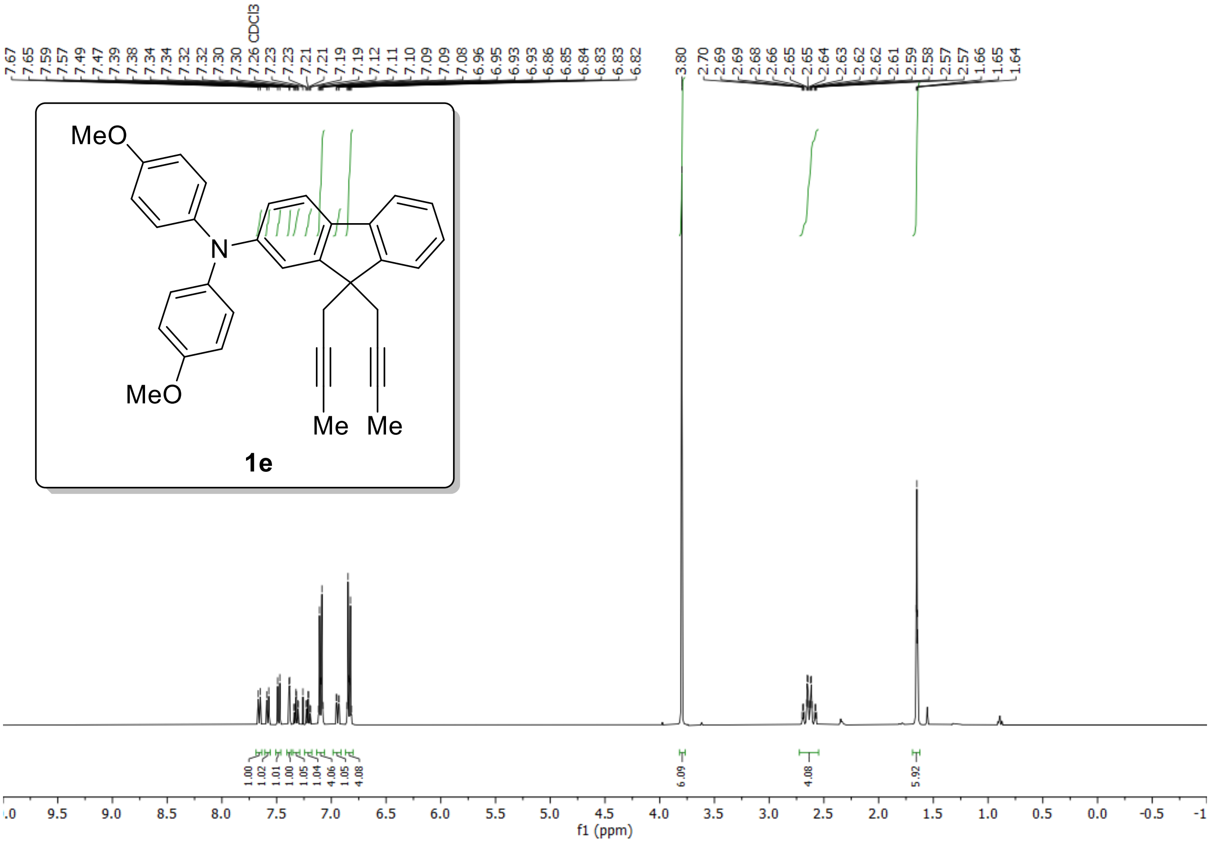
7.26 CDCl3
3.80
MeO
N
MeO
Me Me
1e
6.09
4.08
5.92
f1 (ppm)

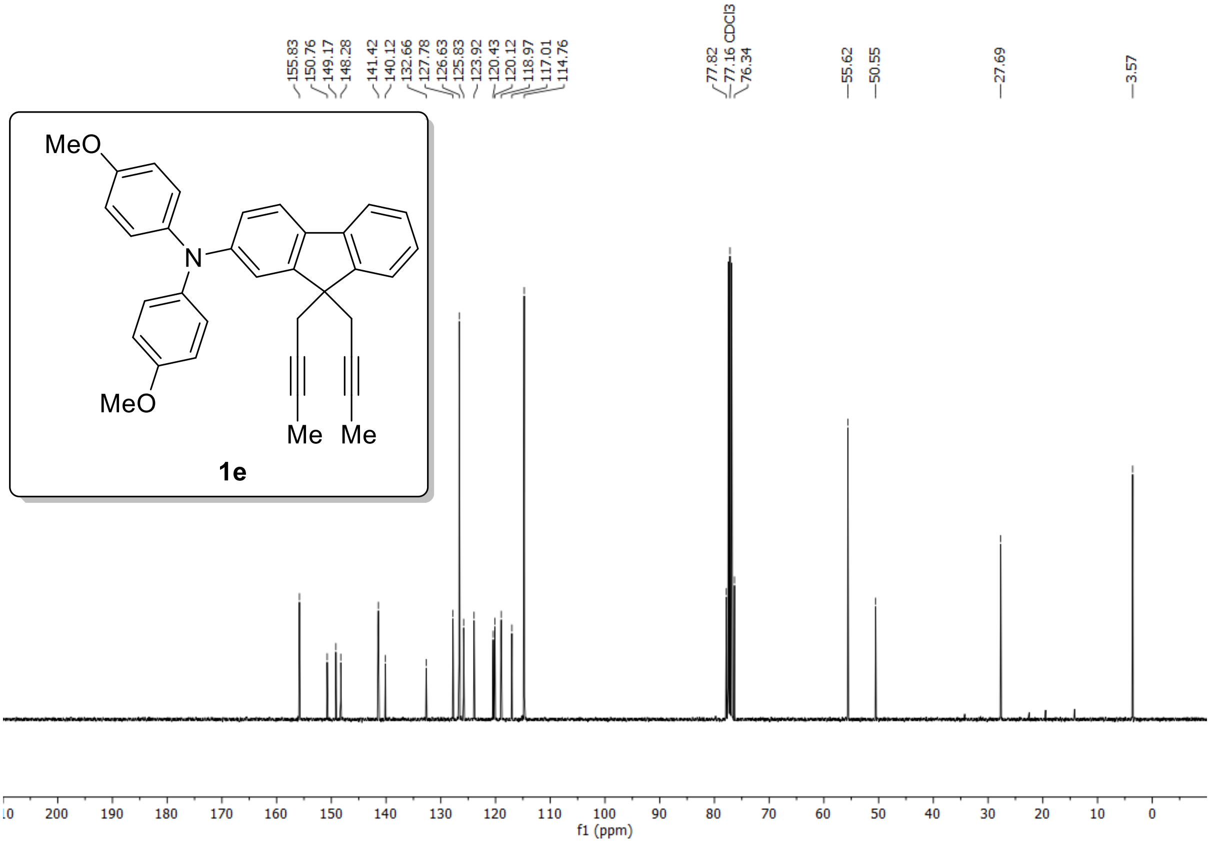
155.83
150.76
149.17
148.28
141.42
140.12
132.66
127.78
126.63
125.83
123.92
120.43
120.12
118.97
117.01
114.76
77.82
77.16 CDCl3
76.34
55.62
50.55
27.69
3.57
MeO
N
MeO
Me Me
1e
f1 (ppm)

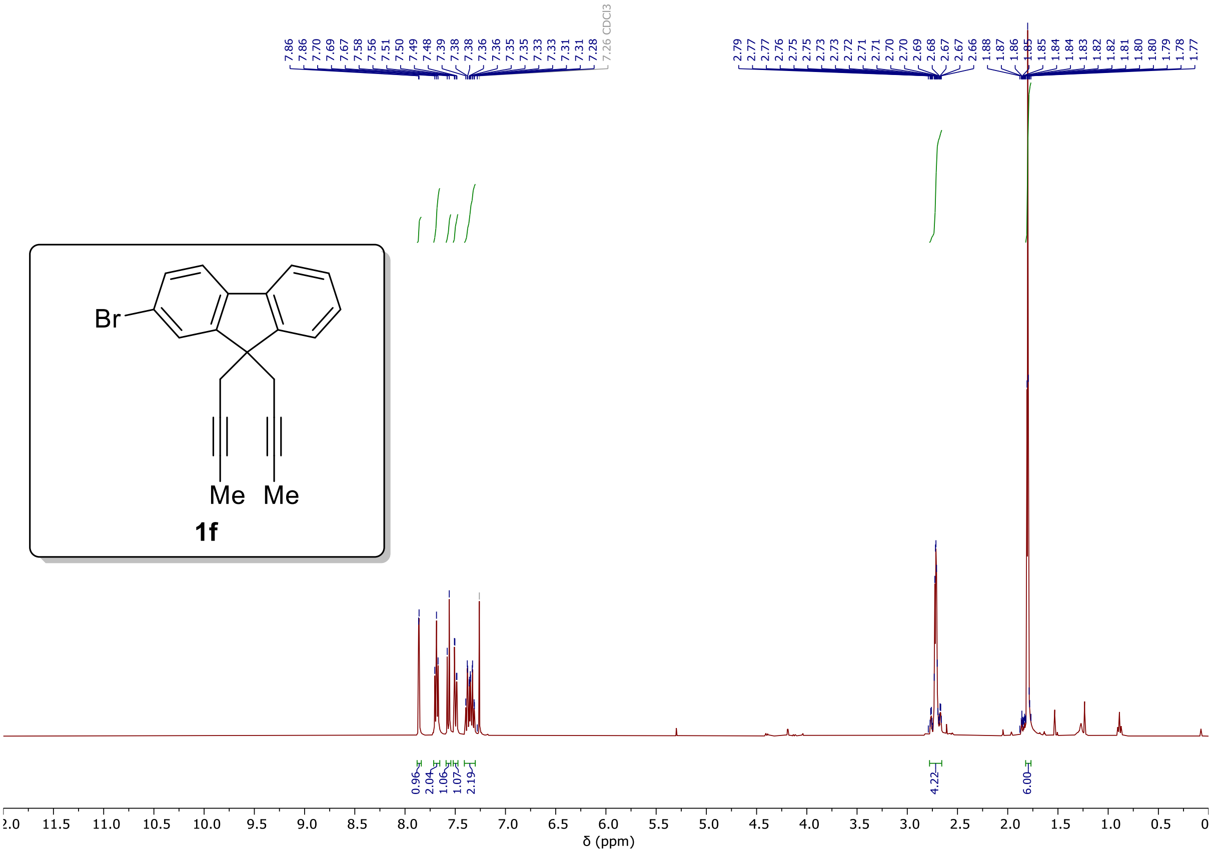
7.86
7.86
7.70
7.69
7.67
7.58
7.56
7.51
7.50
7.49
7.48
7.39
7.38
7.38
7.36
7.36
7.35
7.35
7.33
7.33
7.31
7.31
7.28
7.26 CDCl3
2.79
2.77
2.77
2.76
2.75
2.75
2.73
2.72
2.71
2.71
2.70
2.70
2.69
2.68
2.67
2.66
1.88
1.87
1.86
1.85
1.85
1.84
1.84
1.83
1.82
1.82
1.81
1.80
1.80
1.79
1.78
1.77
Br
Me Me
1f
0.96
2.04
1.06
1.07
2.19
4.22
6.00
δ (ppm)

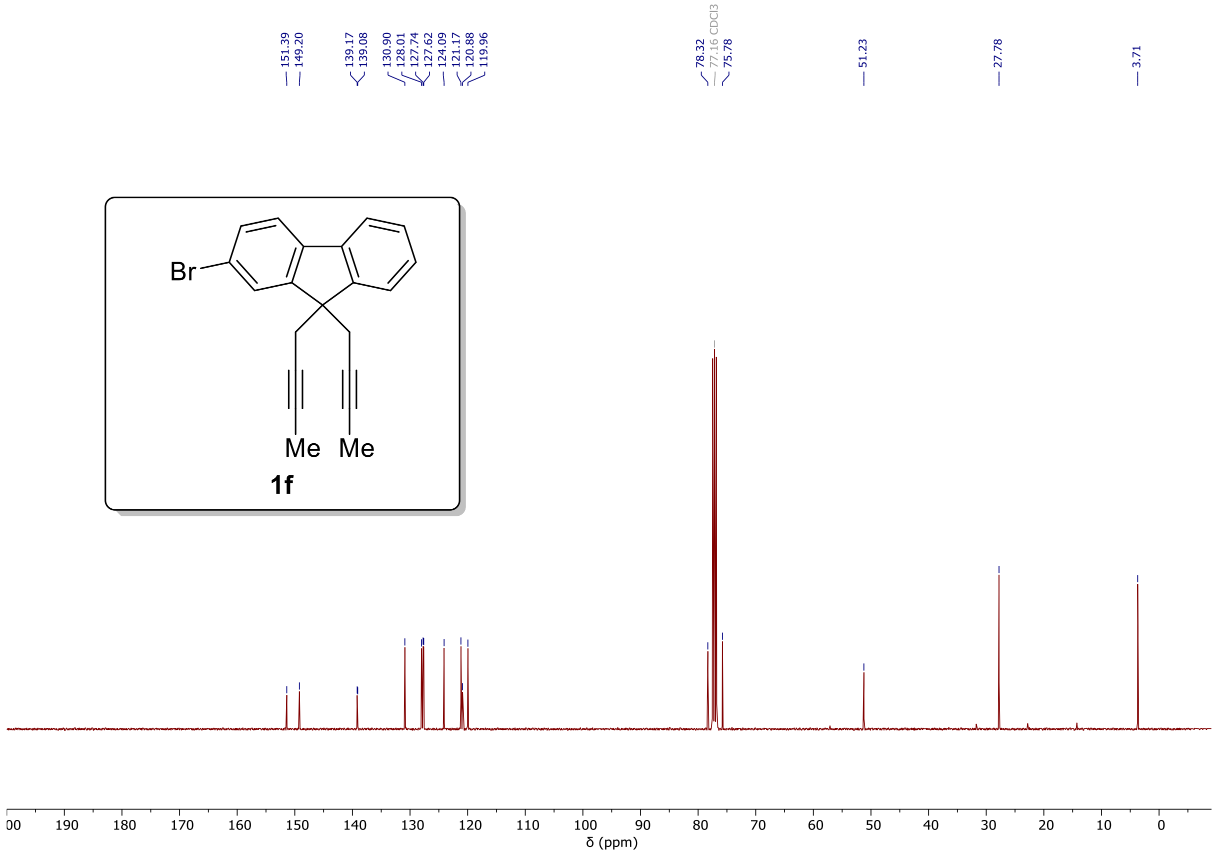
151.39
149.20
139.17
139.08
130.90
128.01
127.74
127.62
124.09
121.17
120.88
119.96
78.32
77.16 CDCl3
75.78
51.23
27.78
3.71
Br
Me Me
1f
δ (ppm)

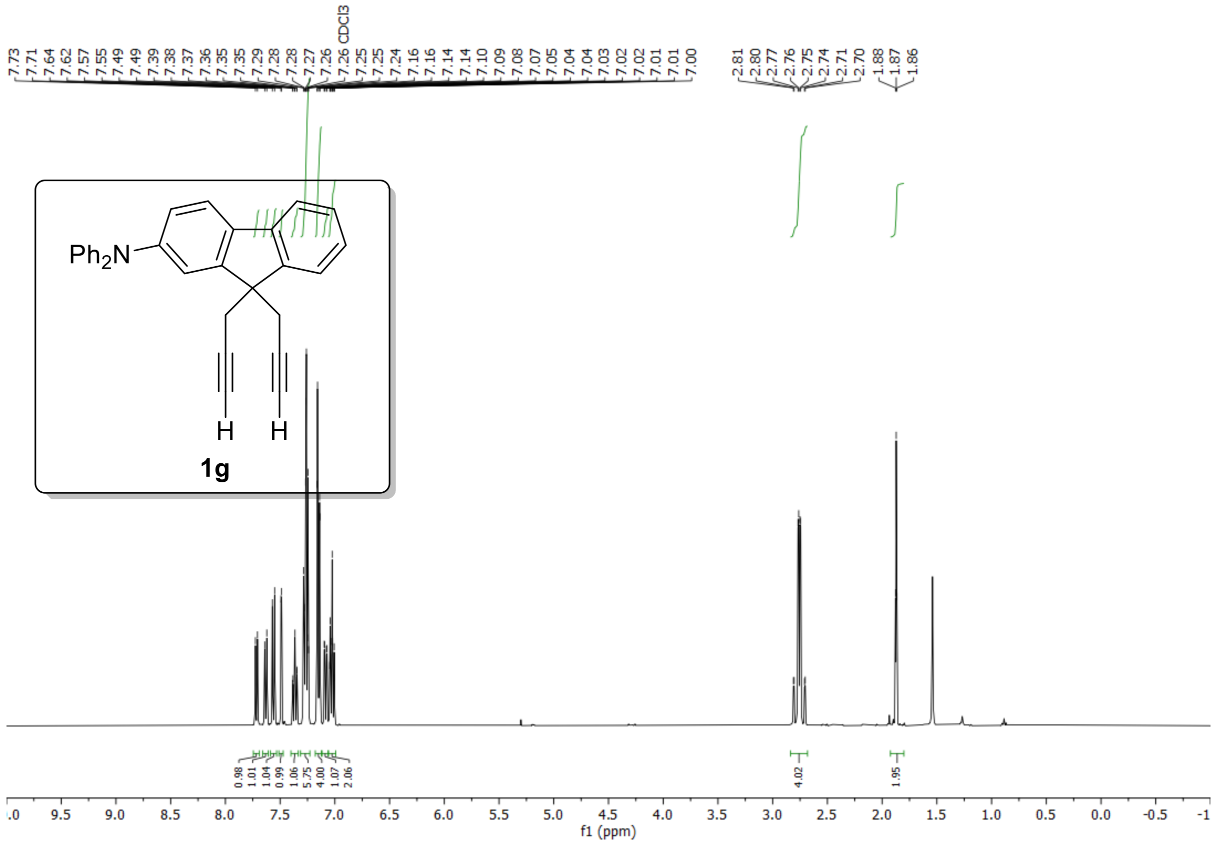
7.73
7.71
7.64
7.62
7.57
7.55
7.49
7.49
7.39
7.38
7.37
7.36
7.35
7.35
7.29
7.28
7.28
7.27
7.26
7.26 CDCl3
7.25
7.25
7.24
7.16
7.16
7.14
7.10
7.09
7.08
7.07
7.05
7.04
7.04
7.03
7.02
7.02
7.01
7.01
7.00
2.81
2.80
2.77
2.76
2.75
2.74
2.71
2.70
1.88
1.87
1.86
Ph2N
H H
1g
0.98
1.01
1.04
0.99
1.06
5.75
4.00
1.07
2.06
4.02
1.95
f1 (ppm)

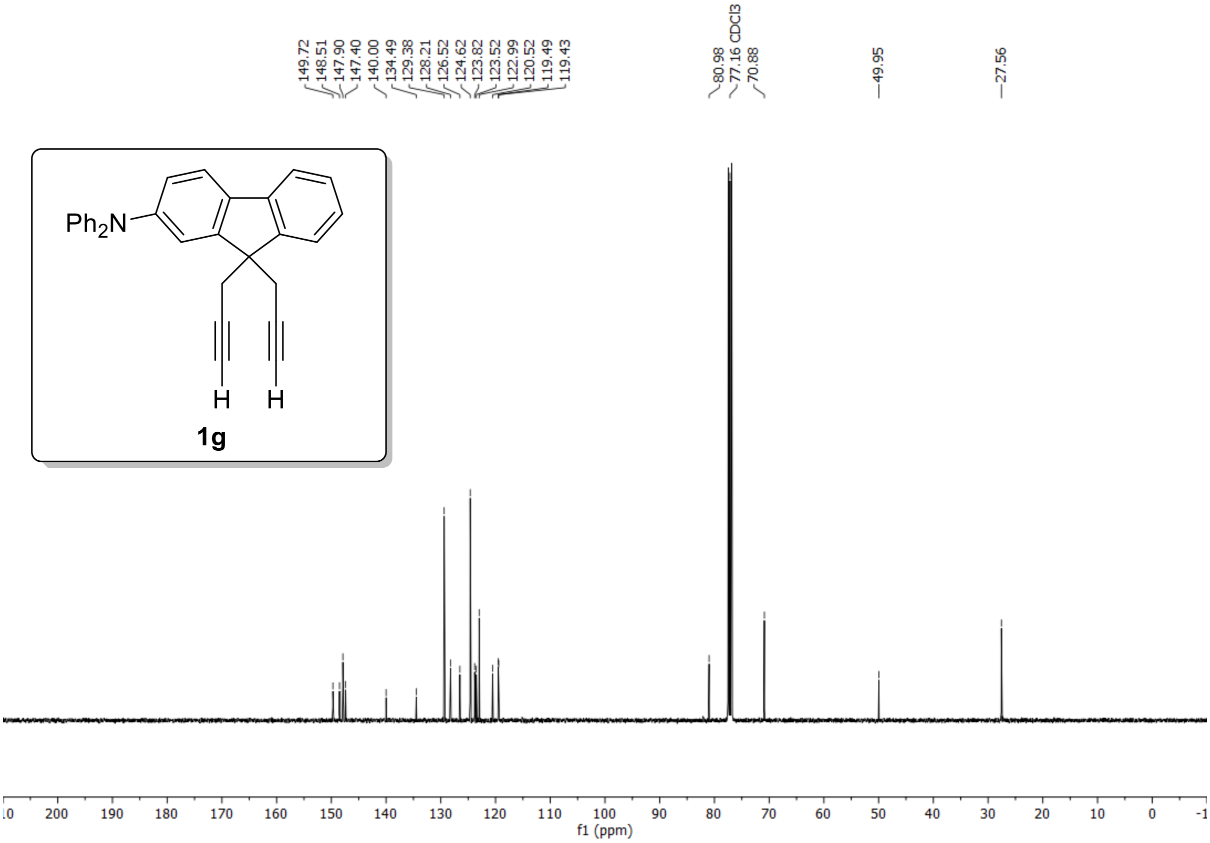
149.72
148.51
147.90
147.40
140.00
134.49
129.38
128.21
126.52
124.62
123.82
123.52
122.99
120.52
119.49
119.43
80.98
77.16 CDCl3
70.88
49.95
27.56
Ph2N
H H
f1 (ppm)

1g

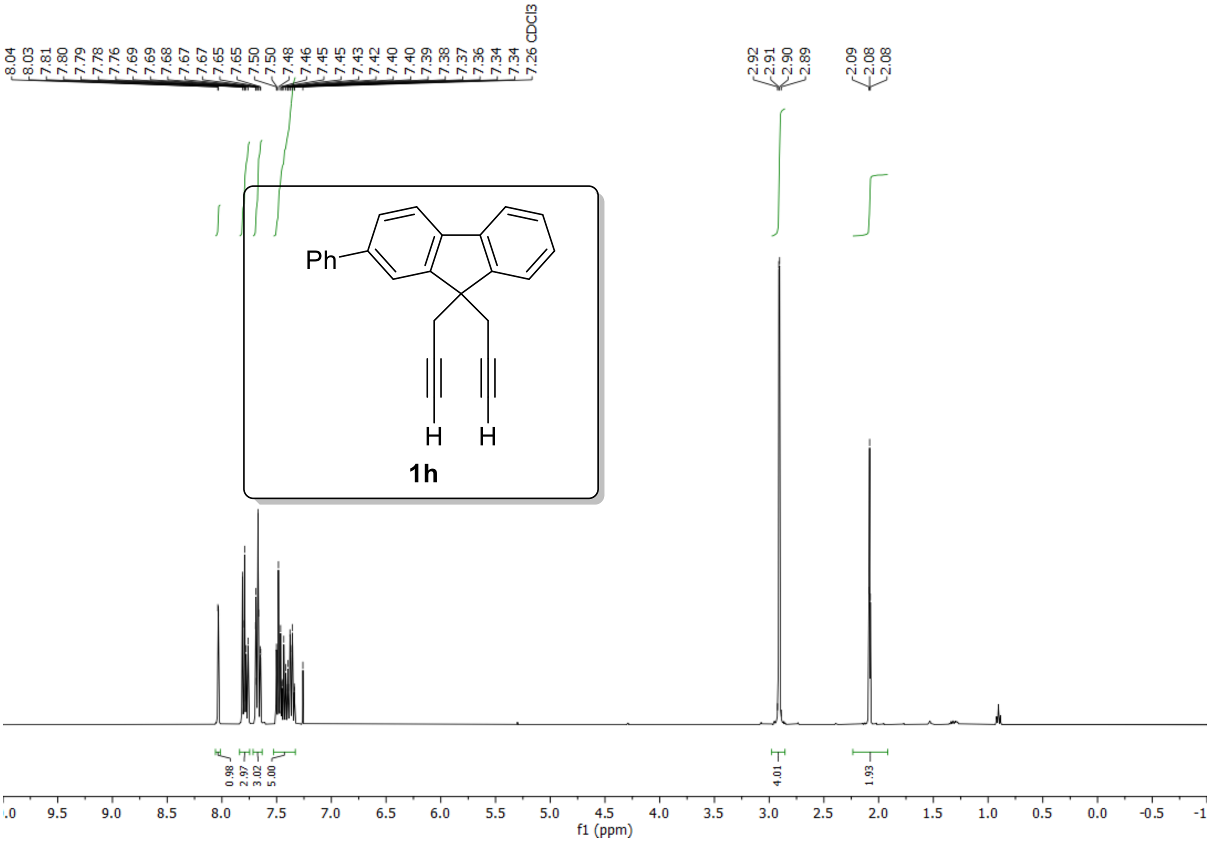
8.04
8.03
7.81
7.80
7.79
7.78
7.76
7.69
7.69
7.68
7.67
7.67
7.65
7.50
7.50
7.48
7.46
7.45
7.45
7.43
7.42
7.40
7.40
7.39
7.38
7.37
7.36
7.34
7.26 CDCl3
2.92
2.91
2.90
2.89
2.09
2.08
2.08
Ph
H H
0.98
2.97
3.02
5.00
4.01
1.93
f1 (ppm)

1h

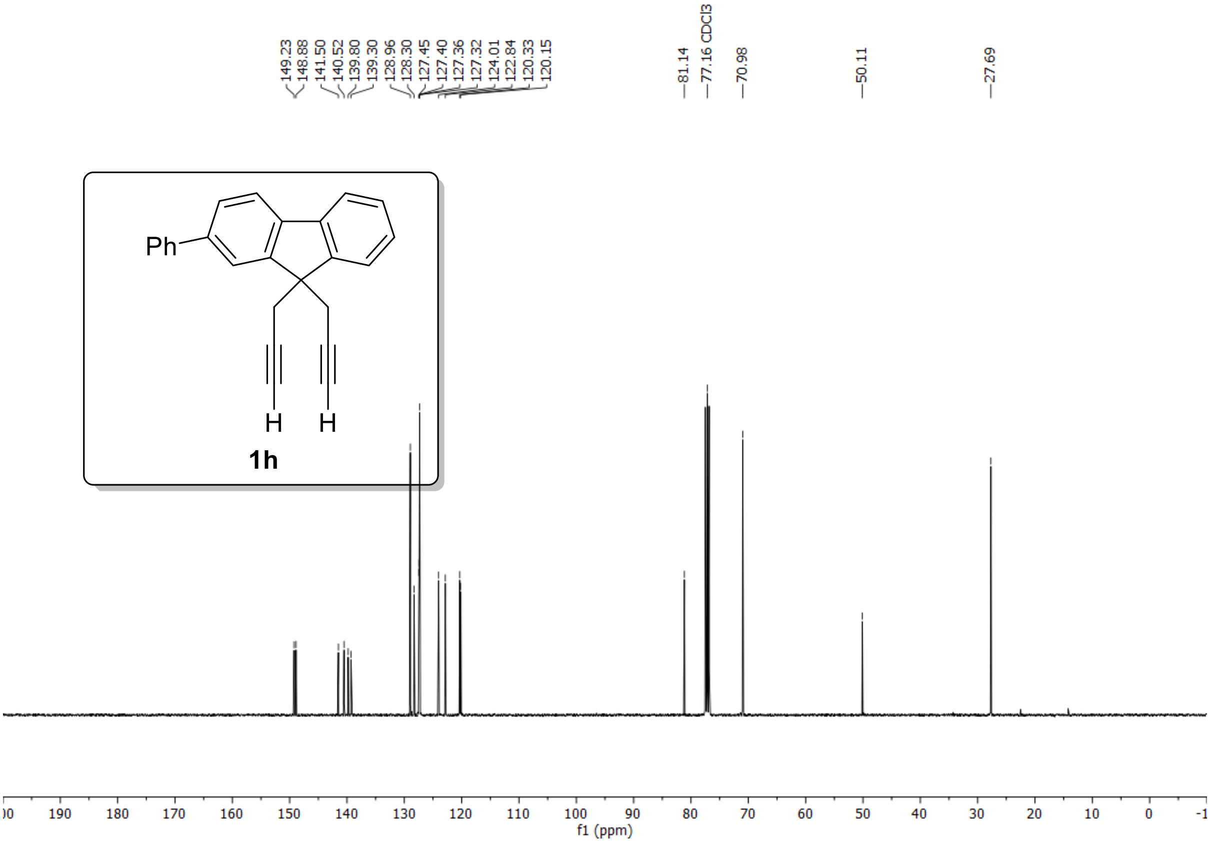
Ph
H
H
1h
f1 (ppm)

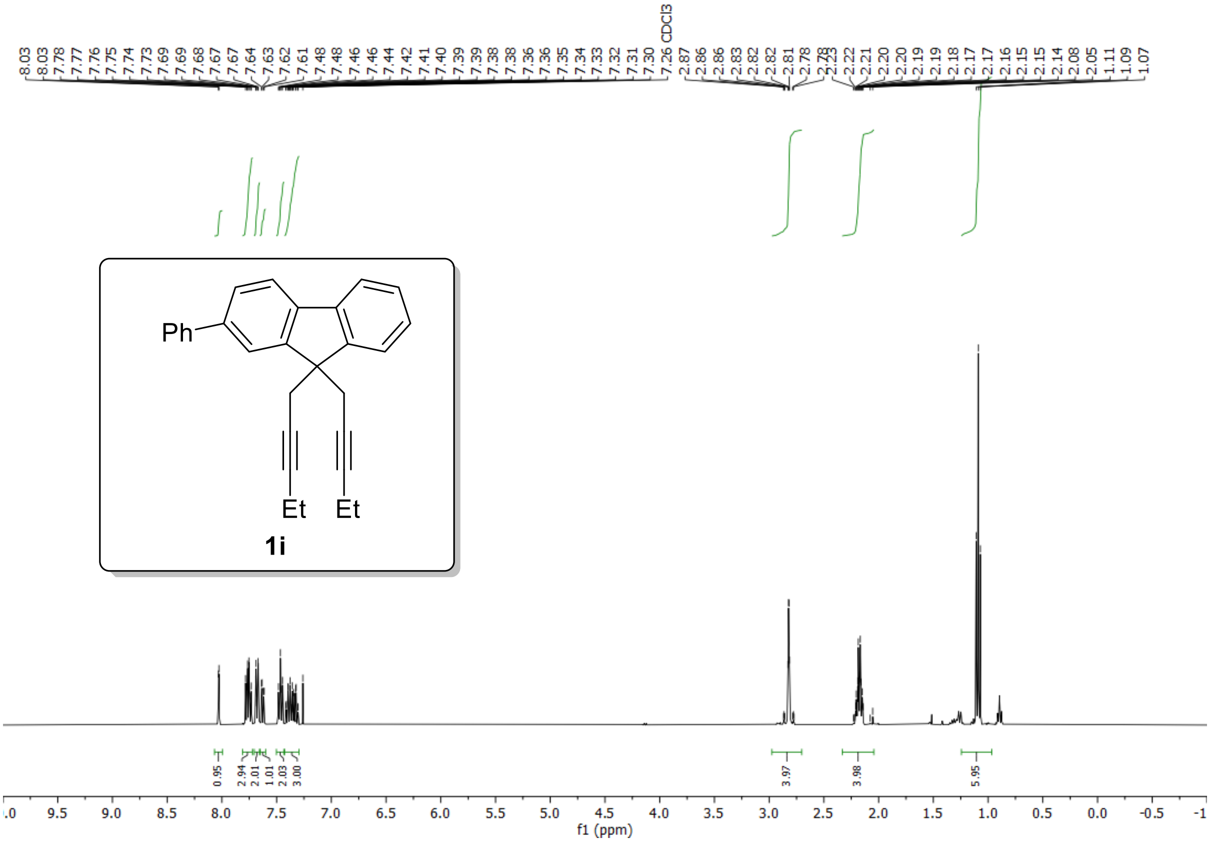
Ph
Et
Et
1i
f1 (ppm)

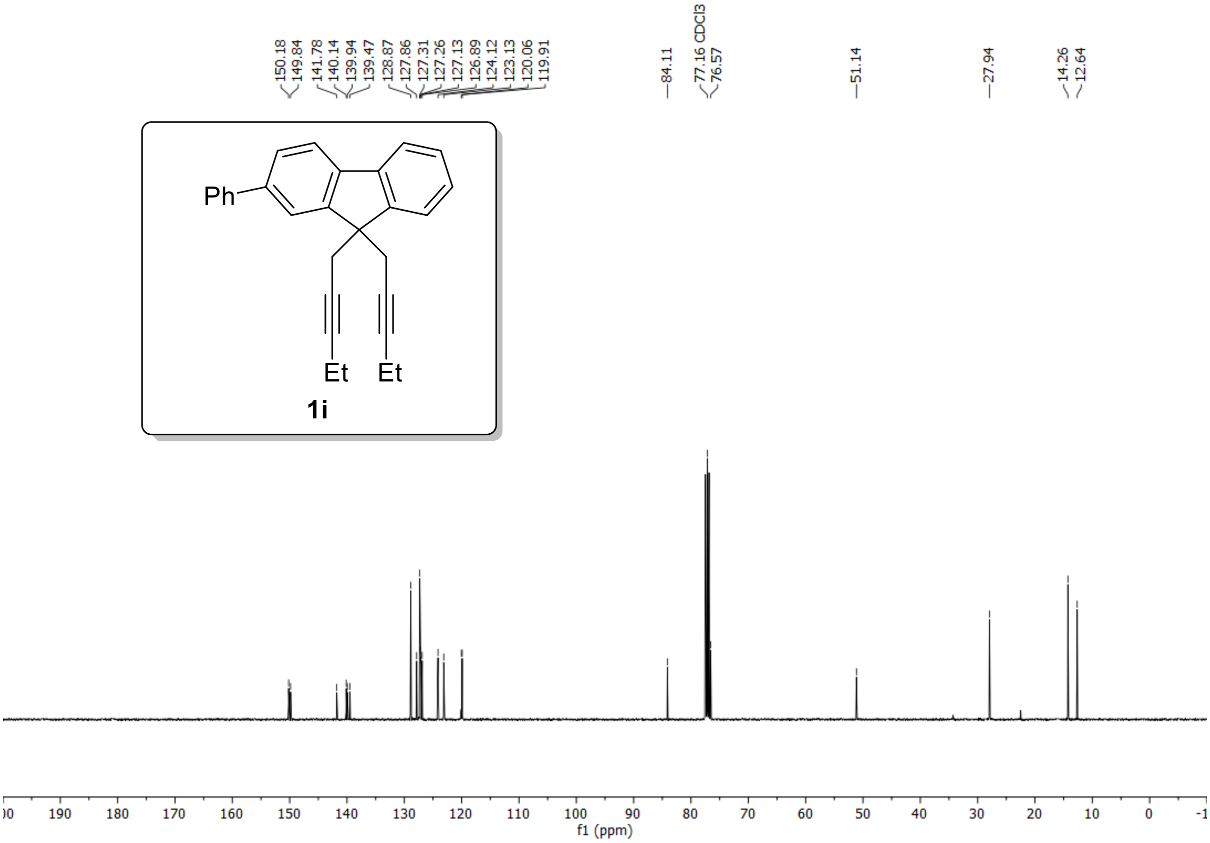
150.18
149.84
141.78
140.14
139.94
139.47
128.87
127.86
127.31
127.26
127.13
126.89
124.12
123.13
120.06
119.91
84.11
77.16 CDCl3
76.57
51.14
27.94
14.26
12.64
Ph
Et
Et
1i
f1 (ppm)

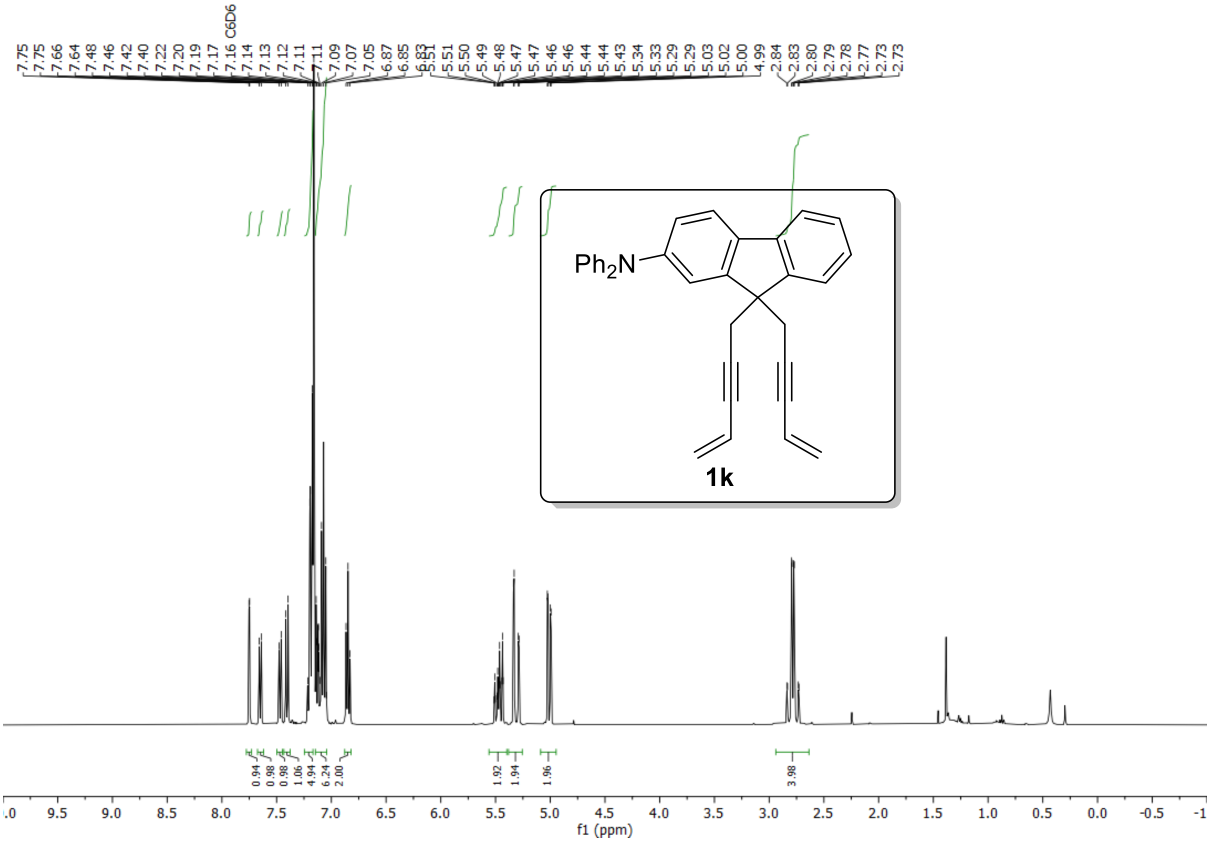
7.75
7.75
7.66
7.64
7.48
7.46
7.42
7.40
7.22
7.20
7.19
7.17
7.16 C6D6
7.14
7.13
7.12
7.11
7.11
7.09
7.07
7.05
6.87
6.85
6.83
5.51
5.50
5.49
5.48
5.47
5.47
5.46
5.46
5.44
5.44
5.43
5.34
5.33
5.29
5.29
5.03
5.02
5.00
4.99
2.84
2.83
2.80
2.79
2.78
2.77
2.73
2.73
Ph2N
1k
0.94
0.98
0.98
1.06
4.94
6.24
2.00
1.92
1.94
1.96
3.98
f1 (ppm)

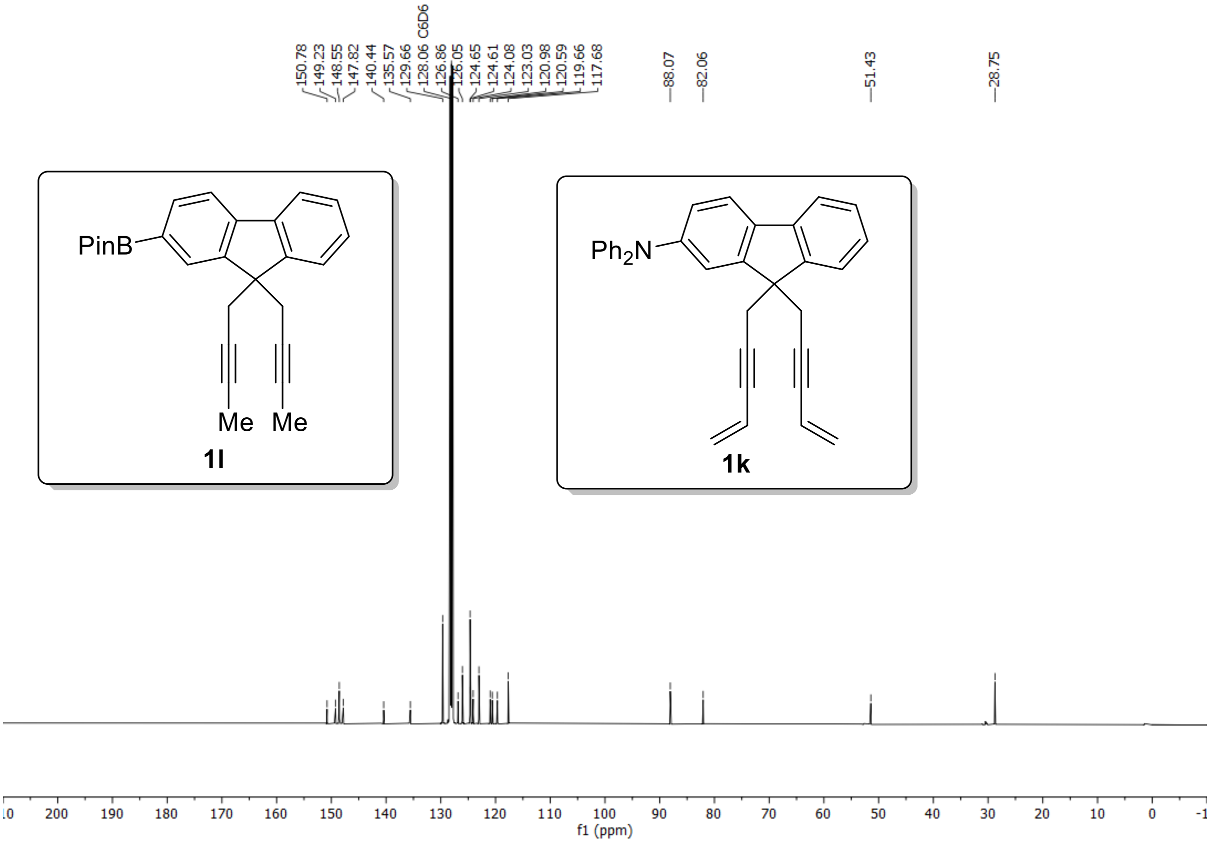
150.78
149.23
148.55
147.82
140.44
135.57
129.66
128.06 C6D6
126.86
126.05
124.65
124.61
124.08
123.03
120.98
120.59
119.66
117.68
88.07
82.06
51.43
28.75
PinB
Me Me
1l
Ph2N
1k
f1 (ppm)

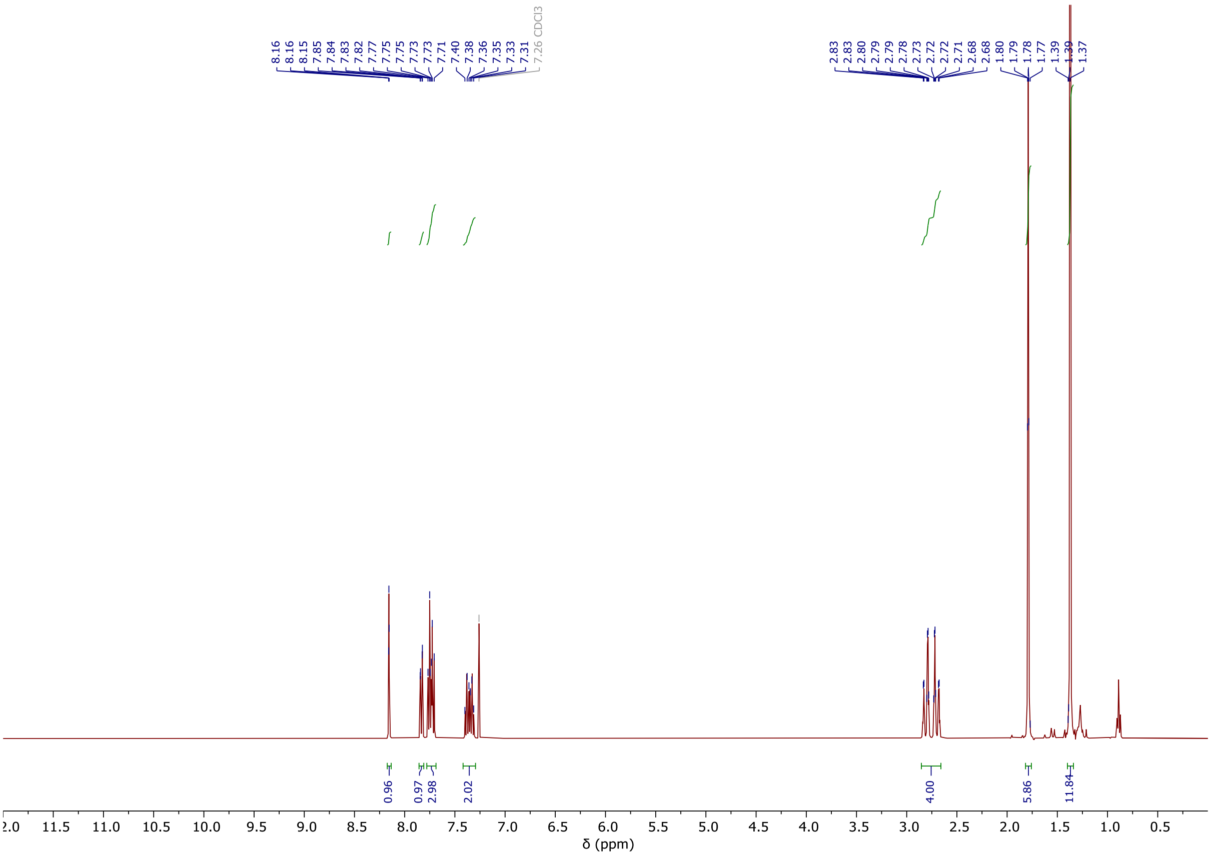
8.16
8.16
8.15
7.85
7.84
7.83
7.82
7.77
7.75
7.75
7.73
7.73
7.71
7.40
7.38
7.36
7.35
7.33
7.31
7.26 CDCl3
2.83
2.83
2.80
2.79
2.79
2.78
2.73
2.72
2.72
2.71
2.68
1.80
1.79
1.78
1.77
1.39
1.39
1.37
0.96
0.97
2.98
2.02
4.00
5.86
11.84
δ (ppm)

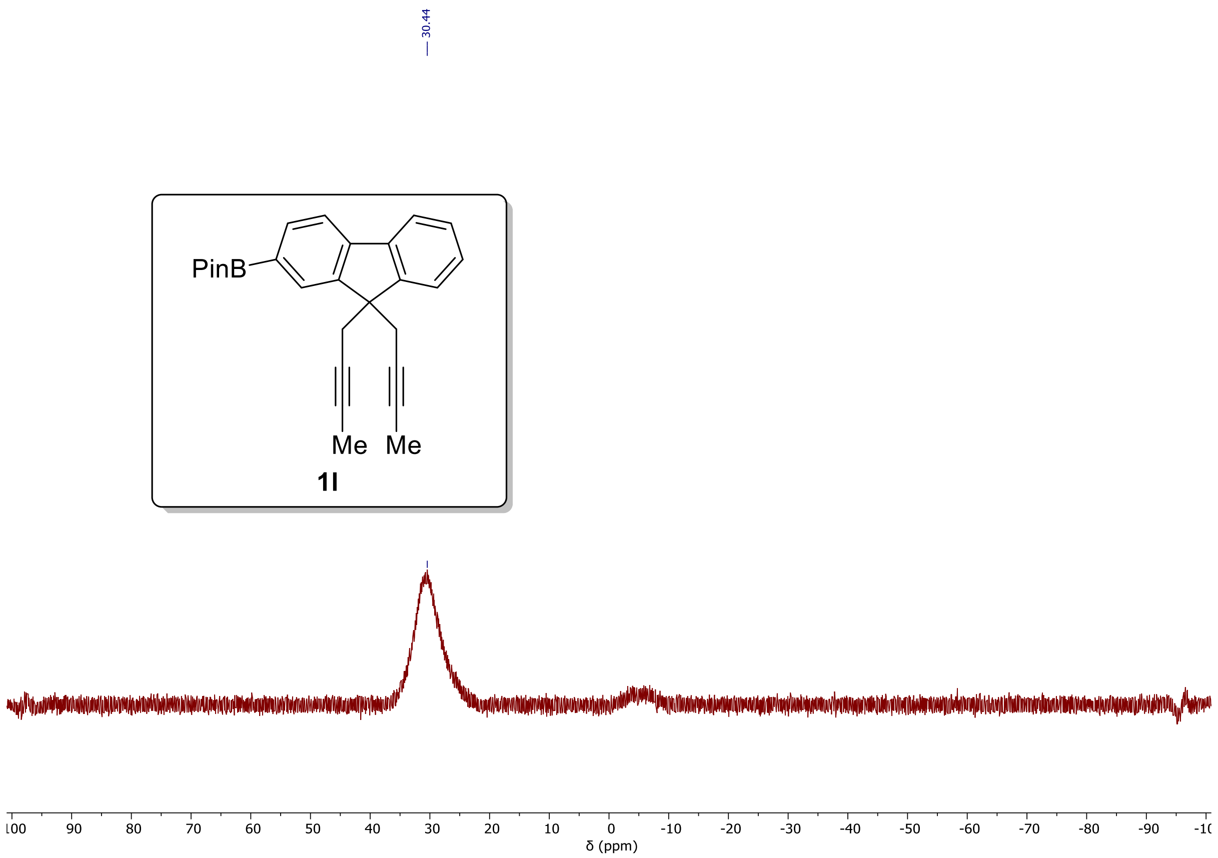
30.44
PinB
Me Me
1l
100
90
80
70
60
50
40
30
20
10
0
-10
-20
-30
-40
-50
-60
-70
-80
-90
δ (ppm)

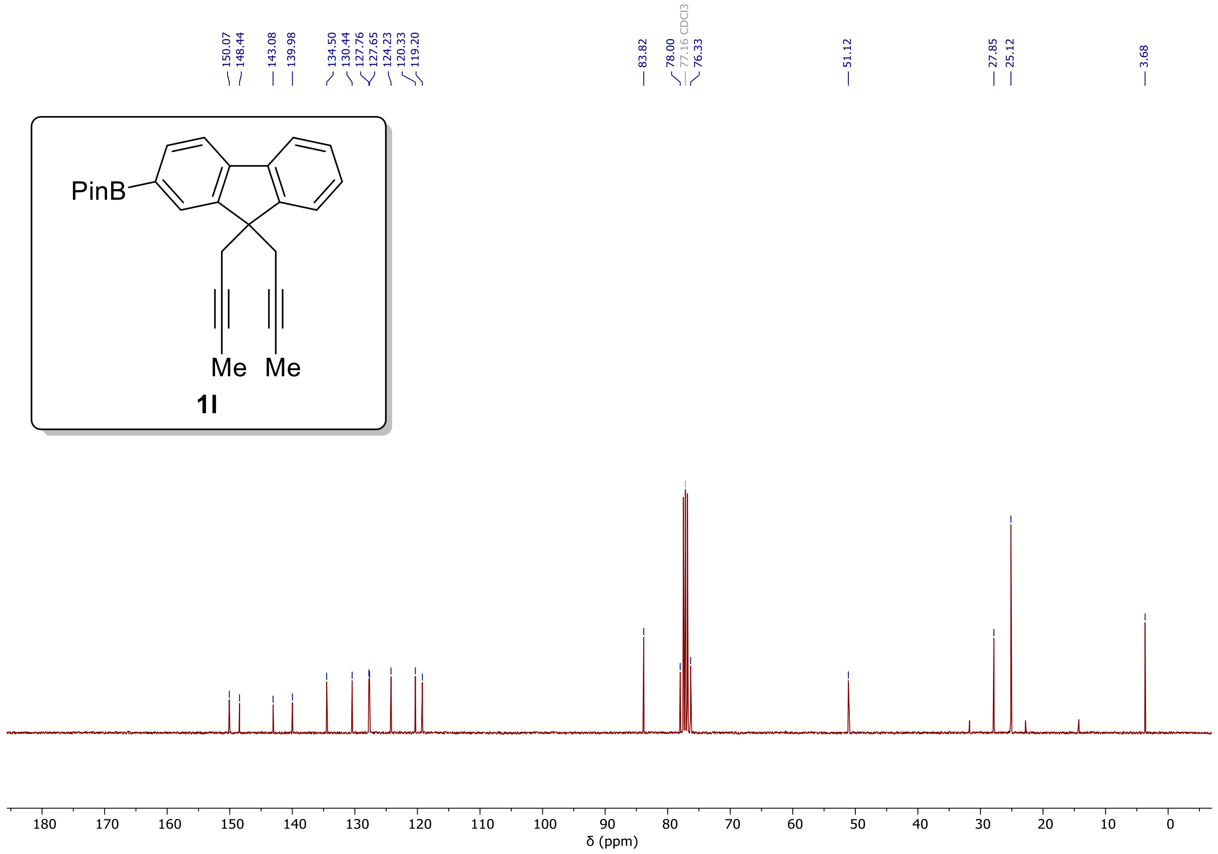
150.07
148.44
143.08
139.98
134.50
130.44
127.76
127.65
124.23
120.33
119.20
83.82
78.00
77.16 CDCl3
76.33
51.12
27.85
25.12
3.68
PinB
Me Me
1l
180
170
160
150
140
130
120
110
100
90
80
70
60
50
40
30
20
10
0
δ (ppm)

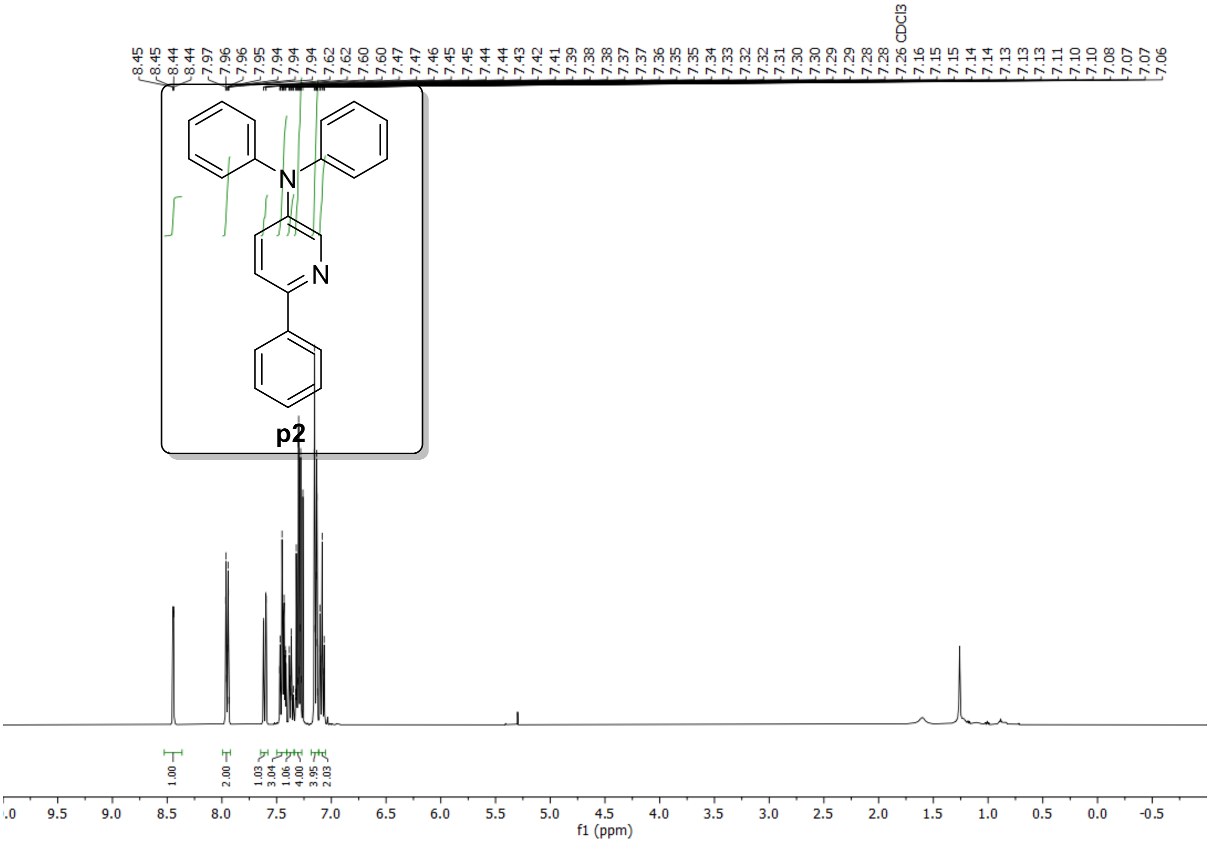
8.45 8.45 8.44 8.44 7.97 7.96 7.96 7.95 7.94 7.94 7.94 7.94 7.62 7.62 7.60 7.47 7.47 7.46 7.45 7.45 7.44 7.44 7.43 7.42 7.41 7.39 7.38 7.38 7.37 7.37 7.36 7.35 7.35 7.34 7.33 7.32 7.32 7.31 7.30 7.30 7.29 7.29 7.28 7.28 7.26 CDCl3 7.16 7.15 7.15 7.14 7.14 7.13 7.13 7.13 7.11 7.10 7.10 7.08 7.07 7.07 7.06
N
N
p2
1.00
2.00
1.03
3.04
1.06
4.00
3.95
2.03
f1 (ppm)

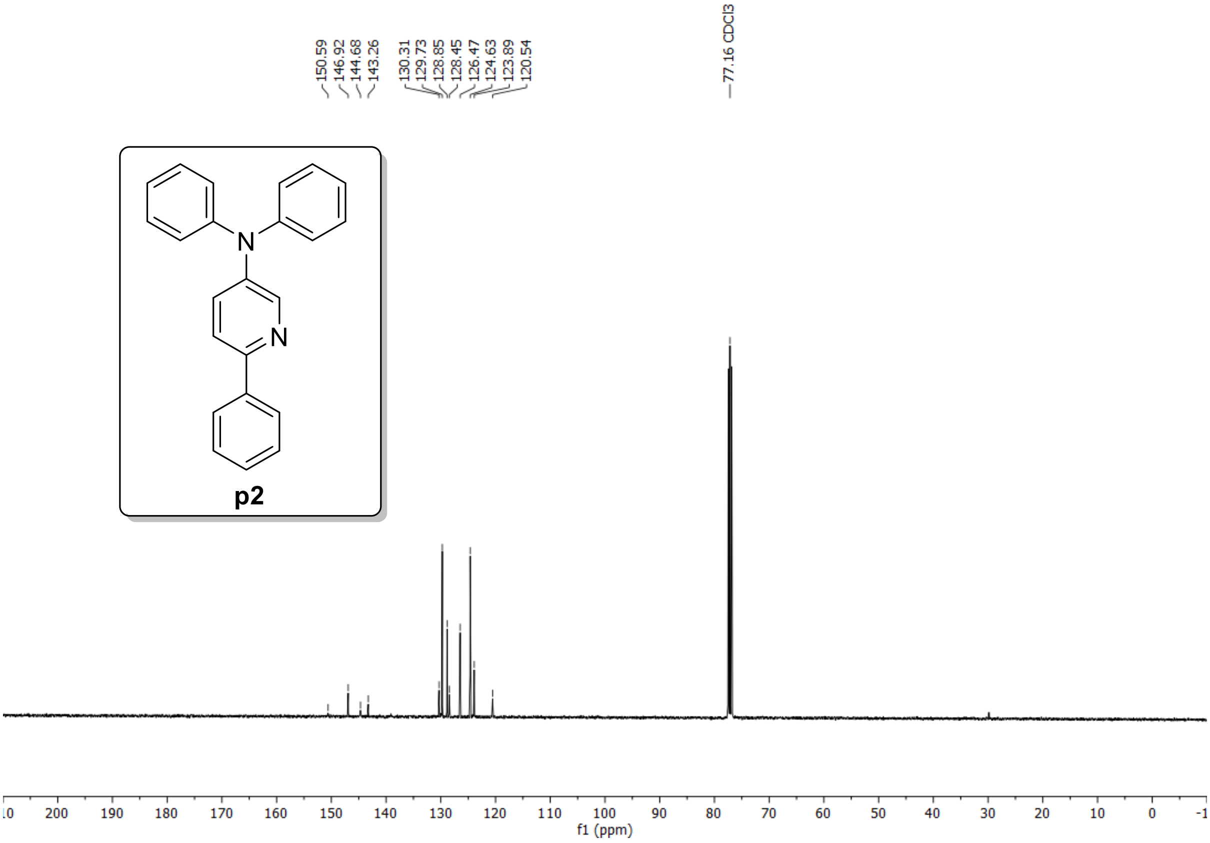
150.59
146.92
144.68
143.26
130.31
129.73
128.85
128.45
126.47
124.63
123.89
120.54
77.16 CDCl3
N
N
p2
f1 (ppm)

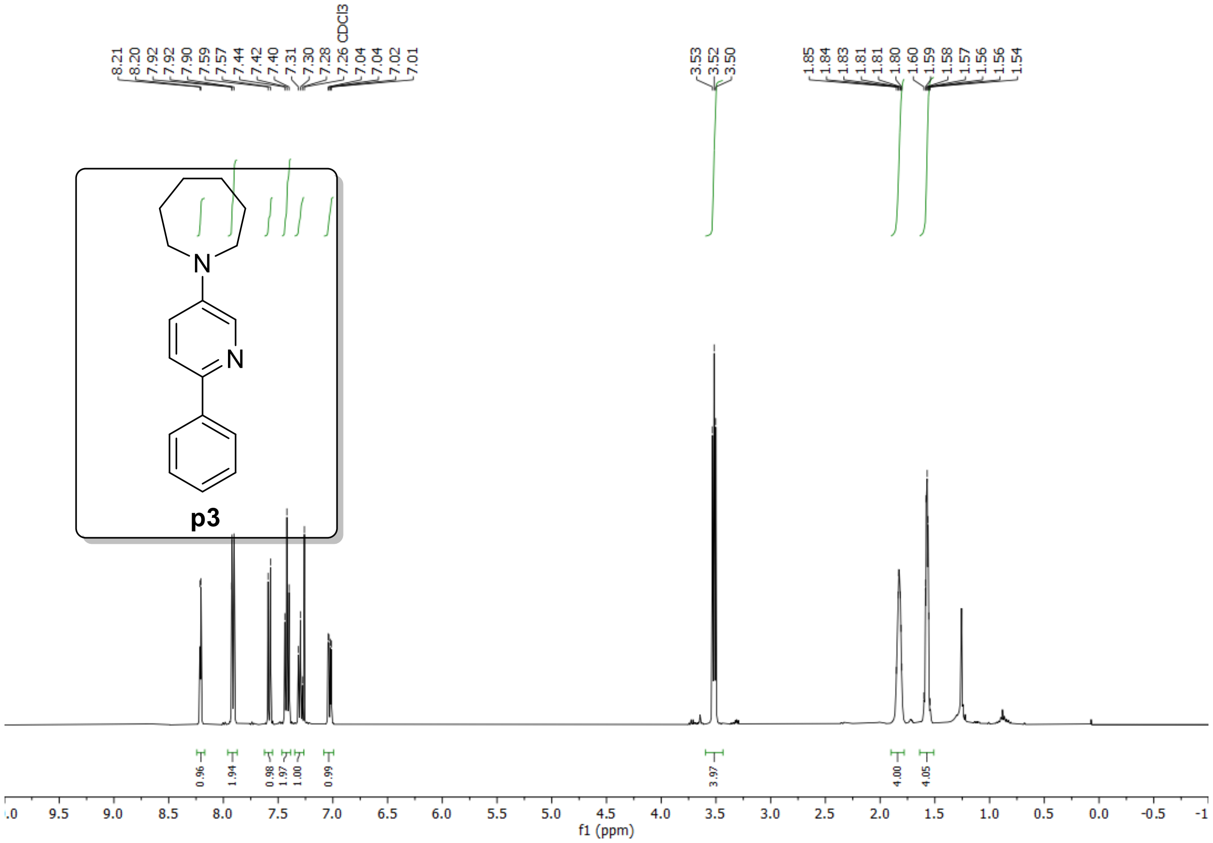
8.21 8.20 7.92 7.92 7.90 7.59 7.57 7.44 7.42 7.40 7.31 7.30 7.28 7.26 CDCl3 7.04 7.04 7.02 7.01
3.53 3.52 3.50
1.85 1.84 1.83 1.81 1.81 1.80 1.60 1.59 1.58 1.57 1.56 1.56 1.54
p3
0.96 1.94 0.98 1.97 1.00 0.99 3.97 4.00 4.05
f1 (ppm)

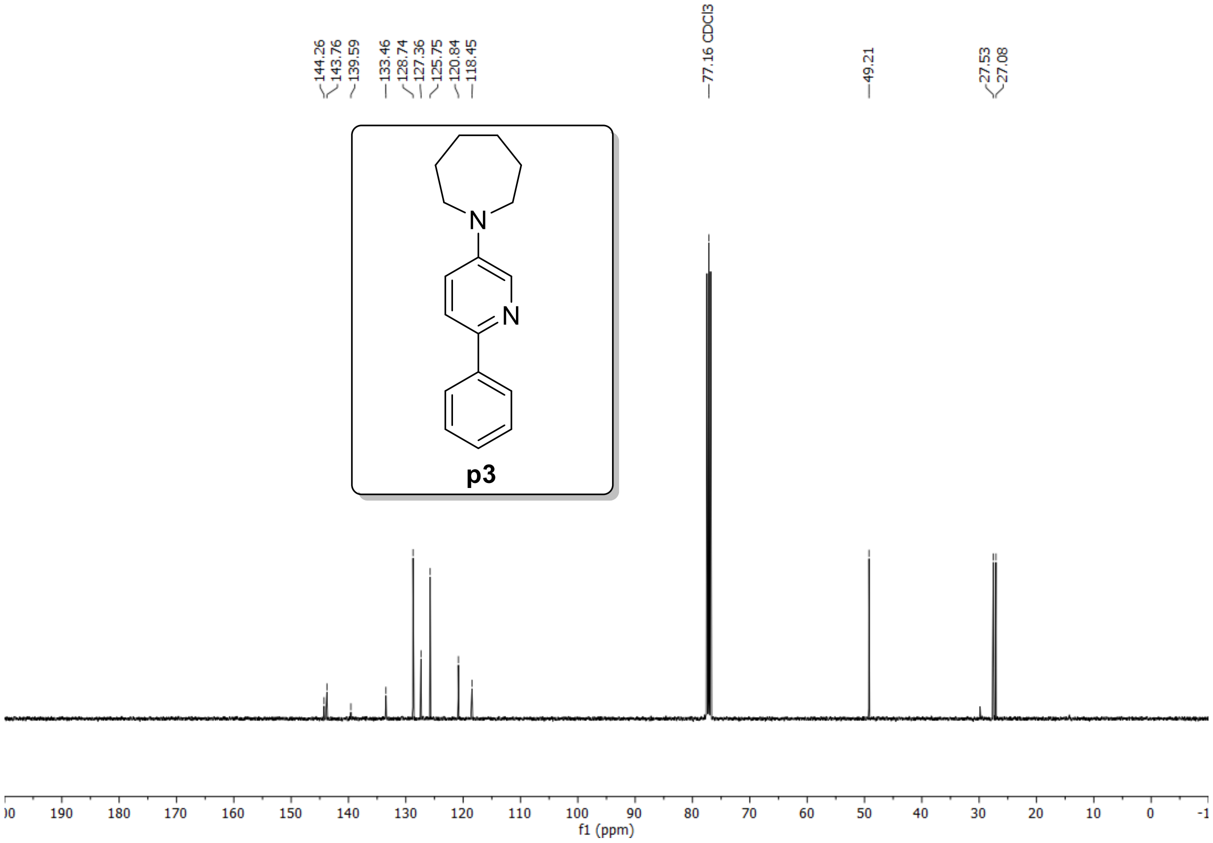
144.26 143.76 139.59 133.46 128.74 127.36 125.75 120.84 118.45
77.16 CDCl3
49.21
27.53 27.08
p3
f1 (ppm)

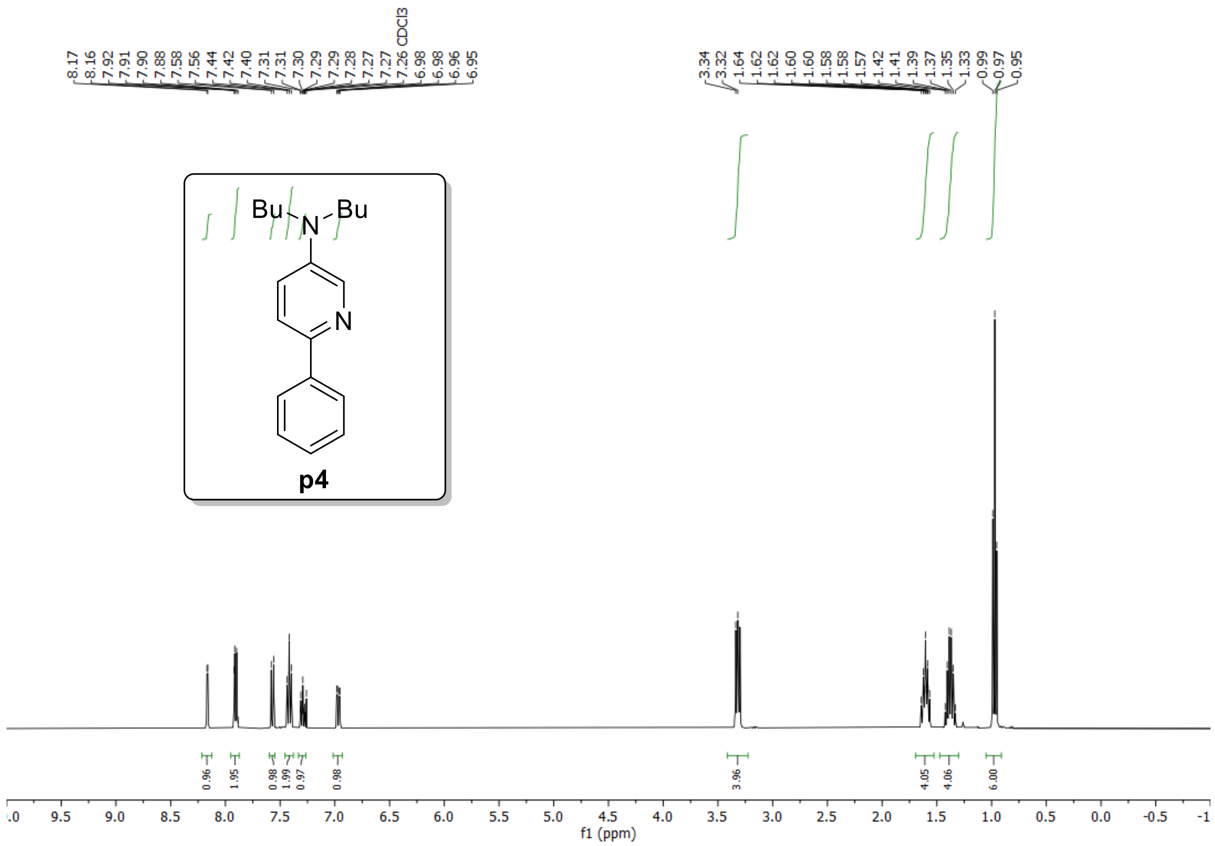

8.17
8.16
7.92
7.91
7.90
7.88
7.58
7.56
7.44
7.42
7.40
7.31
7.31
7.30
7.29
7.29
7.28
7.27
7.27
7.26 CDCl3
6.98
6.98
6.96
6.95
3.34
3.32
1.64
1.62
1.62
1.60
1.60
1.58
1.58
1.57
1.42
1.41
1.39
1.37
1.35
1.33
0.99
0.97
0.95
Bu
N
Bu
N
p4
0.96
1.95
0.98
1.99
0.97
0.98
3.96
4.05
4.06
6.00
f1 (ppm)


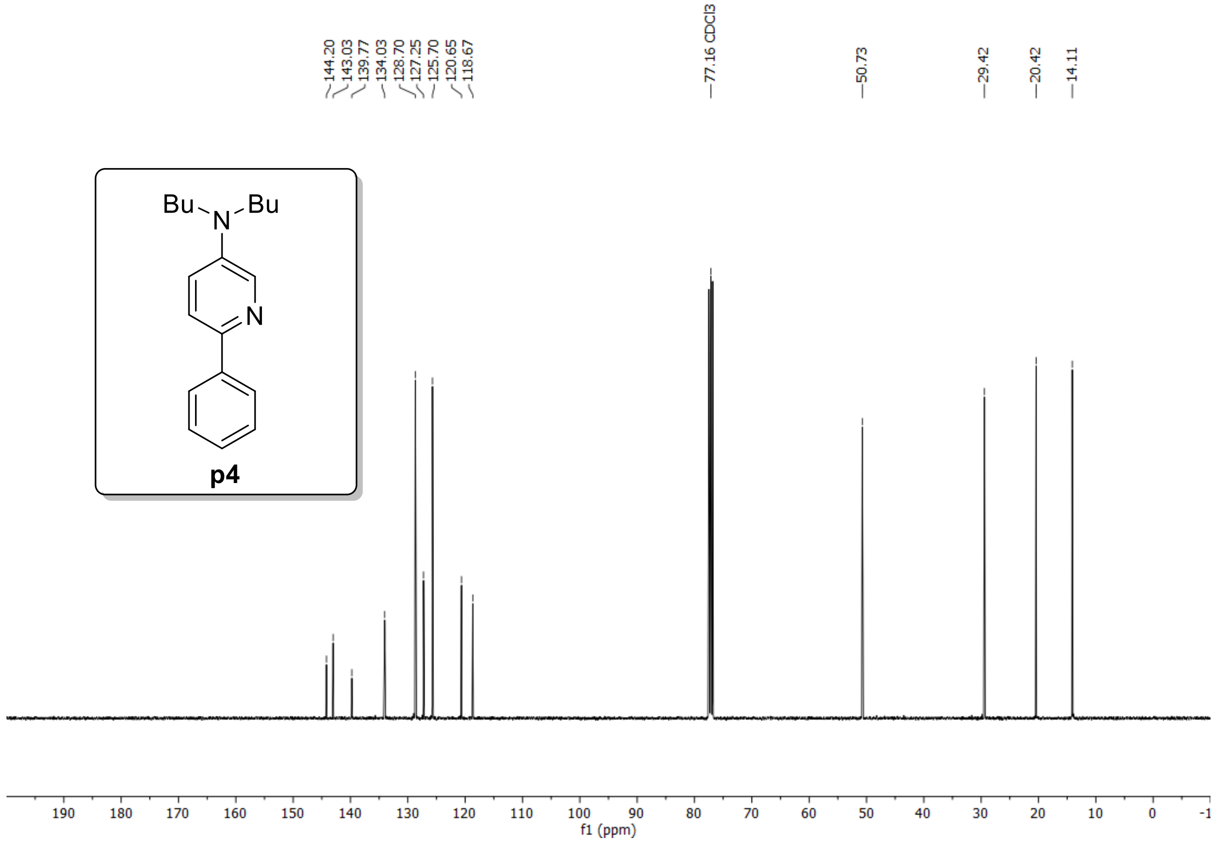

144.20
143.03
139.77
134.03
128.70
127.25
125.70
120.65
118.67
77.16 CDCl3
50.73
29.42
20.42
14.11
Bu
N
Bu
N
p4
f1 (ppm)

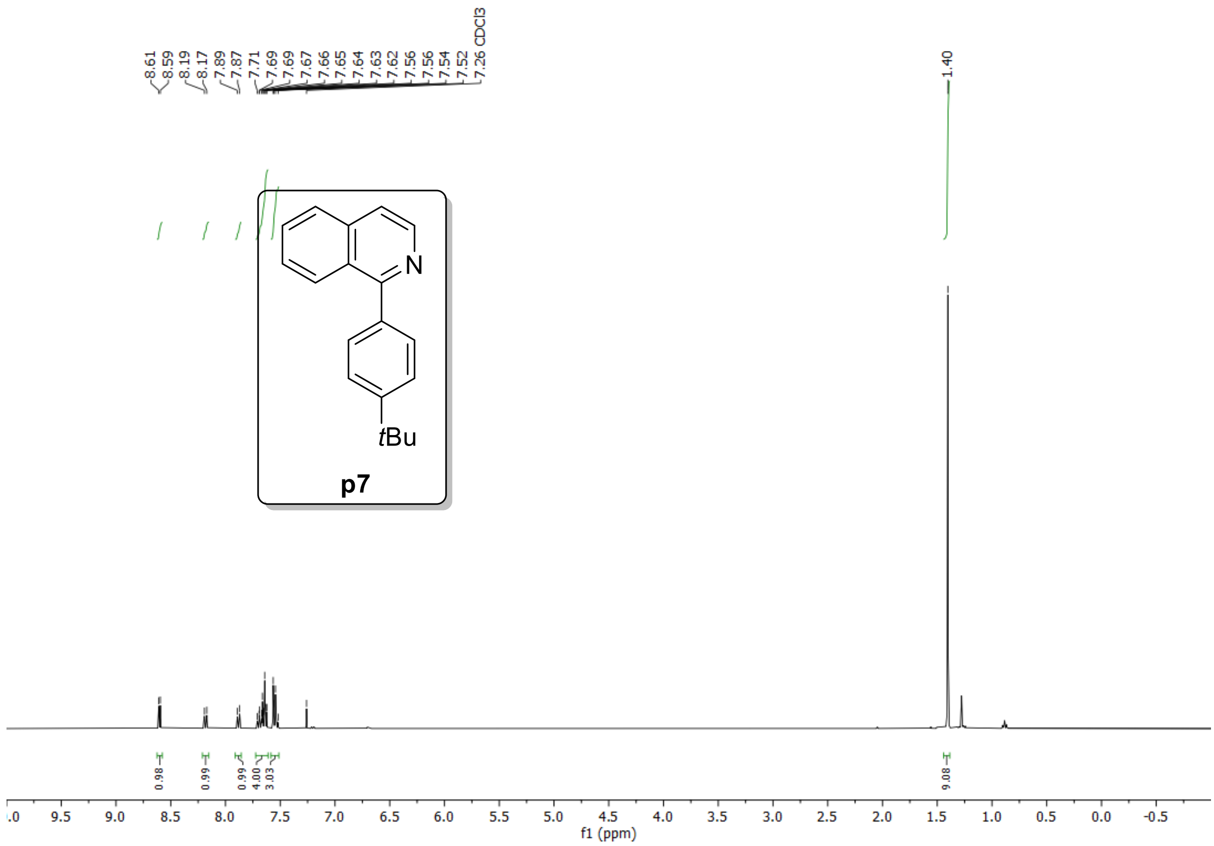
8.61
8.59
8.19
8.17
7.89
7.87
7.71
7.69
7.69
7.67
7.66
7.65
7.64
7.63
7.62
7.56
7.56
7.54
7.52
7.26 CDCl3
1.40
N
tBu
p7
0.98
0.99
0.99
4.00
3.03
9.08
f1 (ppm)

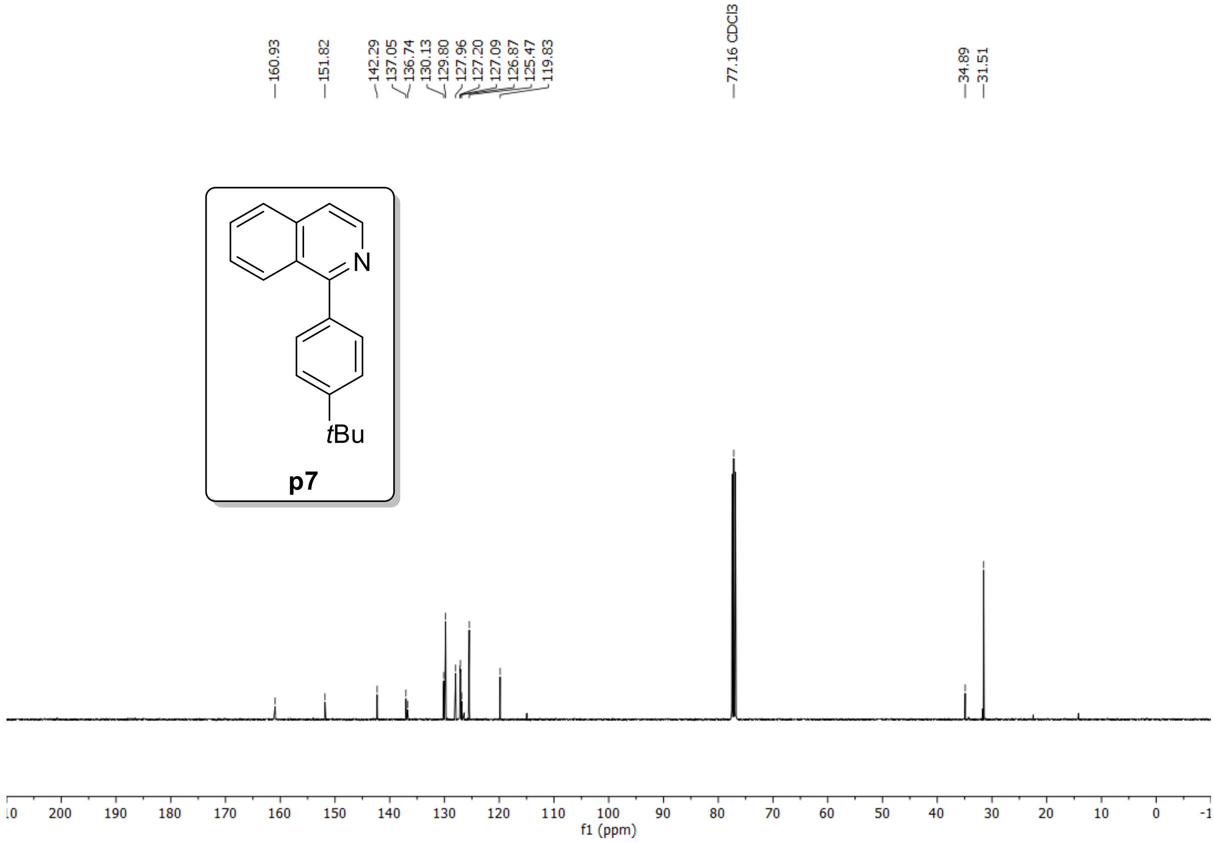
160.93
151.82
142.29
137.05
136.74
130.13
129.80
127.96
127.20
127.09
126.87
125.47
119.83
77.16 CDCl3
34.89
31.51
N
tBu
p7
f1 (ppm)

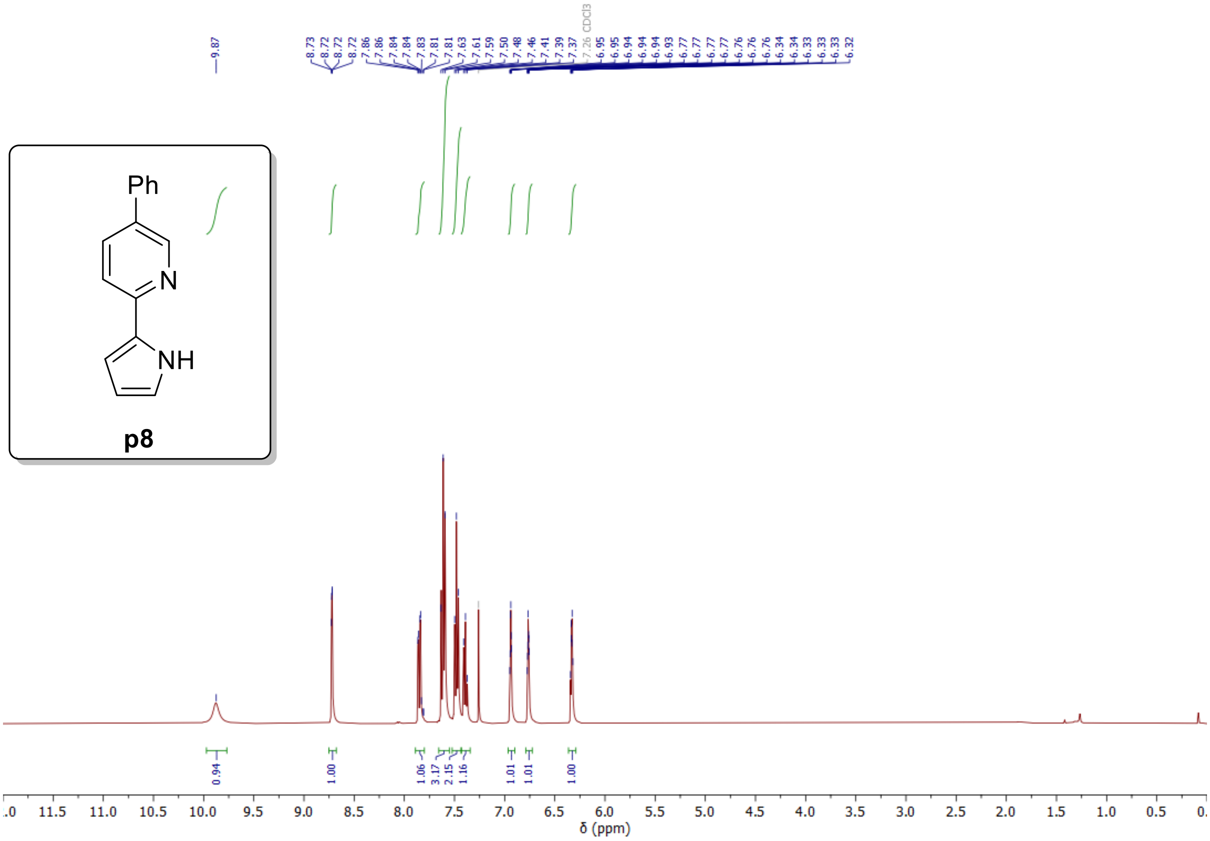
9.87
8.73 8.72 8.72 8.72 7.86 7.84 7.84 7.83 7.81 7.81 7.63 7.61 7.59 7.50 7.48 7.46 7.41 7.39 7.37
7.26 CDCl3
6.95 6.95 6.94 6.94 6.94 6.93 6.77 6.77 6.77 6.77 6.76 6.76 6.34 6.33 6.33 6.33 6.32
Ph
N
NH
p8
0.94 1.00 1.06 3.17 2.15 1.16 1.01 1.01 1.00
δ (ppm)

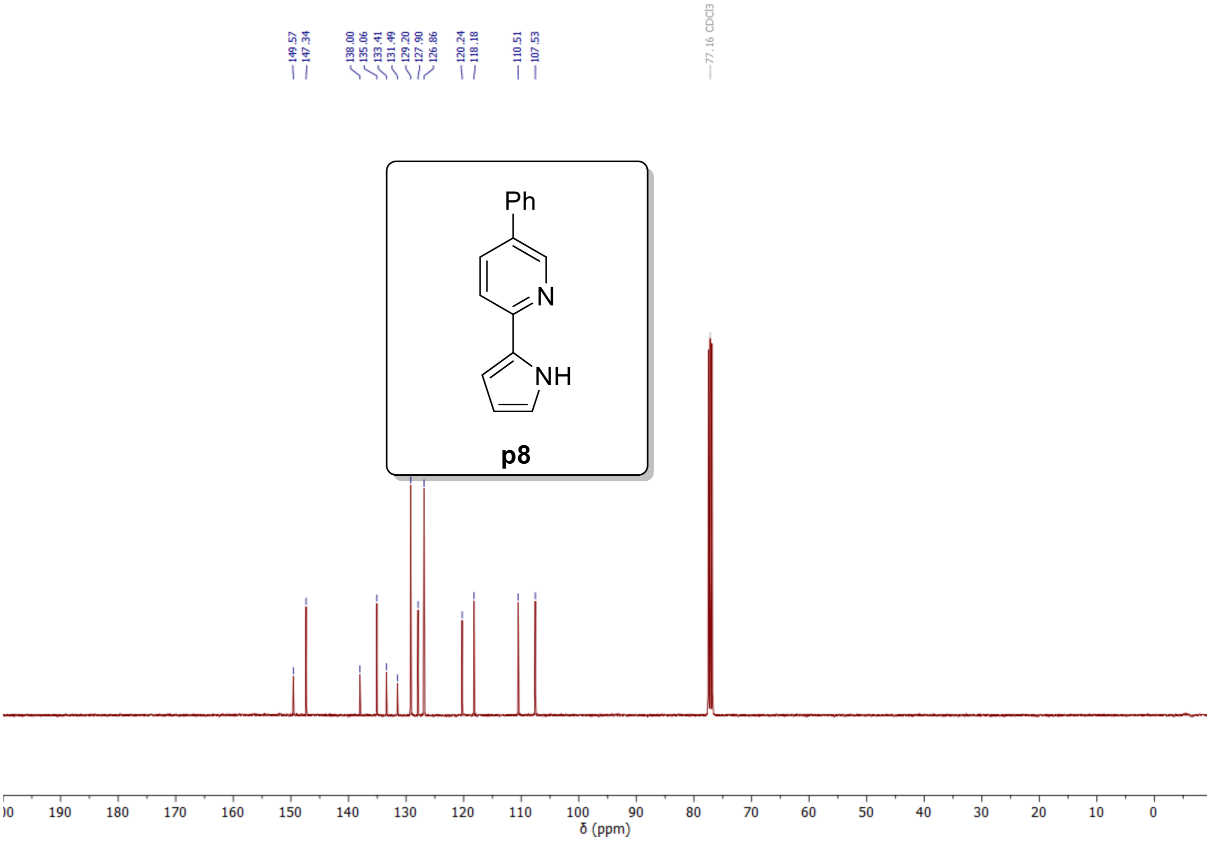
149.57 147.34
138.00 135.06 133.41 131.49 129.20 127.90 126.86
120.24 118.18
110.51 107.53
77.16 CDCl3
Ph
N
NH
p8
δ (ppm)

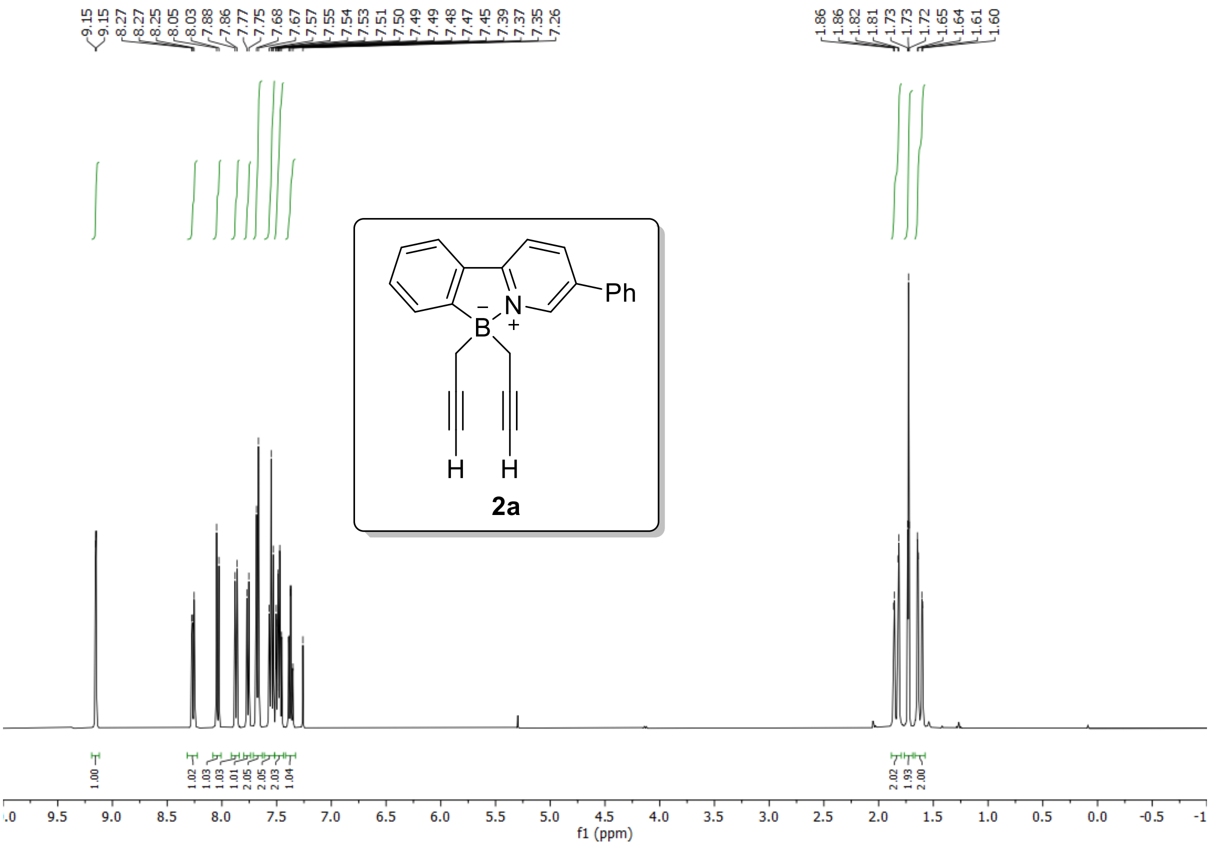
9.15
9.15
8.27
8.27
8.25
8.05
8.03
7.88
7.86
7.77
7.75
7.68
7.67
7.57
7.55
7.54
7.53
7.51
7.50
7.49
7.49
7.48
7.47
7.45
7.39
7.37
7.35
7.26
1.86
1.86
1.82
1.81
1.73
1.73
1.72
1.65
1.64
1.61
1.60
Ph
N
B
H
H
2a
1.00
1.02
1.03
1.03
1.01
2.05
2.05
2.03
1.04
2.02
1.93
2.00
f1 (ppm)

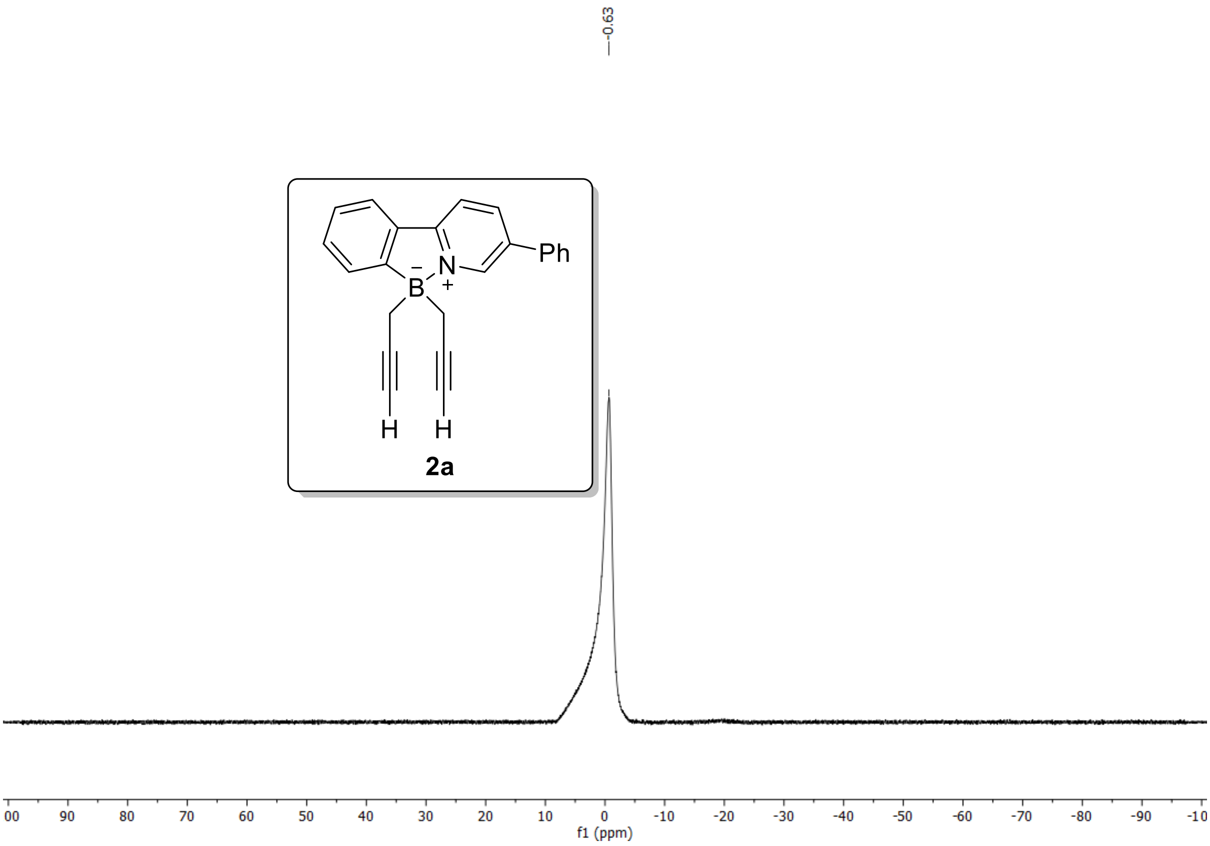
-0.63
Ph
N
B
H
H
2a
f1 (ppm)

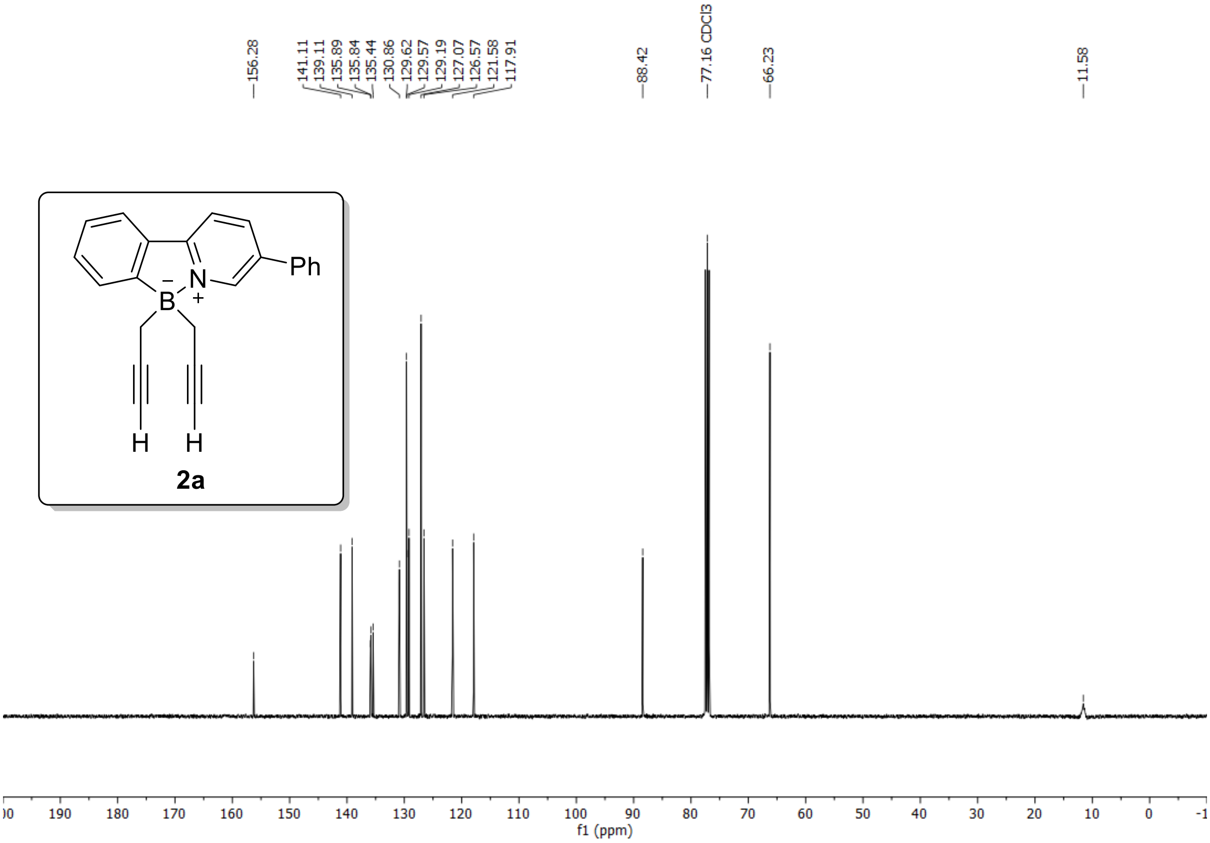

156.28
141.11
139.11
135.89
135.84
135.44
130.86
129.62
129.57
129.19
127.07
126.57
121.58
117.91
88.42
77.16 CDCl3
66.23
11.58
Ph
B
N
H H
2a
f1 (ppm)


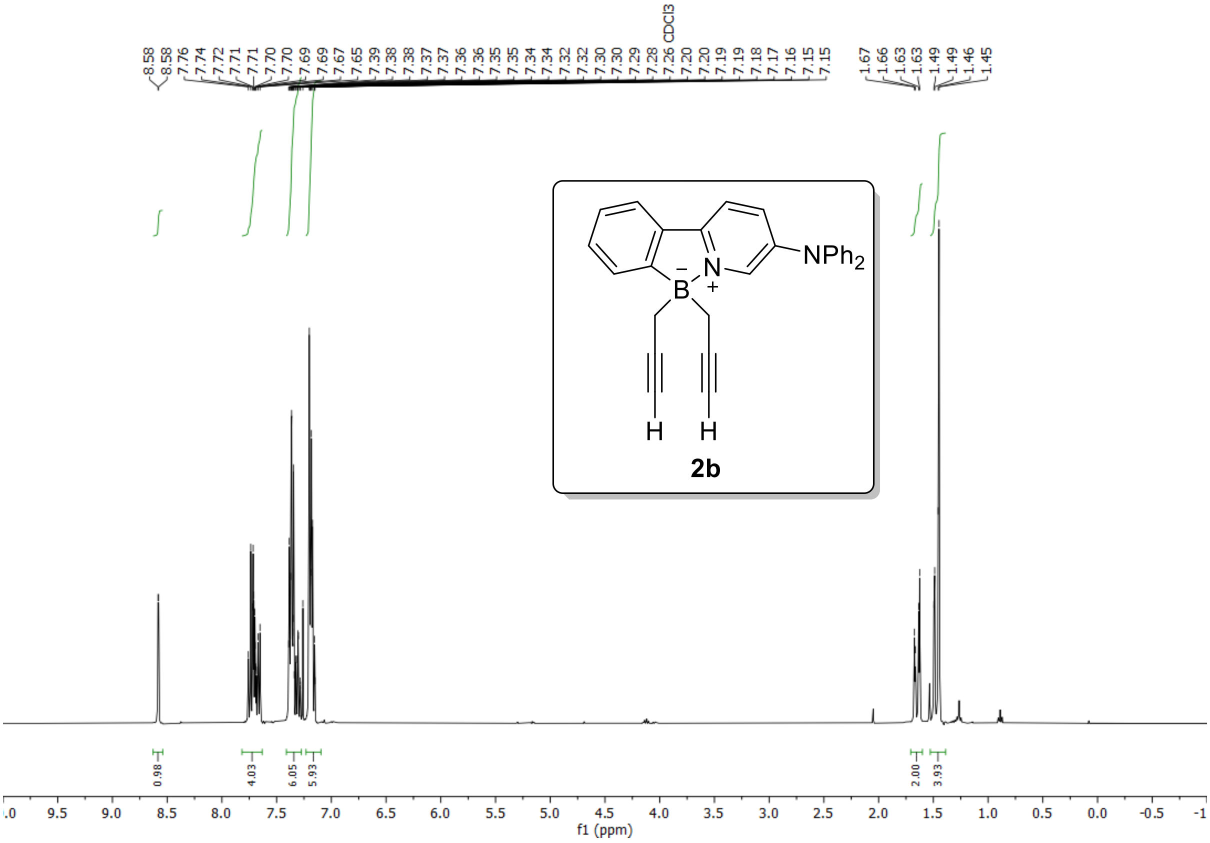

8.58
8.58
7.76
7.74
7.72
7.71
7.71
7.70
7.70
7.69
7.69
7.67
7.65
7.39
7.38
7.38
7.37
7.37
7.36
7.36
7.35
7.35
7.34
7.34
7.32
7.32
7.30
7.30
7.29
7.28
7.26 CDCl3
7.20
7.20
7.19
7.19
7.18
7.17
7.16
7.15
7.15
1.67
1.66
1.63
1.63
1.49
1.49
1.46
1.45
NPh2
B
N
H H
2b
0.98
4.03
6.05
5.93
2.00
3.93
f1 (ppm)

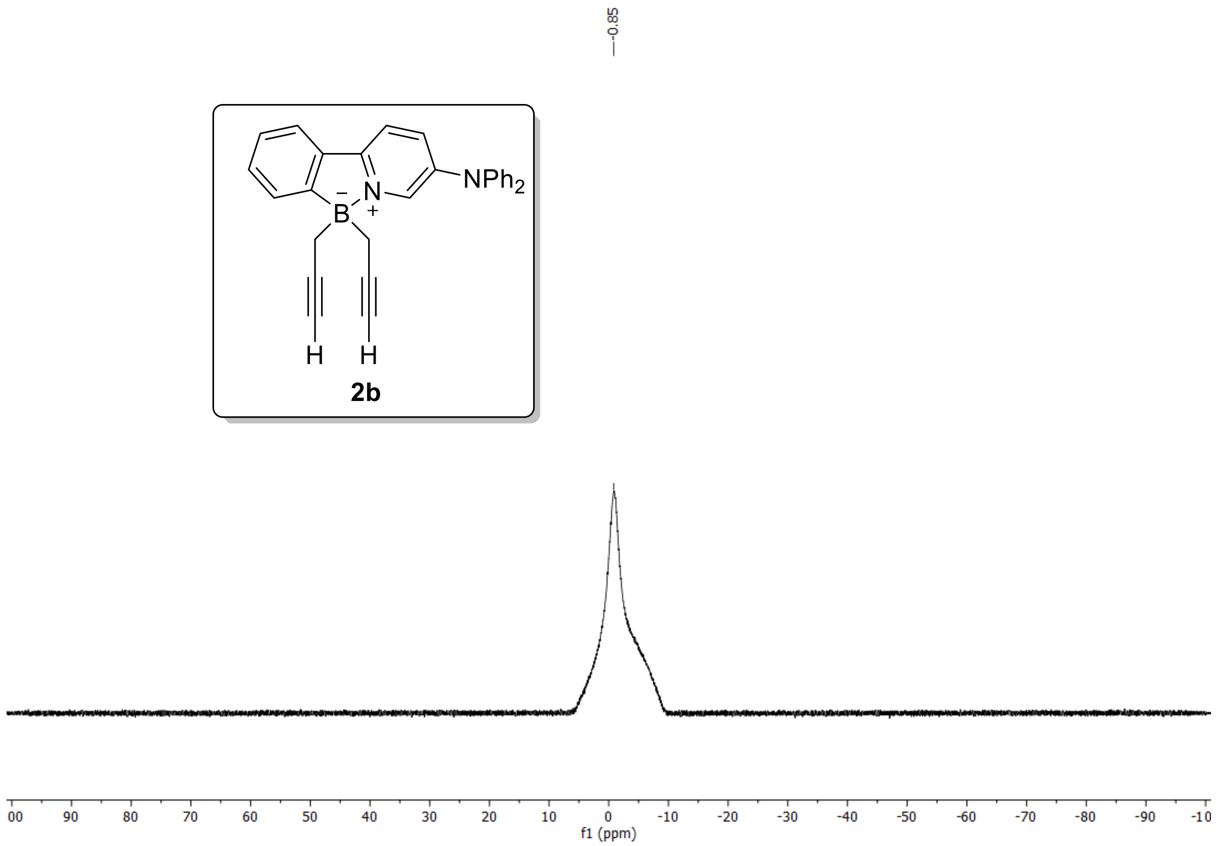
—0.85
NPh2
B
N
H H
2b
f1 (ppm)

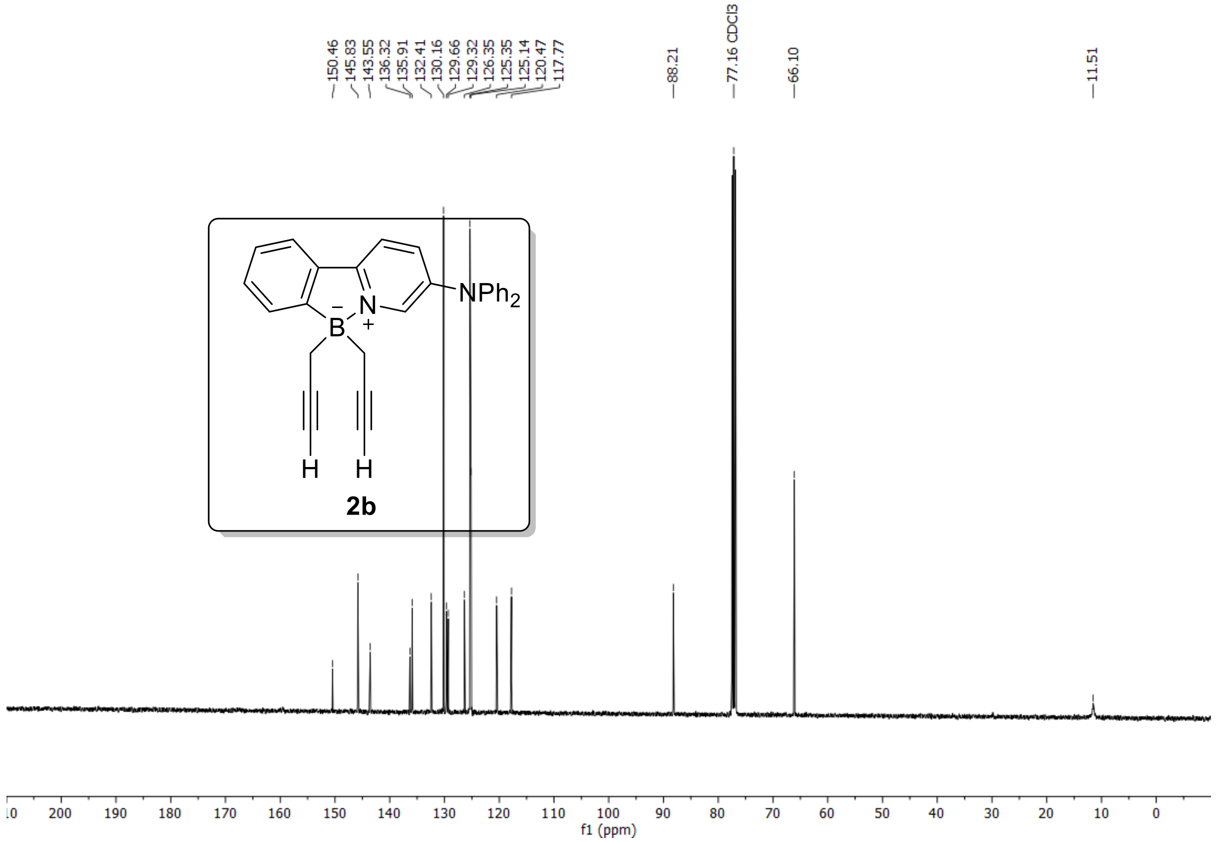
150.46
145.83
143.55
136.32
135.91
132.41
130.16
129.66
129.32
126.35
125.35
125.14
120.47
117.77
—88.21
—77.16 CDCl3
—66.10
—11.51
NPh2
B
N
H H
2b
f1 (ppm)

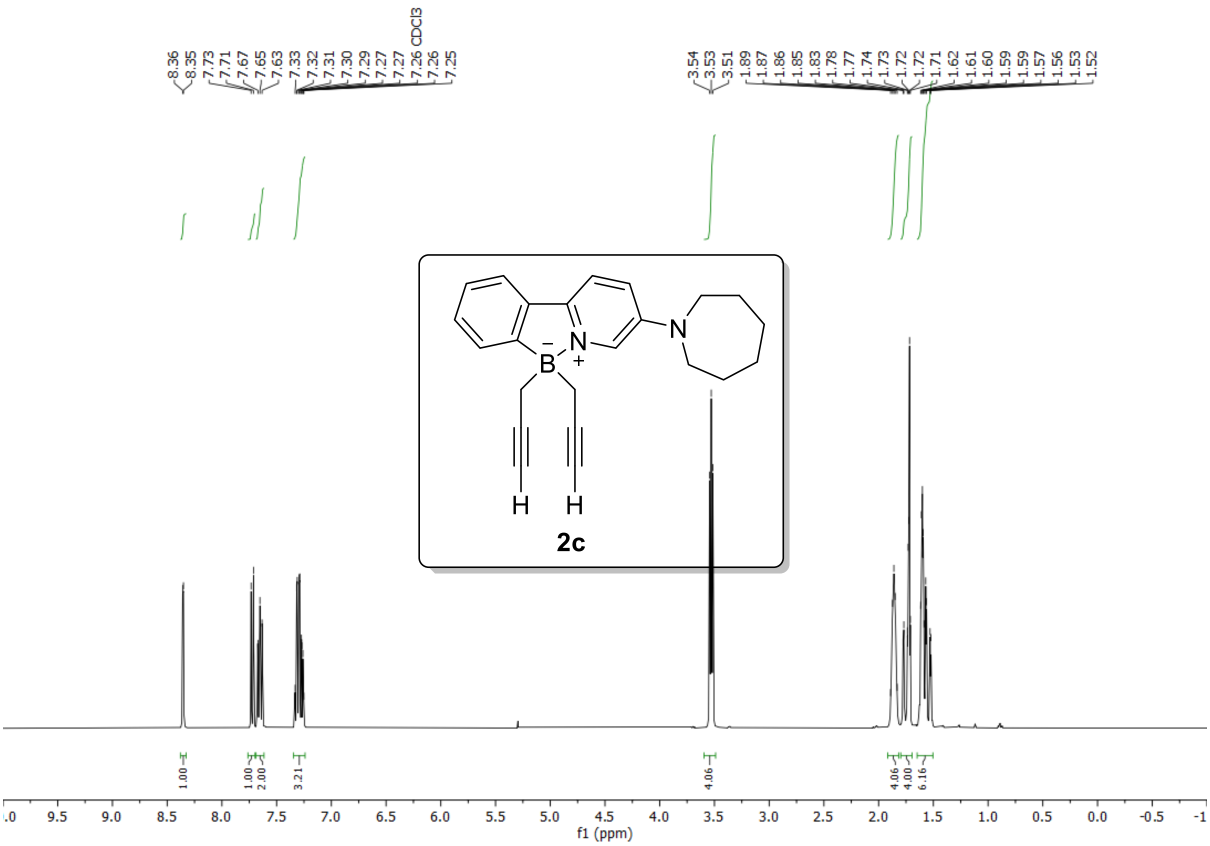
8.36
8.35
7.73
7.71
7.67
7.65
7.63
7.33
7.32
7.31
7.30
7.29
7.27
7.27
7.26 CDCl3
7.26
7.25
3.54
3.53
3.51
1.89
1.87
1.86
1.85
1.83
1.78
1.77
1.74
1.73
1.72
1.72
1.71
1.62
1.61
1.60
1.59
1.59
1.57
1.56
1.53
1.52
N
B
N
H
H
2c
1.00
1.00
2.00
3.21
4.06
4.06
4.00
6.16
f1 (ppm)

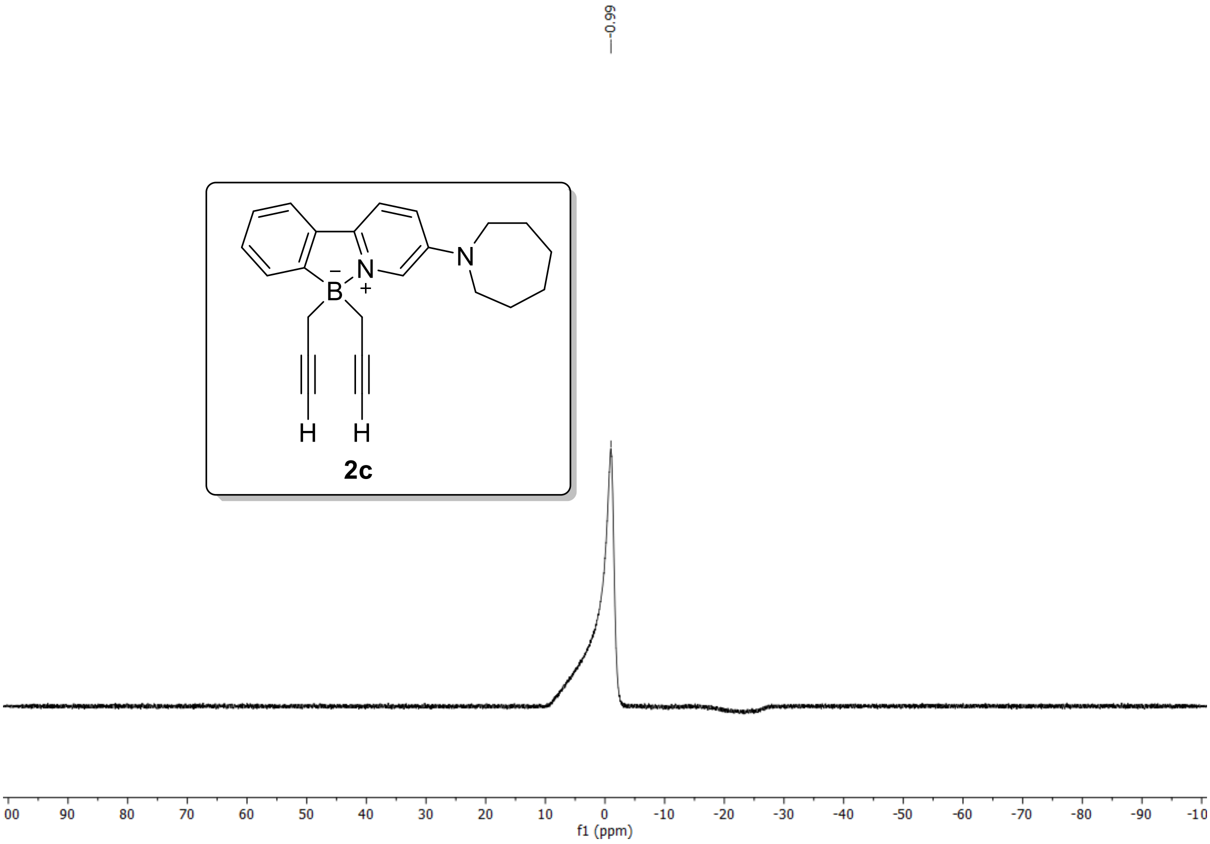
-0.99
N
B
N
H
H
2c
f1 (ppm)

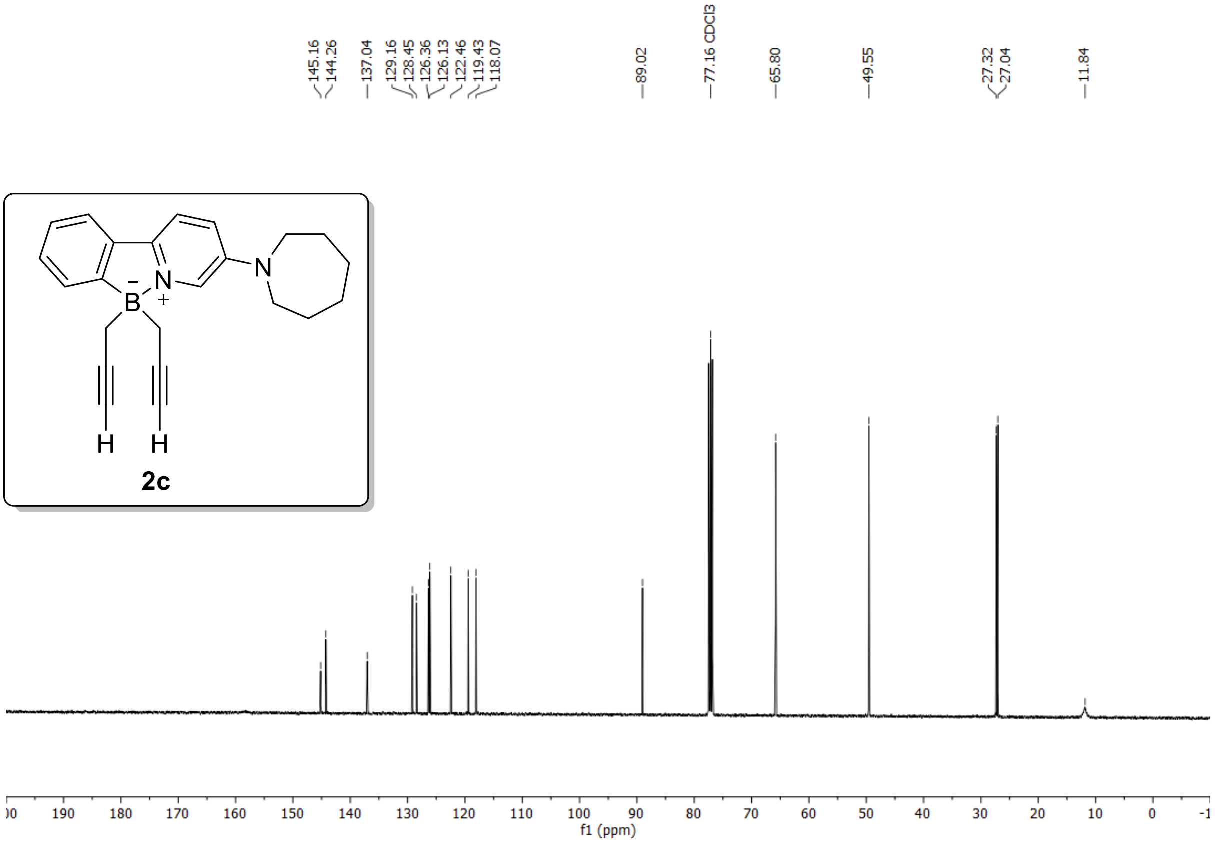
145.16
144.26
137.04
129.16
128.45
126.36
126.13
122.46
119.43
118.07
89.02
77.16 CDCl3
65.80
49.55
27.32
27.04
11.84
2c
f1 (ppm)

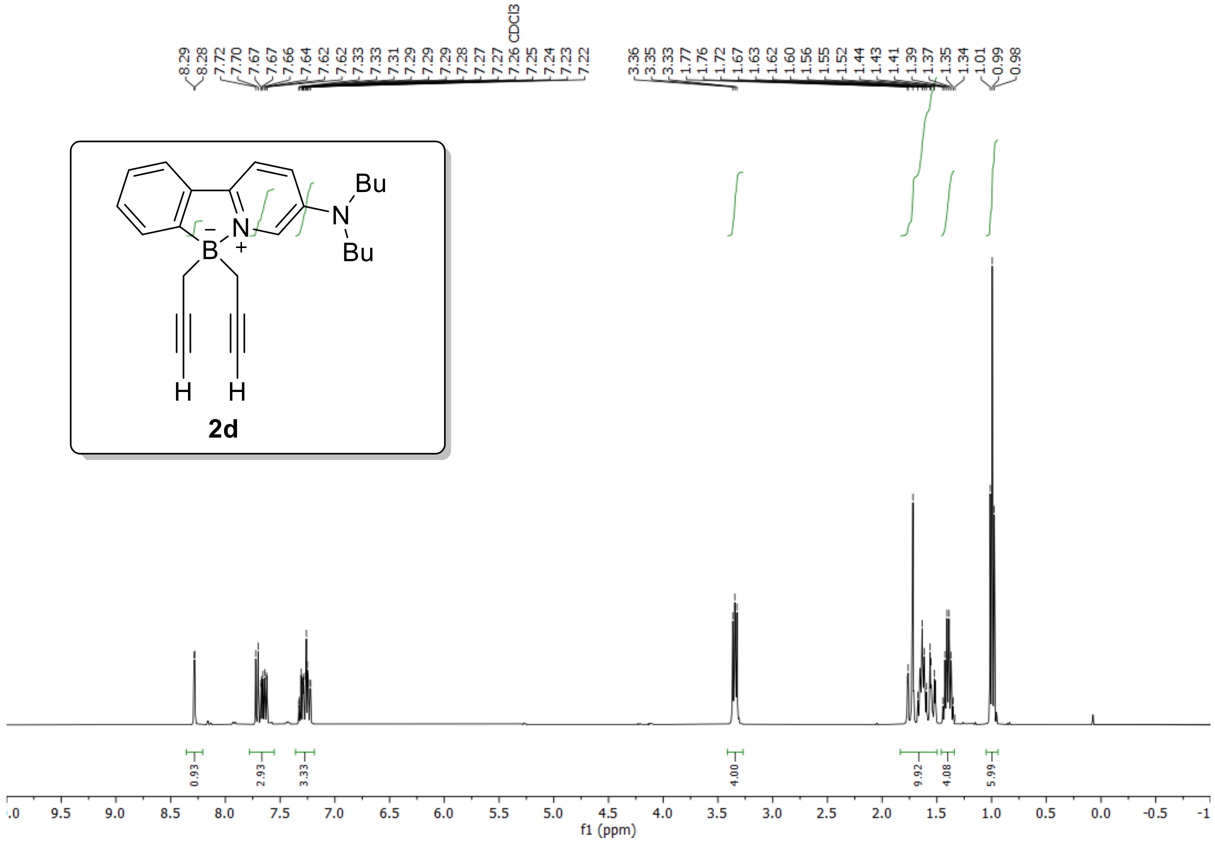
8.29
8.28
7.72
7.70
7.67
7.67
7.66
7.64
7.62
7.62
7.33
7.33
7.31
7.29
7.29
7.28
7.27
7.27
7.26 CDCl3
7.25
7.24
7.23
7.22
3.36
3.35
3.33
1.77
1.76
1.72
1.67
1.63
1.62
1.60
1.56
1.55
1.52
1.44
1.43
1.41
1.39
1.37
1.35
1.34
1.01
0.99
0.98
Bu
Bu
2d
0.93
2.93
3.33
4.00
9.92
4.08
5.99
f1 (ppm)

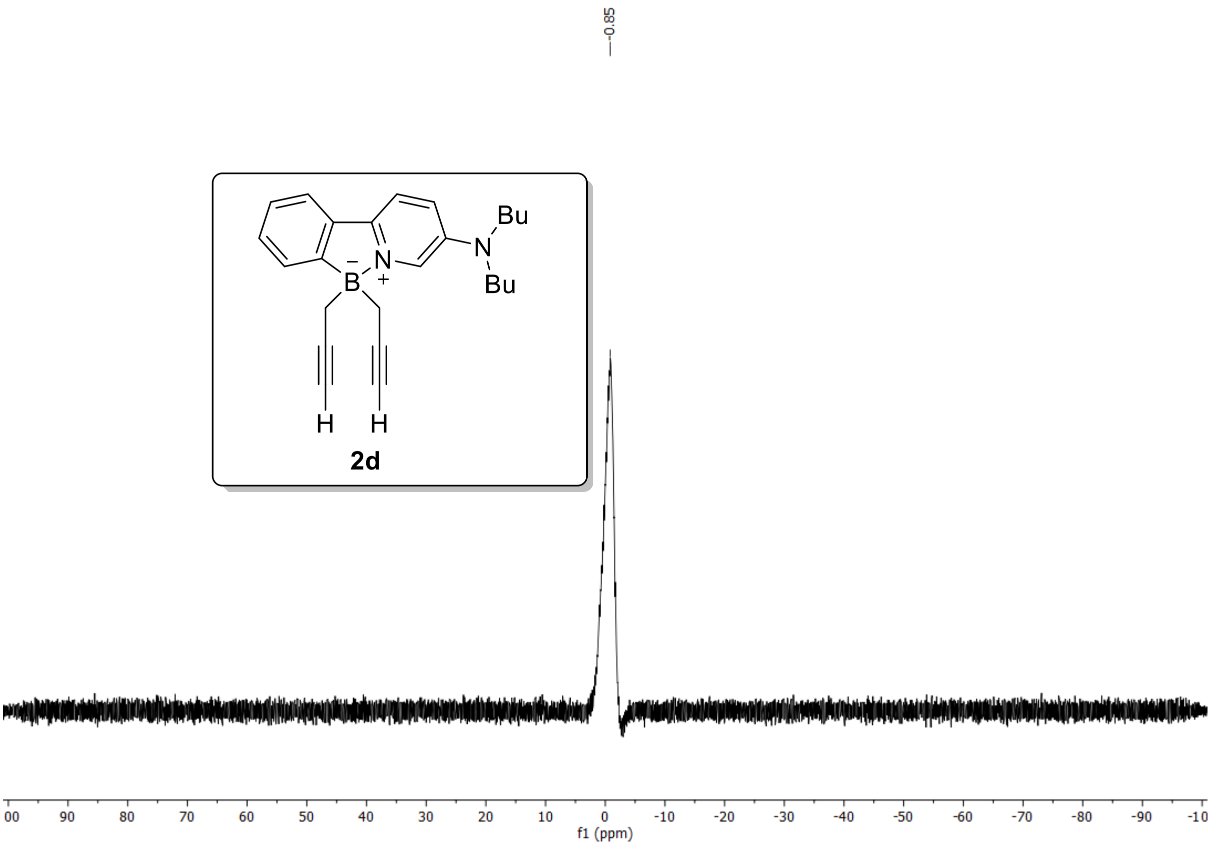

0.85
Bu
N
Bu
B
N
H H
2d
f1 (ppm)


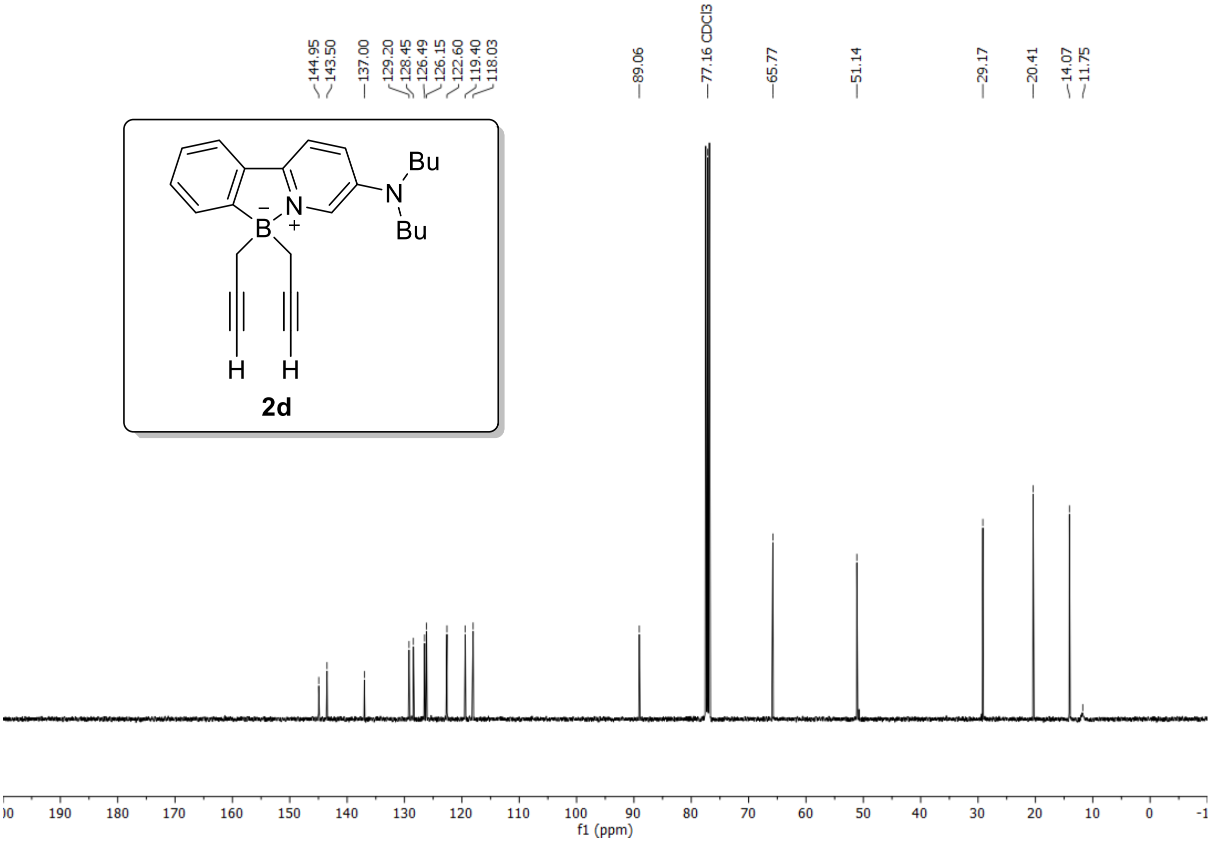

144.95
143.50
137.00
129.20
128.45
126.49
126.15
122.60
119.40
118.03
89.06
77.16 CDCl3
65.77
51.14
29.17
20.41
14.07
11.75
Bu
N
Bu
B
N
H H
2d
f1 (ppm)

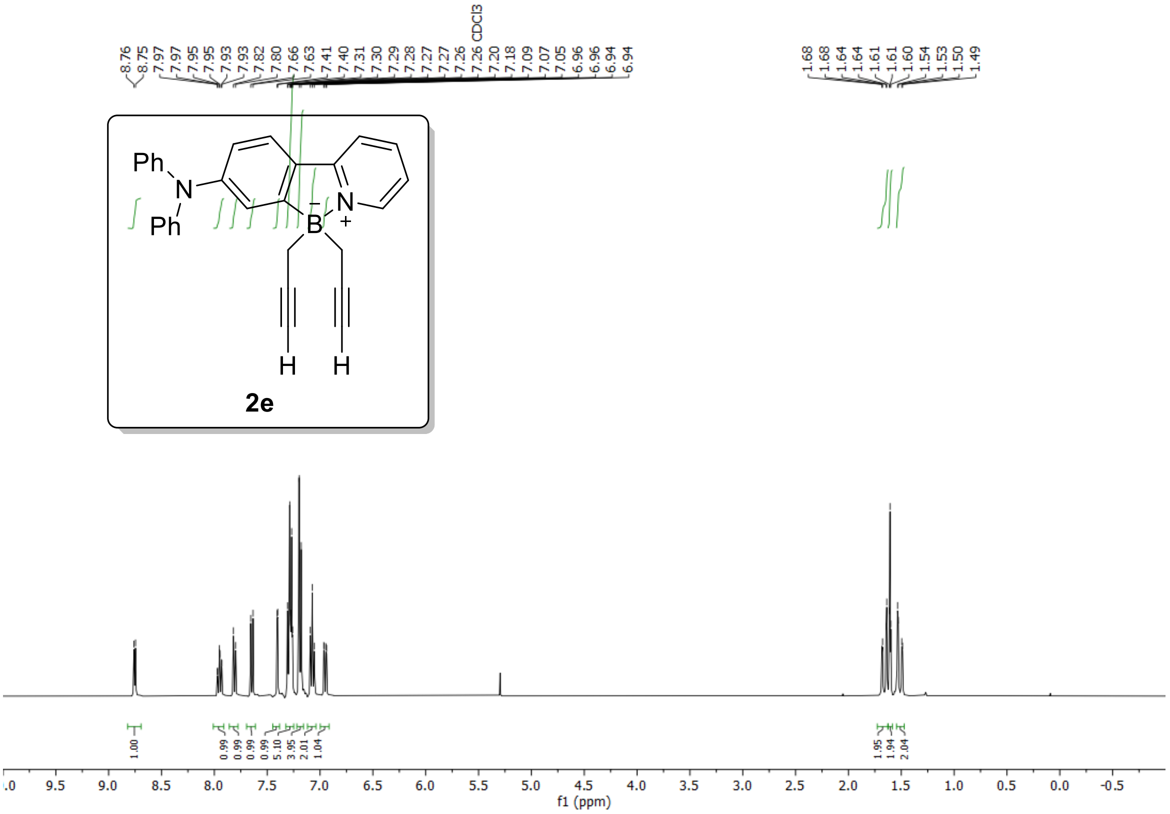

8.76
8.75
7.97
7.97
7.95
7.95
7.93
7.93
7.82
7.80
7.66
7.63
7.41
7.40
7.31
7.30
7.29
7.28
7.27
7.27
7.26
7.26 CDCl3
7.20
7.18
7.09
7.07
7.05
6.96
6.96
6.94
6.94
1.68
1.68
1.64
1.64
1.61
1.61
1.60
1.54
1.53
1.50
1.49
Ph
N
Ph
B
N
H
H
2e
1.00
0.99
0.99
0.99
0.99
5.10
3.95
2.01
1.04
1.95
1.94
2.04
f1 (ppm)


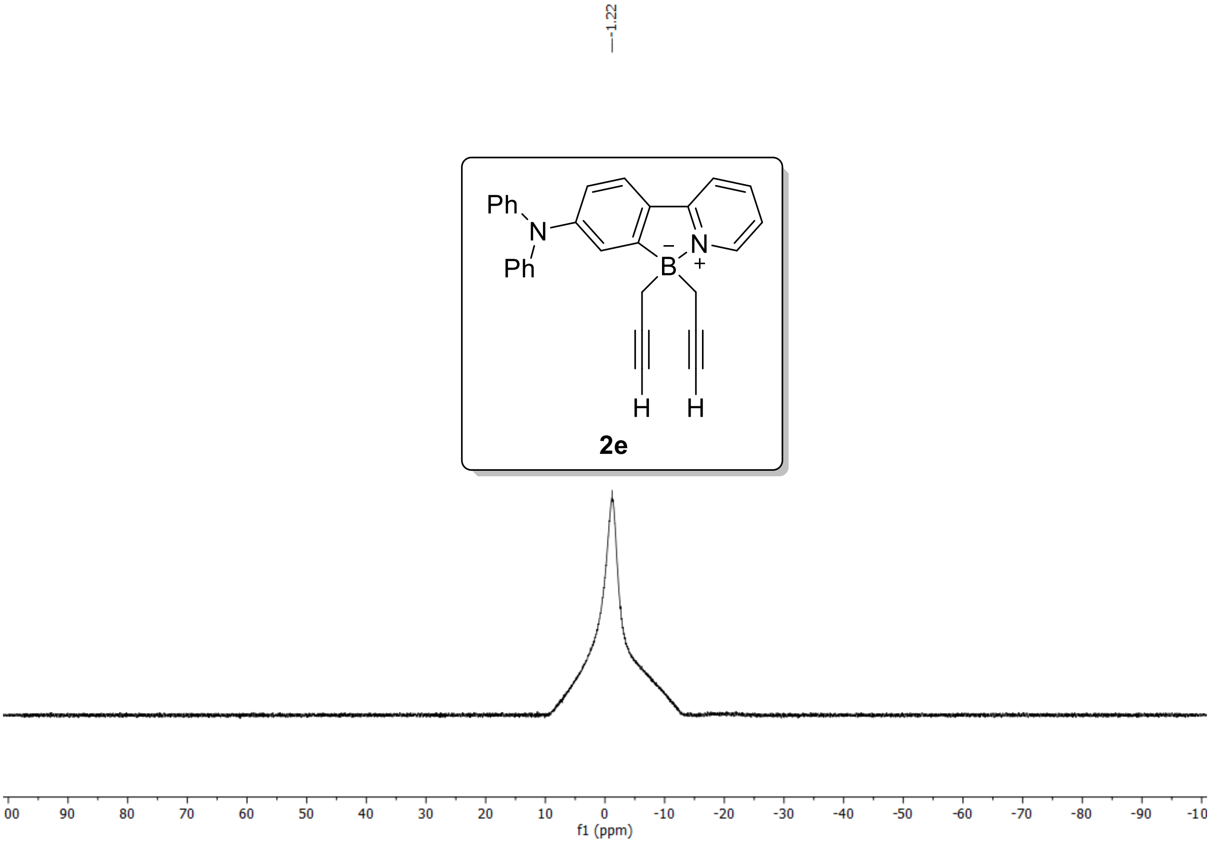

-1.22
Ph
N
Ph
B
N
H
H
2e
f1 (ppm)

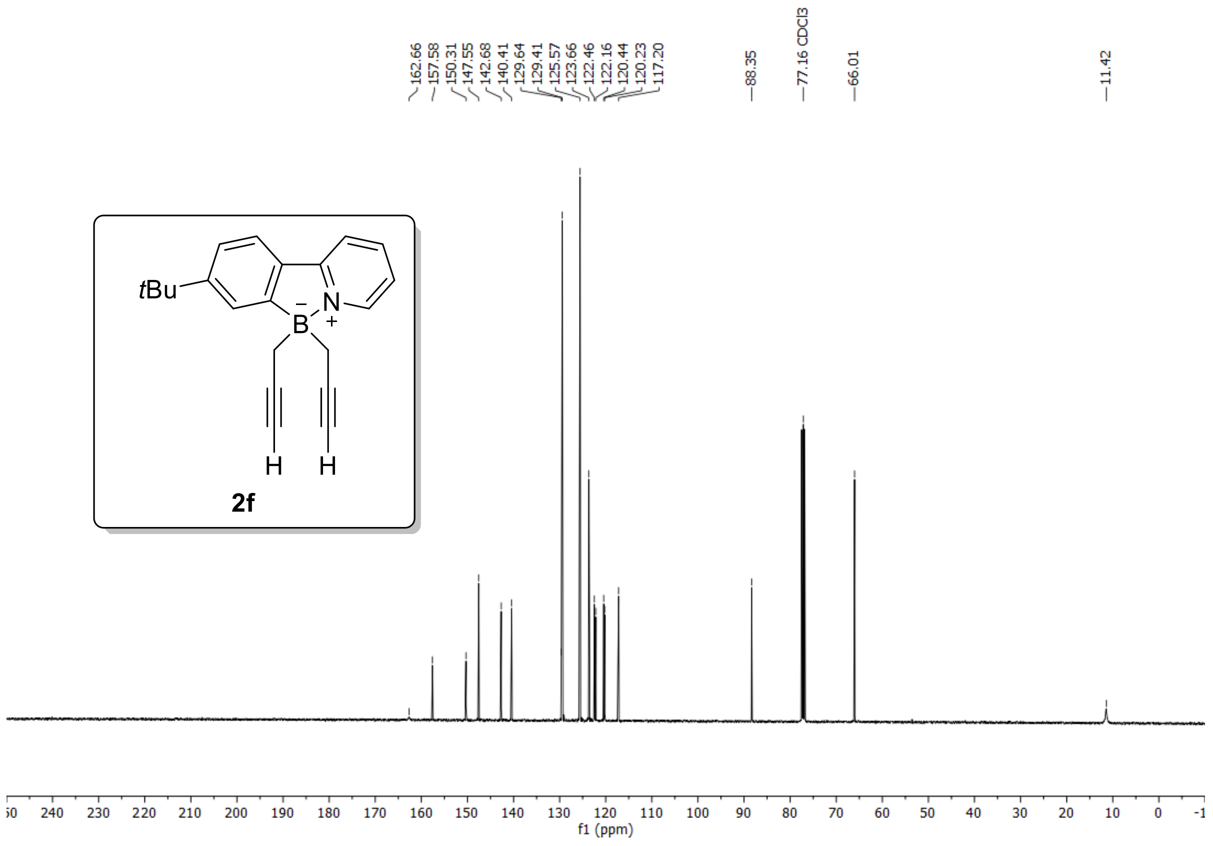
162.66
157.58
150.31
147.55
142.68
140.41
129.64
129.41
125.57
123.66
122.46
122.16
120.44
120.23
117.20
88.35
77.16 CDCl3
66.01
11.42
tBu
B
N
H
H
2f
f1 (ppm)

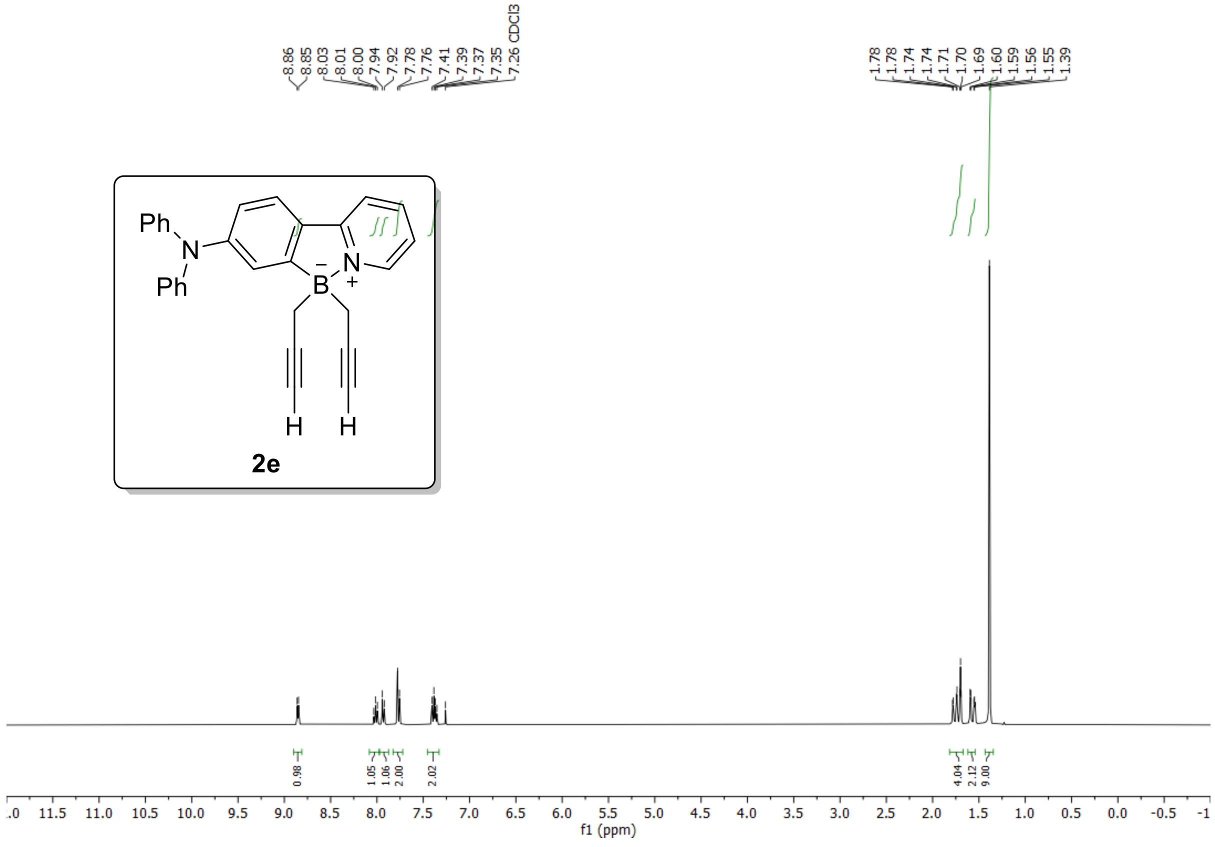
8.86
8.85
8.03
8.01
8.00
7.94
7.92
7.78
7.76
7.41
7.39
7.37
7.35
7.26 CDCl3
1.78
1.78
1.74
1.74
1.71
1.70
1.69
1.60
1.59
1.56
1.55
1.39
Ph
N
Ph
B
N
H
H
2e
0.98
1.05
1.06
2.00
2.02
4.04
2.12
9.00
f1 (ppm)

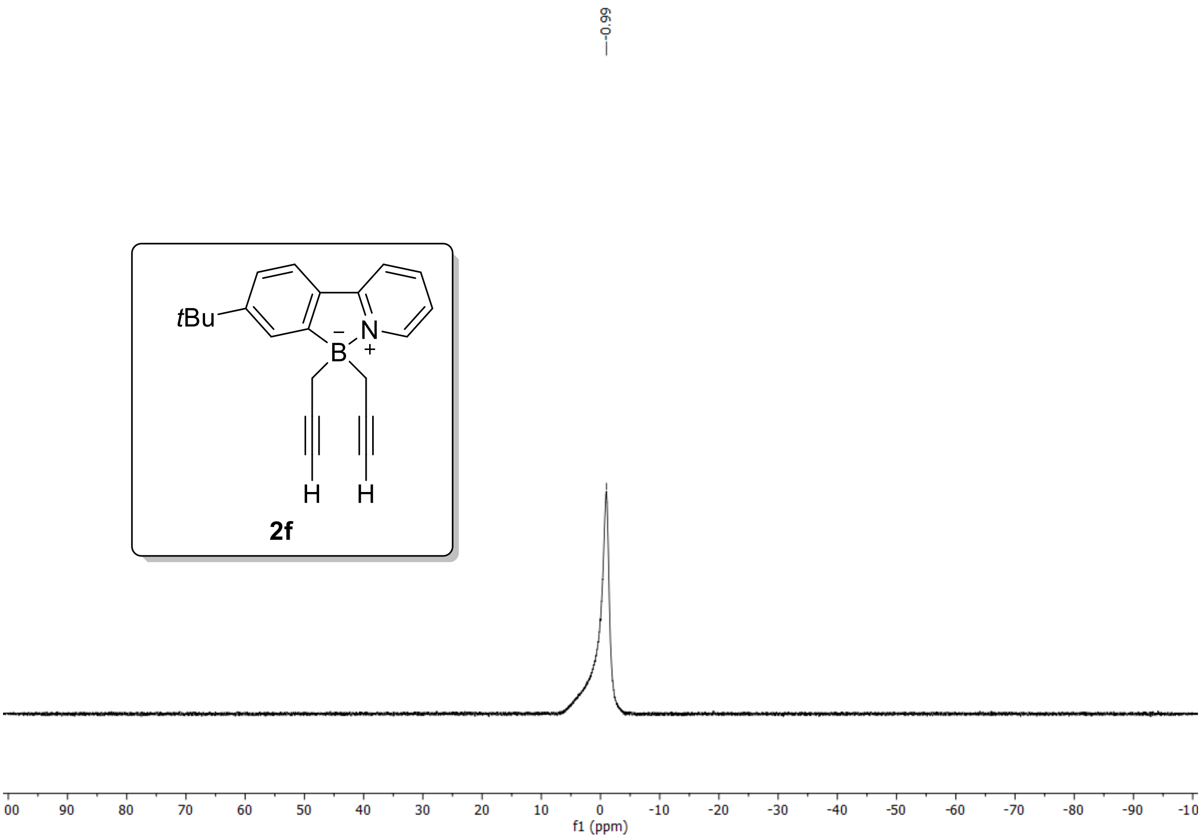
-0.99
tBu
B
N
H H
2f
f1 (ppm)

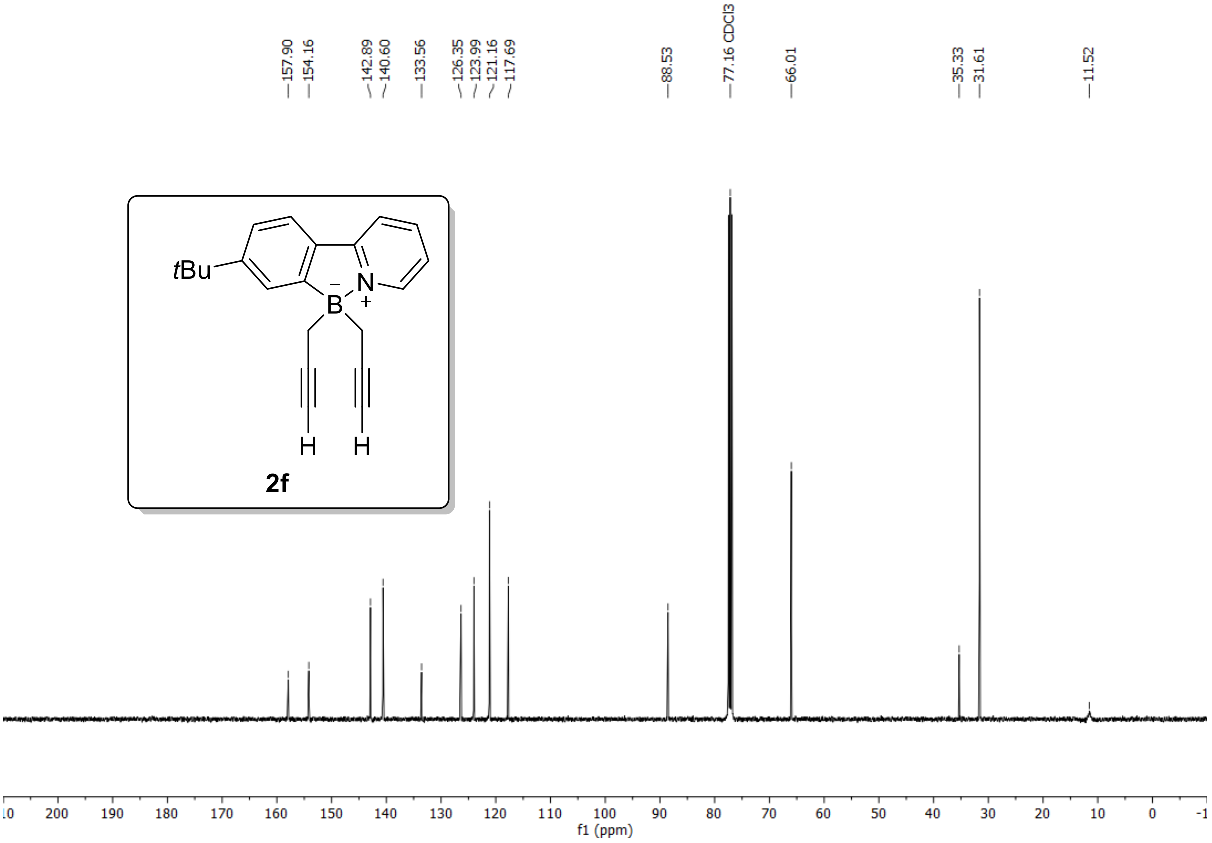
157.90
154.16
142.89
140.60
133.56
126.35
123.99
121.16
117.69
88.53
77.16 CDCl3
66.01
35.33
31.61
11.52
tBu
B
N
H H
2f
f1 (ppm)

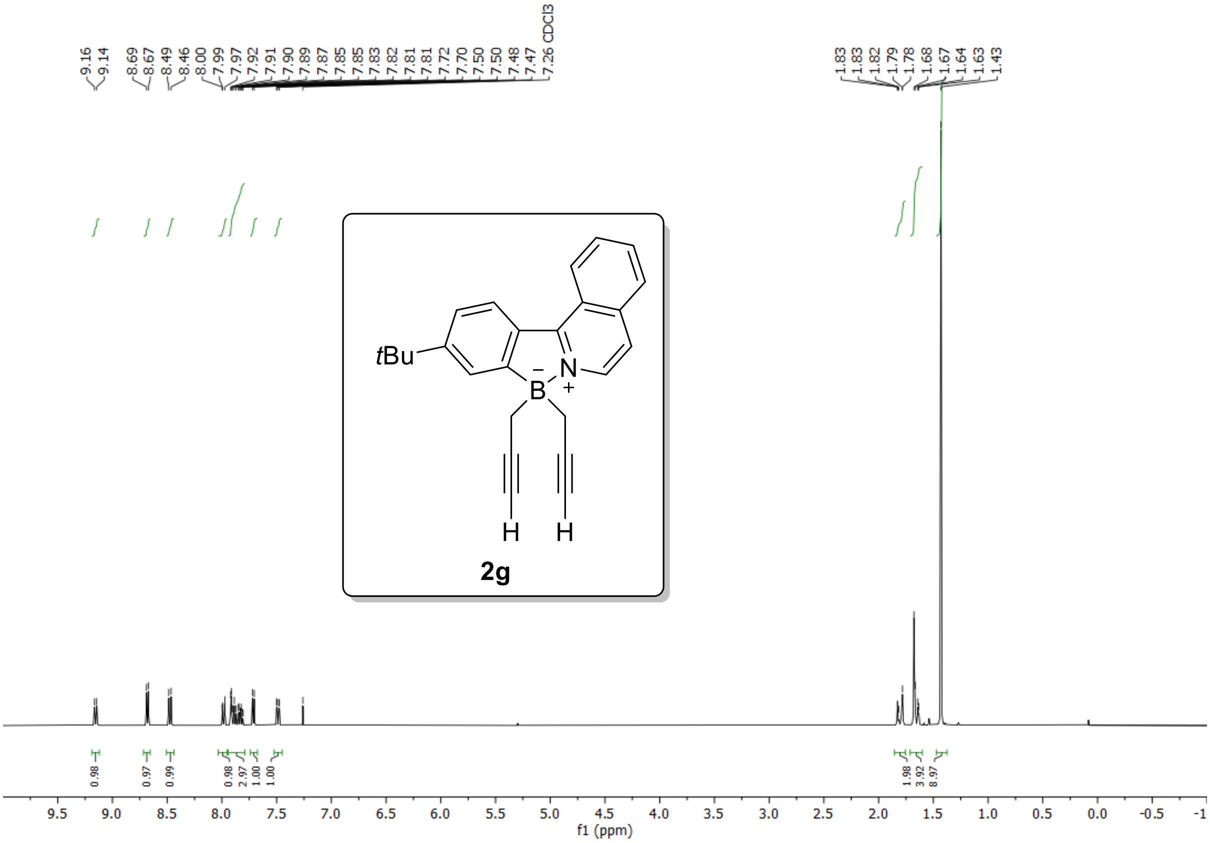
tBu
B
N
H H
2g
f1 (ppm)

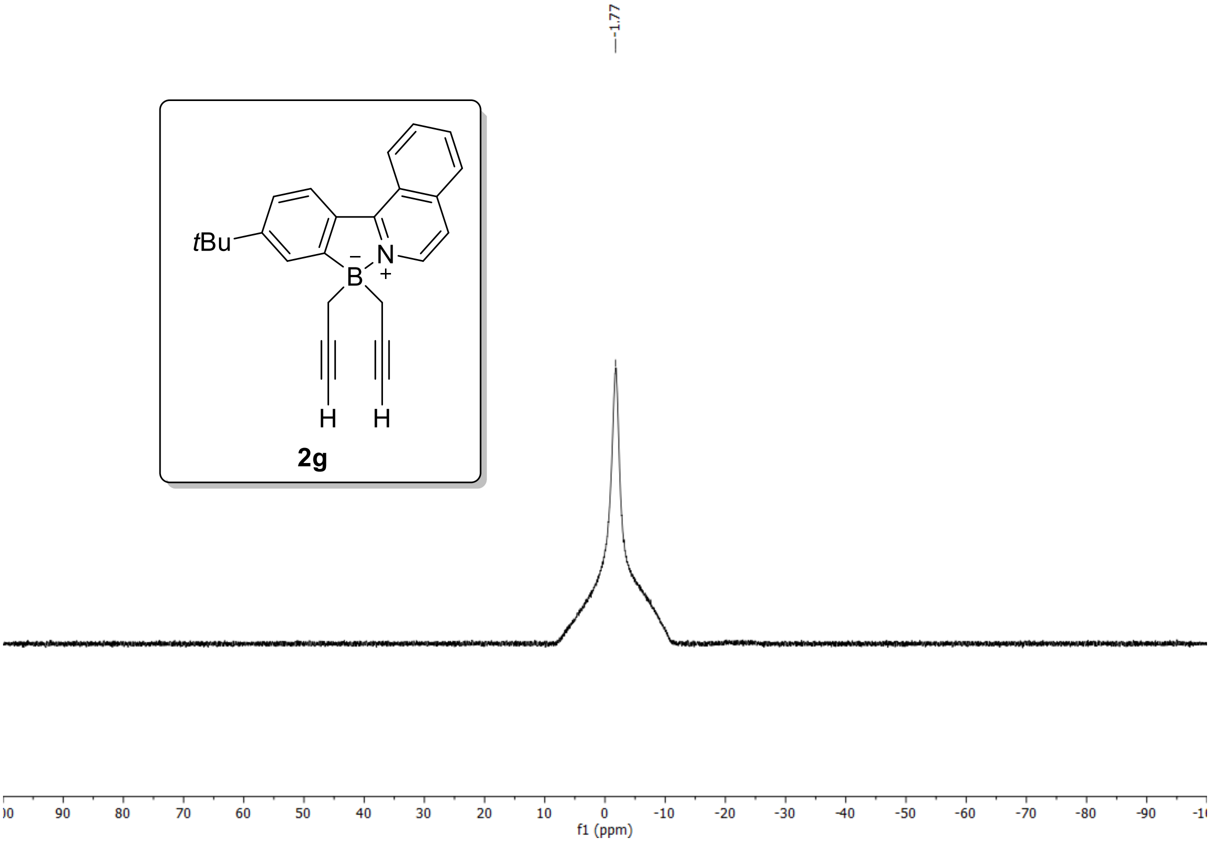
-1.77
tBu
B
N
H H
2g
f1 (ppm)

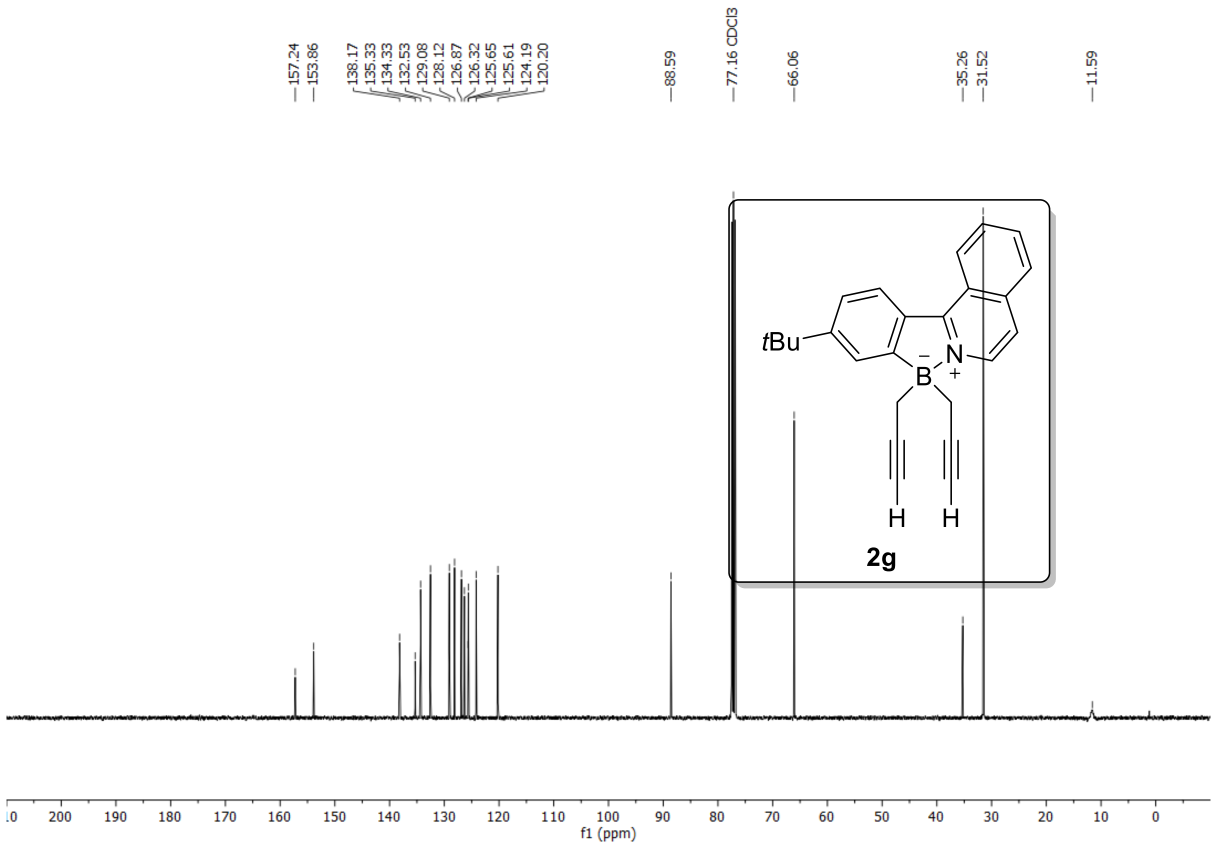
157.24
153.86
138.17
135.33
134.33
132.53
129.08
128.12
126.87
126.32
125.65
125.61
124.19
120.20
88.59
77.16 CDCl3
66.06
35.26
31.52
11.59
tBu
B
N
H
H
2g
f1 (ppm)

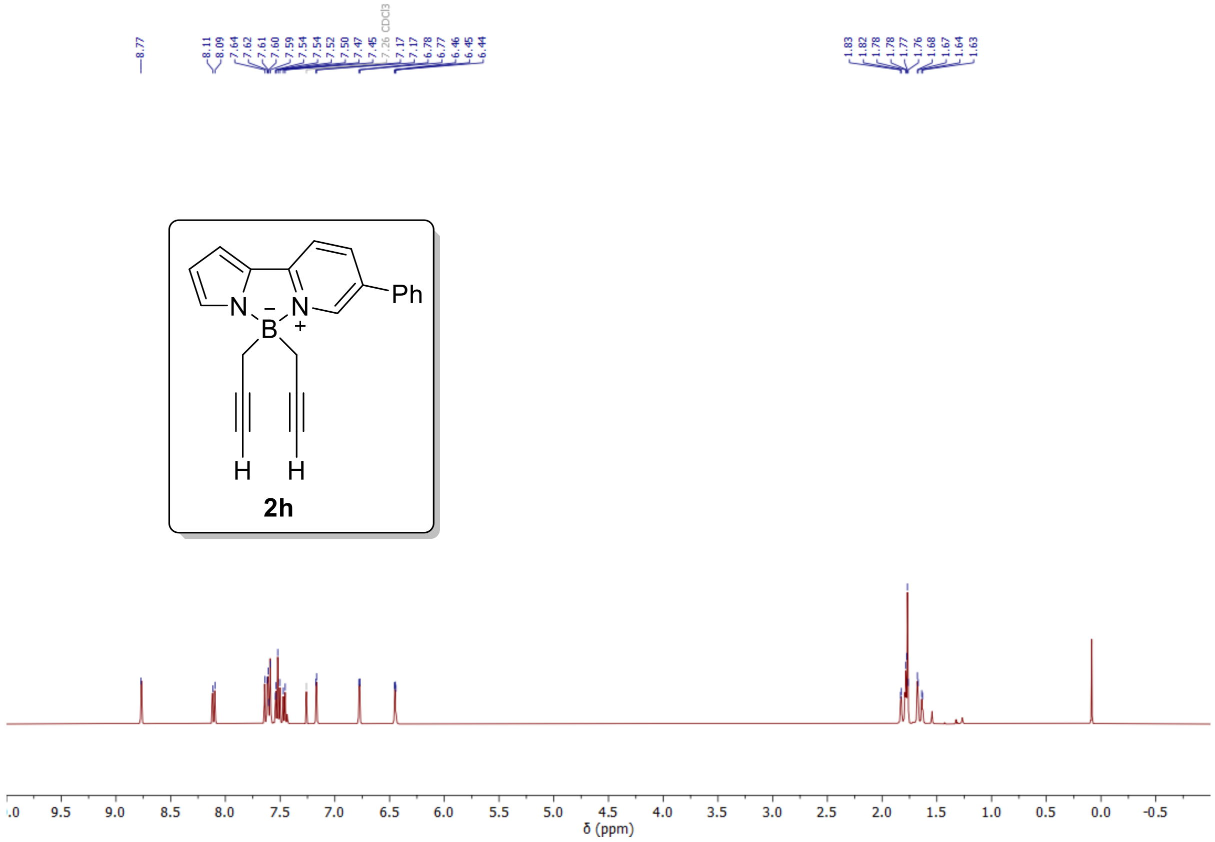
8.77
8.11
8.09
7.64
7.62
7.61
7.60
7.59
7.54
7.54
7.52
7.50
7.47
7.45
7.26 CDCl3
7.17
7.17
6.78
6.77
6.46
6.45
6.44
1.83
1.82
1.78
1.77
1.76
1.68
1.67
1.64
1.63
N
B
N
Ph
H
H
2h
δ (ppm)

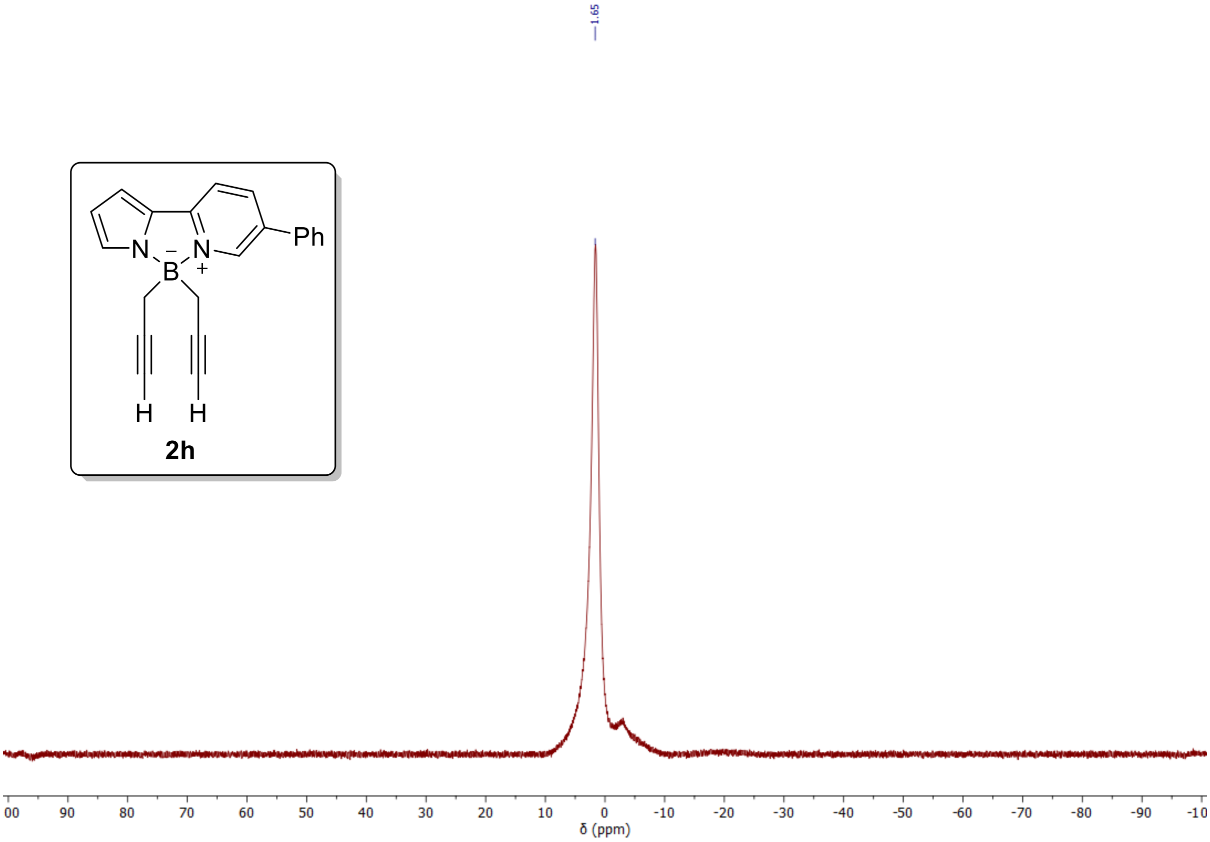
1.65
Ph
N
B
N
H
H
2h
δ (ppm)

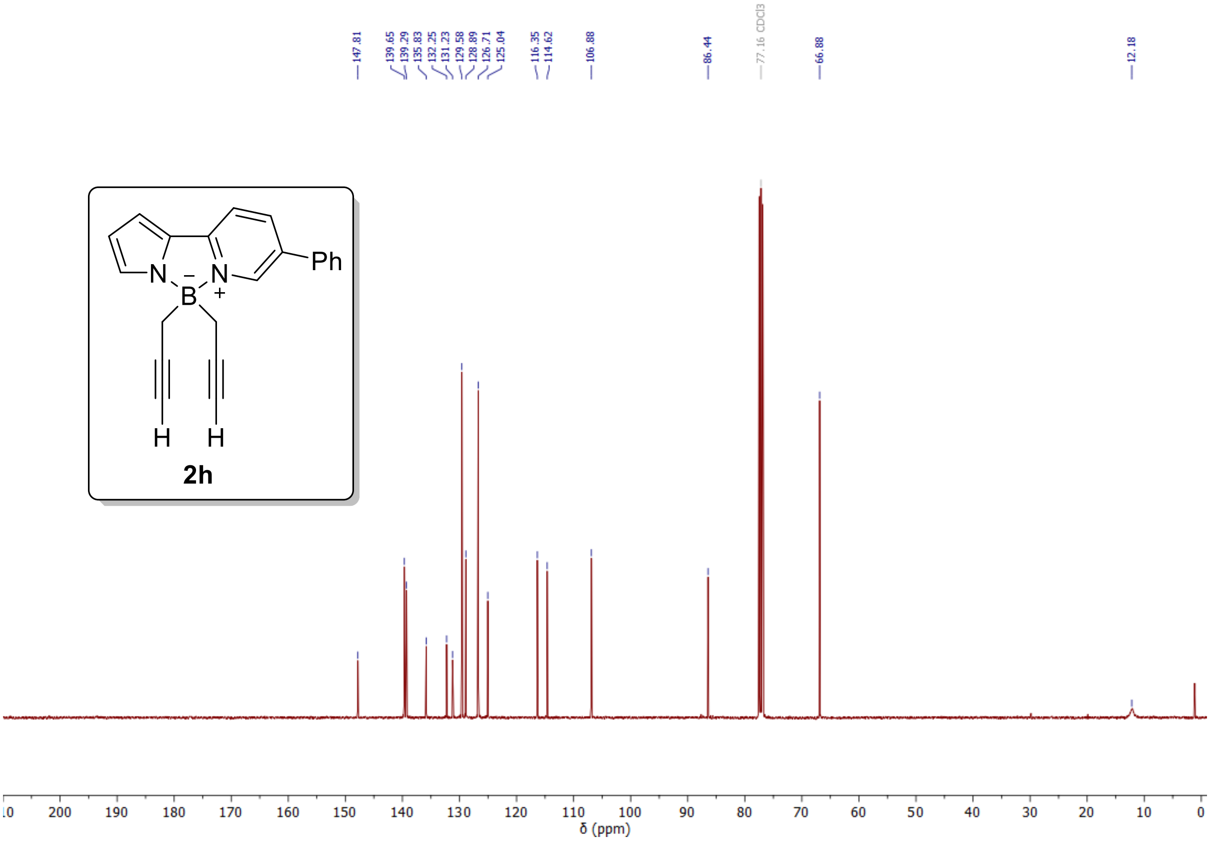
147.81
139.65
139.29
135.83
132.25
131.23
129.58
128.89
126.71
125.04
116.35
114.62
106.88
86.44
77.16 CDCl3
66.88
12.18
Ph
N
B
N
H
H
2h
δ (ppm)

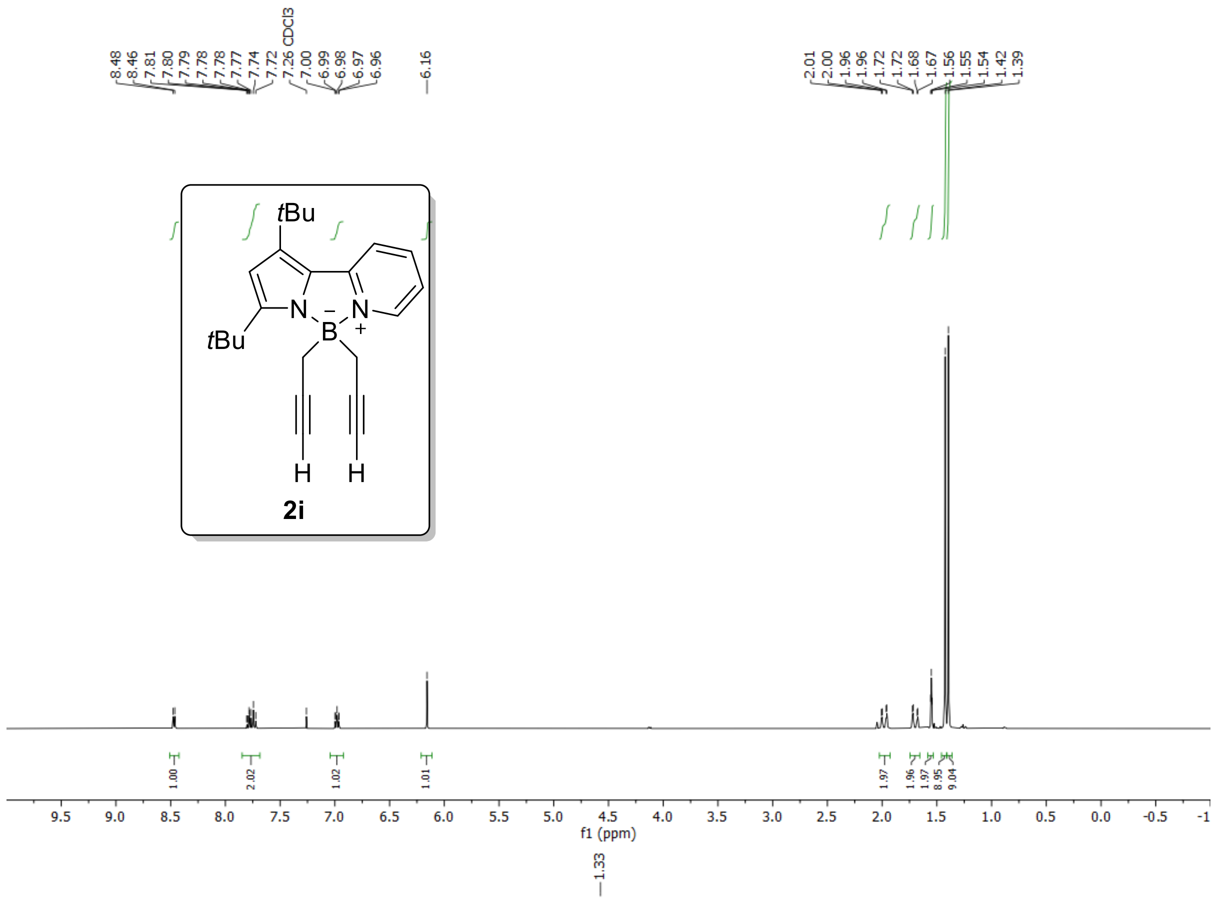
8.48
8.46
7.81
7.80
7.79
7.78
7.78
7.77
7.74
7.72
7.26 CDCl3
7.00
6.99
6.98
6.97
6.96
6.16
2.01
2.00
1.96
1.96
1.72
1.72
1.68
1.67
1.56
1.55
1.54
1.42
1.39
tBu
tBu
N
N
B
H
H
2i
1.00
2.02
1.02
1.01
1.97
1.96
1.97
8.95
9.04
f1 (ppm)

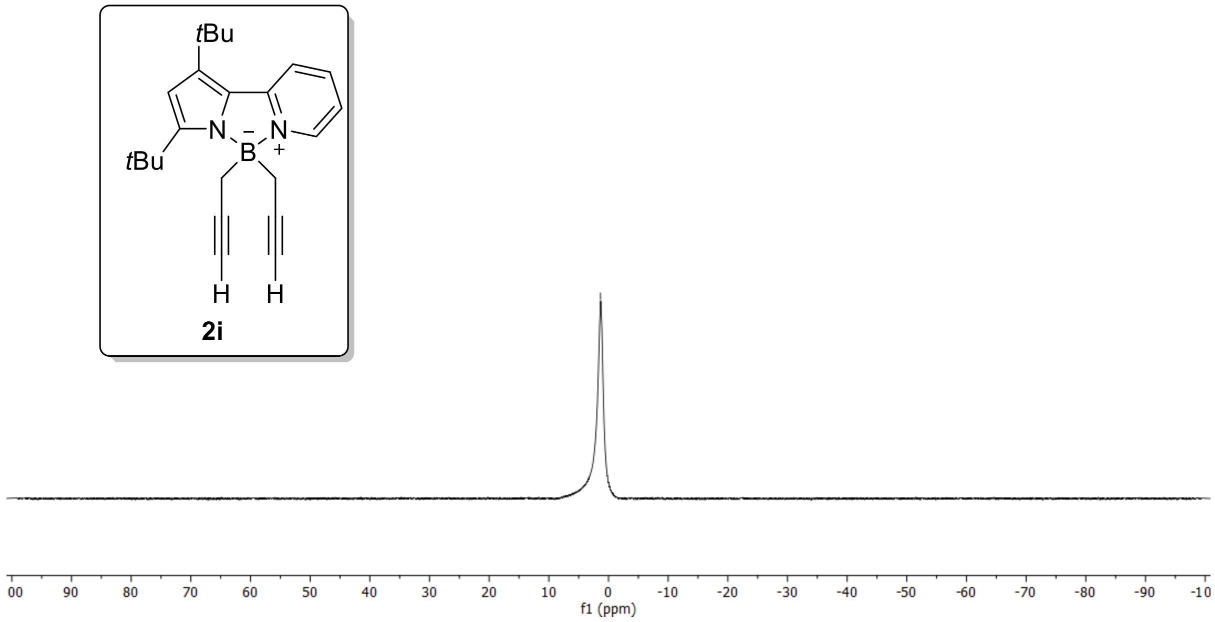
1.33
tBu
tBu
N
N
B
H
H
2i
f1 (ppm)

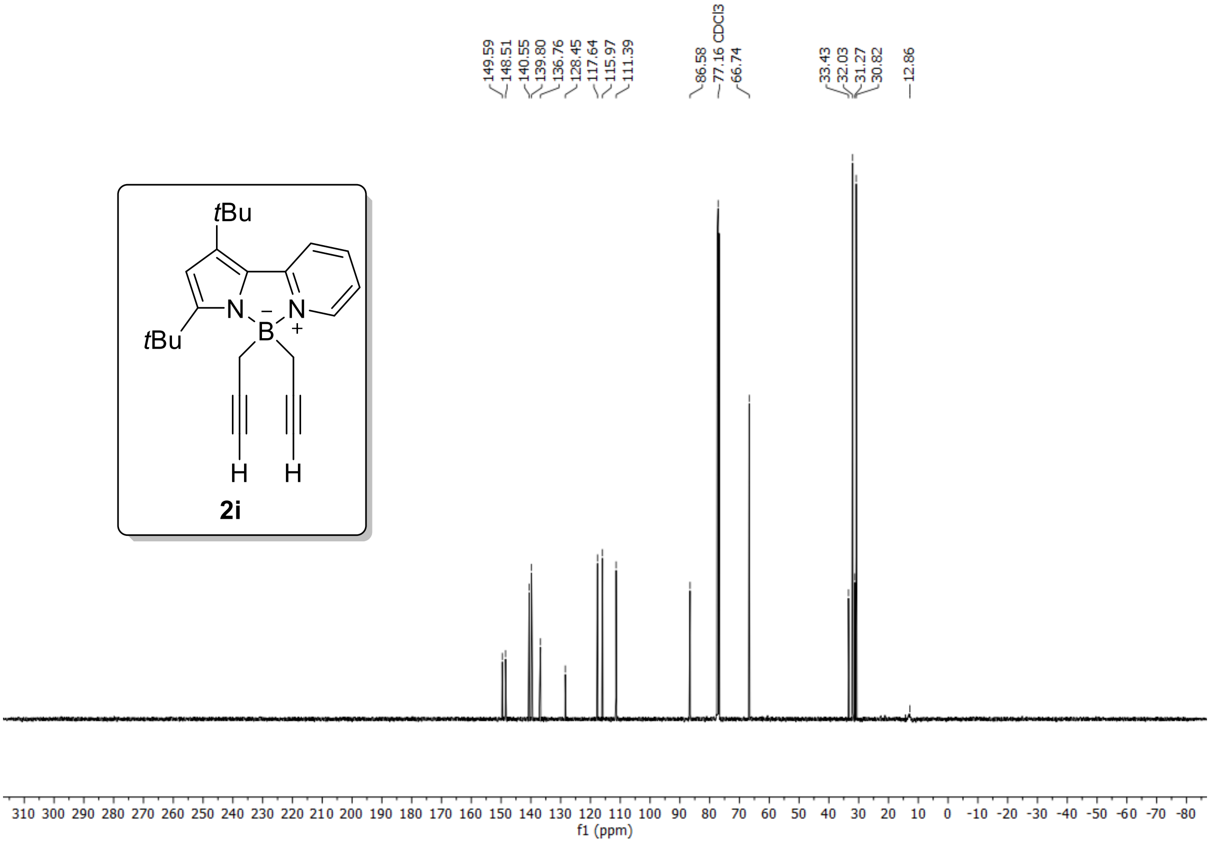
149.59
148.51
140.55
139.80
136.76
128.45
117.64
115.97
111.39
86.58
77.16 CDCl3
66.74
33.43
32.03
31.27
30.82
12.86
tBu
N
B
N
tBu
H H
2i
f1 (ppm)

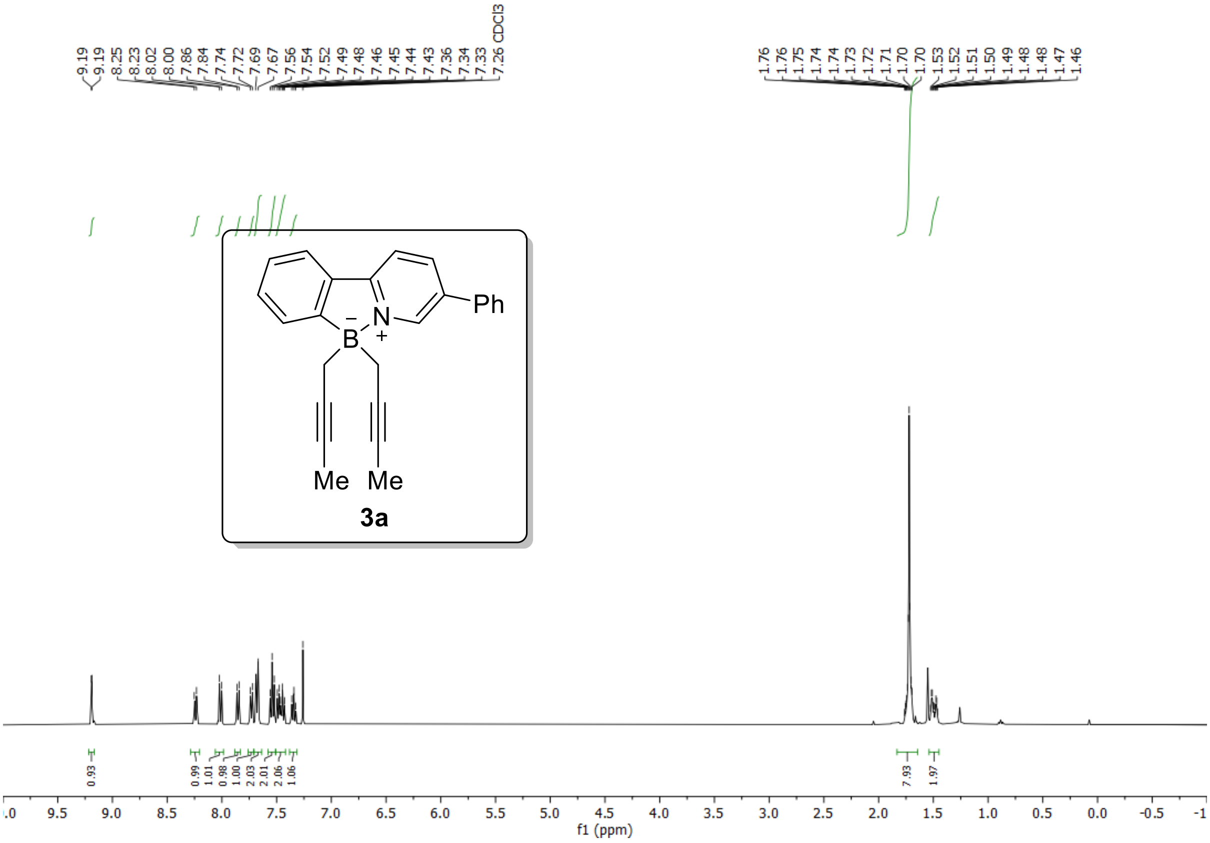
9.19
9.19
8.25
8.23
8.02
8.00
7.86
7.84
7.74
7.72
7.69
7.67
7.56
7.54
7.52
7.49
7.48
7.46
7.45
7.44
7.43
7.36
7.34
7.33
7.26 CDCl3
1.76
1.76
1.75
1.74
1.74
1.73
1.72
1.71
1.70
1.70
1.53
1.52
1.51
1.50
1.49
1.48
1.48
1.47
1.46
B
N
Ph
Me Me
3a
0.93
0.99
1.01
0.98
1.00
2.03
2.01
2.06
1.06
7.93
1.97
f1 (ppm)

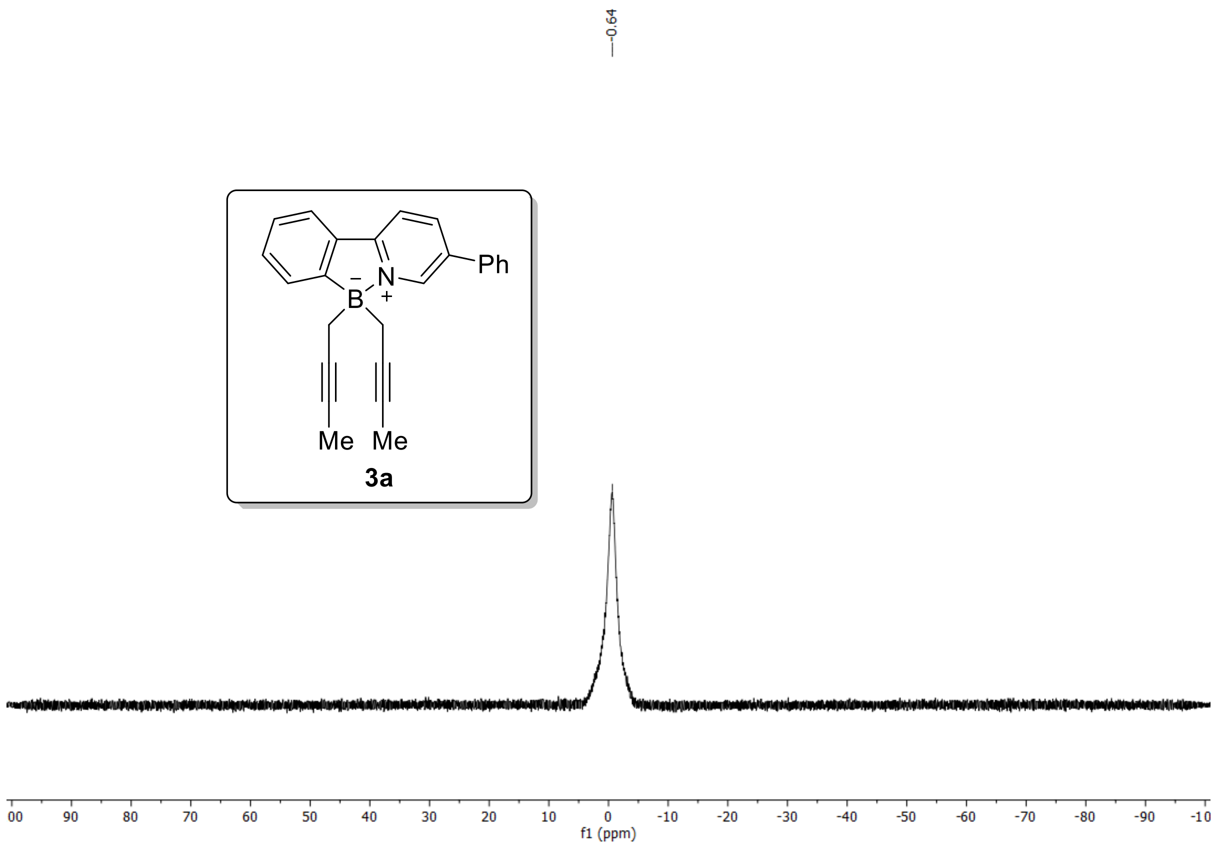
0.64
Ph
B
N
Me Me
3a
f1 (ppm)

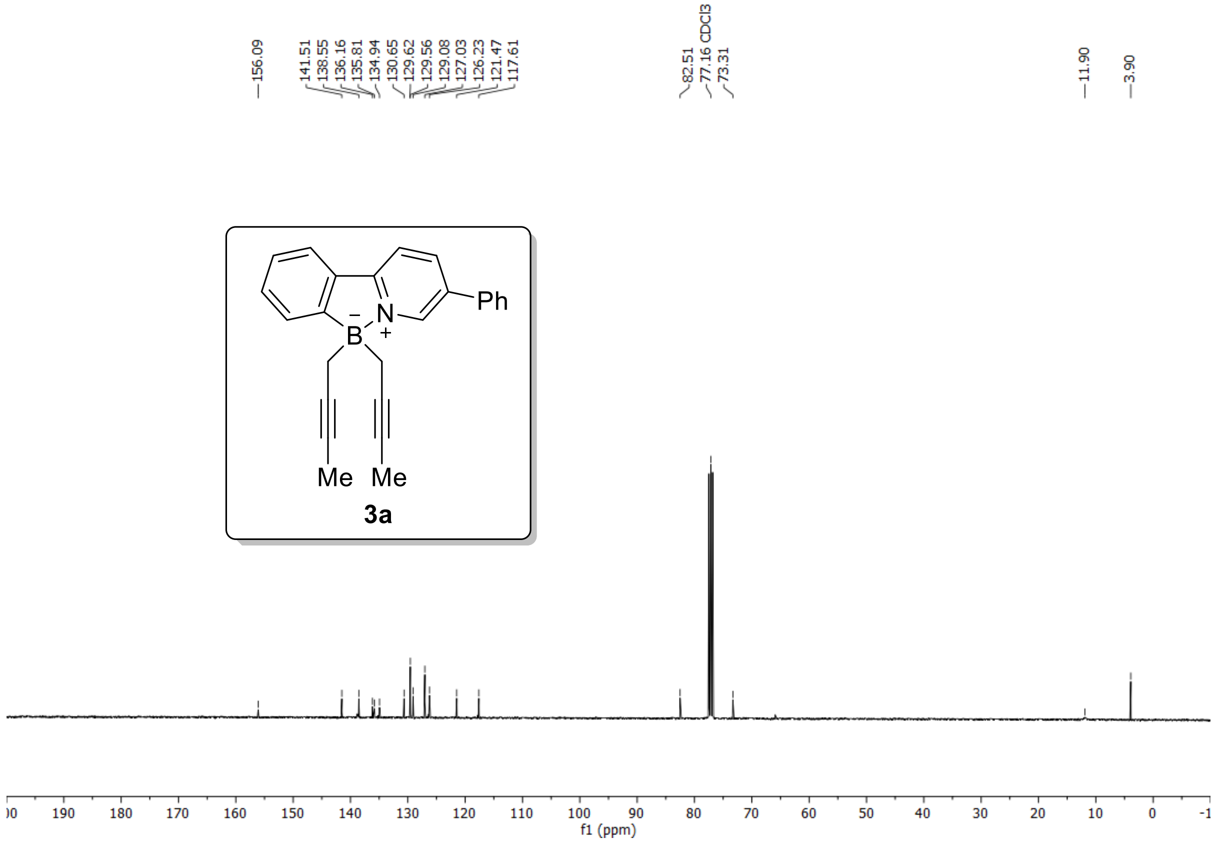
156.09
141.51
138.55
136.16
135.81
134.94
130.65
129.62
129.56
129.08
127.03
126.23
121.47
117.61
82.51
77.16 CDCl3
73.31
11.90
3.90
Ph
B
N
Me Me
3a
f1 (ppm)

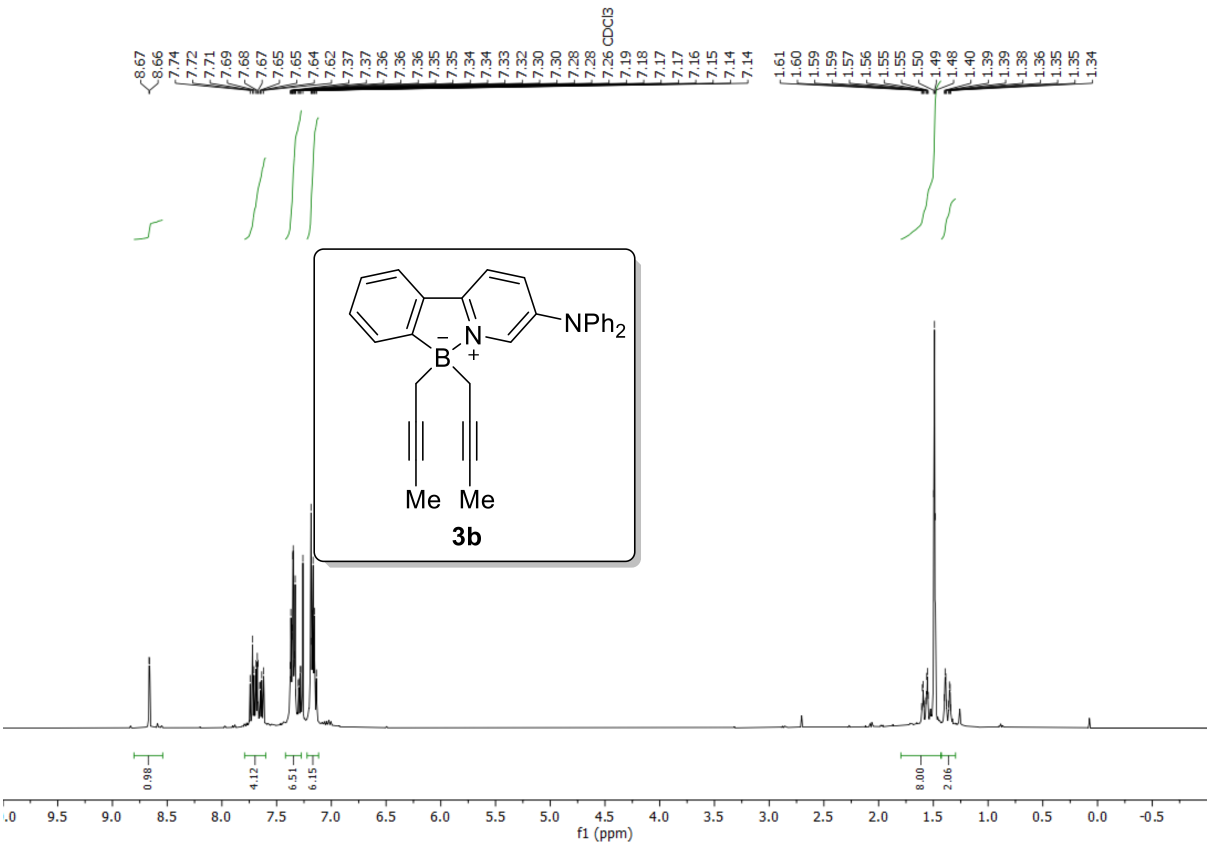
8.67
8.66
7.74
7.72
7.71
7.69
7.68
7.67
7.65
7.65
7.64
7.62
7.37
7.37
7.36
7.36
7.35
7.35
7.34
7.34
7.33
7.32
7.30
7.28
7.28
7.26 CDCl3
7.19
7.18
7.17
7.17
7.16
7.15
7.14
7.14
1.61
1.60
1.59
1.59
1.57
1.56
1.55
1.55
1.50
1.49
1.48
1.40
1.39
1.39
1.38
1.36
1.35
1.35
1.34
NPh2
B
N
Me Me
3b
0.98
4.12
6.51
6.15
8.00
2.06
f1 (ppm)

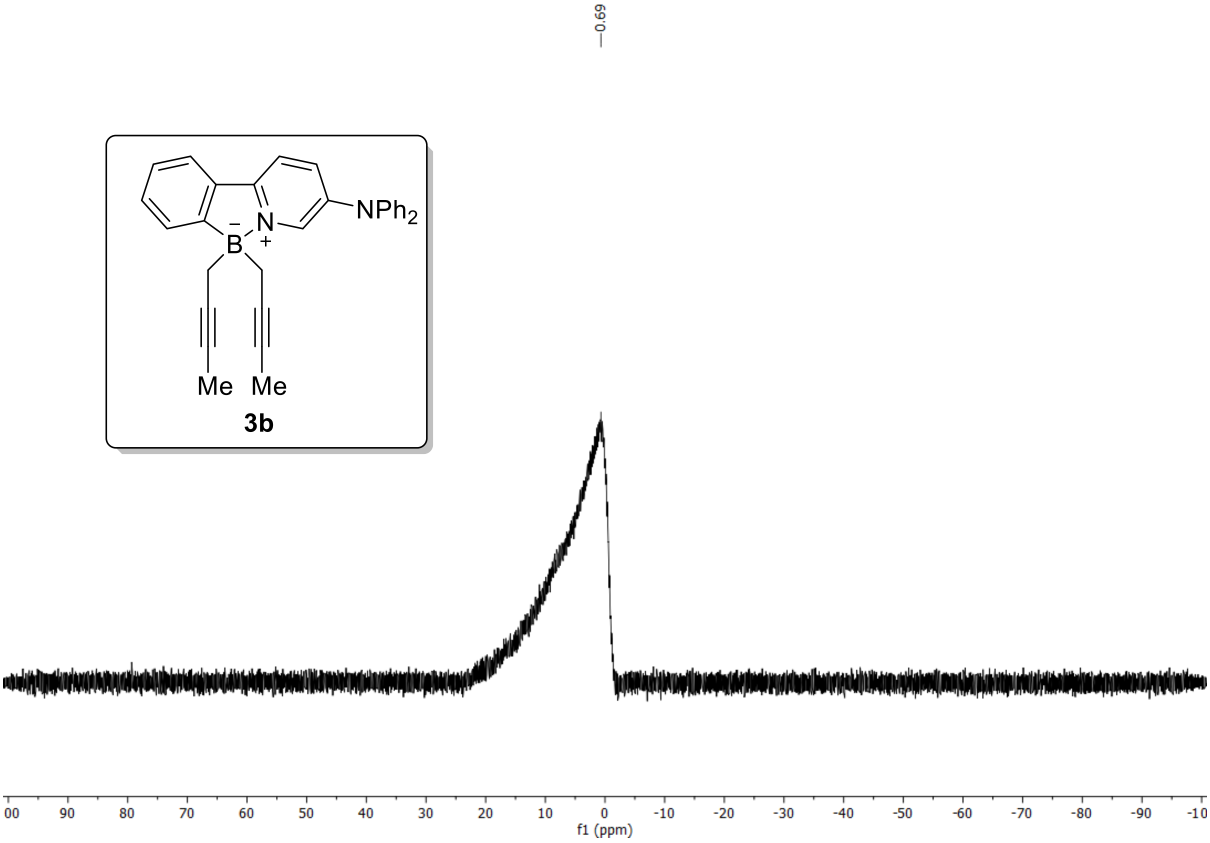
-0.69
NPh2
B
N
Me Me
3b
f1 (ppm)

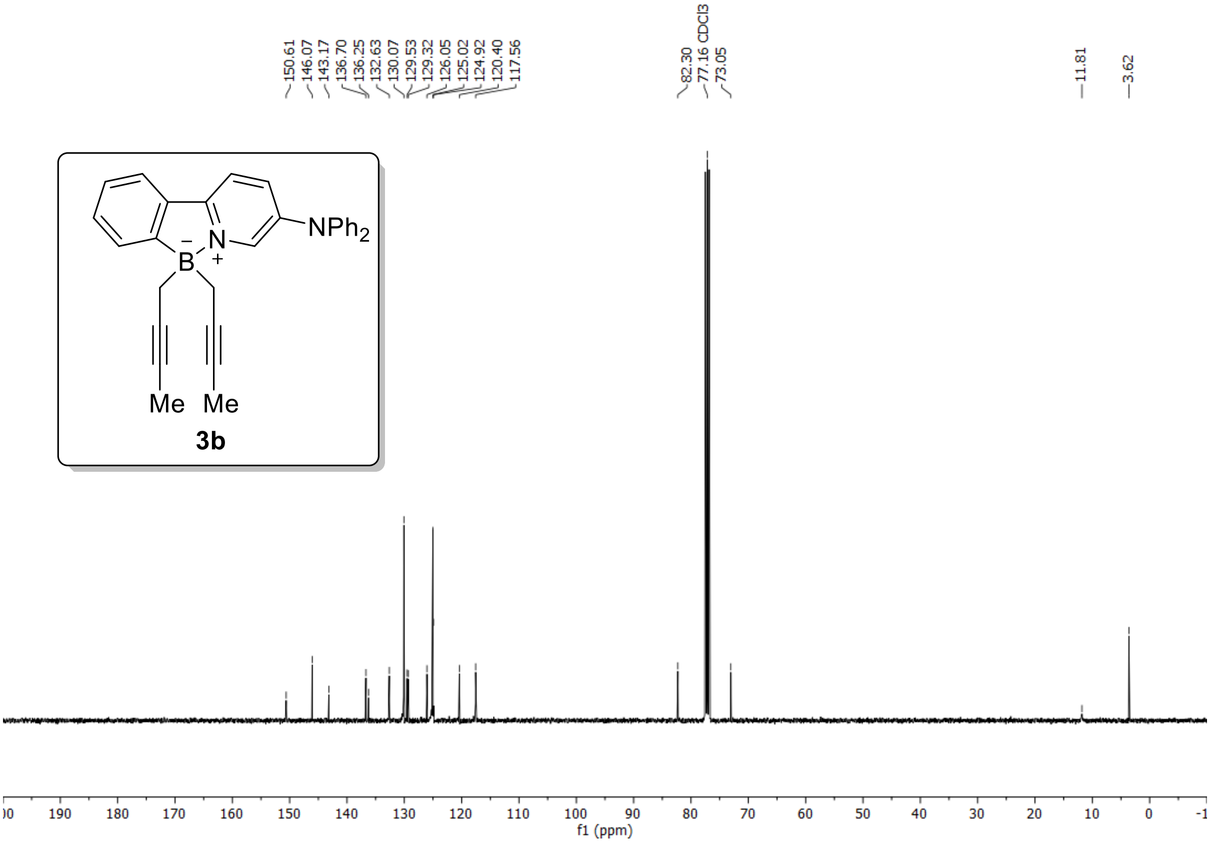
150.61
146.07
143.17
136.70
136.25
132.63
130.07
129.53
129.32
126.05
125.02
124.92
120.40
117.56
82.30
77.16 CDCl3
73.05
11.81
3.62
NPh2
B
N
Me Me
3b
f1 (ppm)

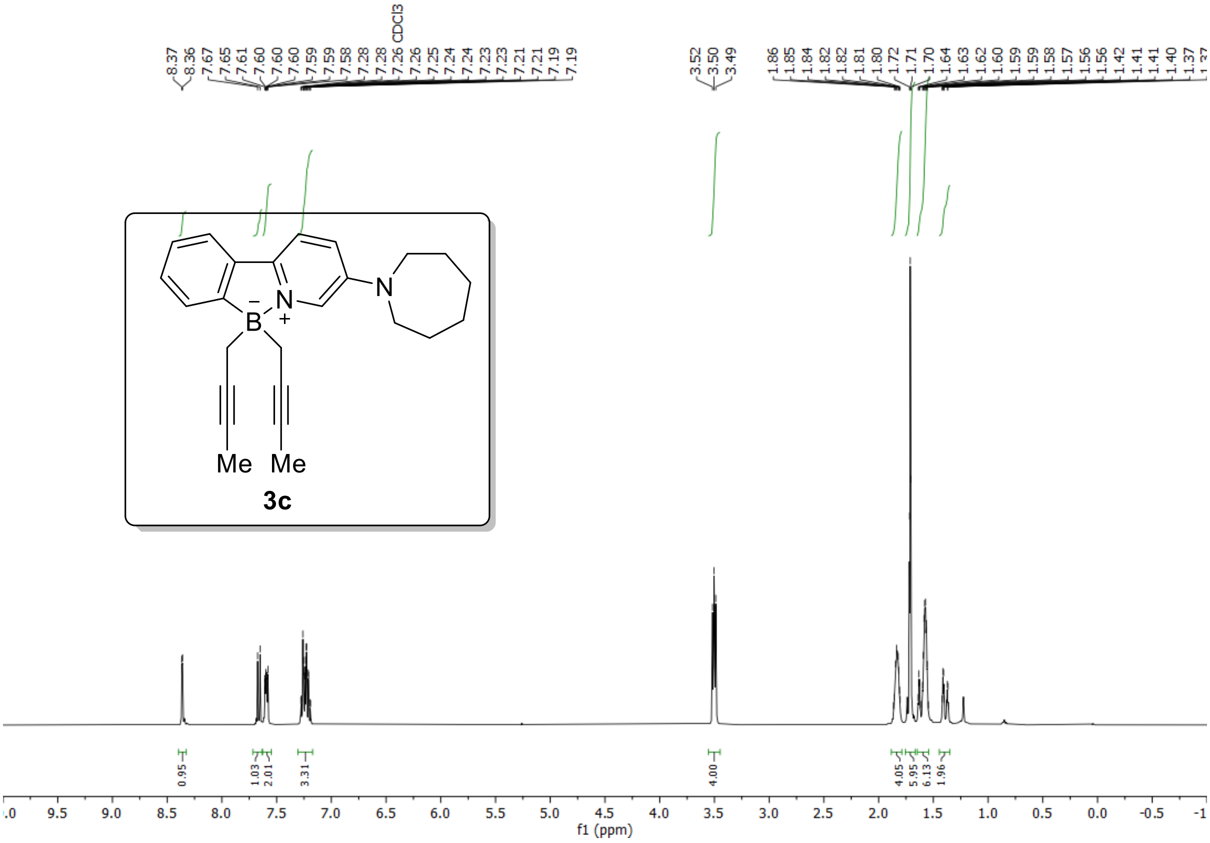
8.37
8.36
7.67
7.65
7.61
7.60
7.60
7.60
7.59
7.59
7.58
7.28
7.26 CDCl3
7.26
7.25
7.24
7.24
7.23
7.23
7.21
7.21
7.19
7.19
3.52
3.50
3.49
1.86
1.85
1.84
1.82
1.82
1.82
1.81
1.80
1.72
1.71
1.70
1.64
1.63
1.62
1.60
1.59
1.59
1.58
1.57
1.56
1.56
1.42
1.41
1.41
1.40
1.37
0.95
1.03
2.01
3.31
4.00
4.05
5.95
6.13
1.96
B
N
N
Me Me
3c
f1 (ppm)

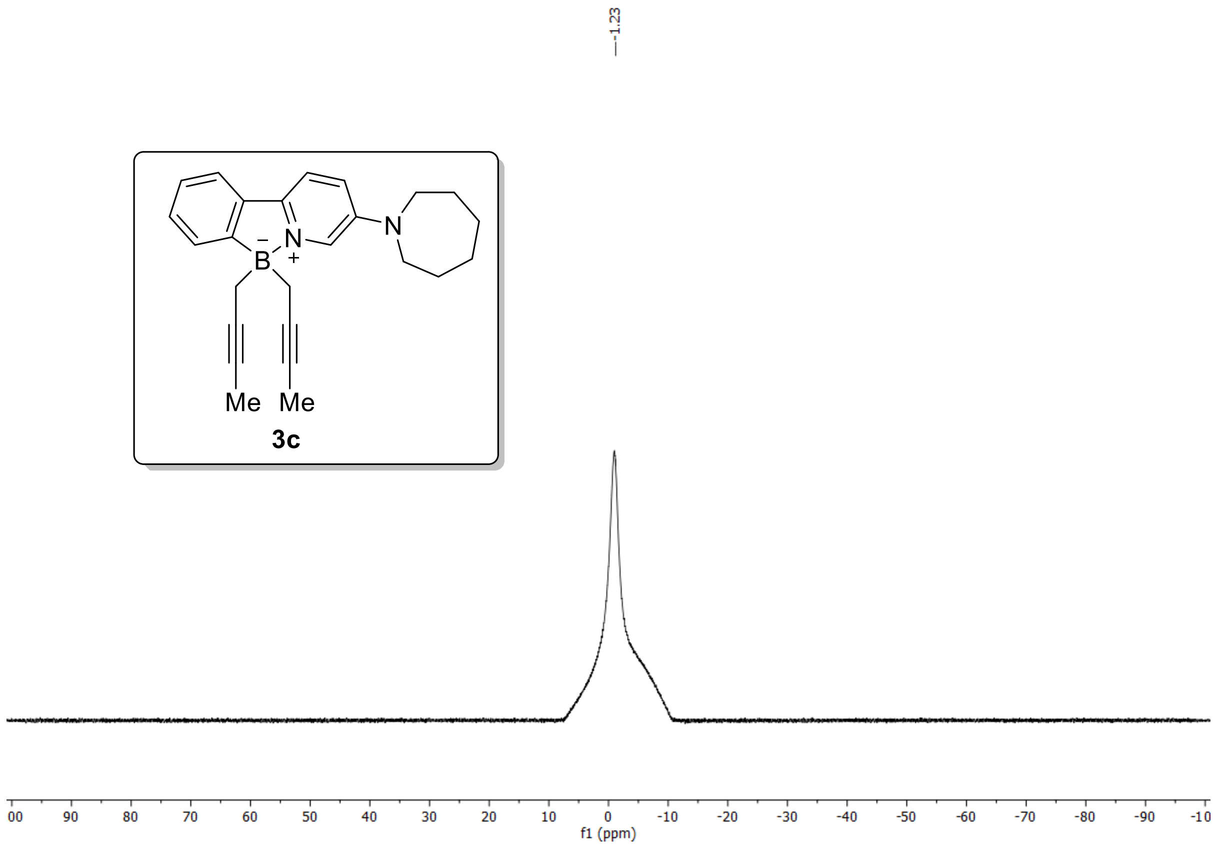
-1.23
Me Me
3c
f1 (ppm)

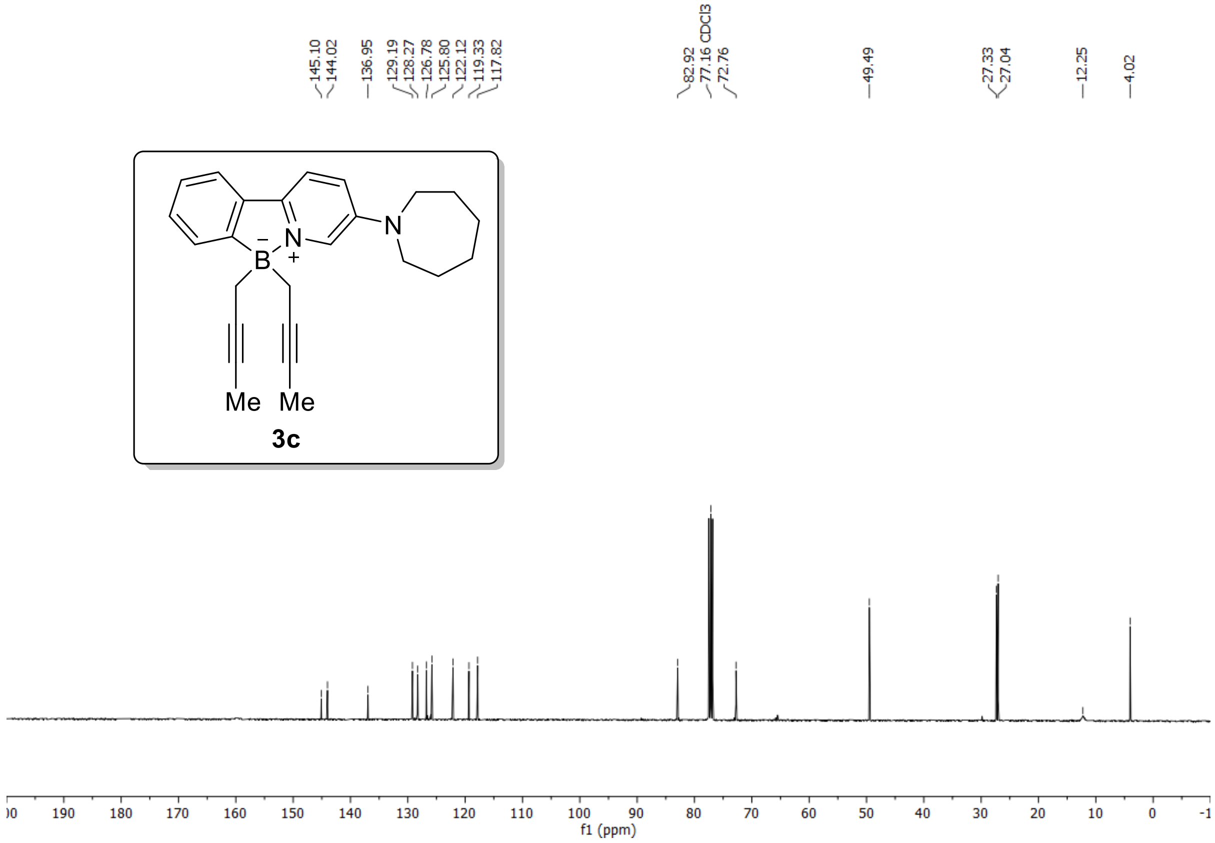
145.10
144.02
136.95
129.19
128.27
126.78
125.80
122.12
119.33
117.82
82.92
77.16 CDCl3
72.76
49.49
27.33
27.04
12.25
4.02
Me Me
3c
f1 (ppm)

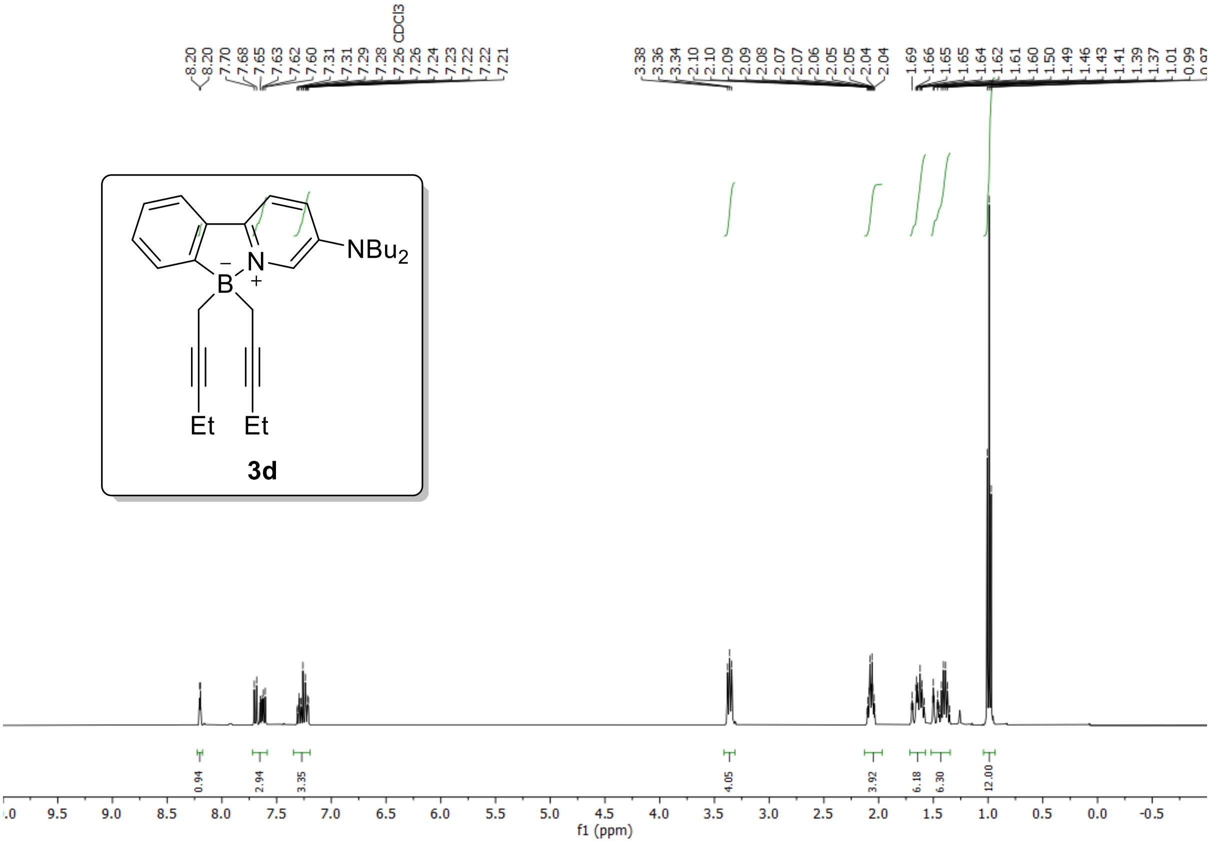
NBu2
Et Et
3d
f1 (ppm)

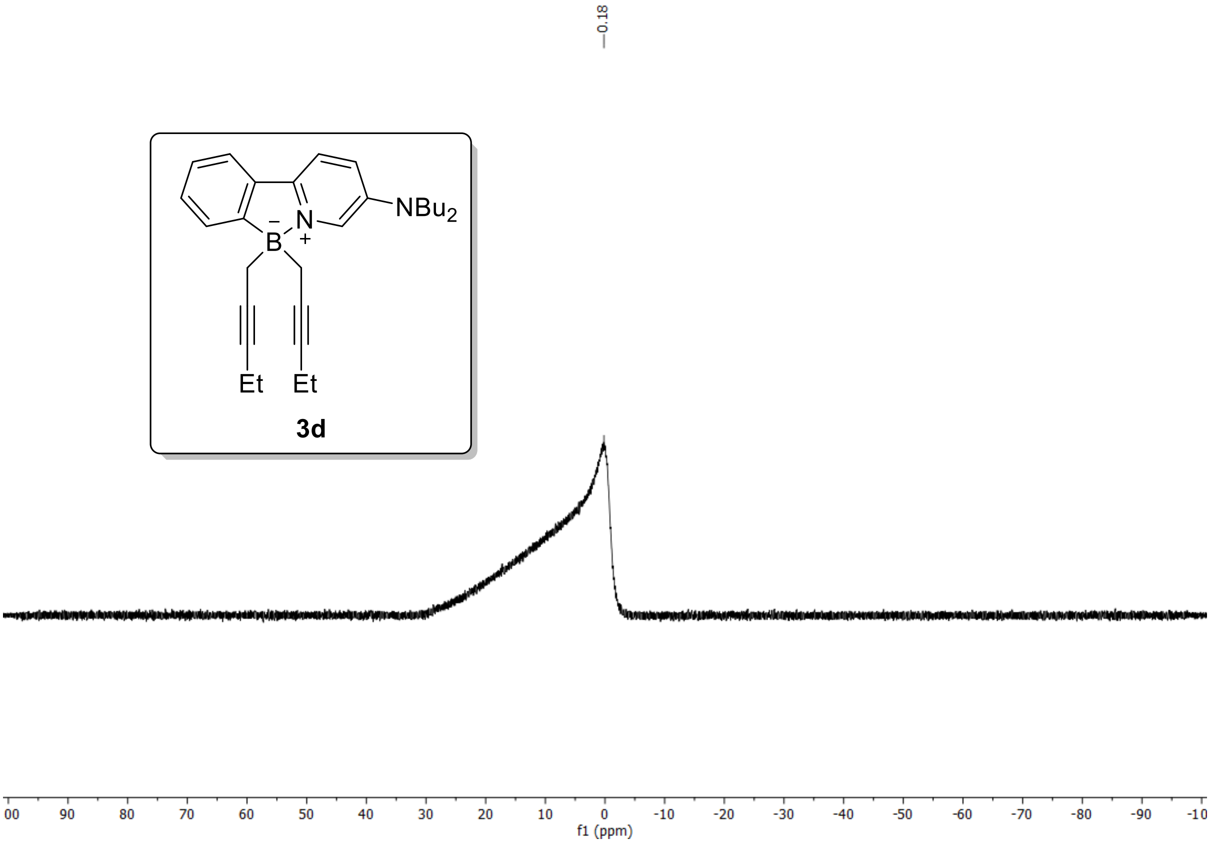
-0.18
NBu2
Et Et
3d
f1 (ppm)

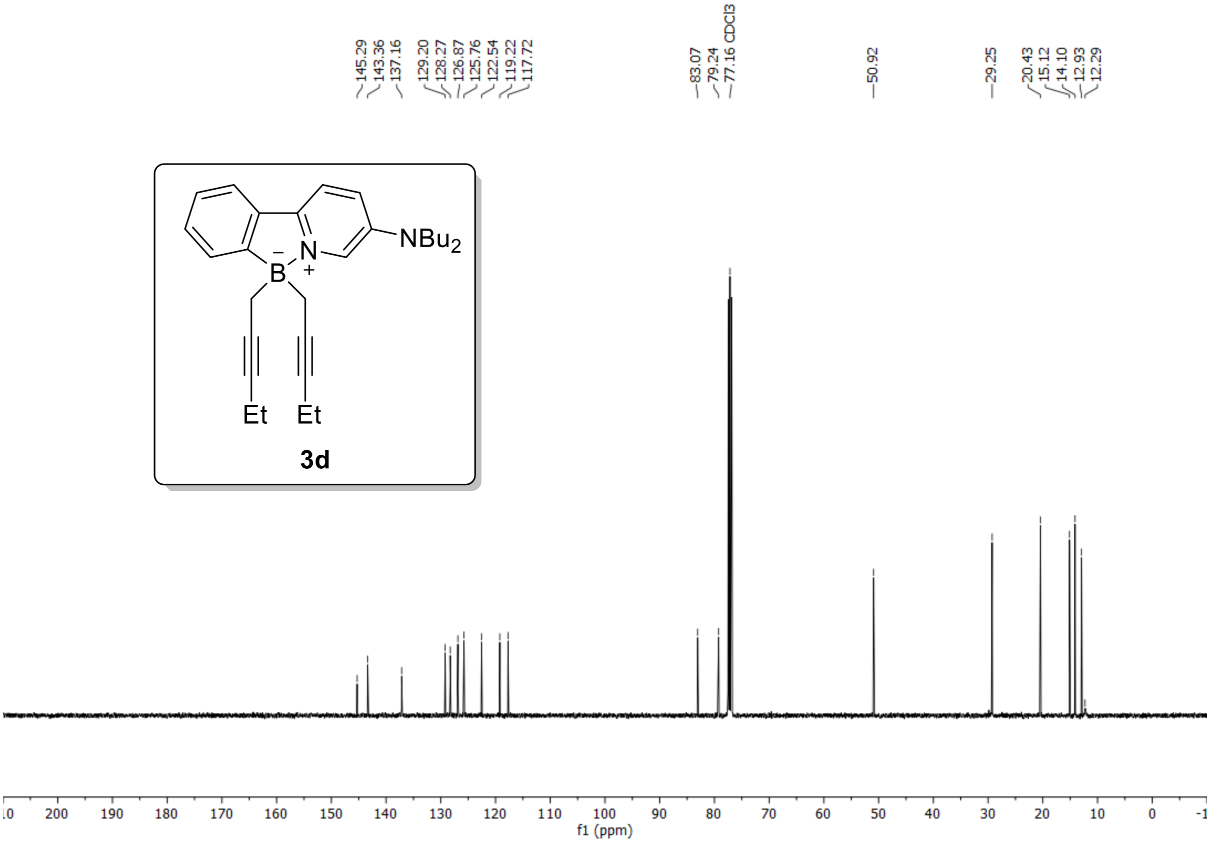
NBu2
3d
f1 (ppm)

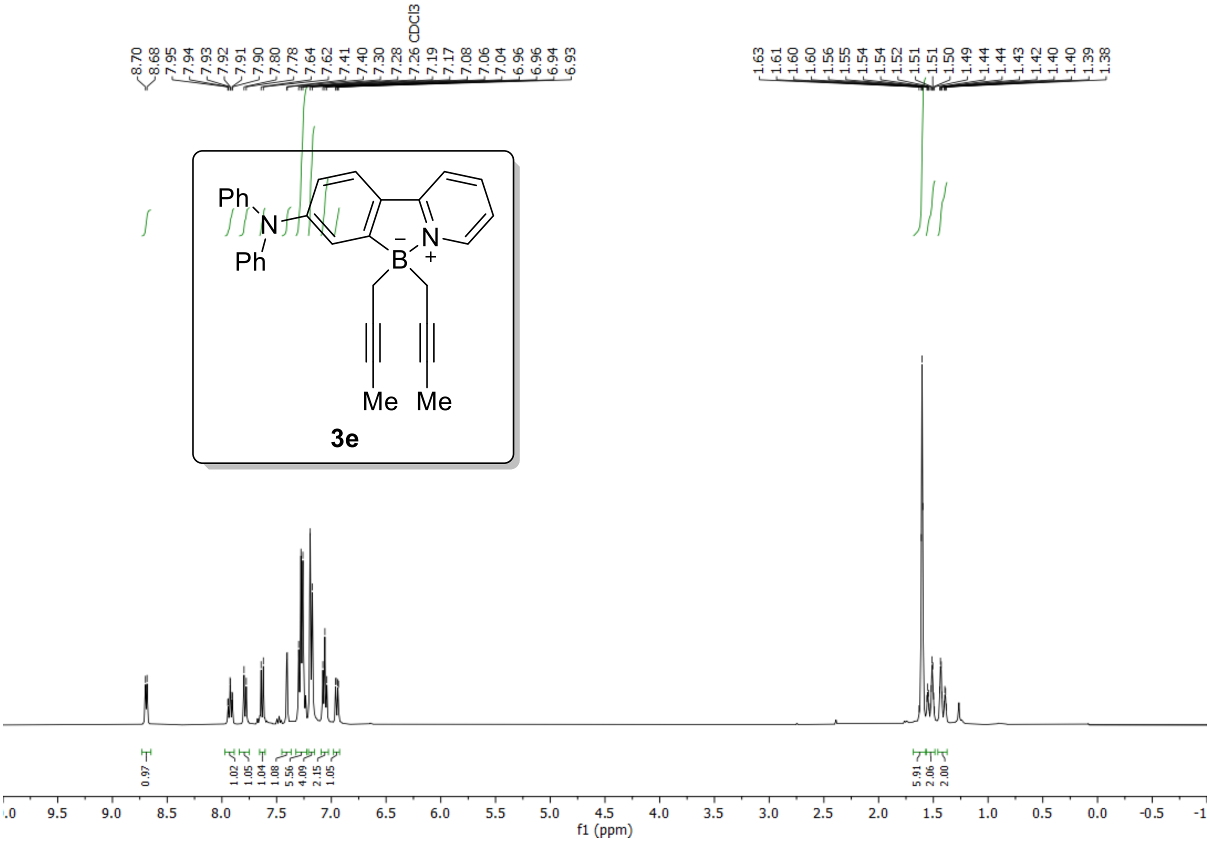
Ph
N
Ph
Me Me
3e
f1 (ppm)

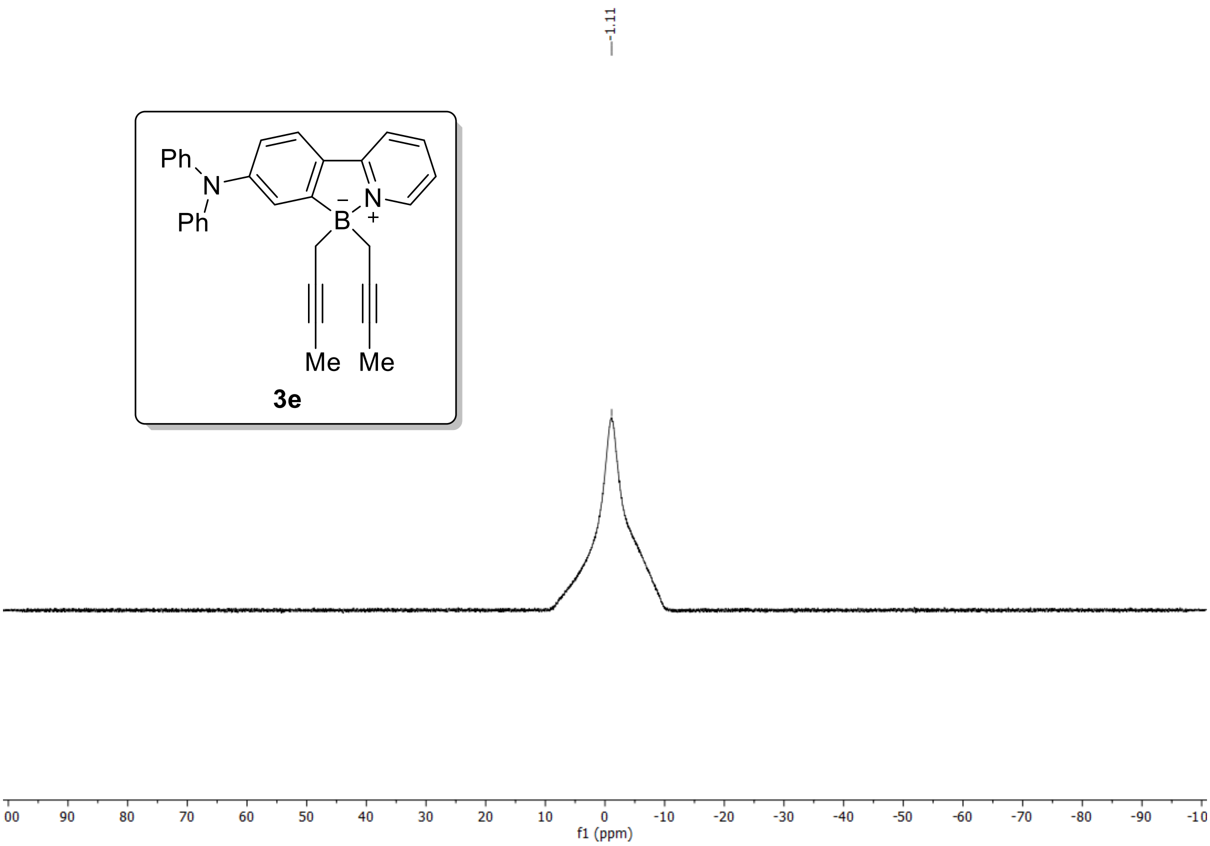
-1.11
Ph
N
Ph
B
N
Me Me
3e
f1 (ppm)

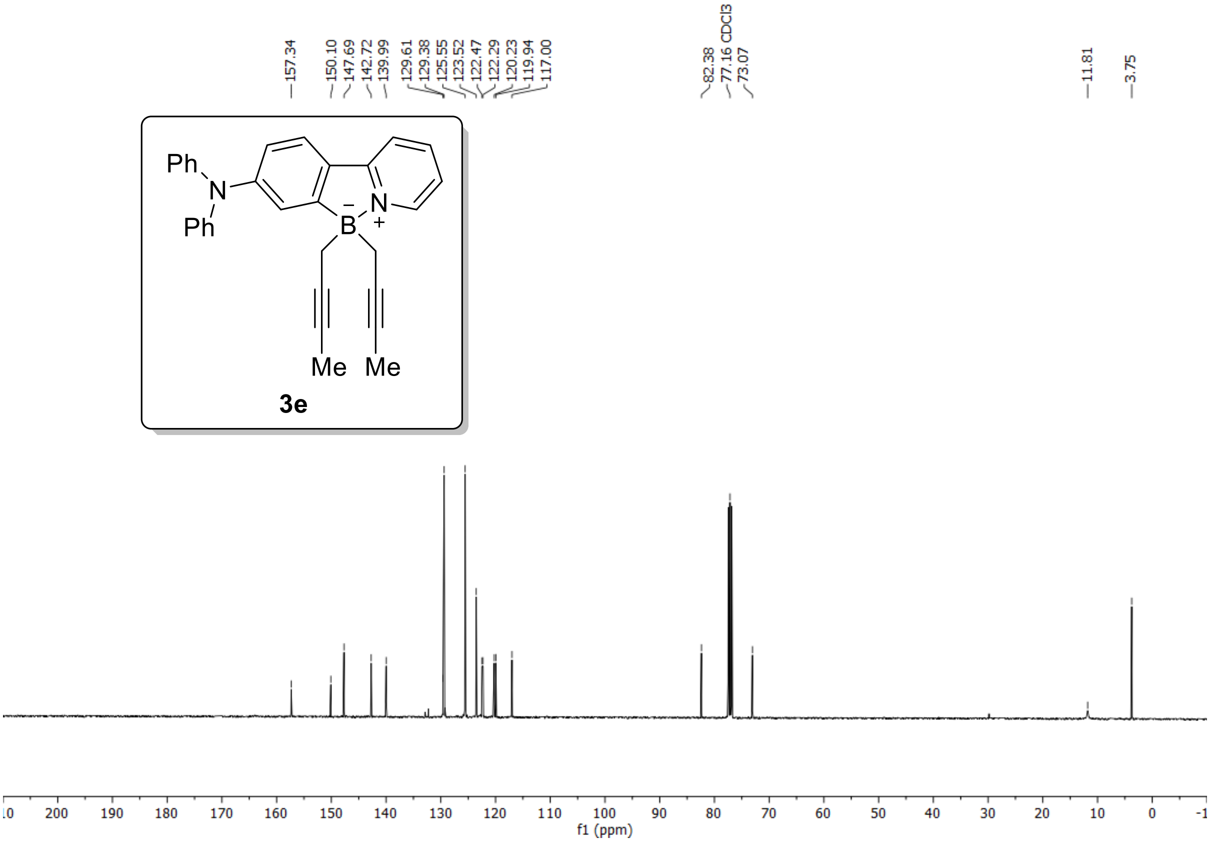
157.34
150.10
147.69
142.72
139.99
129.61
129.38
125.55
123.52
122.47
122.29
120.23
119.94
117.00
82.38
77.16 CDCl3
73.07
11.81
3.75
Ph
N
Ph
B
N
Me Me
3e
f1 (ppm)

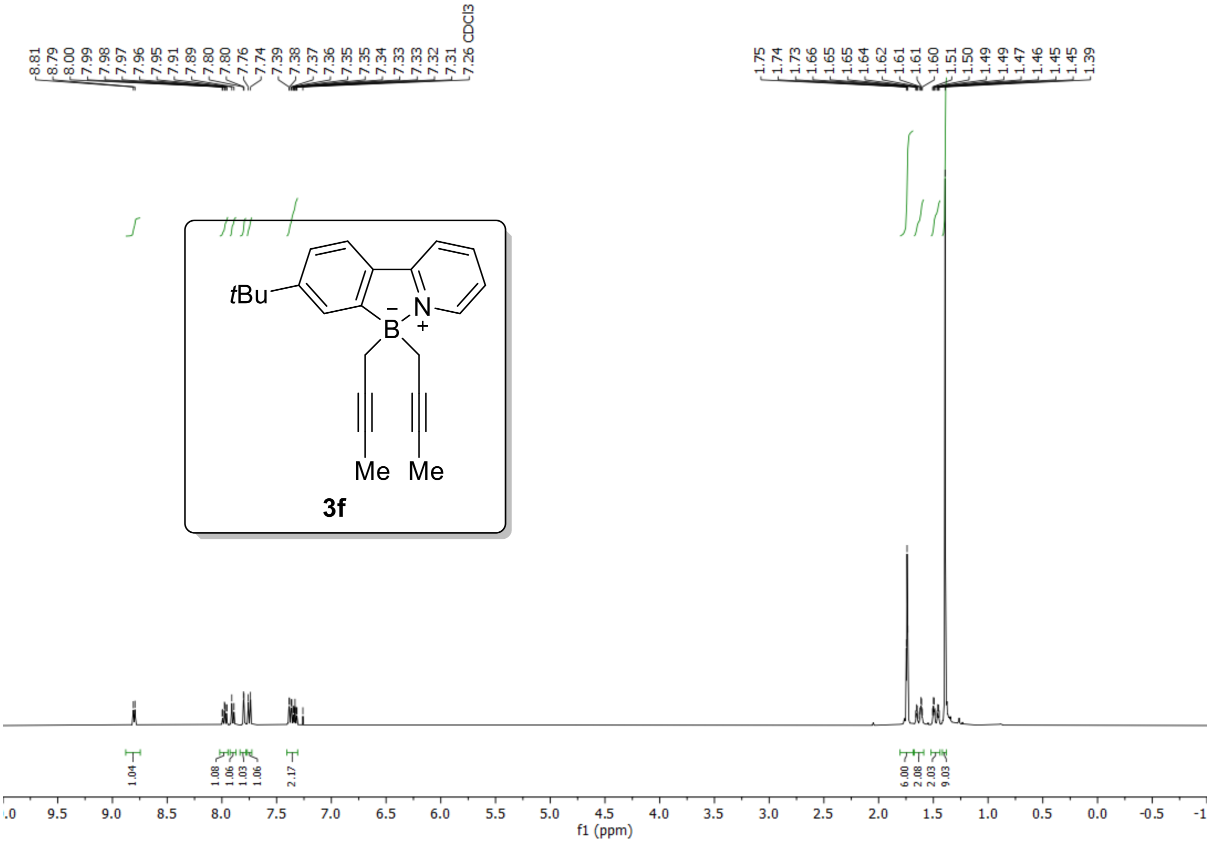
8.81
8.79
8.00
7.99
7.98
7.97
7.96
7.95
7.91
7.89
7.80
7.80
7.76
7.74
7.39
7.38
7.37
7.36
7.35
7.35
7.34
7.33
7.33
7.32
7.31
7.26 CDCl3
1.75
1.74
1.73
1.66
1.65
1.65
1.64
1.62
1.61
1.61
1.60
1.51
1.50
1.49
1.49
1.47
1.46
1.45
1.45
1.39
tBu
B
N
Me Me
3f
1.04
1.08
1.06
1.03
1.06
2.17
6.00
2.08
2.03
9.03
f1 (ppm)

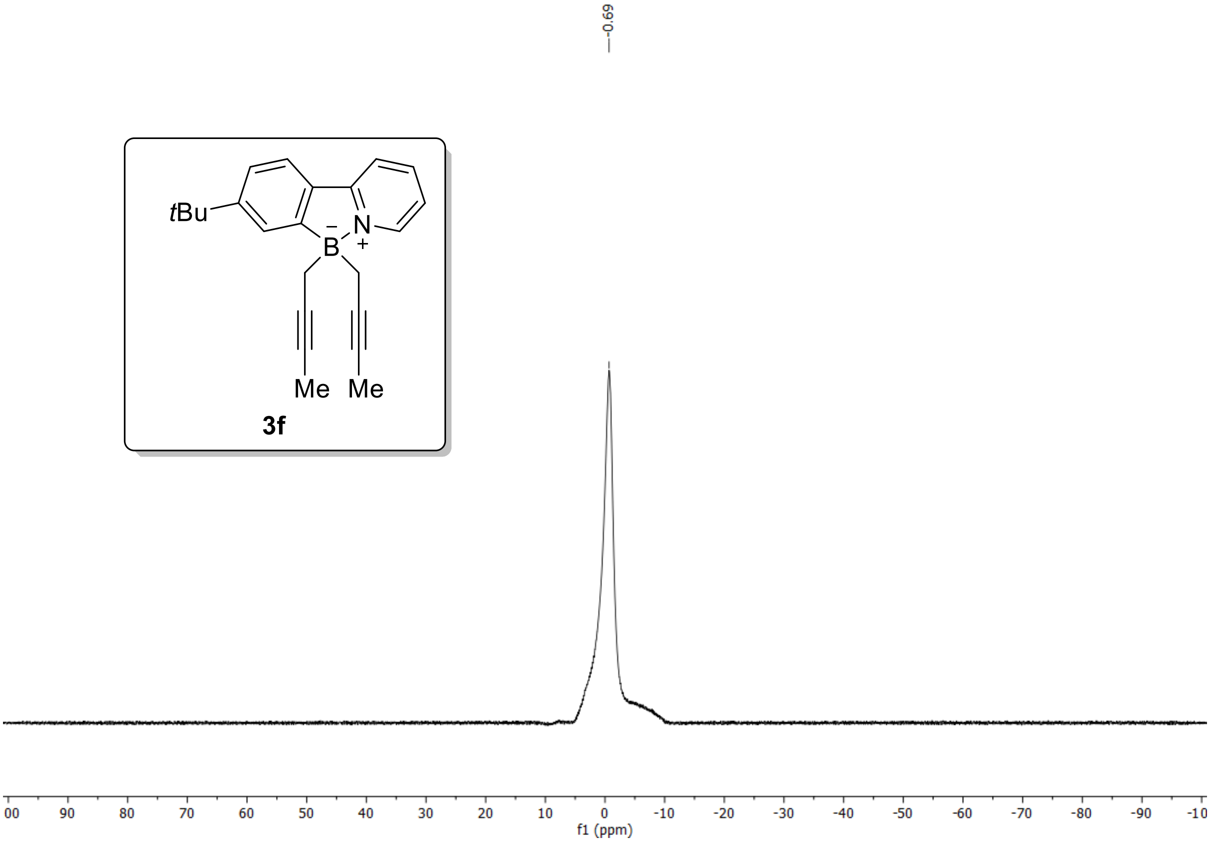
-0.69
tBu
B
N
Me Me
3f
f1 (ppm)

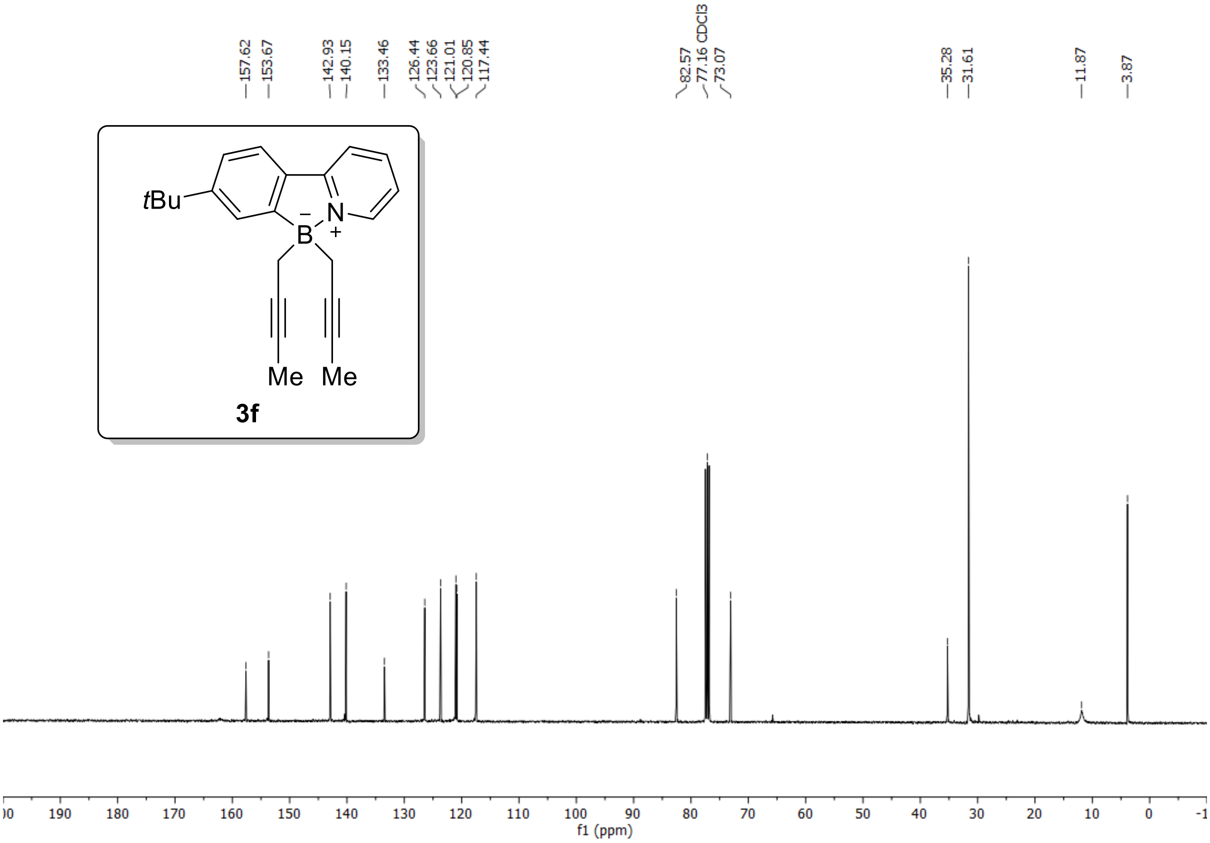

157.62
153.67
142.93
140.15
133.46
126.44
123.66
121.01
120.85
117.44
82.57
77.16 CDCl3
73.07
35.28
31.61
11.87
3.87
tBu
B
N
Me Me
3f
f1 (ppm)


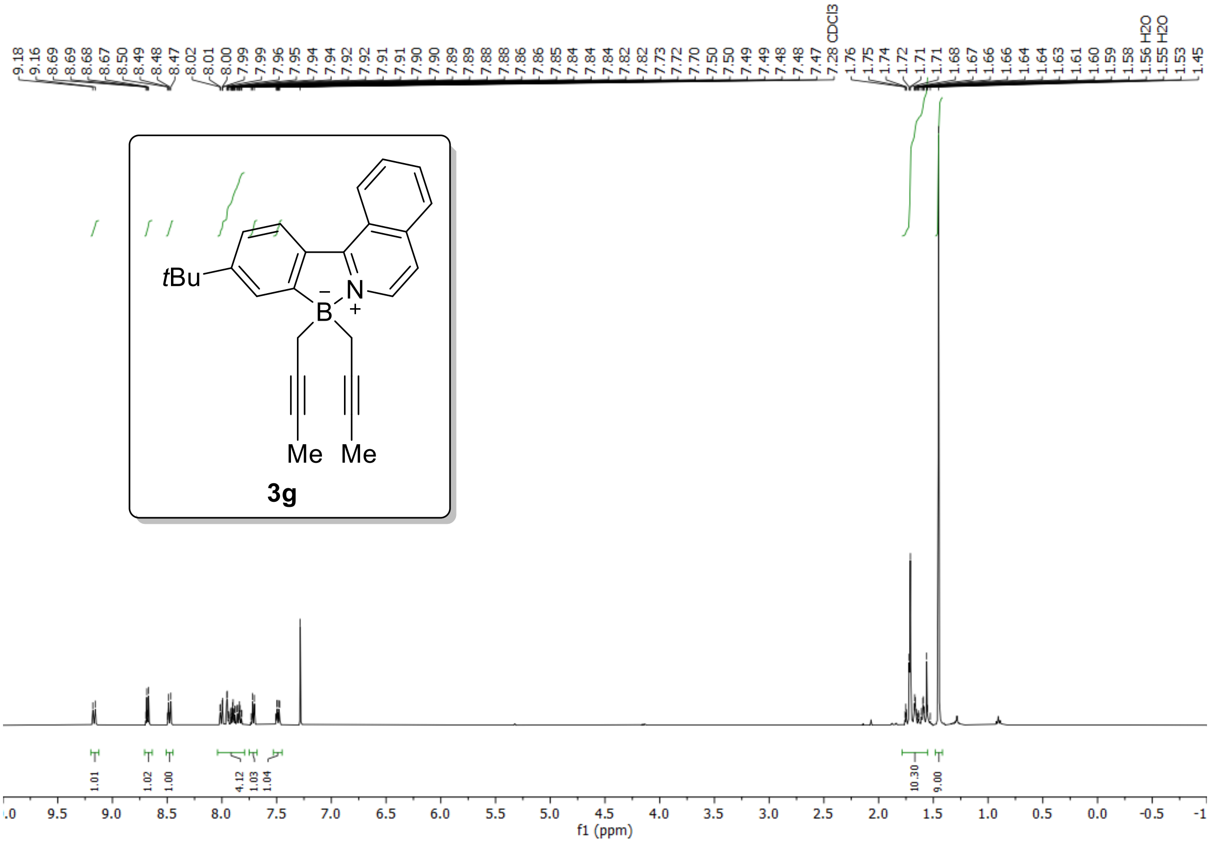

9.18
9.16
8.69
8.69
8.68
8.67
8.50
8.49
8.48
8.47
8.02
8.01
8.00
7.99
7.99
7.96
7.95
7.94
7.94
7.92
7.92
7.91
7.91
7.90
7.90
7.89
7.89
7.88
7.88
7.86
7.86
7.85
7.84
7.84
7.84
7.82
7.82
7.73
7.72
7.70
7.50
7.50
7.49
7.49
7.48
7.48
7.47
7.28 CDCl3
1.76
1.75
1.74
1.72
1.71
1.71
1.68
1.67
1.66
1.66
1.64
1.64
1.63
1.61
1.60
1.59
1.58
1.56 H2O
1.55 H2O
1.53
1.45
tBu
B
N
Me Me
3g
1.01
1.02
1.00
4.12
1.03
1.04
10.30
9.00
f1 (ppm)

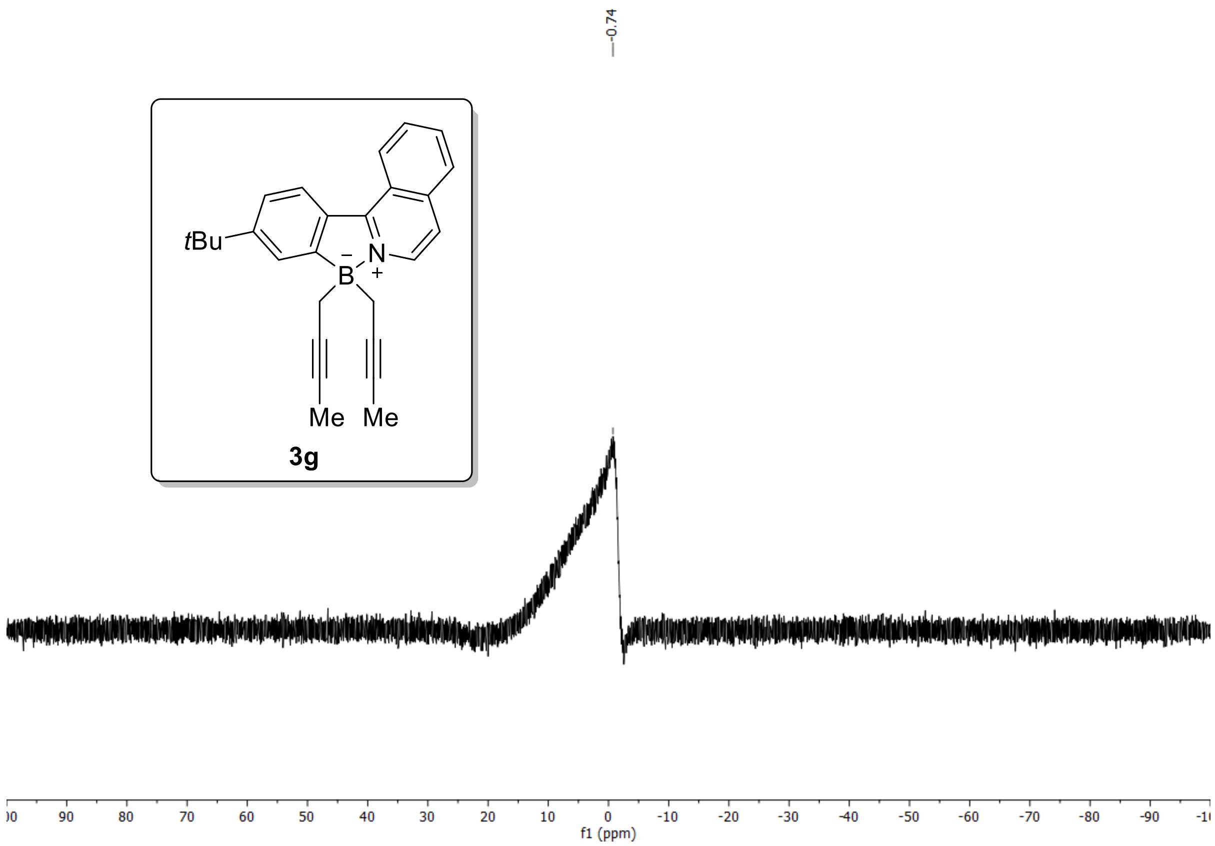

0.74
tBu
B
N
Me Me
3g
f1 (ppm)


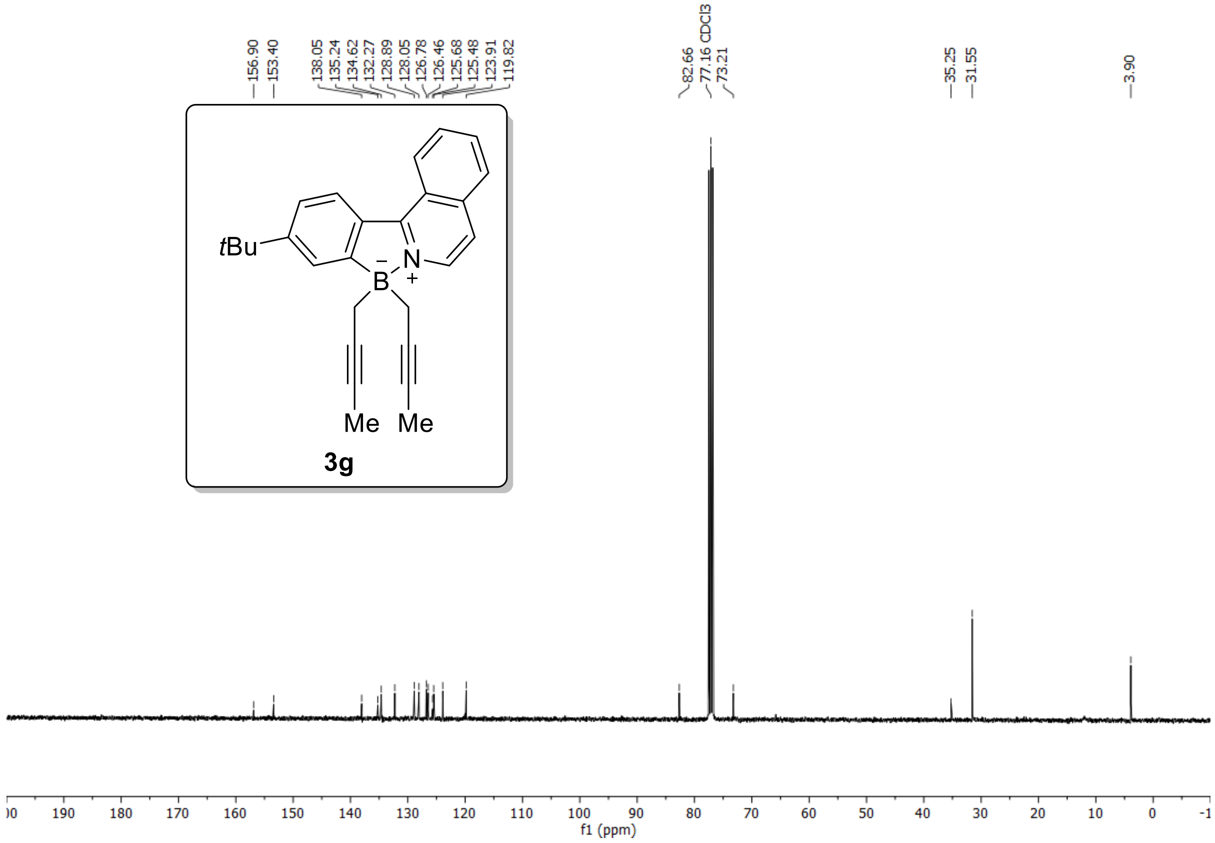

156.90
153.40
138.05
135.24
134.62
132.27
128.89
128.05
126.78
126.46
125.68
125.48
123.91
119.82
82.66
77.16 CDCl3
73.21
35.25
31.55
3.90
tBu
B
N
Me Me
3g
f1 (ppm)

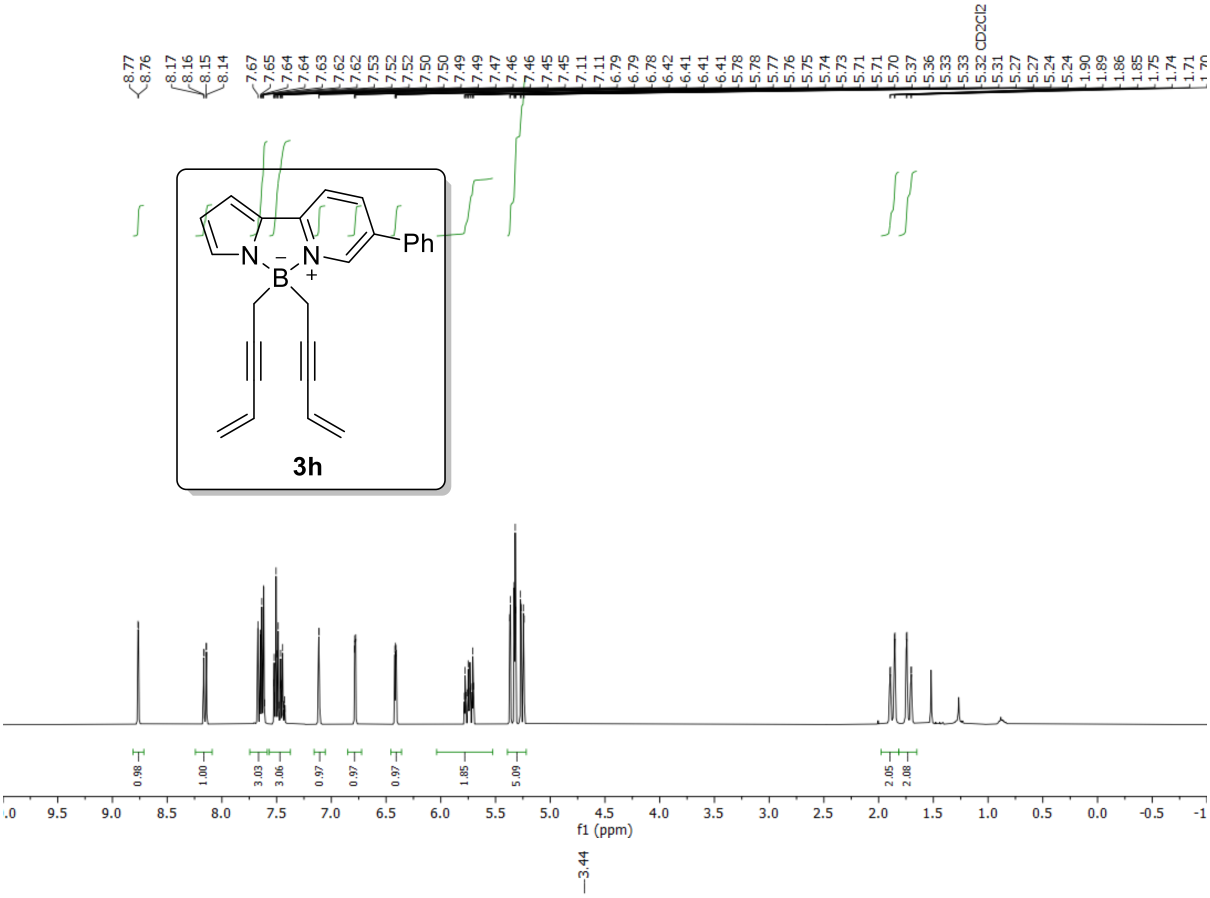

CD2Cl2
Ph
3h
f1 (ppm)


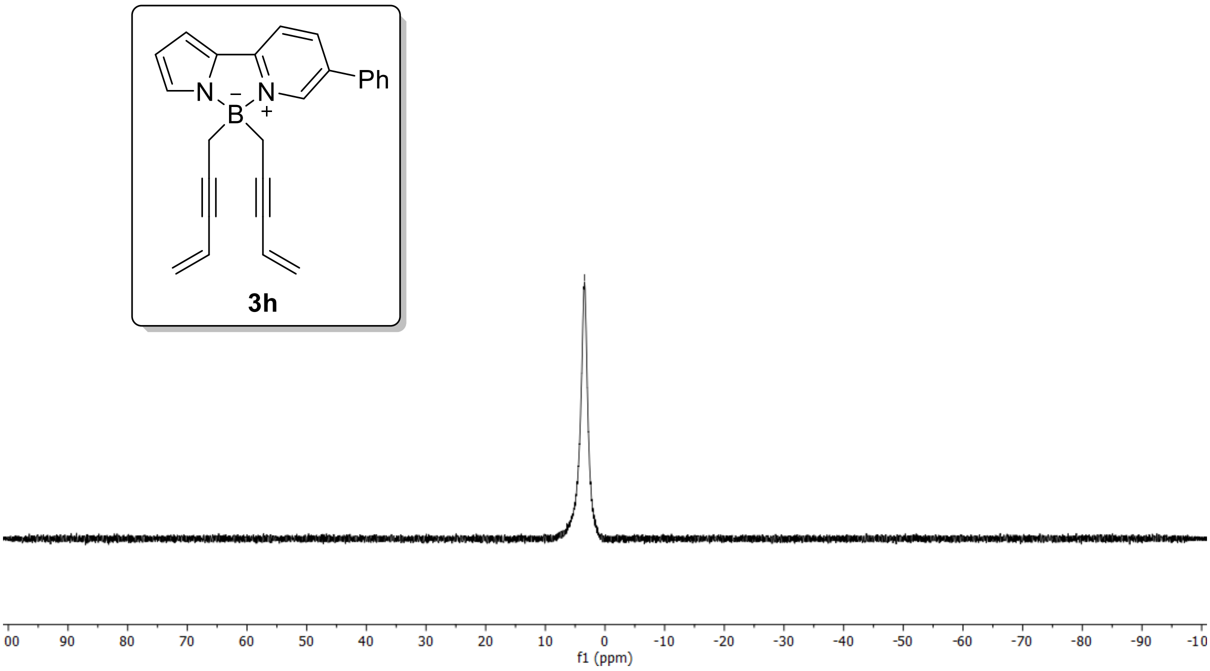

3.44
Ph
3h
f1 (ppm)

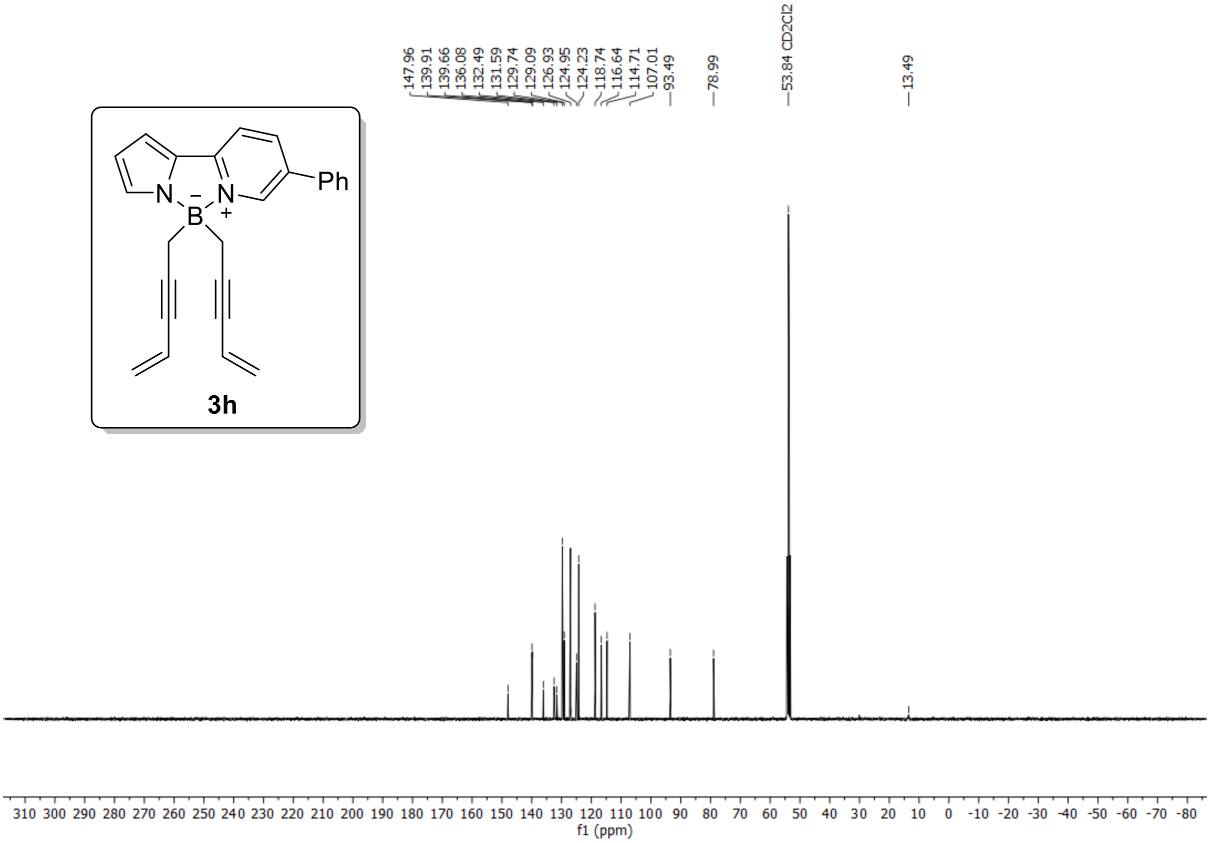
147.96
139.91
139.66
136.08
132.49
131.59
129.74
129.09
126.93
124.95
124.23
118.74
116.64
114.71
107.01
93.49
78.99
53.84 CD2Cl2
13.49
Ph
N
B
N
3h
f1 (ppm)

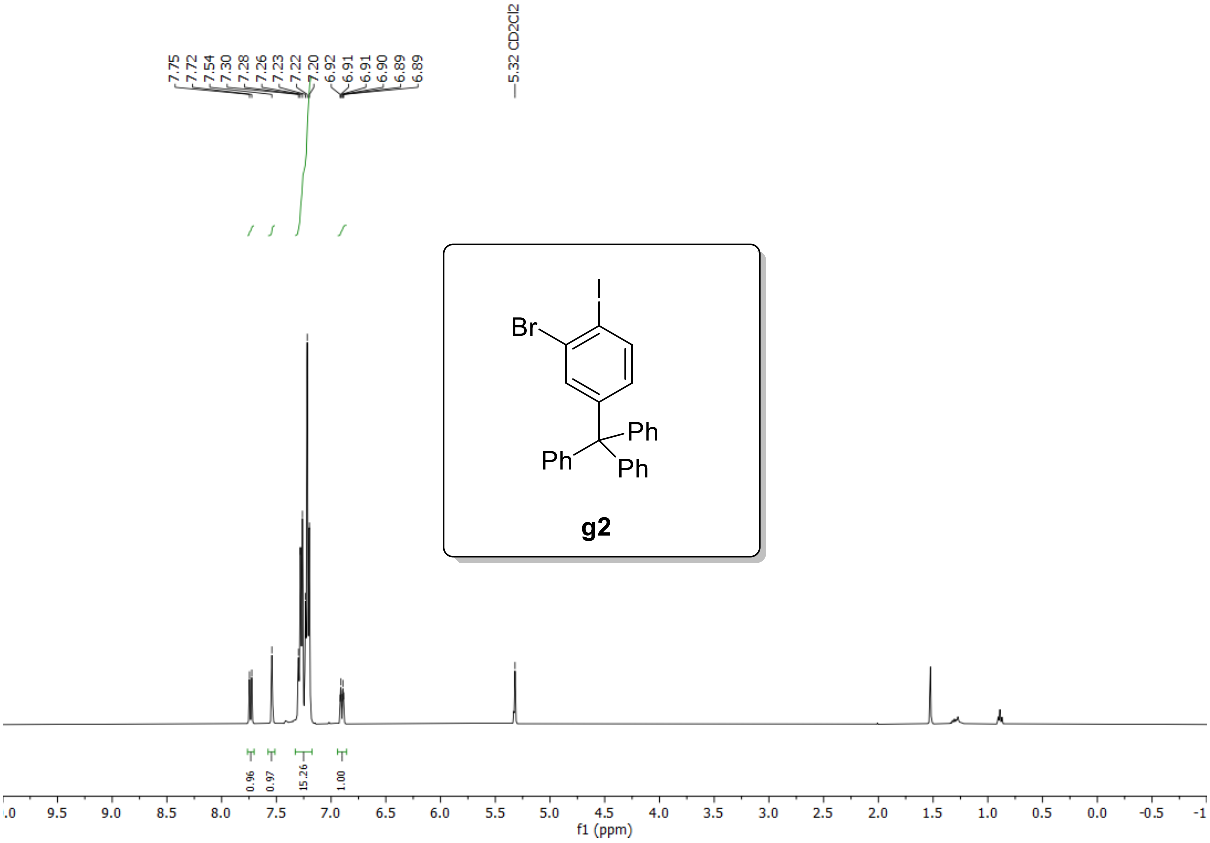
7.75
7.72
7.54
7.30
7.28
7.26
7.23
7.22
7.20
6.92
6.91
6.90
6.89
6.89
5.32 CD2Cl2
I
Br
Ph
Ph
Ph
g2
0.96
0.97
15.26
1.00
f1 (ppm)

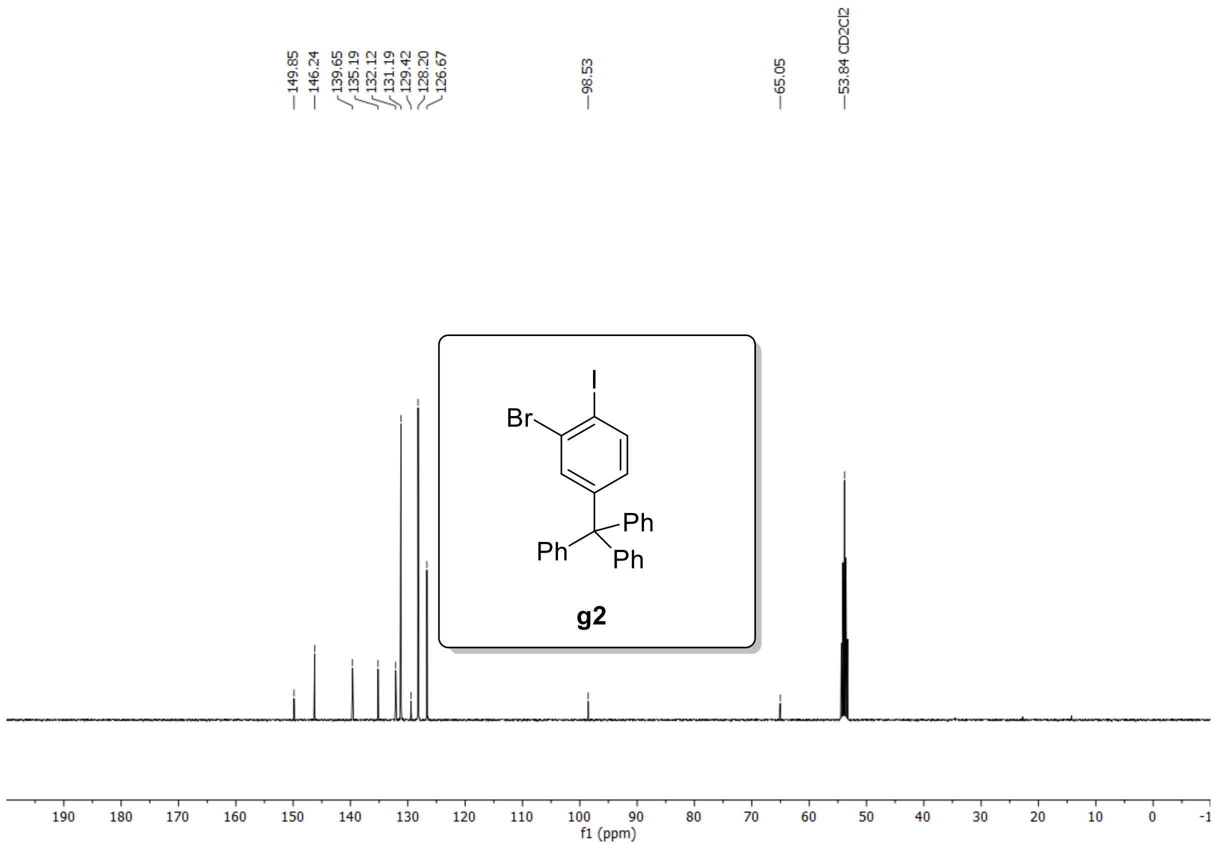
149.85
146.24
139.65
135.19
132.12
131.19
129.42
128.20
126.67
98.53
65.05
53.84 CD2Cl2
Br
I
Ph
Ph
Ph
g2
f1 (ppm)

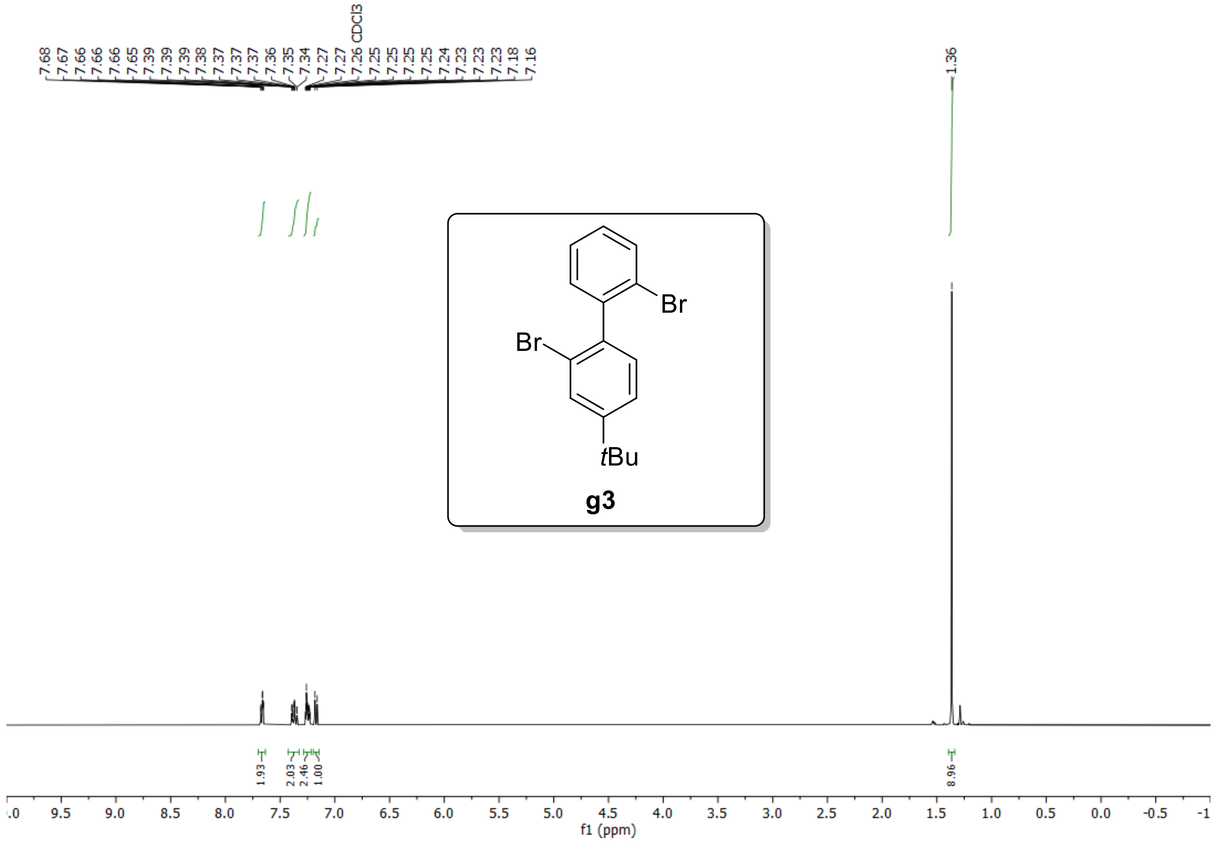
7.68
7.67
7.66
7.66
7.66
7.65
7.39
7.39
7.39
7.38
7.37
7.37
7.37
7.36
7.35
7.34
7.27
7.27
7.26 CDCl3
7.25
7.25
7.25
7.25
7.24
7.23
7.23
7.23
7.23
7.18
7.16
1.36
Br
Br
tBu
g3
1.93
2.03
2.46
1.00
8.96
f1 (ppm)

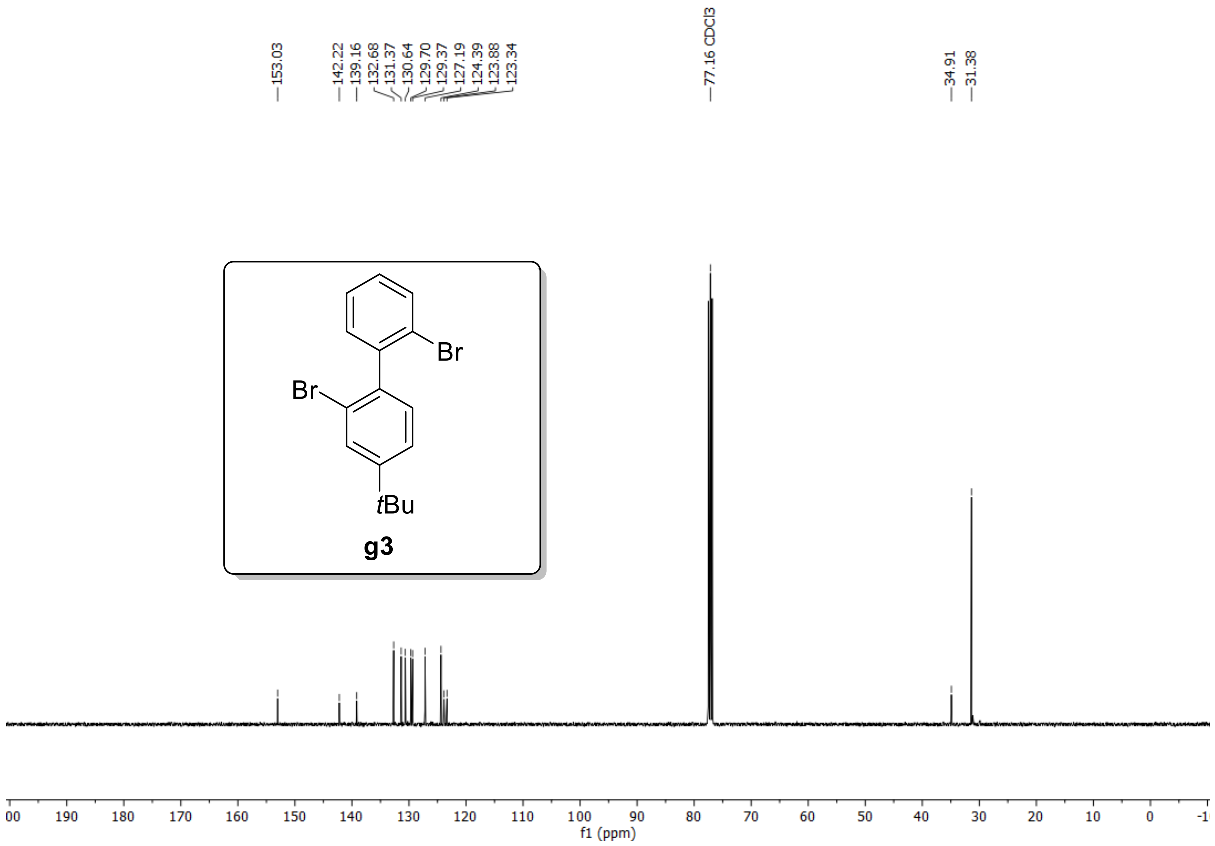
153.03
142.22
139.16
132.68
131.37
130.64
129.70
129.37
127.19
124.39
123.88
123.34
77.16 CDCl3
34.91
31.38
Br
Br
tBu
g3
f1 (ppm)

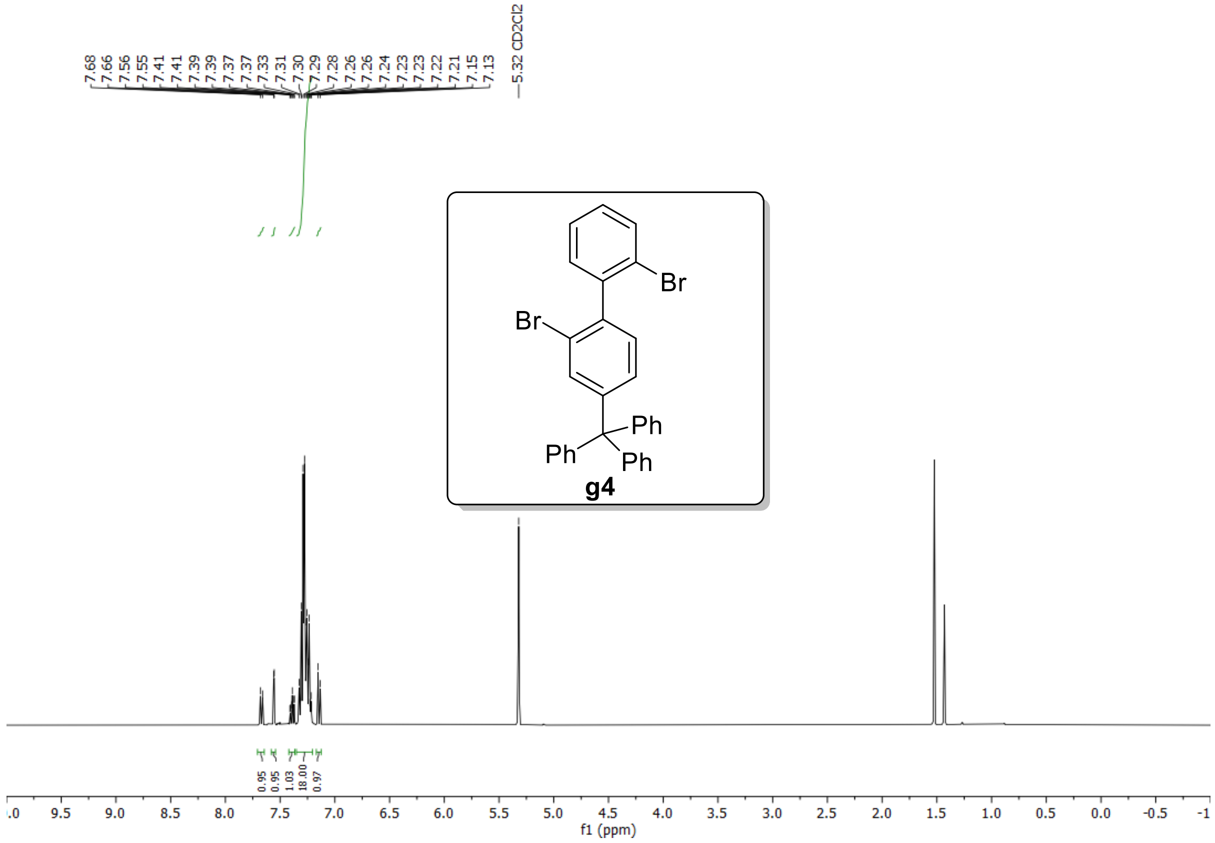
7.68
7.66
7.56
7.55
7.41
7.41
7.39
7.39
7.37
7.37
7.33
7.31
7.30
7.29
7.28
7.26
7.26
7.24
7.23
7.23
7.22
7.21
7.15
7.13
5.32 CD2Cl2
Br
Br
Ph
Ph
Ph
g4
0.95
0.95
1.03
18.00
0.97
f1 (ppm)

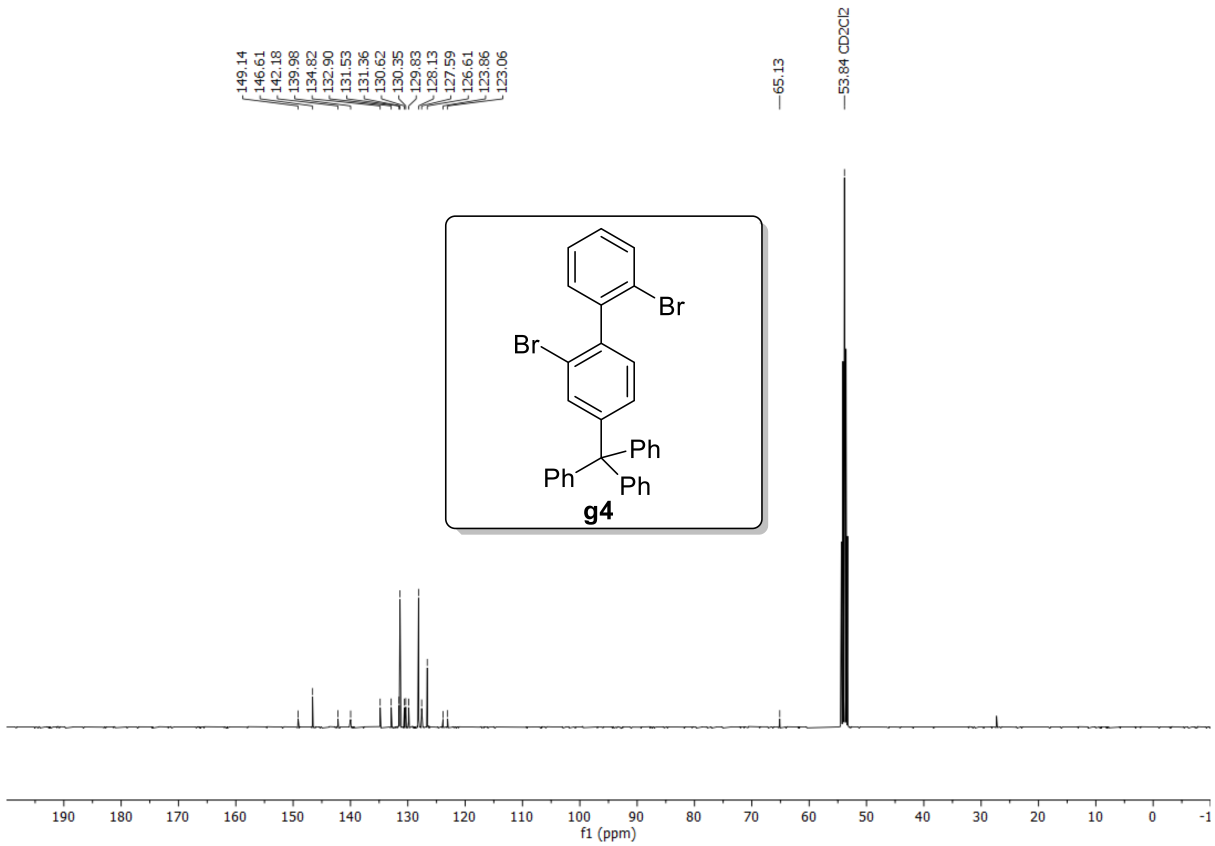
Br
Br
Ph
Ph
Ph
g4
f1 (ppm)

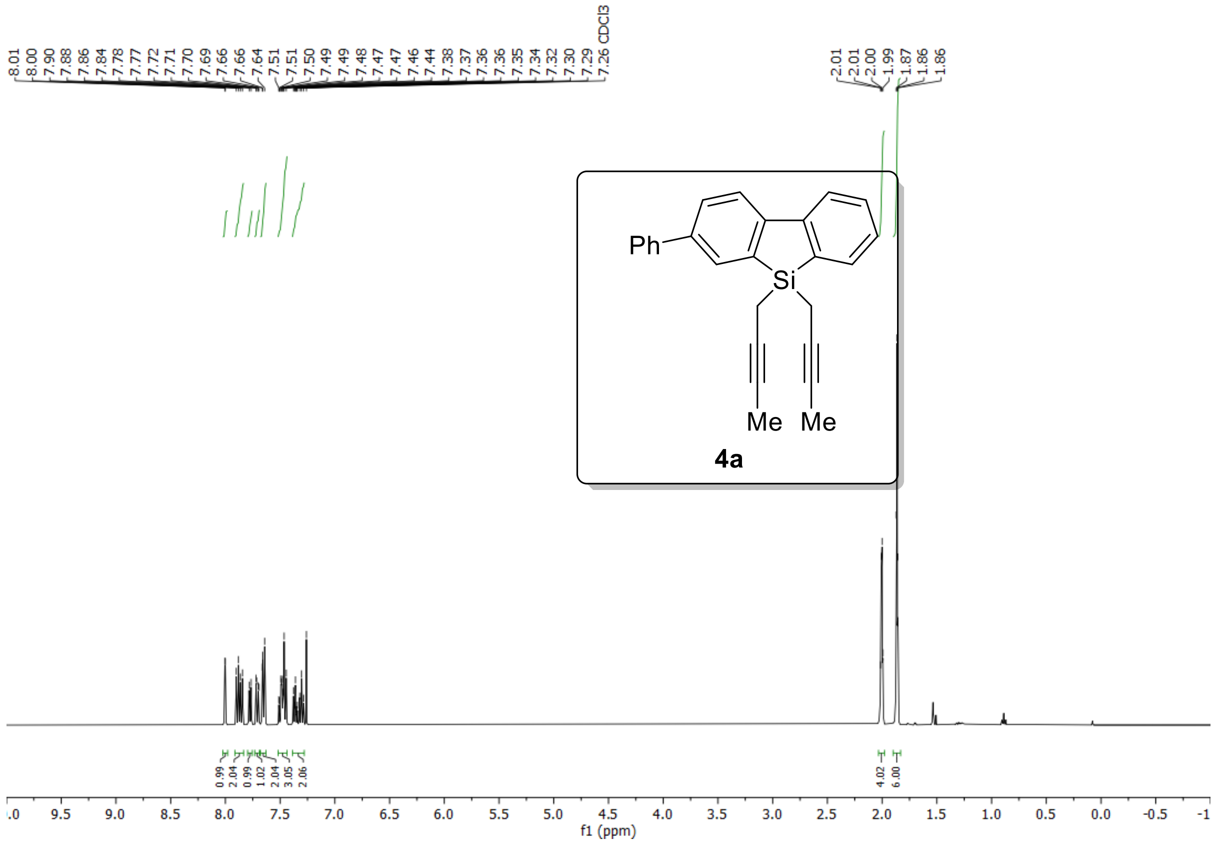
Ph
Si
Me
Me
4a
f1 (ppm)

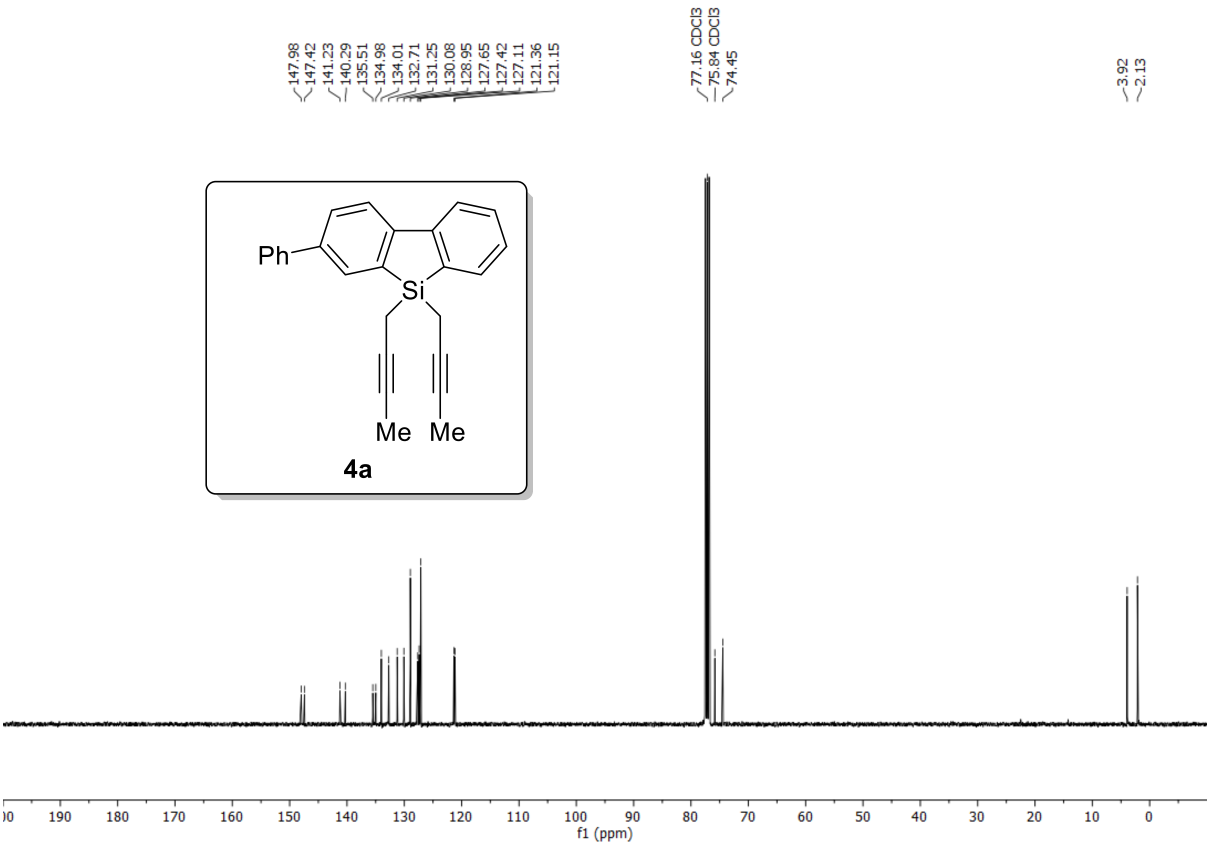
147.98
147.42
141.23
140.29
135.51
134.98
134.01
132.71
131.25
130.08
128.95
127.65
127.42
127.11
121.36
121.15
77.16 CDCl3
75.84 CDCl3
74.45
3.92
2.13
Ph
Si
Me Me
4a
f1 (ppm)

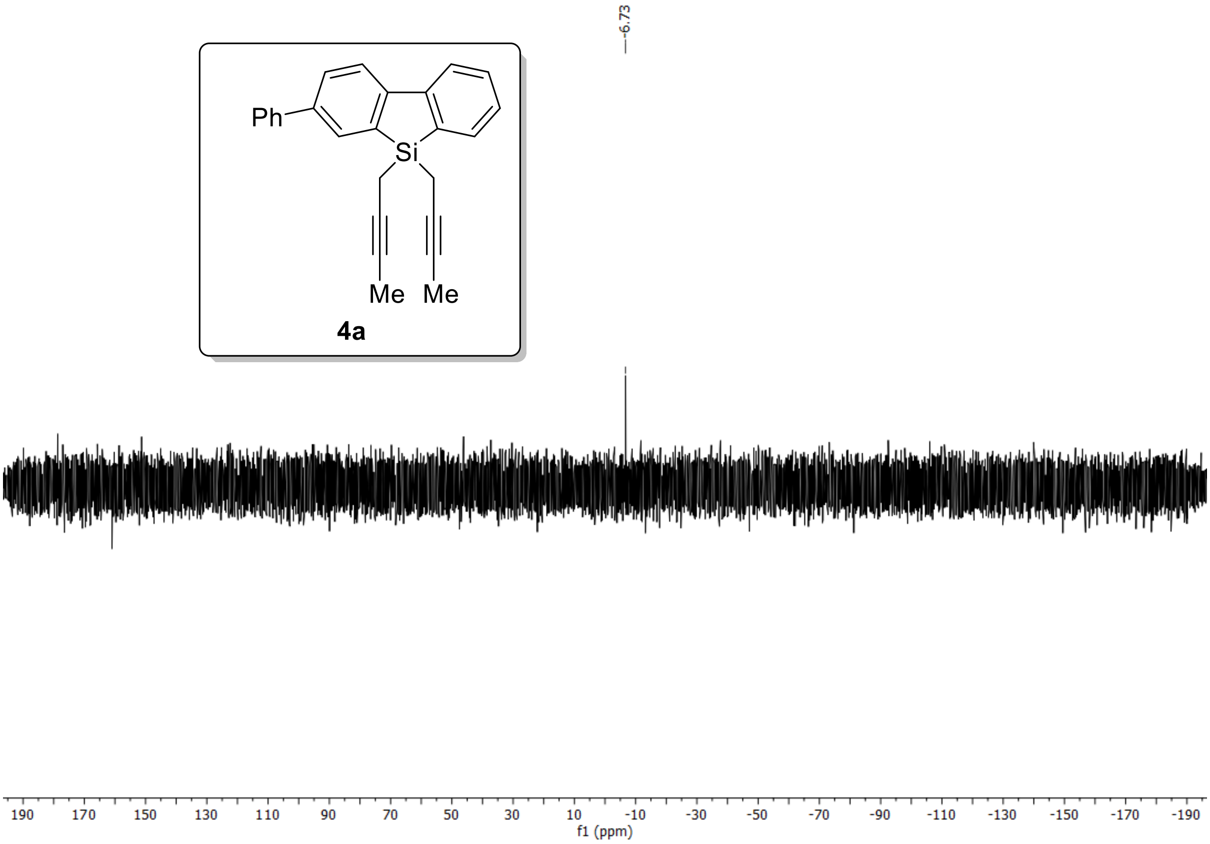
-6.73
Ph
Si
Me Me
4a
f1 (ppm)

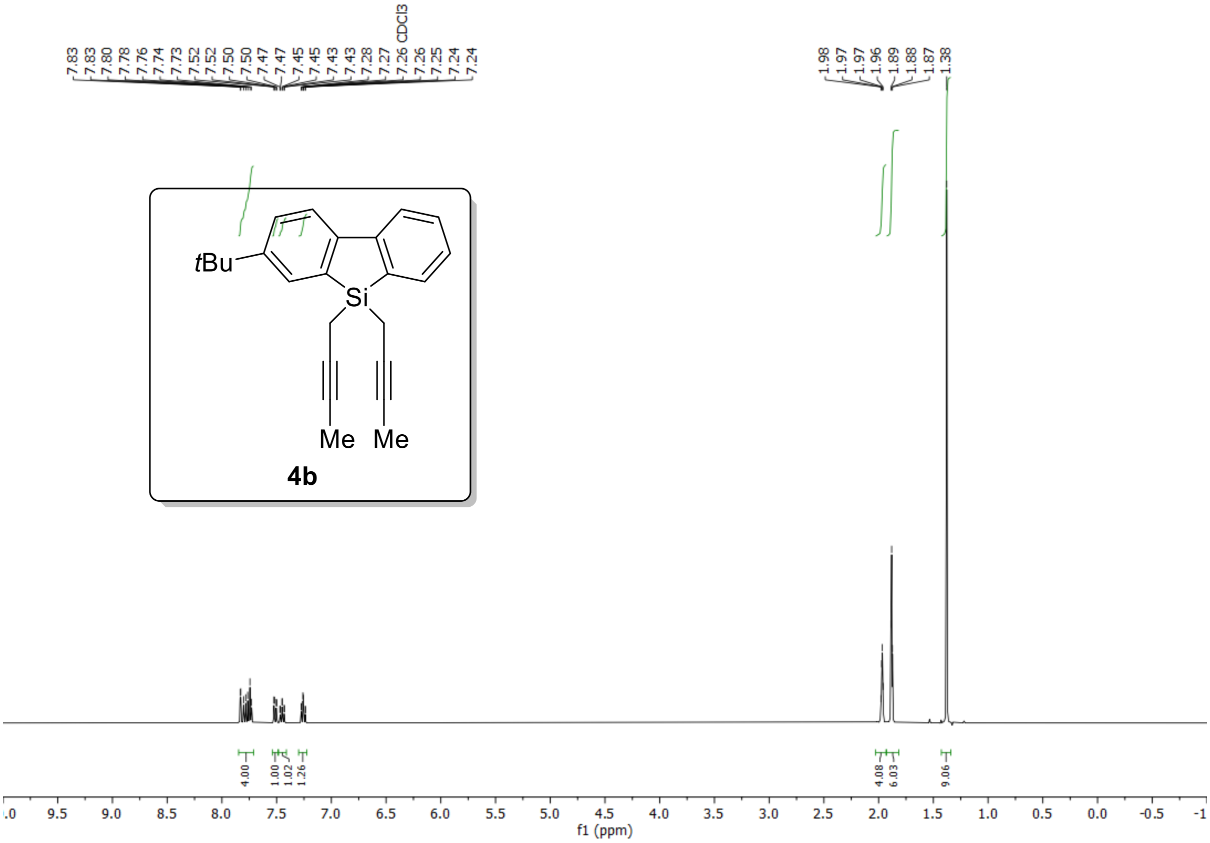
7.83
7.83
7.80
7.78
7.76
7.74
7.73
7.52
7.52
7.50
7.50
7.47
7.47
7.45
7.45
7.43
7.43
7.28
7.27
7.26 CDCl3
7.26
7.25
7.24
7.24
1.98
1.97
1.97
1.96
1.89
1.88
1.87
1.38
tBu
Si
Me Me
4b
4.00
1.00
1.02
1.26
4.08
6.03
9.06
f1 (ppm)

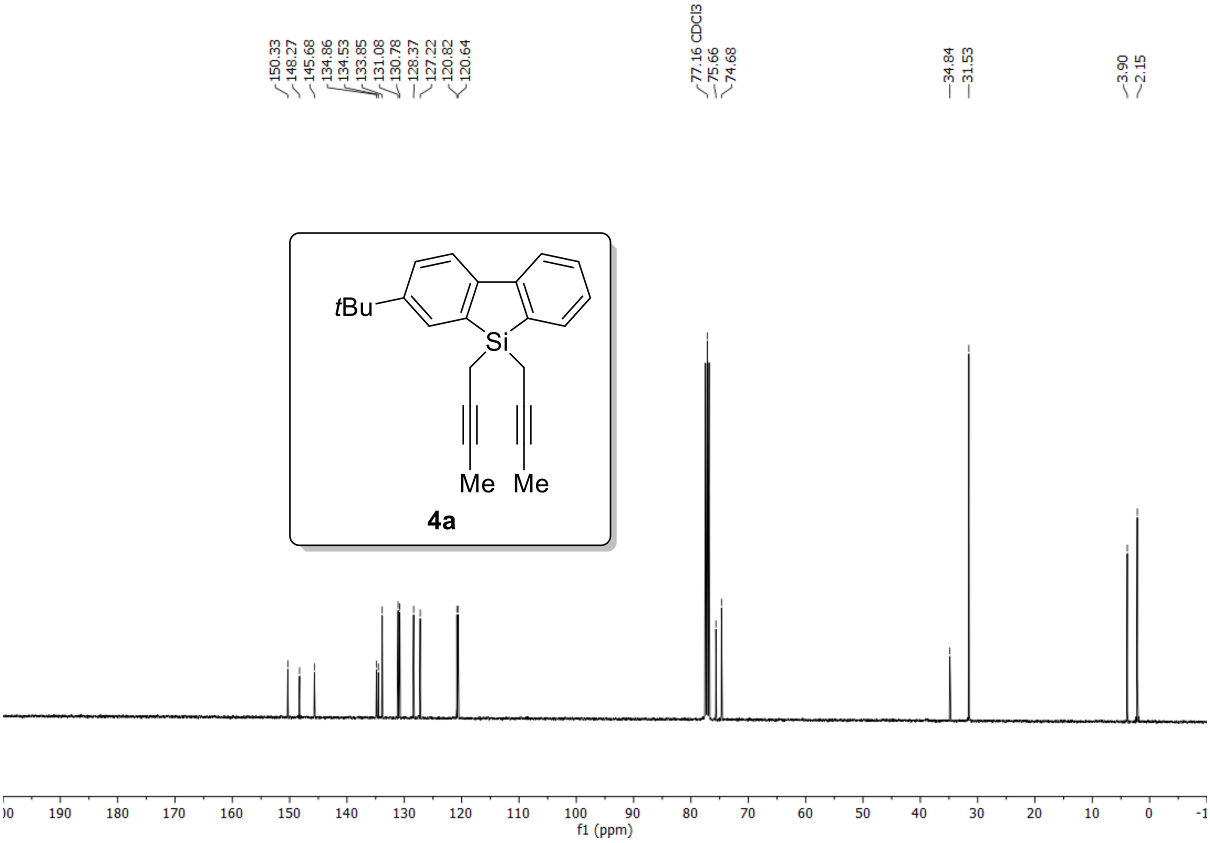
150.33
148.27
145.68
134.86
134.53
133.85
131.08
130.78
128.37
127.22
120.82
120.64
77.16 CDCl3
75.66
74.68
34.84
31.53
3.90
2.15
tBu
Si
Me Me
4a
f1 (ppm)

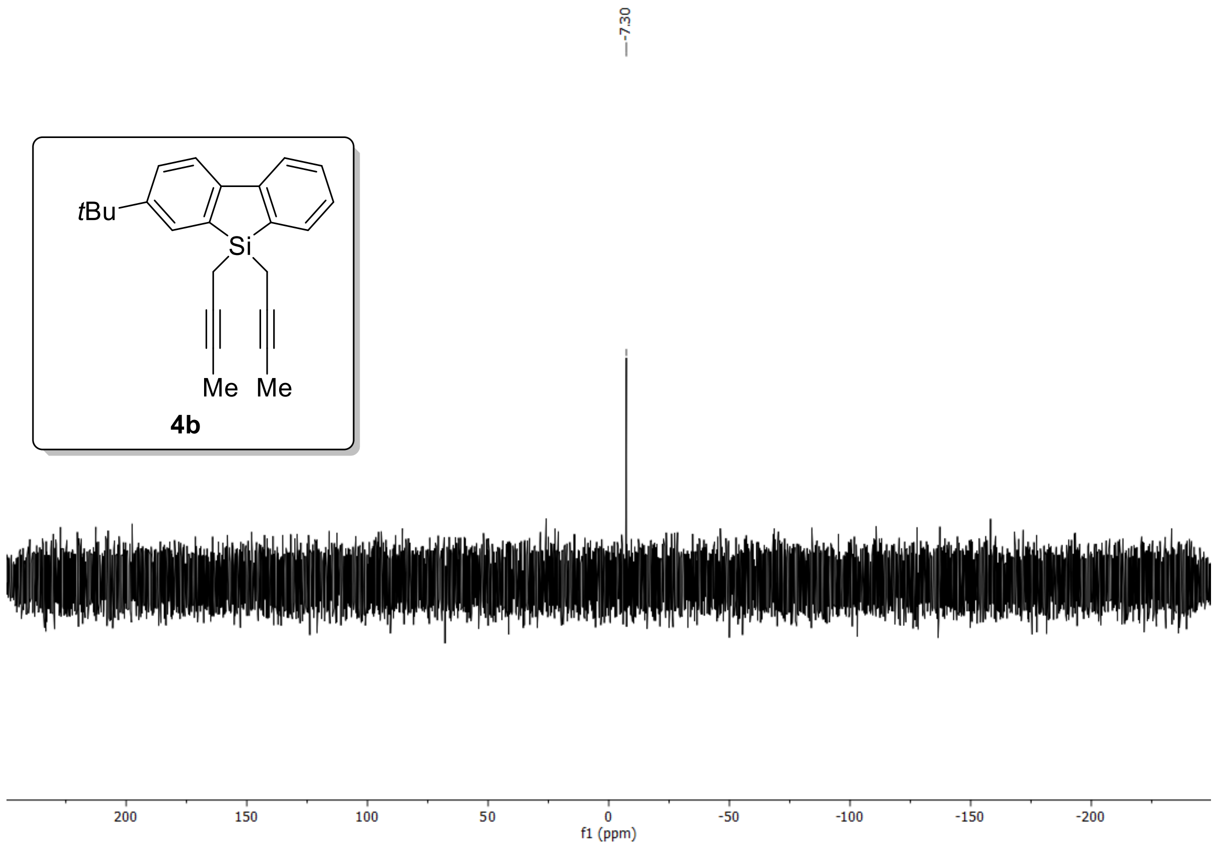
-7.30
tBu
Si
Me Me
4b
200 150 100 50 0 -50 -100 -150 -200
f1 (ppm)

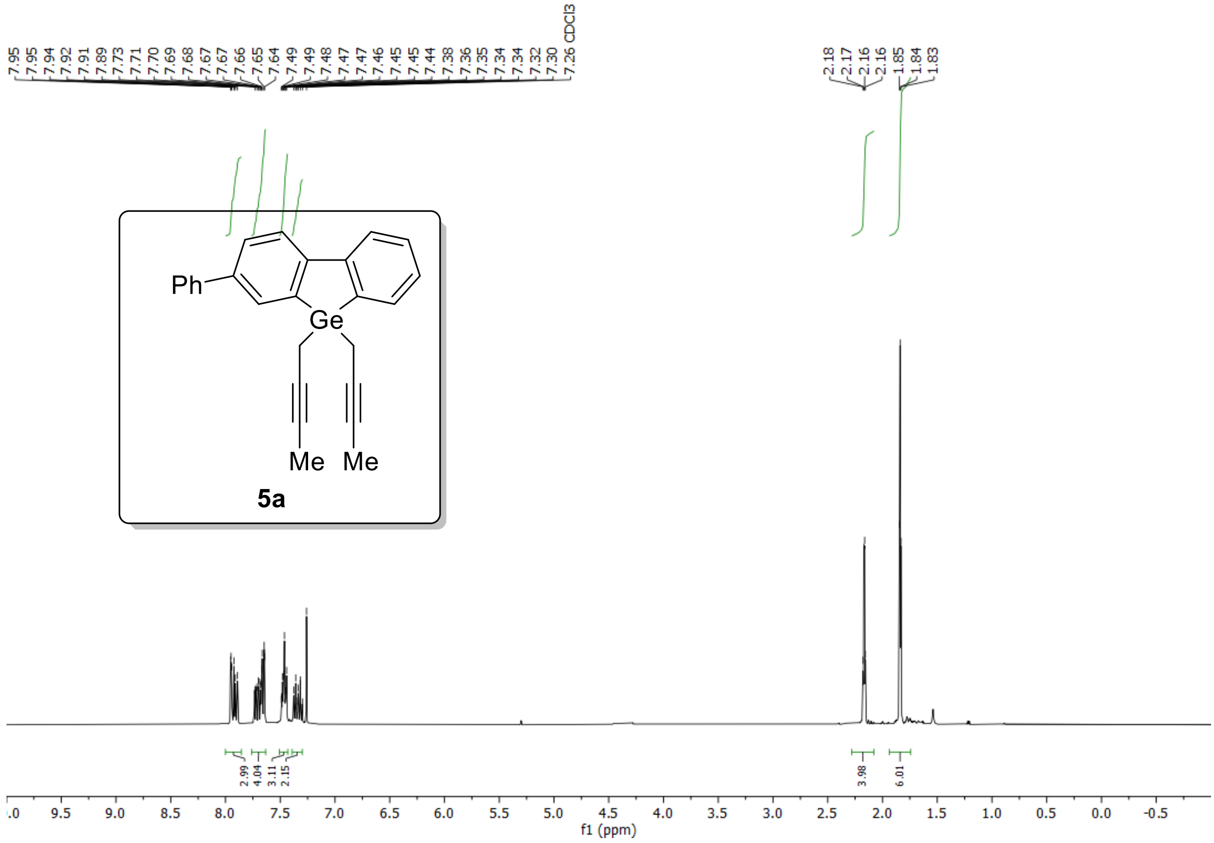
7.95 7.95 7.94 7.92 7.91 7.89 7.73 7.71 7.70 7.69 7.68 7.67 7.67 7.66 7.65 7.64 7.49 7.49 7.48 7.47 7.47 7.46 7.45 7.45 7.44 7.38 7.36 7.35 7.34 7.32 7.30 7.26 CDCl3
2.18 2.17 2.16 2.16 1.85 1.84 1.83
Ph
Ge
Me Me
5a
2.99 4.04 3.11 2.15
3.98
6.01
.0 9.5 9.0 8.5 8.0 7.5 7.0 6.5 6.0 5.5 5.0 4.5 4.0 3.5 3.0 2.5 2.0 1.5 1.0 0.5 0.0 -0.5
f1 (ppm)

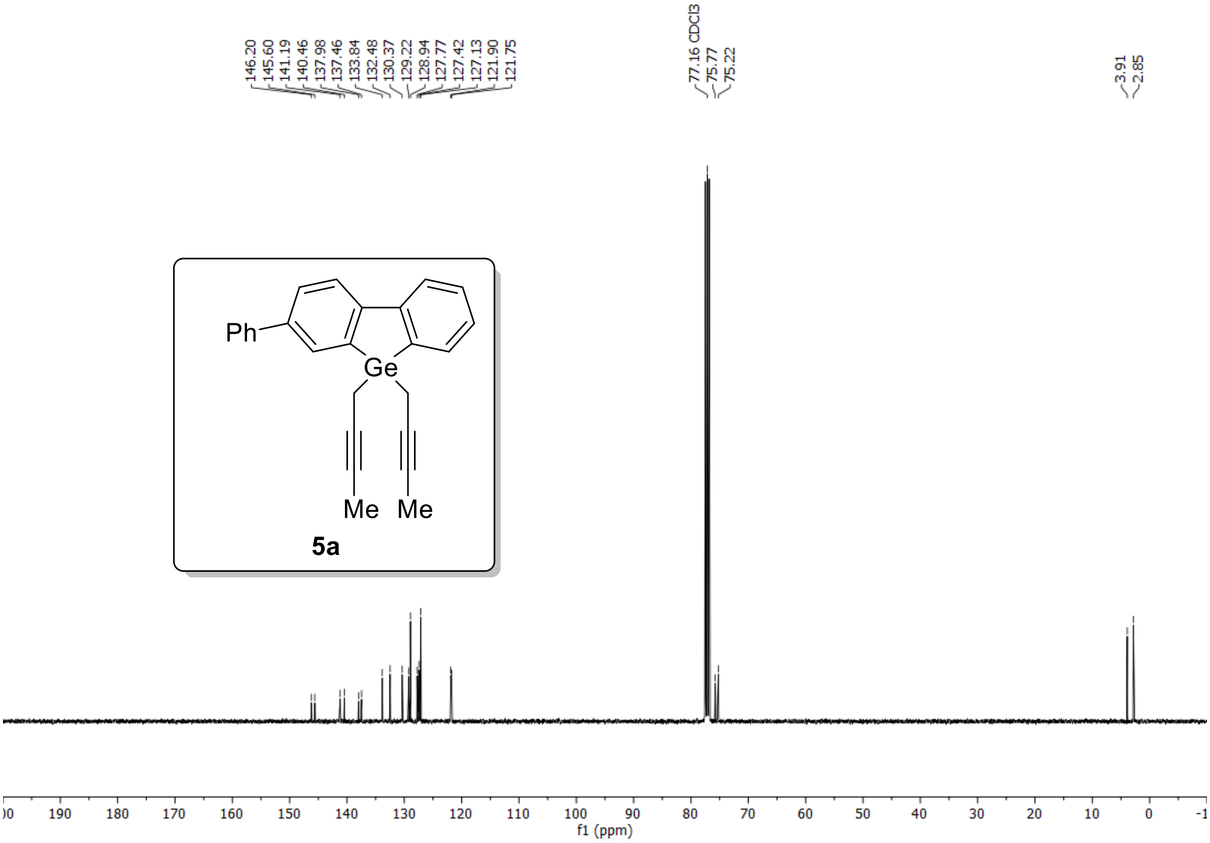
146.20
145.60
141.19
140.46
137.98
137.46
133.84
132.48
130.37
129.22
128.94
127.77
127.42
127.13
121.90
121.75
77.16 CDCl3
75.77
75.22
3.91
2.85
Ph
Ge
Me Me
5a
f1 (ppm)

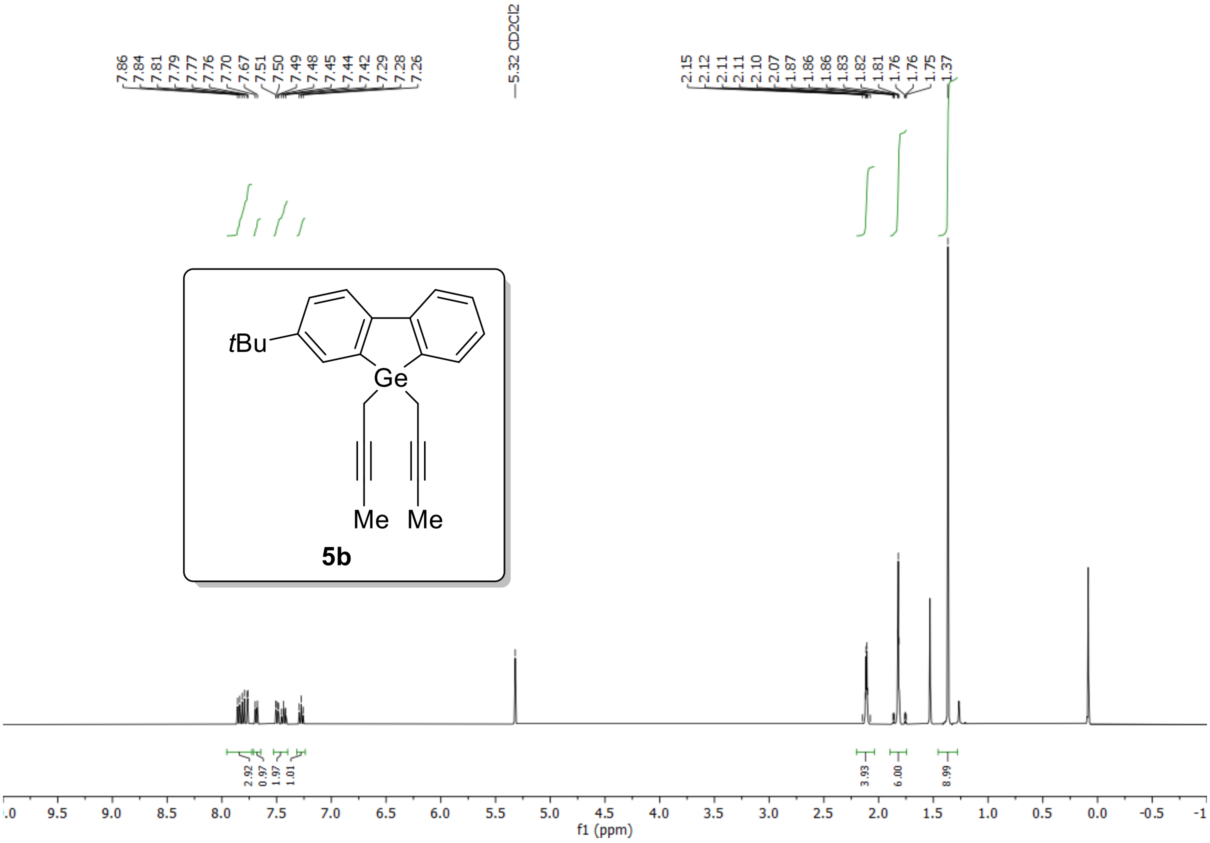
7.86
7.84
7.81
7.79
7.77
7.76
7.70
7.67
7.51
7.50
7.49
7.48
7.45
7.44
7.42
7.29
7.28
7.26
5.32 CD2Cl2
2.15
2.12
2.11
2.11
2.10
2.07
1.87
1.86
1.86
1.83
1.82
1.81
1.76
1.76
1.75
1.37
tBu
Ge
Me Me
5b
2.92
0.97
1.97
1.01
3.93
6.00
8.99
f1 (ppm)

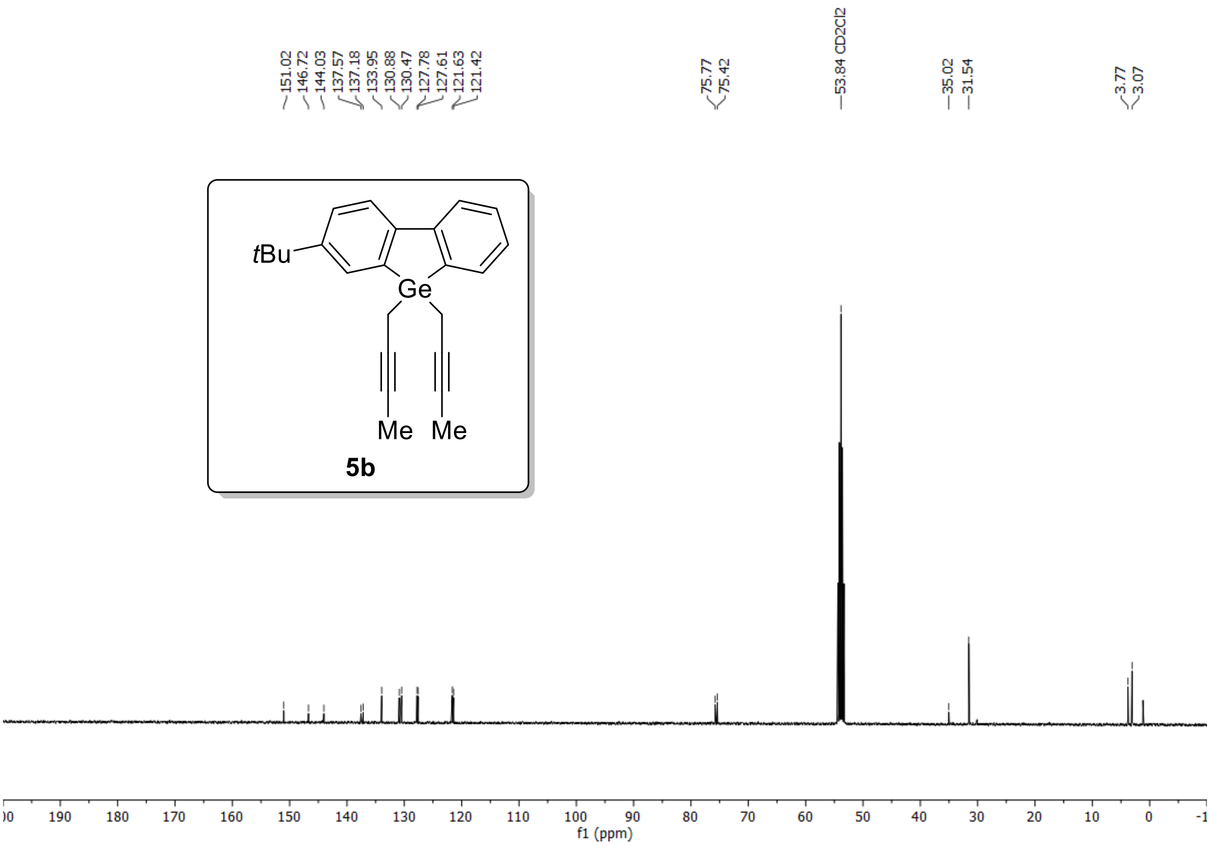

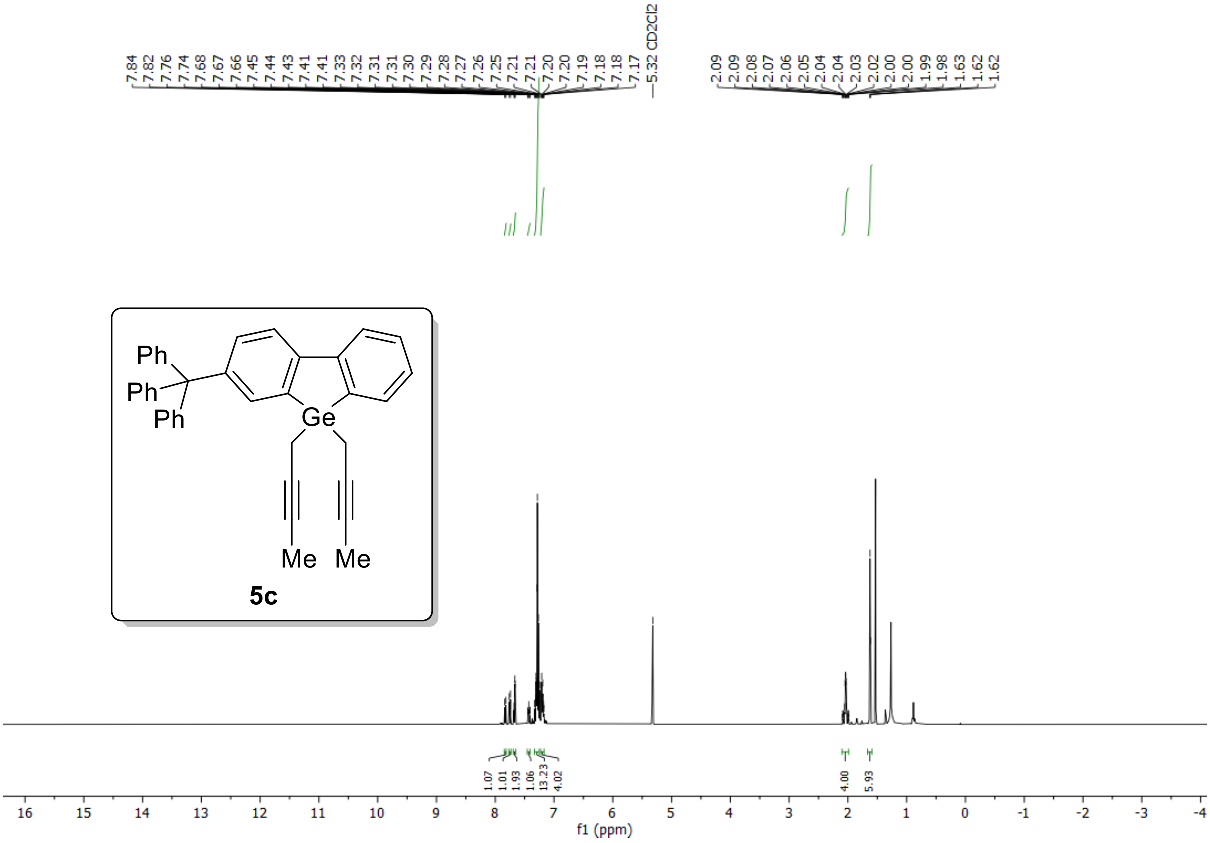

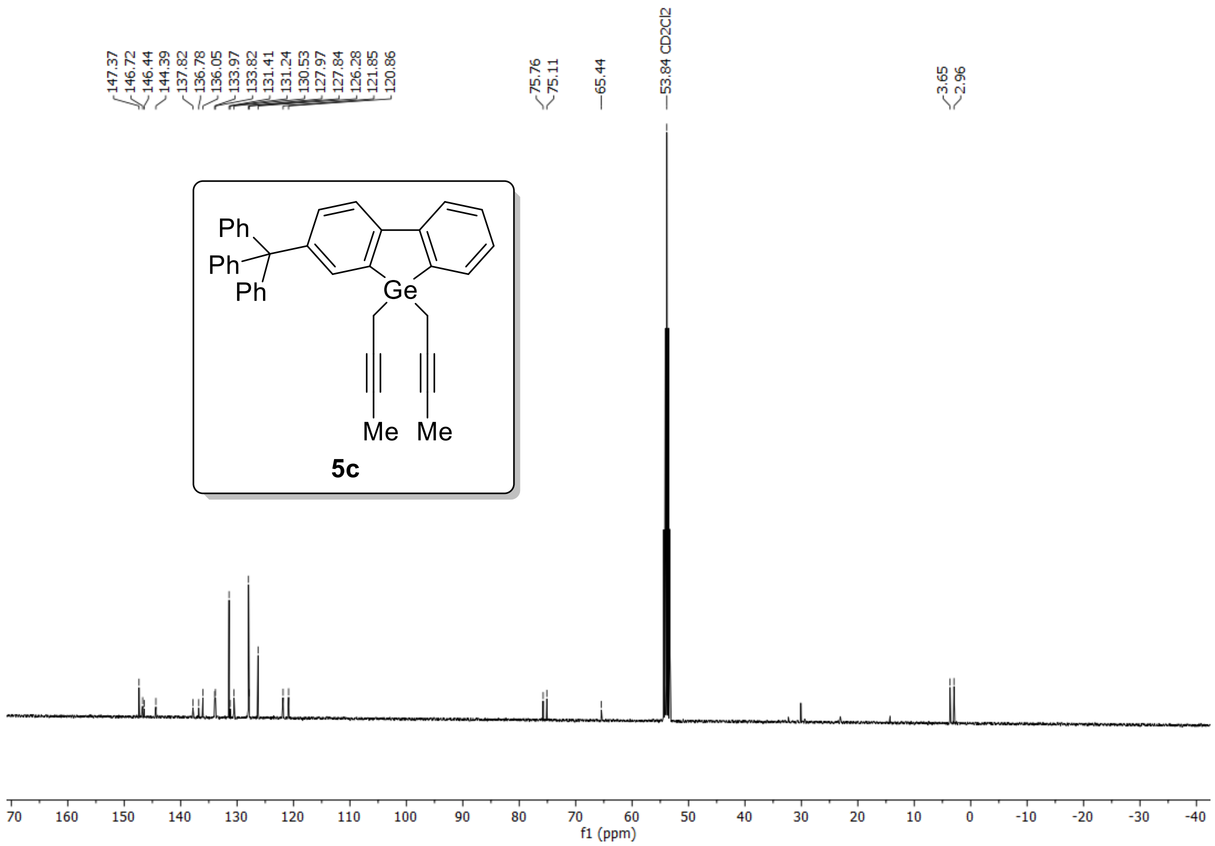
147.37
146.72
146.44
144.39
137.82
136.78
136.05
133.97
133.82
131.41
131.24
130.53
127.97
127.84
126.28
121.85
120.86
75.76
75.11
65.44
53.84 CD2Cl2
3.65
2.96
Ph
Ph
Ph
Ge
Me
Me
5c
f1 (ppm)

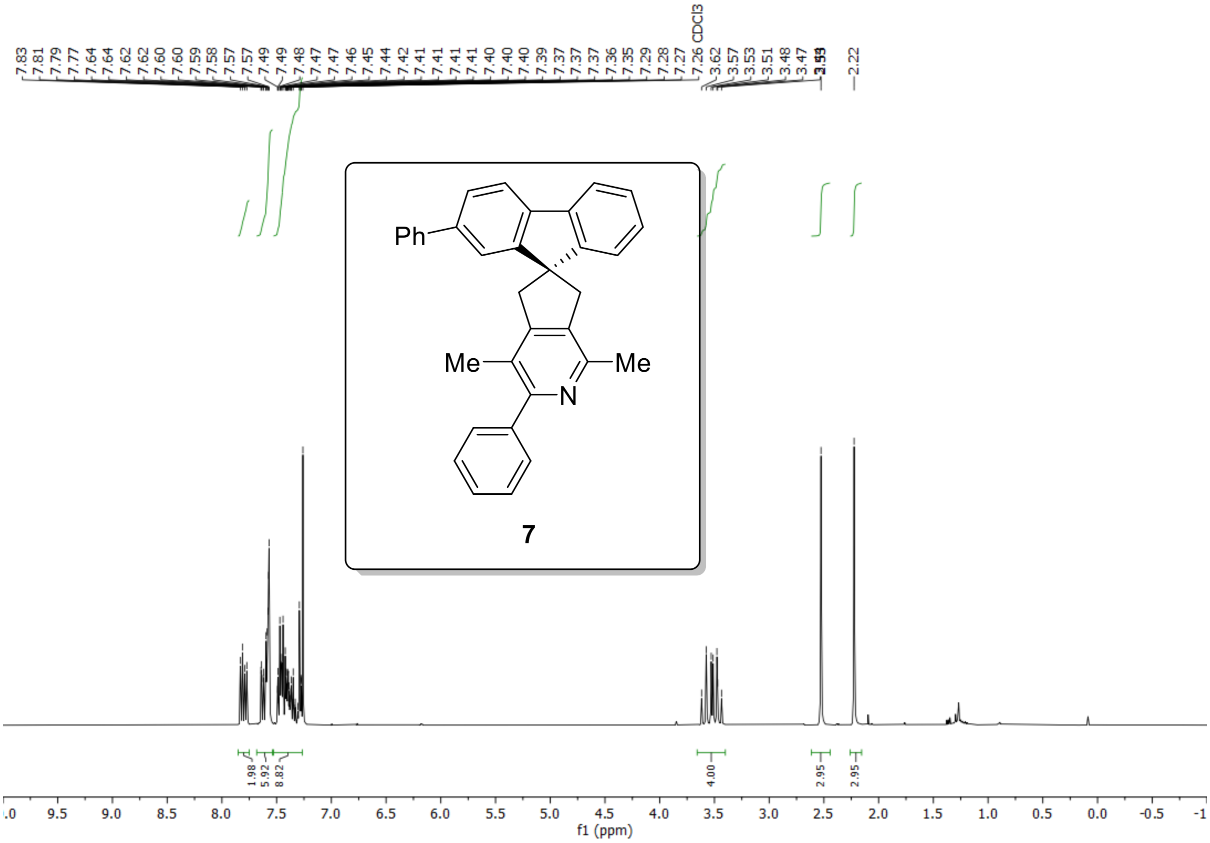
7.83
7.81
7.79
7.77
7.64
7.64
7.62
7.62
7.60
7.60
7.59
7.58
7.57
7.57
7.49
7.49
7.48
7.47
7.47
7.46
7.45
7.44
7.42
7.41
7.41
7.41
7.41
7.40
7.40
7.39
7.37
7.37
7.37
7.36
7.35
7.29
7.28
7.27
7.26 CDCl3
3.62
3.57
3.53
3.51
3.48
3.47
2.53
[illegible]
2.22
Ph
Me
Me
N
7
1.98
5.92
8.82
4.00
2.95
2.95
f1 (ppm)

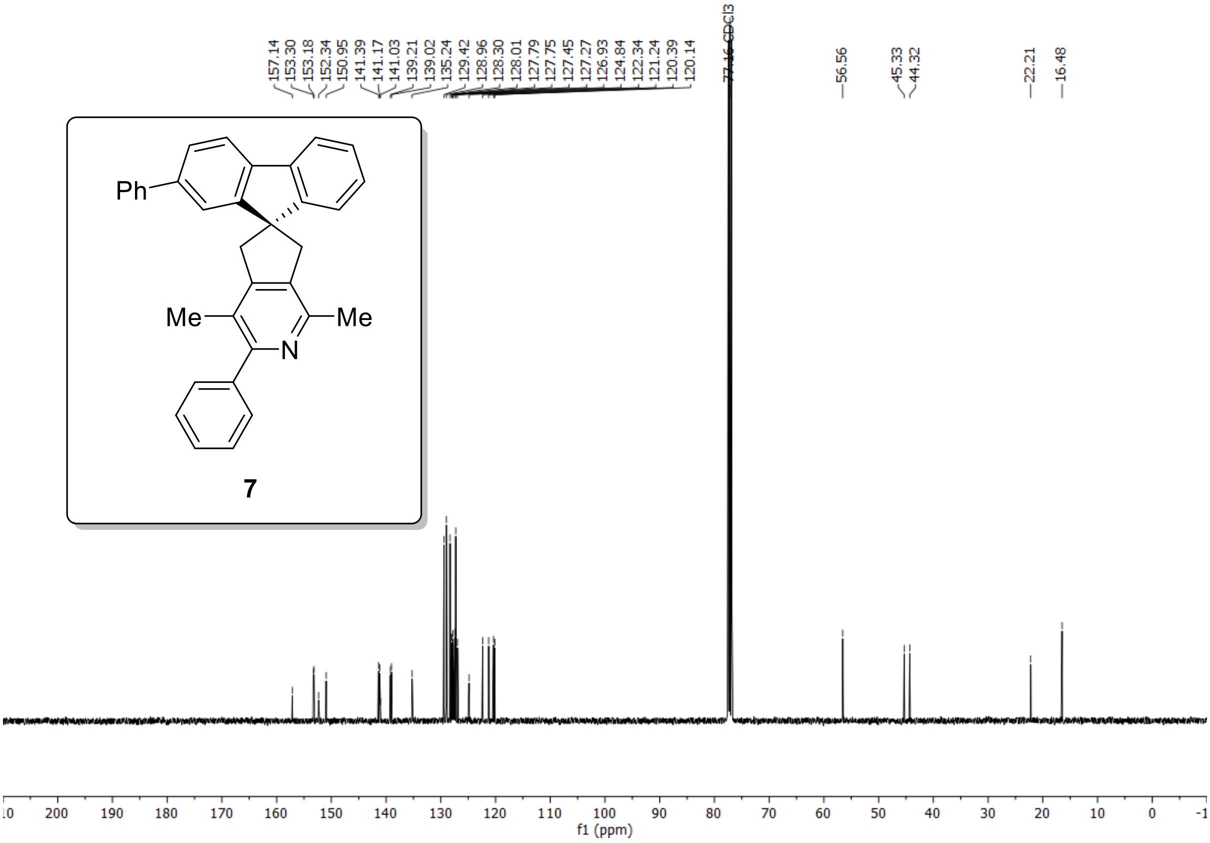
157.14
153.30
153.18
152.34
150.95
141.39
141.17
141.03
139.21
139.02
135.24
129.42
128.96
128.30
128.01
127.79
127.75
127.45
127.27
126.93
124.84
122.34
121.24
120.39
120.14
77.16 CDCl3
56.56
45.33
44.32
22.21
16.48
Ph
Me
Me
N
7
f1 (ppm)

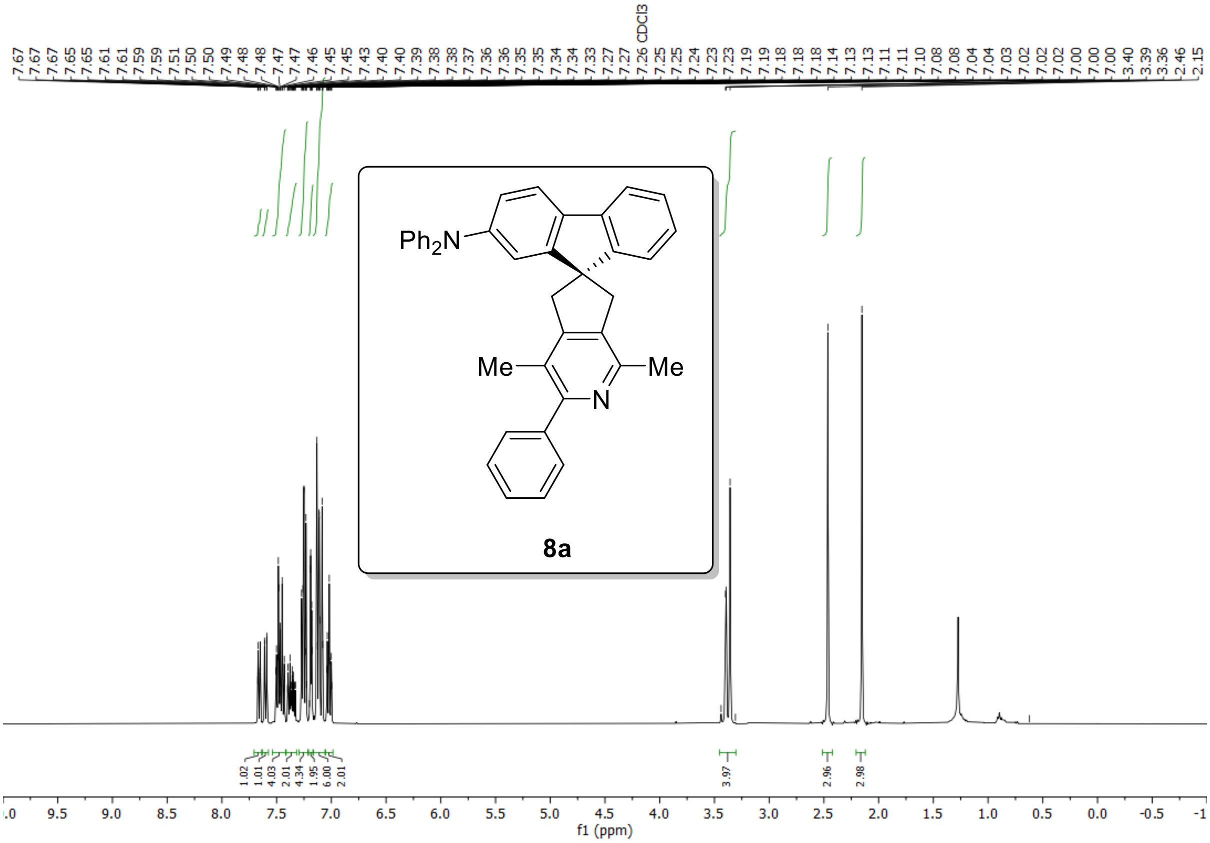
7.67 7.67 7.65 7.65 7.61 7.61 7.59 7.59 7.51 7.50 7.50 7.49 7.48 7.48 7.47 7.47 7.46 7.45 7.45 7.43 7.40 7.40 7.39 7.38 7.38 7.37 7.36 7.36 7.35 7.35 7.34 7.34 7.33 7.27 7.27 7.26 CDCl3 7.25 7.25 7.24 7.23 7.23 7.19 7.19 7.18 7.18 7.18 7.14 7.13 7.13 7.11 7.11 7.10 7.08 7.08 7.04 7.04 7.03 7.02 7.02 7.02 7.00 7.00 3.40 3.39 3.36 2.46 2.15
Ph2N
Me
Me
N
8a
1.02 1.01 4.03 2.01 4.34 1.95 6.00 2.01
3.97
2.96
2.98
f1 (ppm)

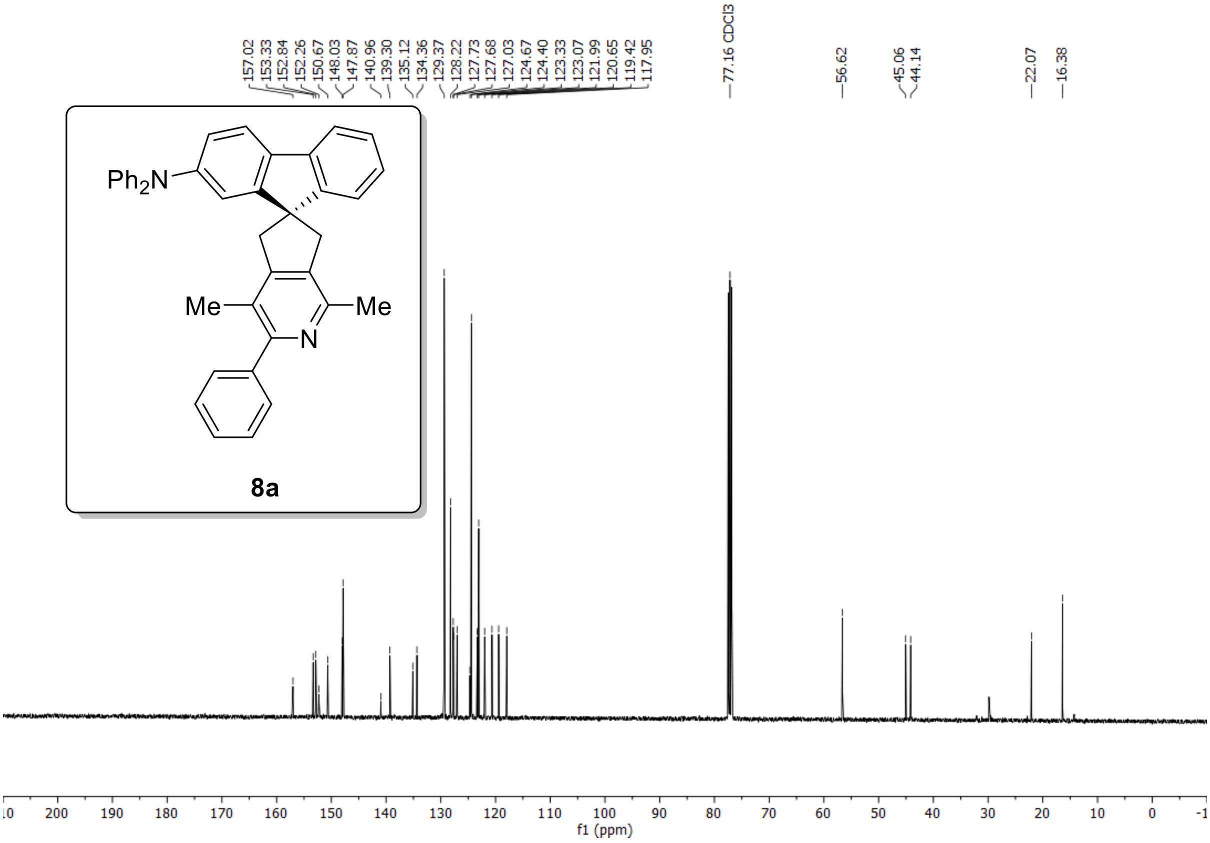
157.02 153.33 152.84 152.26 150.67 148.03 147.87 140.96 139.30 135.12 134.36 129.37 128.22 127.73 127.68 127.03 124.67 124.40 123.33 123.07 121.99 120.65 119.42 117.95
77.16 CDCl3
56.62
45.06 44.14
22.07
16.38
Ph2N
Me
Me
N
8a
f1 (ppm)

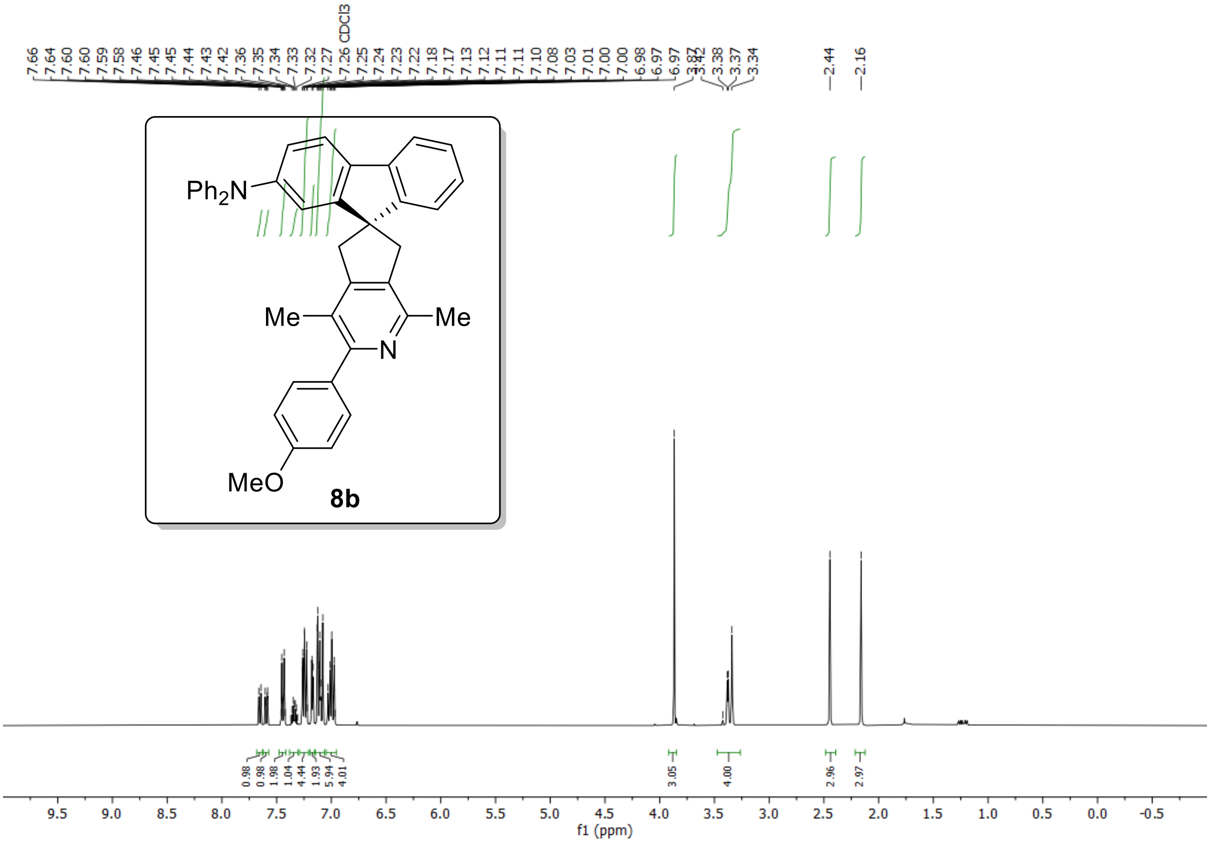
7.66 7.64 7.60 7.60 7.59 7.58 7.46 7.45 7.45 7.44 7.43 7.42 7.36 7.35 7.34 7.33 7.32 7.27 7.26 CDCl3 7.25 7.24 7.23 7.22 7.18 7.17 7.13 7.12 7.11 7.11 7.10 7.08 7.03 7.01 7.00 7.00 6.98 6.97 3.87 3.42 3.38 3.37 3.34 2.44 2.16
Ph2N
Me
Me
N
MeO
8b
f1 (ppm)

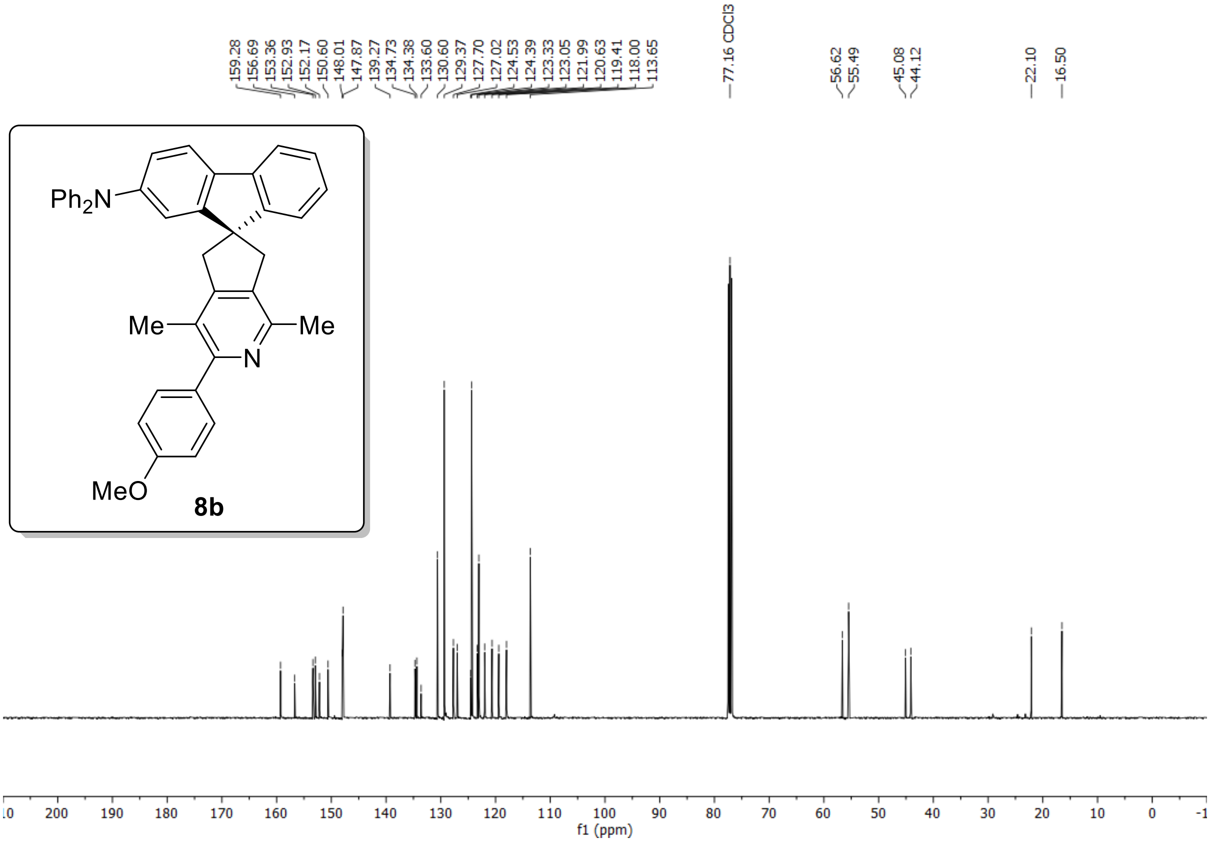
159.28 156.69 153.36 152.93 152.17 150.60 148.01 147.87 139.27 134.73 134.38 133.60 130.60 129.37 127.70 127.02 124.53 124.39 123.33 123.05 121.99 120.63 119.41 118.00 113.65 77.16 CDCl3 56.62 55.49 45.08 44.12 22.10 16.50
Ph2N
Me
Me
N
MeO
8b
f1 (ppm)

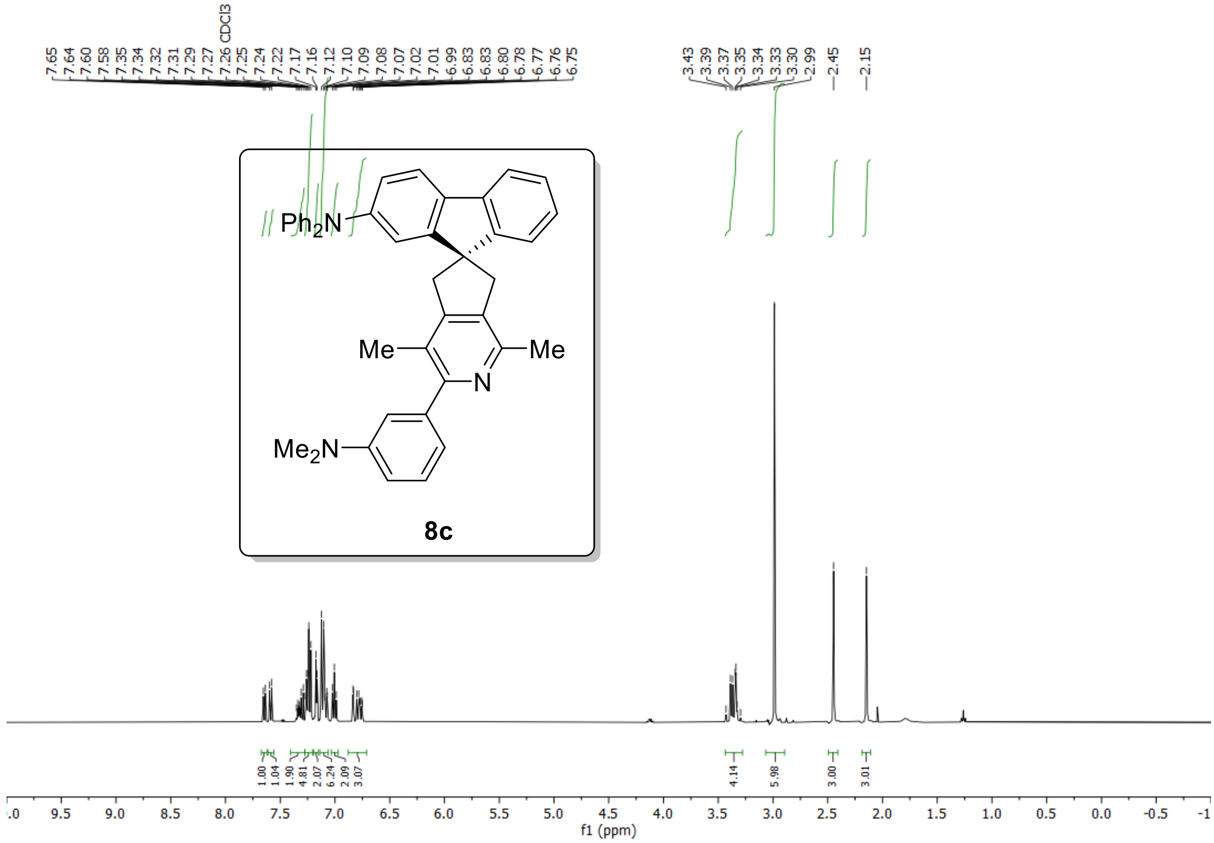

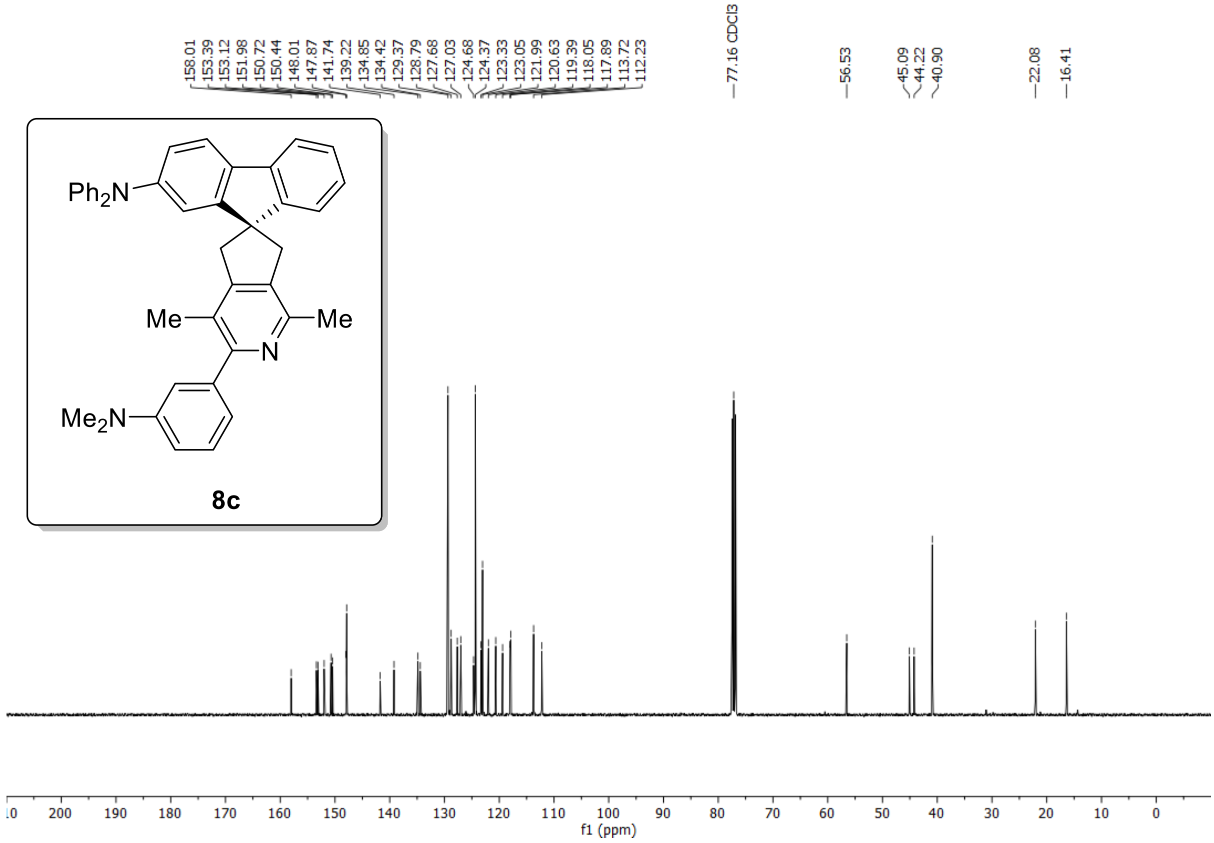

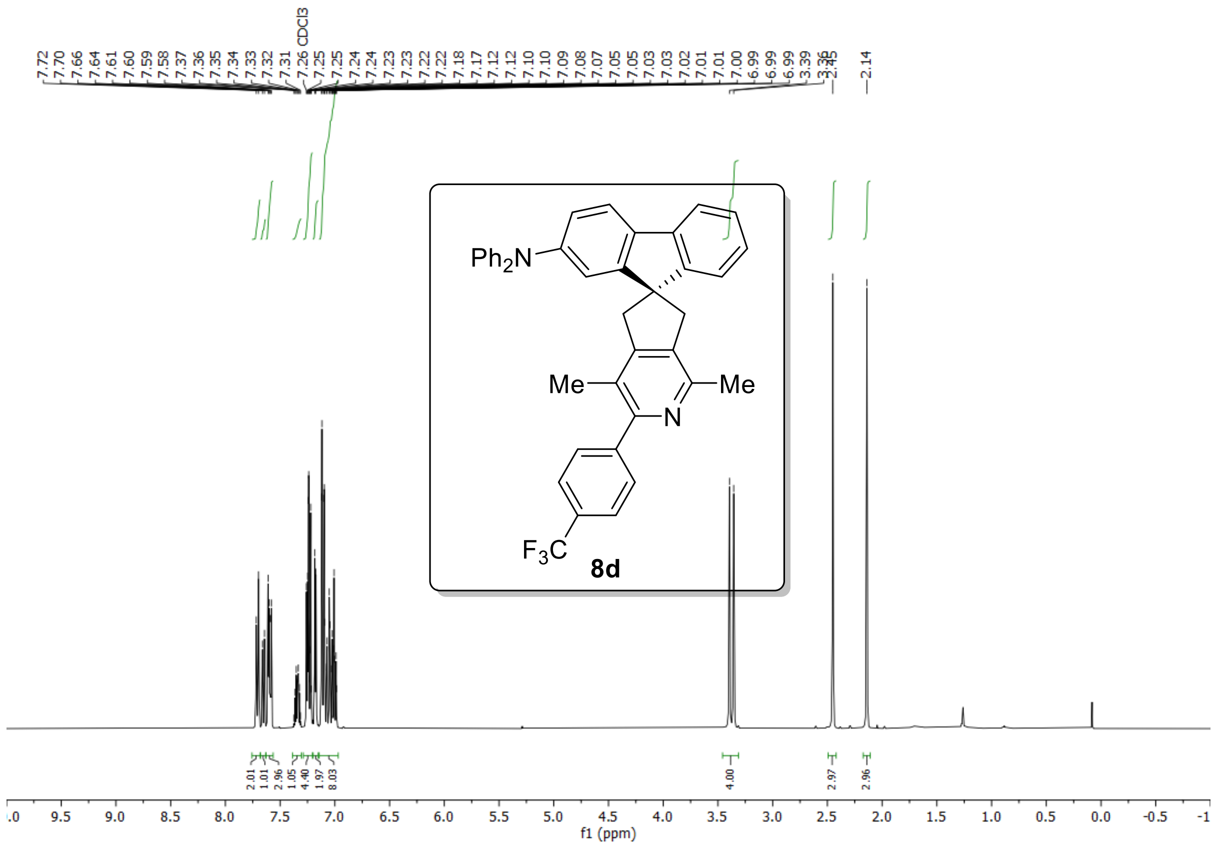
7.72
7.70
7.66
7.64
7.61
7.60
7.59
7.58
7.37
7.36
7.35
7.34
7.33
7.32
7.31
7.26 CDCl3
7.25
7.25
7.24
7.24
7.23
7.23
7.22
7.22
7.18
7.17
7.12
7.12
7.10
7.10
7.09
7.08
7.07
7.05
7.05
7.03
7.03
7.02
7.01
7.01
7.00
6.99
6.99
6.99
3.39
3.36
2.45
2.14
Ph2N
Me
Me
N
F3C
8d
2.01
1.01
2.96
1.05
4.40
1.97
8.03
4.00
2.97
2.96
f1 (ppm)

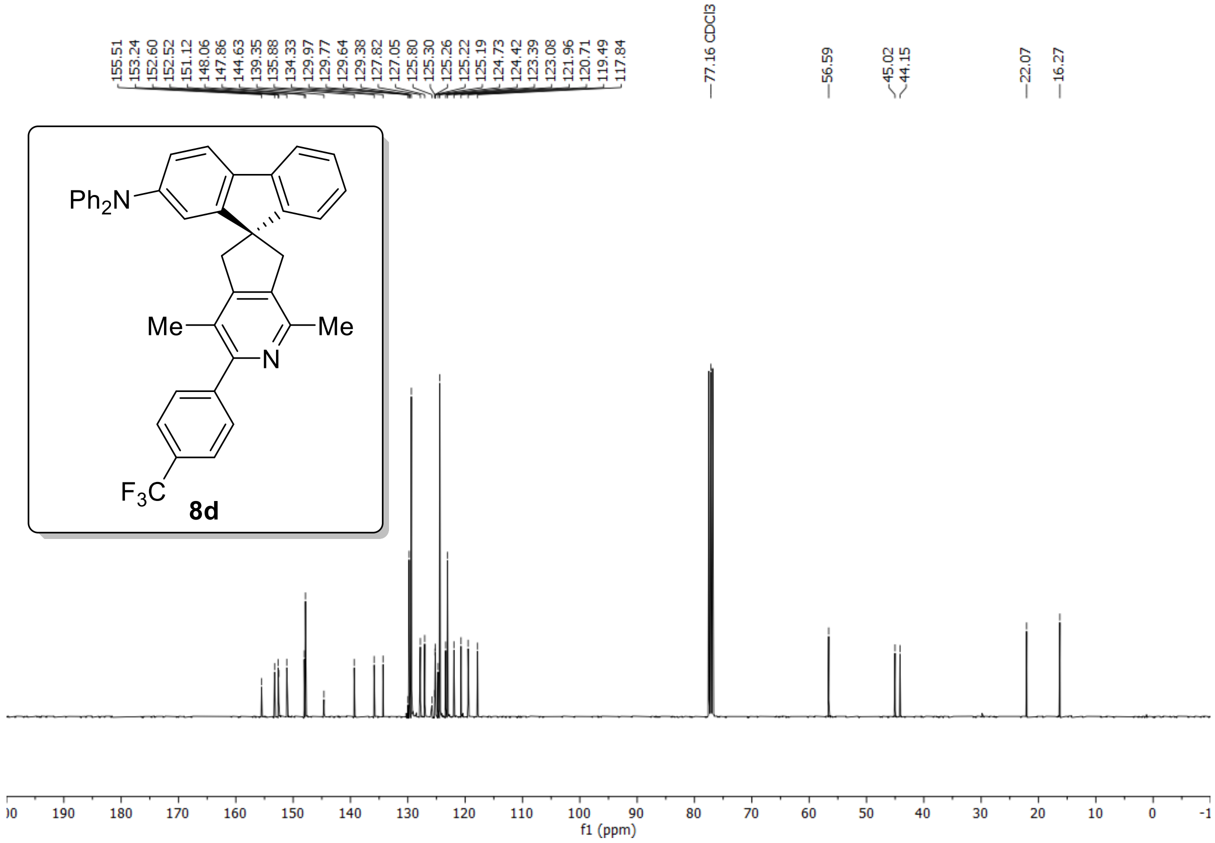
155.51
153.24
152.60
152.52
151.12
148.06
147.86
144.63
139.35
135.88
134.33
129.97
129.77
129.64
129.38
127.82
127.05
125.80
125.30
125.26
125.22
125.19
124.73
124.42
123.39
123.08
121.96
120.71
119.49
117.84
77.16 CDCl3
56.59
45.02
44.15
22.07
16.27
Ph2N
Me
Me
N
F3C
8d
f1 (ppm)

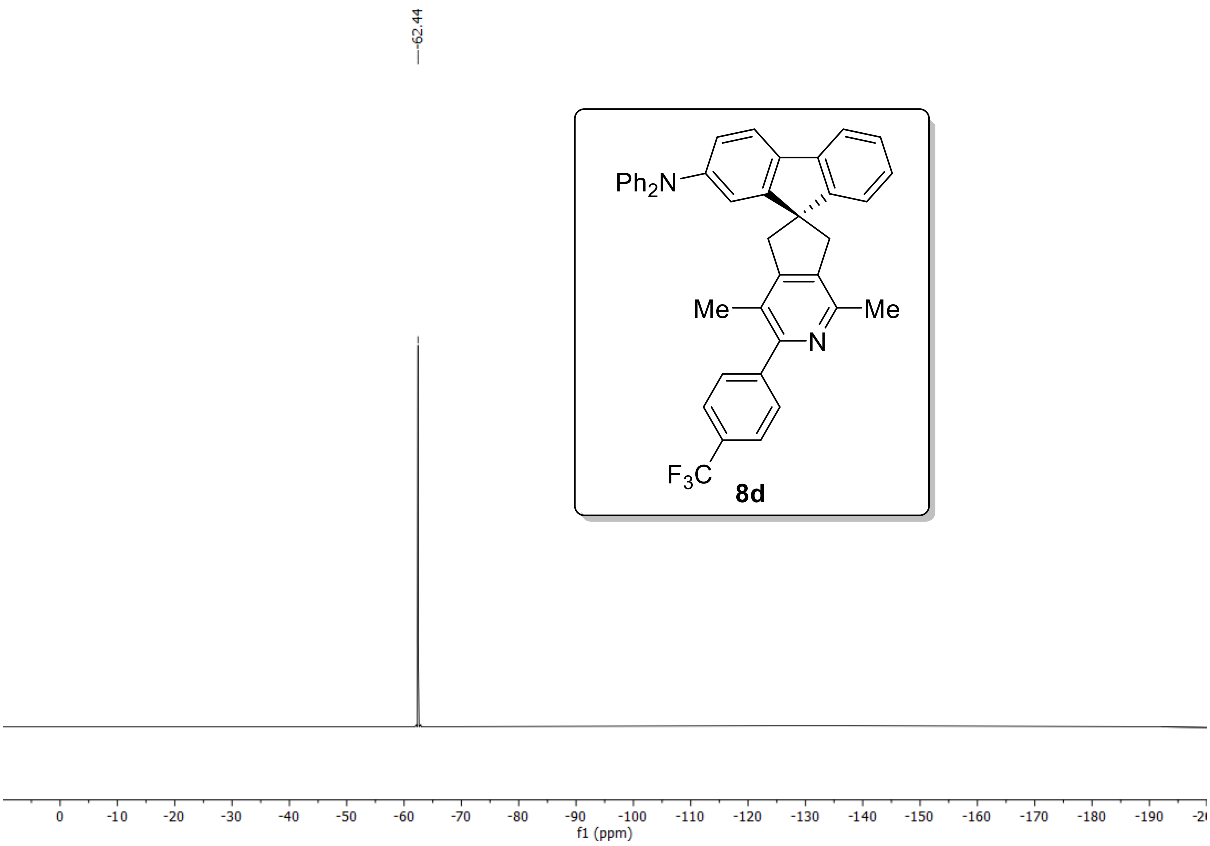

—62.44
Ph2N
Me
Me
N
F3C
8d
0
-10
-20
-30
-40
-50
-60
-70
-80
-90
-100
-110
-120
-130
-140
-150
-160
-170
-180
-190
f1 (ppm)


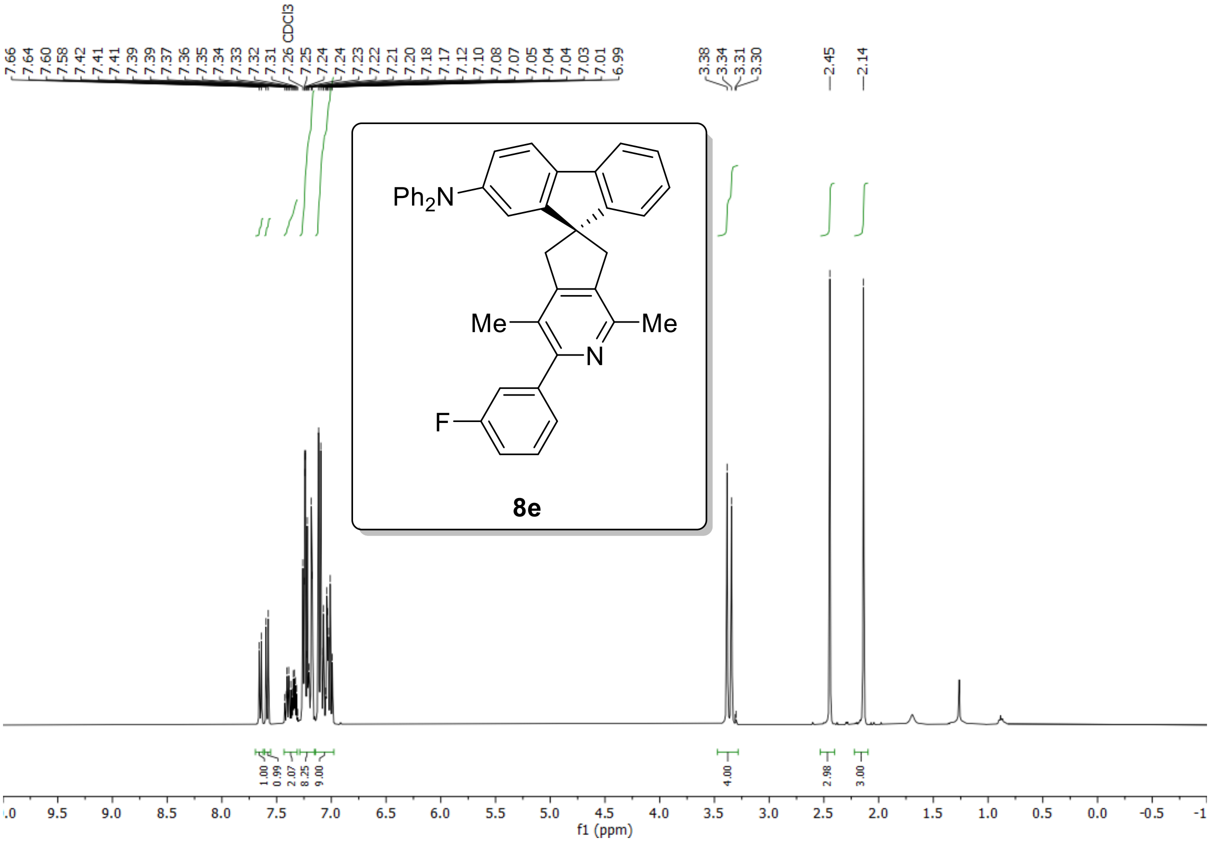

CDCl3
3.38
3.34
3.31
3.30
2.45
2.14
Ph2N
Me
Me
N
F
8e
4.00
2.98
3.00
9.5
9.0
8.5
8.0
7.5
7.0
6.5
6.0
5.5
5.0
4.5
4.0
3.5
3.0
2.5
2.0
1.5
1.0
0.5
0.0
-0.5
f1 (ppm)

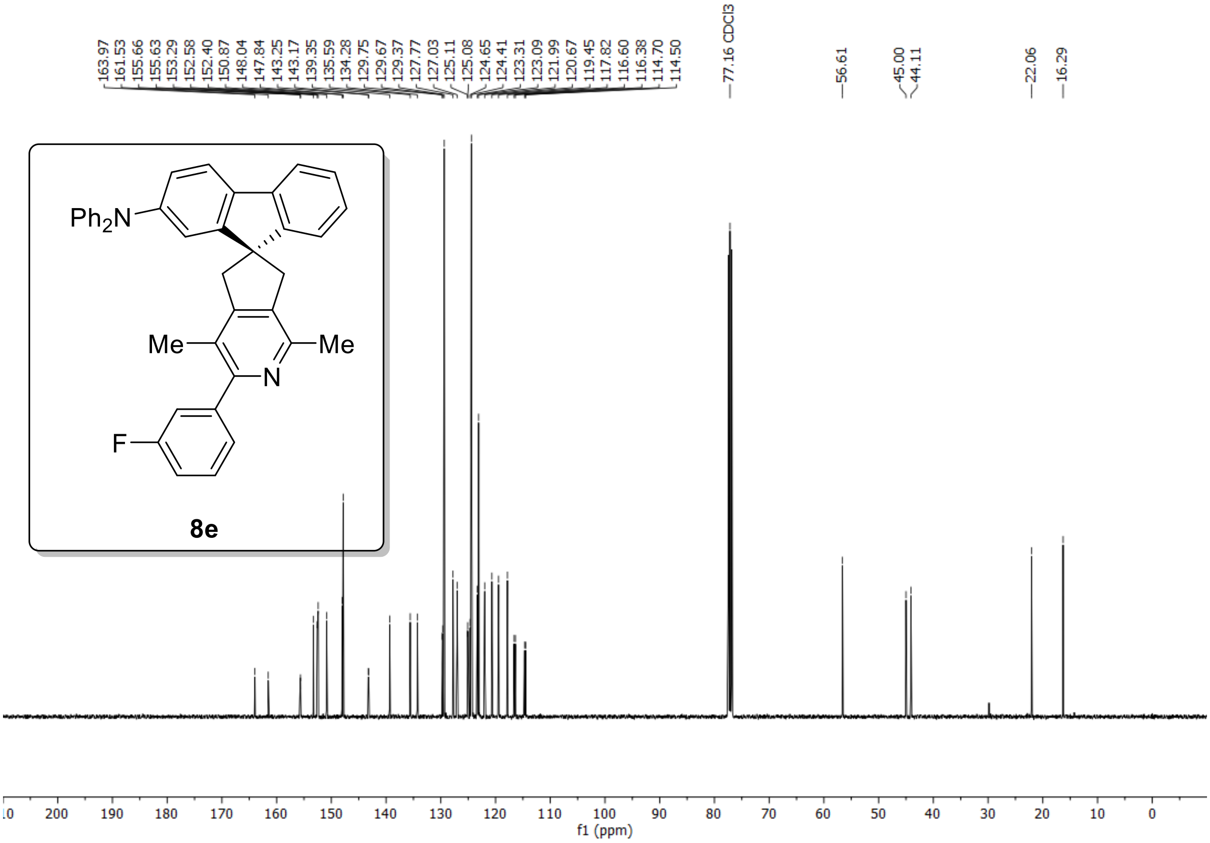
163.97
161.53
155.66
155.63
153.29
152.58
152.40
150.87
148.04
147.84
143.25
143.17
139.35
135.59
134.28
129.75
129.67
129.37
127.77
127.03
125.11
125.08
124.65
124.41
123.31
123.09
121.99
120.67
119.45
117.82
116.60
116.38
114.70
114.50
77.16 CDCl3
56.61
45.00
44.11
22.06
16.29
Ph2N
Me
Me
N
F
8e
f1 (ppm)

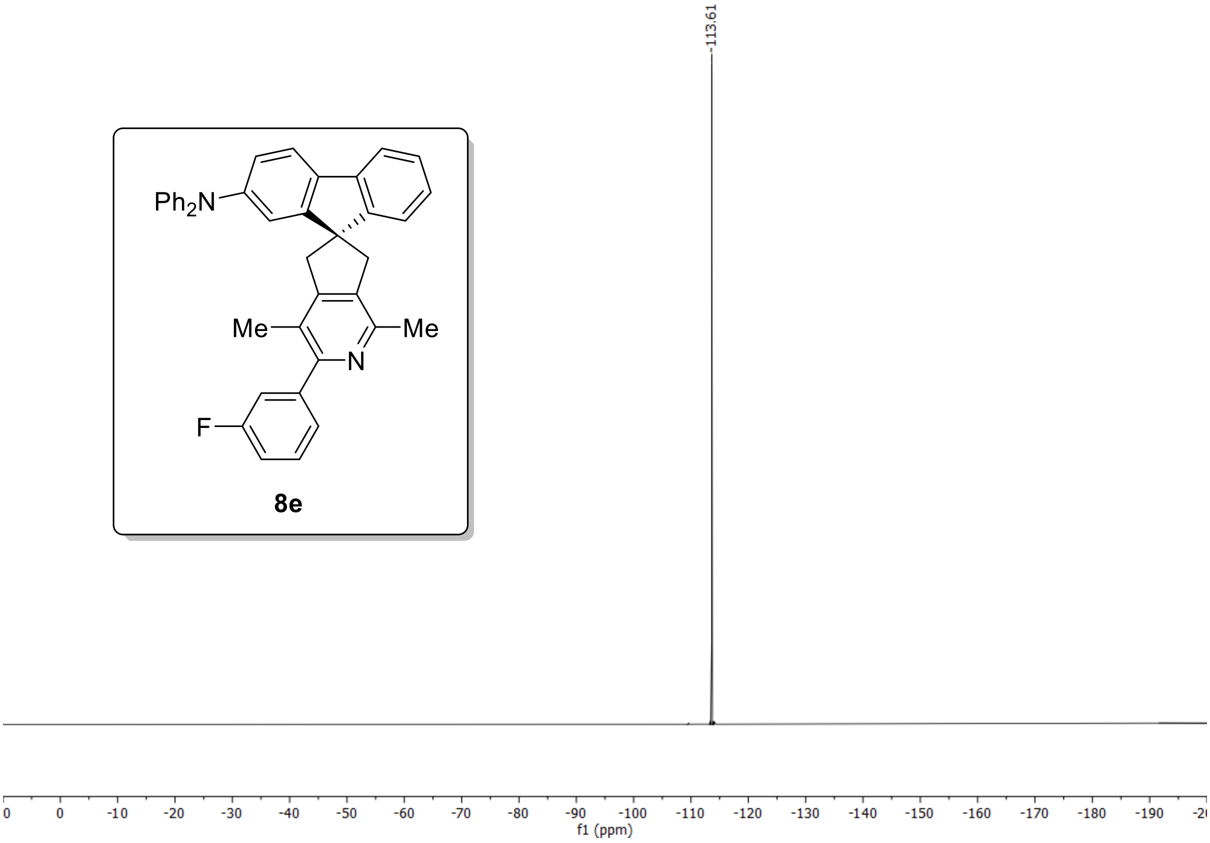
-113.61
Ph2N
Me
Me
N
F
8e
f1 (ppm)

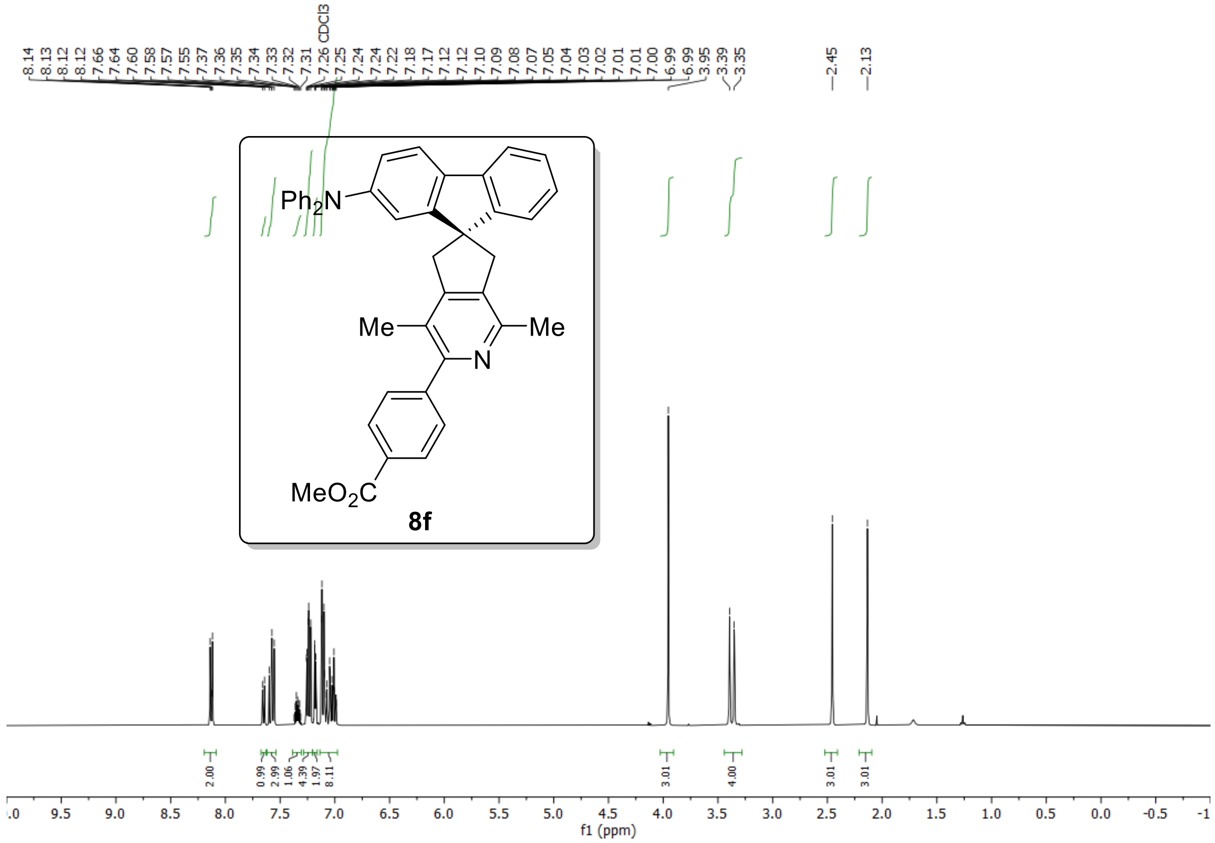
8.14
8.13
8.12
8.12
7.66
7.64
7.60
7.58
7.57
7.55
7.37
7.36
7.35
7.34
7.33
7.32
7.31
7.26 CDCl3
7.25
7.24
7.24
7.22
7.18
7.17
7.12
7.12
7.10
7.09
7.08
7.07
7.05
7.04
7.03
7.02
7.01
7.01
7.00
6.99
6.99
3.95
3.39
3.35
2.45
2.13
Ph2N
Me
Me
N
MeO2C
8f
2.00
0.99
2.99
1.06
4.39
1.97
8.11
3.01
4.00
3.01
3.01
f1 (ppm)

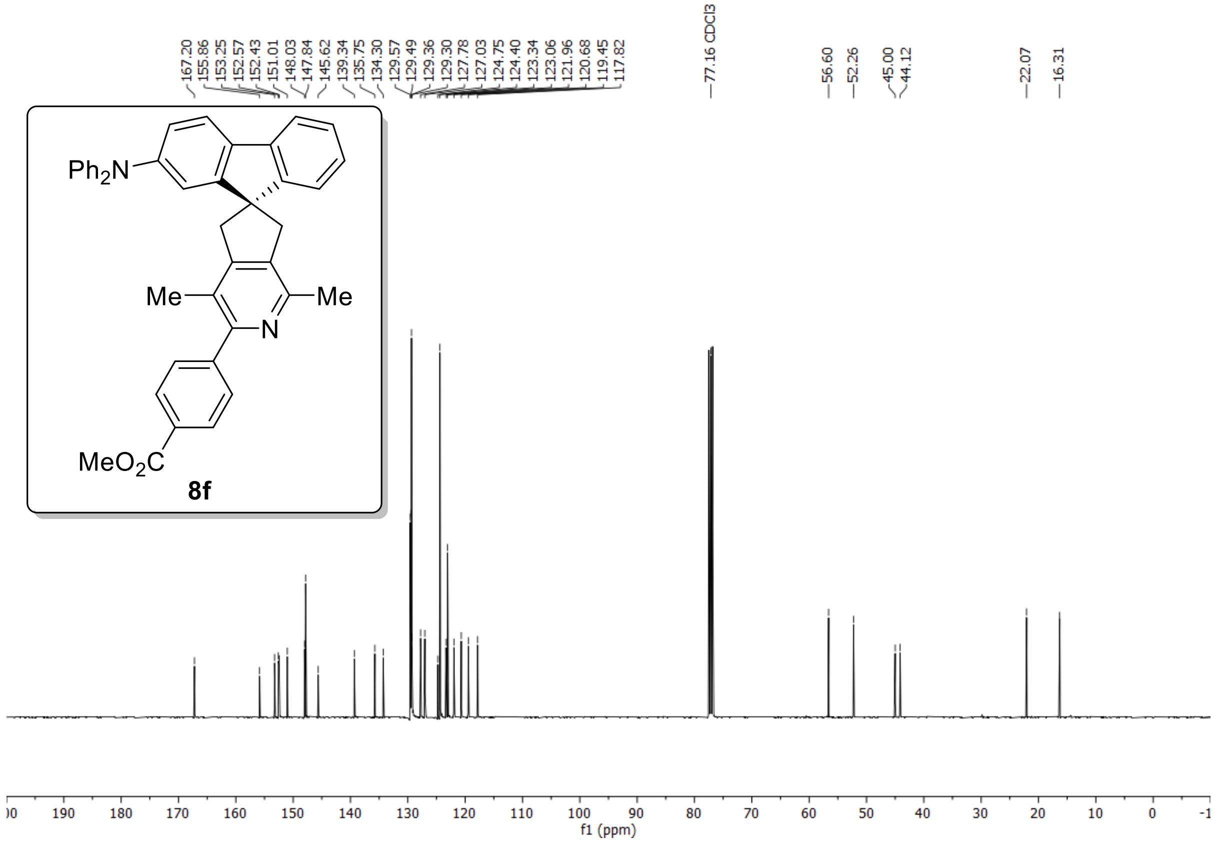
167.20
155.86
153.25
152.57
152.43
151.01
148.03
147.84
145.62
139.34
135.75
134.30
129.57
129.49
129.36
129.30
127.78
127.03
124.75
124.40
123.34
123.06
121.96
120.68
119.45
117.82
77.16 CDCl3
56.60
52.26
45.00
44.12
22.07
16.31
Ph2N
Me
Me
N
MeO2C
8f
f1 (ppm)

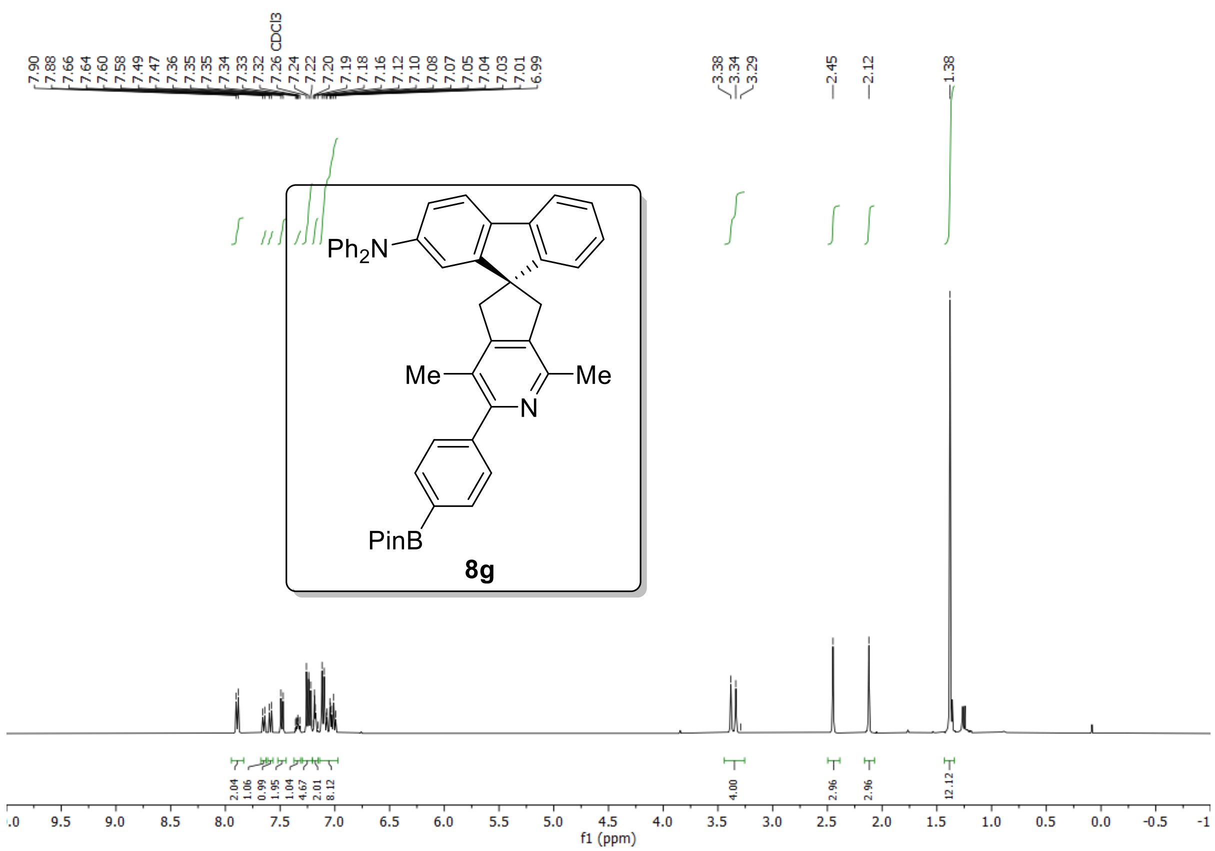
7.90
7.88
7.66
7.64
7.60
7.58
7.49
7.47
7.36
7.35
7.35
7.34
7.33
7.32
7.26 CDCl3
7.24
7.22
7.20
7.19
7.18
7.16
7.12
7.10
7.08
7.07
7.05
7.04
7.03
7.01
6.99
3.38
3.34
3.29
2.45
2.12
1.38
Ph2N
Me
Me
N
PinB
8g
2.04
1.06
0.99
1.95
1.04
4.67
2.01
8.12
4.00
2.96
2.96
12.12
f1 (ppm)

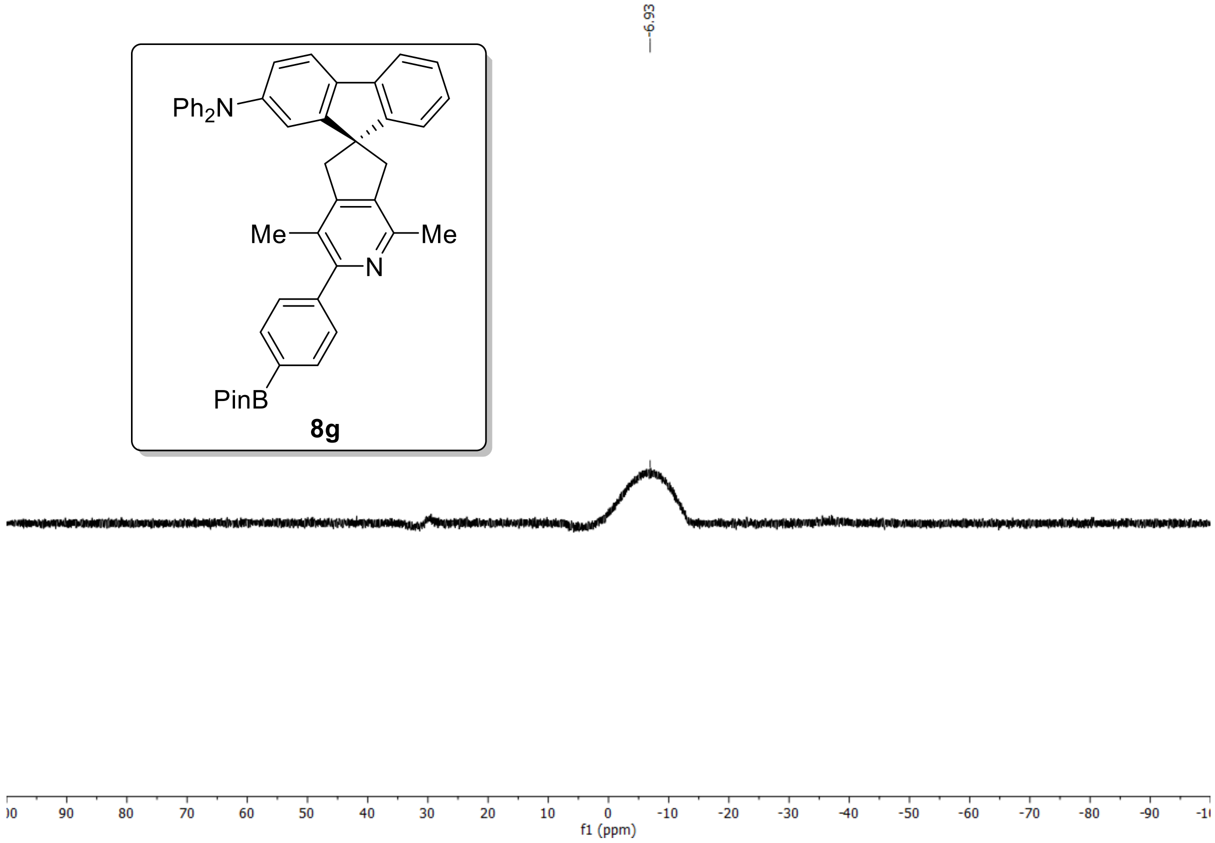
-6.93
Ph2N
Me
Me
N
PinB
8g
f1 (ppm)

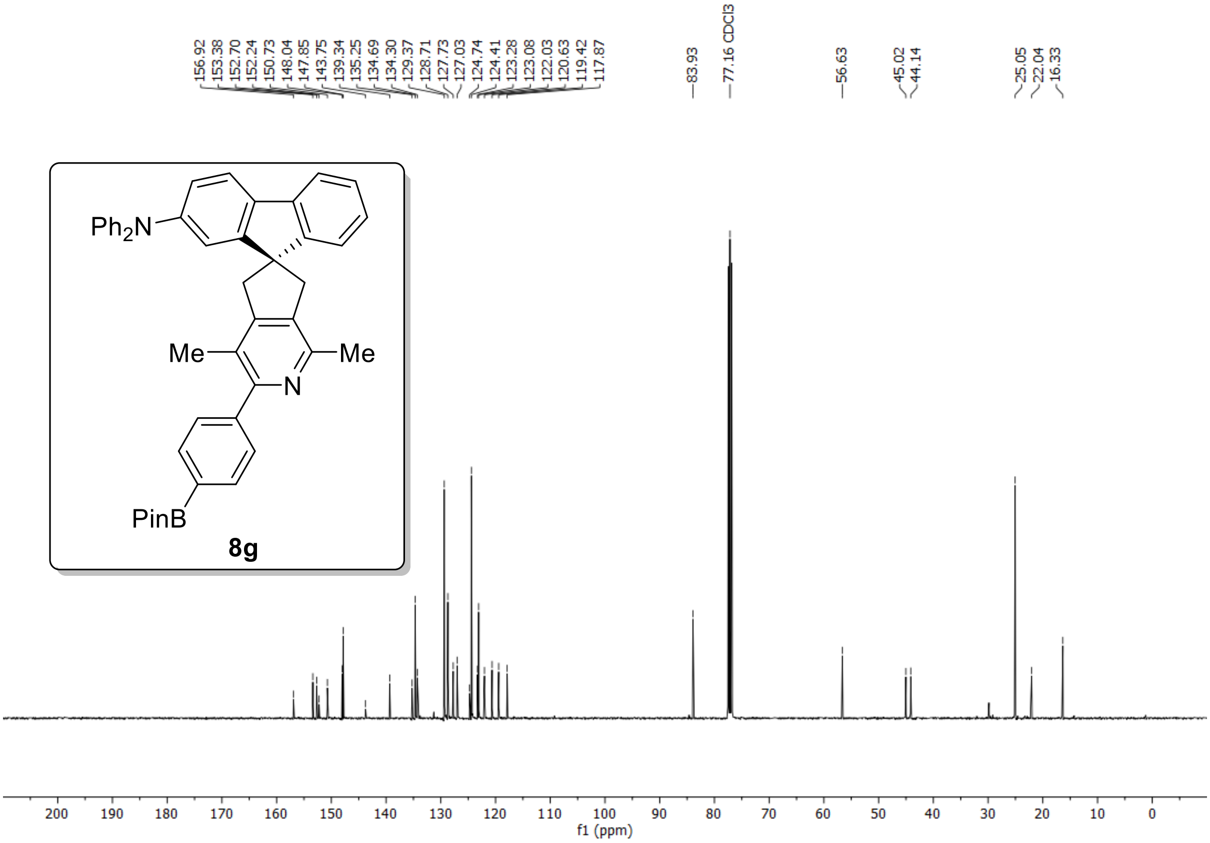
156.92
153.38
152.70
152.24
150.73
148.04
147.85
143.75
139.34
135.25
134.69
134.30
129.37
128.71
127.73
127.03
124.74
124.41
123.28
123.08
122.03
120.63
119.42
117.87
83.93
77.16 CDCl3
56.63
45.02
44.14
25.05
22.04
16.33
Ph2N
Me
Me
N
PinB
8g
f1 (ppm)

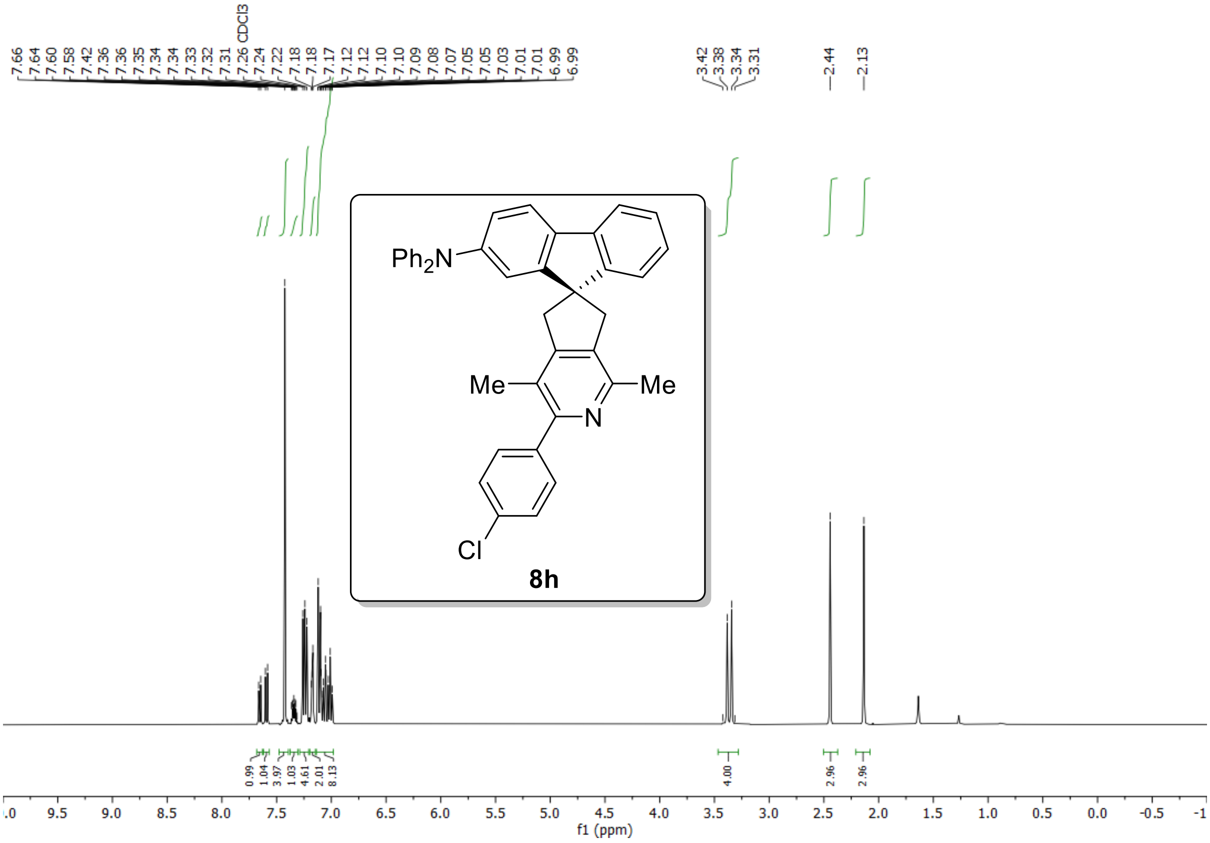
7.66
7.64
7.60
7.58
7.42
7.36
7.36
7.35
7.34
7.34
7.33
7.32
7.31
7.26 CDCl3
7.24
7.22
7.18
7.18
7.17
7.12
7.10
7.10
7.09
7.08
7.07
7.05
7.05
7.03
7.01
7.01
6.99
6.99
3.42
3.38
3.34
3.31
2.44
2.13
Ph2N
Me
Me
N
Cl
8h
0.99
1.04
3.97
1.03
4.61
2.01
8.13
4.00
2.96
2.96
f1 (ppm)

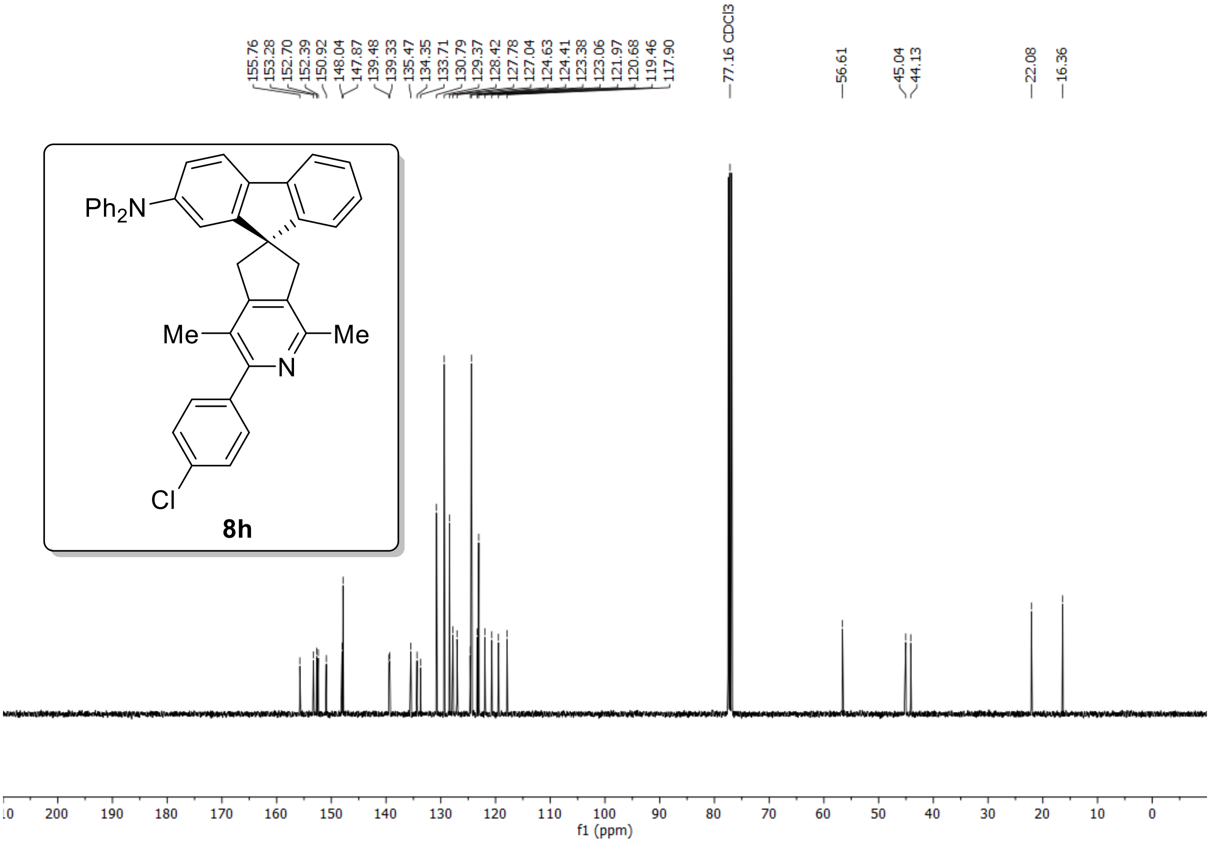
155.76
153.28
152.70
152.39
150.92
148.04
147.87
139.48
139.33
135.47
134.35
133.71
130.79
129.37
128.42
127.78
127.04
124.63
124.41
123.38
123.06
121.97
120.68
119.46
117.90
77.16 CDCl3
56.61
45.04
44.13
22.08
16.36
Ph2N
Me
Me
N
Cl
8h
f1 (ppm)

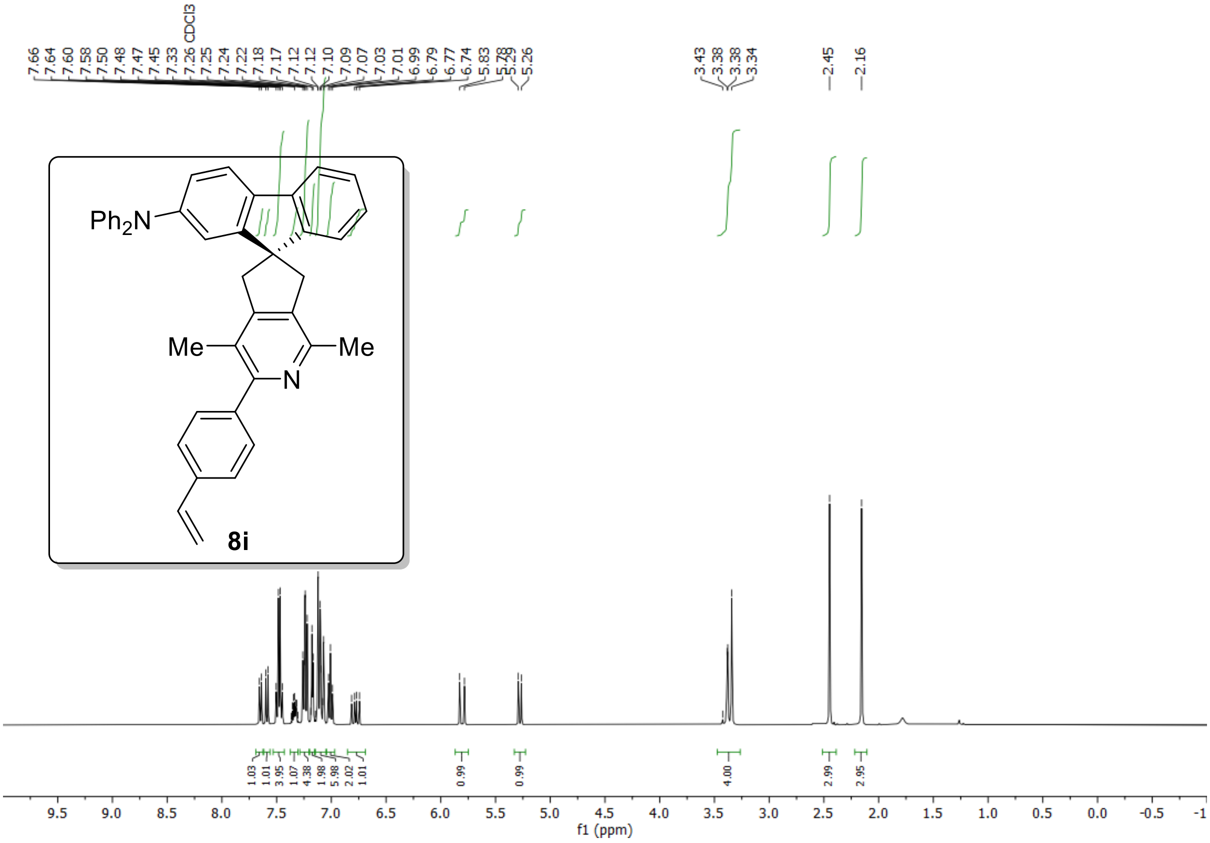
7.66
7.64
7.60
7.58
7.50
7.48
7.47
7.45
7.33
7.26 CDCl3
7.25
7.24
7.22
7.18
7.17
7.12
7.12
7.10
7.09
7.07
7.03
7.01
6.99
6.79
6.77
6.74
5.83
5.78
5.29
5.26
3.43
3.38
3.38
3.34
2.45
2.16
Ph2N
Me
Me
N
8i
1.03
1.01
3.95
1.07
4.38
1.98
5.98
2.02
1.01
0.99
0.99
4.00
2.99
2.95
f1 (ppm)

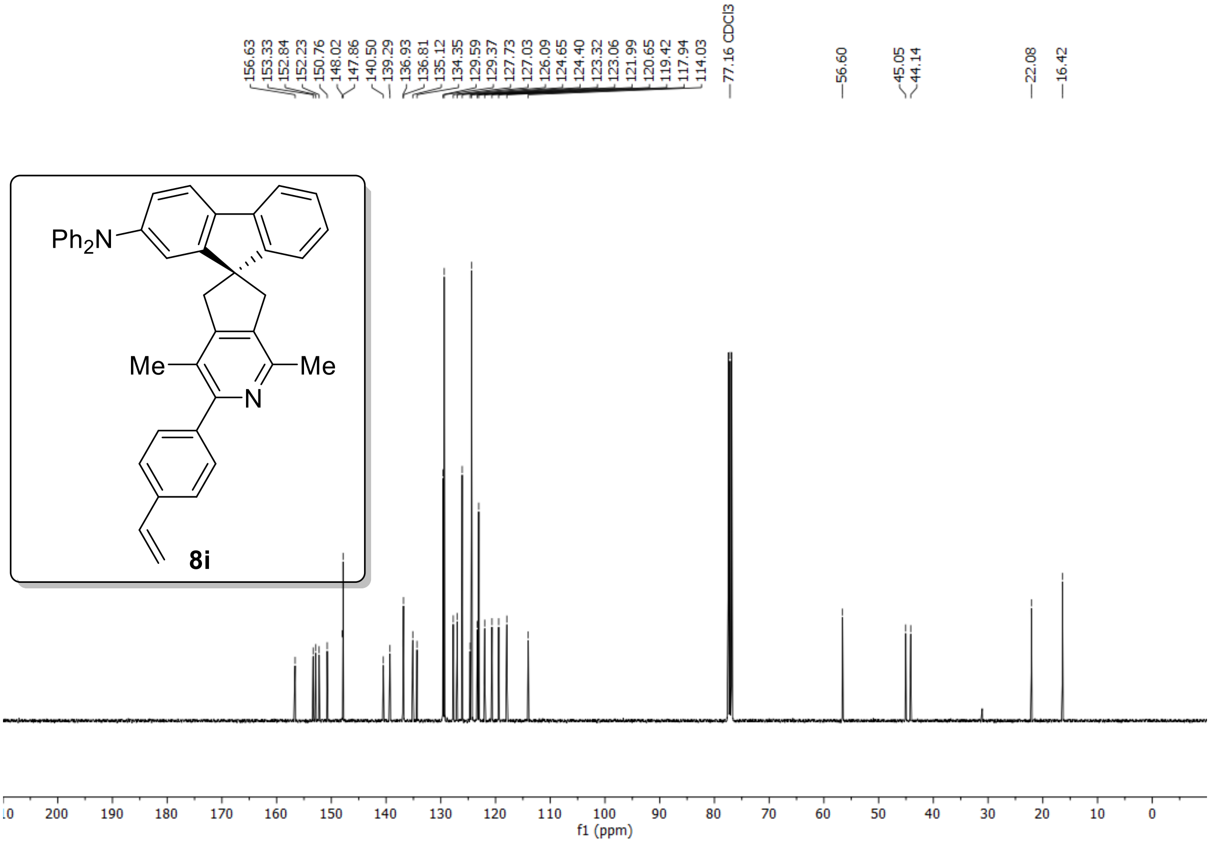
156.63 153.33 152.84 152.23 150.76 148.02 147.86 140.50 139.29 136.93 136.81 135.12 134.35 129.59 129.37 127.73 127.03 126.09 124.65 124.40 123.32 123.06 121.99 120.65 119.42 117.94 114.03
77.16 CDCl3
56.60
45.05 44.14
22.08
16.42
Ph2N
Me
Me
N
8i
f1 (ppm)

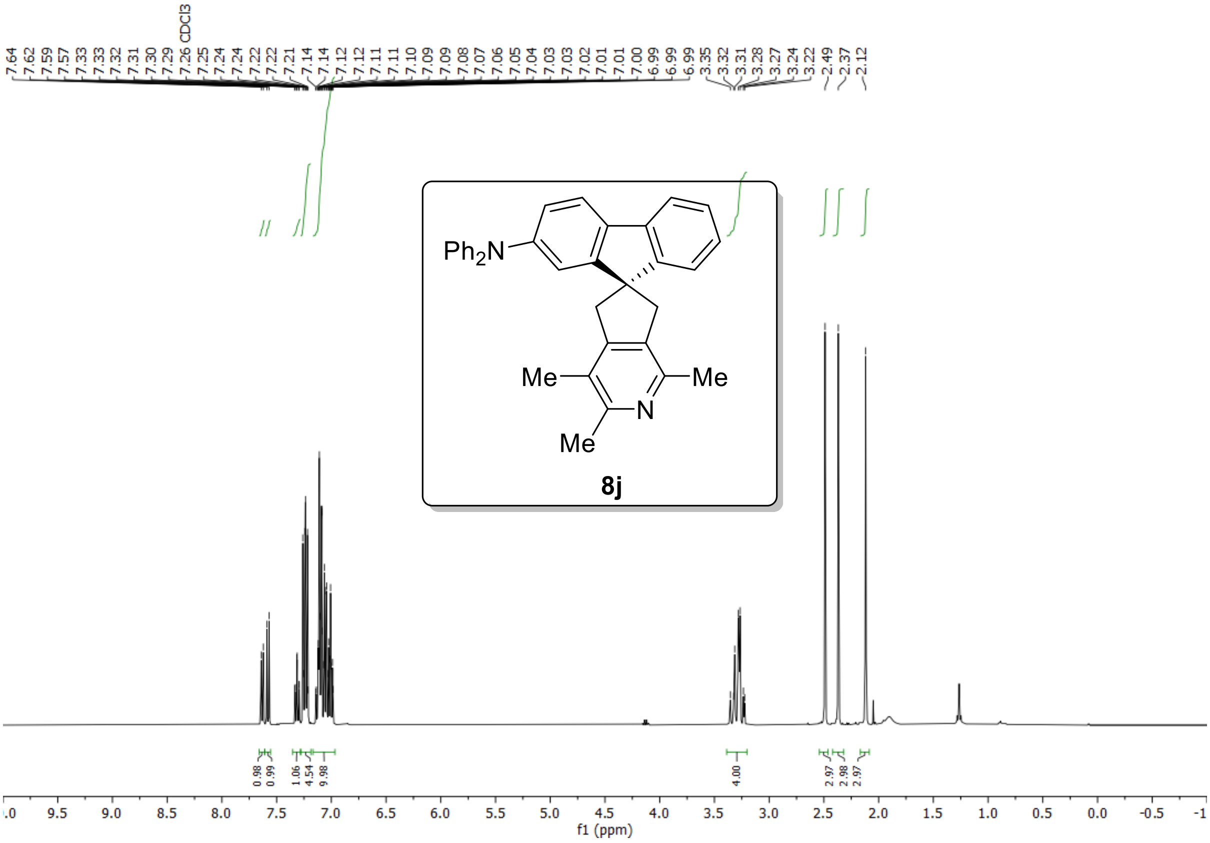
7.64 7.62 7.59 7.57 7.33 7.33 7.32 7.31 7.30 7.29 7.26 CDCl3 7.25 7.24 7.24 7.22 7.22 7.21 7.14 7.14 7.12 7.12 7.11 7.11 7.10 7.09 7.09 7.08 7.07 7.06 7.05 7.04 7.03 7.03 7.02 7.01 7.01 7.00 6.99 6.99 3.35 3.32 3.31 3.28 3.27 3.24 3.22 2.49 2.37 2.12
Ph2N
Me
Me
N
Me
8j
0.98 0.99 1.06 4.54 9.98 4.00 2.97 2.98 2.97
f1 (ppm)

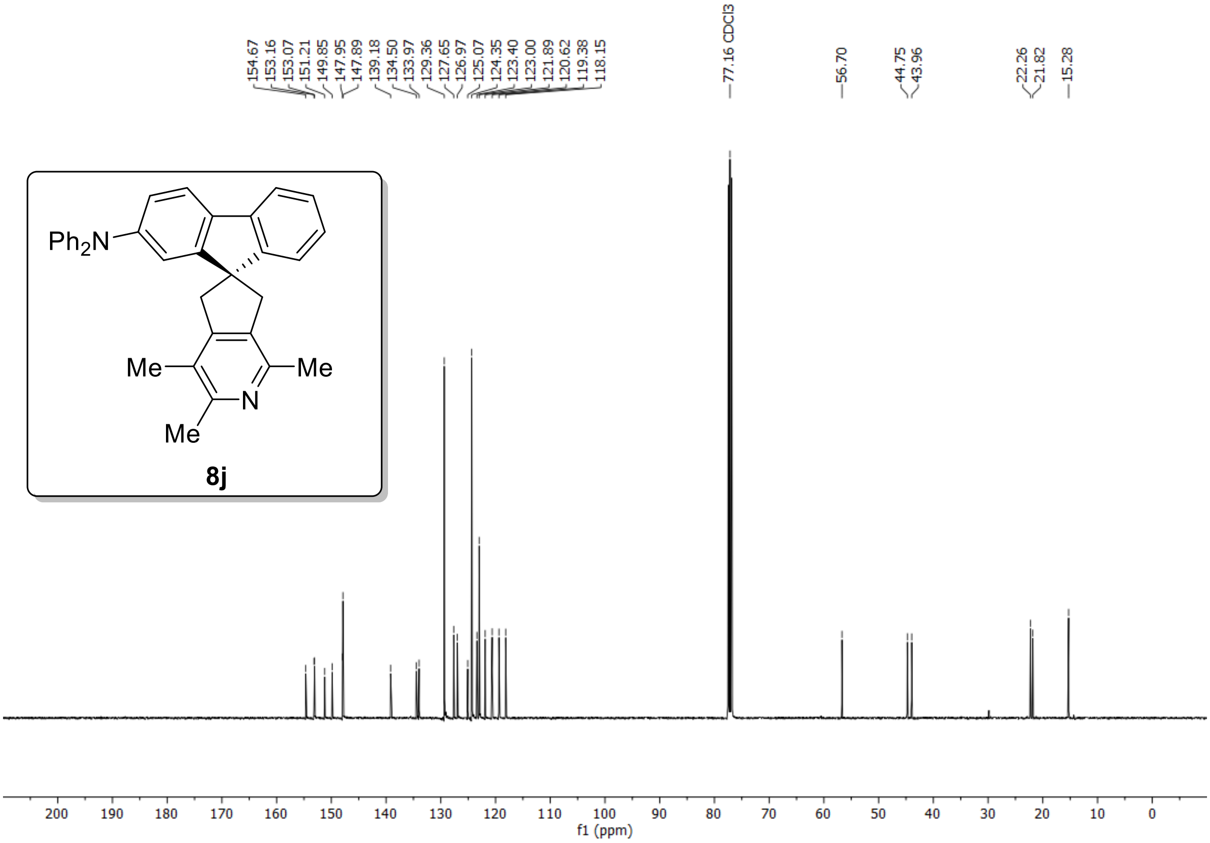
154.67
153.16
153.07
151.21
149.85
147.95
147.89
139.18
134.50
133.97
129.36
127.65
126.97
125.07
124.35
123.40
123.00
121.89
120.62
119.38
118.15
77.16 CDCl3
56.70
44.75
43.96
22.26
21.82
15.28
Ph2N
Me
Me
N
Me
8j
f1 (ppm)

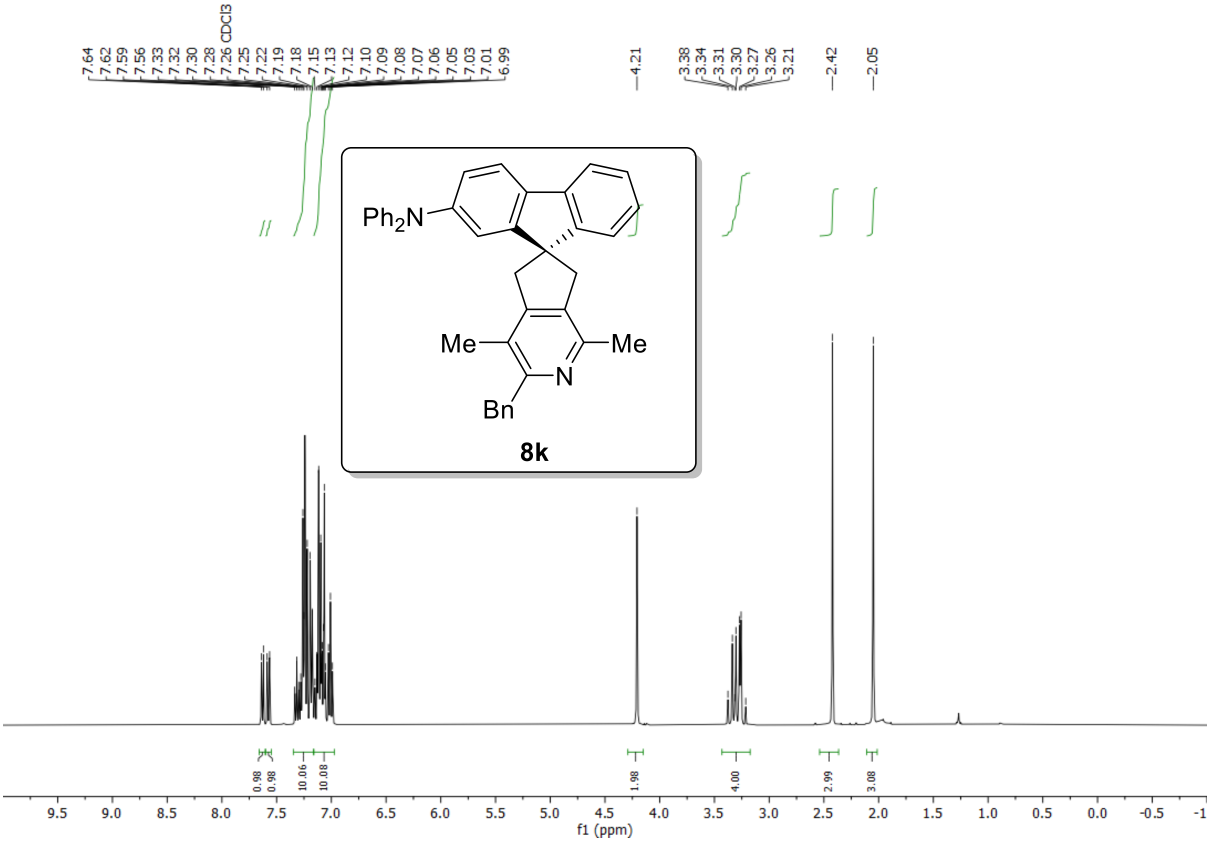
7.64
7.62
7.59
7.56
7.33
7.32
7.30
7.28
7.26 CDCl3
7.25
7.22
7.19
7.18
7.15
7.13
7.12
7.10
7.09
7.08
7.07
7.06
7.05
7.03
7.01
6.99
4.21
3.38
3.34
3.31
3.30
3.27
3.26
3.21
2.42
2.05
Ph2N
Me
Me
N
Bn
8k
0.98
0.98
10.06
10.08
1.98
4.00
2.99
3.08
f1 (ppm)

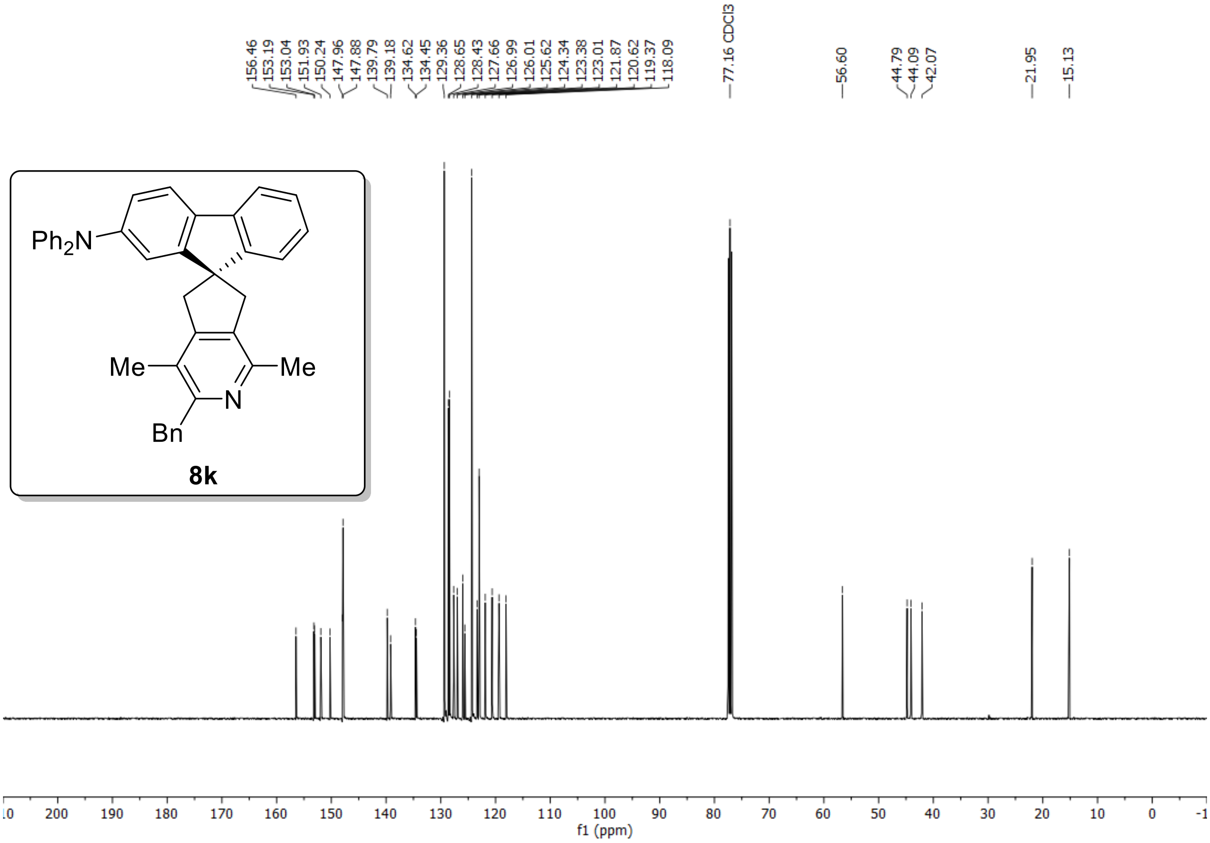
156.46
153.19
153.04
151.93
150.24
147.96
147.88
139.79
139.18
134.62
134.45
129.36
128.65
128.43
127.66
126.99
126.01
125.62
124.34
123.38
123.01
121.87
120.62
119.37
118.09
77.16 CDCl3
56.60
44.79
44.09
42.07
21.95
15.13
Ph2N
Me
Me
N
Bn
8k
f1 (ppm)

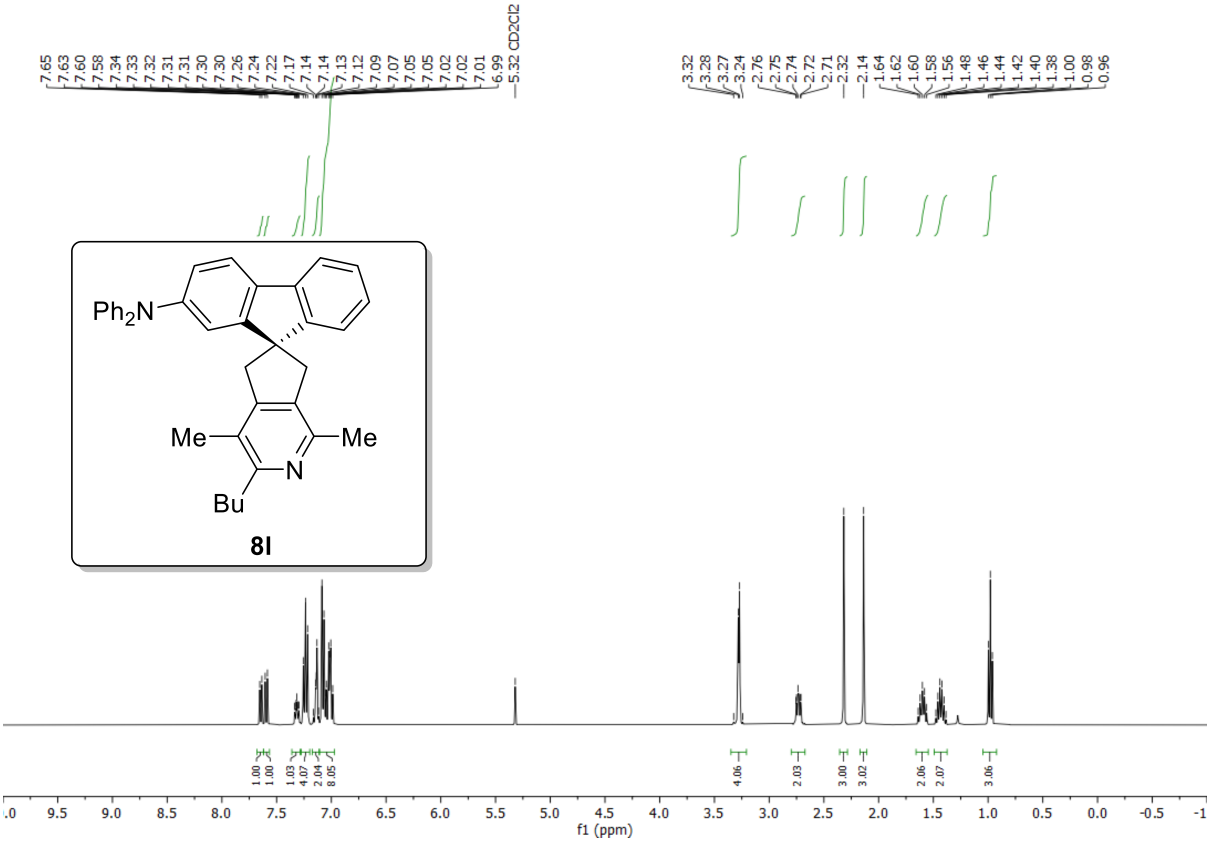
7.65
7.63
7.60
7.58
7.34
7.33
7.32
7.31
7.31
7.30
7.30
7.26
7.24
7.22
7.17
7.14
7.14
7.13
7.12
7.09
7.07
7.05
7.02
7.02
7.01
6.99
5.32 CD2Cl2
3.32
3.28
3.27
3.24
2.76
2.75
2.74
2.72
2.71
2.32
2.14
1.64
1.62
1.60
1.58
1.56
1.48
1.46
1.44
1.42
1.40
1.38
1.00
0.98
0.96
Ph2N
Me
Me
N
Bu
8l
1.00
1.00
1.03
4.07
2.04
8.05
4.06
2.03
3.00
3.02
2.06
2.07
3.06
f1 (ppm)

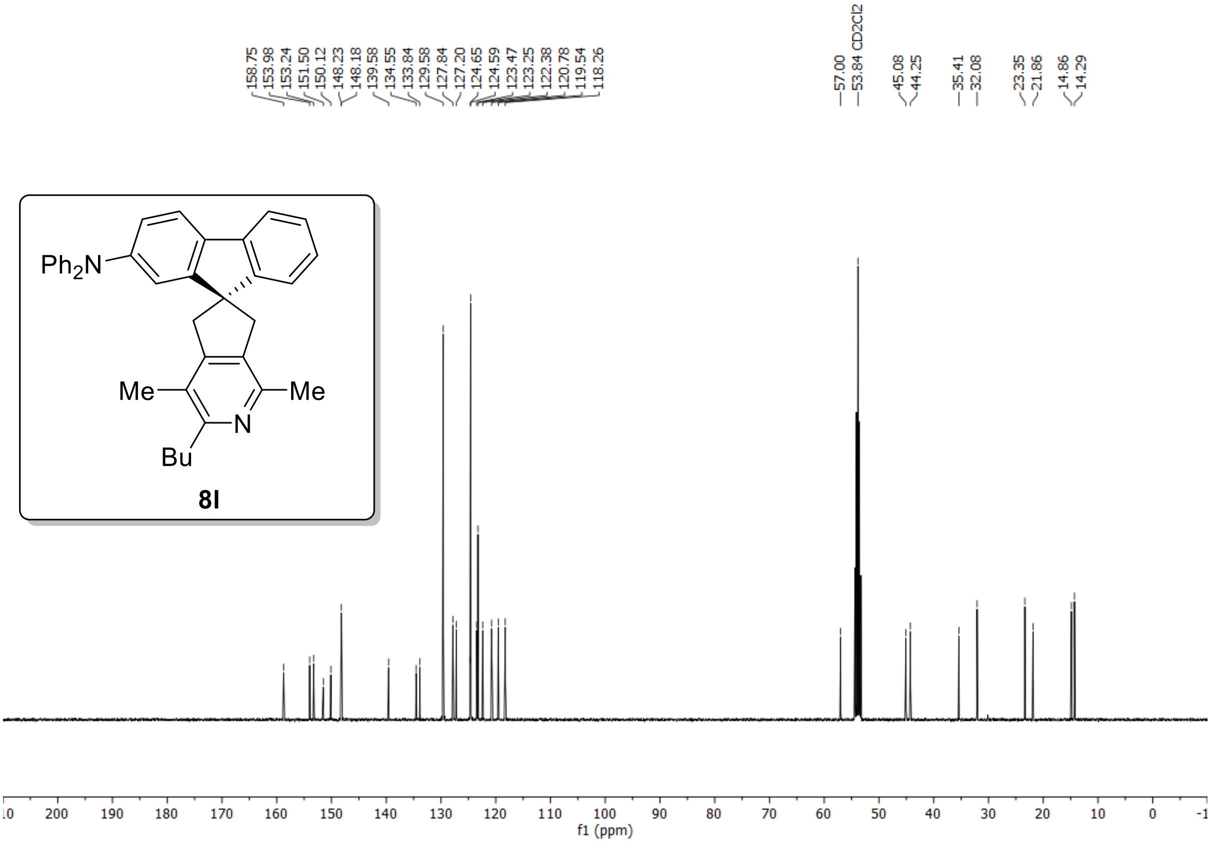
158.75
153.98
153.24
151.50
150.12
148.23
148.18
139.58
134.55
133.84
129.58
127.84
127.20
124.65
124.59
123.47
123.25
122.38
120.78
119.54
118.26
57.00
53.84 CD2Cl2
45.08
44.25
35.41
32.08
23.35
21.86
14.86
14.29
Ph2N
Me
Me
N
Bu
8l
f1 (ppm)

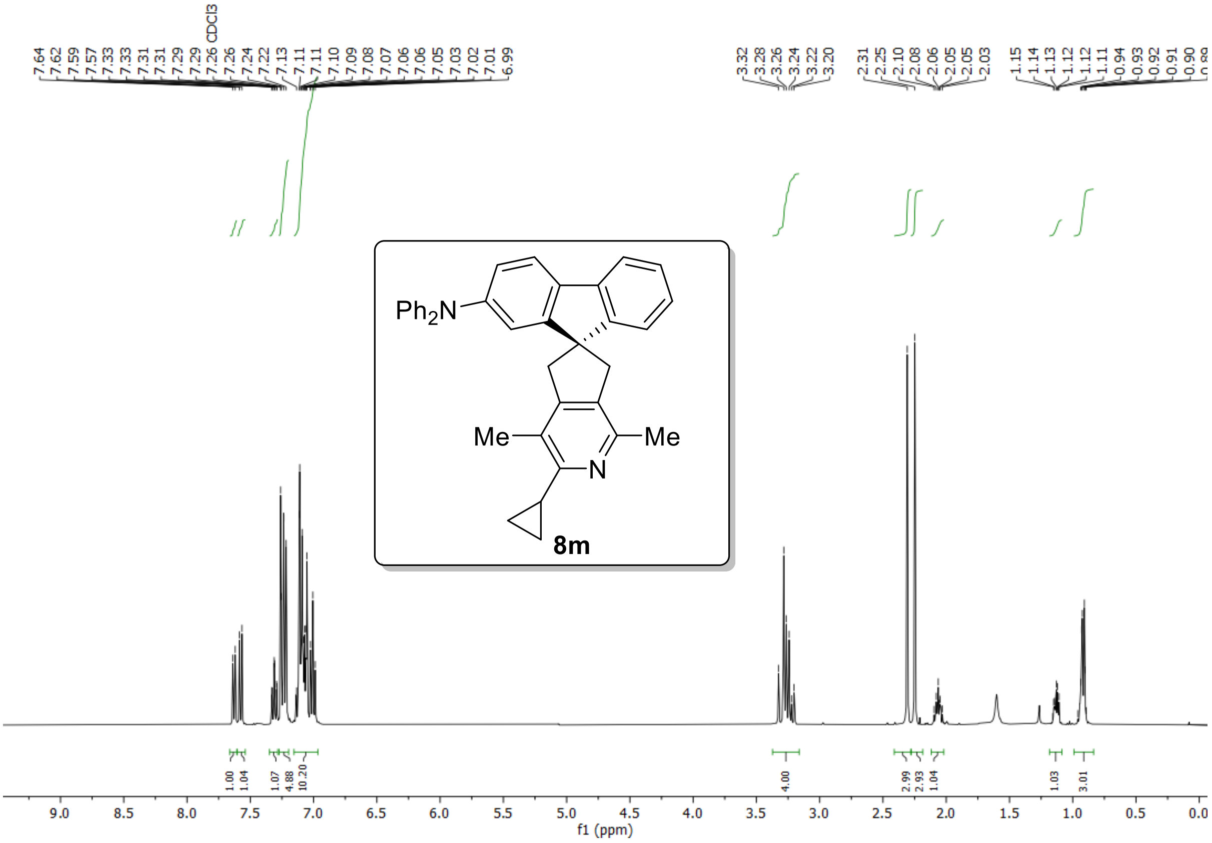
CDCl3
Ph2N
Me
Me
N
8m
1.00
1.04
1.07
4.88
10.20
4.00
2.99
2.93
1.04
1.03
3.01
f1 (ppm)

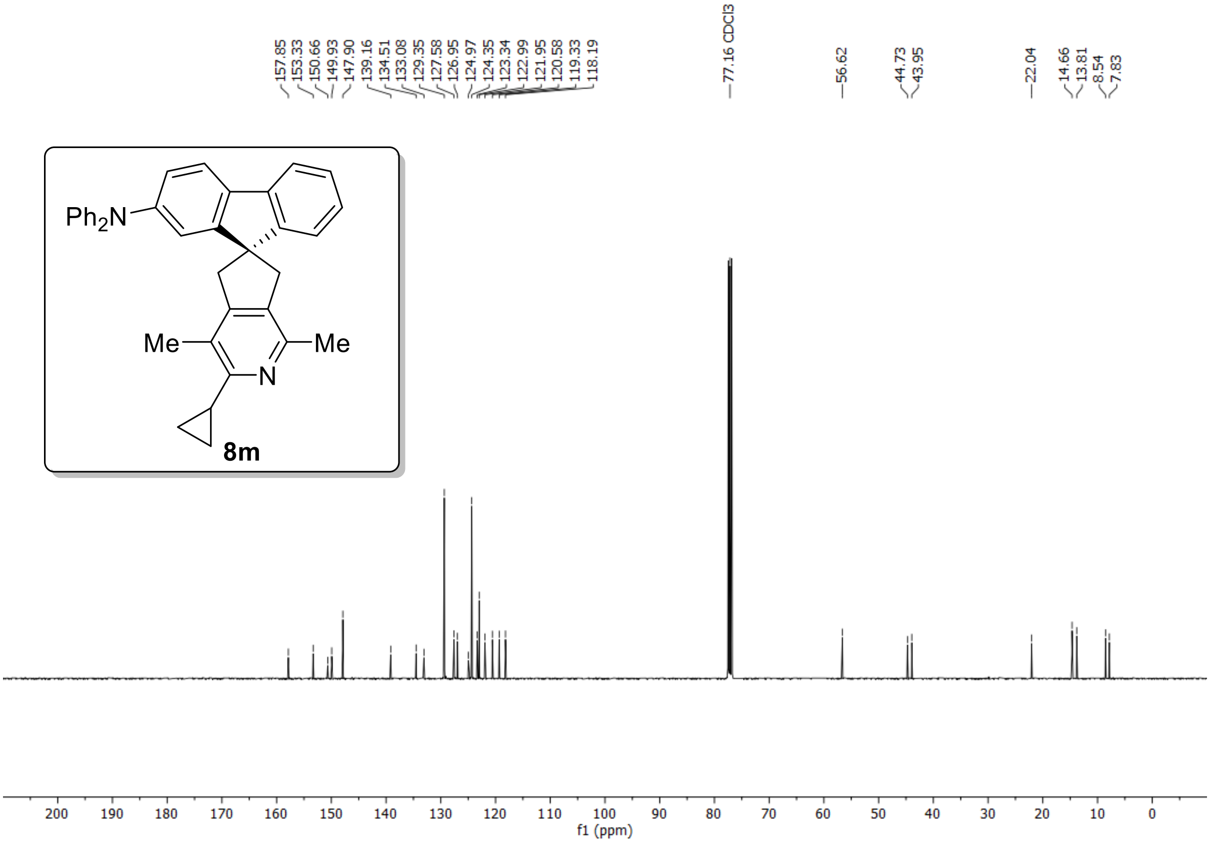
157.85
153.33
150.66
149.93
147.90
139.16
134.51
133.08
129.35
127.58
126.95
124.97
124.35
123.34
122.99
121.95
120.58
119.33
118.19
77.16 CDCl3
56.62
44.73
43.95
22.04
14.66
13.81
8.54
7.83
Ph2N
Me
Me
N
8m
f1 (ppm)

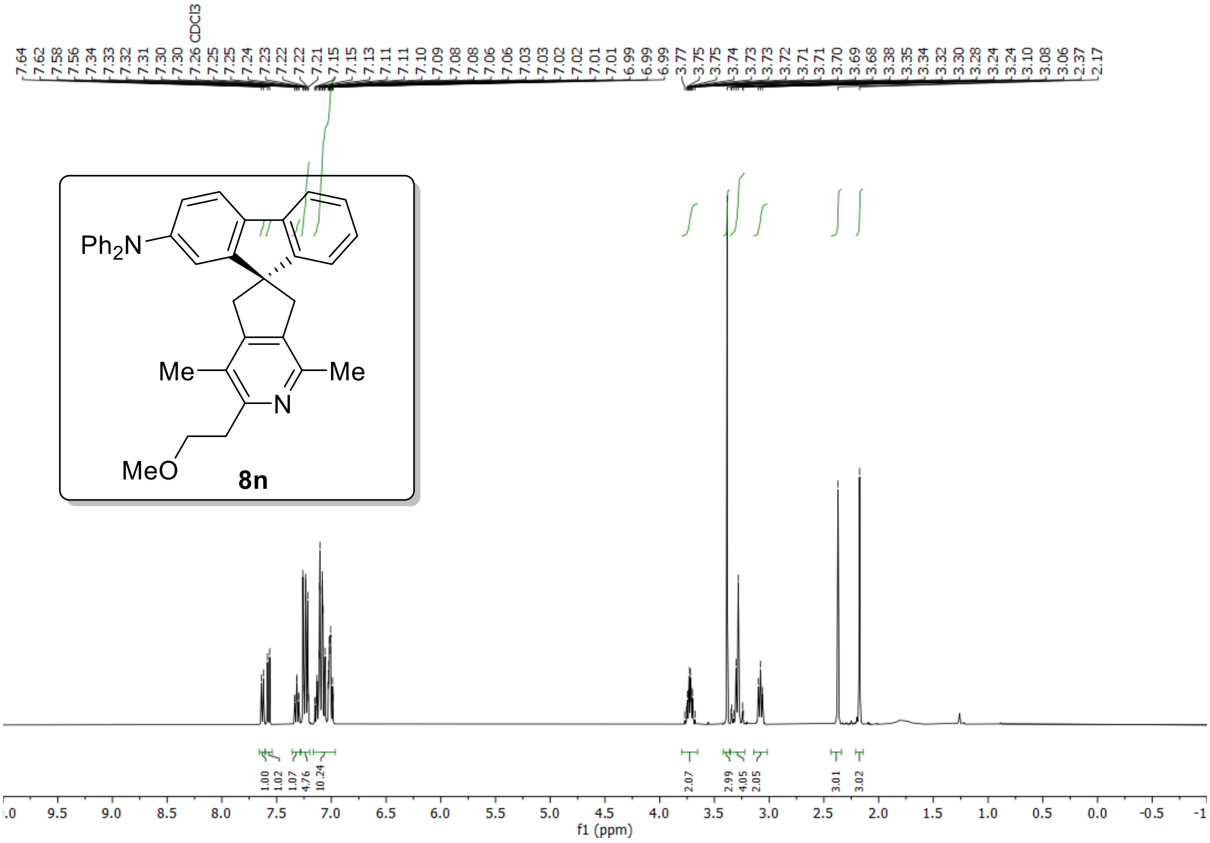
7.64
7.62
7.58
7.56
7.34
7.33
7.32
7.31
7.30
7.30
7.26 CDCl3
7.25
7.25
7.24
7.23
7.22
7.22
7.21
7.15
7.15
7.13
7.11
7.11
7.10
7.09
7.08
7.08
7.06
7.06
7.03
7.03
7.02
7.02
7.01
7.01
6.99
6.99
6.99
3.77
3.75
3.75
3.74
3.73
3.73
3.72
3.71
3.71
3.70
3.69
3.68
3.38
3.35
3.34
3.32
3.30
3.28
3.24
3.24
3.10
3.08
3.06
2.37
2.17
Ph2N
Me
Me
N
MeO
8n
1.00
1.02
1.07
4.76
10.24
2.07
2.99
4.05
2.05
3.01
3.02
f1 (ppm)

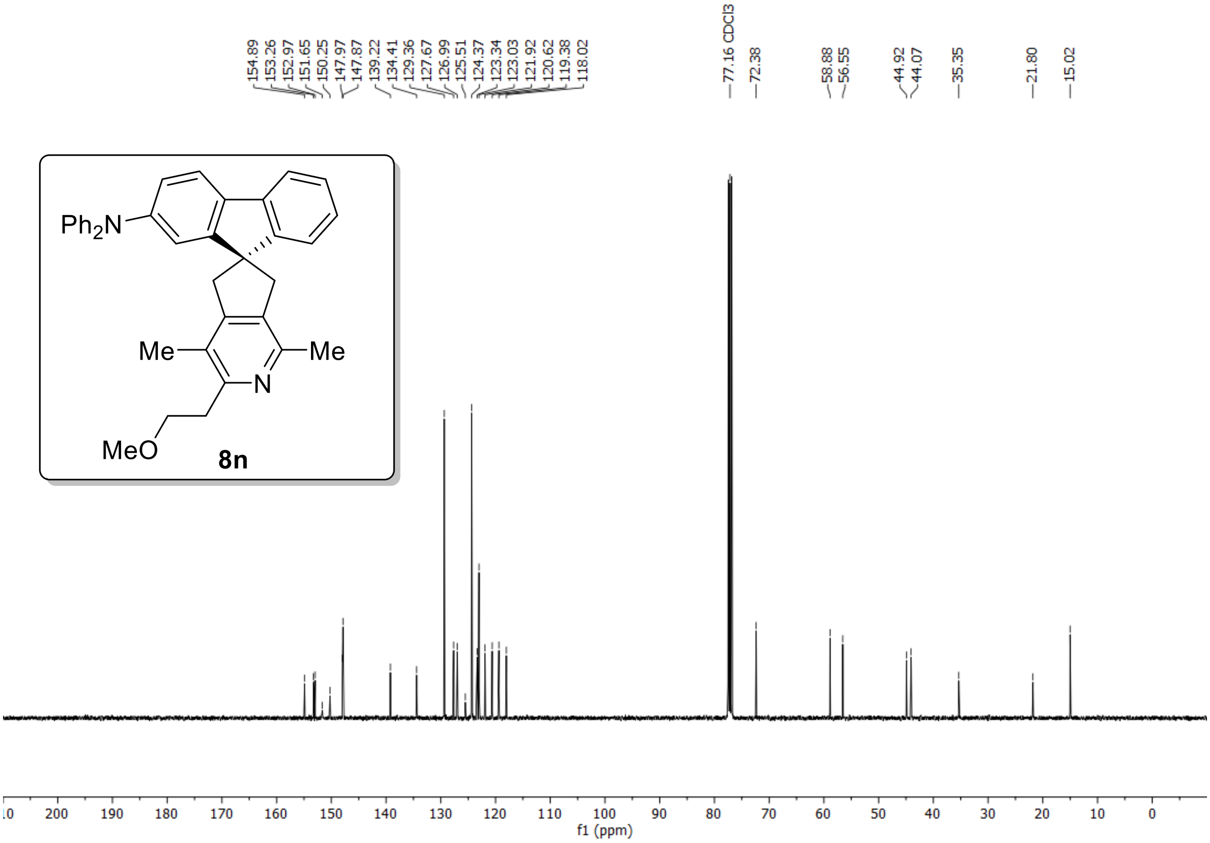
154.89
153.26
152.97
151.65
150.25
147.97
147.87
139.22
134.41
129.36
127.67
126.99
125.51
124.37
123.34
123.03
121.92
120.62
119.38
118.02
77.16 CDCl3
72.38
58.88
56.55
44.92
44.07
35.35
21.80
15.02
Ph2N
Me
Me
N
MeO
8n
f1 (ppm)

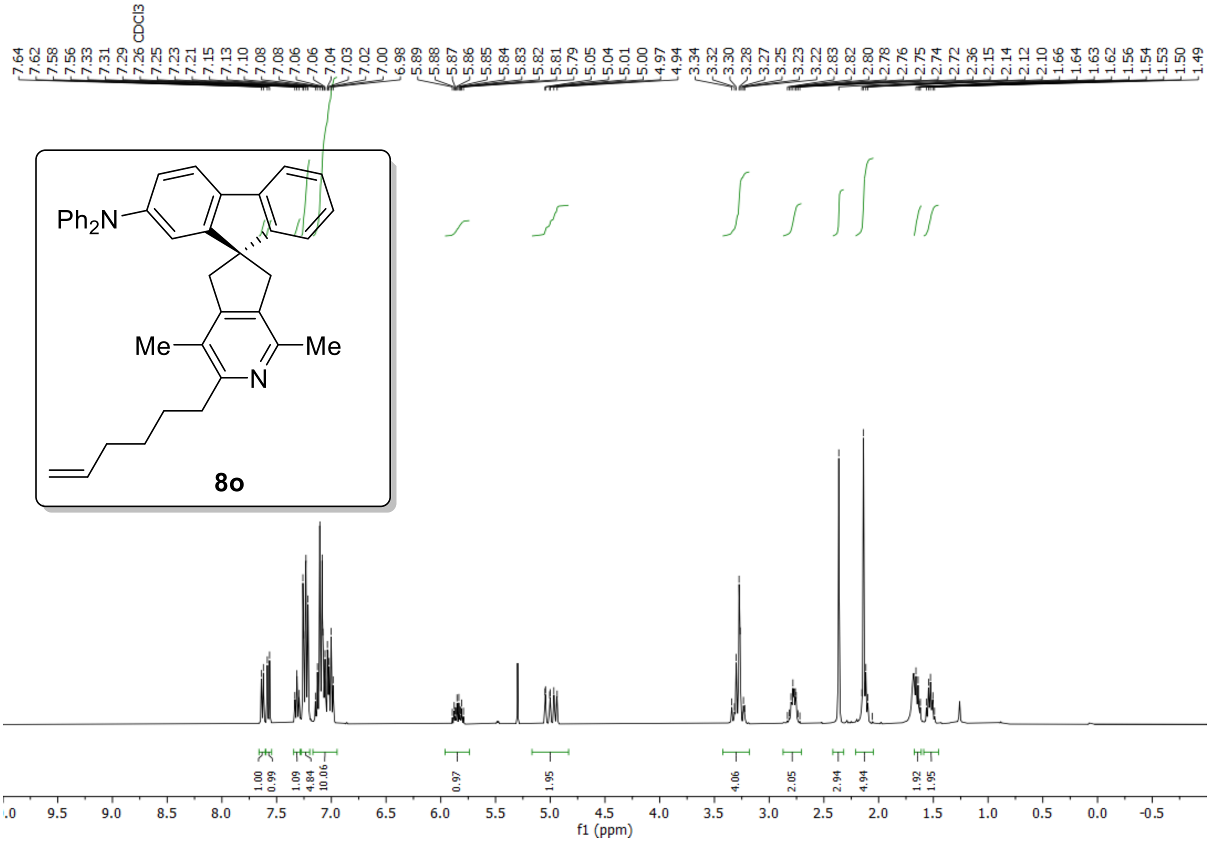
7.64
7.62
7.58
7.56
7.33
7.31
7.29
7.26 CDCl3
7.25
7.23
7.21
7.15
7.13
7.10
7.08
7.08
7.06
7.06
7.04
7.03
7.02
7.00
6.98
5.89
5.88
5.87
5.86
5.85
5.84
5.83
5.82
5.81
5.79
5.05
5.04
5.01
5.00
4.97
4.94
3.34
3.32
3.30
3.28
3.27
3.25
3.23
3.22
2.83
2.82
2.80
2.78
2.76
2.75
2.74
2.72
2.36
2.15
2.14
2.12
2.10
1.66
1.64
1.63
1.62
1.56
1.54
1.53
1.50
1.49
Ph2N
Me
Me
N
8o
1.00
0.99
1.09
4.84
10.06
0.97
1.95
4.06
2.05
2.94
4.94
1.92
1.95
f1 (ppm)

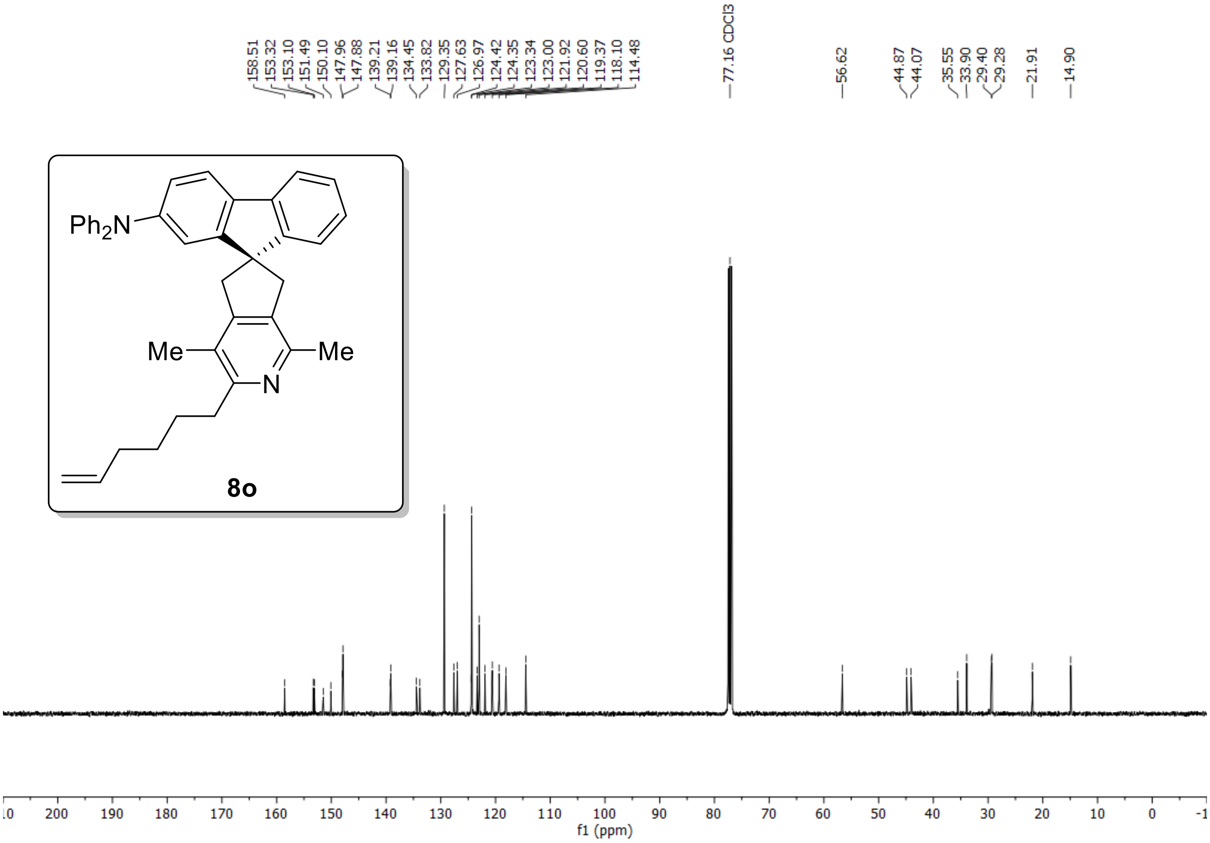
158.51
153.32
153.10
151.49
150.10
147.96
147.88
139.21
139.16
134.45
133.82
129.35
127.63
126.97
124.42
124.35
123.34
123.00
121.92
120.60
119.37
118.10
114.48
77.16 CDCl3
56.62
44.87
44.07
35.55
33.90
29.40
29.28
21.91
14.90
Ph2N
Me
Me
N
8o
f1 (ppm)

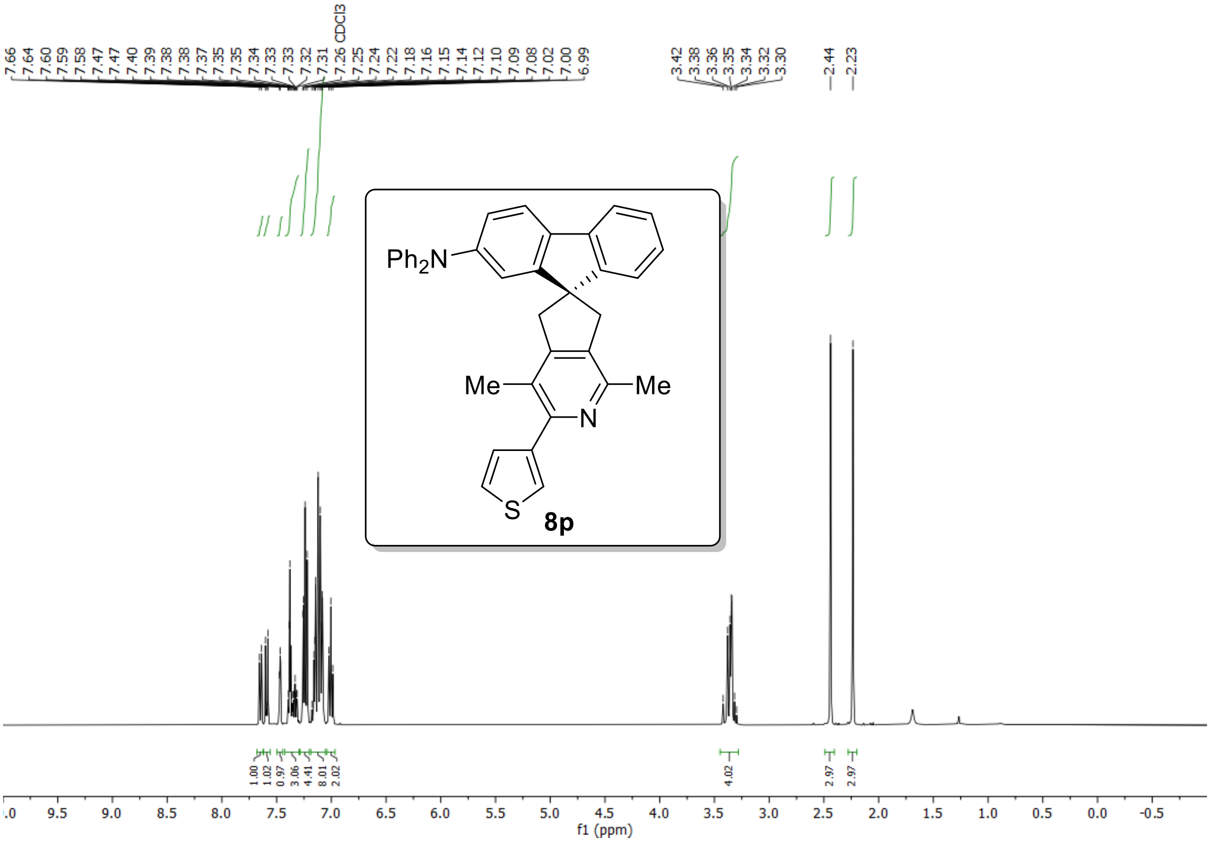
7.66
7.64
7.60
7.59
7.58
7.47
7.47
7.40
7.39
7.38
7.38
7.37
7.35
7.35
7.34
7.33
7.33
7.32
7.31
7.26 CDCl3
7.25
7.24
7.22
7.18
7.16
7.15
7.14
7.12
7.10
7.09
7.08
7.02
7.00
6.99
3.42
3.38
3.36
3.35
3.34
3.32
3.30
2.44
2.23
Ph2N
Me
Me
N
S
8p
1.00
1.02
0.97
3.06
4.41
8.01
2.02
4.02
2.97
2.97
f1 (ppm)

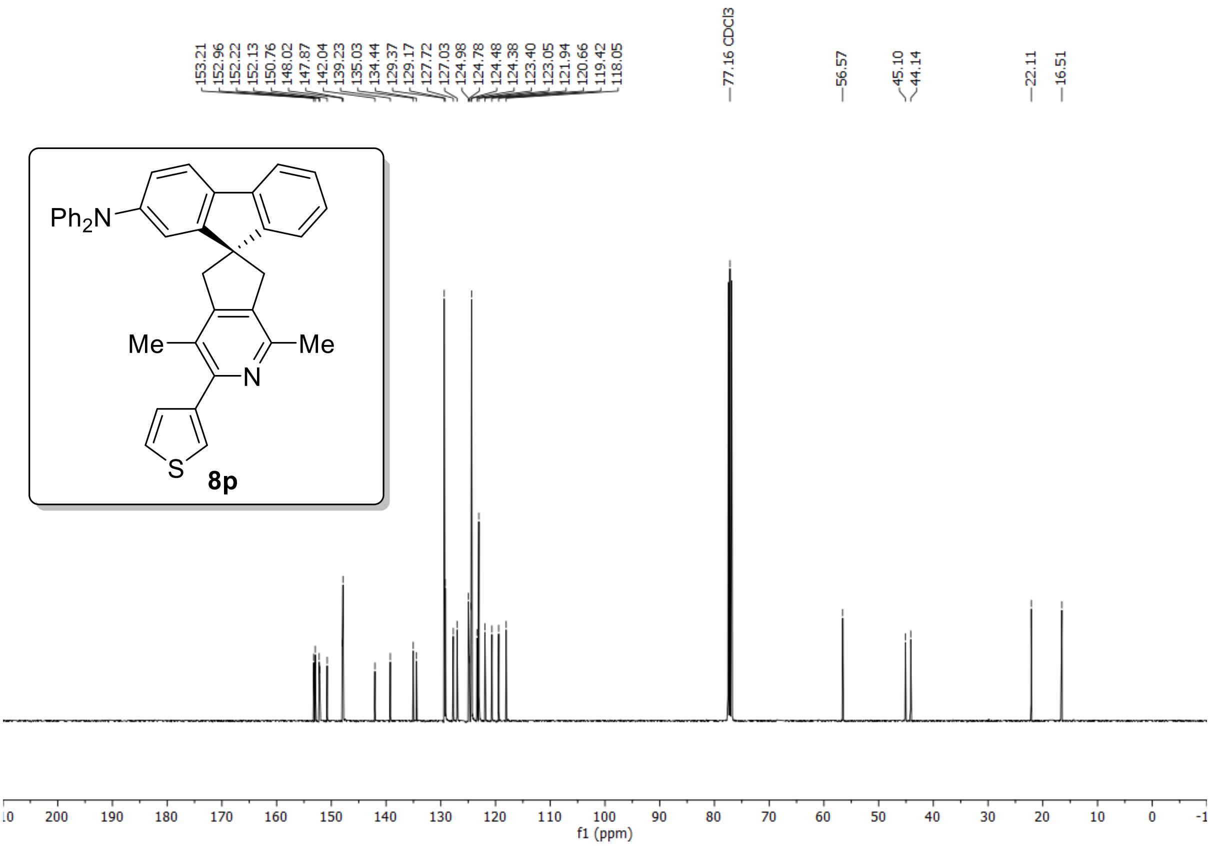

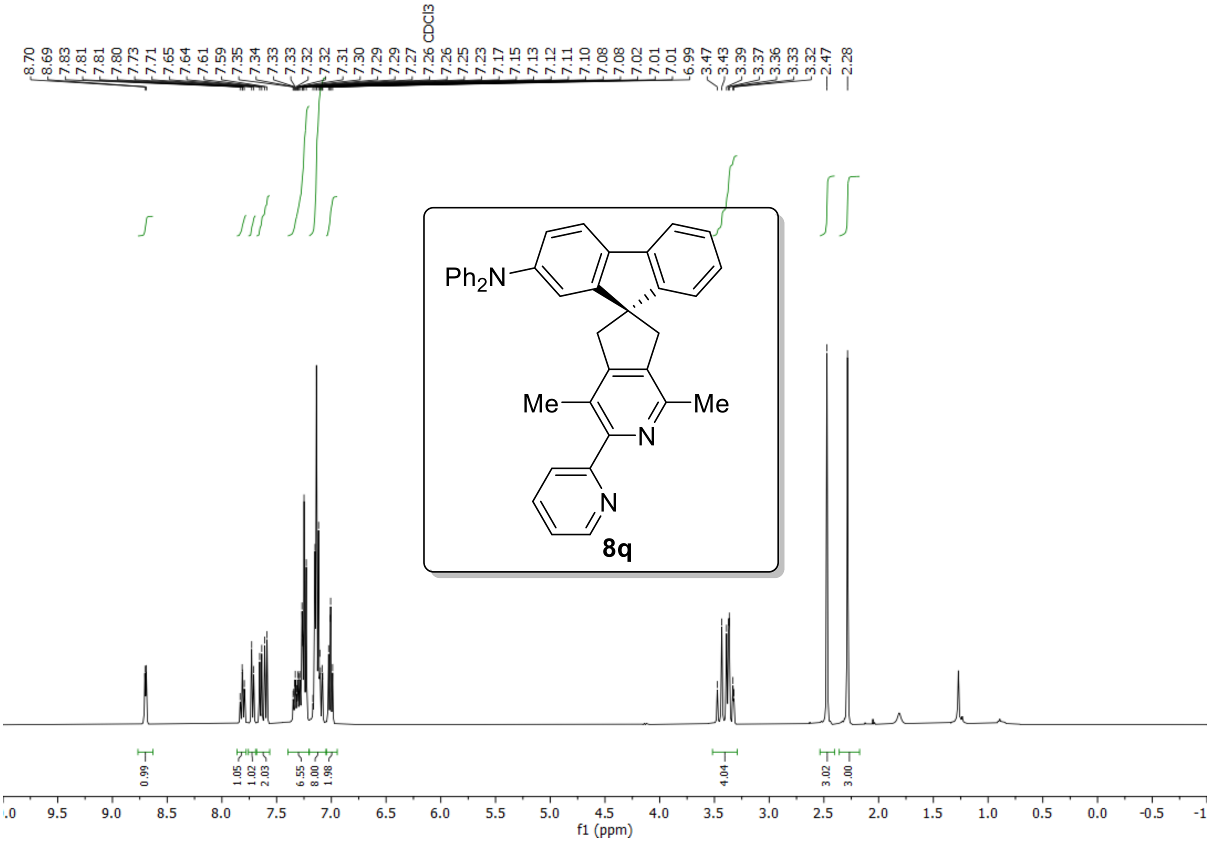

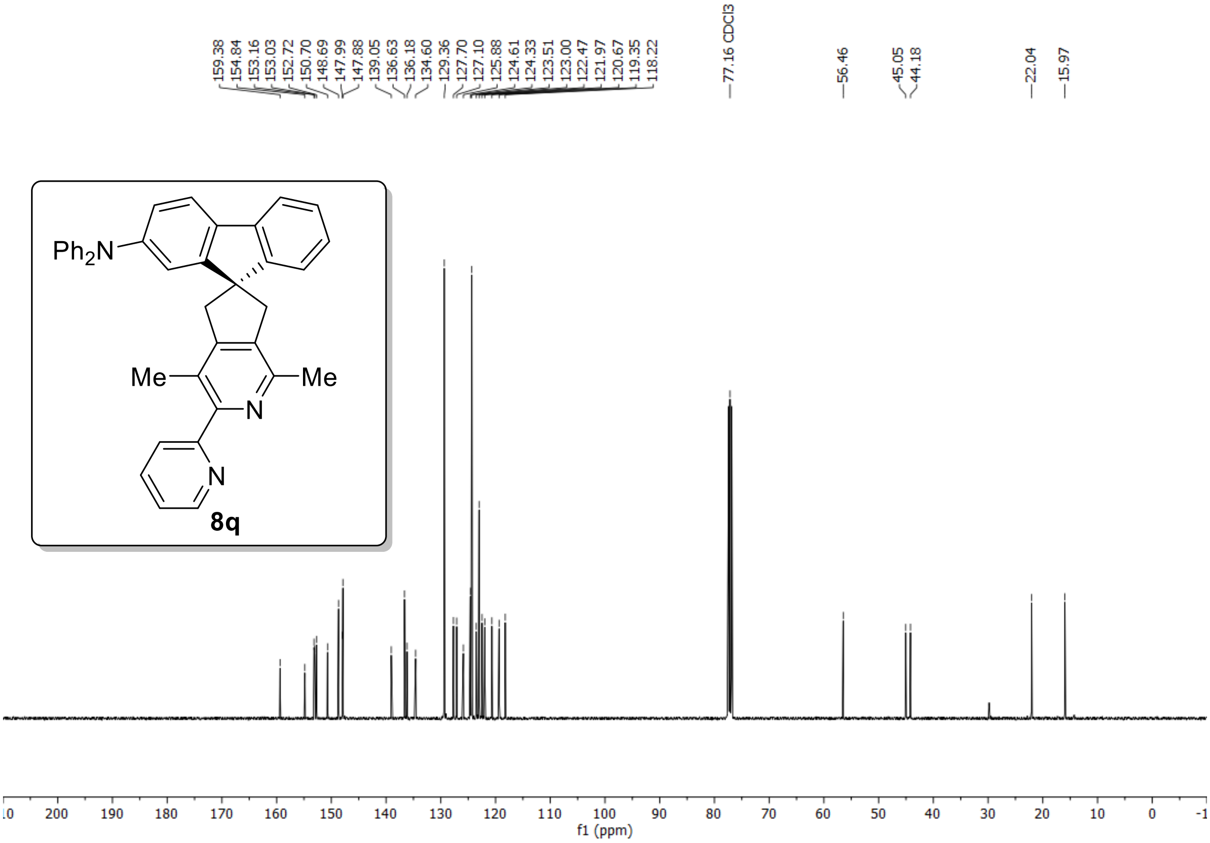

159.38
154.84
153.16
153.03
152.72
150.70
148.69
147.99
147.88
139.05
136.63
136.18
134.60
129.36
127.70
127.10
125.88
124.61
124.33
123.51
123.00
122.47
121.97
120.67
119.35
118.22
77.16 CDCl3
56.46
45.05
44.18
22.04
15.97
Ph2N
Me
Me
N
N
8q
f1 (ppm)


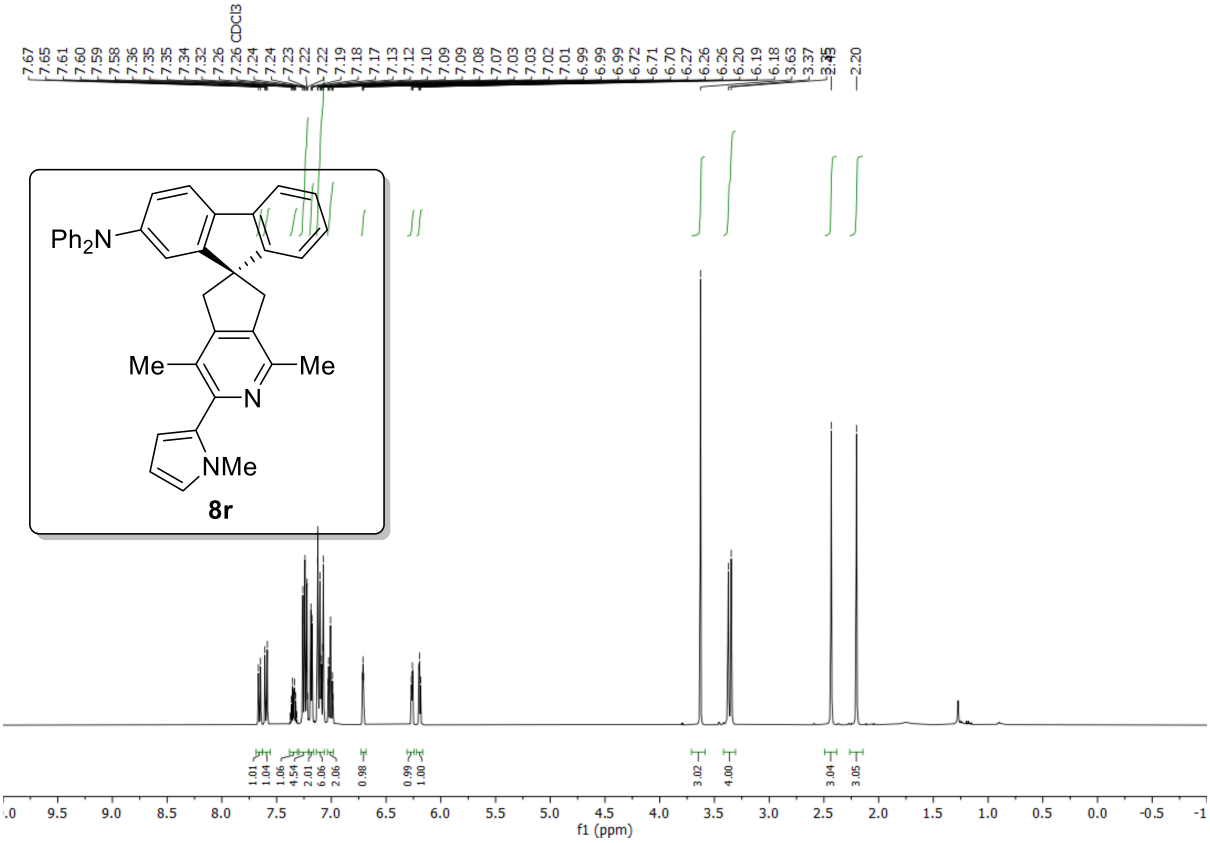

7.67
7.65
7.61
7.60
7.59
7.58
7.36
7.35
7.35
7.34
7.32
7.26
7.26 CDCl3
7.24
7.24
7.23
7.22
7.22
7.19
7.18
7.17
7.13
7.12
7.10
7.09
7.09
7.08
7.07
7.03
7.02
7.01
6.99
6.99
6.99
6.72
6.71
6.70
6.27
6.26
6.20
6.19
6.18
3.63
3.37
2.45
2.20
Ph2N
Me
Me
N
NMe
8r
1.01
1.04
1.06
4.54
2.01
6.06
2.06
0.98
0.99
1.00
3.02
4.00
3.04
3.05
f1 (ppm)

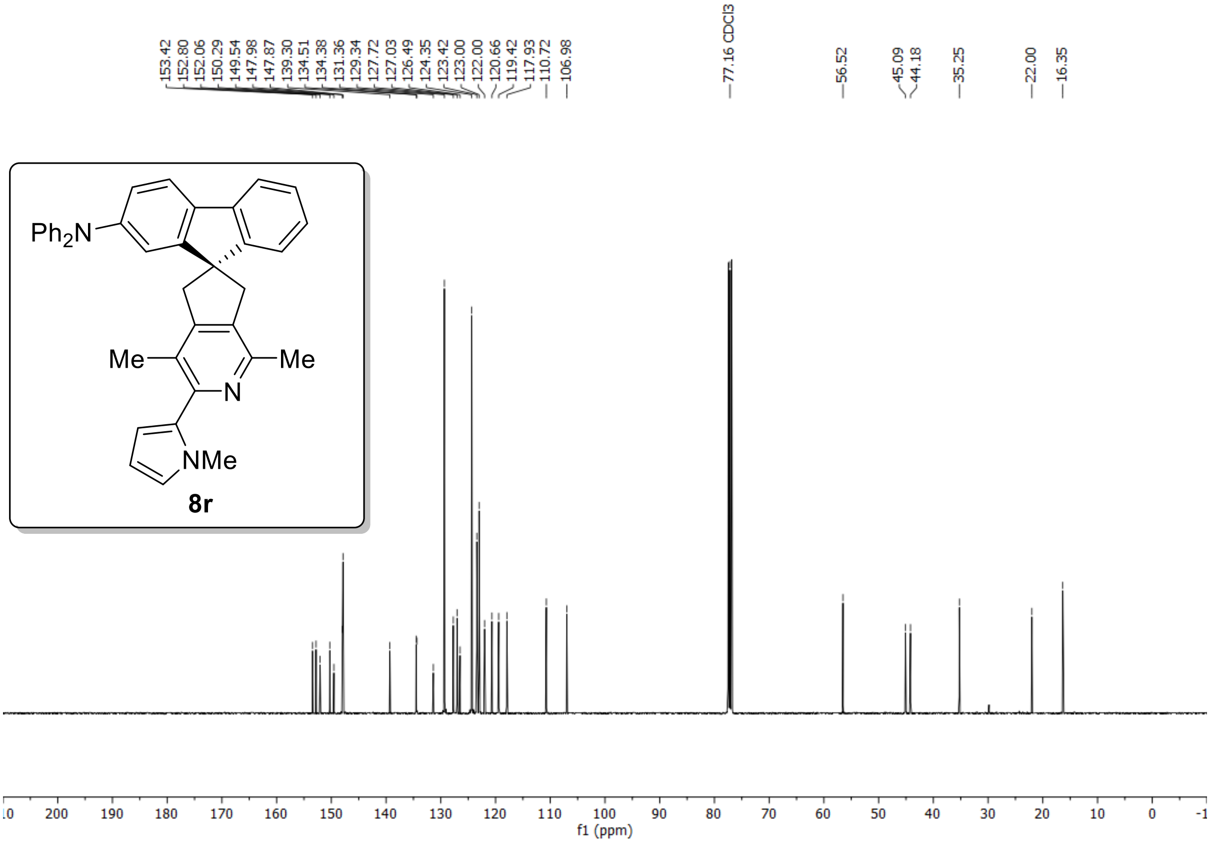
153.42
152.80
152.06
150.29
149.54
147.98
147.87
139.30
134.51
134.38
131.36
129.34
127.72
127.03
126.49
124.35
123.42
123.00
122.00
120.66
119.42
117.93
110.72
106.98
77.16 CDCl3
56.52
45.09
44.18
35.25
22.00
16.35
Ph2N
Me
Me
N
NMe
8r
f1 (ppm)

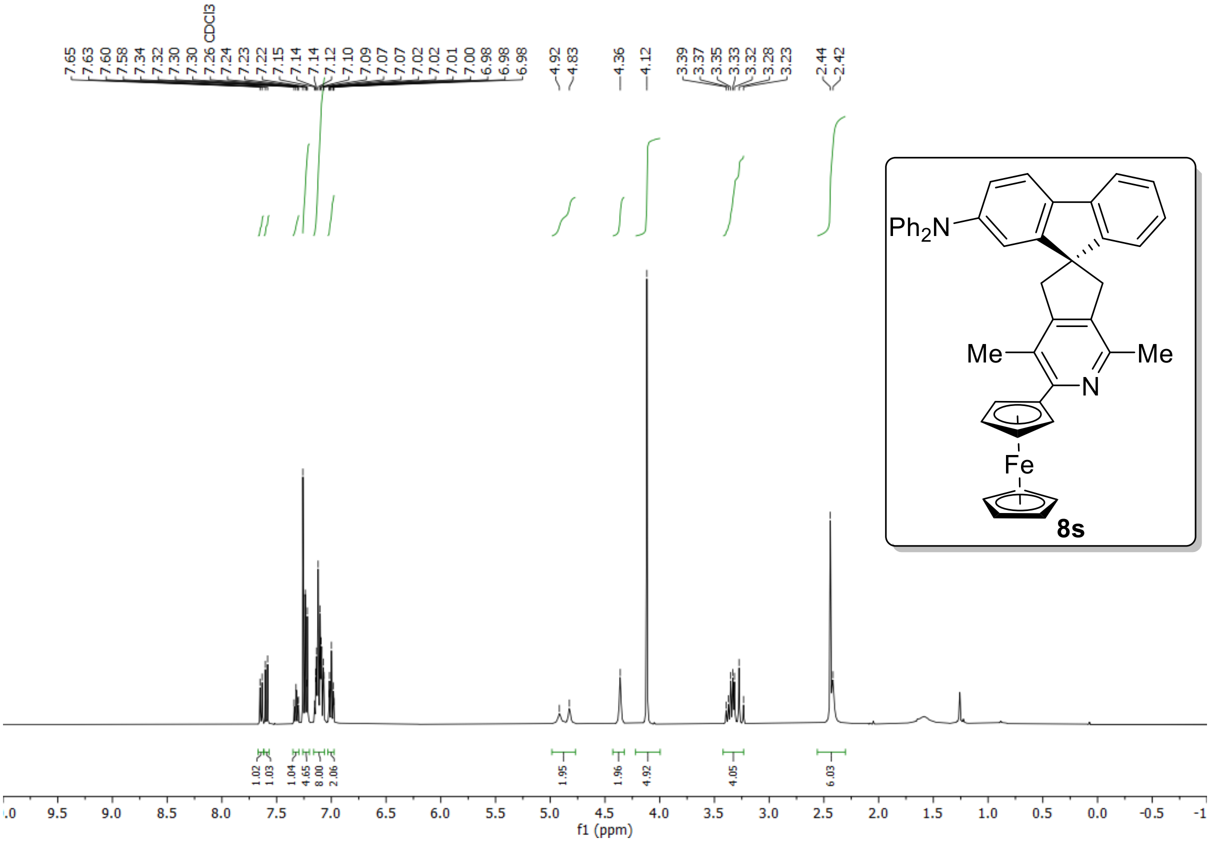
7.65
7.63
7.60
7.58
7.34
7.32
7.30
7.30
7.26 CDCl3
7.24
7.23
7.22
7.15
7.14
7.12
7.10
7.09
7.07
7.02
7.02
7.01
7.00
6.98
6.98
6.98
4.92
4.83
4.36
4.12
3.39
3.37
3.35
3.33
3.32
3.28
3.23
2.44
2.42
Ph2N
Me
Me
N
Fe
8s
1.02
1.03
1.04
4.65
8.00
2.06
1.95
1.96
4.92
4.05
6.03
f1 (ppm)

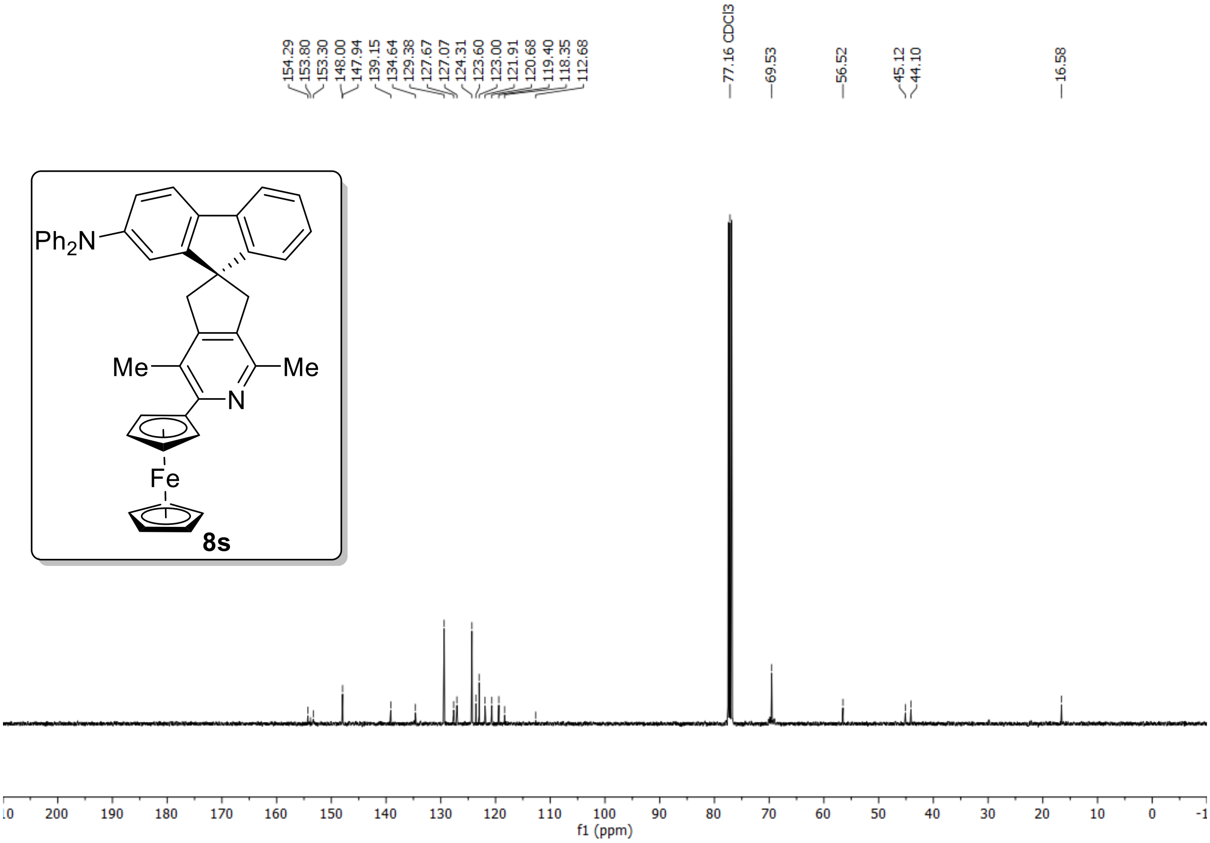
154.29
153.80
153.30
148.00
147.94
139.15
134.64
129.38
127.67
127.07
124.31
123.60
123.00
121.91
120.68
119.40
118.35
112.68
77.16 CDCl3
69.53
56.52
45.12
44.10
16.58
Ph2N
Me
Me
N
Fe
8s
f1 (ppm)

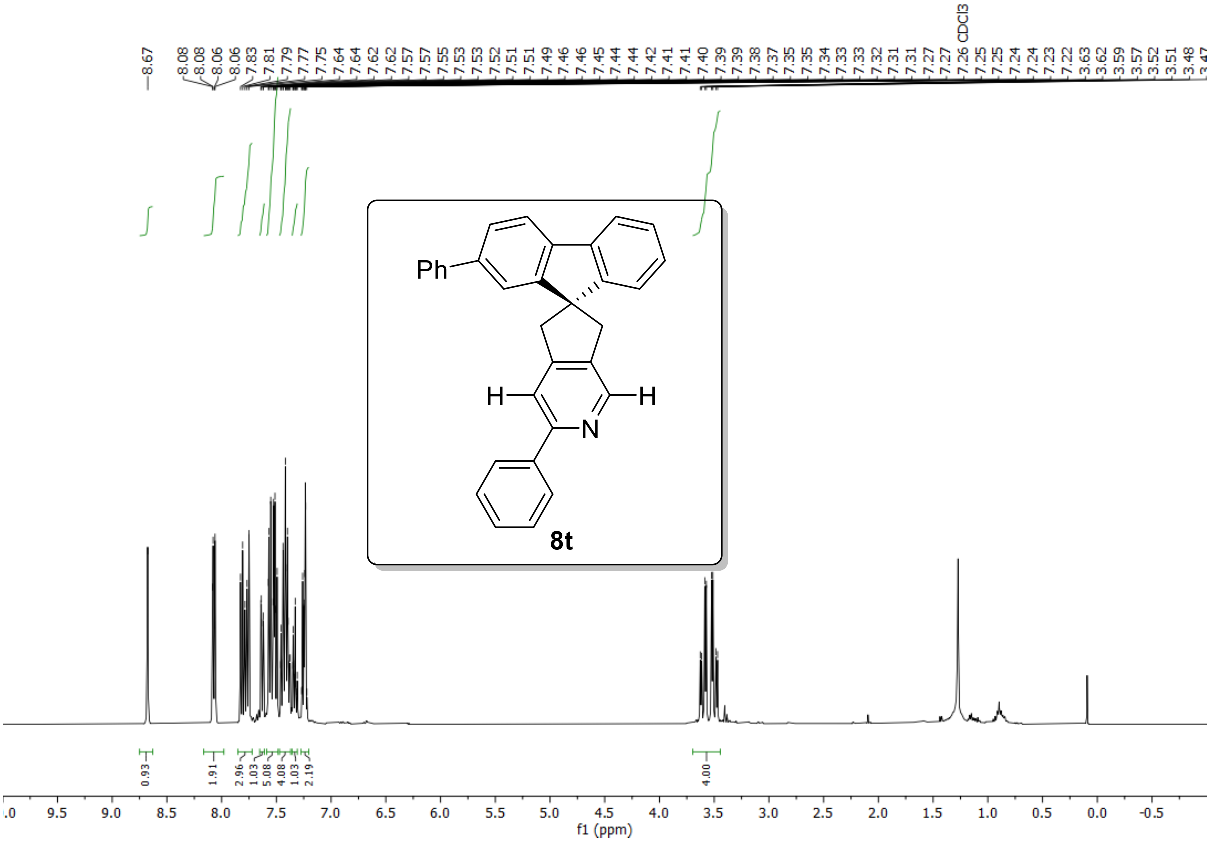
8.67
8.08
8.08
8.06
8.06
7.83
7.81
7.79
7.77
7.75
7.64
7.64
7.62
7.62
7.57
7.57
7.55
7.53
7.53
7.52
7.51
7.51
7.49
7.46
7.46
7.45
7.44
7.44
7.42
7.41
7.41
7.40
7.39
7.39
7.38
7.37
7.35
7.35
7.34
7.33
7.33
7.32
7.31
7.31
7.27
7.26 CDCl3
7.25
7.25
7.24
7.24
7.23
7.22
3.63
3.62
3.59
3.57
3.52
3.51
3.48
3.47
Ph
H
H
N
8t
0.93
1.91
2.96
1.03
5.08
4.08
1.03
2.19
4.00
f1 (ppm)

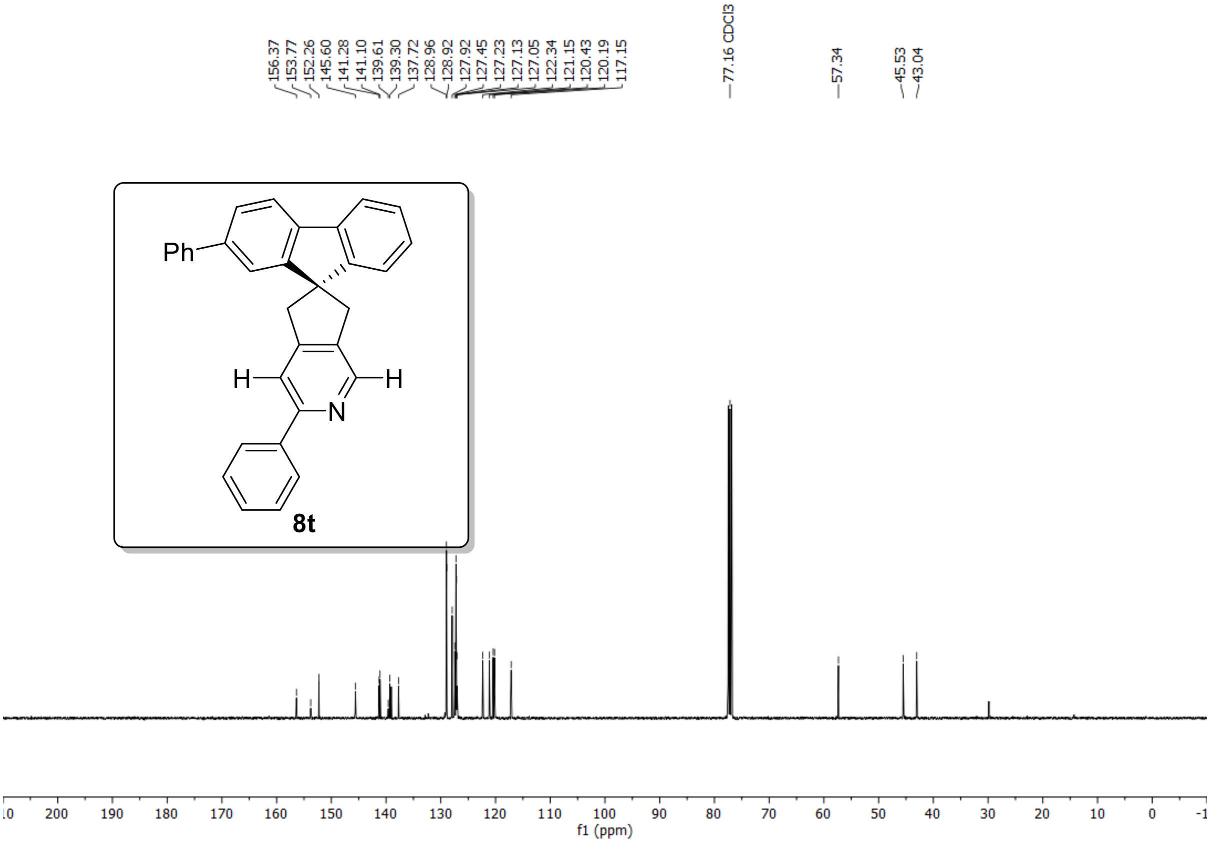
156.37
153.77
152.26
145.60
141.28
141.10
139.61
139.30
137.72
128.96
128.92
127.92
127.45
127.23
127.13
127.05
122.34
121.15
120.43
120.19
117.15
77.16 CDCl3
57.34
45.53
43.04
Ph
H
H
N
8t
f1 (ppm)

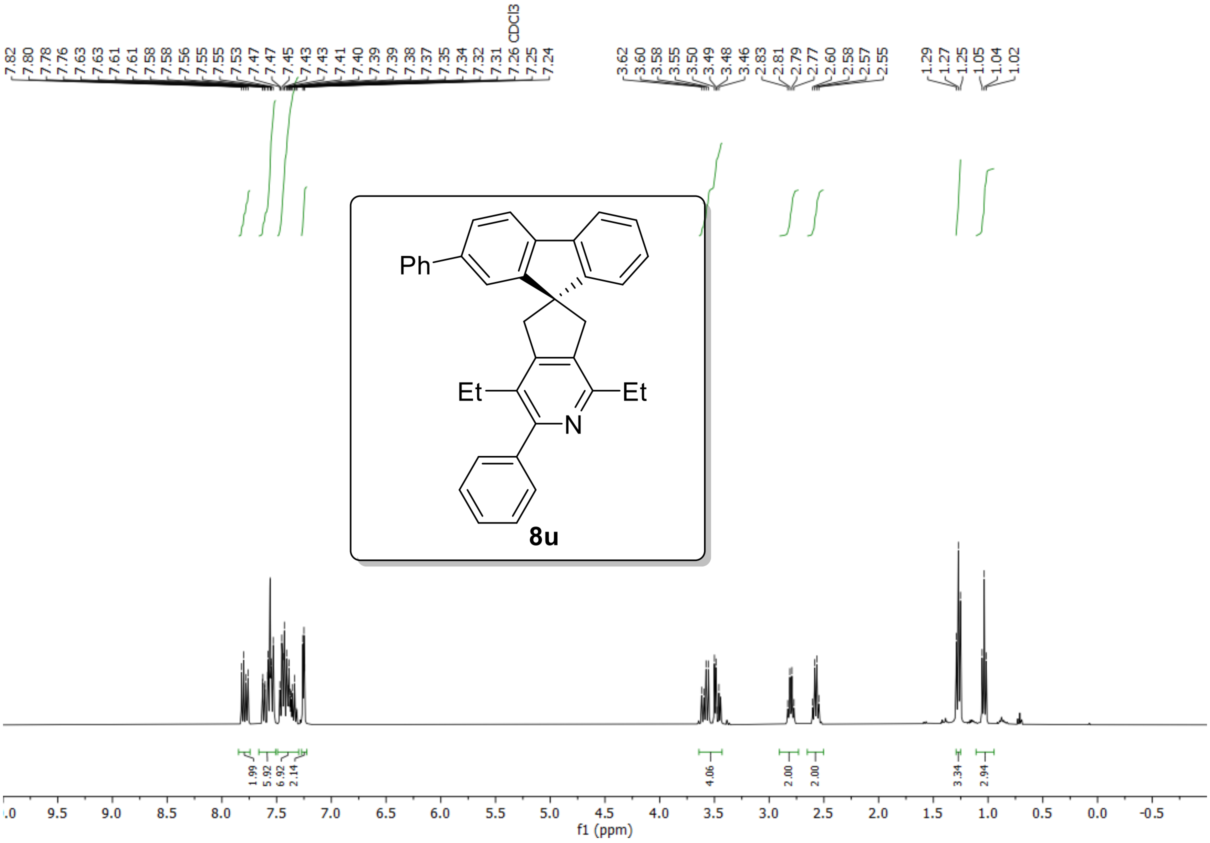
7.82
7.80
7.78
7.76
7.63
7.63
7.61
7.61
7.58
7.58
7.56
7.55
7.55
7.53
7.47
7.47
7.45
7.43
7.43
7.41
7.40
7.39
7.39
7.38
7.37
7.35
7.34
7.32
7.31
7.26 CDCl3
7.25
7.24
3.62
3.60
3.58
3.55
3.50
3.49
3.48
3.46
2.83
2.81
2.79
2.77
2.60
2.58
2.57
2.55
1.29
1.27
1.25
1.05
1.04
1.02
Ph
Et
Et
N
8u
1.99
5.92
6.92
2.14
4.06
2.00
2.00
3.34
2.94
f1 (ppm)

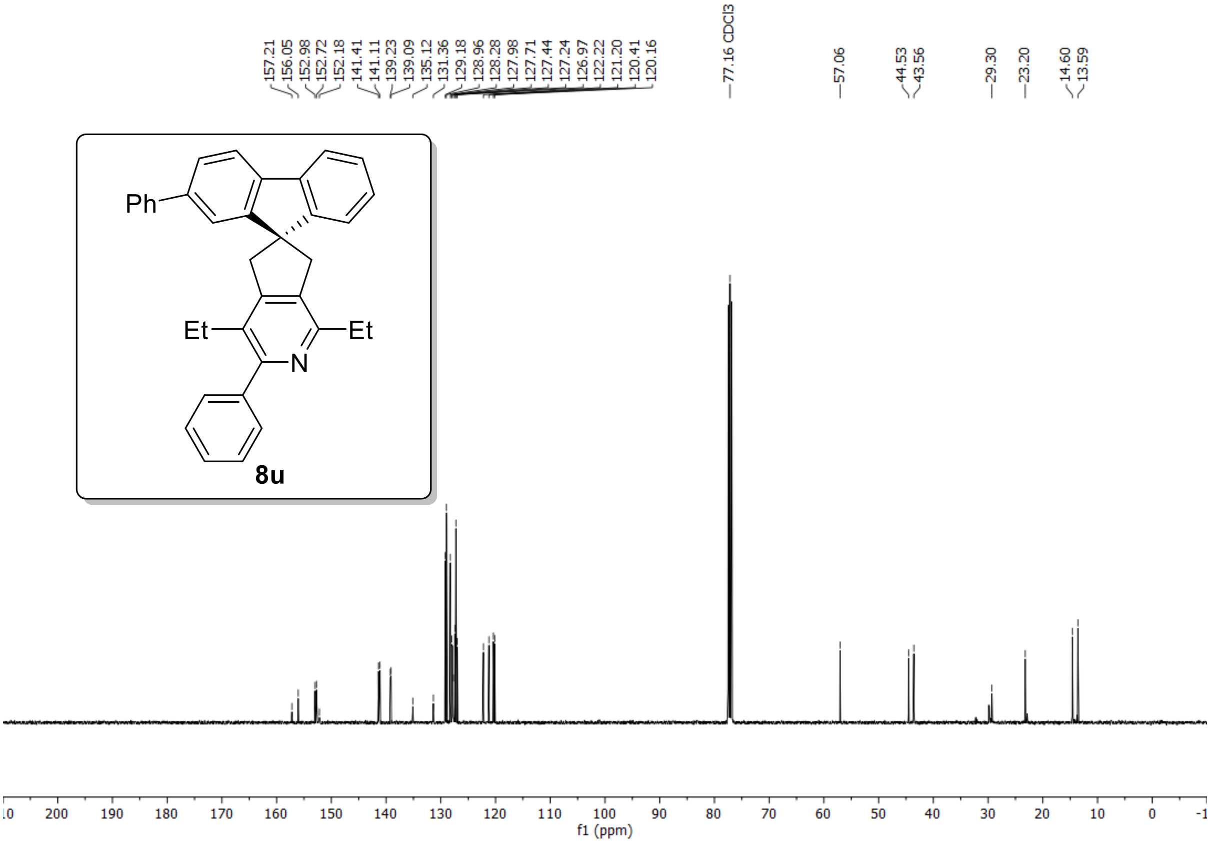
157.21 156.05 152.98 152.72 152.18 141.41 141.11 139.23 139.09 135.12 131.36 129.18 128.96 128.28 127.98 127.71 127.44 127.24 126.97 122.22 121.20 120.41 120.16
77.16 CDCl3
57.06
44.53 43.56
29.30
23.20
14.60 13.59
Ph
Et
Et
N
8u
f1 (ppm)

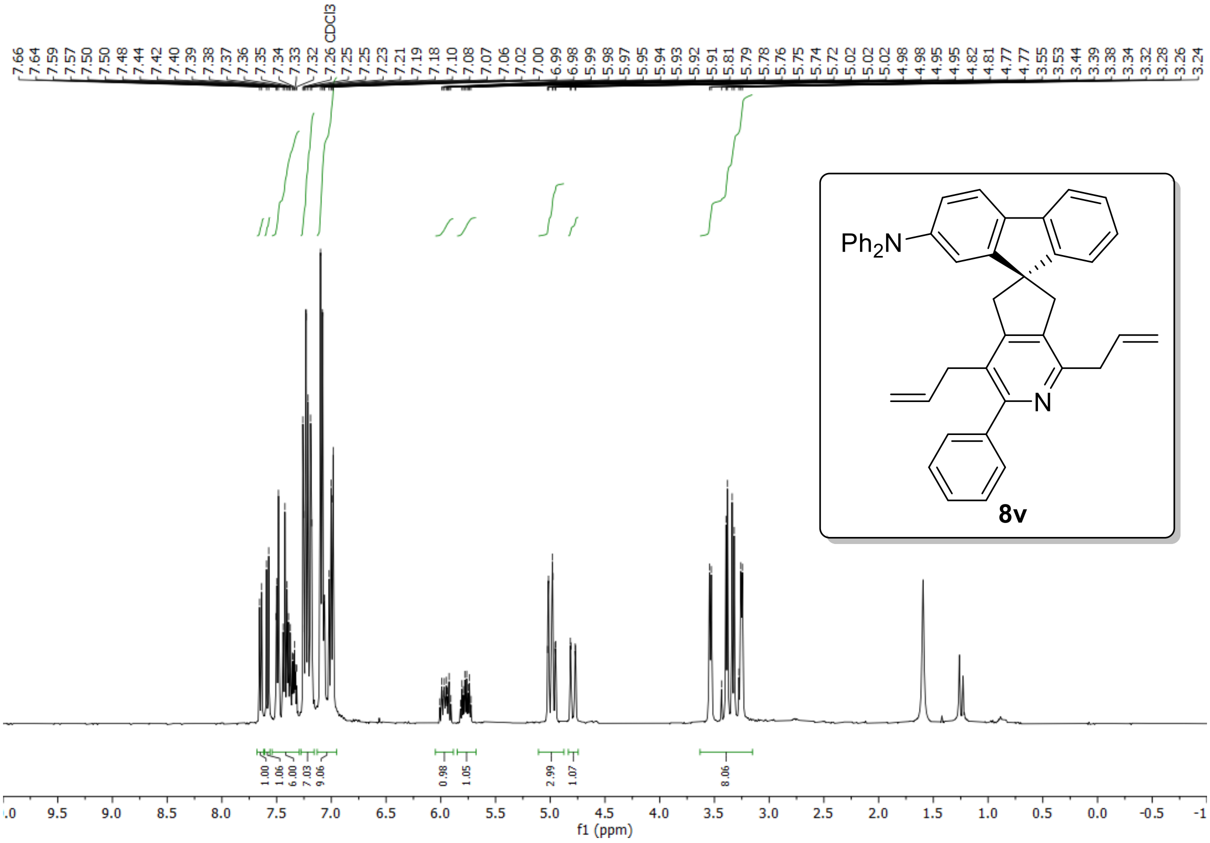
7.66 7.64 7.59 7.57 7.50 7.50 7.48 7.44 7.42 7.40 7.39 7.38 7.37 7.36 7.35 7.34 7.33 7.32 7.26 CDCl3 7.25 7.25 7.23 7.21 7.19 7.18 7.10 7.08 7.07 7.06 7.02 7.00 6.99 6.98 5.99 5.98 5.97 5.95 5.94 5.93 5.92 5.91 5.81 5.79 5.78 5.76 5.75 5.74 5.72 5.02 5.02 5.02 4.98 4.98 4.95 4.95 4.82 4.81 4.77 4.77 3.55 3.53 3.44 3.39 3.38 3.34 3.32 3.28 3.26 3.24
$Ph_2N$
N
8v
1.00 1.06 6.00 7.03 9.06 0.98 1.05 2.99 1.07 8.06
f1 (ppm)

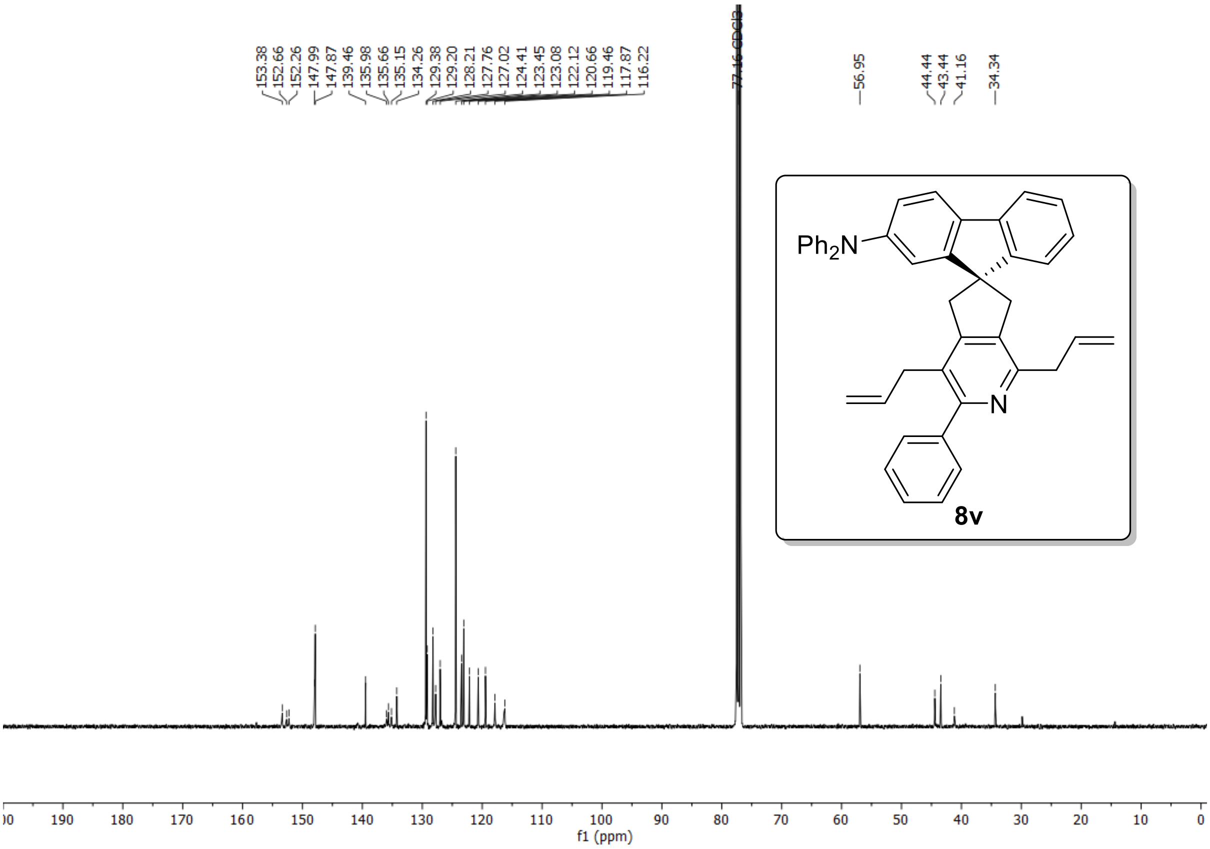
153.38
152.66
152.26
147.99
147.87
139.46
135.98
135.66
135.15
134.26
129.38
129.20
128.21
127.76
127.02
124.41
123.45
123.08
122.12
120.66
119.46
117.87
116.22
77.16 CDCl3
56.95
44.44
43.44
41.16
34.34
Ph2N
N
8v
f1 (ppm)

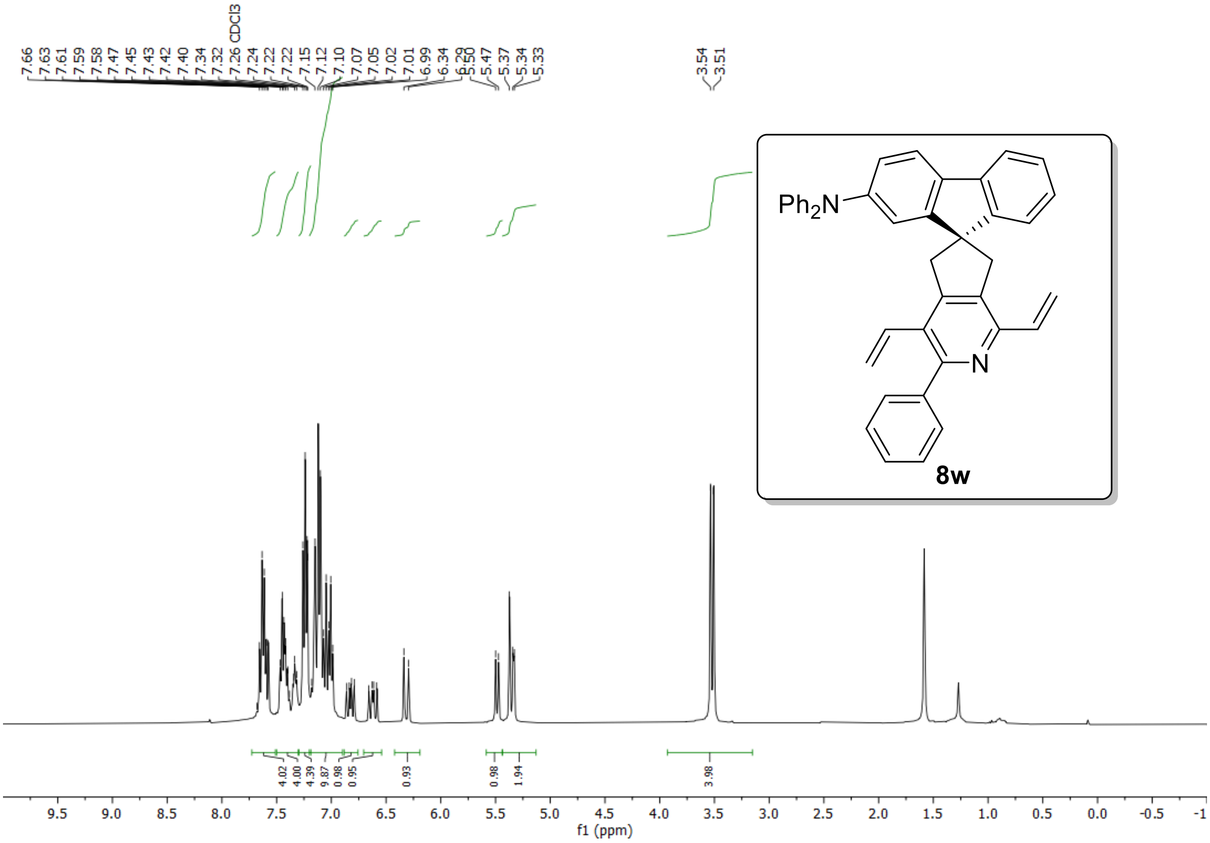
7.66
7.63
7.61
7.59
7.58
7.47
7.45
7.43
7.42
7.40
7.34
7.32
7.26 CDCl3
7.24
7.22
7.22
7.15
7.12
7.10
7.07
7.05
7.02
7.01
6.99
6.34
6.29
5.50
5.47
5.37
5.34
5.33
3.54
3.51
Ph2N
N
8w
4.02
4.00
4.39
9.87
0.98
0.95
0.93
0.98
1.94
3.98
f1 (ppm)

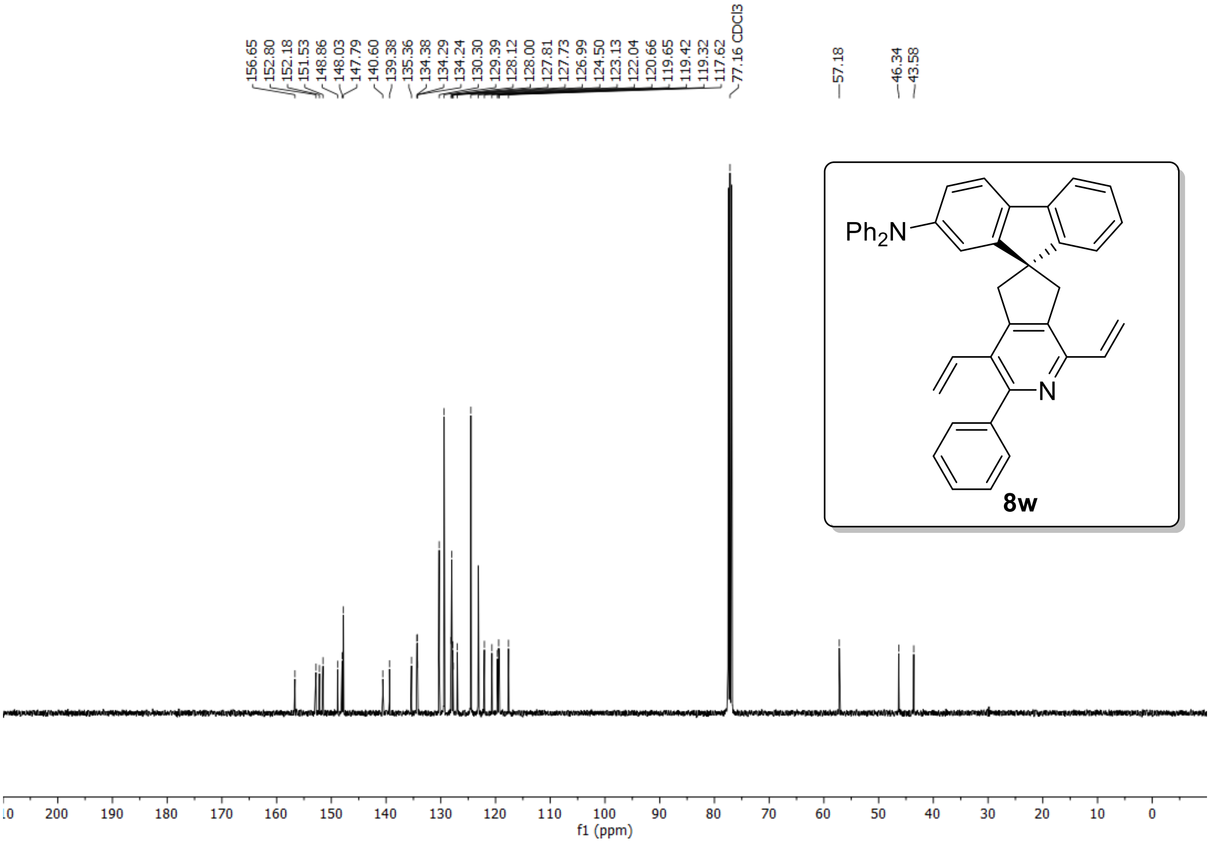
Ph2N
8w
f1 (ppm)

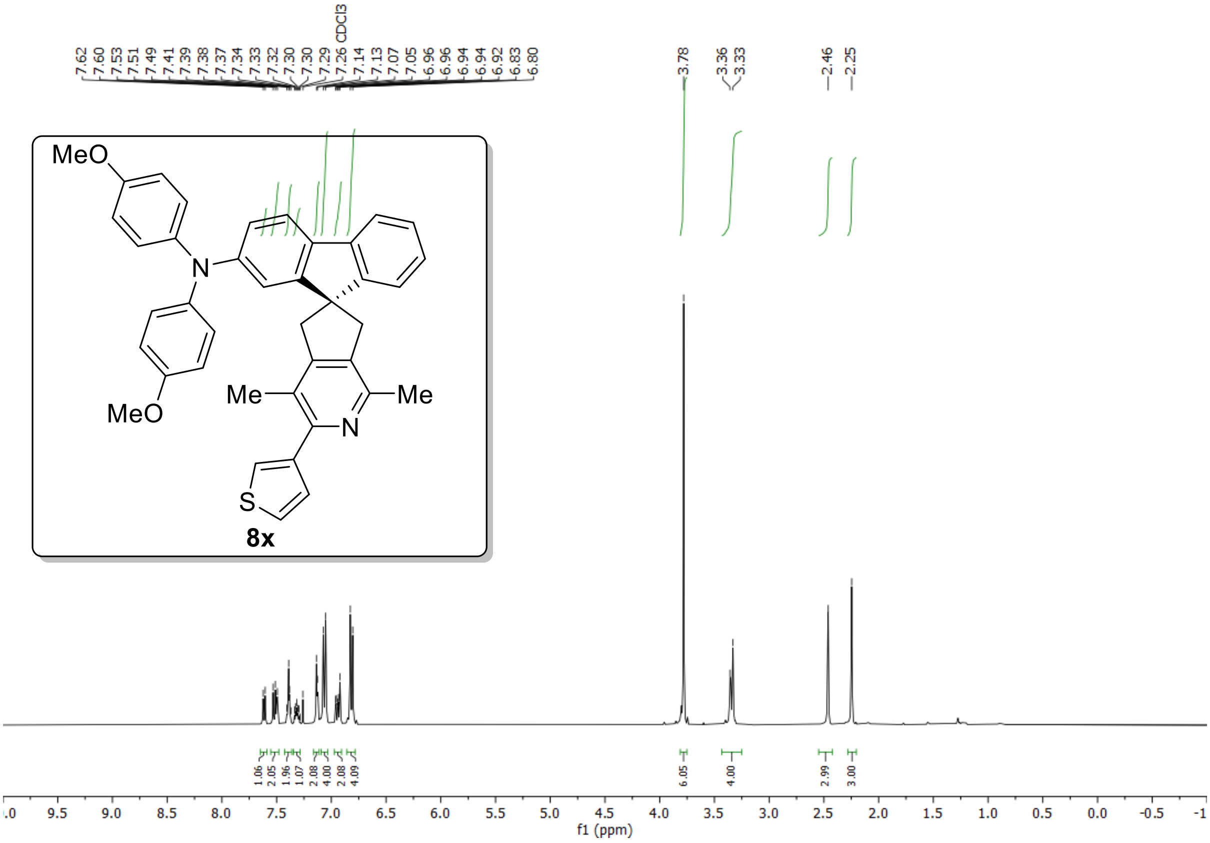
MeO
N
MeO
Me
Me
N
S
8x
f1 (ppm)

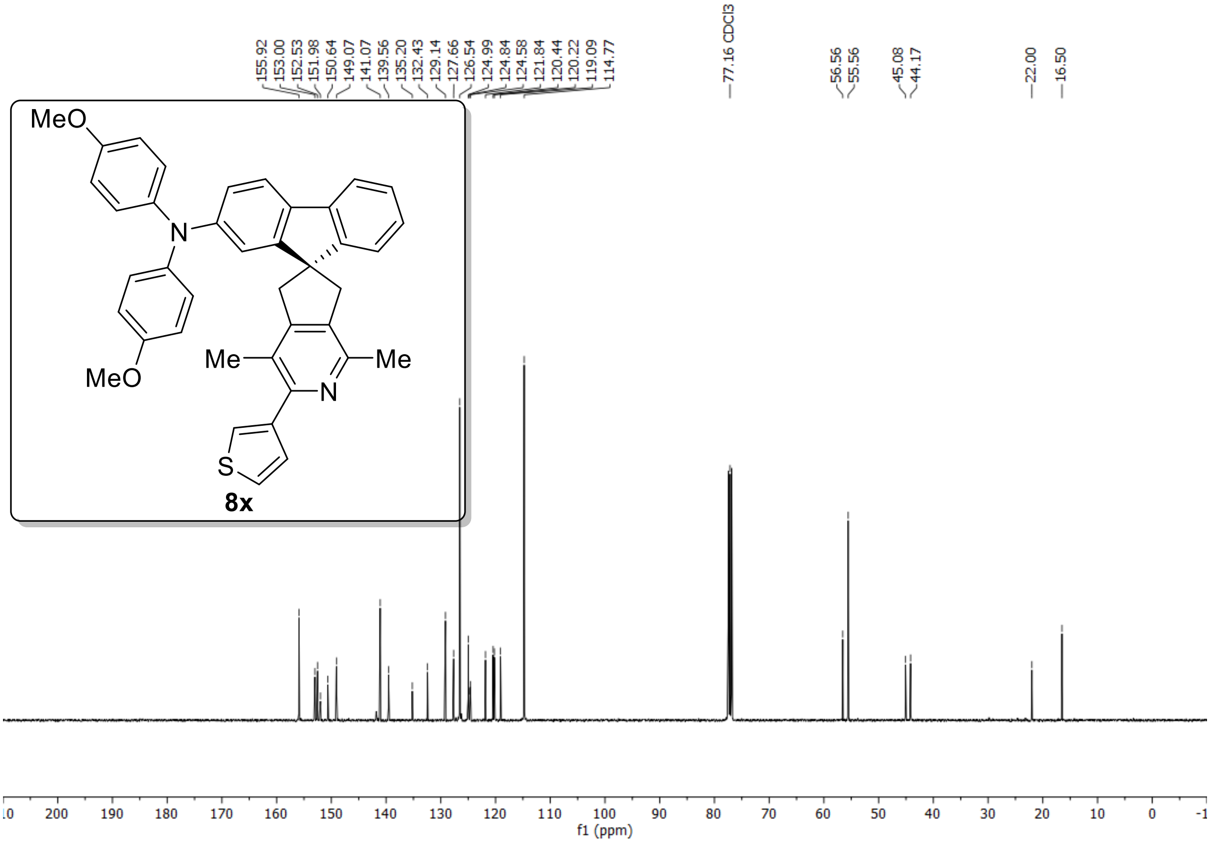

155.92
153.00
152.53
151.98
150.64
149.07
141.07
139.56
135.20
132.43
129.14
127.66
126.54
124.99
124.84
124.58
121.84
120.44
120.22
119.09
114.77
77.16 CDCl3
56.56
55.56
45.08
44.17
22.00
16.50
MeO
N
MeO
Me
Me
N
S
8x
f1 (ppm)


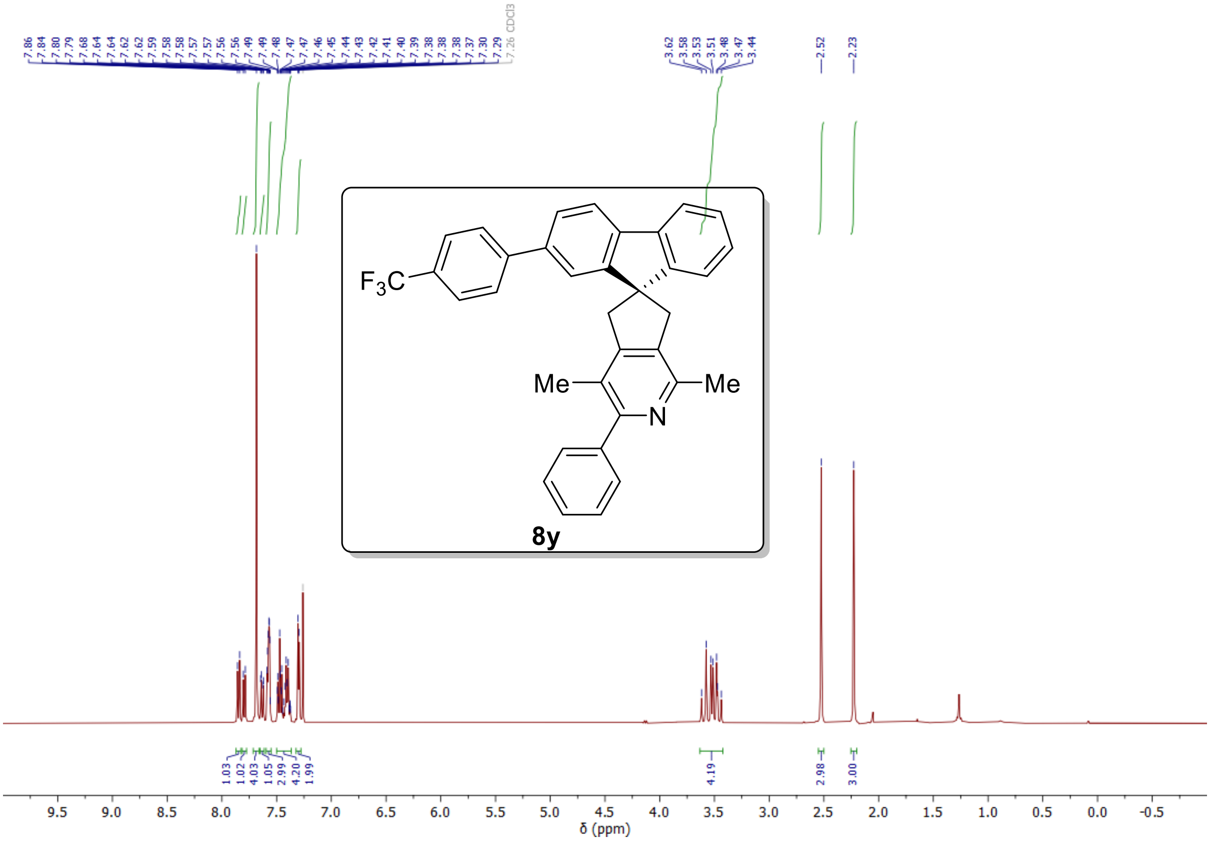

3.62
3.58
3.53
3.51
3.48
3.47
3.44
2.52
2.23
$F_3C$
Me
Me
N
8y
δ (ppm)

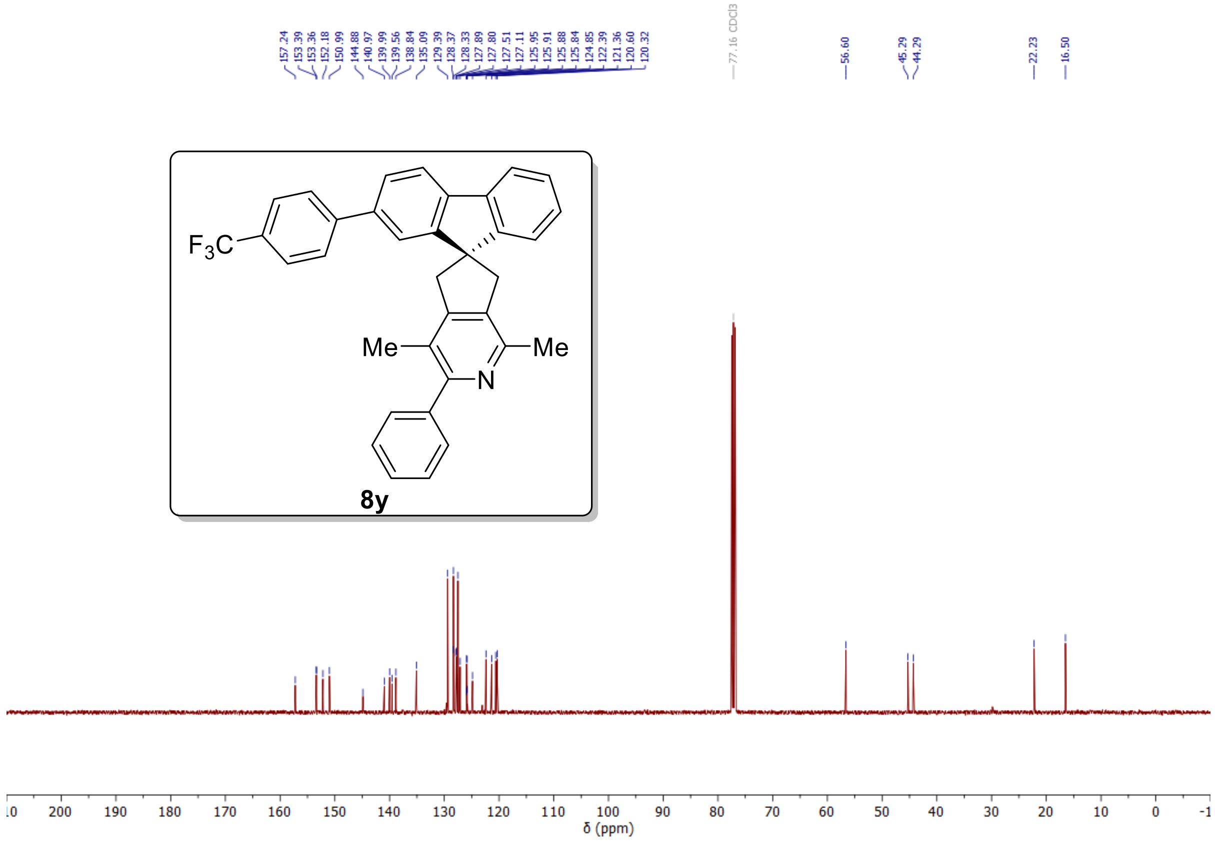
157.24
153.39
153.36
152.18
150.99
144.88
140.97
139.99
139.56
138.84
135.09
129.39
128.37
128.33
127.89
127.80
127.51
127.11
125.95
125.91
125.88
125.84
124.85
122.39
121.36
120.60
120.32
77.16 CDCl3
56.60
45.29
44.29
22.23
16.50
$F_3C$
Me
Me
N
8y
δ (ppm)

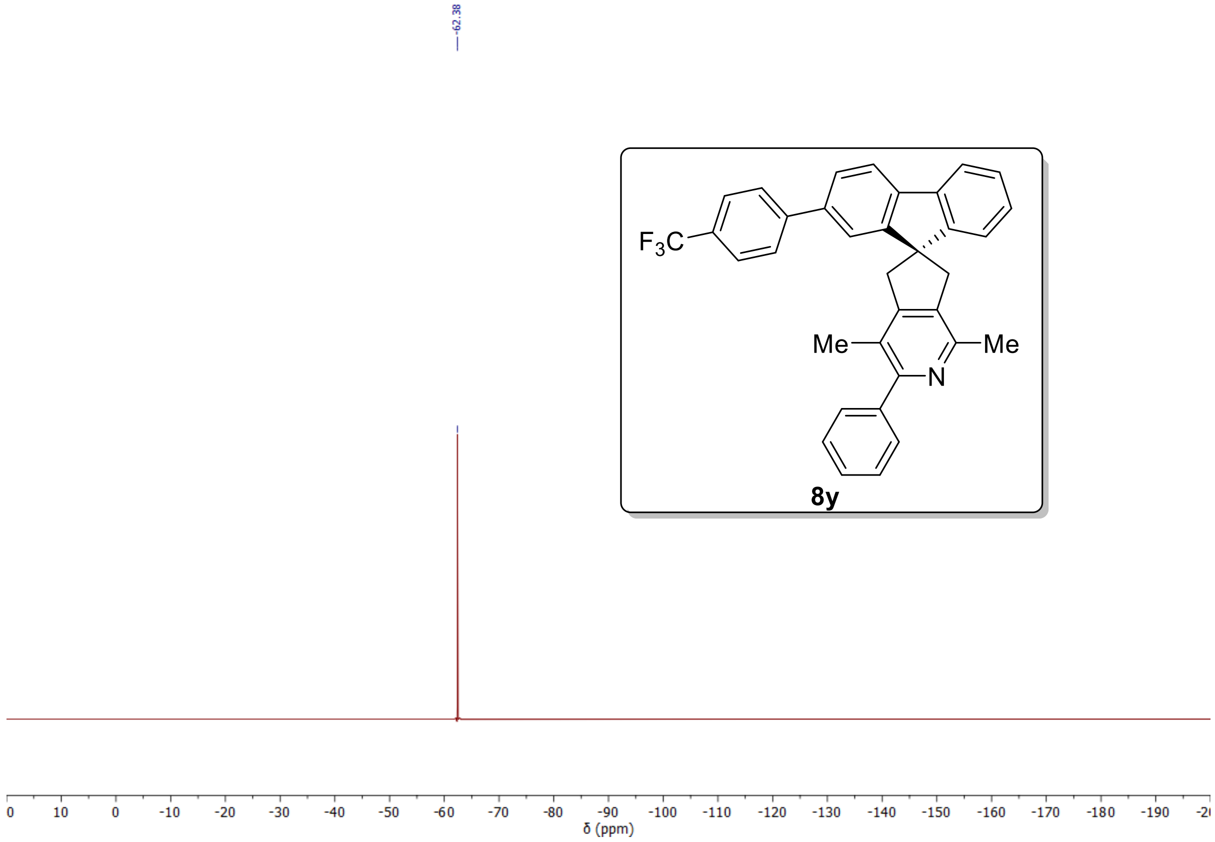
-62.38
$F_3C$
Me
Me
N
8y
δ (ppm)

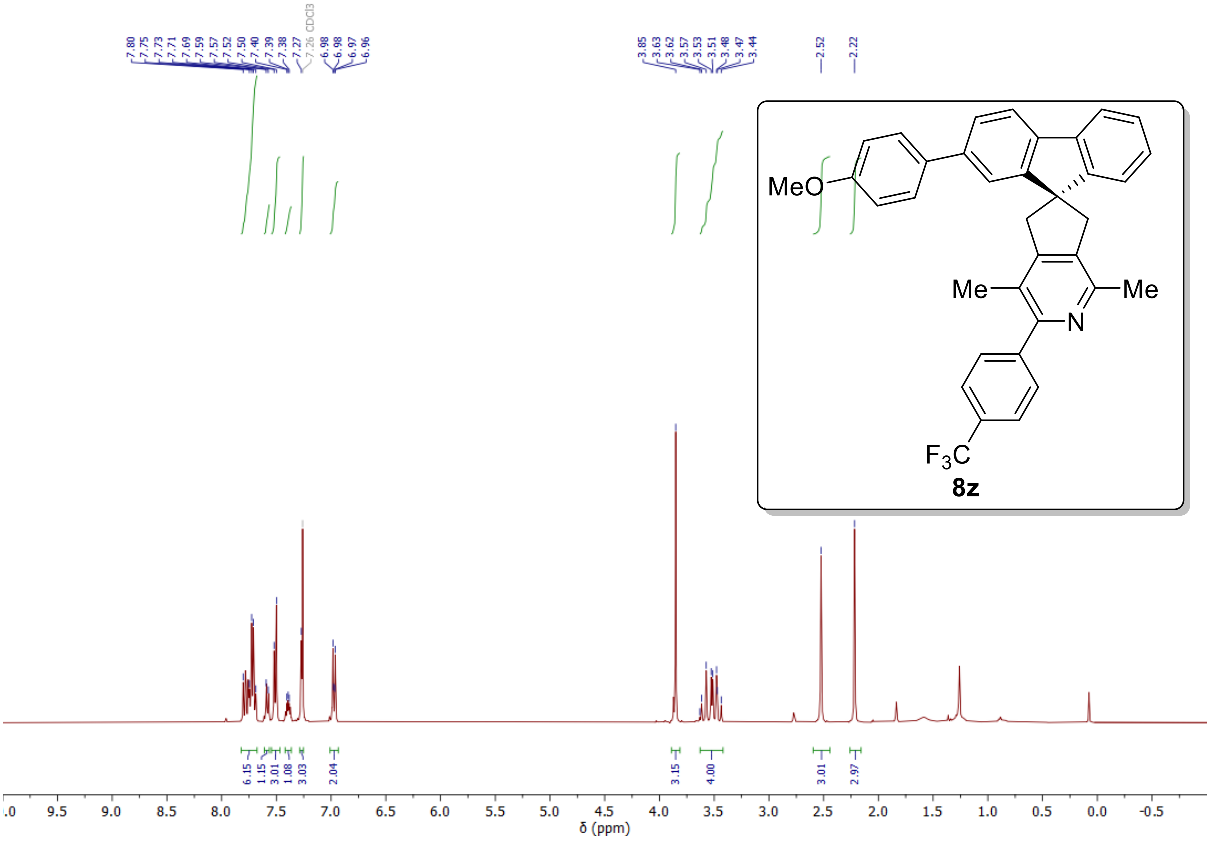
MeO
Me
Me
N
F3C
8z
δ (ppm)

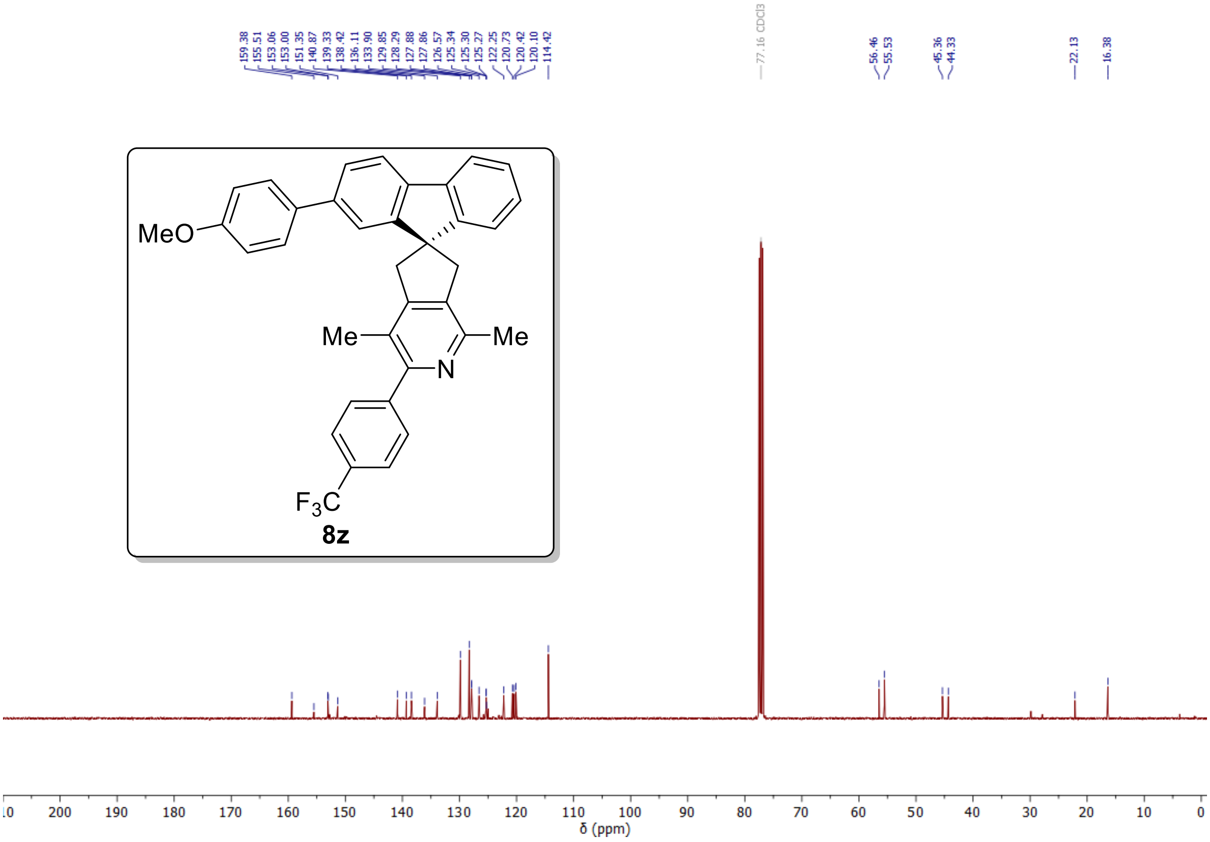
MeO
Me
Me
N
F3C
8z
δ (ppm)

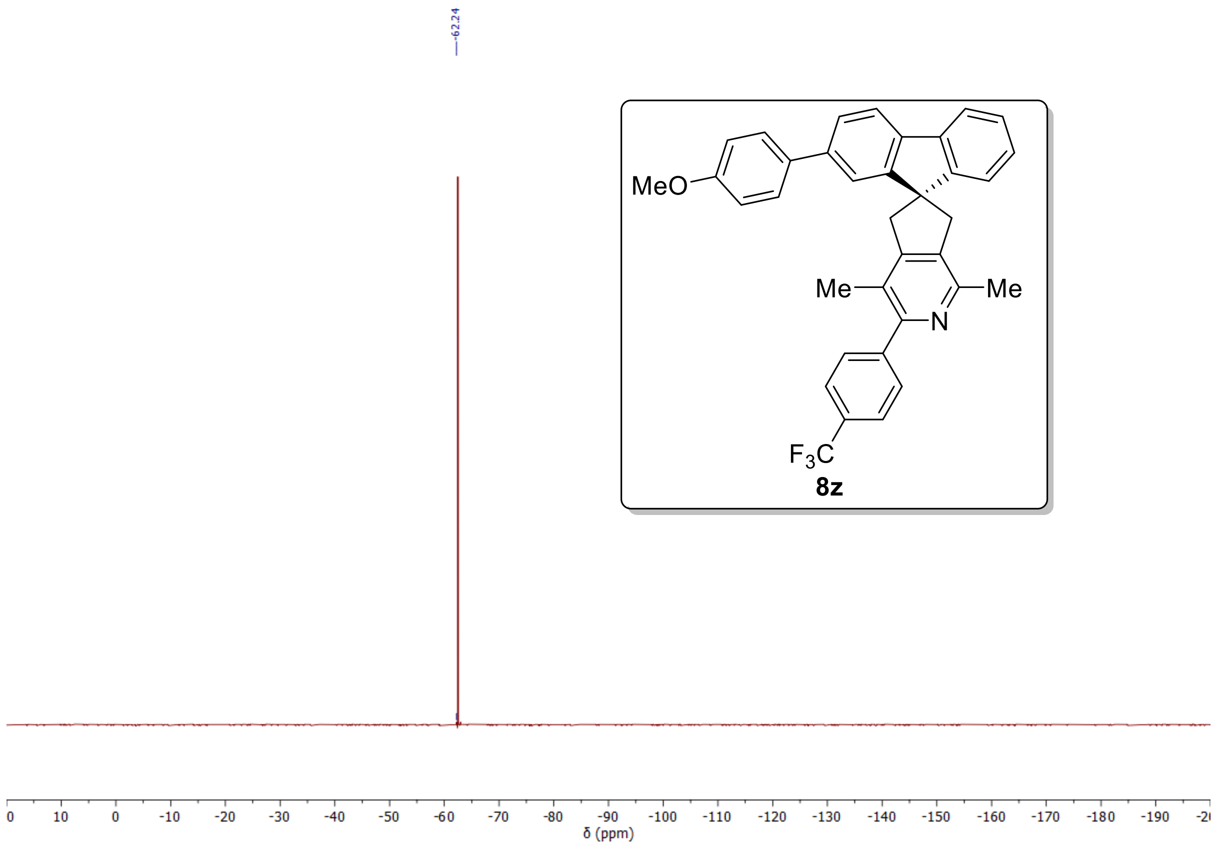
-62.24
MeO
Me
Me
N
F3C
8z
δ (ppm)

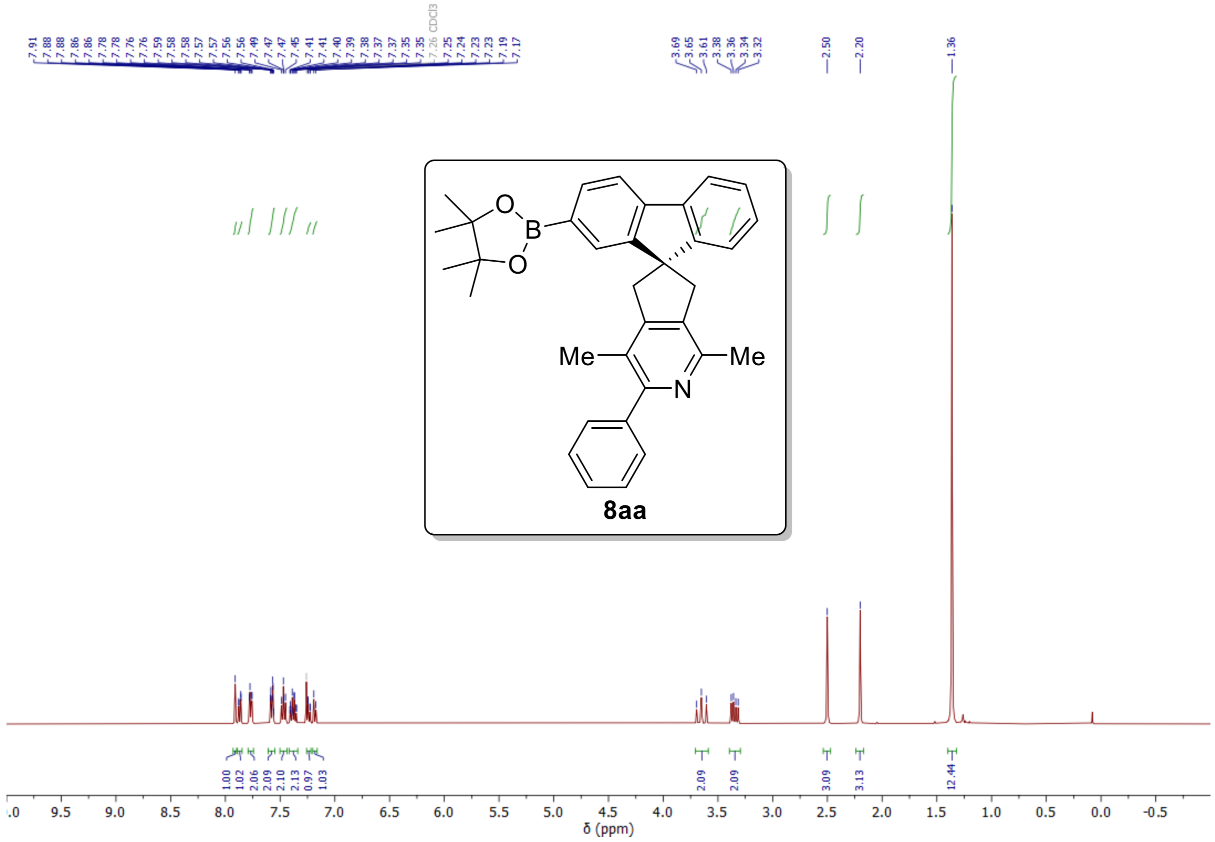
O
B
O
Me
Me
N
8aa
δ (ppm)

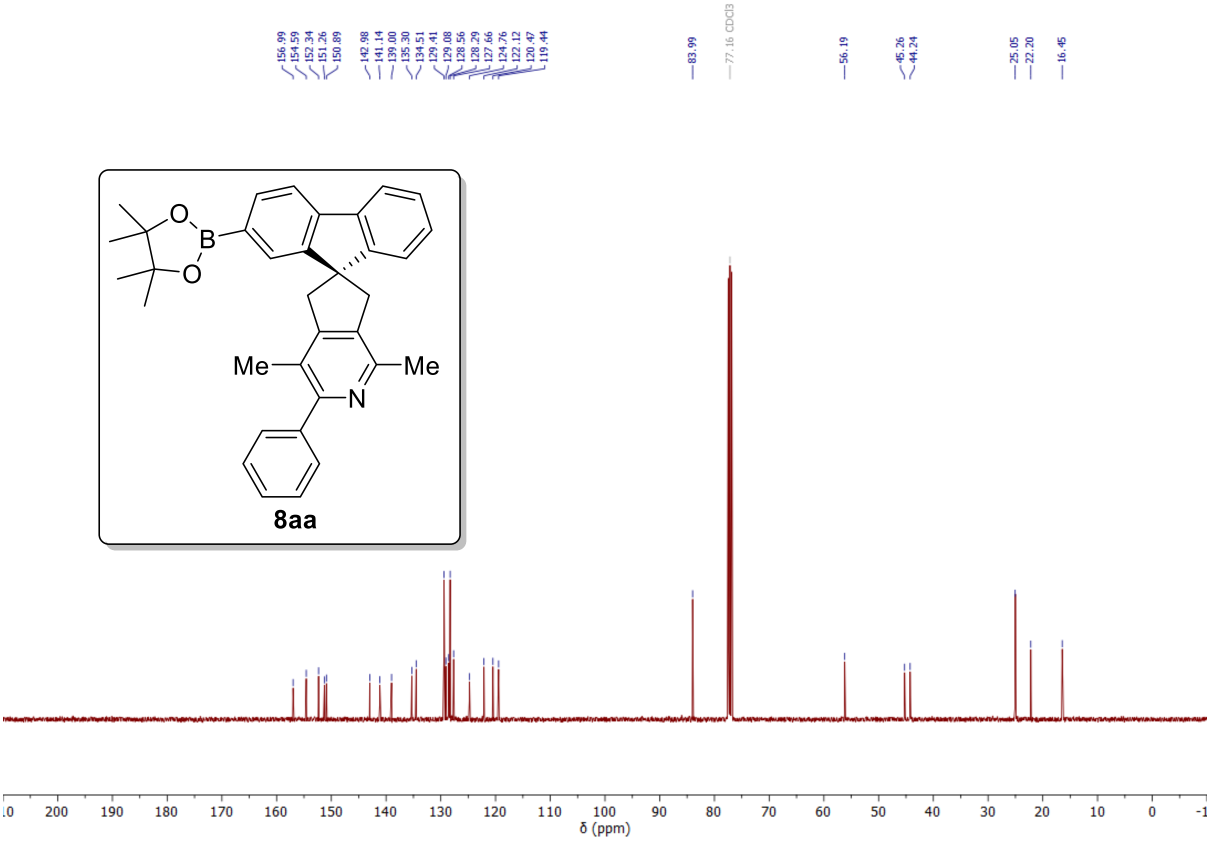
156.99
154.59
152.34
151.26
150.89
142.98
141.14
139.00
135.30
134.51
129.41
129.08
128.56
128.29
127.66
124.76
122.12
120.47
119.44
83.99
77.16 CDCl3
56.19
45.26
44.24
25.05
22.20
16.45
O
B
O
Me
Me
N
8aa
δ (ppm)

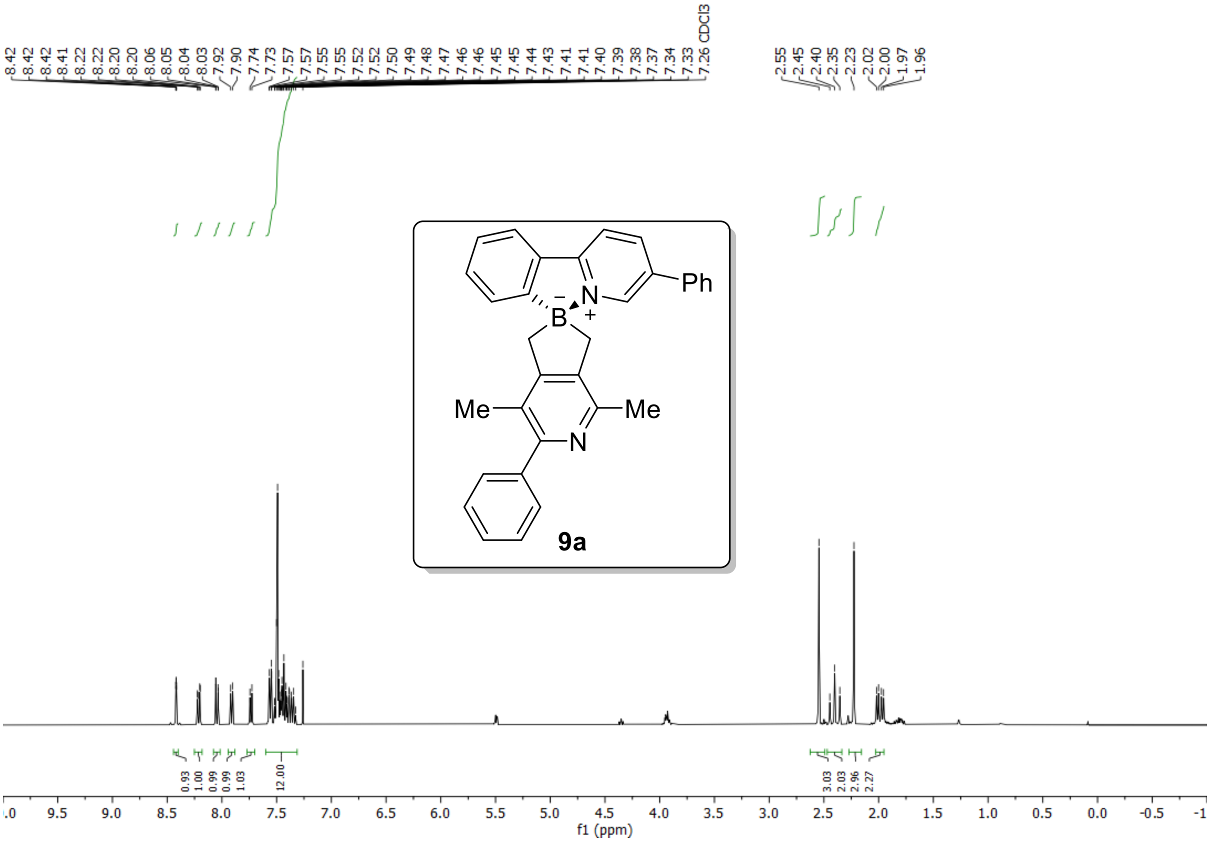
CDCl3
Ph
N
B
Me
Me
N
9a
f1 (ppm)

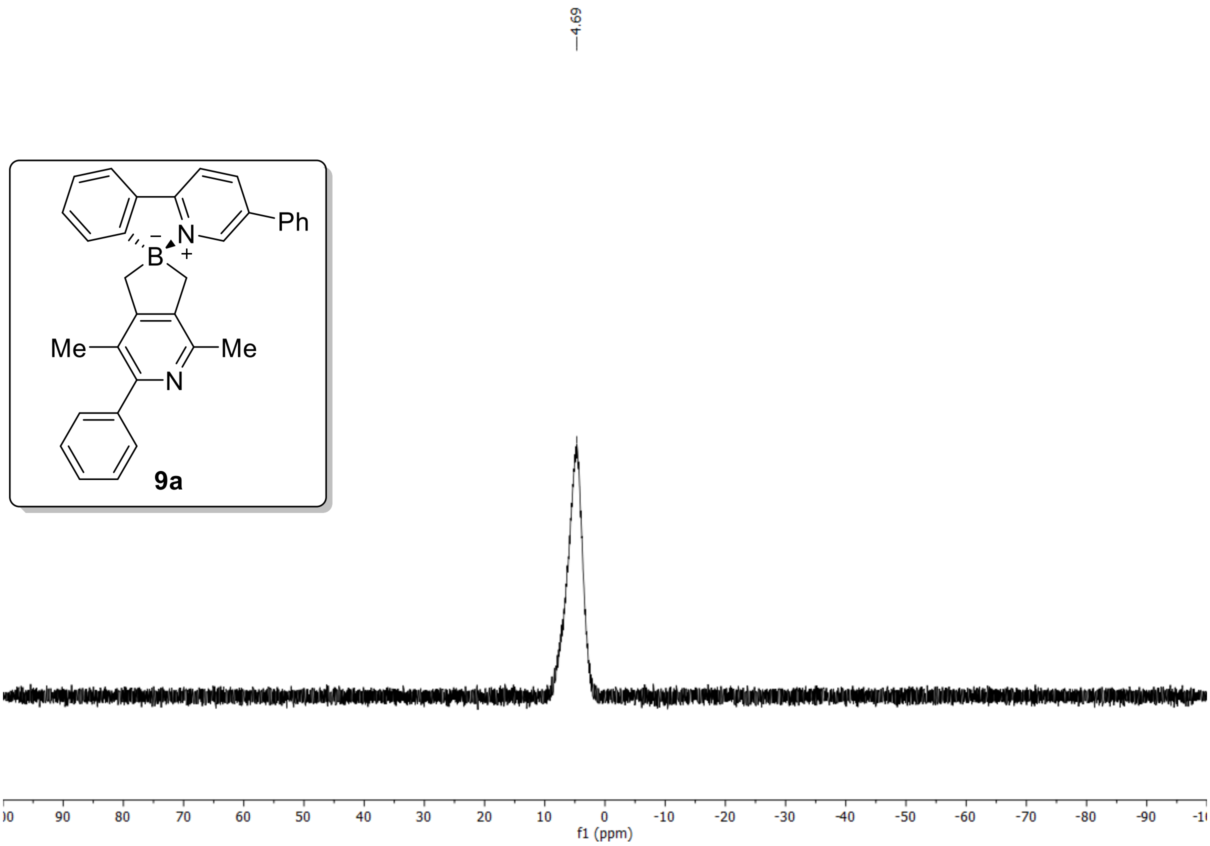
4.69
Ph
B
N
Me
Me
N
9a
f1 (ppm)

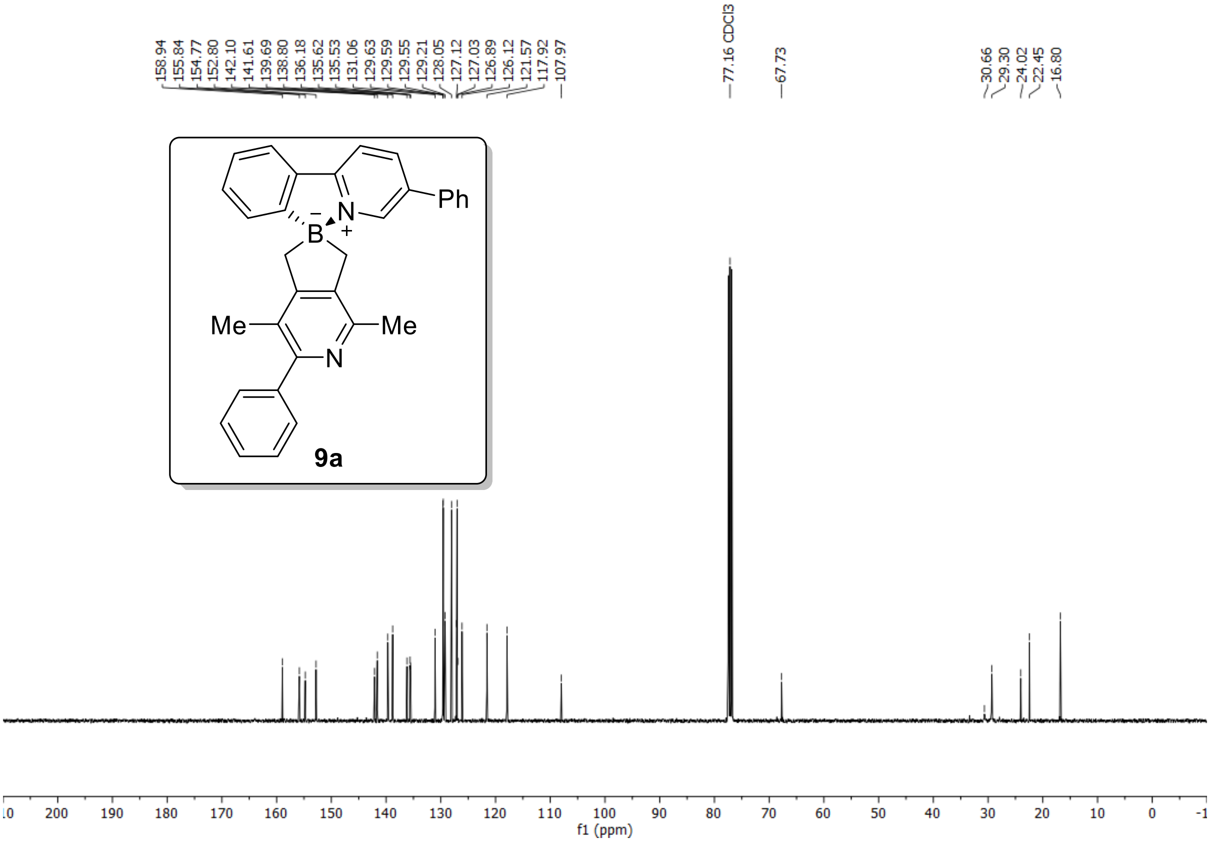
158.94
155.84
154.77
152.80
142.10
141.61
139.69
138.80
136.18
135.62
135.53
131.06
129.63
129.59
129.55
129.21
128.05
127.12
127.03
126.89
126.12
121.57
117.92
107.97
77.16 CDCl3
67.73
30.66
29.30
24.02
22.45
16.80
Ph
B
N
Me
Me
N
9a
f1 (ppm)

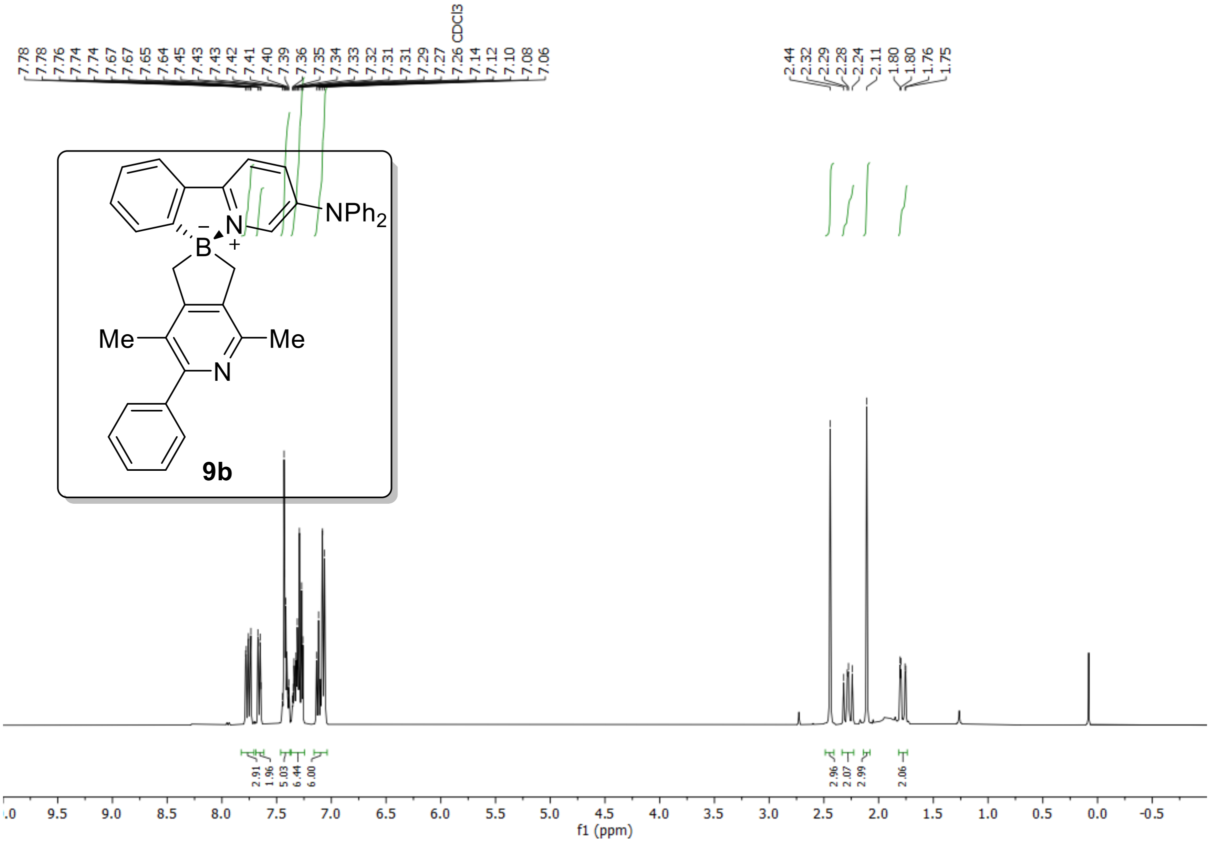

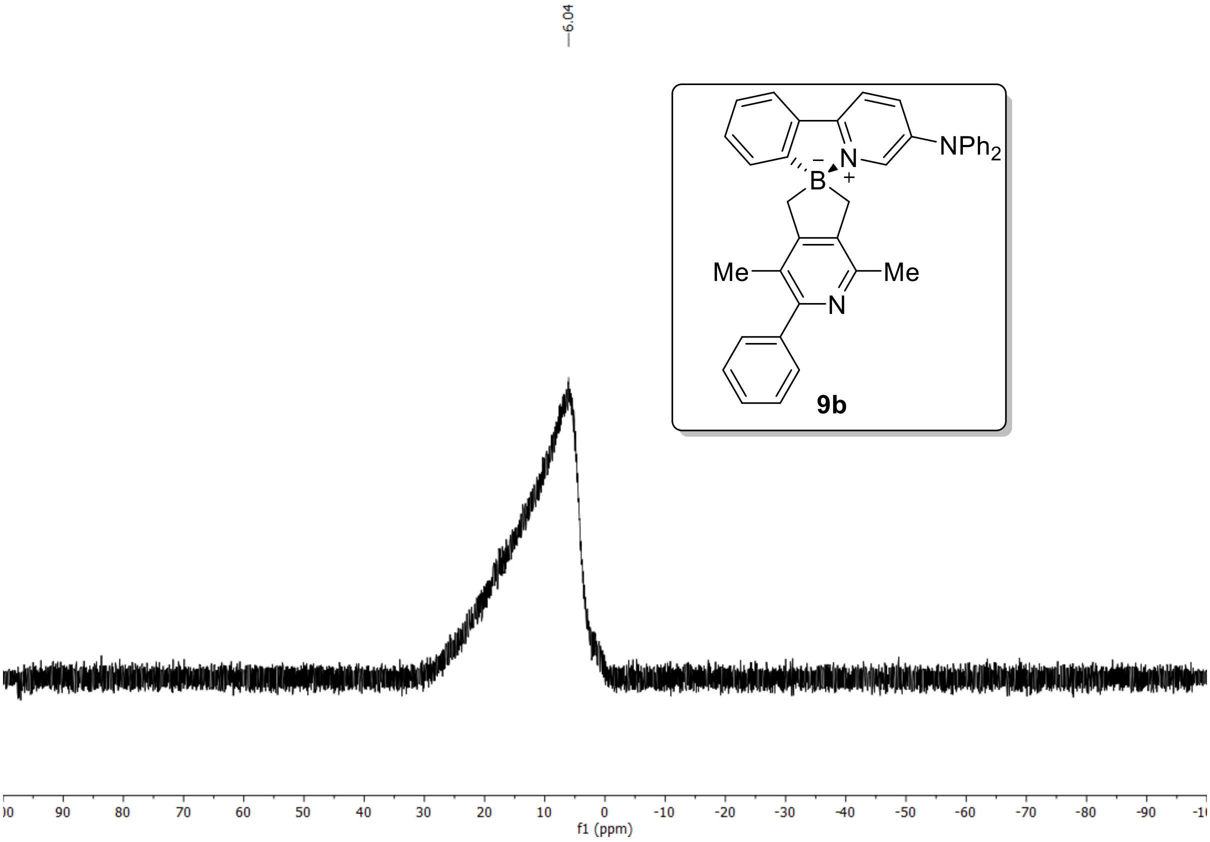

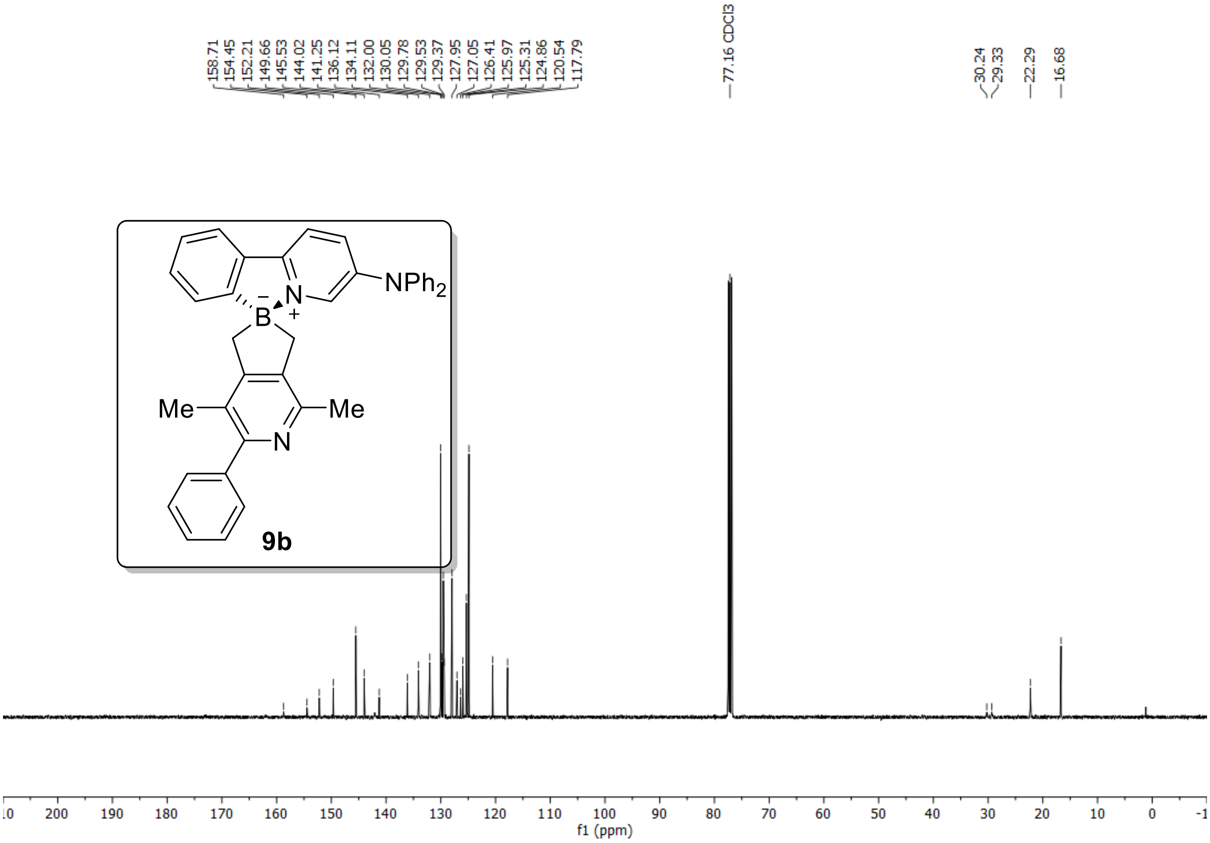
158.71 154.45 152.21 149.66 145.53 144.02 141.25 136.12 134.11 132.00 130.05 129.78 129.53 129.37 127.95 127.05 126.41 125.97 125.31 124.86 120.54 117.79
77.16 CDCl3
30.24 29.33
22.29
16.68
NPh2
N
B
Me
Me
N
9b
f1 (ppm)

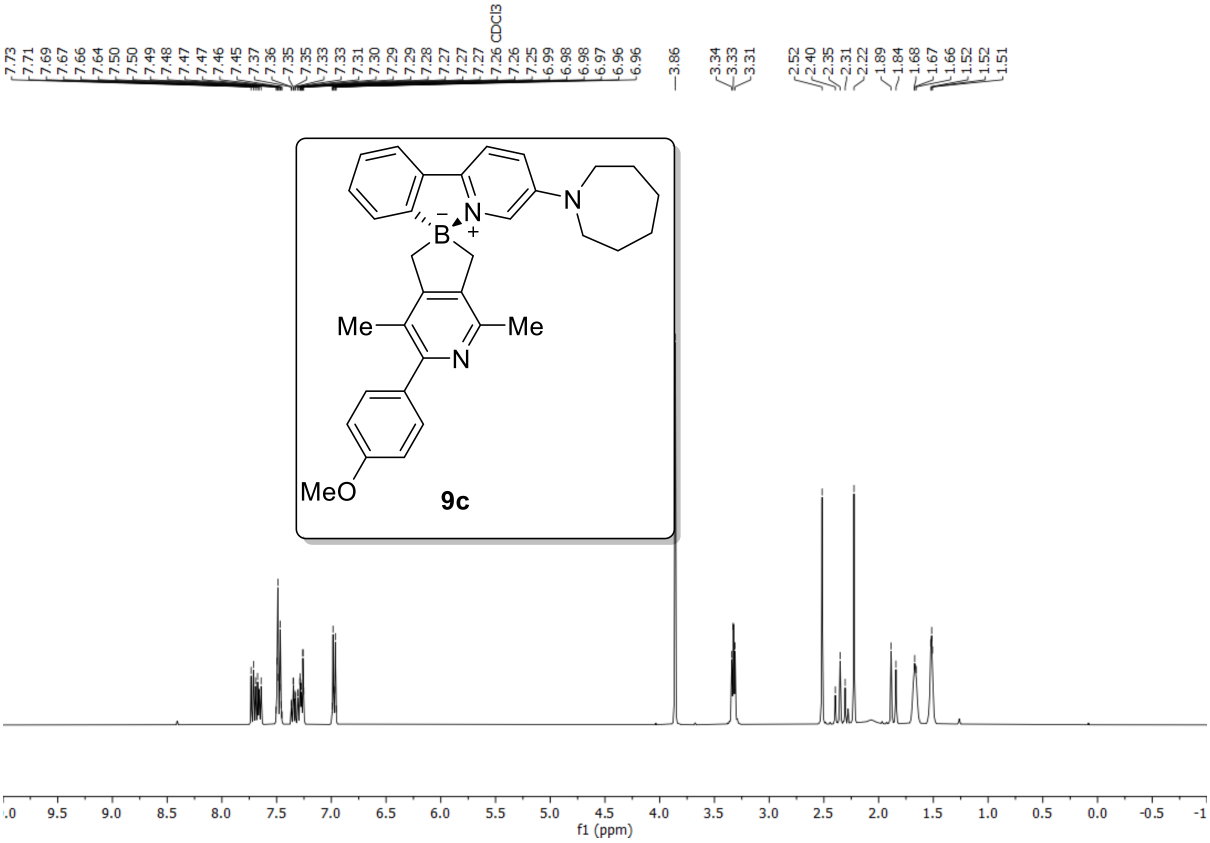
7.73 7.71 7.69 7.67 7.66 7.64 7.50 7.50 7.49 7.48 7.47 7.46 7.45 7.37 7.36 7.35 7.35 7.33 7.33 7.31 7.30 7.29 7.29 7.28 7.27 7.27 7.27 7.26 CDCl3 7.26 7.25 6.99 6.98 6.98 6.97 6.96 6.96
3.86
3.34 3.33 3.31
2.52 2.40 2.35 2.31 2.22 1.89 1.84 1.68 1.67 1.66 1.52 1.52 1.51
N
N
B
Me
Me
N
MeO
9c
f1 (ppm)

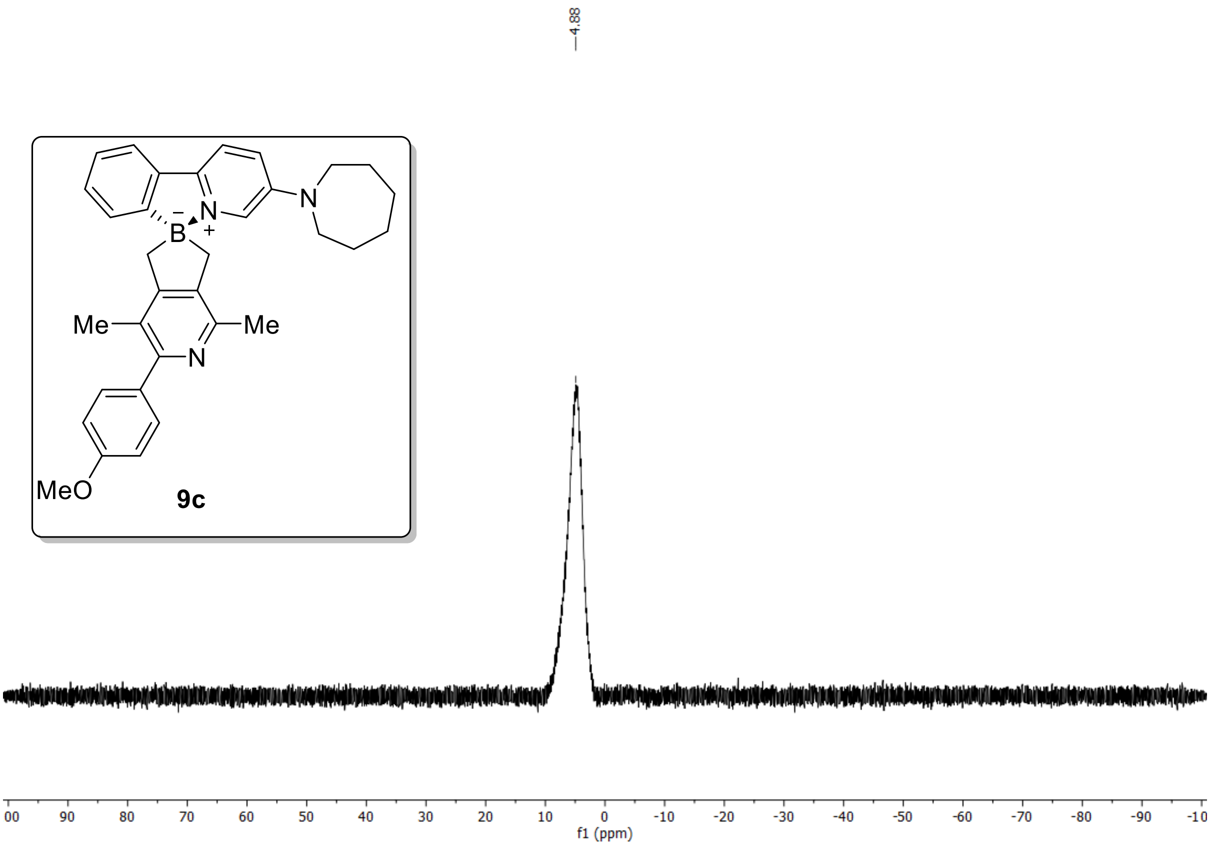
4.88
9c
00 90 80 70 60 50 40 30 20 10 0 -10 -20 -30 -40 -50 -60 -70 -80 -90 -10
f1 (ppm)

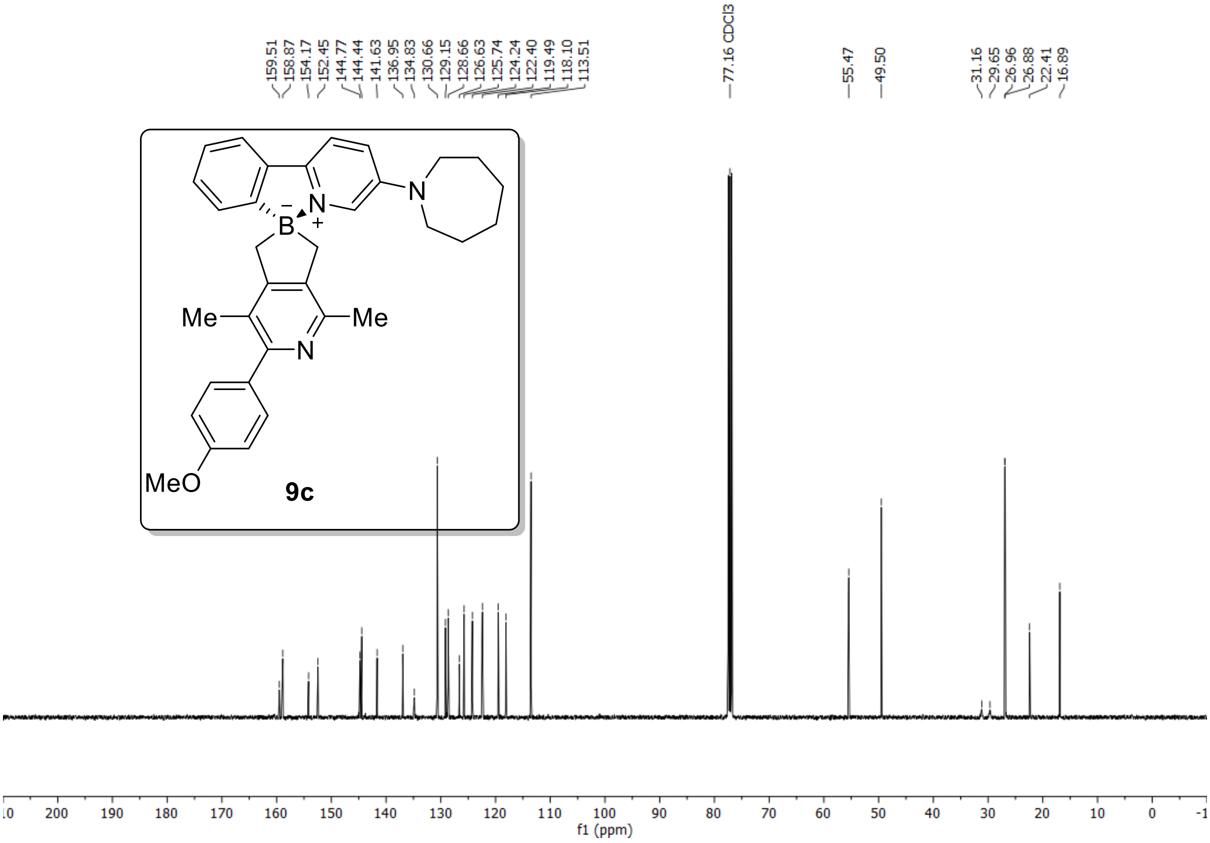
159.51
158.87
154.17
152.45
144.77
144.44
141.63
136.95
134.83
130.66
129.15
128.66
126.63
125.74
124.24
122.40
119.49
118.10
113.51
77.16 CDCl3
55.47
49.50
31.16
29.65
26.96
26.88
22.41
16.89
9c
f1 (ppm)

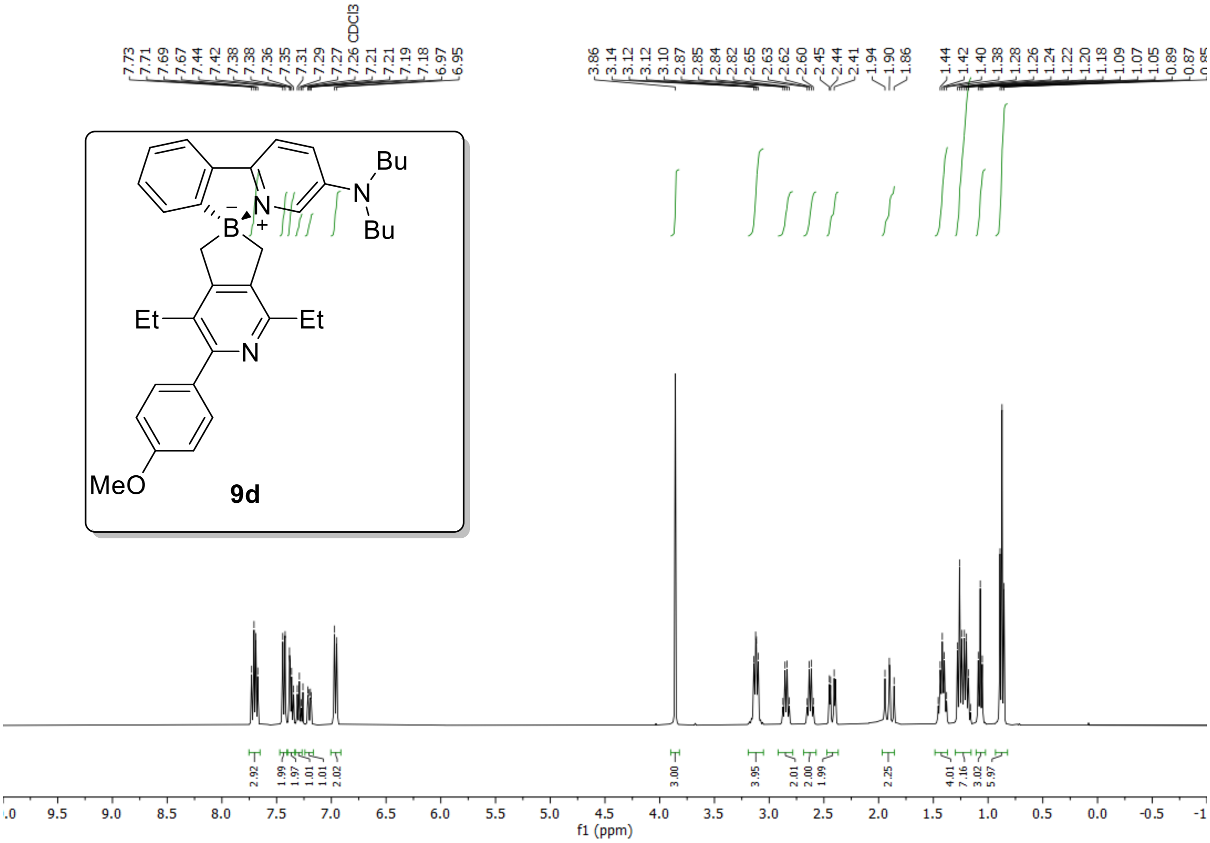
7.73
7.71
7.69
7.67
7.44
7.42
7.38
7.38
7.36
7.35
7.31
7.29
7.27
7.26 CDCl3
7.21
7.21
7.19
7.18
6.97
6.95
3.86
3.14
3.12
3.12
3.10
2.87
2.85
2.84
2.82
2.65
2.63
2.62
2.60
2.45
2.44
2.41
1.94
1.90
1.86
1.44
1.42
1.40
1.38
1.28
1.26
1.24
1.22
1.20
1.18
1.09
1.07
1.05
0.89
0.87
Bu
N
Bu
B
N
Et
Et
N
MeO
9d
2.92
1.99
1.97
1.01
1.01
2.02
3.00
3.95
2.01
2.00
1.99
2.25
4.01
7.16
3.02
5.97
f1 (ppm)

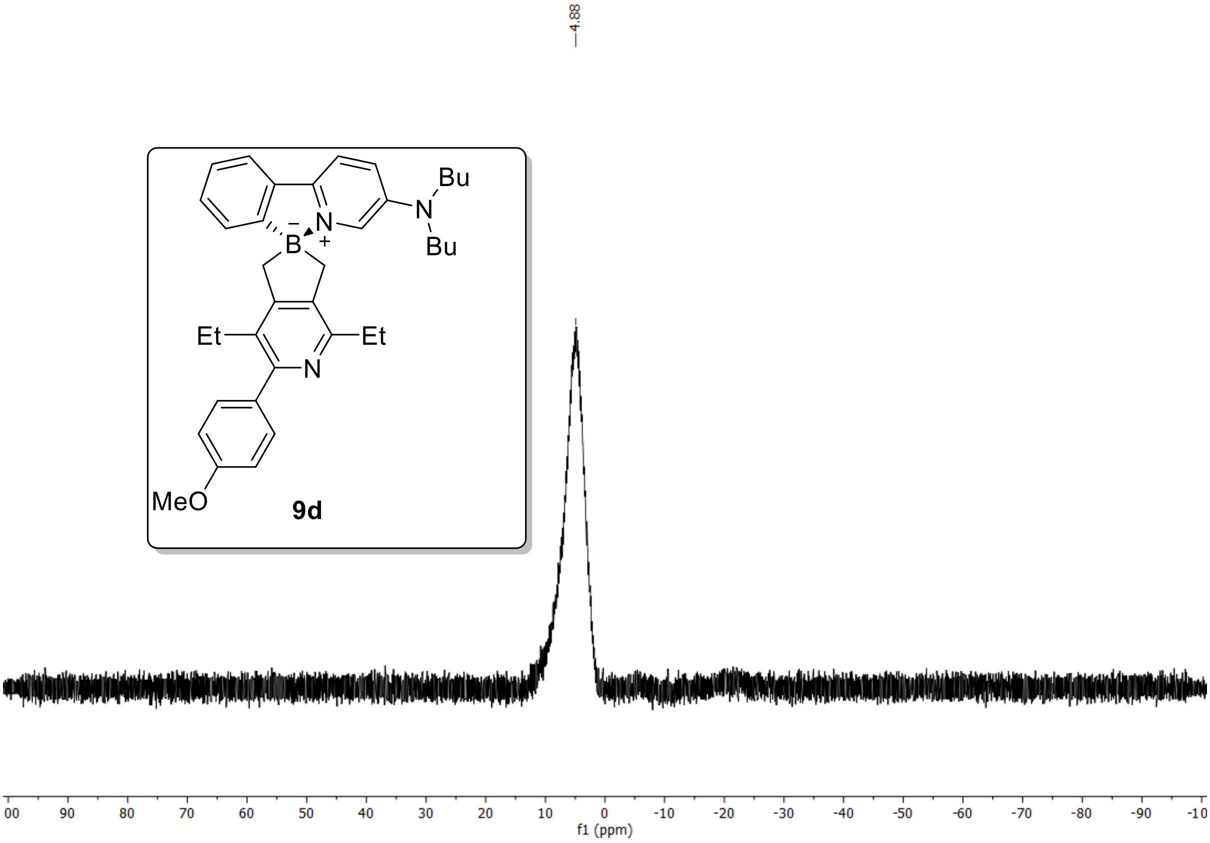
4.88
Bu
N
Bu
B
N
Et
Et
N
MeO
9d
f1 (ppm)

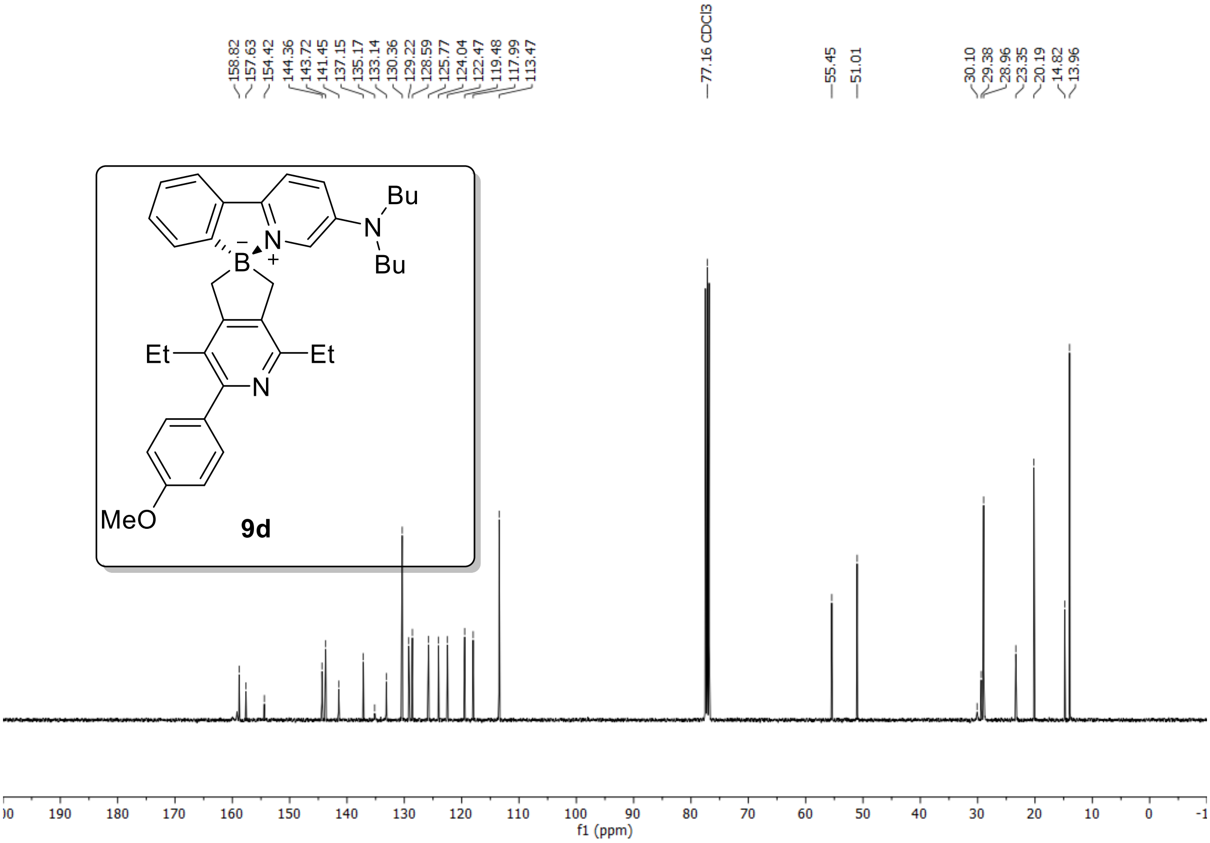

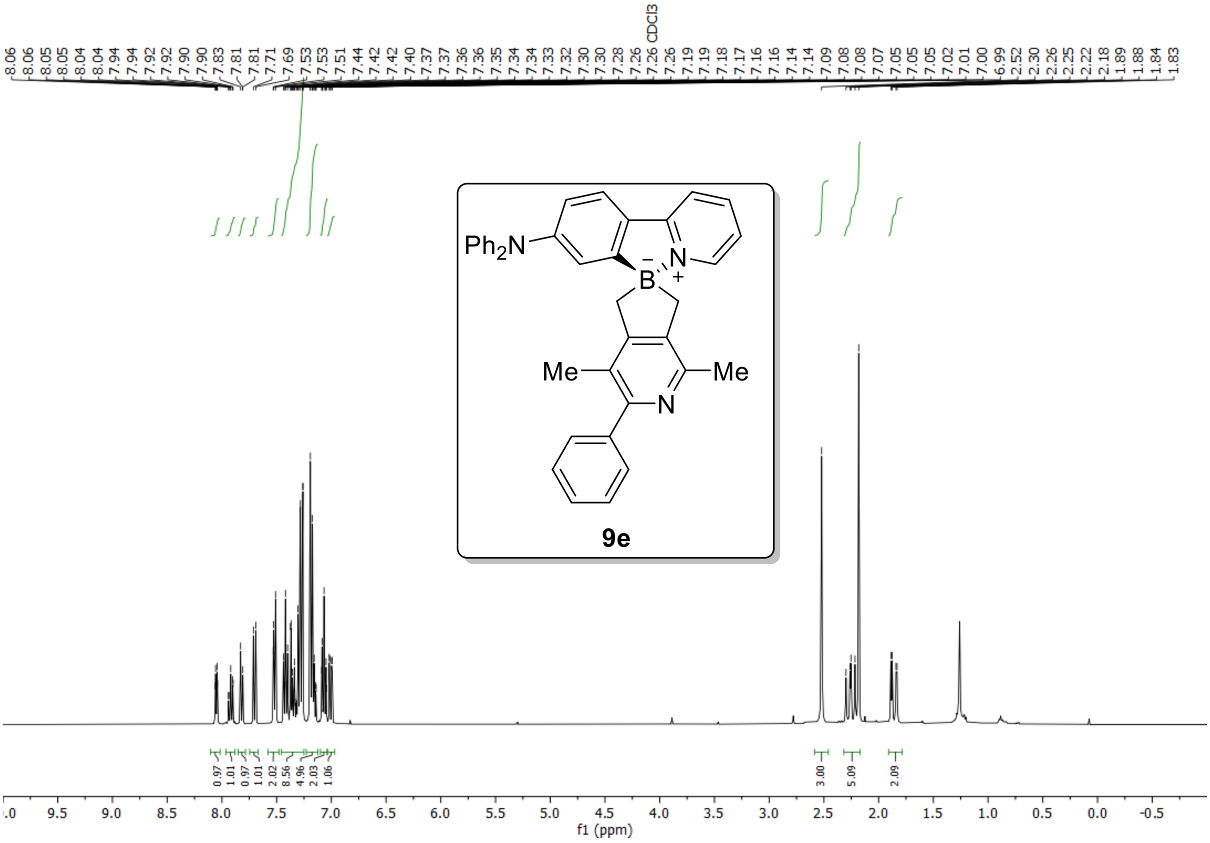

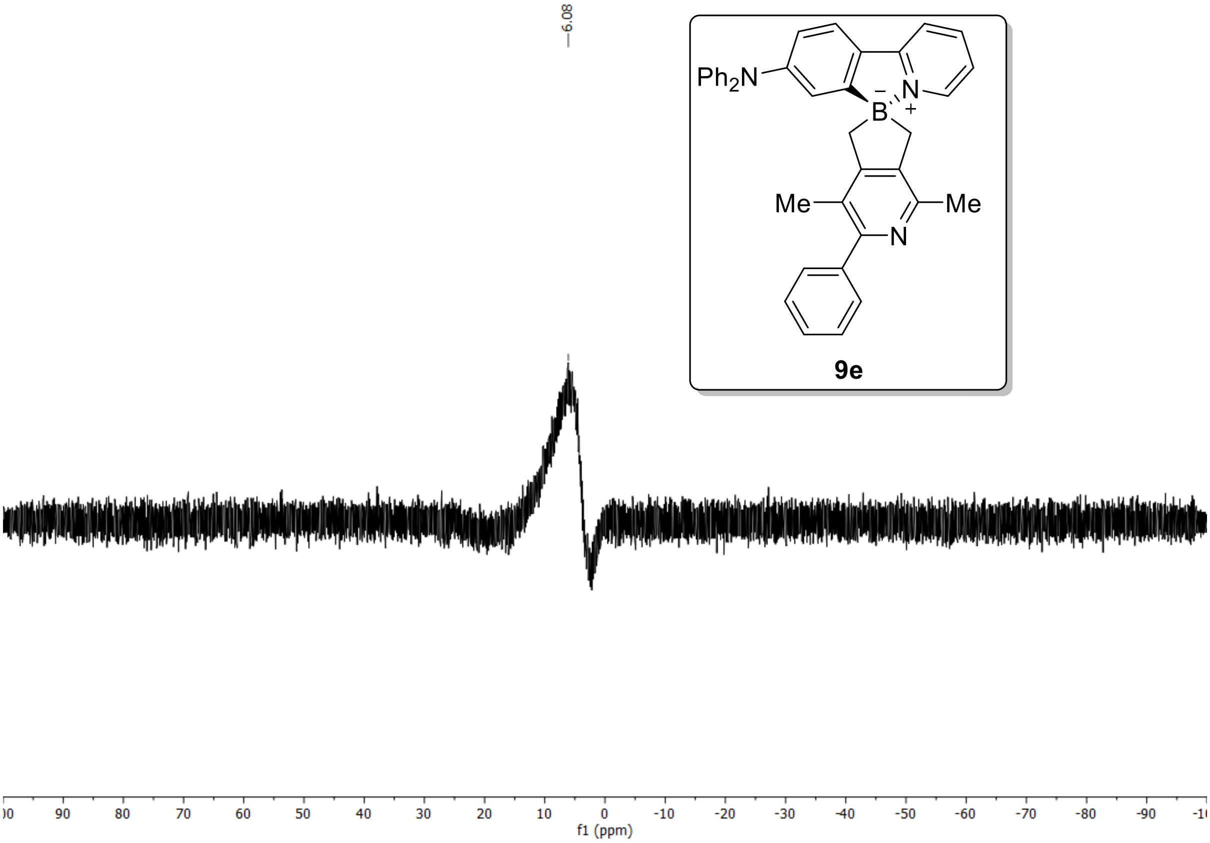
6.08
Ph2N
B
N
Me
Me
N
9e
f1 (ppm)

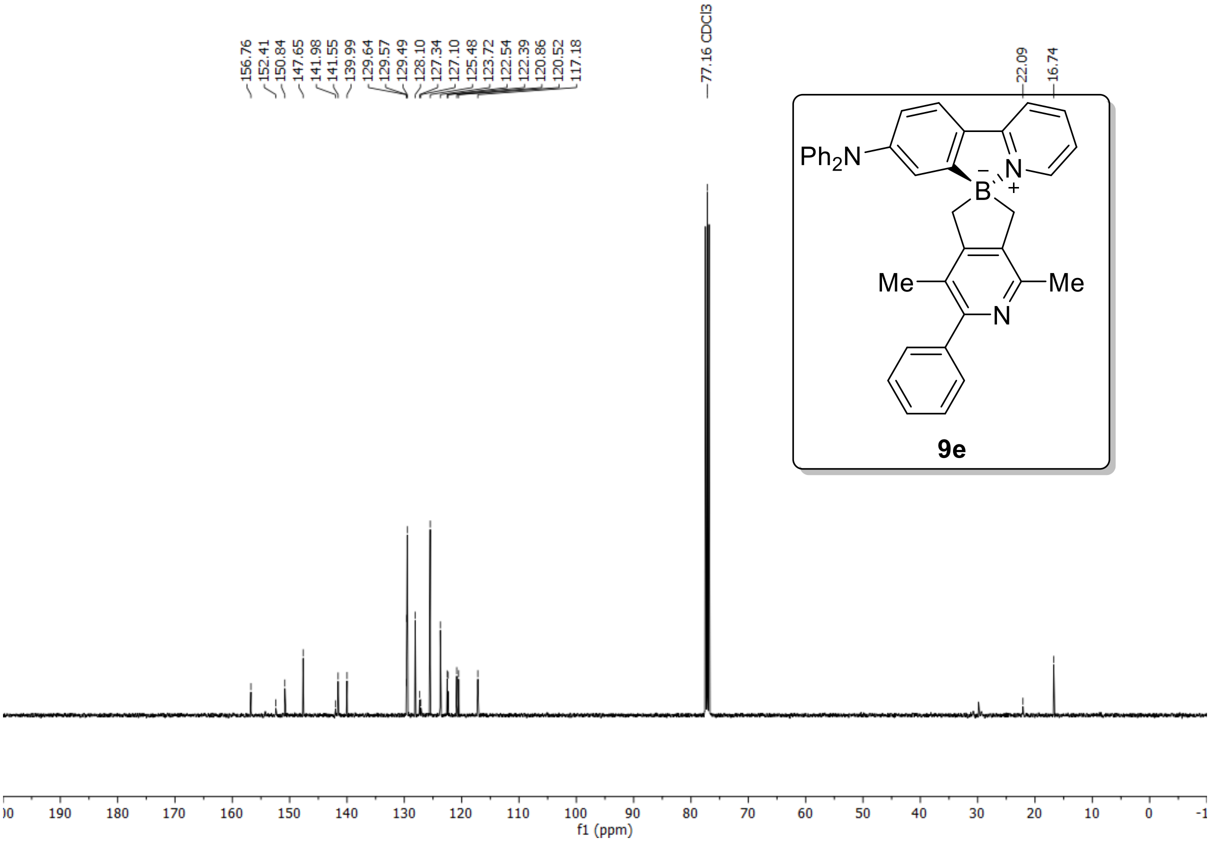
156.76
152.41
150.84
147.65
141.98
141.55
139.99
129.64
129.57
129.49
128.10
127.34
127.10
125.48
123.72
122.54
122.39
120.86
120.52
117.18
77.16 CDCl3
22.09
16.74
Ph2N
B
N
Me
Me
N
9e
f1 (ppm)

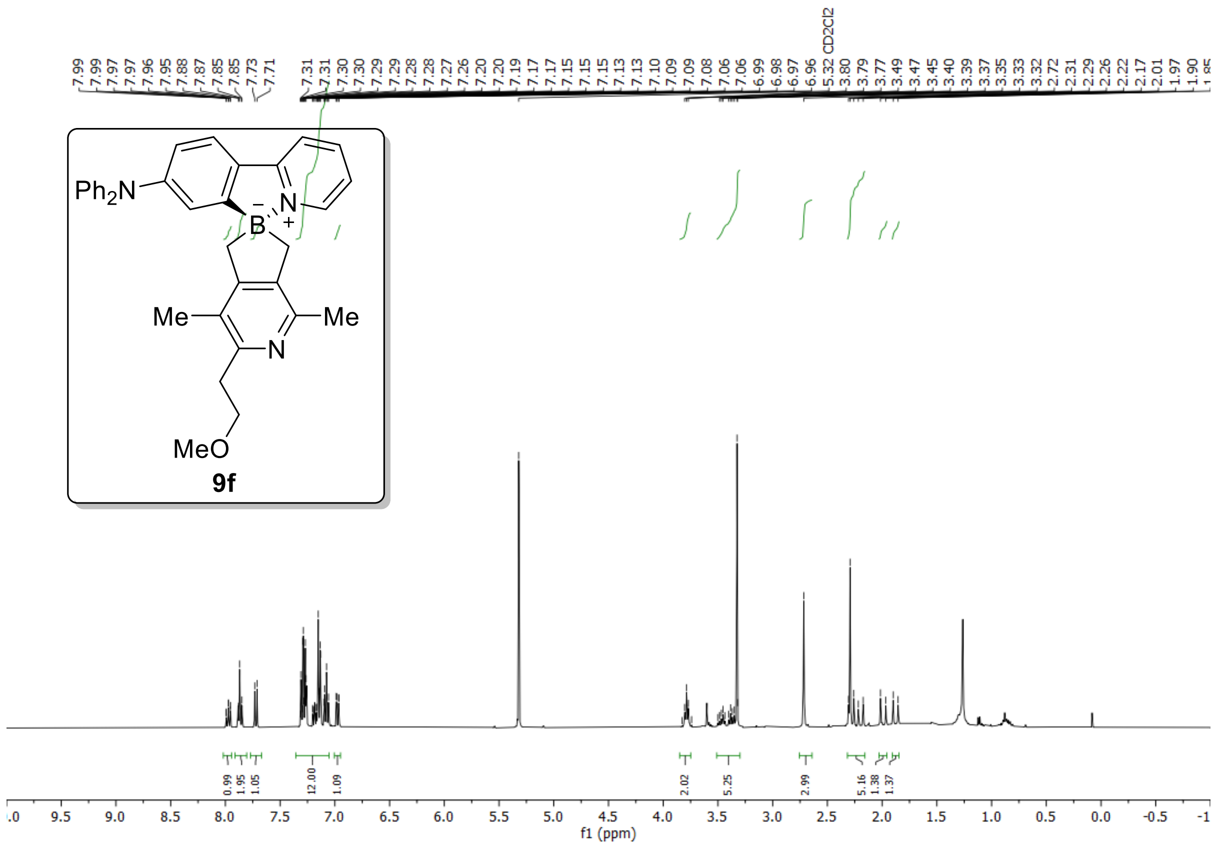

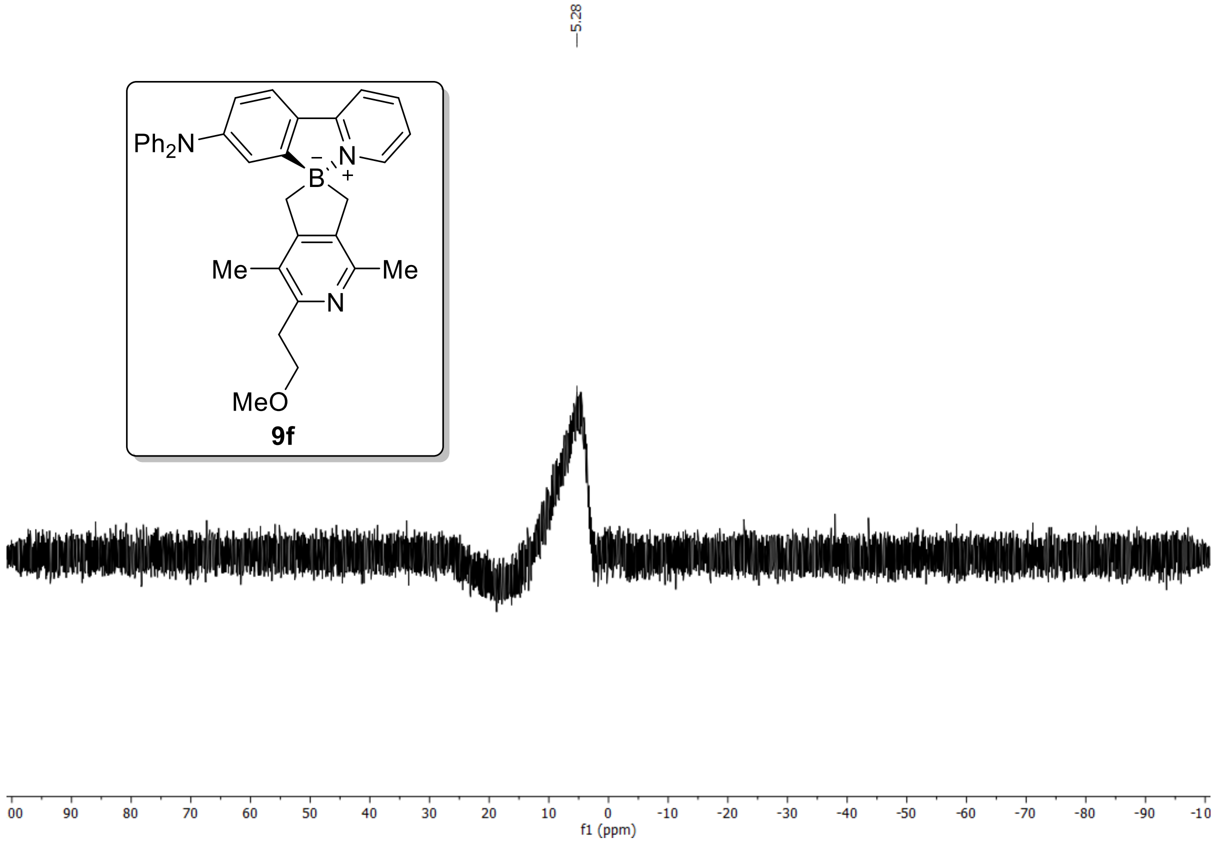

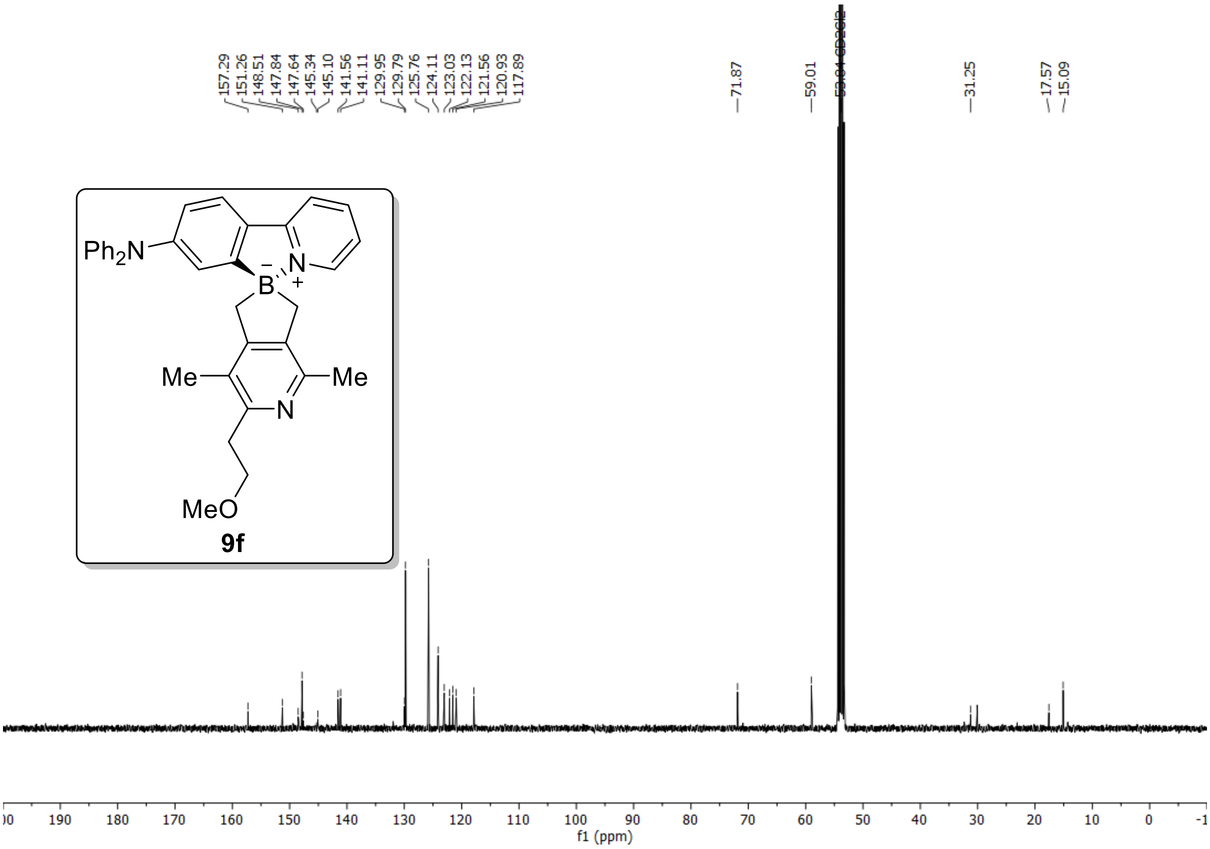
157.29
151.26
148.51
147.84
147.64
145.34
145.10
141.56
141.11
129.95
129.79
125.76
124.11
123.03
122.13
121.56
120.93
117.89
71.87
59.01
31.25
17.57
15.09
Ph2N
B
N
Me
Me
N
MeO
9f
f1 (ppm)

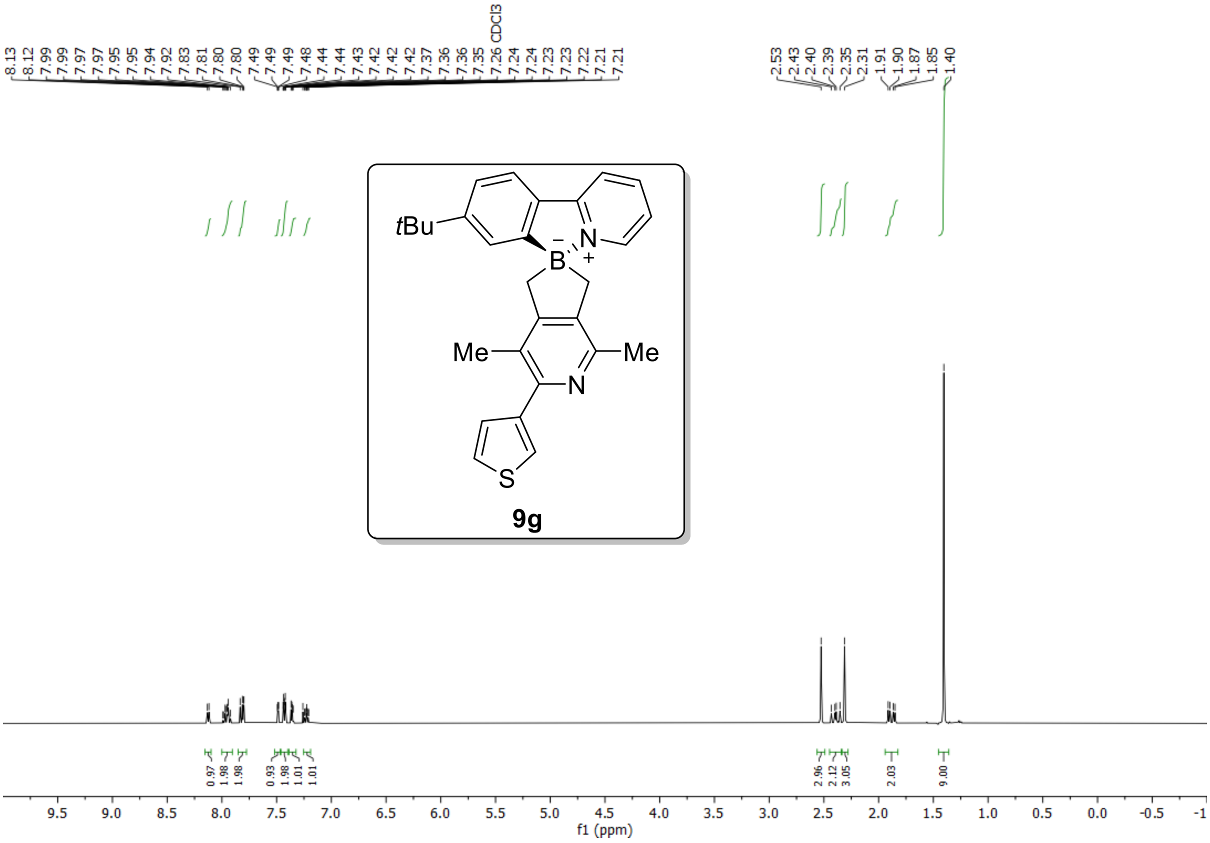
8.13
8.12
7.99
7.99
7.97
7.97
7.95
7.95
7.94
7.92
7.83
7.81
7.80
7.80
7.49
7.49
7.49
7.48
7.44
7.43
7.42
7.42
7.42
7.37
7.36
7.35
7.26 CDCl3
7.24
7.24
7.23
7.23
7.22
7.21
7.21
2.53
2.43
2.40
2.39
2.35
2.31
1.91
1.90
1.87
1.85
1.40
tBu
B
N
Me
Me
N
S
9g
0.97
1.98
1.98
0.93
1.98
1.01
1.01
2.96
2.12
3.05
2.03
9.00
f1 (ppm)

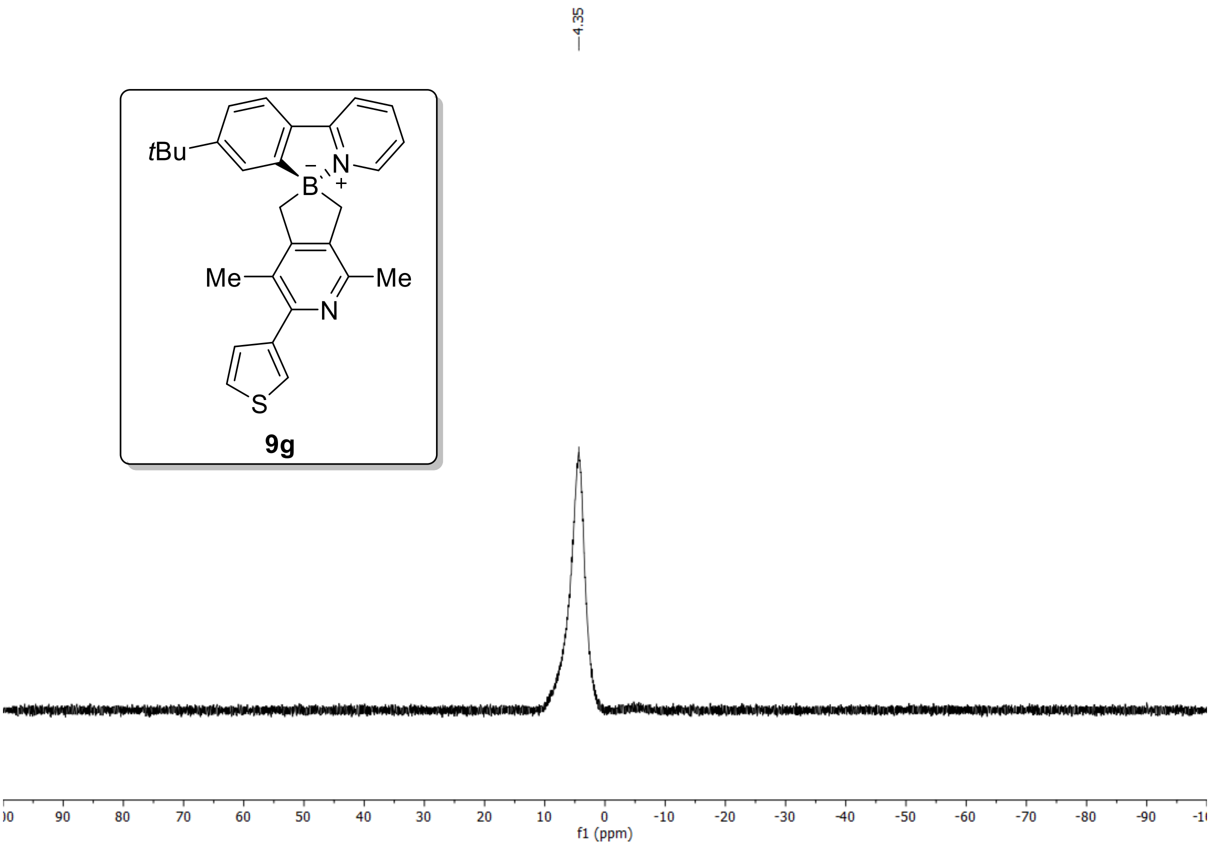

4.35
tBu
B
N
Me
Me
N
S
9g
f1 (ppm)


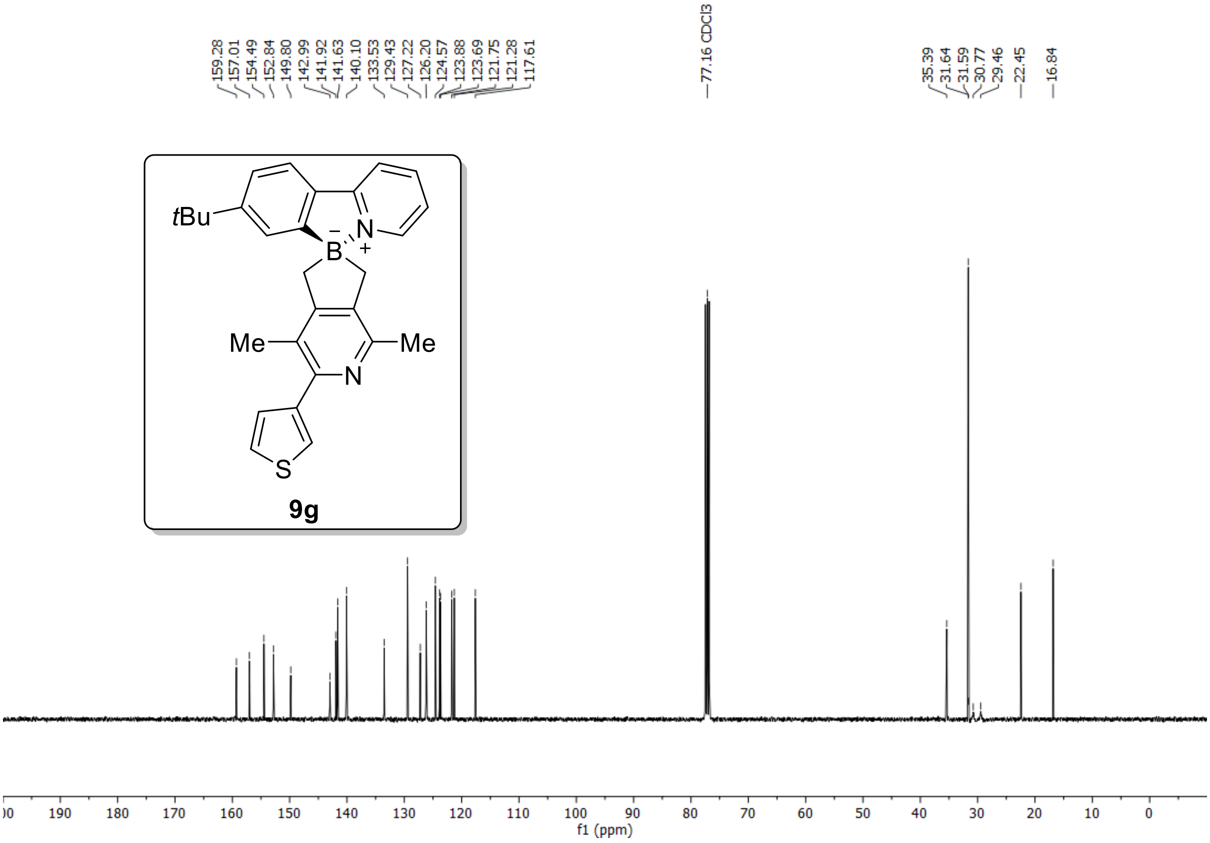

159.28
157.01
154.49
152.84
149.80
142.99
141.92
141.63
140.10
133.53
129.43
127.22
126.20
124.57
123.88
123.69
121.75
121.28
117.61
77.16 CDCl3
35.39
31.64
31.59
30.77
29.46
22.45
16.84
tBu
B
N
Me
Me
N
S
9g
f1 (ppm)

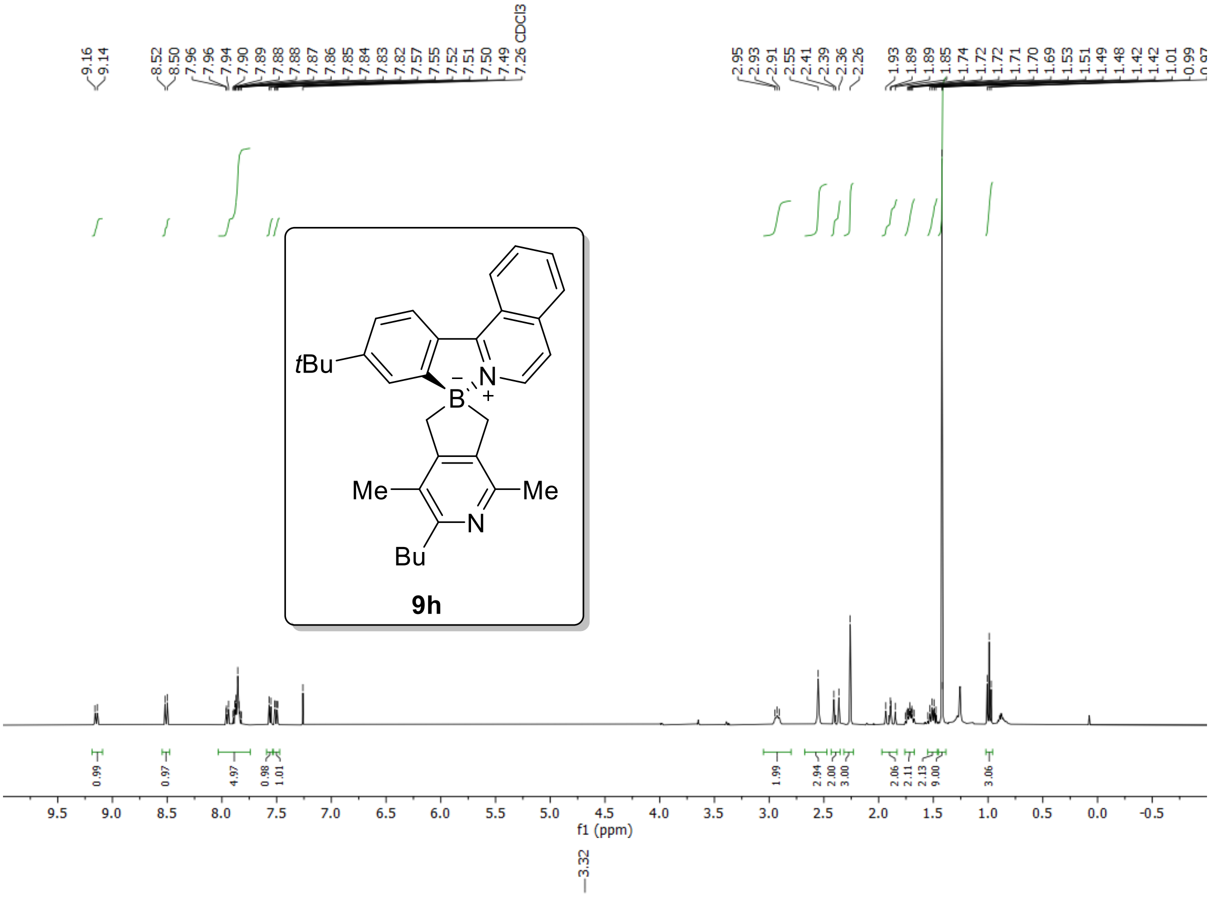

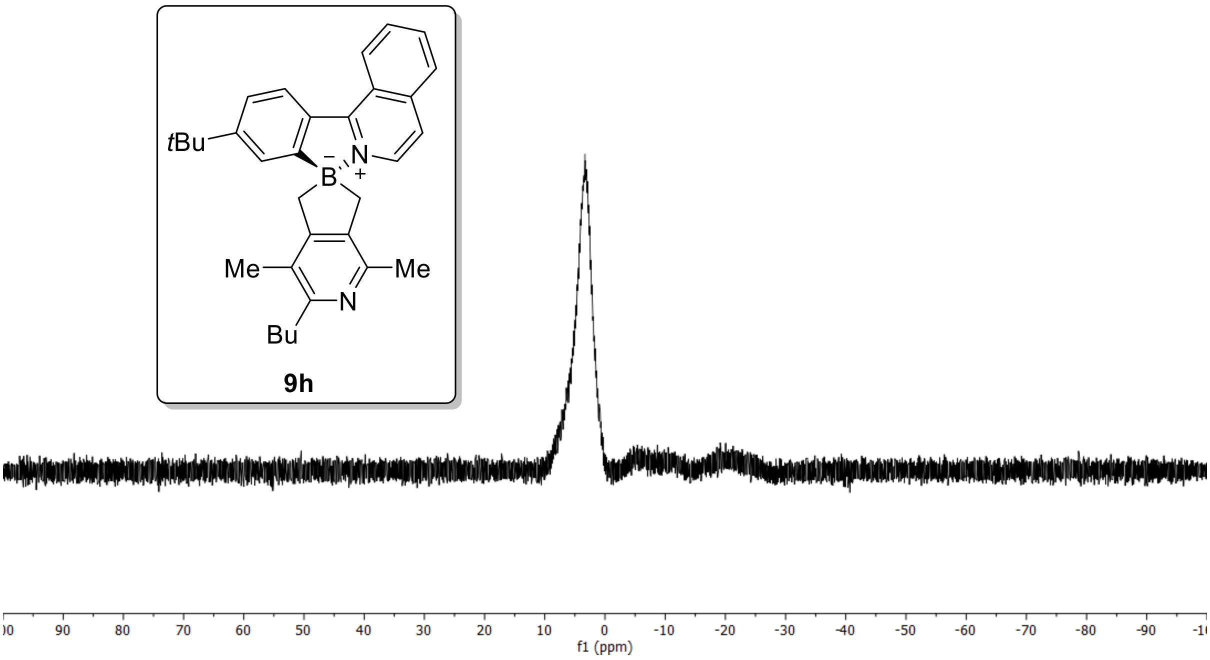

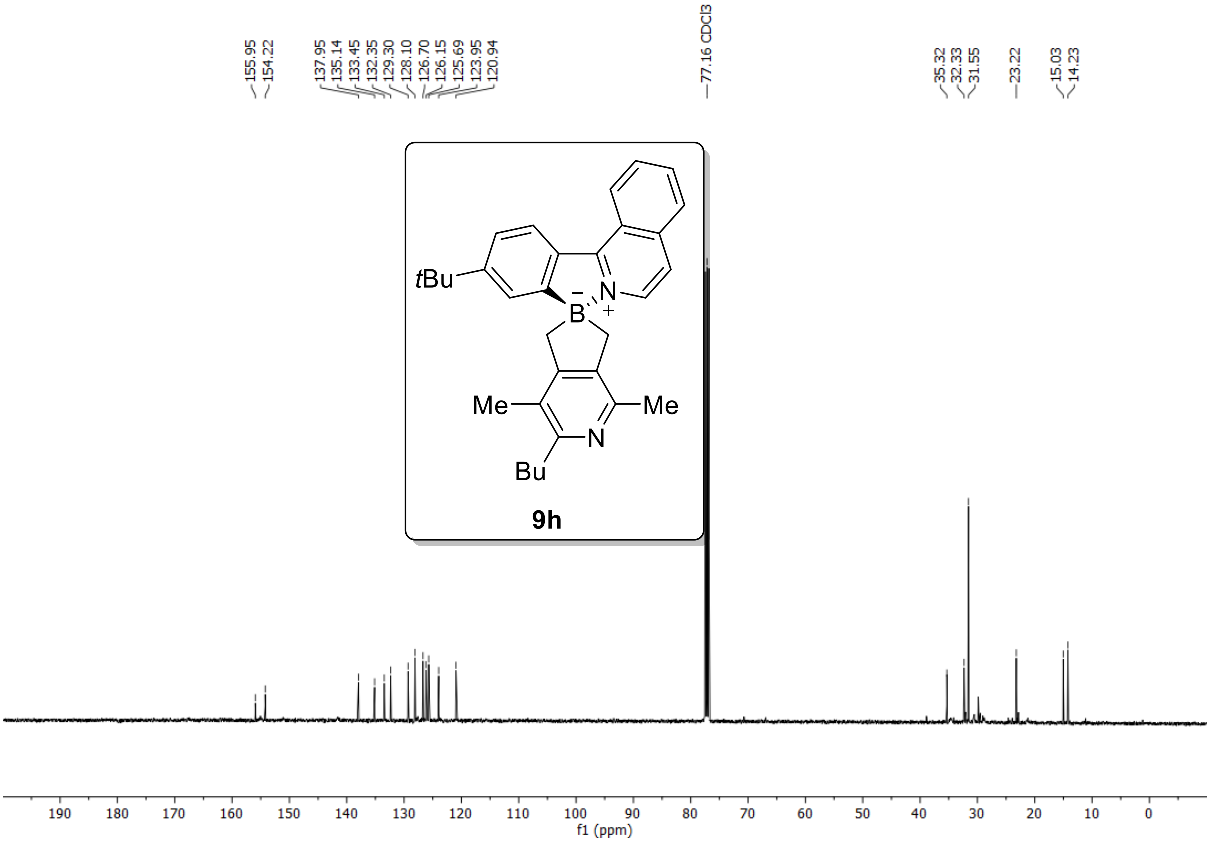
155.95
154.22
137.95
135.14
133.45
132.35
129.30
128.10
126.70
126.15
125.69
123.95
120.94
77.16 CDCl3
35.32
32.33
31.55
23.22
15.03
14.23
tBu
B
N
Me
Me
N
Bu
9h
190 180 170 160 150 140 130 120 110 100 90 80 70 60 50 40 30 20 10 0
f1 (ppm)

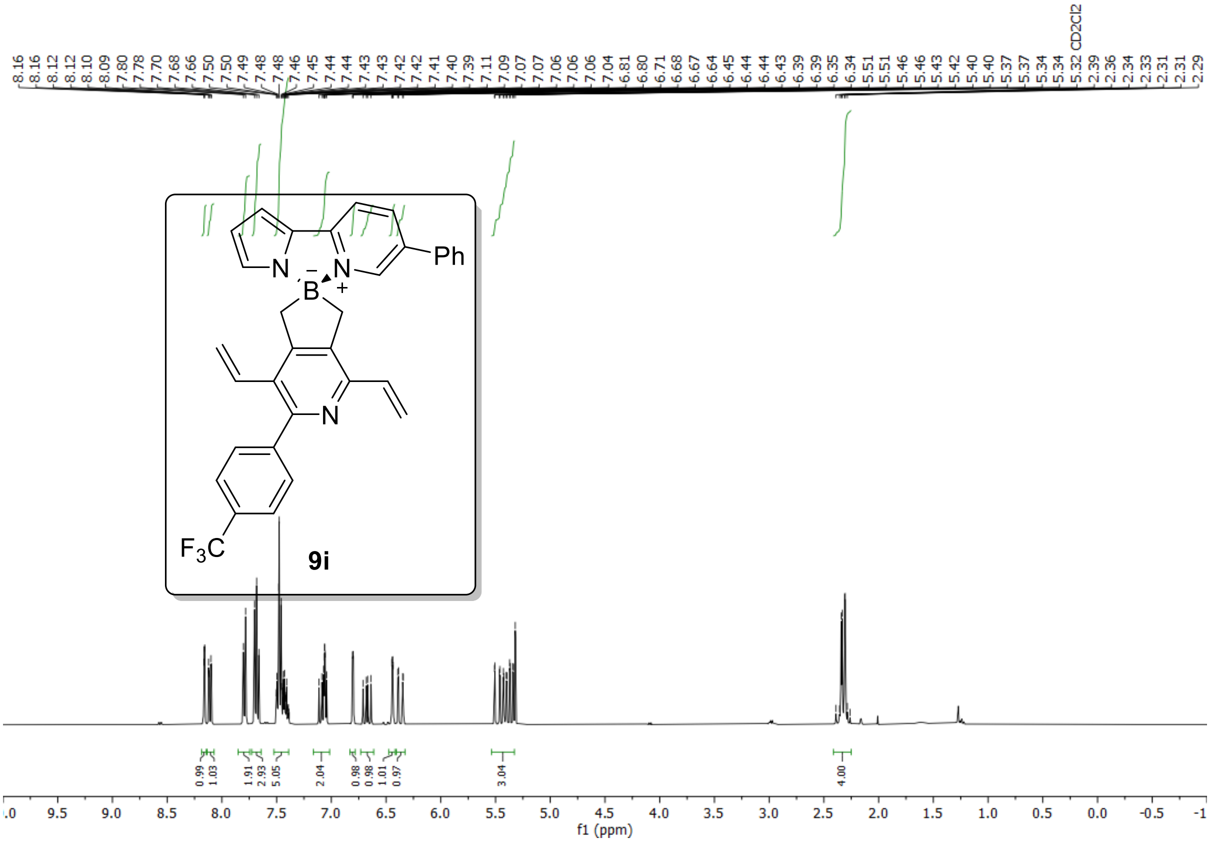
CD2Cl2
N
B
N
Ph
N
F3C
9i
0.99
1.03
1.91
2.93
5.05
2.04
0.98
0.98
1.01
0.97
3.04
4.00
9.5 9.0 8.5 8.0 7.5 7.0 6.5 6.0 5.5 5.0 4.5 4.0 3.5 3.0 2.5 2.0 1.5 1.0 0.5 0.0 -0.5
f1 (ppm)

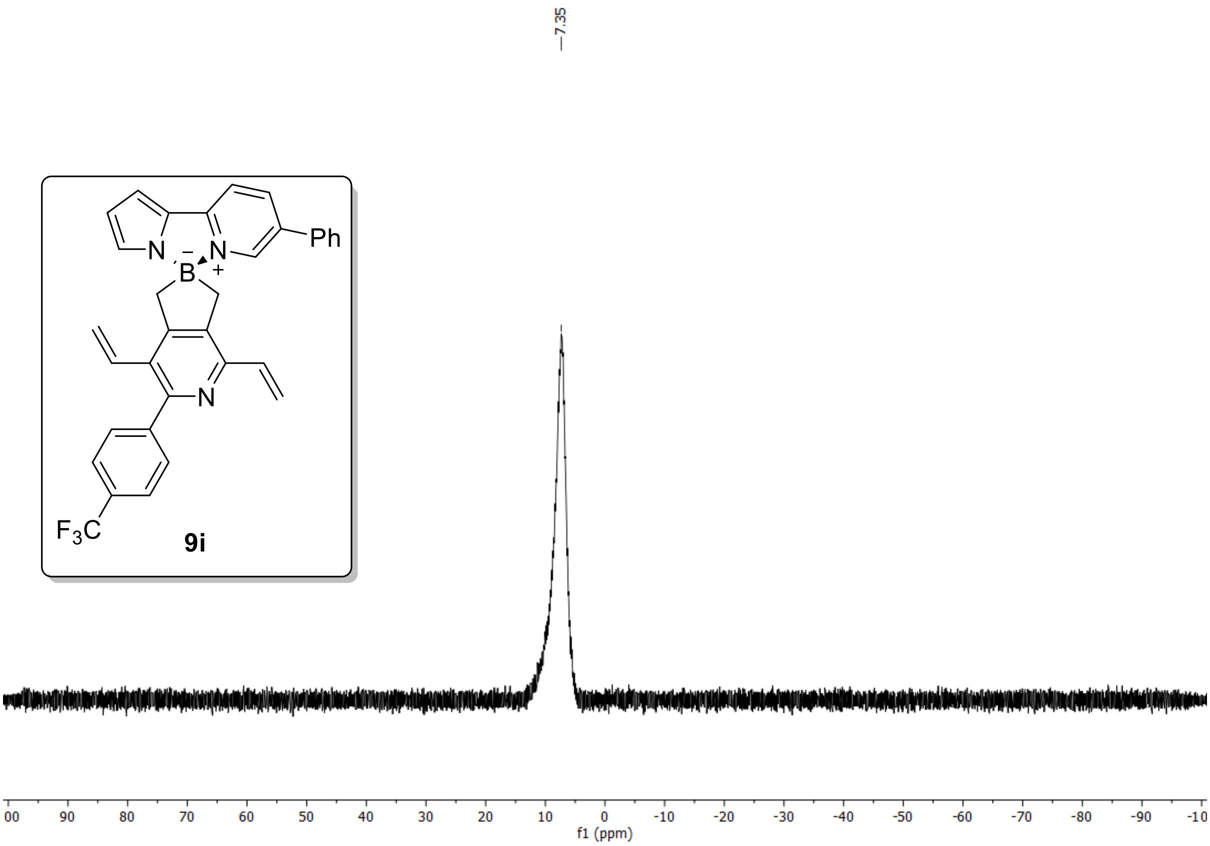
7.35
Ph
N
B
N
F3C
9i
f1 (ppm)

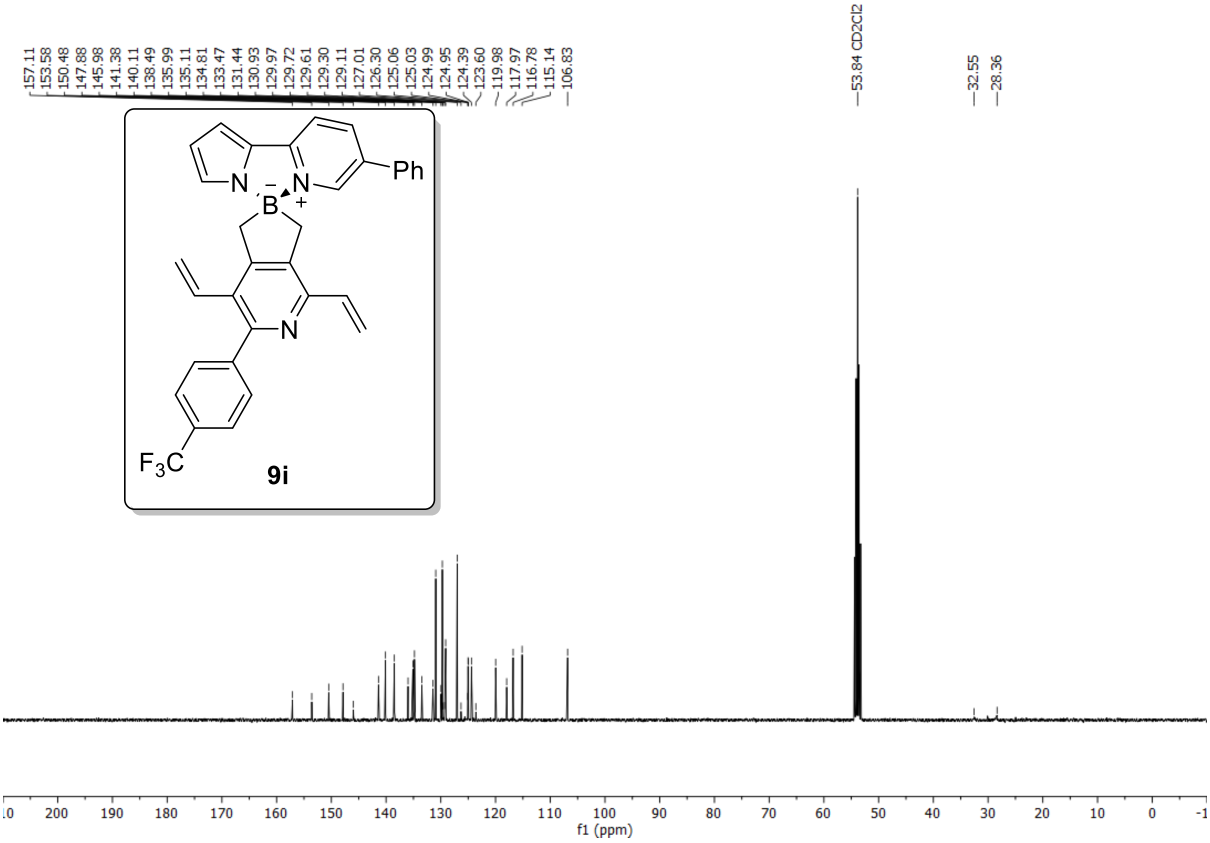
157.11
153.58
150.48
147.88
145.98
141.38
140.11
138.49
135.99
135.11
134.81
133.47
131.44
130.93
129.97
129.72
129.61
129.30
129.11
127.01
126.30
125.06
125.03
124.99
124.95
124.39
123.60
119.98
117.97
116.78
115.14
106.83
53.84 CD2Cl2
32.55
28.36
Ph
N
B
N
F3C
9i
f1 (ppm)

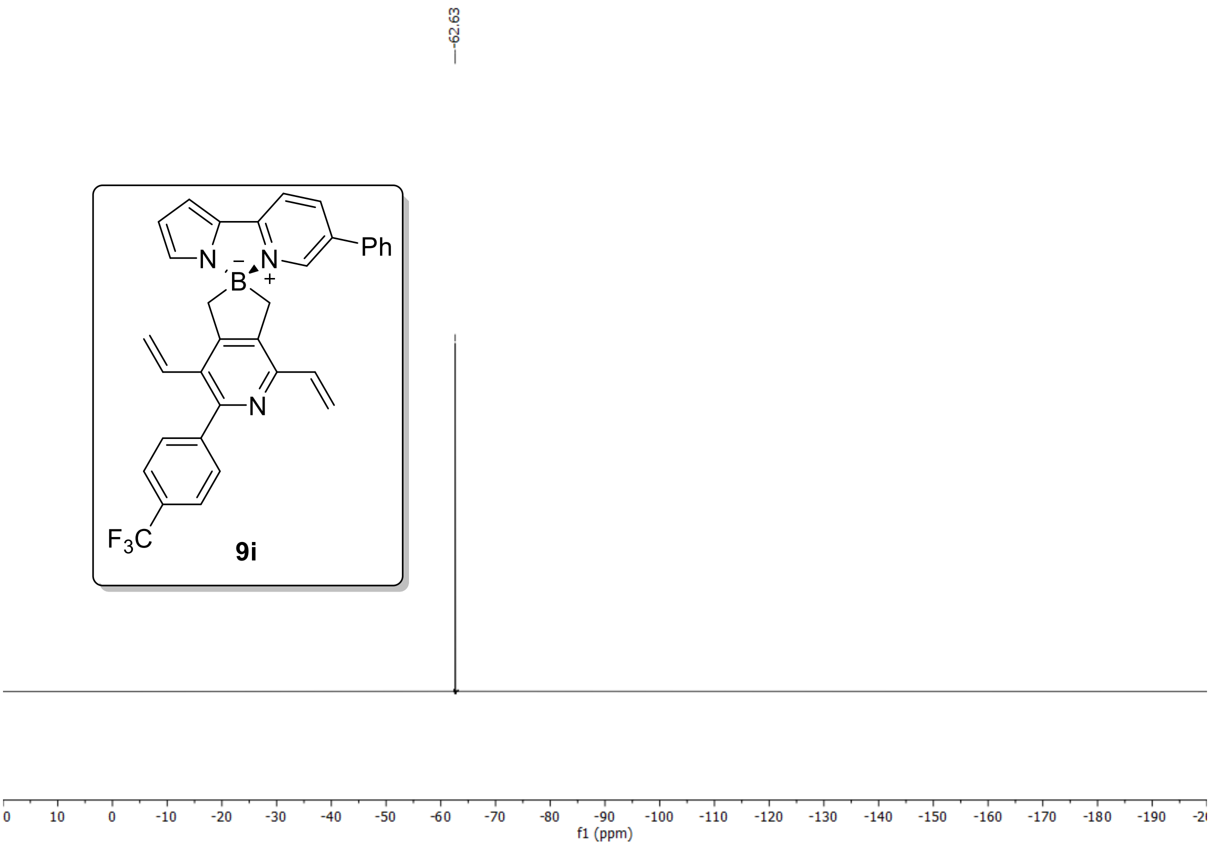

-62.63
Ph
F3C
9i
f1 (ppm)


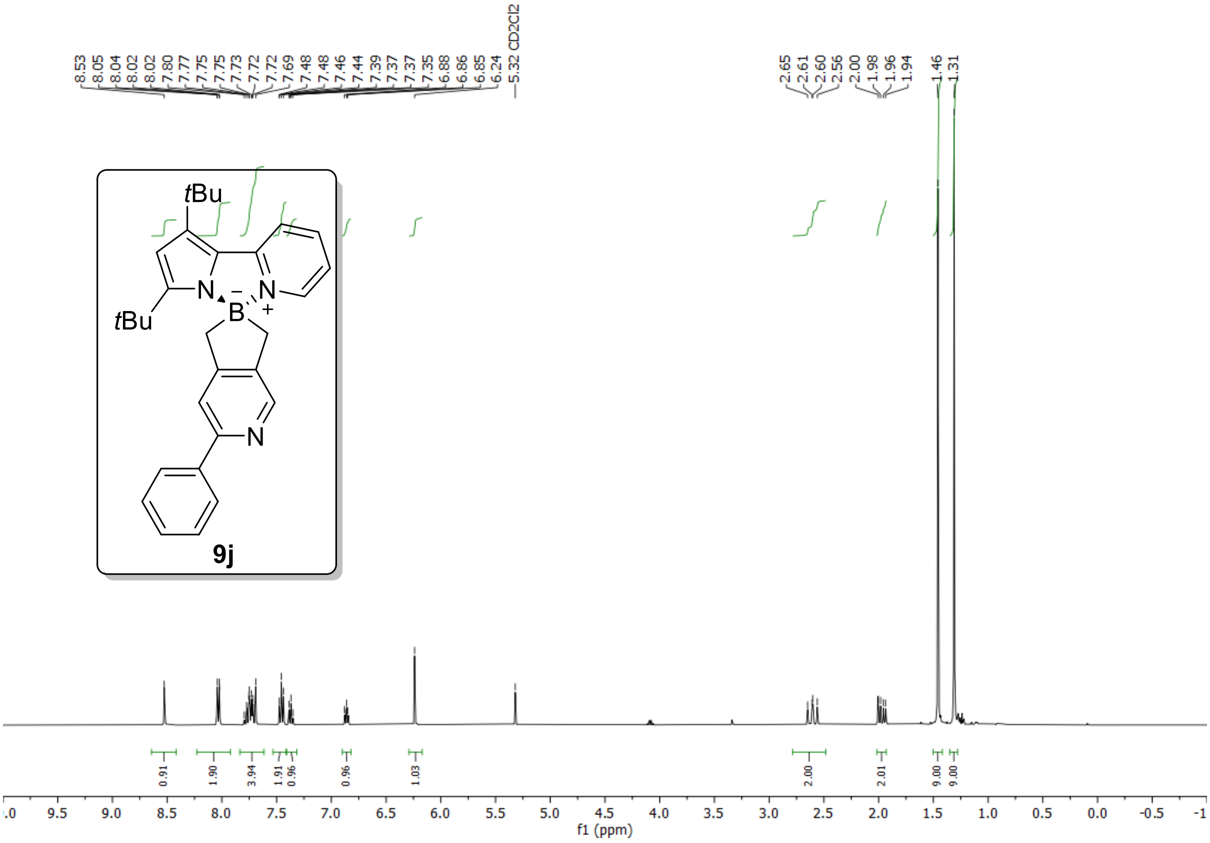

8.53 8.05 8.04 8.02 8.02 7.80 7.77 7.75 7.75 7.73 7.72 7.72 7.69 7.48 7.48 7.46 7.44 7.39 7.37 7.37 7.35 6.88 6.86 6.85 6.24
5.32 CD2Cl2
2.65 2.61 2.60 2.56 2.00 1.98 1.96 1.94
1.46 1.31
tBu
tBu
9j
0.91 1.90 3.94 1.91 0.96 0.96 1.03 2.00 2.01 9.00 9.00
f1 (ppm)

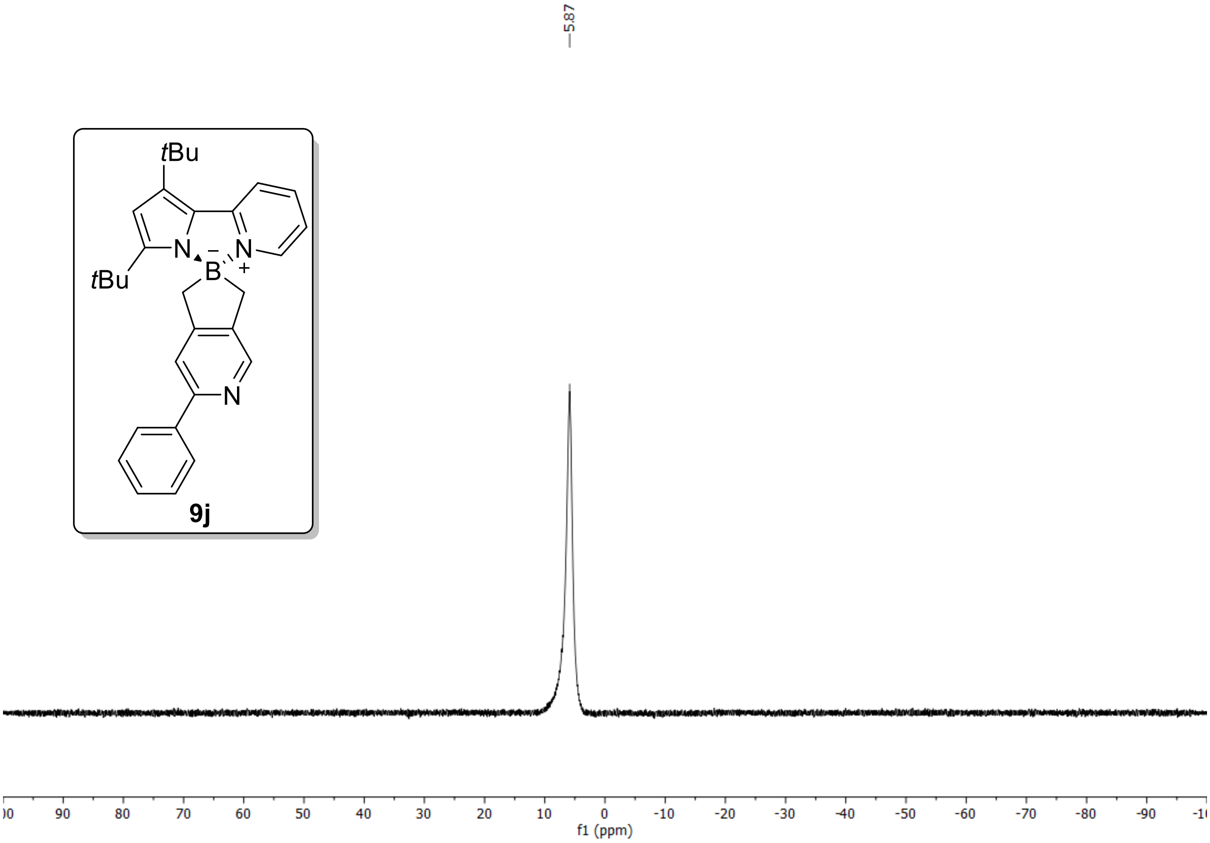

5.87
tBu
N
B
N
tBu
N
9j
f1 (ppm)


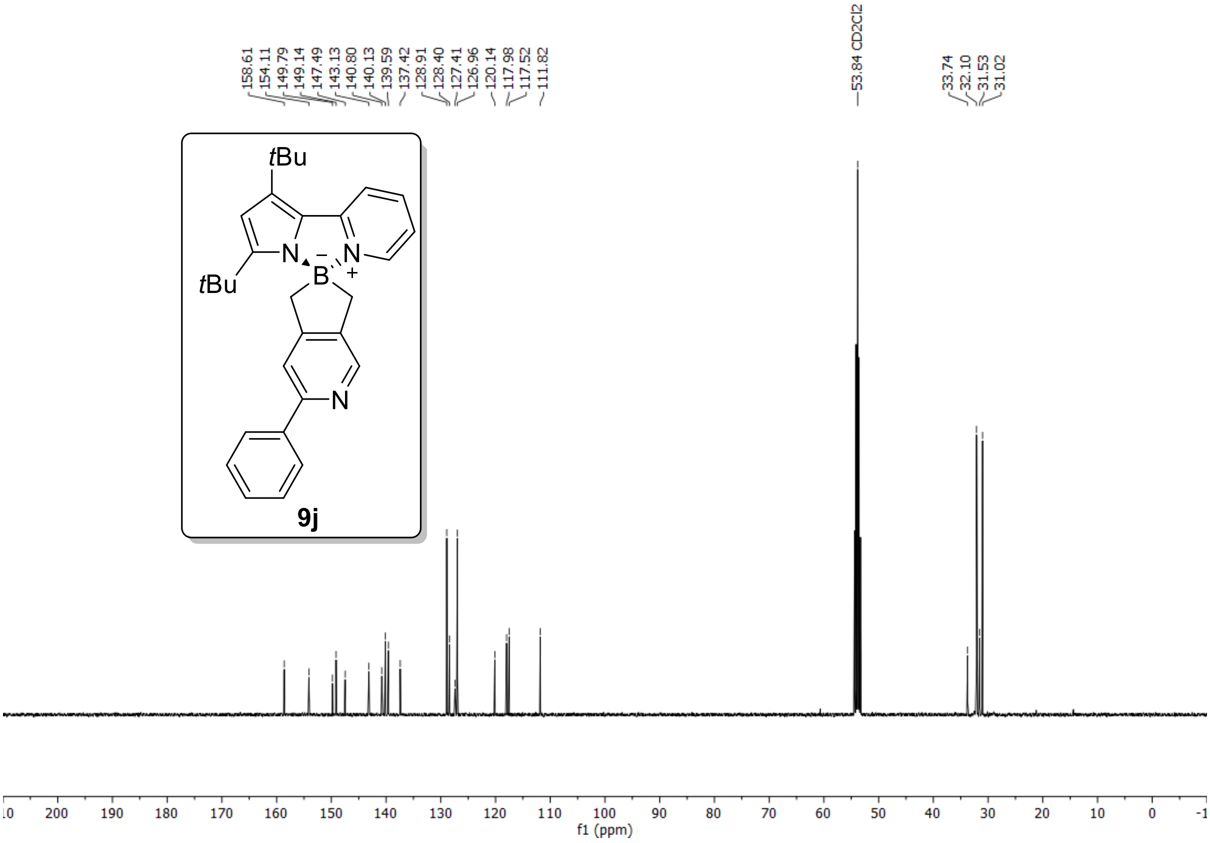

158.61
154.11
149.79
149.14
147.49
143.13
140.80
140.13
139.59
137.42
128.91
128.40
127.41
126.96
120.14
117.98
117.52
111.82
53.84 CD2Cl2
33.74
32.10
31.53
31.02
tBu
N
B
N
tBu
N
9j
f1 (ppm)

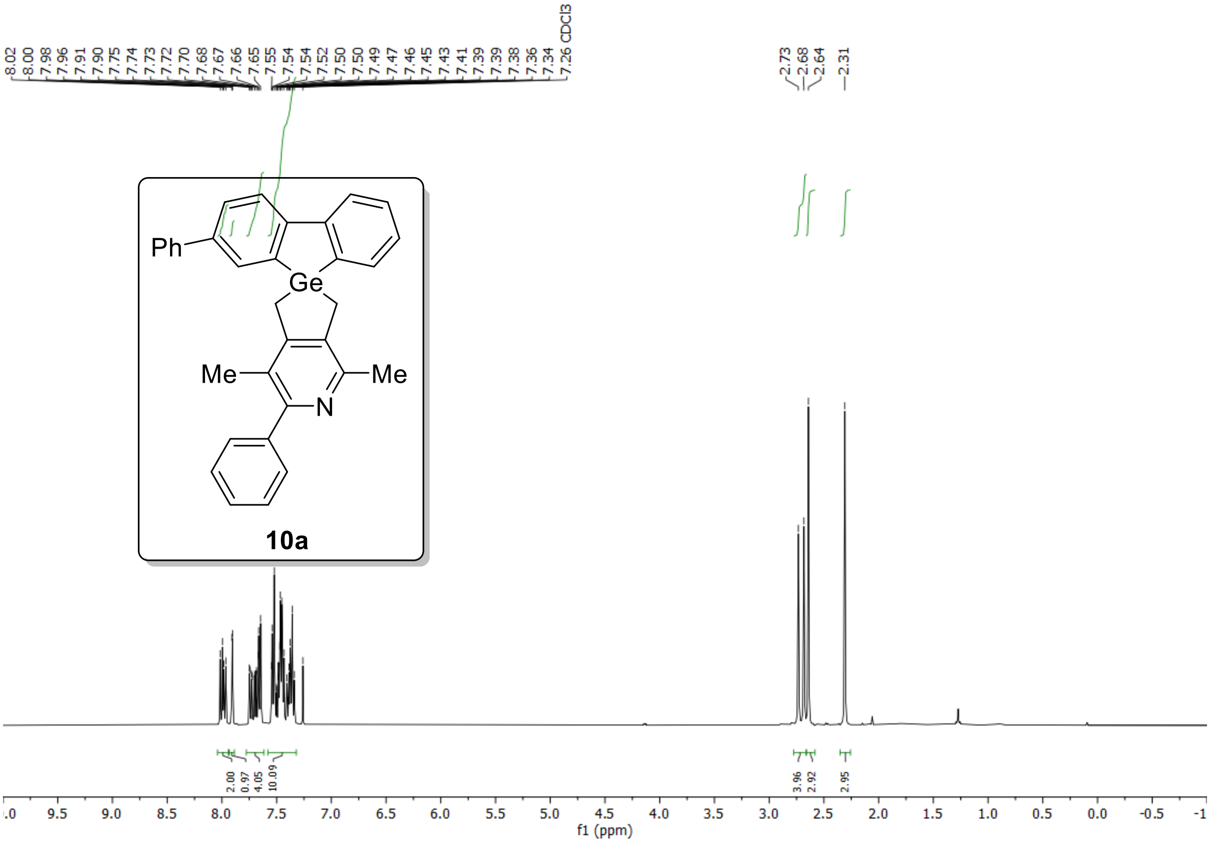
8.02 8.00 7.98 7.96 7.91 7.90 7.75 7.74 7.73 7.72 7.70 7.68 7.67 7.66 7.65 7.55 7.54 7.54 7.52 7.50 7.50 7.49 7.47 7.46 7.45 7.43 7.41 7.39 7.39 7.38 7.36 7.34 7.26 CDCl3
2.73 2.68 2.64 2.31
Ph
Ge
Me
Me
N
10a
2.00 0.97 4.05 10.09
3.96 2.92 2.95
f1 (ppm)

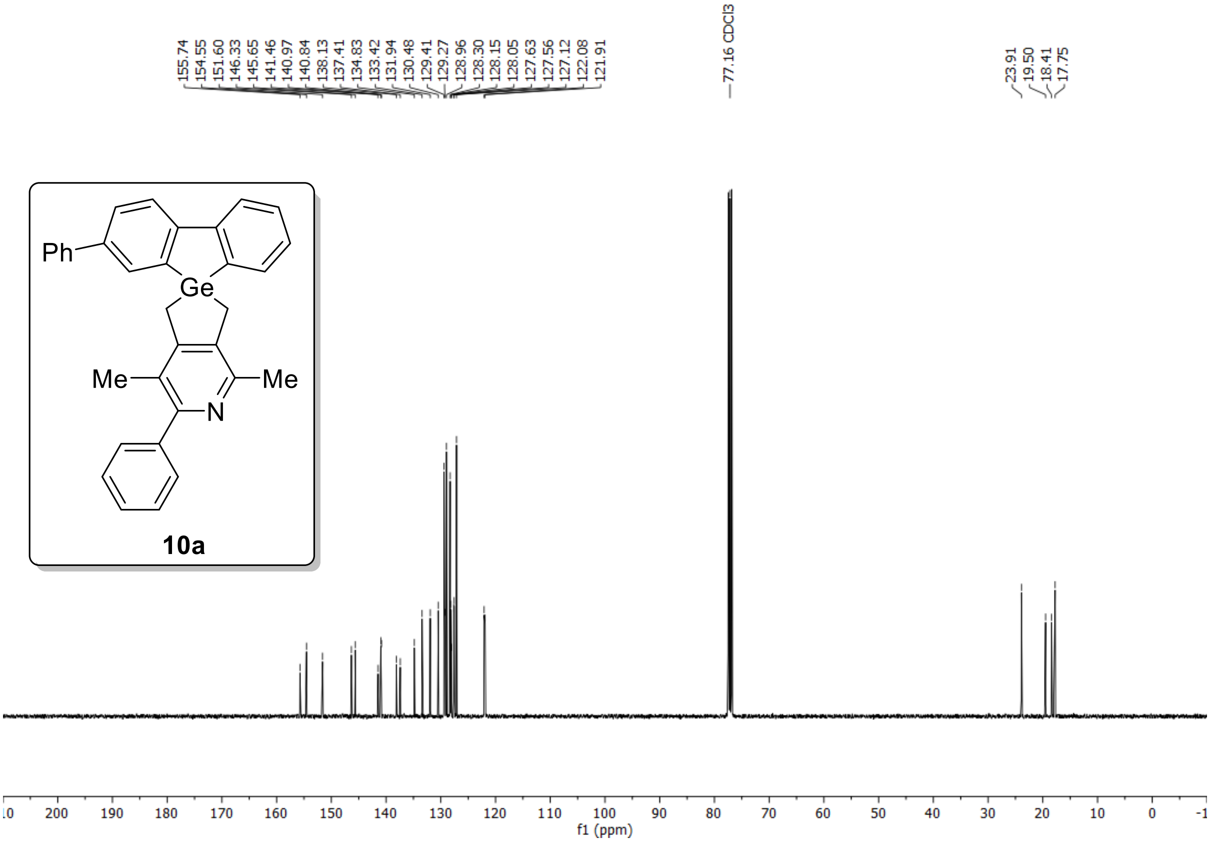
155.74 154.55 151.60 146.33 145.65 141.46 140.97 140.84 138.13 137.41 134.83 133.42 131.94 130.48 129.41 129.27 128.96 128.30 128.15 128.05 127.63 127.56 127.12 122.08 121.91
77.16 CDCl3
23.91 19.50 18.41 17.75
Ph
Ge
Me
Me
N
10a
f1 (ppm)

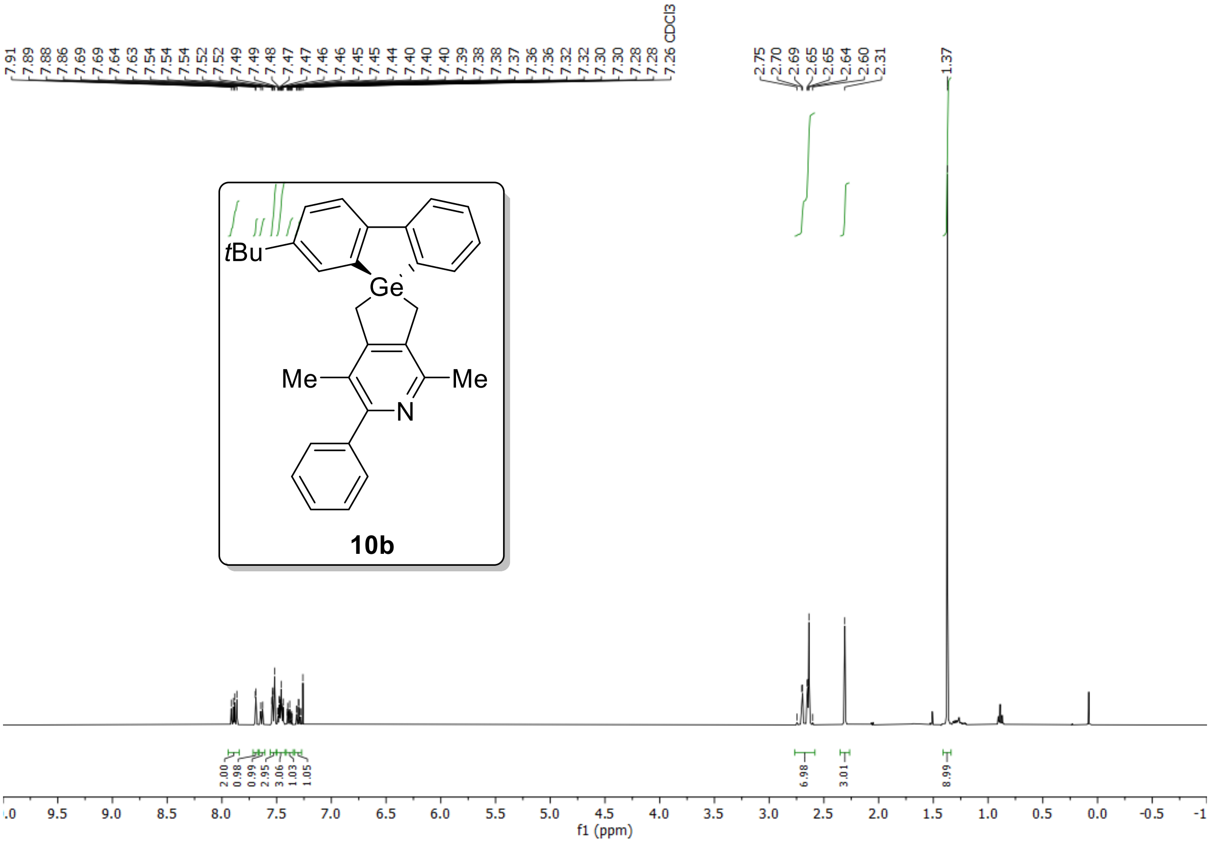
7.91 7.89 7.88 7.86 7.69 7.69 7.64 7.63 7.54 7.54 7.54 7.54 7.52 7.52 7.49 7.49 7.48 7.47 7.47 7.46 7.46 7.45 7.45 7.44 7.40 7.40 7.39 7.38 7.38 7.37 7.36 7.36 7.32 7.32 7.30 7.30 7.28 7.28 7.26 CDCl3
2.75 2.70 2.69 2.65 2.65 2.64 2.60 2.31
1.37
tBu
Ge
Me
Me
N
10b
2.00 0.98 0.99 2.95 3.06 1.03 1.05
6.98
3.01
8.99
f1 (ppm)

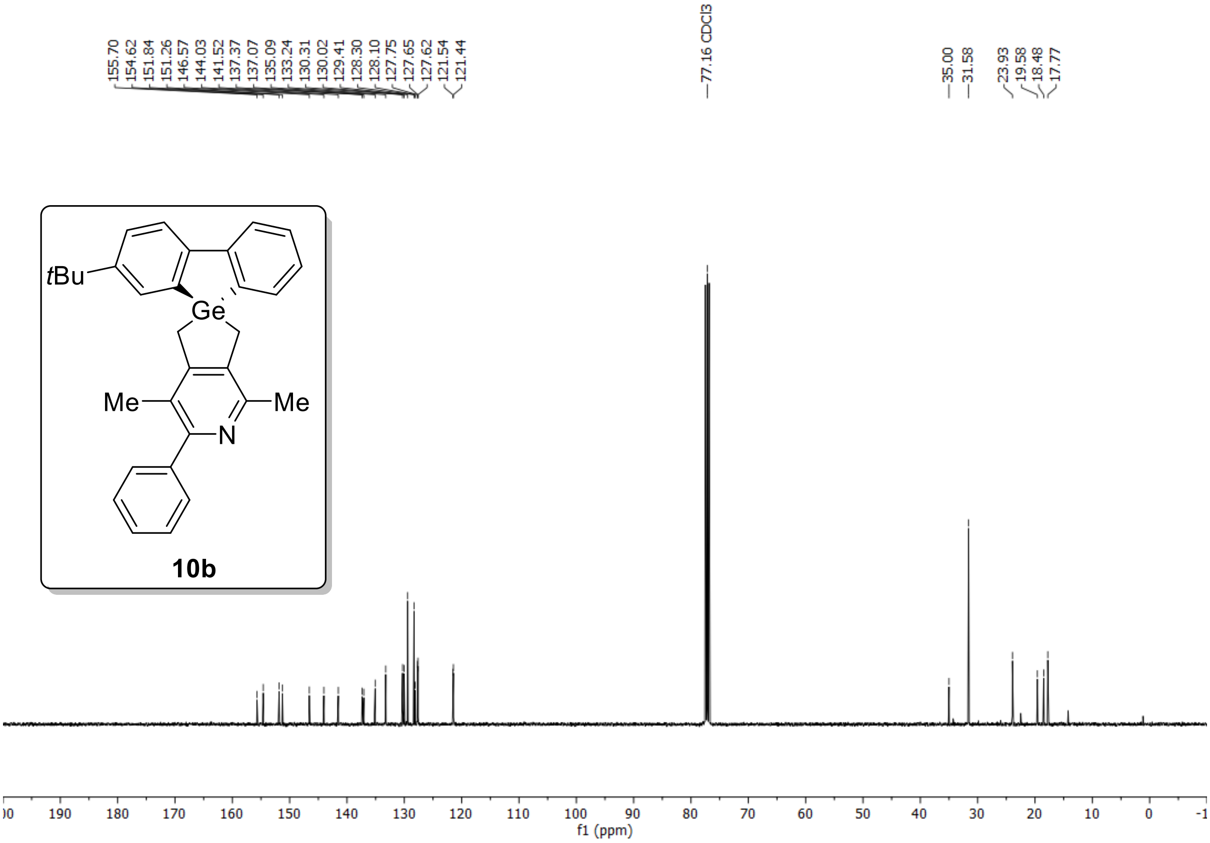
155.70 154.62 151.84 151.26 146.57 144.03 141.52 137.37 137.07 135.09 133.24 130.31 130.02 129.41 128.30 128.10 127.75 127.65 127.62 121.54 121.44
77.16 CDCl3
35.00 31.58
23.93 19.58 18.48 17.77
tBu
Ge
Me
Me
N
10b
f1 (ppm)

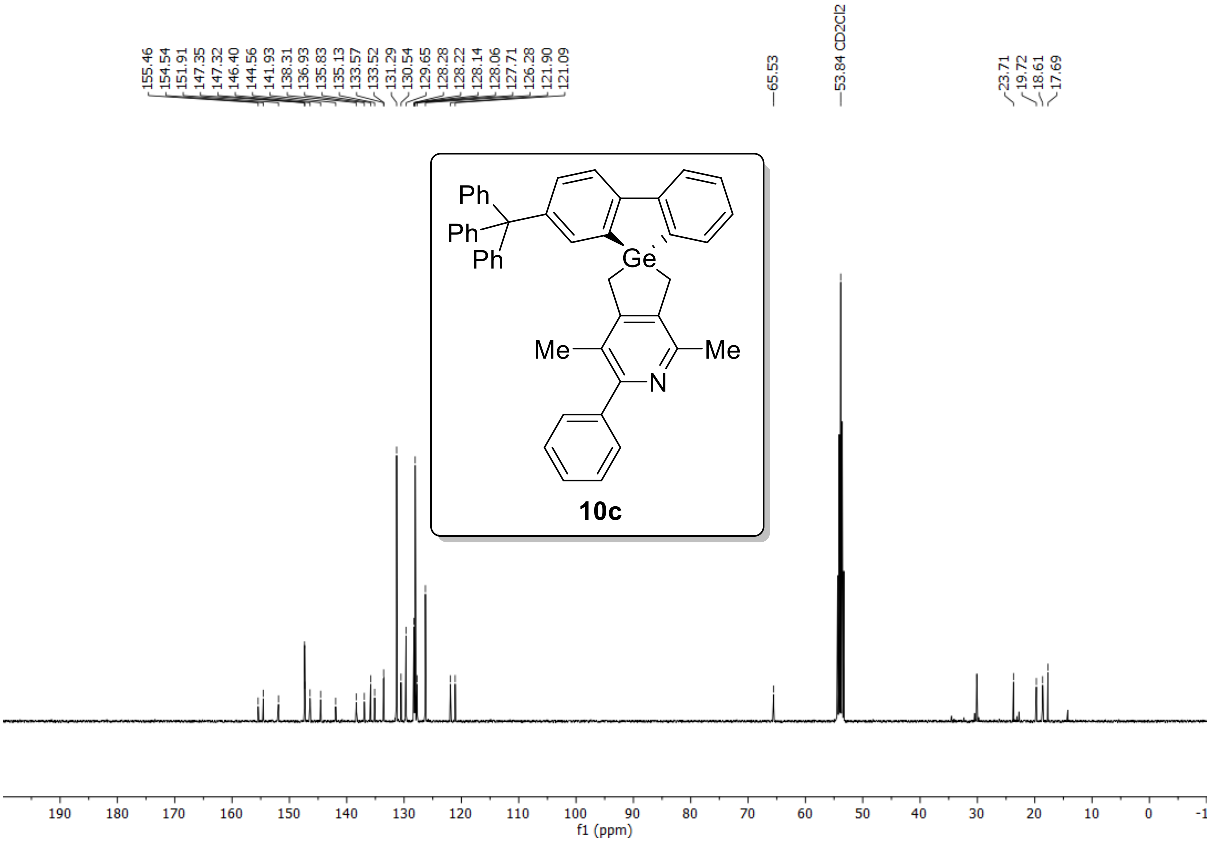
155.46
154.54
151.91
147.35
147.32
146.40
144.56
141.93
138.31
136.93
135.83
135.13
133.57
133.52
131.29
130.54
129.65
128.28
128.22
128.14
128.06
127.71
126.28
121.90
121.09
65.53
53.84 CD2Cl2
23.71
19.72
18.61
17.69
Ph
Ph
Ph
Ge
Me
Me
N
10c
190
180
170
160
150
140
130
120
110
100
90
80
70
60
50
40
30
20
10
0
-1
f1 (ppm)

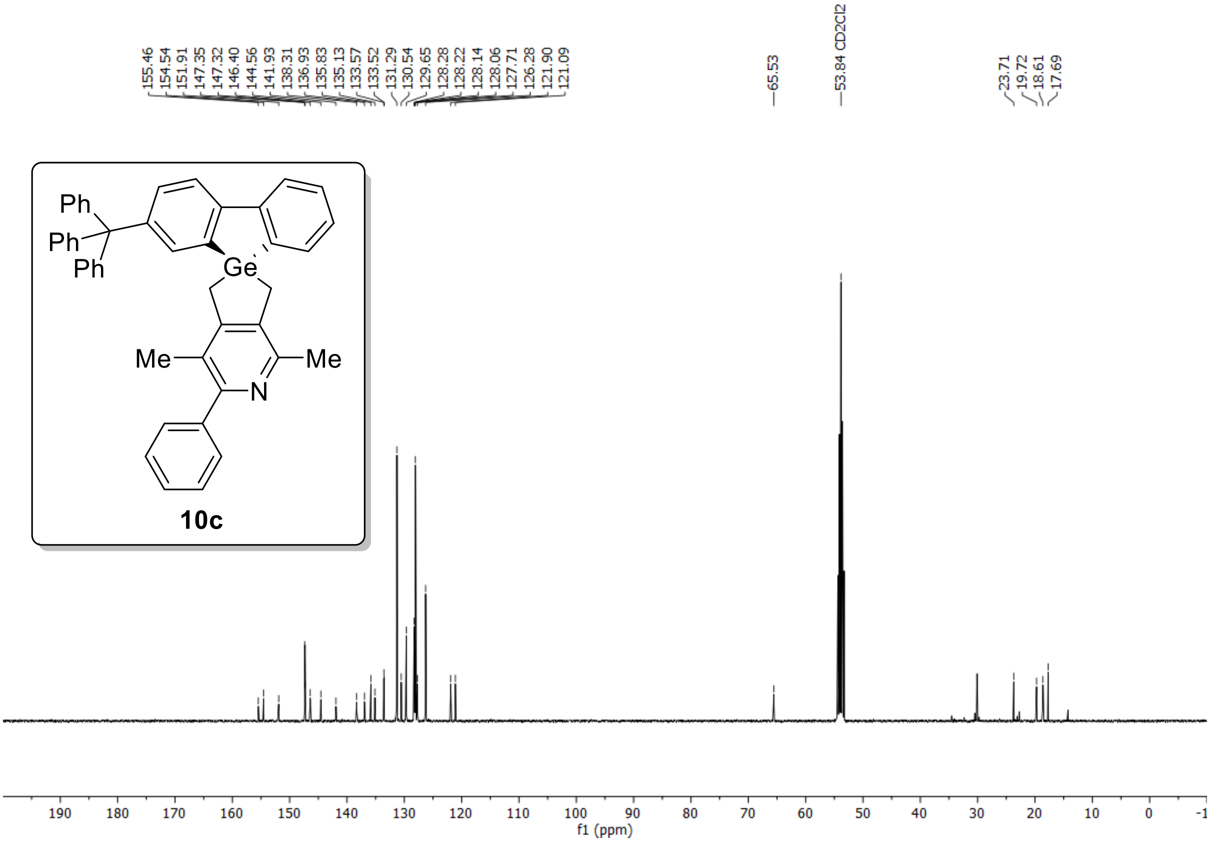
155.46
154.54
151.91
147.35
147.32
146.40
144.56
141.93
138.31
136.93
135.83
135.13
133.57
133.52
131.29
130.54
129.65
128.28
128.22
128.14
128.06
127.71
126.28
121.90
121.09
65.53
53.84 CD2Cl2
23.71
19.72
18.61
17.69
Ph
Ph
Ph
Ge
Me
Me
N
10c
190
180
170
160
150
140
130
120
110
100
90
80
70
60
50
40
30
20
10
0
-1
f1 (ppm)

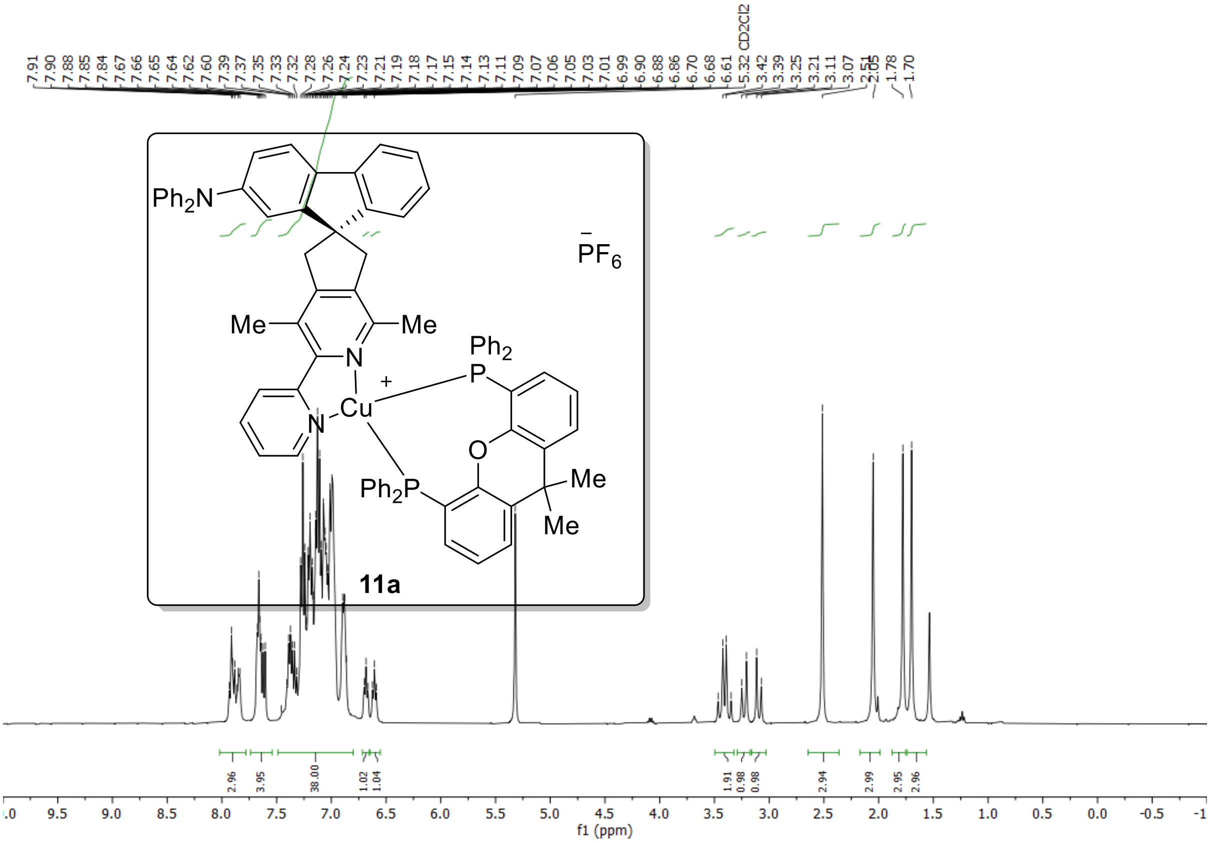

7.91 7.90 7.88 7.85 7.84 7.67 7.66 7.65 7.64 7.62 7.60 7.39 7.37 7.35 7.33 7.32 7.28 7.26 7.24 7.23 7.21 7.19 7.18 7.17 7.15 7.14 7.13 7.11 7.09 7.07 7.06 7.05 7.03 7.01 6.99 6.90 6.88 6.86 6.70 6.68 6.61 CD2Cl2 5.32 3.42 3.39 3.25 3.21 3.11 3.07 2.51 2.05 1.78 1.70
Ph2N
PF6
Me
Me
Ph2
P
N
+
Cu
N
O
Ph2P
Me
Me
11a
2.96 3.95 38.00 1.02 1.04 1.91 0.98 0.98 2.94 2.99 2.95 2.96
f1 (ppm)


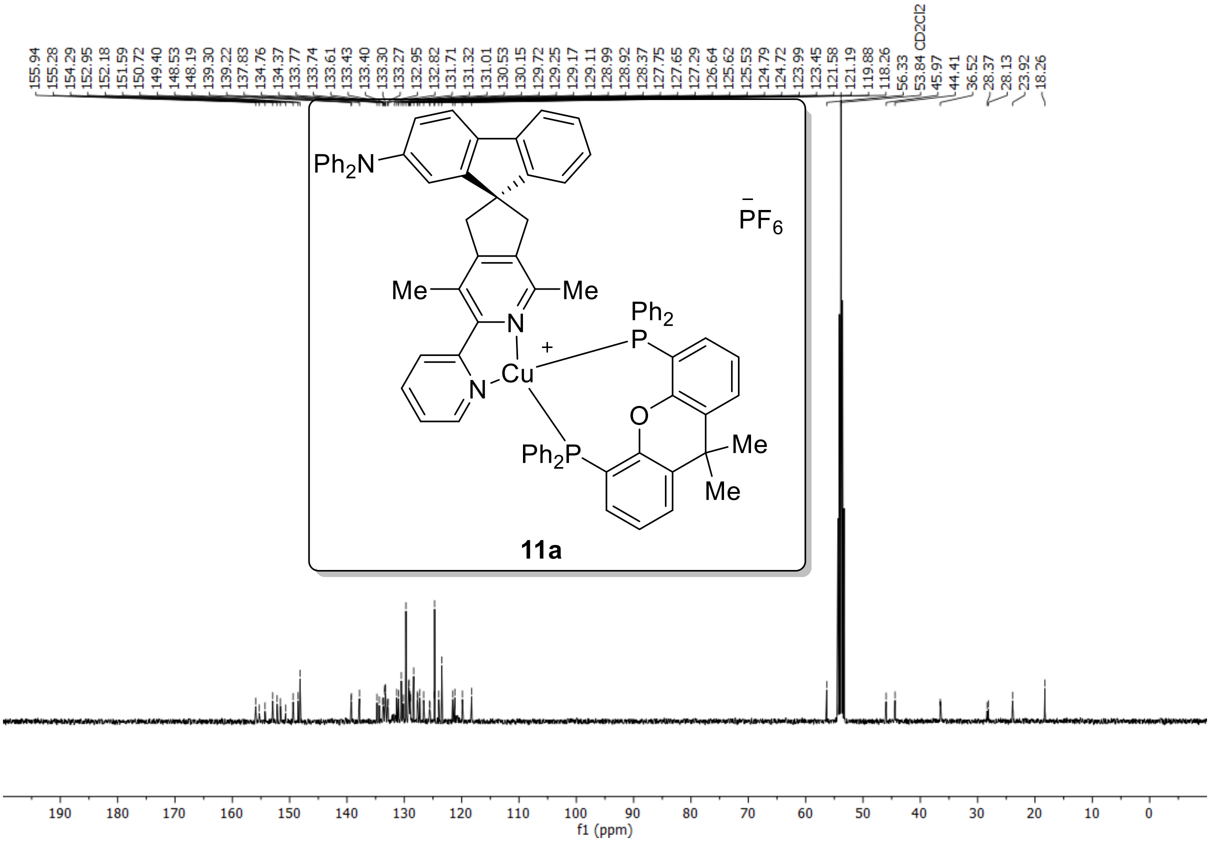

155.94 155.28 154.29 152.95 152.18 151.59 150.72 149.40 148.53 148.19 139.30 139.22 137.83 134.76 134.37 133.77 133.74 133.61 133.43 133.40 133.30 133.27 132.95 132.82 131.71 131.32 131.01 130.53 130.15 129.72 129.25 129.17 129.11 128.99 128.92 128.37 127.75 127.65 127.29 126.64 125.62 125.53 124.79 124.72 123.99 123.45 121.58 121.19 119.88 118.26 56.33 53.84 CD2Cl2 45.97 44.41 36.52 28.37 28.13 23.92 18.26
Ph2N
PF6
Me
Me
Ph2
P
N
+
Cu
N
O
Ph2P
Me
Me
11a
f1 (ppm)

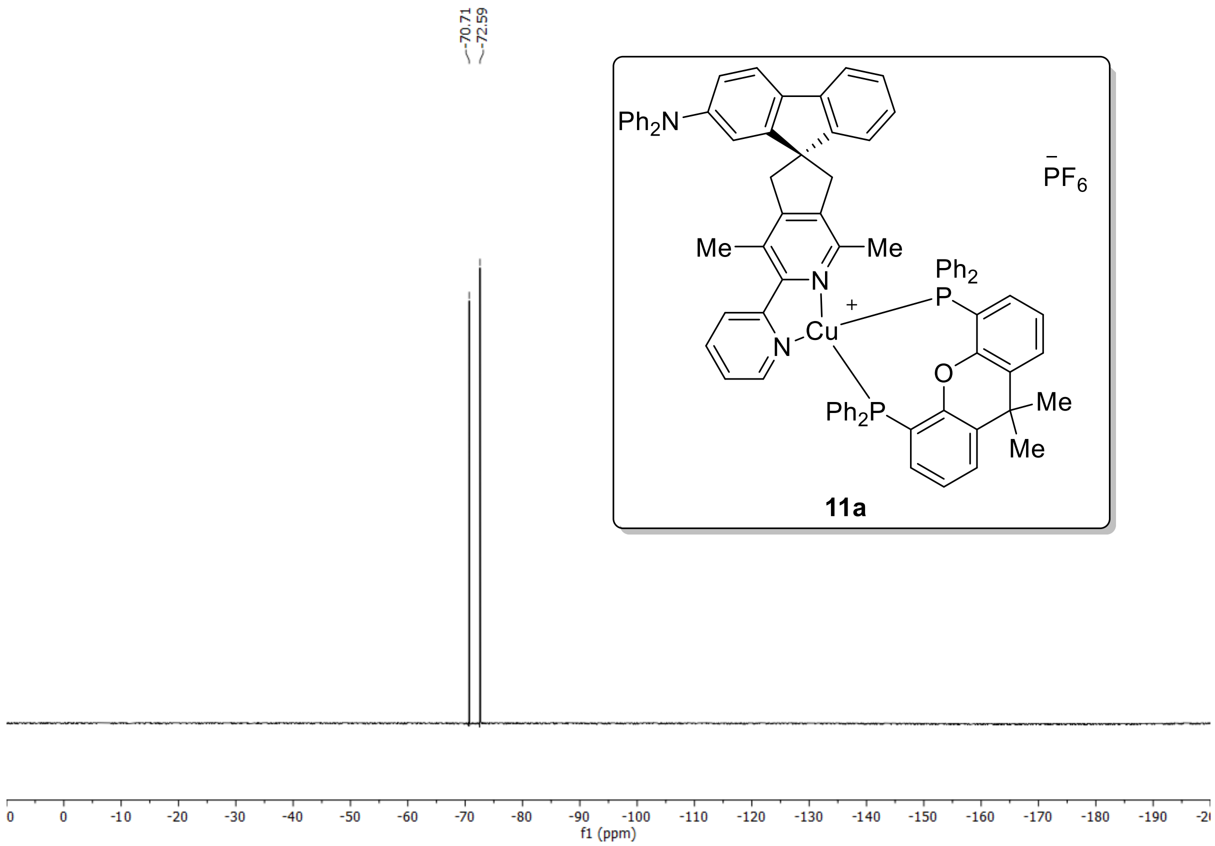
-70.71
-72.59
Ph2N
PF6
Me
Me
Ph2
P
N
Cu
+
N
O
Ph2P
Me
Me
11a
f1 (ppm)

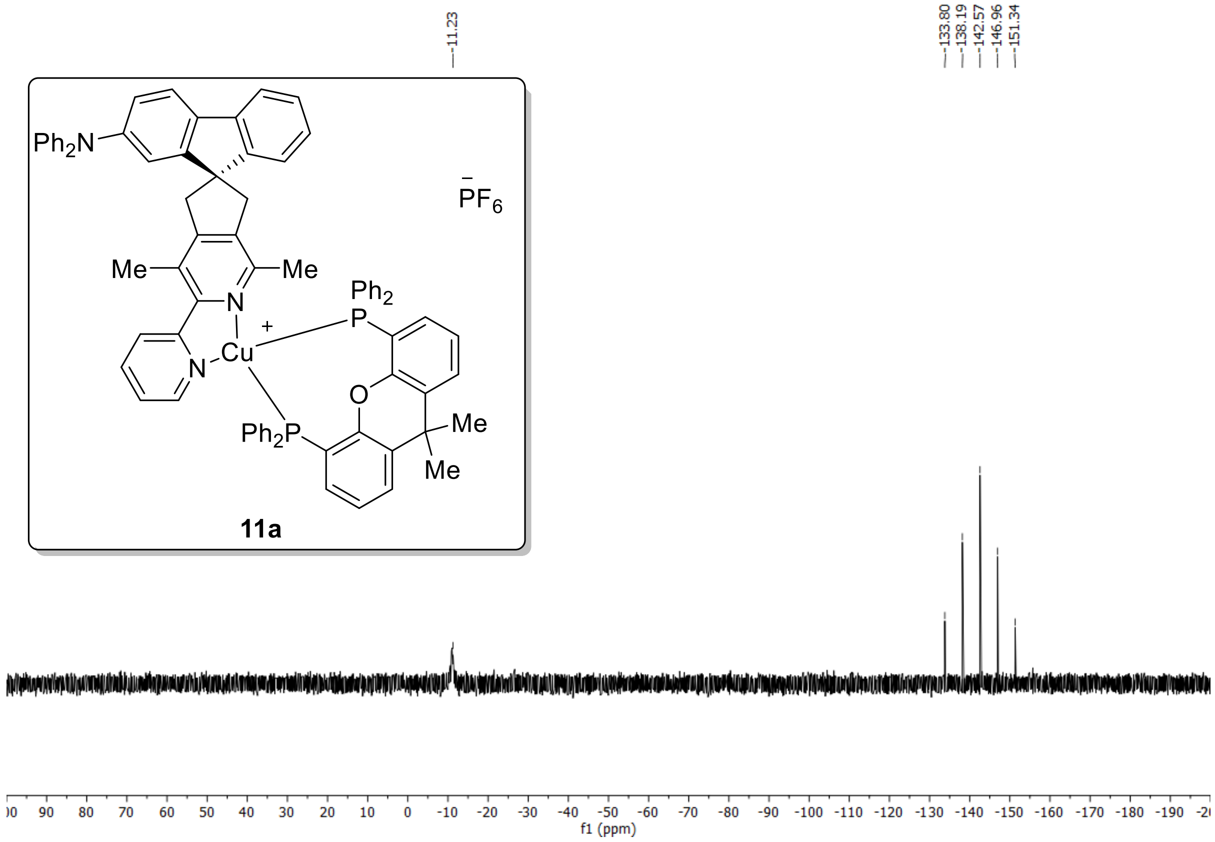
-11.23
-133.80
-138.19
-142.57
-146.96
-151.34
Ph2N
PF6
Me
Me
Ph2
P
N
Cu
+
N
O
Ph2P
Me
Me
11a
f1 (ppm)

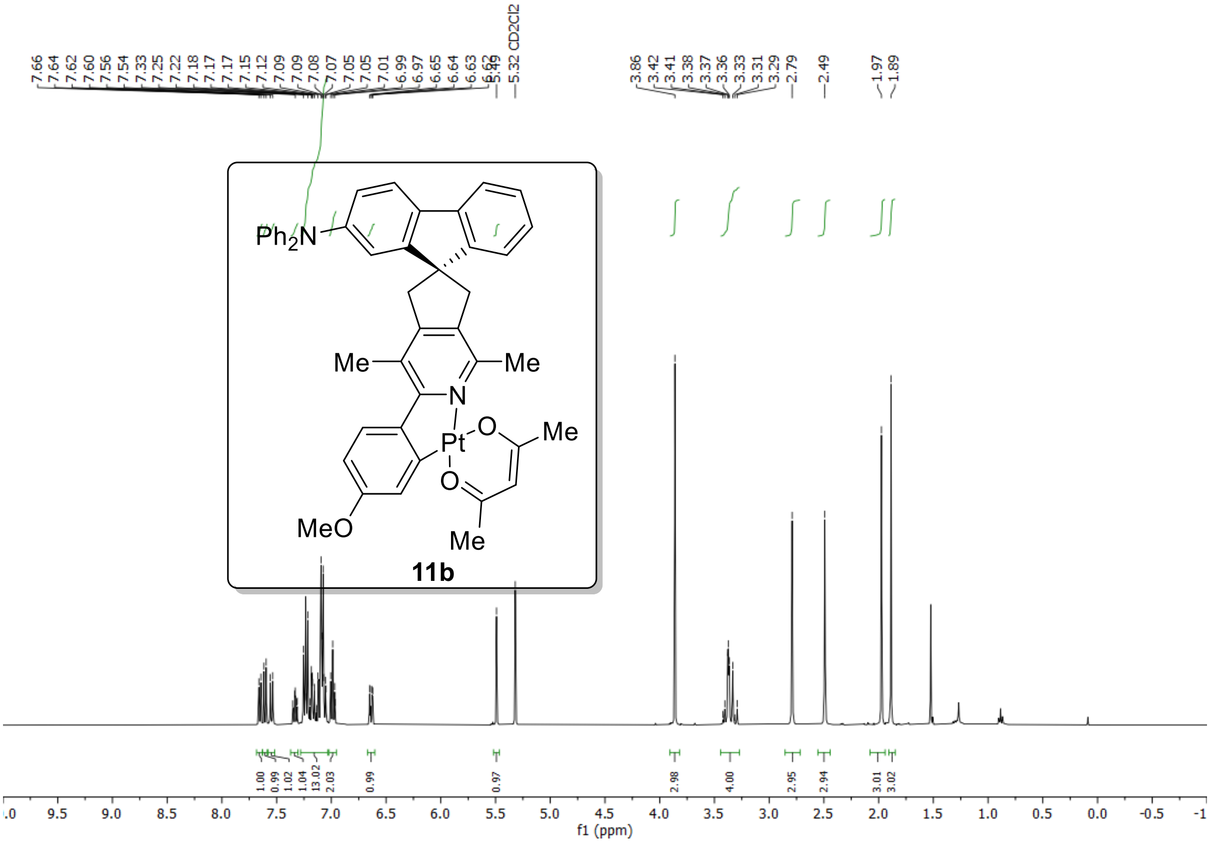
7.66 7.64 7.62 7.60 7.56 7.54 7.33 7.25 7.22 7.18 7.17 7.17 7.15 7.12 7.09 7.09 7.08 7.07 7.05 7.05 7.01 6.99 6.97 6.65 6.64 6.63 6.62 5.49
5.32 CD2Cl2
3.86 3.42 3.41 3.38 3.37 3.36 3.33 3.31 3.29 2.79 2.49 1.97 1.89
Ph2N
Me
Me
N
Pt
O
O
Me
MeO
Me
11b
1.00 0.99 1.02 1.04 13.02 2.03 0.99 0.97 2.98 4.00 2.95 2.94 3.01 3.02
f1 (ppm)

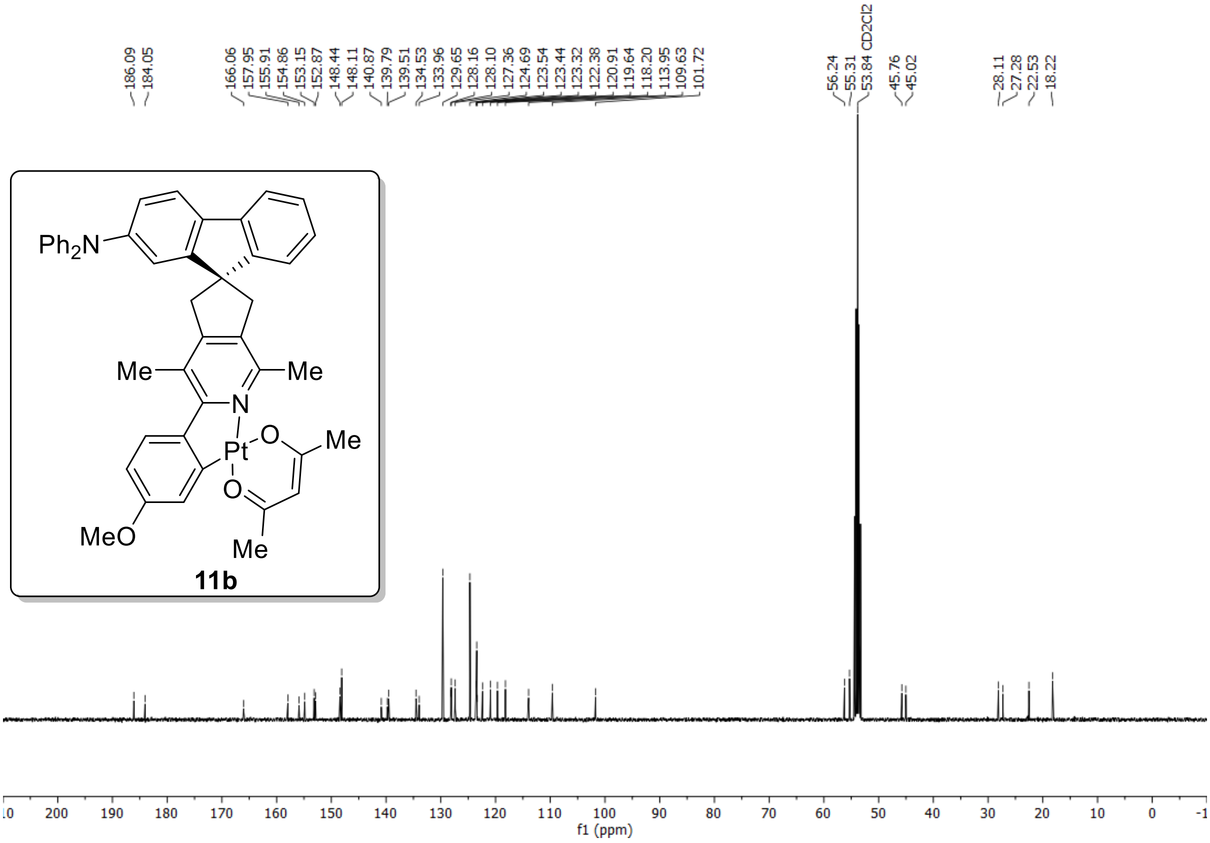
186.09 184.05 166.06 157.95 155.91 154.86 153.15 152.87 148.44 148.11 140.87 139.79 139.51 134.53 133.96 129.65 128.16 128.10 127.36 124.69 123.54 123.44 123.32 122.38 120.91 119.64 118.20 113.95 109.63 101.72
56.24 55.31
53.84 CD2Cl2
45.76 45.02 28.11 27.28 22.53 18.22
Ph2N
Me
Me
N
Pt
O
O
Me
MeO
Me
11b
f1 (ppm)

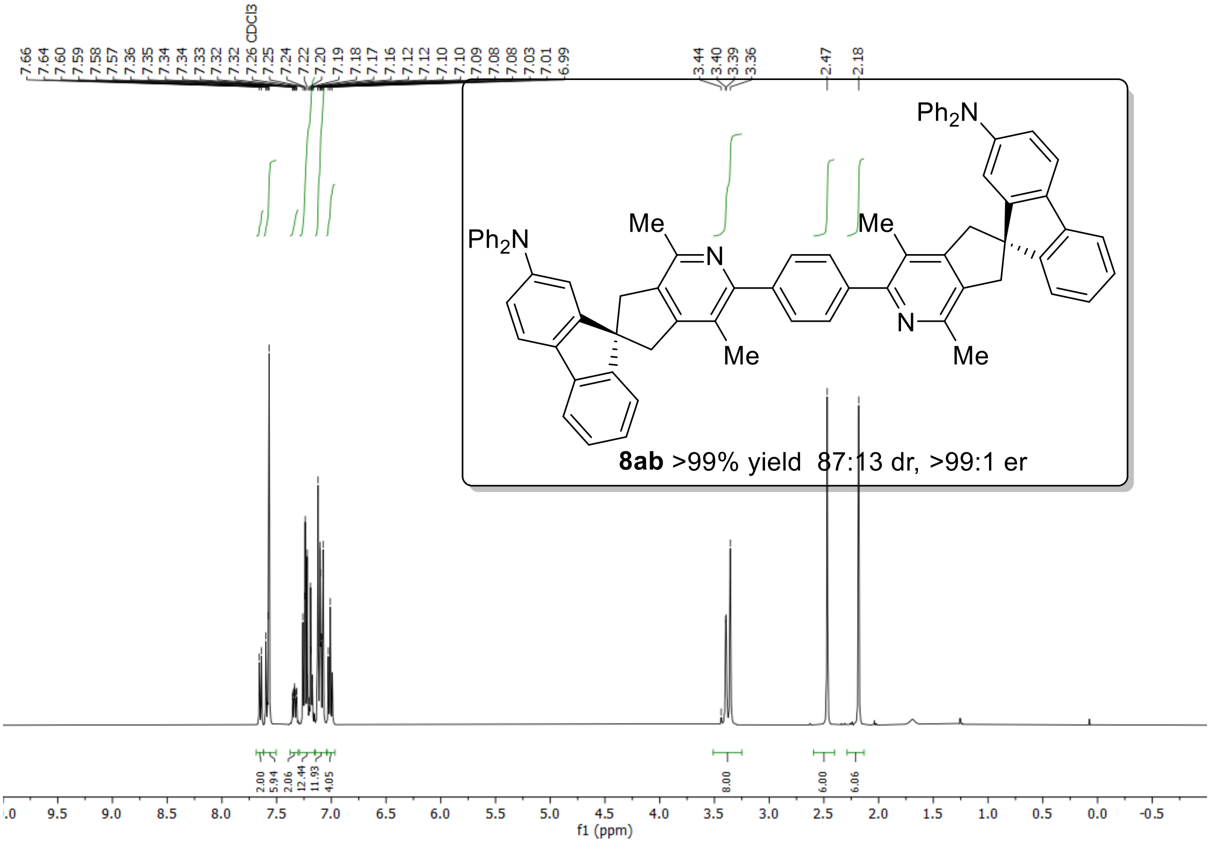
Ph2N
Me
N
Me
Me
N
Me
Ph2N
8ab >99% yield 87:13 dr, >99:1 er
7.26 CDCl3
3.44
3.40
3.39
3.36
2.47
2.18
2.00
5.94
2.06
12.44
11.93
4.05
8.00
6.00
6.06
f1 (ppm)

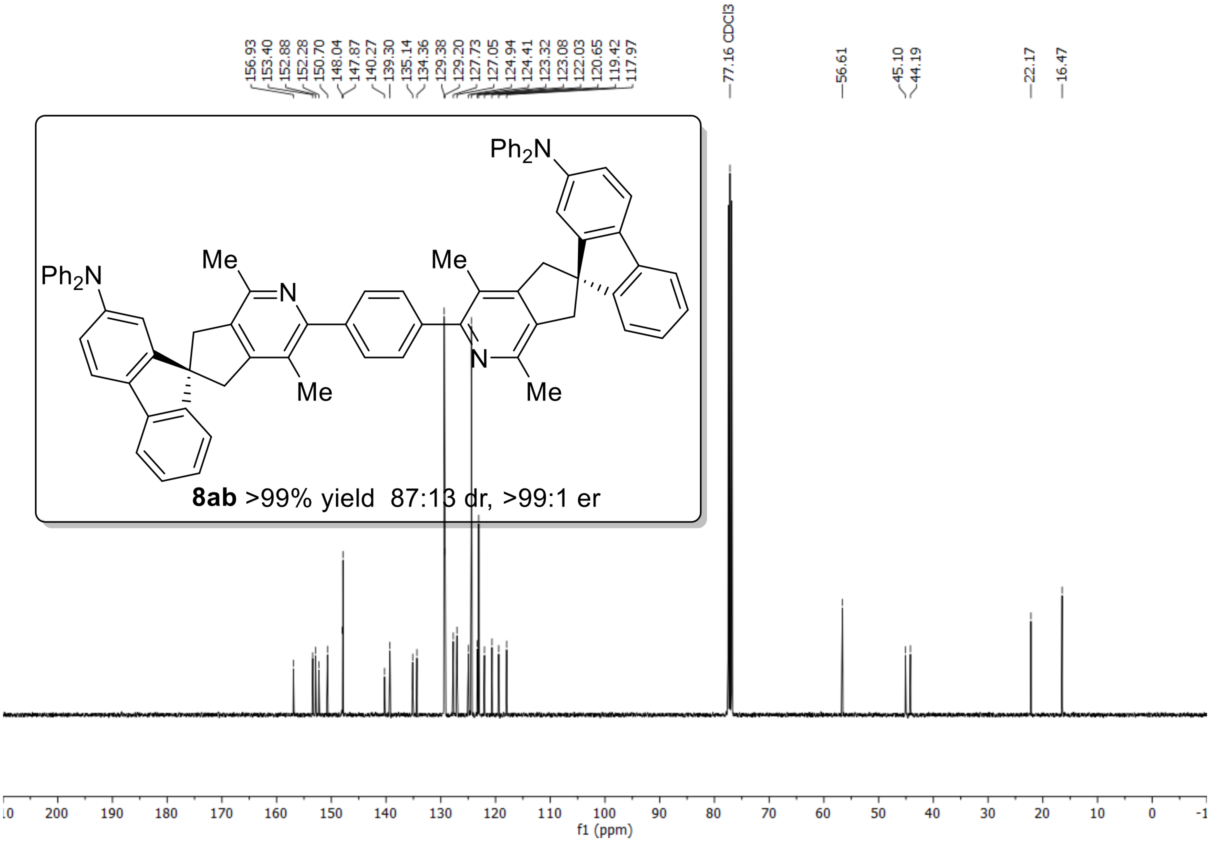
Ph2N
Me
N
Me
Me
N
Me
Ph2N
8ab >99% yield 87:13 dr, >99:1 er
156.93
153.40
152.88
152.28
150.70
148.04
147.87
140.27
139.30
135.14
134.36
129.38
129.20
127.73
127.05
124.94
124.41
123.32
123.08
122.03
120.65
119.42
117.97
77.16 CDCl3
56.61
45.10
44.19
22.17
16.47
f1 (ppm)

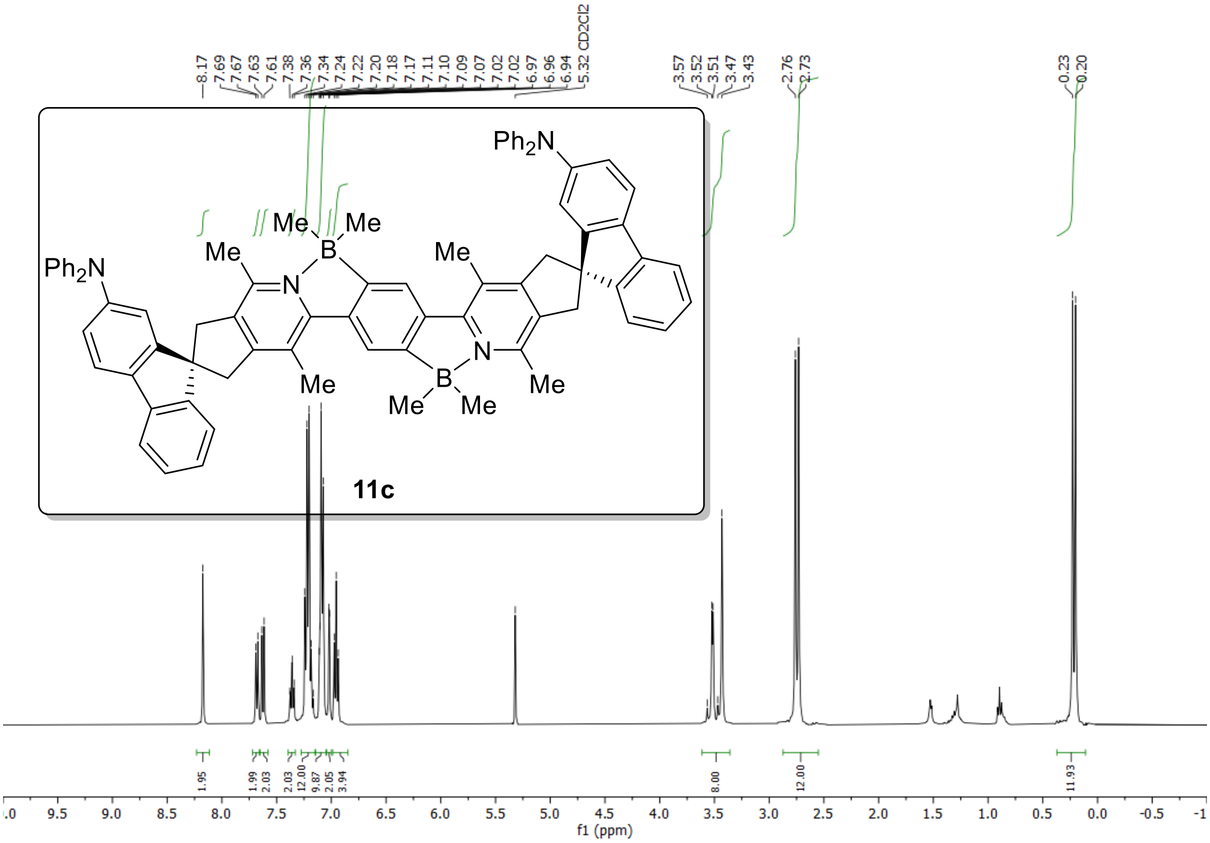

8.17
7.69
7.67
7.63
7.61
7.38
7.36
7.34
7.24
7.22
7.20
7.18
7.17
7.11
7.10
7.09
7.07
7.02
7.02
6.97
6.96
6.94
5.32 CD2Cl2
3.57
3.52
3.51
3.47
3.43
2.76
2.73
0.23
0.20
Ph2N
Me
Me
Me
B
N
Me
11c
1.95
1.99
2.03
2.03
12.00
9.87
2.05
3.94
8.00
12.00
11.93
f1 (ppm)


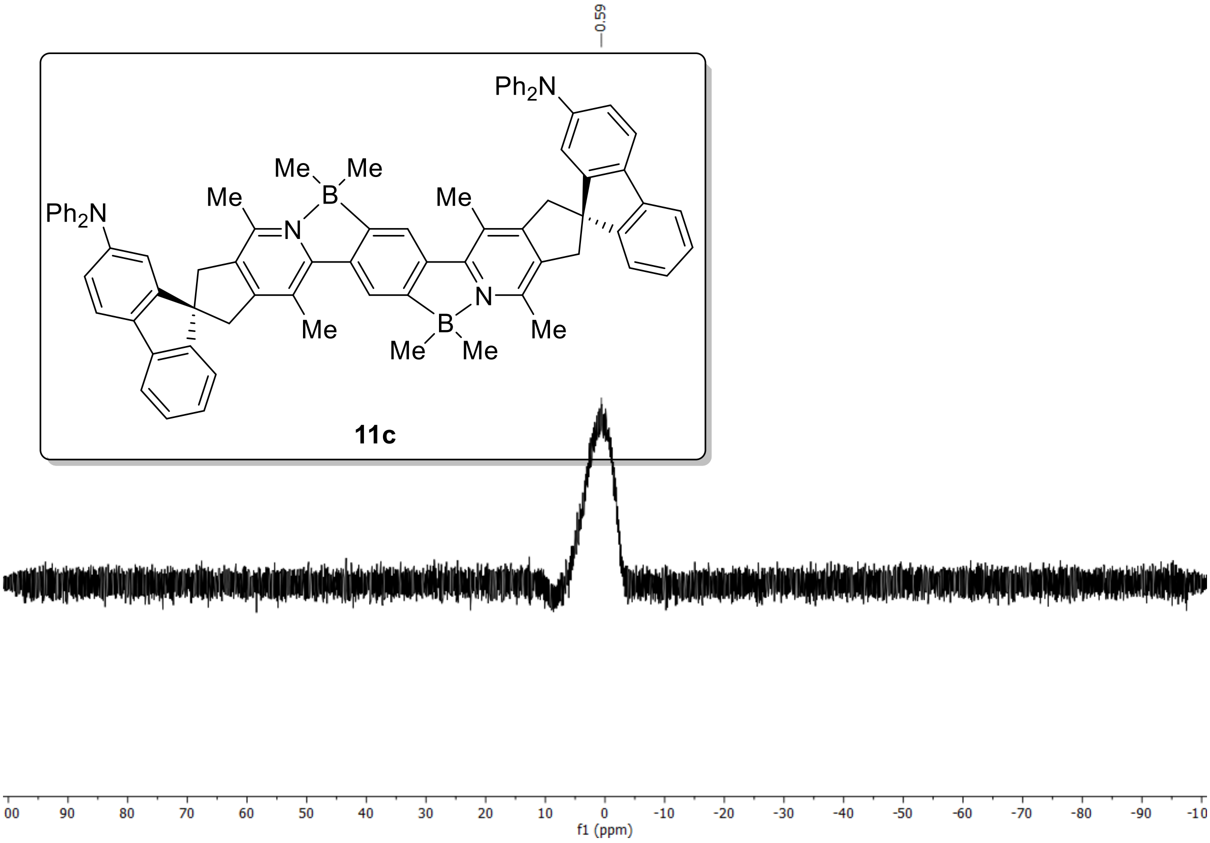

0.59
Ph2N
Me
B
N
11c
f1 (ppm)

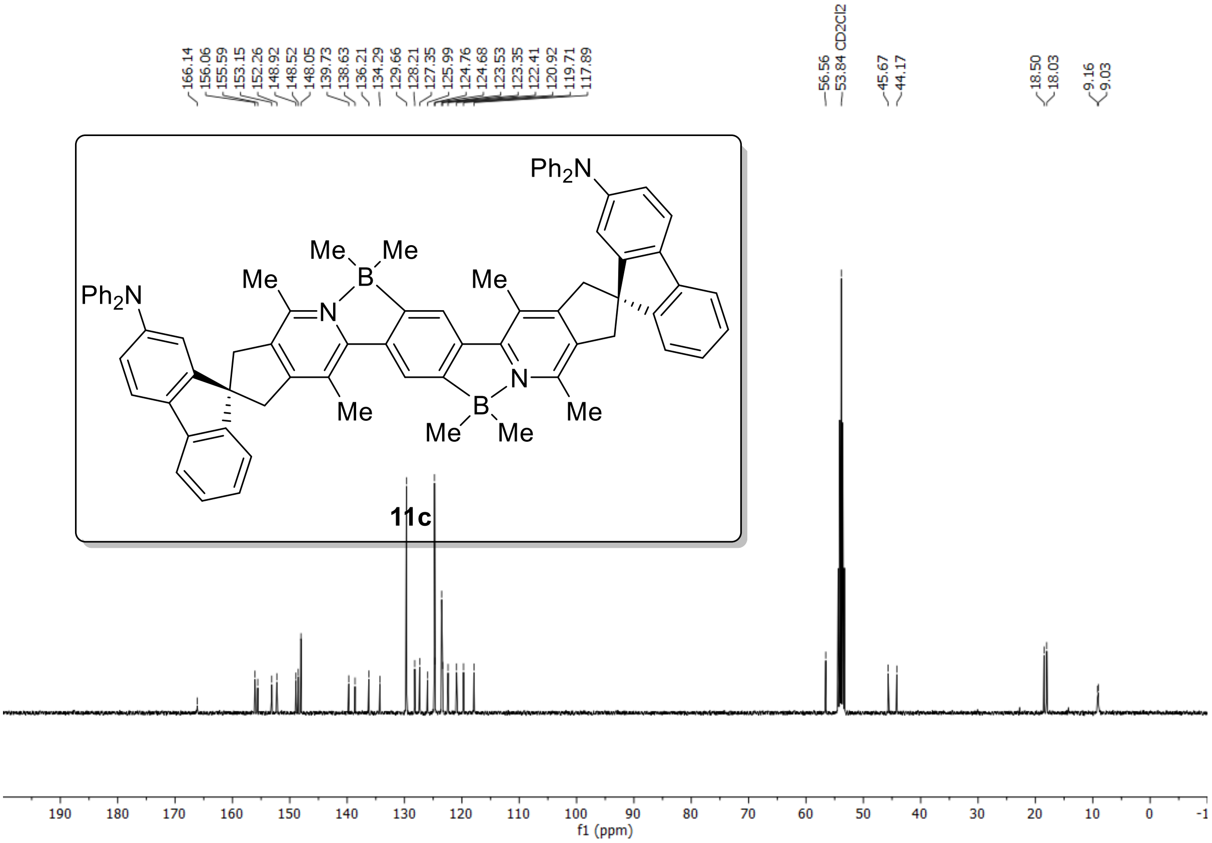
166.14
156.06
155.59
153.15
152.26
148.92
148.52
148.05
139.73
138.63
136.21
134.29
129.66
128.21
127.35
125.99
124.76
124.68
123.53
123.35
122.41
120.92
119.71
117.89
56.56
53.84 CD2Cl2
45.67
44.17
18.50
18.03
9.16
9.03
Ph2N
Me
Me
B
N
Me
Me
Me
N
B
Me
Me
Me
Ph2N
11c
190 180 170 160 150 140 130 120 110 100 90 80 70 60 50 40 30 20 10 0 -1
f1 (ppm)

## Supplementary references


1. Diesel, J., Finogenova, A. M. & Cramer, N. Nickel-Catalyzed Enantioselective Pyridone C-H Functionalizations Enabled by a Bulky N- Heterocyclic Carbene Ligand. *J. Am. Chem. Soc.* 140, 4489–4493 (2018).

2. Cao, Y. X., Wodrich, M. D. & Cramer, N. Nickel-catalyzed direct stereoselective α-allylation of ketones with non-conjugated dienes. *Nat. Commun*. 14, 7640 (2023).

3. Braconi, E. & Cramer, N. A Chiral Naphthyridine Diimine Ligand Enables Nickel-Catalyzed Asymmetric Alkylidenecyclopropanations. *Angew. Chem. In. Ed*. 59, 16425–16429 (2020).

4. Diesel, J., Grosheva, D., Kodama, S. & Cramer. N. A Bulky Chiral N-Heterocyclic Carbene Nickel Catalyst Enables Enantioselective C−H Functionalizations of Indoles and Pyrroles. *Angewandte Chemie* 131, 11160–11164 (2019).

5. Cai, Y., Zhang, J. W., Li, F., Liu, J. M. & Shi, S. L. Nickel/N-Heterocyclic Carbene Complex-Catalyzed Enantioselective Redox-Neutral Coupling of Benzyl Alcohols and Alkynes to Allylic Alcohols. *ACS. Catal.* 9, 1–6 (2019).

6. Martinez, C. R. & Iverson, B. L. Rethinking the term "π-stacking". *Chem. Sci.* 3, 2191–2201 (2012).

7. Poater, A., Ragone, F., Giudice, S., Costabile, C., Dorta, R., Nolan, S. P. & Cavallo, L. Thermodynamics of N-Heterocyclic Carbene Dimerization: The Balance of Sterics and Electronics. *Organometallics.* 27, 2679−2681 (2008).

8. Poater, A., Ragone, F., Mariz, R., Dorta, R. & Cavallo. L. Comparing the Enantioselective Power of Steric and Electrostatic Effects inTransition-Metal-Catalyzed Asymmetric Synthesis. *Chem. Eur. J.* 16, 14348−14353 (2010).

9. Sprick, R. S. *et al.* Visible-Light-Driven Hydrogen Evolution Using Planarized Conjugated Polymer Photocatalysts. *Angew. Chem. In. Ed*. 55, 1792–1796 (2016).

10. Zhou, Z. *et al.* Glucopyranoside-Functionalized NHCs-Pd(II)-PEPPSI Complexes: Anomeric Isomerism Controlled and Catalytic Activity in Aqueous Suzuki Reaction. *Catal Letters* 152, 838–847 (2022).

11. Wang, M. *et al.* Large femtosecond two-photon absorption cross sections of fullerosome vesicle nanostructures derived from a highly photoresponsive amphiphilic C 60-light-harvesting fluorene dyad. *Journal of Physical Chemistry C* 115, 18552–18559 (2011).

12. Akhmetov, V., Feofanov, M., Sharapa, D. I. & Amsharov, K. Alumina-Mediated π-Activation of Alkynes. *J Am Chem Soc* 143, 15420–15426 (2021).

13. Grisorio, R. *et al.* Aryl 5-substitution of a phenyl-pyridine based ligand as a viable way to influence the opto-electronic properties of bis-cyclometalated Ir(iii) heteroleptic complexes. *Dalton Transactions* 42, 8939–8950 (2013).

14. Topchiy, M. A., Asachenko, A. F. & Nechaev, M. S. Solvent-free Buchwald-Hartwig reaction of aryl and heteroaryl halides with secondary amines. *Eur. J. Org. Chem.* 2014, 3319–3322 (2014).

15. Liu, C., Rao, X., Song, X., Qiu, J. & Jin, Z. Palladium-catalyzed ligand-free and aqueous Suzuki reaction for the construction of (hetero)aryl-substituted triphenylamine derivatives. *RSC Adv* 3, 526–531 (2013).

16. Li, X., Teng, Y., Feng, F., Hu, Q. & Yuan, Z. Aqueous Suzuki-Miyaura Reaction with 0.6 Equiv. of Base: Green and Efficient Access to Biaryls and Unsymmetrical Terphenyls. *ChemistrySelect* 3, 6022–6027 (2018).

17. Li, Z. Y., Chaminda Lakmal, H. H. & Cui, X. Enabling Catalytic Arene C-H Amidomethylation via Bis(tosylamido)methane as a Sustainable Formaldimine Releaser. *Org Lett.* 21, 3735–3740 (2019).

18. Kagabu, S. & Mizoguchi, S. A Unique Synthetic Method for pyridine-ring Containing Ter-, Quarter-, and Quinquearyl, and Vinylogues by Thermolysis of 2,2'-Dichlorocyclopropylmethyleneamines. *Synthesis*. 372-376 (1996).
19. Klappa, J. J., Rich, A. E. & McNeill, K. One-step synthesis of 3,5-disubstituted-2-pyridylpyrroles from the condensation of 1,3-diones and 2-(aminomethyl)pyridine. *Org Lett* 4, 435–437 (2002).
20. Zu, B., Guo, Y. & He, C. Catalytic Enantioselective Construction of Chiroptical Boron-Stereogenic Compounds. *J. Am. Chem. Soc*. 143, 16302–16310 (2021).
21. D. M. Chen, Q. Qin, Z. B. Sun, Q. Peng, C. H. Zhao. Synthesis and properties of B, N-bridged p-terphenyls. *Chemical Communications* 50, 782–784 (2014).
22. Keller, S. *et al*. Shine bright or live long: substituent effects in [Cu(N^N) (P^P)]+-based light-emitting electrochemical cells where N^N is a 6- substituted 2,2'-bipyridine. *J. Mater. Chem. C*. 4, 3857–3871 (2016).
23. Brooks, J., Babayan, Y., Lamansky, S., Djurovich, P. I., Tsyba, I., Bau, R. & Thompson, M. E. Synthesis and Characterization of Phosphorescent CyclometalatedPlatinum Complexes. *Inorg. Chem*. 41, 3055– 3066 (2002).
24. Iglesias, M. J., Blandez, J. F., Fructos, M. R., Prieto, A., Álvarez, E., Belderrain, T. R. & Nicasio, M. C. Synthesis, Structural Characterization, and Catalytic Activity of IPrNi(styrene)2 Amination of Aryl Tosylates. *Organometallics.* 31, 6312−6316 (2012).
25. Stolley, R. M., Duong, H. A. & Louie, J. Mechanistic evaluation of the Ni(IPr)2-catalyzed cycloaddition of alkynes and nitriles to afford pyridines: Evidence for the formation of a key η1-Ni(IPr)2(RCN) intermediate. *Organometallics*. 32, 4952–4960 (2013).
